# Supplementary material for: An Explanation of Exceptions from Chargaff’s Second Parity Rule/Strand Symmetry of DNA Molecules
Source: Genes (Basel). 2022 Oct 23;13(11):1929. doi: 10.3390/genes13111929 (PMC9689577; doi:10.3390/genes13111929)
Supplement: Supplementary file 1 [file genes-13-01929-s001.zip › Supplementary Information_24_10_2022-10.25.pdf]

Supplementary Information

Supplementary Table S1. Relative frequencies for A-T and G-C base pairs from human chromosomes. Frequencies are very nearly in accordance with CSPR (Figure 3A). The differences to CSPR are very small, not exceeding 0.5%. Slightly higher values are for T vs. A from chromosomes 8 and 22. Also larger values are for G with respect to C, except in Y chromosome, where they are comparable.

Chr.	Total bp	A%	T%	G%	C%	Total %
**1.**	248,956,422	26.94	27.01	19.33	19.30	92.58
**2.**	242,193,529	29.64	29.72	20.01	19.95	99.32
**3.**	198,295,559	30.10	30.17	19.84	19.79	99.9
**4.**	190,214,555	30.79	30.82	19.10	19.05	99.76
**5.**	181,538,259	30.13	30.27	19.76	19.68	99.84
**6.**	170,805,979	30.06	30.08	19.74	19.70	99.58
**7.**	159,345,973	29.53	29.63	20.32	20.28	99.76
**8.**	145,138,636	29.86	29.83	20.05	20.00	99.74
**9.**	138,394,717	25.82	25.86	18.19	18.14	88.01
**10.**	133,797,422	29.06	29.17	20.72	20.66	99.61
**11.**	135,086,622	29.08	29.14	20.70	20.66	99.58
**12.**	133,275,309	29.54	29.63	20.40	20.33	99.9
**13.**	114,364,328	26.27	26.37	16.56	16.47	85.67
**14.**	107,043,718	24.92	25.14	17.34	17.21	84.61
**15.**	101,991,189	24.03	24.07	17.48	17.41	82.99
**16.**	90,338,345	24.97	25.21	20.26	20.12	90.56
**17.**	83,257,441	27.19	27.27	22.64	22.49	99.59
**18.**	80,373,285	29.92	30.09	19.98	19.65	99.64
**19.**	58,617,616	25.83	26.07	23.99	23.81	99.7
**20.**	64,444,167	27.73	28.03	21.87	21.59	99.22
**21.**	46,709,983	25.31	25.38	17.61	17.52	85.82
**22.**	50,818,464	20.43	20.41	18.19	18.03	77.06
**X.**	156,040,895	29.96	30.07	19.67	19.56	99.26
**Y.**	57,227,415	13.78	13.90	9.24	9.24	46.16

Supplementary Table S2. Relative frequencies of complementary A-T and C-G base pairs from 200,000 bp concatenated sequence. 1000 randomly selected 200-bp genomic subsequence are taken from each human chromosome (Figure 3B).

Chr.	A%	T%	G%	C%	Total %
**1.**	27.52	27.45	22.2	22.44	99.61
**2.**	29.31	29.39	20.48	20.82	100
**3.**	29.48	29.71	20.21	20.6	100
**4.**	30.11	30.17	19.48	19.64	99.40
**5.**	30.10	30.44	19.70	19.47	99.71
**6.**	29.30	29.51	20.20	20.20	99.21
**7.**	29.46	29.40	20.67	20.17	99.70
**8.**	29.39	29.55	20.48	20.27	99.69
**9.**	23.43	23.94	15.34	15.62	78.33
**10.**	29.70	29.39	20.29	20.12	99.50
**11.**	28.60	28.40	21.18	21.13	99.31
**12.**	29.46	29.40	20.67	20.17	99.70
**13.**	24.10	24.13	15.14	15.03	78.40
**14.**	23.59	23.93	15.56	15.37	78.45
**15.**	28.36	29.37	19.96	18.60	96.29
**16.**	27.99	27.58	22.11	21.73	99.41
**17.**	27.54	27.77	22.29	22.19	99.79
**18.**	30.18	30.30	19.78	19.45	99.71
**19.**	21.10	21.17	25.71	24.77	92.75
**20.**	26.43	25.36	20.90	21.81	94.50
**21.**	29.86	30.10	18.70	17.90	96.56
**22.**	24.02	23.90	16.66	15.56	80.14
**X.**	29.40	29.73	20.03	19.95	99.11
**Y.**	19.76	20.73	20.33	20.61	81.43

Supplementary Table S3. Relative frequencies of complementary A-T and C-G base pairs from coding DNA in human chromosomes (see Figure 3C). Genomic data are from assembly coding DNA in human chromosomes. Relative frequencies of complementary base pairs strongly differ so that CSPR (strand symmetry) is significantly violated (see Figure 3C). It should be noted that in coding DNA of all chromosomes the frequency of A is larger than of T. The frequency of G is in most chromosomes larger than frequency of C, except in chromosomes 11, 16, 19, 20 and 22.

Chr.	Total bp	A%	T%	G%	C%	Total %
**1.**	17,202,568	26.02	24.58	24.93	24.47	100
**2.**	13,259,725	27.82	25.55	23.82	22.81	100
**3.**	10,559,026	27.03	25.35	24.15	23.47	100
**4.**	7,267,086	28.61	26.66	22.96	21.77	100
**5.**	8,095,671	27.68	25.81	23.77	22.75	100
**6.**	8,919,062	27.23	25.38	24.09	23.30	100
**7.**	8,085,329	26.26	24.64	24.68	24.42	100
**8.**	6,311,950	26.65	25.16	24.56	23.63	100
**9.**	6,696,586	25.67	23.84	25.42	25.07	100
**10.**	7,162,625	27.31	25.27	23.98	23.44	100
**11.**	10,187,352	24.87	23.74	25.6	25.79	100
**12.**	10,043,616	26.56	25.10	24.45	23.89	100
**13.**	3,029,633	28.92	27.11	22.63	21.34	100
**14.**	6,137,318	26.46	24.73	24.86	23.95	100
**15.**	6,337,119	26.66	24.88	24.63	23.83	100
**16.**	7,435,019	23.22	22.14	27.15	27.49	100
**17.**	10,435,019	23.74	22.33	26.91	27.02	100
**18.**	3,334,171	27.92	26.19	23.31	22.58	100
**19.**	10,573,294	23.07	21.04	27.67	28.23	100
**20.**	4,280,919	24.57	23.00	26.21	26.23	100
**21.**	1,937,468	25.91	24.51	25.16	24.42	100
**22.**	3,664,793	22.57	21.30	28.04	28.09	100
**X.**	6,660,403	26.87	25.28	24.27	23.58	100
**Y.**	350,406	29.21	27.90	21.77	21.12	100

Supplementary Table S4. Relative frequencies of complementary A-T and C-G base pairs from NBPF genes including introns in human chromosomes (Figure 3D). Genomic data are from assembly coding DNA in human chromosomes. Relative frequencies of complementary base pairs strongly differ so that CSPR (strand symmetry) is significantly violated (Figure 3D). It should be noted that in all genes the frequencies of T are larger than the frequencies of A, except in the gene 5.P and in 21.P are comparable. Such relationship does not hold for G and C.

Gene	Total bp	A%	T%	G%	C%	Total %
**1**	52,444	27.03	28.91	22.52	21.54	100
**2.P**	4,543	22.39	28.75	23.42	25.45	100
**3**	46,078	26.69	30.18	21.83	21.3	100
**4**	21,941	26.44	28.46	21.99	23.19	100
**5.P**	36,177	33.71	26.27	20.27	19.75	100
**6**	21,921	26.45	28.46	21.88	23.21	100
**7**	10,845	26.84	27.87	22.81	22.48	100
**8**	32,587	26.42	29.11	22.86	21.62	100
**9**	50,735	26.17	30.27	22.17	21.4	100
**10**	80,306	24.96	31.04	22.34	21.66	100
**11**	51,477	25.76	28.99	22.85	22.4	100
**12**	58,659	26.36	29.66	22.30	21.68	100
**13.P**	38,138	28.01	31.48	20.11	20.40	100
**14**	149,567	26.76	31.83	21.56	19.85	100
**15**	41,489	26.12	30.98	21.73	21.17	100
**17.P**	41,626	27.94	30.60	20.68	20.79	100
**19**	166,939	26.40	32.00	21.42	20.18	100
**20**	117,079	24.46	31.45	22.24	21.85	100
**21.P**	22,652	27.98	27.75	22.02	22.25	100
**22.P**	15,204	26.08	29.27	21.08	23.57	100
**25.P**	36,714	27.49	30.40	21.43	20.68	100
**26**	118,759	27.39	31.80	21.36	19.45	100

Supplementary Table S5. Relative frequencies of complementary A-T and C-G base pairs from NBPF introns in human chromosomes. Genomic data are from assembly coding DNA in human chromosomes. Relative frequencies of complementary base pairs strongly differ so that CSPR (strand symmetry) is significantly violated (see Figure 3c). It

should be noted that in all genes the frequencies of T are larger than the frequencies of A, except in the gene 5.P, and in 21.P are comparable. Such relationship does not hold for G and C.

Gene	Total bp	Intron total bp	%	A%	T%	G%	C%	Total %
**1**	52,444	45,237	86.26	27.03	29.59	22.12	21.96	100
**2P**	4,543	2,729	60.07	22.90	32.54	20.85	22.10	98.39
**3**	46,078	41,033	89.05	26.85	30.56	21.49	21.10	100
**4**	21,941	18,242	83.14	25.92	29.62	21.48	22.98	100
**5.P**	36,177	33,371	92.24	33.96	26.32	20.11	19.61	100
**6**	21,921	18,135	82.73	25.88	29.64	21.50	22.98	100
**7**	10,845	8,319	76.71	25.78	29.34	22.65	22.23	100
**8**	32,587	26,057	79.96	25.77	30.09	22.59	21.55	100
**9**	50,735	43,678	86.09	26.03	31.08	21.66	21.23	100
**10**	8,1306	67,065	82.48	24.03	32.64	21.51	21.82	100
**11**	51,477	44,678	86.72	25.52	29.52	22.50	22.47	100
**12**	58,659	50,398	85.92	26.12	30.54	21.82	21.52	100
**13P**	38,138	35,155	92.18	27.96	32.15	19.74	20.15	100
**14**	149,567	53,849	36.00	24.18	32.45	21.60	21.77	100
**15**	41,489	30,717	74.04	26.41	31.74	20.97	20.89	100
**17.P**	41,626	38,307	92.03	28.04	31.07	20.41	20.48	100
**19**	166,939	151,314	90.64	26.22	32.91	20.84	20.03	100
**20**	117,079	96,908	82.77	23.41	33.31	21.24	22.05	100
**21.P**	22,652	20,001	88.30	28.16	28.04	21.74	22.06	100
**22.P**	15,204	12,425	81.72	25.42	30.41	22.80	23.37	100
**25.P**	36,714	31,687	86.31	27.58	31.18	20.84	20.41	100
**26**	118,759	112,069	94.37	27.41	32.36	21.04	19.19	100

Supplementary Table S6. Relative frequencies of complementary A-T and C-G base pairs from exons in NBPF genes. Genomic data are from assembly coding DNA in human chromosome 1. Relative frequencies of complementary base pairs strongly differ so that CSPR (strand symmetry) is significantly violated (Figure 3E). This holds even for exons longer than 6,000 bp. In all exons the frequencies of A are larger than the frequencies of T. Such relationship does not hold for G and C.

Gene	Total bp	A%	T%	G%	C%	Total %
1	5,932	28.39	23.35	24.95	23.31	100
2P	661	27.23	24.96	24.05	23.75	100
3	3,704	26.54	25.22	24.92	23.33	100
4	2,485	27.65	20.68	25.19	26.48	100
5	1,606	28.46	23.35	23.66	24.53	100
6	2,222	27.14	20.75	25.38	26.73	100
7	1,326	31.30	19.61	24.96	24.13	100
8	5,330	29.57	23.41	22.55	24.47	100
9	5,835	28.31	23.65	25.64	22.40	100
10	13,042	29.78	22.48	26.72	21.02	100
11	5,494	27.54	23.15	26.14	23.17	100
12	7,061	29.02	23.14	25.45	22.39	100
13	1,783	29.44	20.64	26.25	23.67	100
14	10,779	29.58	22.81	26.57	21.04	100
15	4,707	28.23	25.37	24.28	22.12	100
17P	2,119	28.88	22.23	25.39	23.50	100
19	14,425	28.76	22.80	27.11	21.32	100
20	18,760	29.61	22.04	27.34	21.01	100
21P	1,451	28.12	21.50	27.57	22.81	100
22P	1,580	26.58	23.48	23.04	26.90	100
25P	3,827	28.80	25.06	24.48	21.66	100
26	4,602	28.01	20.58	27.94	23.47	100

Supplementary Table S7a. Relative frequencies of trinucleotides in all 20 quadruplets in the whole human chromosome 1. *f*1, frequencies in QboxD-RC for each quadruplet; *f*2, in QboxC-R for each quadruplet; *f*u = *f*1+*f*2, sum of frequencies in both strands has the same or very similar values within the same quadruplet, in accordance with CSPR (strand symmetry).

**A-T rich quadruplets**		***f*1**	***f*2**	***f*u**	**C-G rich quadruplets**		***f*1**	***f*2**	***F*u**
**ATG**	ATG(D)	1.78	1.10	2.89	CGT	CGT(D)	0.26	1.46	1.72
	CAT(RC(D))	1.79	1.11	2.90		ACG(RC(D))	0.25	1.46	1.71
	TAC(C(D))	1.10	1.78	2.89		GCA(C(D))	1.46	0.26	1.72
	GTA(R(D))	1.11	1.79	2.90		TGC(R(D))	1.46	0.25	1.71
**TGA**	TGA(D)	1.95	1.62	3.57	GTC	GTC(D)	0.96	2.11	3.06
	TCA(RC(D))	1.96	1.61	3.58		GAC(RC(D))	0.96	2.09	3.05
	ACT(C(D))	1.62	1.95	3.57		CAG(C(D))	2.11	0.96	3.06
	AGT(R(D))	1.61	1.96	3.58		CTG(R(D))	2.09	0.96	3.05
**TAG**	TAG(D)	1.28	1.32	2.60	GCT	GCT(D)	1.44	0.23	1.66
	CTA(RC(D))	1.28	1.33	2.60		AGC(RC(D))	1.44	0.23	1.67
	ATC(C(D))	1.32	1.28	2.60		CGA(C(D))	0.23	1.44	1.66
	GAT(R(D))	1.33	1.28	2.60		TCG(R(D))	0.23	1.44	1.67
**TAA**	TAA(D)	1.99	2.39	4.37	GCC	GCC(D)	1.25	0.29	1.55
	TTA(RC(D))	1.98	2.37	4.36		GGC(RC(D))	1.25	0.29	1.55
	ATT(C(D))	2.39	1.99	4.37		CGG(C(D))	0.29	1.25	1.55
	AAT(R(D))	2.37	1.98	4.36		CCG(R(D))	0.29	1.25	1.55
**AAC**	AAC(D)	1.45	1.89	3.33	CCA	CCA(D)	1.88	1.18	3.06
	GTT(RC(D))	1.44	1.86	3.31		TGG(RC(D))	1.90	1.18	3.08
	TTG(C(D))	1.89	1.45	3.33		GGT(C(D))	1.18	1.88	3.06
	CAA(R(D))	1.86	1.44	3.31		ACC(R(D))	1.18	1.90	3.08
**AAG**	AAG(D)	1.99	1.97	3.96	CCT	CCT(D)	1.85	1.60	3.45
	CTT(RC(D))	2.01	1.96	3.97		AGG(RC(D))	1.85	1.59	3.44
	TTC(C(D))	1.97	1.99	3.96		GGA(C(D))	1.60	1.85	3.45
	GAA(R(D))	1.96	2.01	3.97		TCC(R(D))	1.59	1.85	3.44
**ACA**	ACA(D)	0.99	0.99	1.98	CAC	CAC(D)	0.76	0.77	1.53
	TGT(RC(D))	0.99	0.99	1.98		GTG(RC(D))	0.77	0.76	1.53
	TGT(C(D))	0.99	0.99	1.98		GTG(C(D))	0.77	0.76	1.53
	ACA(R(D))	0.99	0.99	1.98		CAC(R(D))	0.76	0.77	1.53
**ATA**	ATA(D)	0.97	0.97	1.94	CGC	CGC(D)	0.13	0.13	0.25
	TAT(RC(D))	0.97	0.97	1.94		GCG(RC(D))	0.13	0.13	0.25
	TAT(C(D))	0.97	0.97	1.94		GCG(C(D))	0.13	0.13	0.25
	ATA(R(D))	0.97	0.97	1.94		CGC(R(D))	0.13	0.13	0.25
**AGA**	AGA(D)	1.12	1.11	2.23	CTC	CTC(D)	0.88	0.88	1.76
	TCT(RC(D))	1.11	1.12	2.23		GAG(RC(D))	0.88	0.88	1.76
	TCT(C(D))	1.11	1.12	2.23		GAG(C(D))	0.88	0.88	1.76
	AGA(R(D))	1.12	1.11	2.23		CTC(R(D))	0.88	0.88	1.76
**AAA**	AAA(D)	1.85	1.86	3.71	CCC	CCC(D)	0.69	0.69	1.38
	TTT(RC(D))	1.86	1.85	3.71		GGG(RC(D))	0.69	0.69	1.38
	TTT(C(D))	1.86	1.85	3.71		GGG(C(D))	0.69	0.69	1.38
	AAA(R(D))	1.85	1.86	3.71		CCC(R(D))	0.69	0.69	1.38

Supplementary Table S7b. Relative frequencies of trinucleotides of all 20 quadruplets for a sequence of 200,000 bp formed of randomly selected subsequence from human chromosome 1. For description see caption to Table 4a. Differences between frequencies within each quadruplet are still small, revealing an effect of CSPR (strand symmetry).

**A-T rich quadruplet**		***f*1**	***f*2**	***f*u**	**C-G rich quadruplet**		***f*1**	***f*2**	***F*u**
**ATG**	ATG(D)	1.66	1.06	2.73	CGT	CGT(D)	0.29	1.56	1.85
	CAT(RC(D))	1.70	1.03	2.73		ACG(RC(D))	0.29	1.56	1.86
	TAC(C(D))	1.06	1.66	2.73		GCA(C(D))	1.56	0.29	1.85
	GTA(R(D))	1.03	1.70	2.73		TGC(R(D))	1.56	0.29	1.86
**TGA**	TGA(D)	1.91	1.56	3.47	GTC	GTC(D)	1.08	2.38	3.45
	TCA(RC(D))	1.96	1.58	3.54		GAC(RC(D))	1.00	2.33	3.32
	ACT(C(D))	1.56	1.91	3.47		CAG(C(D))	2.38	1.08	3.45
	AGT(R(D))	1.58	1.96	3.54		CTG(R(D))	2.33	1.00	3.32
**TAG**	TAG(D)	1.18	1.31	2.49	GCT	GCT(D)	1.59	0.27	1.85
	CTA(RC(D))	1.24	1.25	2.49		AGC(RC(D))	1.63	0.25	1.89
	ATC(C(D))	1.31	1.18	2.49		CGA(C(D))	0.27	1.59	1.85
	GAT(R(D))	1.25	1.24	2.49		TCG(R(D))	0.25	1.63	1.89
**TAA**	TAA(D)	1.65	2.09	3.74	GCC	GCC(D)	1.56	0.40	1.96
	TTA(RC(D))	1.69	2.04	3.73		GGC(RC(D))	1.53	0.39	1.91
	ATT(C(D))	2.09	1.65	3.74		CGG(C(D))	0.40	1.56	1.96
	AAT(R(D))	2.04	1.69	3.73		CCG(R(D))	0.39	1.53	1.91
**AAC**	AAC(D)	1.32	1.78	3.10	CCA	CCA(D)	2.12	1.33	3.45
	GTT(RC(D))	1.31	1.80	3.10		TGG(RC(D))	2.08	1.30	3.38
	TTG(C(D))	1.78	1.32	3.10		GGT(C(D))	1.33	2.12	3.45
	CAA(R(D))	1.80	1.31	3.10		ACC(R(D))	1.30	2.08	3.38
**AAG**	AAG(D)	1.88	1.78	3.66	CCT	CCT(D)	2.08	1.66	3.75
	CTT(RC(D))	1.87	1.79	3.65		AGG(RC(D))	2.04	1.73	3.77
	TTC(C(D))	1.78	1.88	3.66		GGA(C(D))	1.66	2.08	3.75
	GAA(R(D))	1.79	1.87	3.65		TCC(R(D))	1.73	2.04	3.77
**ACA**	ACA(D)	0.95	0.93	1.87	CAC	CAC(D)	0.83	0.82	1.65
	TGT(RC(D))	0.93	0.95	1.87		GTG(RC(D))	0.82	0.83	1.65
	TGT(C(D))	0.93	0.95	1.87		GTG(C(D))	0.82	0.83	1.65
	ACA(R(D))	0.95	0.93	1.87		CAC(R(D))	0.83	0.82	1.65
**ATA**	ATA(D)	0.78	0.82	1.59	CGC	CGC(D)	0.17	0.17	0.34
	TAT(RC(D))	0.82	0.78	1.59		GCG(RC(D))	0.17	0.17	0.34
	TAT(C(D))	0.82	0.78	1.59		GCG(C(D))	0.17	0.17	0.34
	ATA(R(D))	0.78	0.78	1.56		CGC(R(D))	0.17	0.17	0.34
**AGA**	AGA(D)	1.06	1.08	2.14	CTC	CTC(D)	0.97	0.96	1.94
	TCT(RC(D))	1.08	1.06	2.14		GAG(RC(D))	0.96	0.97	1.94
	TCT(C(D))	1.08	1.06	2.14		GAG(C(D))	0.96	0.97	1.94
	AGA(R(D))	1.06	1.08	2.14		CTC(R(D))	0.97	0.96	1.94
**AAA**	AAA(D)	1.69	1.62	3.31	CCC	CCC(D)	0.87	0.85	1.72
	TTT(RC(D))	1.62	1.69	3.31		GGG(RC(D))	0.85	0.87	1.72
	TTT(C(D))	1.62	1.69	3.31		GGG(C(D))	0.85	0.87	1.72
	AAA(R(D))	1.69	1.62	3.31		CCC(R(D))	0.87	0.85	1.72

Supplementary Table S7c. Relative frequencies of trinucleotides of all 20 quadruplets for coding DNA from human chromosome 1. For description see caption to Table 4a. Differences between frequencies within each quadruplet is substantial (differences between D and RC and between C and R), revealing violation of CSPR (strand symmetry).

**A-T rich quadruplet**		***f*1**	***f*2**	***f*u**	**C-G rich quadruplet**		***f*1**	***f*2**	***F*u**
**ATG**	ATG(D)	1.73	0.96	2.69	CGT	CGT(D)	0.45	1.74	2.19
	CAT(RC(D))	1.53	0.82	2.35		ACG(RC(D))	0.49	1.79	2.28
	TAC(C(D))	0.96	1.73	2.69		GCA(C(D))	1.74	0.45	2.19
	GTA(R(D))	0.82	1.53	2.35		TGC(R(D))	1.79	0.49	2.28
**TGA**	TGA(D)	1.94	1.44	3.38	GTC	GTC(D)	1.08	2.65	3.73
	TCA(RC(D))	1.76	1.44	3.20		GAC(RC(D))	1.32	2.68	4.00
	ACT(C(D))	1.44	1.94	3.38		CAG(C(D))	2.65	1.08	3.73
	AGT(R(D))	1.44	1.76	3.20		CTG(R(D))	2.68	1.32	4.00
**TAG**	TAG(D)	0.80	1.23	2.03	GCT	GCT(D)	1.83	0.52	2.35
	CTA(RC(D))	1.00	1.34	2.33		AGC(RC(D))	1.94	0.46	2.40
	ATC(C(D))	1.23	0.80	2.03		CGA(C(D))	0.52	1.83	2.35
	GAT(R(D))	1.34	1.00	2.33		TCG(R(D))	0.46	1.94	2.40
**TAA**	TAA(D)	1.16	1.56	2.72	GCC	GCC(D)	1.96	0.83	2.79
	TTA(RC(D))	1.21	1.55	2.76		GGC(RC(D))	1.82	0.82	2.64
	ATT(C(D))	1.56	1.16	2.72		CGG(C(D))	0.83	1.96	2.79
	AAT(R(D))	1.55	1.21	2.76		CCG(R(D))	0.82	1.82	2.64
**AAC**	AAC(D)	1.31	1.62	2.93	CCA	CCA(D)	2.23	1.17	3.40
	GTT(RC(D))	1.19	1.71	2.90		TGG(RC(D))	2.28	1.48	3.76
	TTG(C(D))	1.62	1.31	2.93		GGT(C(D))	1.17	2.23	3.40
	CAA(R(D))	1.71	1.19	2.90		ACC(R(D))	1.48	2.28	3.76
**AAG**	AAG(D)	2.17	1.70	3.87	CCT	CCT(D)	2.18	2.16	4.34
	CTT(RC(D))	1.78	2.16	3.94		AGG(RC(D))	2.05	1.78	3.83
	TTC(C(D))	1.70	2.17	3.87		GGA(C(D))	2.16	2.18	4.34
	GAA(R(D))	2.16	1.78	3.94		TCC(R(D))	1.78	2.05	3.83
**ACA**	ACA(D)	0.85	0.88	1.73	CAC	CAC(D)	0.77	0.86	1.63
	TGT(RC(D))	0.88	0.85	1.73		GTG(RC(D))	0.86	0.77	1.63
	TGT(C(D))	0.88	0.85	1.73		GTG(C(D))	0.86	0.77	1.63
	ACA(R(D))	0.85	0.88	1.73		CAC(R(D))	0.77	0.86	1.63
**ATA**	ATA(D)	0.54	0.60	1.14	CGC	CGC(D)	0.34	0.35	0.70
	TAT(RC(D))	0.60	0.54	1.14		GCG(RC(D))	0.35	0.34	0.70
	TAT(C(D))	0.60	0.54	1.14		GCG(C(D))	0.35	0.34	0.70
	ATA(R(D))	0.54	0.54	1.09		CGC(R(D))	0.34	0.35	0.70
**AGA**	AGA(D)	1.16	0.92	2.08	CTC	CTC(D)	0.91	1.07	1.98
	TCT(RC(D))	0.92	1.16	2.08		GAG(RC(D))	1.07	0.91	1.98
	TCT(C(D))	0.92	1.16	2.08		GAG(C(D))	1.07	0.91	1.98
	AGA(R(D))	1.16	0.92	2.08		CTC(R(D))	0.91	1.07	1.98
**AAA**	AAA(D)	1.26	1.17	2.43	CCC	CCC(D)	1.03	0.89	1.92
	TTT(RC(D))	1.17	1.26	2.43		GGG(RC(D))	0.89	1.03	1.92
	TTT(C(D))	1.17	1.26	2.43		GGG(C(D))	0.89	1.03	1.92
	AAA(R(D))	1.26	1.17	2.43		CCC(R(D))	1.03	0.89	1.92

Supplementary Table S8. Coding and noncoding DNA in some symbionts. From genomic data from assembly, it appears that in (bold) some bases of coding DNA are more numerous than their number in total genome, or the difference between complementary bases in noncoding DNA are unexpectedly large *(Pseudovibrio_sp.FO-BEG1)*.

Candidatus hodgkinia cicadicola

Nucleotide	Whole genome		Coding part		Noncod. p.	Deficit b.
	No. bases	%	No. bases	%	No. bases	No. bases
A	25,566	17.78	25,694	19.57		**- 128**
C	39,777	27.66	34,191	26.05	5,586	
G	44,178	30.72	42,829	32.63	1,349	
T	34,274	23.84	28,560	21.76	5,714	
Sum	143,795		131,274			

Filifactor alocis

Nucleotide	Whole genome		Coding part		Noncod. p.	Deficit b.
	No. bases	%	No. bases	%	No. bases	No. bases
A	590,529	30.58	557,830	35.07	32,699	
C	388,473	20.12	233,618	14.69	154,855	
G	295,984	15.33	345,888	21.74		**-46,904**
T	656,026	33.97	453,427	28.50	202,599	
Sum	1,931,012		1,590,763			

Pseudovibrio_sp.FO-BEG1

Nucleotide	Whole genome		Coding part		Noncoding part	
	No. bases	%	No. bases	%	No. bases	%
A	1,470,574	24.85	1,177,607	23.16	292,967	**35.22**
C	1,624,070	27.45	1,320,139	25.96	303,931	**36.54**
G	1,475,755	24.94	1,393,701	27.41	82,054	**9.87**
T	1,346,359	22.75	1,193,648	23.47	152,711	**18.36**
N	122	0.00	34	0.00	88	0.01
Sum	5,916,833		5,126,302		831,751	

Candidatus carsonela ruddi

Nucleotide	Whole genome		Coding part		Noncod. p.	Deficit b.
	No. bases	%	No.bases	%	No. bases	No. bases
A	70,377	43.29	70,663	46.00		**-286**
C	11,653	7.17	8,042	5.24	3,611	
G	11,070	6.81	12,313	8.015		**-1,243**
T	69,489	42.74	62,597	40.75	6,892	
Sum	162,589		153,615			

Supplementary Table S9. A-T rich and C-G rich quadruplet matrices from each of human chromosome, 200,000-bp random sequence and coding DNA of each human chromosome. (Uploaded in separated file Supplementary Table 9. pdf.)

Supplementary Table S10. A-T rich and C-G rich quadruplet matrices from each NBPF gene, introns and exons from NBPF genes. (Uploaded in separated file Supplementary Table 10. pdf.)
