# Supplementary material for: An Explanation of Exceptions from Chargaff’s Second Parity Rule/Strand Symmetry of DNA Molecules
Source: Genes (Basel). 2022 Oct 23;13(11):1929. doi: 10.3390/genes13111929 (PMC9689577; doi:10.3390/genes13111929)

NBPF1 sequence

A-T rich												
**D**	**f**	**%**	**RC**	**f**	**%**	**C**	**f**	**%**	**R**	**f**	**%**	
**ATG**	1047,00	2,00	CAT	995,00	1,90	TAC	476,00	0,91	GTA	538,00	1,03	Ia
**TGA**	1152,00	2,20	TCA	1104,00	2,11	ACT	774,00	1,48	AGT	906,00	1,73	
**TAG**	600,00	1,14	CTA	556,00	1,06	ATC	724,00	1,38	GAT	699,00	1,33	
**TAA**	794,00	1,51	TTA	791,00	1,51	ATT	1011,00	1,93	AAT	1073,00	2,05	Ib
**AAC**	710,00	1,35	GTT	829,00	1,58	TTG	1032,00	1,97	CAA	874,00	1,67	
**AAG**	995,00	1,90	CTT	1099,00	2,10	TTC	1116,00	2,13	GAA	1110,00	2,12	
**ACA**	980,00	1,87	TGT	1307,00	2,49	TGT*			ACA*			Ic
**ATA**	763,00	1,45	TAT	778,00	1,48	TAT*			ATA*			
**AGA**	1136,00	2,17	TCT	1386,00	2,64	TCT*			AGA*			
**AAA**	1546,00	2,95	TTT	1703,00	3,25	TTT*			AAA*			

Quadruplet		f1	f2	fu
ATG	ATG(D)	2,00	0,91	2,90
	CAT(RC(D))	1,90	1,03	2,92
	TAC(C(D))	0,91	2,00	2,90
	GTA(R(D))	1,03	1,90	2,92
TGA	TGA(D)	2,20	1,48	3,67
	TCA(RC(D))	2,11	1,73	3,83
	ACT(C(D))	1,48	2,20	3,67
	AGT(R(D))	1,73	2,11	3,83
TAG	TAG(D)	1,14	1,38	2,52
	CTA(RC(D))	1,06	1,33	2,39
	ATC(C(D))	1,38	1,14	2,52
	GAT(R(D))	1,33	1,06	2,39
TAA	TAA(D)	1,51	1,93	3,44
	TTA(RC(D))	1,51	2,05	3,55
	ATT(C(D))	1,93	1,51	3,44
	AAT(R(D))	2,05	1,51	3,55
AAC	AAC(D)	1,35	1,97	3,32
	GTT(RC(D))	1,58	1,67	3,25
	TTG(C(D))	1,97	1,35	3,32
	CAA(R(D))	1,67	1,58	3,25
AAG	AAG(D)	1,90	2,13	4,03
	CTT(RC(D))	2,10	2,12	4,21
	TTC(C(D))	2,13	1,90	4,03
	GAA(R(D))	2,12	2,10	4,21
ACA	ACA(D)	0,93	1,25	2,18
	TGT(RC(D))	1,25	0,93	2,18
	TGT(C(D))	1,25	0,93	2,18
	ACA(R(D))	0,93	1,25	2,18
ATA	ATA(D)	0,73	0,74	1,47
	TAT(RC(D))	0,74	0,73	1,47
	TAT(C(D))	0,74	0,73	1,47
	ATA(R(D))	0,73	0,73	1,45
AGA	AGA(D)	1,08	1,32	2,40
	TCT(RC(D))	1,32	1,08	2,40
	TCT(C(D))	1,32	1,08	2,40
	AGA(R(D))	1,08	1,32	2,40
AAA	AAA(D)	1,47	1,62	3,10
	TTT(RC(D))	1,62	1,47	3,10
	TTT(C(D))	1,62	1,47	3,10
	AAA(R(D))	1,47	1,62	3,10

Composition of DNA sequence in NBPF1

Base	No.of bases	%
A	14178	27,03%
C	11296	21,54%
G	11810	22,52%
T	15160	28,91%
N	0	0,00%
Sum	52444	100,00%

% of coding bases from complete NBPF1 (including N bases)

86,26%

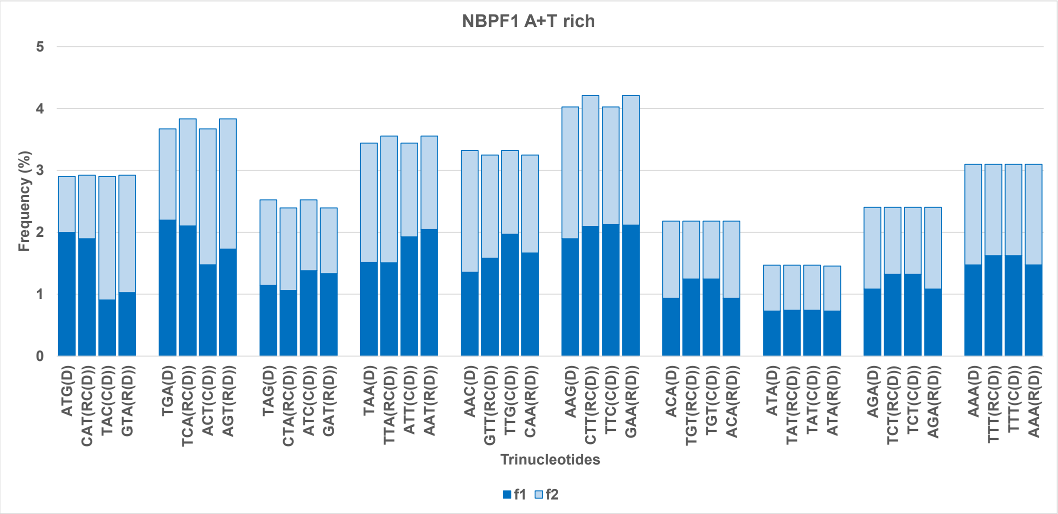

NBPF1 exons

A-T rich

D	f	%	RC	f	%	C	f	%	R	f	%	
**ATG**	113,00	1,91	CAT	105,00	1,77	TAC	40,00	0,67	GTA	37,00	0,62	Ia
**TGA**	135,00	2,28	TCA	167,00	2,82	ACT	89,00	1,50	AGT	98,00	1,65	
**TAG**	47,00	0,79	CTA	33,00	0,56	ATC	81,00	1,37	GAT	73,00	1,23	
**TAA**	43,00	0,73	TTA	67,00	1,13	ATT	83,00	1,40	AAT	92,00	1,55	Ib
**AAC**	98,00	1,65	GTT	87,00	1,47	TTG	102,00	1,72	CAA	123,00	2,07	
**AAG**	162,00	2,73	CTT	88,00	1,48	TTC	89,00	1,50	GAA	186,00	3,14	
**ACA**	116,00	1,96	TGT	104,00	1,75	TGT*			ACA*			Ic
**ATA**	48,00	0,81	TAT	55,00	0,93	TAT*			ATA*			
**AGA**	179,00	3,02	TCT	95,00	1,60	TCT*			AGA*			
**AAA**	147,00	2,48	TTT	110,00	1,85	TTT*			AAA*			

Quadruplet		f1	f2	fu
ATG	ATG(D)	1,91	0,67	2,58
	CAT(RC(D))	1,77	0,62	2,39
	TAC(C(D))	0,67	1,91	2,58
	GTA(R(D))	0,62	1,77	2,39
TGA	TGA(D)	2,28	1,50	3,78
	TCA(RC(D))	2,82	1,65	4,47
	ACT(C(D))	1,50	2,28	3,78
	AGT(R(D))	1,65	2,82	4,47
TAG	TAG(D)	0,79	1,37	2,16
	CTA(RC(D))	0,56	1,23	1,79
	ATC(C(D))	1,37	0,79	2,16
	GAT(R(D))	1,23	0,56	1,79
TAA	TAA(D)	0,73	1,40	2,12
	TTA(RC(D))	1,13	1,55	2,68
	ATT(C(D))	1,40	0,73	2,12
	AAT(R(D))	1,55	1,13	2,68
AAC	AAC(D)	1,65	1,72	3,37
	GTT(RC(D))	1,47	2,07	3,54
	TTG(C(D))	1,72	1,65	3,37
	CAA(R(D))	2,07	1,47	3,54
AAG	AAG(D)	2,73	1,50	4,23
	CTT(RC(D))	1,48	3,14	4,62
	TTC(C(D))	1,50	2,73	4,23
	GAA(R(D))	3,14	1,48	4,62
ACA	ACA(D)	0,98	0,88	1,85
	TGT(RC(D))	0,88	0,98	1,85
	TGT(C(D))	0,88	0,98	1,85
	ACA(R(D))	0,98	0,88	1,85
ATA	ATA(D)	0,40	0,46	0,87
	TAT(RC(D))	0,46	0,40	0,87
	TAT(C(D))	0,46	0,40	0,87
	ATA(R(D))	0,40	0,40	0,81
AGA	AGA(D)	1,51	0,80	2,31
	TCT(RC(D))	0,80	1,51	2,31
	TCT(C(D))	0,80	1,51	2,31
	AGA(R(D))	1,51	0,80	2,31
AAA	AAA(D)	1,24	0,93	2,17
	TTT(RC(D))	0,93	1,24	2,17
	TTT(C(D))	0,93	1,24	2,17
	AAA(R(D))	1,24	0,93	2,17

Composition of DNA sequence in NBPF1 exons

Base	No.of bases	%
A	1684	28,39%
C	1383	23,31%
G	1480	24,95%
T	1385	23,35%
N	0	0,00%
Sum	5932	100,00%

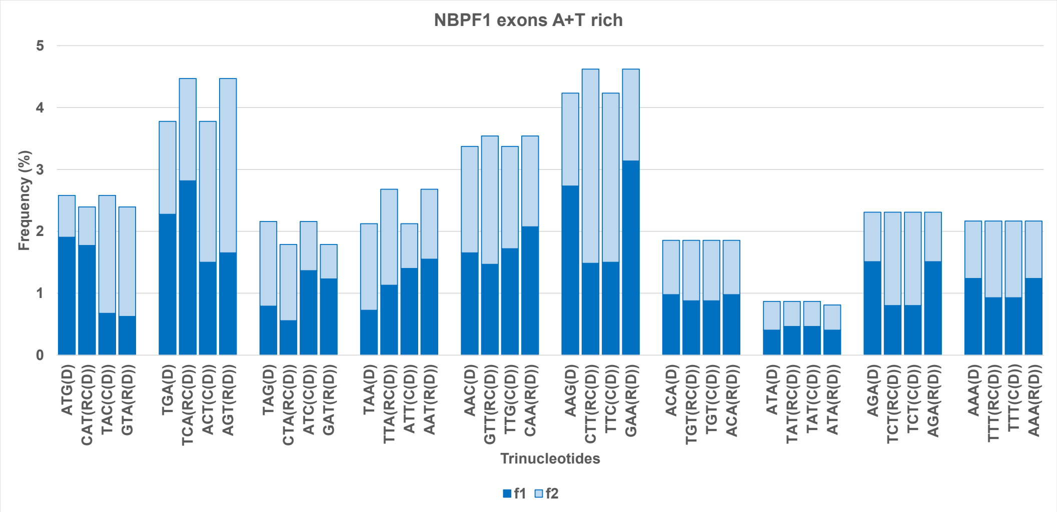

NBPF1 introns

A-T rich

D	f	%	RC	f	%	C	f	%	R	f	%	
**ATG**	113,00	1,91	CAT	105,00	1,77	TAC	40,00	0,67	GTA	37,00	0,62	Ia
**TGA**	135,00	2,28	TCA	167,00	2,82	ACT	89,00	1,50	AGT	98,00	1,65	
**TAG**	47,00	0,79	CTA	33,00	0,56	ATC	81,00	1,37	GAT	73,00	1,23	
**TAA**	43,00	0,73	TTA	67,00	1,13	ATT	83,00	1,40	AAT	92,00	1,55	Ib
**AAC**	98,00	1,65	GTT	87,00	1,47	TTG	102,00	1,72	CAA	123,00	2,07	
**AAG**	162,00	2,73	CTT	88,00	1,48	TTC	89,00	1,50	GAA	186,00	3,14	
**ACA**	116,00	1,96	TGT	104,00	1,75	TGT*			ACA*			Ic
**ATA**	48,00	0,81	TAT	55,00	0,93	TAT*			ATA*			
**AGA**	179,00	3,02	TCT	95,00	1,60	TCT*			AGA*			
**AAA**	147,00	2,48	TTT	110,00	1,85	TTT*			AAA*			

Quadruplet		f1	f2	fu
ATG	ATG(D)	1,91	0,67	2,58
	CAT(RC(D))	1,77	0,62	2,39
	TAC(C(D))	0,67	1,91	2,58
	GTA(R(D))	0,62	1,77	2,39
TGA	TGA(D)	2,28	1,50	3,78
	TCA(RC(D))	2,82	1,65	4,47
	ACT(C(D))	1,50	2,28	3,78
	AGT(R(D))	1,65	2,82	4,47
TAG	TAG(D)	0,79	1,37	2,16
	CTA(RC(D))	0,56	1,23	1,79
	ATC(C(D))	1,37	0,79	2,16
	GAT(R(D))	1,23	0,56	1,79
TAA	TAA(D)	0,73	1,40	2,12
	TTA(RC(D))	1,13	1,55	2,68
	ATT(C(D))	1,40	0,73	2,12
	AAT(R(D))	1,55	1,13	2,68
AAC	AAC(D)	1,65	1,72	3,37
	GTT(RC(D))	1,47	2,07	3,54
	TTG(C(D))	1,72	1,65	3,37
	CAA(R(D))	2,07	1,47	3,54
AAG	AAG(D)	2,73	1,50	4,23
	CTT(RC(D))	1,48	3,14	4,62
	TTC(C(D))	1,50	2,73	4,23
	GAA(R(D))	3,14	1,48	4,62
ACA	ACA(D)	0,98	0,88	1,85
	TGT(RC(D))	0,88	0,98	1,85
	TGT(C(D))	0,88	0,98	1,85
	ACA(R(D))	0,98	0,88	1,85
ATA	ATA(D)	0,40	0,46	0,87
	TAT(RC(D))	0,46	0,40	0,87
	TAT(C(D))	0,46	0,40	0,87
	ATA(R(D))	0,40	0,40	0,81
AGA	AGA(D)	1,51	0,80	2,31
	TCT(RC(D))	0,80	1,51	2,31
	TCT(C(D))	0,80	1,51	2,31
	AGA(R(D))	1,51	0,80	2,31
AAA	AAA(D)	1,24	0,93	2,17
	TTT(RC(D))	0,93	1,24	2,17
	TTT(C(D))	0,93	1,24	2,17
	AAA(R(D))	1,24	0,93	2,17

Composition of DNA sequence in NBPF1 introns

Base	No.of bases	%
A	12226	27,03%
C	9619	21,26%
G	10005	22,12%
T	13387	29,59%
N	0	0,00%
Sum	45237	100,00%

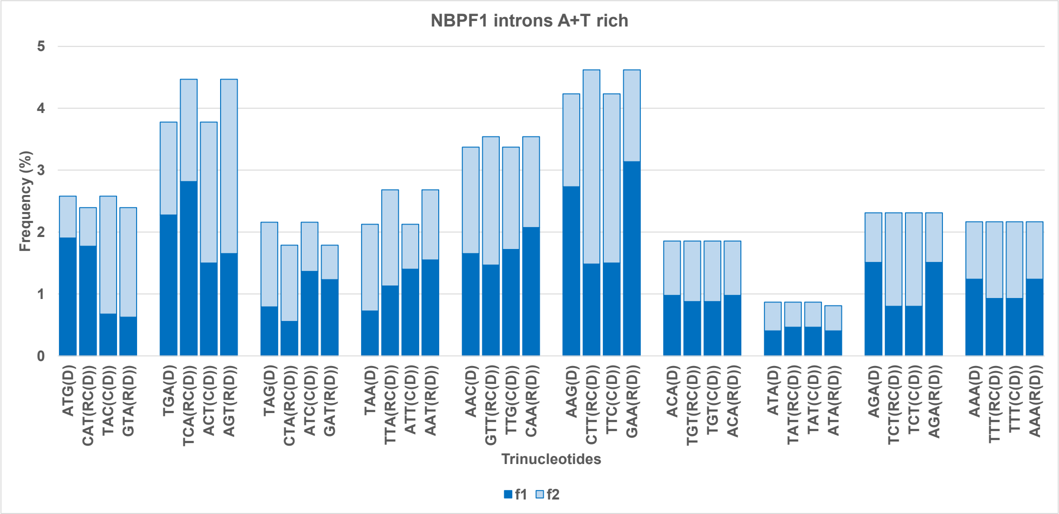

NBPF1 sequence

						C+G rich						
**D**	**f**	**%**	**RC**	**f**	**%**	**C**	**f**	**%**	**R**	**f**	**%**	
**CGT**	149,00	0,28	ACG	125,00	0,24	GCA	805,00	1,54	TGC	787,00	1,50	IIa
**GTC**	654,00	1,25	GAC	557,00	1,06	CAG	1231,00	2,35	CTG	1276,00	2,43	
**GCT**	782,00	1,49	AGC	706,00	1,35	CGA	148,00	0,28	TCG	138,00	0,26	
**GCC**	623,00	1,19	GGC	745,00	1,42	CGG	147,00	0,28	CCG	153,00	0,29	IIb
**CCA**	970,00	1,85	TGG	1082,00	2,06	GGT	632,00	1,21	ACC	623,00	1,19	
**CCT**	1036,00	1,98	AGG	1059,00	2,02	GGA	911,00	1,74	TCC	914,00	1,74	
**CAC**	759,00	1,45	GTG	973,00	1,86	GTG*			CAC*			IIc
**CGC**	137,00	0,26	GCG	165,00	0,31	GCG*			CGC*			
**CTC**	1048,00	2,00	GAG	981,00	1,87	GAG*			CTC*			
**CCC**	716,00	1,37	GGG	806,00	1,54	GGG*			CCC*			

Quadruplet		f1	f2	fu
CGT	CGT(D)	0,28	1,54	1,82
	ACG(RC(D))	0,24	1,50	1,74
	GCA(C(D))	1,54	0,28	1,82
	TGC(R(D))	1,50	0,24	1,74
GTC	GTC(D)	1,25	2,35	3,59
	GAC(RC(D))	1,06	2,43	3,50
	CAG(C(D))	2,35	1,25	3,59
	CTG(R(D))	2,43	1,06	3,50
GCT	GCT(D)	1,49	0,28	1,77
	AGC(RC(D))	1,35	0,26	1,61
	CGA(C(D))	0,28	1,49	1,77
	TCG(R(D))	0,26	1,35	1,61
GCC	GCC(D)	1,19	0,28	1,47
	GGC(RC(D))	1,42	0,29	1,71
	CGG(C(D))	0,28	1,19	1,47
	CCG(R(D))	0,29	1,42	1,71
CCA	CCA(D)	1,85	1,21	3,05
	TGG(RC(D))	2,06	1,19	3,25
	GGT(C(D))	1,21	1,85	3,05
	ACC(R(D))	1,19	2,06	3,25
CCT	CCT(D)	1,98	1,74	3,71
	AGG(RC(D))	2,02	1,74	3,76
	GGA(C(D))	1,74	1,98	3,71
	TCC(R(D))	1,74	2,02	3,76
CAC	CAC(D)	0,72	0,93	1,65
	GTG(RC(D))	0,93	0,72	1,65
	GTG(C(D))	0,93	0,72	1,65
	CAC(R(D))	0,72	0,93	1,65
CGC	CGC(D)	0,13	0,16	0,29
	GCG(RC(D))	0,16	0,13	0,29
	GCG(C(D))	0,16	0,13	0,29
	CGC(R(D))	0,13	0,16	0,29
CTC	CTC(D)	1,00	0,94	1,93
	GAG(RC(D))	0,94	1,00	1,93
	GAG(C(D))	0,94	1,00	1,93
	CTC(R(D))	1,00	0,94	1,93
CCC	CCC(D)	0,68	0,77	1,45
	GGG(RC(D))	0,77	0,68	1,45
	GGG(C(D))	0,77	0,68	1,45
	CCC(R(D))	0,68	0,77	1,45

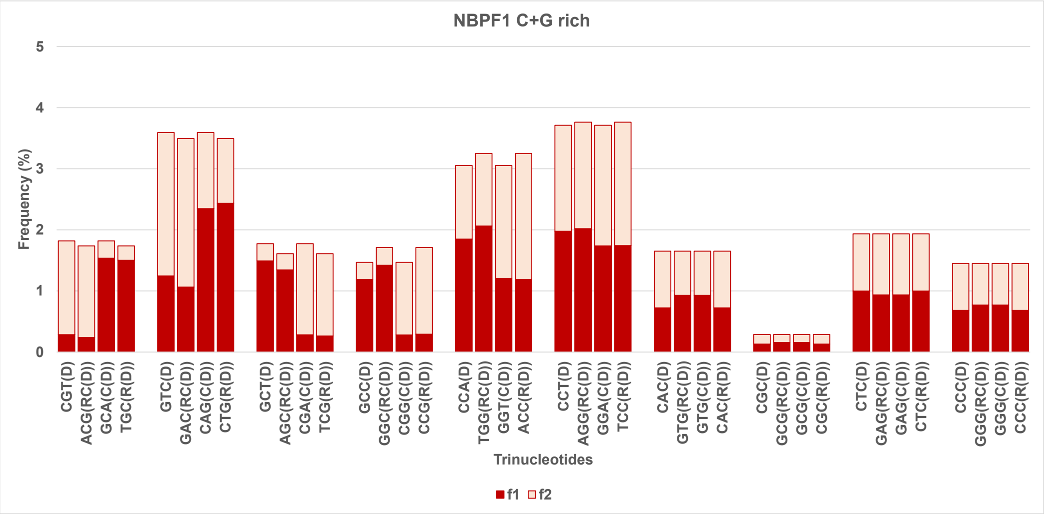

NBPF1 exons

C+G rich

D	f	%	RC	f	%	C	f	%	R	f	%	
**CGT**	15,00	0,25	ACG	33,00	0,56	GCA	97,00	1,64	TGC	92,00	1,55	IIa
**GTC**	74,00	1,25	GAC	86,00	1,45	CAG	193,00	3,25	CTG	159,00	2,68	
**GCT**	108,00	1,82	AGC	128,00	2,16	CGA	35,00	0,59	TCG	10,00	0,17	
**GCC**	98,00	1,65	GGC	92,00	1,55	CGG	29,00	0,49	CCG	27,00	0,46	IIb
**CCA**	122,00	2,06	TGG	119,00	2,01	GGT	58,00	0,98	ACC	67,00	1,13	
**CCT**	125,00	2,11	AGG	150,00	2,53	GGA	148,00	2,50	TCC	109,00	1,84	
**CAC**	81,00	1,37	GTG	77,00	1,30	GTG*			CAC*			IIc
**CGC**	18,00	0,30	GCG	27,00	0,46	GCG*			CGC*			
**CTC**	137,00	2,31	GAG	152,00	2,56	GAG*			CTC*			
**CCC**	93,00	1,57	GGG	79,00	1,33	GGG*			CCC*			

Quadruplet		f1	f2	fu
	CGT(D)	0,25	1,64	1,89
CGT	ACG(RC(D))	0,56	1,55	2,11
	GCA(C(D))	1,64	0,25	1,89
	TGC(R(D))	1,55	0,56	2,11
	GTC(D)	1,25	3,25	4,50
GTC	GAC(RC(D))	1,45	2,68	4,13
	CAG(C(D))	3,25	1,25	4,50
	CTG(R(D))	2,68	1,45	4,13
	GCT(D)	1,82	0,59	2,41
GCT	AGC(RC(D))	2,16	0,17	2,33
	CGA(C(D))	0,59	1,82	2,41
	TCG(R(D))	0,17	2,16	2,33
	GCC(D)	1,65	0,49	2,14
GCC	GGC(RC(D))	1,55	0,46	2,01
	CGG(C(D))	0,49	1,65	2,14
	CCG(R(D))	0,46	1,55	2,01
	CCA(D)	2,06	0,98	3,04
CCA	TGG(RC(D))	2,01	1,13	3,14
	GGT(C(D))	0,98	2,06	3,04
	ACC(R(D))	1,13	2,01	3,14
	CCT(D)	2,11	2,50	4,60
CCT	AGG(RC(D))	2,53	1,84	4,37
	GGA(C(D))	2,50	2,11	4,60
	TCC(R(D))	1,84	2,53	4,37
	CAC(D)	0,68	0,65	1,33
CAC	GTG(RC(D))	0,65	0,68	1,33
	GTG(C(D))	0,65	0,68	1,33
	CAC(R(D))	0,68	0,65	1,33
	CGC(D)	0,15	0,23	0,38
CGC	GCG(RC(D))	0,23	0,15	0,38
	GCG(C(D))	0,23	0,15	0,38
	CGC(R(D))	0,15	0,23	0,38
	CTC(D)	1,16	1,28	2,44
CTC	GAG(RC(D))	1,28	1,16	2,44
	GAG(C(D))	1,28	1,16	2,44
	CTC(R(D))	1,16	1,28	2,44
	CCC(D)	0,78	0,67	1,45
CCC	GGG(RC(D))	0,67	0,78	1,45
	GGG(C(D))	0,67	0,78	1,45
	CCC(R(D))	0,78	0,67	1,45

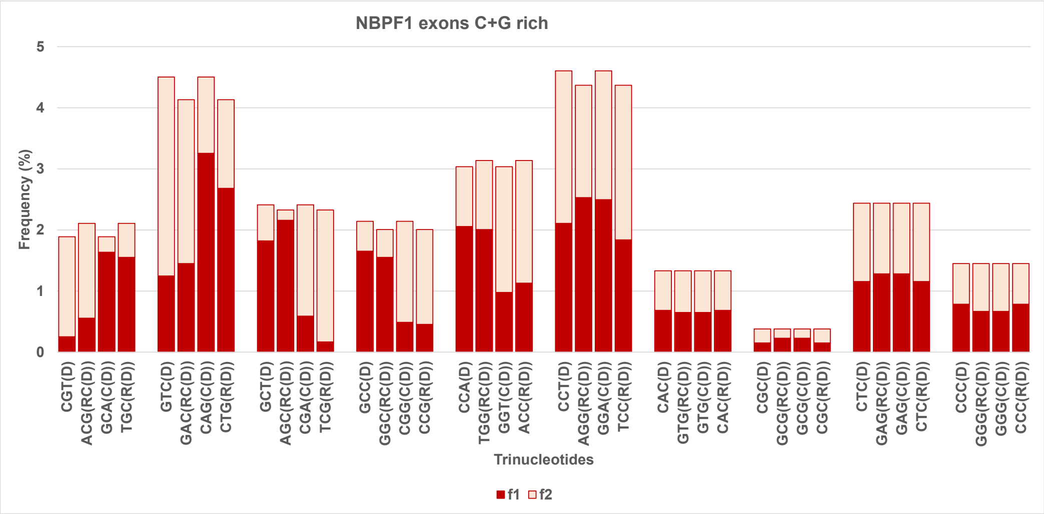

NBPF1 introns

C+G rich

D	f	%	RC	f	%	C	f	%	R	f	%	
**CGT**	15,00	0,25	ACG	33,00	0,56	GCA	97,00	1,64	TGC	92,00	1,55	IIa
**GTC**	74,00	1,25	GAC	86,00	1,45	CAG	193,00	3,25	CTG	159,00	2,68	
**GCT**	108,00	1,82	AGC	128,00	2,16	CGA	35,00	0,59	TCG	10,00	0,17	
**GCC**	98,00	1,65	GGC	92,00	1,55	CGG	29,00	0,49	CCG	27,00	0,46	IIb
**CCA**	122,00	2,06	TGG	119,00	2,01	GGT	58,00	0,98	ACC	67,00	1,13	
**CCT**	125,00	2,11	AGG	150,00	2,53	GGA	148,00	2,50	TCC	109,00	1,84	
**CAC**	81,00	1,37	GTG	77,00	1,30	GTG*			CAC*			IIc
**CGC**	18,00	0,30	GCG	27,00	0,46	GCG*			CGC*			
**CTC**	137,00	2,31	GAG	152,00	2,56	GAG*			CTC*			
**CCC**	93,00	1,57	GGG	79,00	1,33	GGG*			CCC*			

Quadruplet		f1	f2	fu
CGT	CGT(D)	0,25	1,64	1,89
	ACG(RC(D))	0,56	1,55	2,11
	GCA(C(D))	1,64	0,25	1,89
	TGC(R(D))	1,55	0,56	2,11
GTC	GTC(D)	1,25	3,25	4,50
	GAC(RC(D))	1,45	2,68	4,13
	CAG(C(D))	3,25	1,25	4,50
	CTG(R(D))	2,68	1,45	4,13
GCT	GCT(D)	1,82	0,59	2,41
	AGC(RC(D))	2,16	0,17	2,33
	CGA(C(D))	0,59	1,82	2,41
	TCG(R(D))	0,17	2,16	2,33
GCC	GCC(D)	1,65	0,49	2,14
	GGC(RC(D))	1,55	0,46	2,01
	CGG(C(D))	0,49	1,65	2,14
	CCG(R(D))	0,46	1,55	2,01
CCA	CCA(D)	2,06	0,98	3,04
	TGG(RC(D))	2,01	1,13	3,14
	GGT(C(D))	0,98	2,06	3,04
	ACC(R(D))	1,13	2,01	3,14
CCT	CCT(D)	2,11	2,50	4,60
	AGG(RC(D))	2,53	1,84	4,37
	GGA(C(D))	2,50	2,11	4,60
	TCC(R(D))	1,84	2,53	4,37
CAC	CAC(D)	0,68	0,65	1,33
	GTG(RC(D))	0,65	0,68	1,33
	GTG(C(D))	0,65	0,68	1,33
	CAC(R(D))	0,68	0,65	1,33
CGC	CGC(D)	0,15	0,23	0,38
	GCG(RC(D))	0,23	0,15	0,38
	GCG(C(D))	0,23	0,15	0,38
	CGC(R(D))	0,15	0,23	0,38
CTC	CTC(D)	1,16	1,28	2,44
	GAG(RC(D))	1,28	1,16	2,44
	GAG(C(D))	1,28	1,16	2,44
	CTC(R(D))	1,16	1,28	2,44
CCC	CCC(D)	0,78	0,67	1,45
	GGG(RC(D))	0,67	0,78	1,45
	GGG(C(D))	0,67	0,78	1,45
	CCC(R(D))	0,78	0,67	1,45

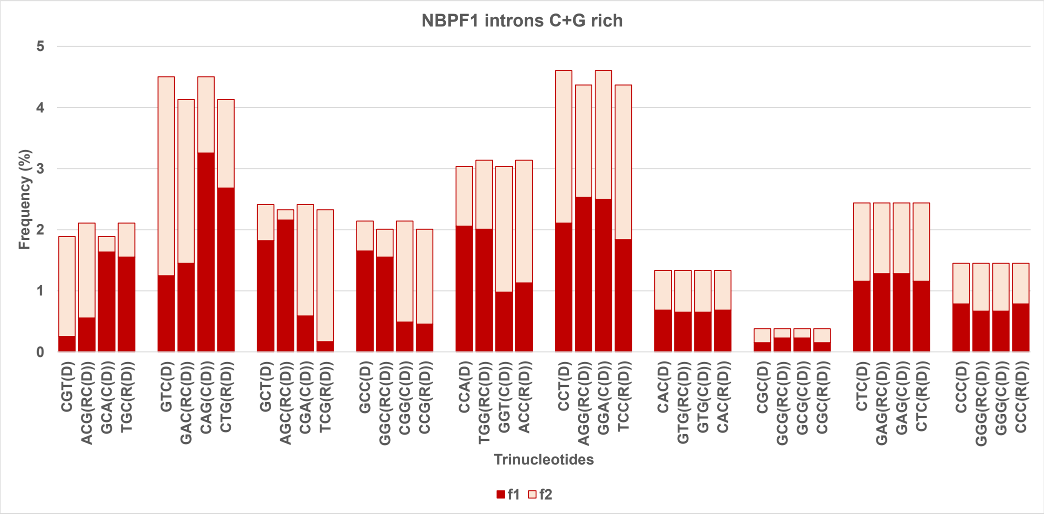

						A-T rich						
**D**	**f**	**%**	**RC**	**f**	**%**	**C**	**f**	**%**	**R**	**f**	**%**	
**ATG**	81,00	1,78	CAT	85,00	1,87	TAC	53,00	1,17	GTA	43,00	0,95	Ia
**TGA**	107,00	2,36	TCA	101,00	2,22	ACT	91,00	2,00	AGT	64,00	1,41	
**TAG**	49,00	1,08	CTA	56,00	1,23	ATC	44,00	0,97	GAT	61,00	1,34	
**TAA**	41,00	0,90	TTA	62,00	1,37	ATT	98,00	2,16	AAT	59,00	1,30	Ib
**AAC**	51,00	1,12	GTT	65,00	1,43	TTG	94,00	2,07	CAA	52,00	1,15	
**AAG**	62,00	1,37	CTT	91,00	2,00	TTC	98,00	2,16	GAA	79,00	1,74	
**ACA**	61,00	1,34	TGT	102,00	2,25	TGT*			ACA*			Ic
**ATA**	45,00	0,99	TAT	62,00	1,37	TAT*			ATA*			
**AGA**	71,00	1,56	TCT	117,00	2,58	TCT*			AGA*			
**AAA**	52,00	1,15	TTT	148,00	3,26	TTT*			AAA*			

Quadruplet		f1	f2	fu
ATG	ATG(D)	1,78	1,17	2,95
	CAT(RC(D))	1,87	0,95	2,82
	TAC(C(D))	1,17	1,78	2,95
	GTA(R(D))	0,95	1,87	2,82
TGA	TGA(D)	2,36	2,00	4,36
	TCA(RC(D))	2,22	1,41	3,63
	ACT(C(D))	2,00	2,36	4,36
	AGT(R(D))	1,41	2,22	3,63
TAG	TAG(D)	1,08	0,97	2,05
	CTA(RC(D))	1,23	1,34	2,58
	ATC(C(D))	0,97	1,08	2,05
	GAT(R(D))	1,34	1,23	2,58
TAA	TAA(D)	0,90	2,16	3,06
	TTA(RC(D))	1,37	1,30	2,66
	ATT(C(D))	2,16	0,90	3,06
	AAT(R(D))	1,30	1,37	2,66
AAC	AAC(D)	1,12	2,07	3,19
	GTT(RC(D))	1,43	1,15	2,58
	TTG(C(D))	2,07	1,12	3,19
	CAA(R(D))	1,15	1,43	2,58
AAG	AAG(D)	1,37	2,16	3,52
	CTT(RC(D))	2,00	1,74	3,74
	TTC(C(D))	2,16	1,37	3,52
	GAA(R(D))	1,74	2,00	3,74
ACA	ACA(D)	0,67	1,12	1,79
	TGT(RC(D))	1,12	0,67	1,79
	TGT(C(D))	1,12	0,67	1,79
	ACA(R(D))	0,67	1,12	1,79
ATA	ATA(D)	0,50	0,68	1,18
	TAT(RC(D))	0,68	0,50	1,18
	TAT(C(D))	0,68	0,50	1,18
	ATA(R(D))	0,50	0,50	0,99
AGA	AGA(D)	0,78	1,29	2,07
	TCT(RC(D))	1,29	0,78	2,07
	TCT(C(D))	1,29	0,78	2,07
	AGA(R(D))	0,78	1,29	2,07
AAA	AAA(D)	0,57	1,63	2,20
	TTT(RC(D))	1,63	0,57	2,20
	TTT(C(D))	1,63	0,57	2,20
	AAA(R(D))	0,57	1,63	2,20

Composition of DNA sequence in NBPF2P

Base	No.of bases	%
A	1017	22,39%
C	1156	25,45%
G	1064	23,42%
T	1306	28,75%
N	0	0,00%
Sum	4543	100,00%

% of coding bases from NBPF2P (including N bases)

60,07%

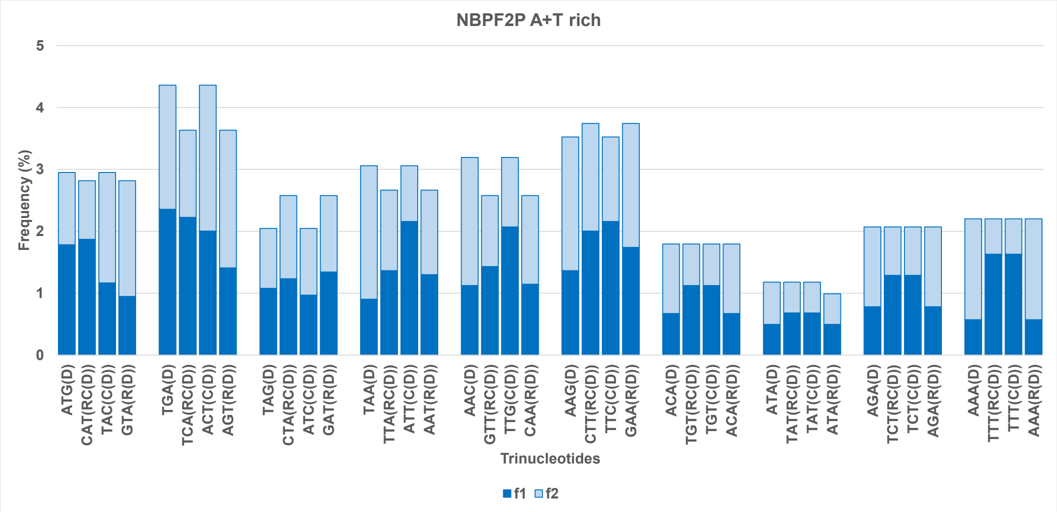

NBPF2P exons

A-T rich

D	f	%	RC	f	%	C	f	%	R	f	%	
**ATG**	9,00	1,37	CAT	9,00	1,37	TAC	13,00	1,97	GTA	6,00	0,91	Ia
**TGA**	13,00	1,97	TCA	18,00	2,73	ACT	23,00	3,49	AGT	12,00	1,82	
**TAG**	6,00	0,91	CTA	4,00	0,61	ATC	2,00	0,30	GAT	11,00	1,67	
**TAA**	2,00	0,30	TTA	10,00	1,52	ATT	12,00	1,82	AAT	3,00	0,46	Ib
**AAC**	9,00	1,37	GTT	6,00	0,91	TTG	17,00	2,58	CAA	10,00	1,52	
**AAG**	20,00	3,03	CTT	21,00	3,19	TTC	12,00	1,82	GAA	20,00	3,03	
**ACA**	9,00	1,37	TGT	2,00	0,30	TGT*			ACA*			Ic
**ATA**	6,00	0,91	TAT	6,00	0,91	TAT*			ATA*			
**AGA**	21,00	3,19	TCT	5,00	0,76	TCT*			AGA*			
**AAA**	9,00	1,37	TTT	20,00	3,03	TTT*			AAA*			

Quadruplet		f1	f2	fu
ATG	ATG(D)	1,37	1,97	3,34
	CAT(RC(D))	1,37	0,91	2,28
	TAC(C(D))	1,97	1,37	3,34
	GTA(R(D))	0,91	1,37	2,28
TGA	TGA(D)	1,97	3,49	5,46
	TCA(RC(D))	2,73	1,82	4,55
	ACT(C(D))	3,49	1,97	5,46
	AGT(R(D))	1,82	2,73	4,55
TAG	TAG(D)	0,91	0,30	1,21
	CTA(RC(D))	0,61	1,67	2,28
	ATC(C(D))	0,30	0,91	1,21
	GAT(R(D))	1,67	0,61	2,28
TAA	TAA(D)	0,30	1,82	2,12
	TTA(RC(D))	1,52	0,46	1,97
	ATT(C(D))	1,82	0,30	2,12
	AAT(R(D))	0,46	1,52	1,97
AAC	AAC(D)	1,37	2,58	3,95
	GTT(RC(D))	0,91	1,52	2,43
	TTG(C(D))	2,58	1,37	3,95
	CAA(R(D))	1,52	0,91	2,43
AAG	AAG(D)	3,03	1,82	4,86
	CTT(RC(D))	3,19	3,03	6,22
	TTC(C(D))	1,82	3,03	4,86
	GAA(R(D))	3,03	3,19	6,22
ACA	ACA(D)	0,68	0,15	0,83
	TGT(RC(D))	0,15	0,68	0,83
	TGT(C(D))	0,15	0,68	0,83
	ACA(R(D))	0,68	0,15	0,83
ATA	ATA(D)	0,46	0,46	0,91
	TAT(RC(D))	0,46	0,46	0,91
	TAT(C(D))	0,46	0,46	0,91
	ATA(R(D))	0,46	0,46	0,91
AGA	AGA(D)	1,59	0,38	1,97
	TCT(RC(D))	0,38	1,59	1,97
	TCT(C(D))	0,38	1,59	1,97
	AGA(R(D))	1,59	0,38	1,97
AAA	AAA(D)	0,68	1,52	2,20
	TTT(RC(D))	1,52	0,68	2,20
	TTT(C(D))	1,52	0,68	2,20
	AAA(R(D))	0,68	1,52	2,20

Composition of DNA sequence in NBPF2P exons

Base	No.of bases	%
A	180	27,23%
C	157	23,75%
G	159	24,05%
T	165	24,96%
N	0	0,00%
Sum	661	100,00%

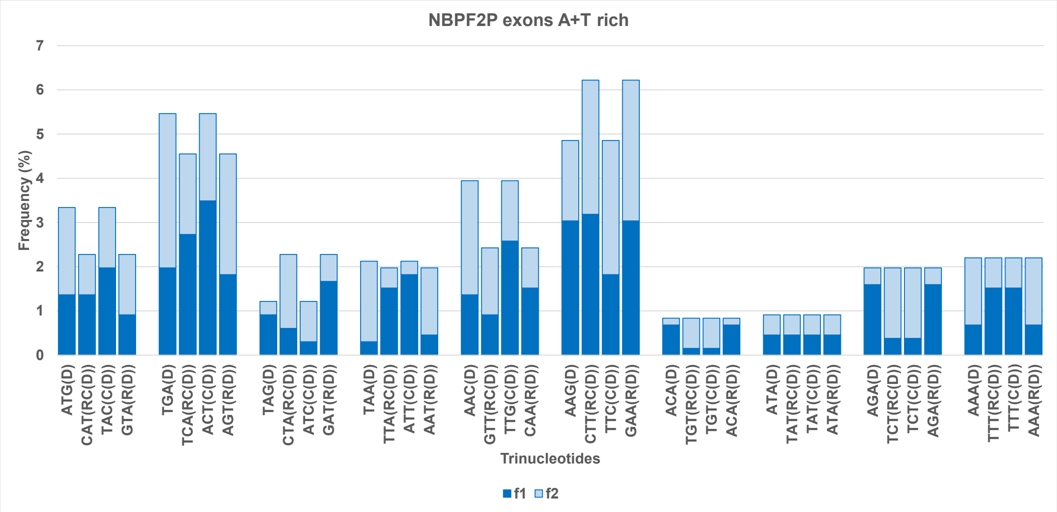

NBPF2P introns

A-T rich

D	f	%	RC	f	%	C	f	%	R	f	%	
**ATG**	57,00	2,13	CAT	54,00	2,02	TAC	31,00	1,16	GTA	24,00	0,90	Ia
**TGA**	76,00	2,84	TCA	63,00	2,35	ACT	52,00	1,94	AGT	35,00	1,31	
**TAG**	28,00	1,05	CTA	39,00	1,46	ATC	31,00	1,16	GAT	41,00	1,53	
**TAA**	33,00	1,23	TTA	43,00	1,61	ATT	73,00	2,73	AAT	52,00	1,94	Ib
**AAC**	31,00	1,16	GTT	45,00	1,68	TTG	60,00	2,24	CAA	35,00	1,31	
**AAG**	27,00	1,01	CTT	53,00	1,98	TTC	68,00	2,54	GAA	42,00	1,57	
**ACA**	37,00	1,38	TGT	80,00	2,99	TGT*			ACA*			Ic
**ATA**	32,00	1,19	TAT	46,00	1,72	TAT*			ATA*			
**AGA**	38,00	1,42	TCT	94,00	3,51	TCT*			AGA*			
**AAA**	39,00	1,46	TTT	110,00	4,11	TTT*			AAA*			

Quadruplet		f1	f2	fu
ATG	ATG(D)	2,13	1,16	3,29
	CAT(RC(D))	2,02	0,90	2,91
	TAC(C(D))	1,16	2,13	3,29
	GTA(R(D))	0,90	2,02	2,91
TGA	TGA(D)	2,84	1,94	4,78
	TCA(RC(D))	2,35	1,31	3,66
	ACT(C(D))	1,94	2,84	4,78
	AGT(R(D))	1,31	2,35	3,66
TAG	TAG(D)	1,05	1,16	2,20
	CTA(RC(D))	1,46	1,53	2,99
	ATC(C(D))	1,16	1,05	2,20
	GAT(R(D))	1,53	1,46	2,99
TAA	TAA(D)	1,23	2,73	3,96
	TTA(RC(D))	1,61	1,94	3,55
	ATT(C(D))	2,73	1,23	3,96
	AAT(R(D))	1,94	1,61	3,55
AAC	AAC(D)	1,16	2,24	3,40
	GTT(RC(D))	1,68	1,31	2,99
	TTG(C(D))	2,24	1,16	3,40
	CAA(R(D))	1,31	1,68	2,99
AAG	AAG(D)	1,01	2,54	3,55
	CTT(RC(D))	1,98	1,57	3,55
	TTC(C(D))	2,54	1,01	3,55
	GAA(R(D))	1,57	1,98	3,55
ACA	ACA(D)	0,69	1,49	2,18
	TGT(RC(D))	1,49	0,69	2,18
	TGT(C(D))	1,49	0,69	2,18
	ACA(R(D))	0,69	1,49	2,18
ATA	ATA(D)	0,60	0,86	1,46
	TAT(RC(D))	0,86	0,60	1,46
	TAT(C(D))	0,86	0,60	1,46
	ATA(R(D))	0,60	0,60	1,19
AGA	AGA(D)	0,71	1,76	2,46
	TCT(RC(D))	1,76	0,71	2,46
	TCT(C(D))	1,76	0,71	2,46
	AGA(R(D))	0,71	1,76	2,46
AAA	AAA(D)	0,73	2,05	2,78
	TTT(RC(D))	2,05	0,73	2,78
	TTT(C(D))	2,05	0,73	2,78
	AAA(R(D))	0,73	2,05	2,78

Composition of DNA sequence in NBPF2P introns

Base	No.of bases	%
A	625	22,90%
C	603	22,10%
G	569	20,85%
T	888	32,54%
N	44	1,61%
Sum	2729	100,00%

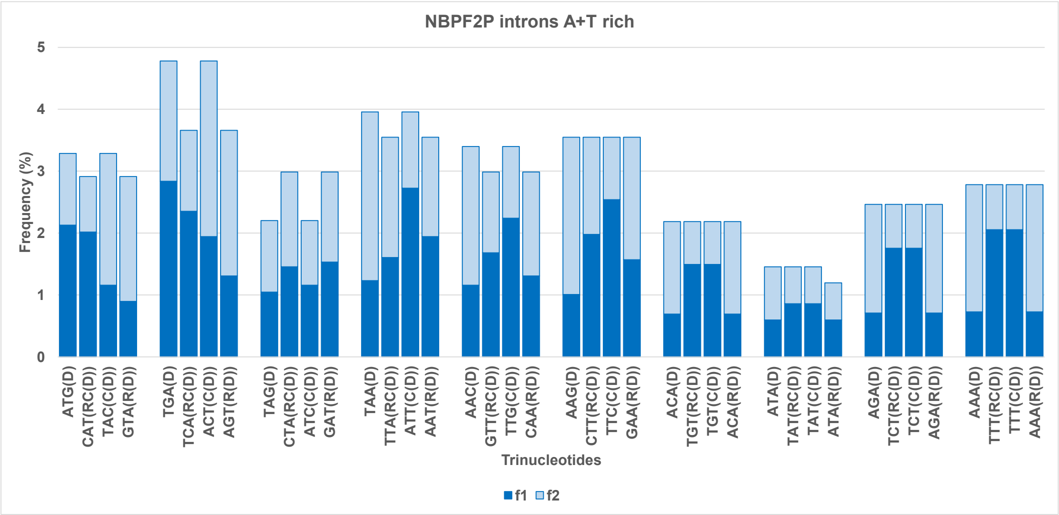

NBPF2P sequence

						C+G rich						
**D**	**f**	**%**	**RC**	**f**	**%**	**C**	**f**	**%**	**R**	**f**	**%**	
**CGT**	20,00	0,44	ACG	16,00	0,35	GCA	68,00	1,50	TGC	62,00	1,37	IIa
**GTC**	60,00	1,32	GAC	64,00	1,41	CAG	105,00	2,31	CTG	125,00	2,75	
**GCT**	80,00	1,76	AGC	63,00	1,39	CGA	19,00	0,42	TCG	18,00	0,40	
**GCC**	81,00	1,78	GGC	86,00	1,89	CGG	28,00	0,62	CCG	51,00	1,12	IIb
**CCA**	81,00	1,78	TGG	98,00	2,16	GGT	51,00	1,12	ACC	69,00	1,52	
**CCT**	111,00	2,44	AGG	89,00	1,96	GGA	78,00	1,72	TCC	93,00	2,05	
**CAC**	69,00	1,52	GTG	69,00	1,52	GTG*			CAC*			IIc
**CGC**	53,00	1,17	GCG	35,00	0,77	GCG*			CGC*			
**CTC**	127,00	2,80	GAG	71,00	1,56	GAG*			CTC*			
**CCC**	83,00	1,83	GGG	73,00	1,61	GGG*			CCC*			

Quadruplet		f1	f2	fu
CGT	CGT(D)	0,44	1,50	1,94
	ACG(RC(D))	0,35	1,37	1,72
	GCA(C(D))	1,50	0,44	1,94
	TGC(R(D))	1,37	0,35	1,72
GTC	GTC(D)	1,32	2,31	3,63
	GAC(RC(D))	1,41	2,75	4,16
	CAG(C(D))	2,31	1,32	3,63
	CTG(R(D))	2,75	1,41	4,16
GCT	GCT(D)	1,76	0,42	2,18
	AGC(RC(D))	1,39	0,40	1,78
	CGA(C(D))	0,42	1,76	2,18
	TCG(R(D))	0,40	1,39	1,78
GCC	GCC(D)	1,78	0,62	2,40
	GGC(RC(D)	1,89	1,12	3,02
	CGG(C(D))	0,62	1,78	2,40
	CCG(R(D))	1,12	1,89	3,02
CCA	CCA(D)	1,78	1,12	2,91
	TGG(RC(D))	2,16	1,52	3,68
	GGT(C(D))	1,12	1,78	2,91
	ACC(R(D))	1,52	2,16	3,68
CCT	CCT(D)	2,44	1,72	4,16
	AGG(RC(D))	1,96	2,05	4,01
	GGA(C(D))	1,72	2,44	4,16
	TCC(R(D))	2,05	1,96	4,01
CAC	CAC(D)	0,76	0,76	1,52
	GTG(RC(D))	0,76	0,76	1,52
	GTG(C(D))	0,76	0,76	1,52
	CAC(R(D))	0,76	0,76	1,52
CGC	CGC(D)	0,58	0,39	0,97
	GCG(RC(D)	0,39	0,58	0,97
	GCG(C(D))	0,39	0,58	0,97
	CGC(R(D))	0,58	0,39	0,97
CTC	CTC(D)	1,40	0,78	2,18
	GAG(RC(D))	0,78	1,40	2,18
	GAG(C(D))	0,78	1,40	2,18
	CTC(R(D))	1,40	0,78	2,18
CCC	CCC(D)	0,91	0,80	1,72
	GGG(RC(D)	0,80	0,91	1,72
	GGG(C(D))	0,80	0,91	1,72
	CCC(R(D))	0,91	0,80	1,72

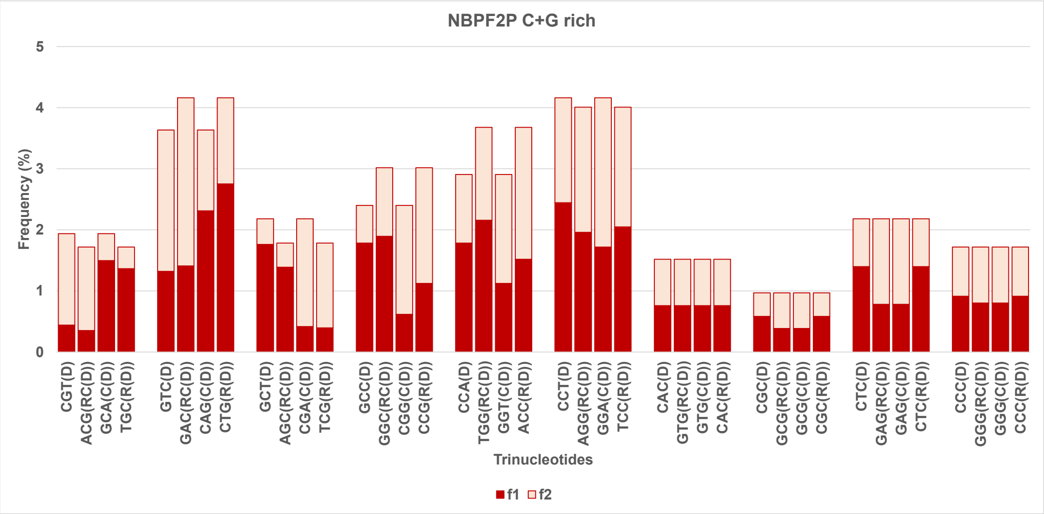

NBPF2P exons

C+G rich

D	f	%	RC	f	%	C	f	%	R	f	%	
**CGT**	4,00	0,61	ACG	5,00	0,76	GCA	9,00	1,37	TGC	15,00	2,28	IIa
**GTC**	5,00	0,76	GAC	15,00	2,28	CAG	24,00	3,64	CTG	16,00	2,43	
**GCT**	11,00	1,67	AGC	11,00	1,67	CGA	2,00	0,30	TCG	2,00	0,30	
**GCC**	16,00	2,43	GGC	10,00	1,52	CGG	1,00	0,15				IIb
**CCA**	17,00	2,58	TGG	17,00	2,58	GGT	4,00	0,61	ACC	9,00	1,37	
**CCT**	15,00	2,28	AGG	19,00	2,88	GGA	23,00	3,49	TCC	7,00	1,06	
**CAC**	10,00	1,52	GTG	5,00	0,76	GTG*			CAC*			IIc
						GCG*			CGC*			
**CTC**	13,00	1,97	GAG	13,00	1,97	GAG*			CTC*			
**CCC**	10,00	1,52	GGG	5,00	0,76	GGG*			CCC*			

Quadruplet		f1	f2	fu
CGT	CGT(D)	0,61	1,37	1,97
	ACG(RC(D))	0,76	2,28	3,03
	GCA(C(D))	1,37	0,61	1,97
	TGC(R(D))	2,28	0,76	3,03
GTC	GTC(D)	0,76	3,64	4,40
	GAC(RC(D))	2,28	2,43	4,70
	CAG(C(D))	3,64	0,76	4,40
	CTG(R(D))	2,43	2,28	4,70
GCT	GCT(D)	1,67	0,30	1,97
	AGC(RC(D))	1,67	0,30	1,97
	CGA(C(D))	0,30	1,67	1,97
	TCG(R(D))	0,30	1,67	1,97
GCC	GCC(D)	2,43	0,15	2,58
	GGC(RC(D))	1,52	0,00	1,52
	CGG(C(D))	0,15	2,43	2,58
	CCG(R(D))	0,00	1,52	1,52
CCA	CCA(D)	2,58	0,61	3,19
	TGG(RC(D))	2,58	1,37	3,95
	GGT(C(D))	0,61	2,58	3,19
	ACC(R(D))	1,37	2,58	3,95
CCT	CCT(D)	2,28	3,49	5,77
	AGG(RC(D))	2,88	1,06	3,95
	GGA(C(D))	3,49	2,28	5,77
	TCC(R(D))	1,06	2,88	3,95
CAC	CAC(D)	0,76	0,38	1,14
	GTG(RC(D))	0,38	0,76	1,14
	GTG(C(D))	0,38	0,76	1,14
	CAC(R(D))	0,76	0,38	1,14
CGC	CGC(D)	0,00	0,00	0,00
	GCG(RC(D))	0,00	0,00	0,00
	GCG(C(D))	0,00	0,00	0,00
	CGC(R(D))	0,00	0,00	0,00
CTC	CTC(D)	0,99	0,99	1,97
	GAG(RC(D))	0,99	0,99	1,97
	GAG(C(D))	0,99	0,99	1,97
	CTC(R(D))	0,99	0,99	1,97
CCC	CCC(D)	0,76	0,38	1,14
	GGG(RC(D))	0,38	0,76	1,14
	GGG(C(D))	0,38	0,76	1,14
	CCC(R(D))	0,76	0,38	1,14

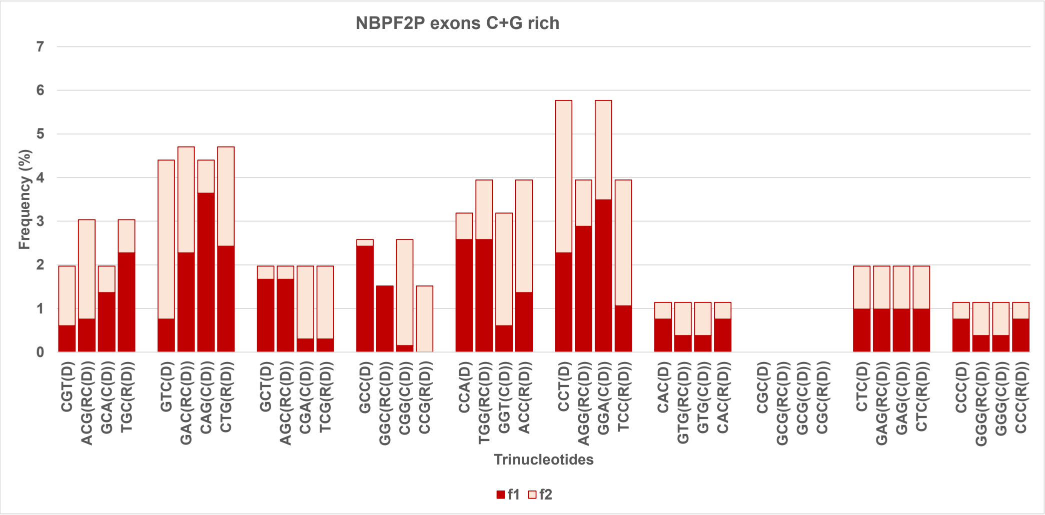

NBPF2P introns

C+G rich

D	f	%	RC	f	%	C	f	%	R	f	%	
**CGT**	8,00	0,30	ACG	4,00	0,15	GCA	41,00	1,53	TGC	29,00	1,08	IIa
**GTC**	39,00	1,46	GAC	34,00	1,27	CAG	49,00	1,83	CTG	83,00	3,10	
**GCT**	35,00	1,31	AGC	28,00	1,05	CGA	9,00	0,34	TCG	5,00	0,19	
**GCC**	20,00	0,75	GGC	38,00	1,42	CGG	1,00	0,04	CCG	10,00	0,37	IIb
**CCA**	33,00	1,23	TGG	61,00	2,28	GGT	33,00	1,23	ACC	41,00	1,53	
**CCT**	76,00	2,84	AGG	48,00	1,79	GGA	39,00	1,46	TCC	58,00	2,17	
**CAC**	37,00	1,38	GTG	48,00	1,79	GTG*			CAC*			IIc
**CGC**	5,00	0,19	GCG	4,00	0,15	GCG*			CGC*			
**CTC**	82,00	3,06	GAG	45,00	1,68	GAG*			CTC*			
**CCC**	28,00	1,05	GGG	38,00	1,42	GGG*			CCC*			

Quadruplet		f1	f2	fu
CGT	CGT(D)	0,30	1,53	1,83
	ACG(RC(D))	0,15	1,08	1,23
	GCA(C(D))	1,53	0,30	1,83
	TGC(R(D))	1,08	0,15	1,23
GTC	GTC(D)	1,46	1,83	3,29
	GAC(RC(D))	1,27	3,10	4,37
	CAG(C(D))	1,83	1,46	3,29
	CTG(R(D))	3,10	1,27	4,37
GCT	GCT(D)	1,31	0,34	1,64
	AGC(RC(D))	1,05	0,19	1,23
	CGA(C(D))	0,34	1,31	1,64
	TCG(R(D))	0,19	1,05	1,23
GCC	GCC(D)	0,75	0,04	0,78
	GGC(RC(D))	1,42	0,37	1,79
	CGG(C(D))	0,04	0,75	0,78
	CCG(R(D))	0,37	1,42	1,79
CCA	CCA(D)	1,23	1,23	2,46
	TGG(RC(D))	2,28	1,53	3,81
	GGT(C(D))	1,23	1,23	2,46
	ACC(R(D))	1,53	2,28	3,81
CCT	CCT(D)	2,84	1,46	4,29
	AGG(RC(D))	1,79	2,17	3,96
	GGA(C(D))	1,46	2,84	4,29
	TCC(R(D))	2,17	1,79	3,96
CAC	CAC(D)	0,69	0,90	1,59
	GTG(RC(D))	0,90	0,69	1,59
	GTG(C(D))	0,90	0,69	1,59
	CAC(R(D))	0,69	0,90	1,59
CGC	CGC(D)	0,09	0,07	0,17
	GCG(RC(D))	0,07	0,09	0,17
	GCG(C(D))	0,07	0,09	0,17
	CGC(R(D))	0,09	0,07	0,17
CTC	CTC(D)	1,53	0,84	2,37
	GAG(RC(D))	0,84	1,53	2,37
	GAG(C(D))	0,84	1,53	2,37
	CTC(R(D))	1,53	0,84	2,37
CCC	CCC(D)	0,52	0,71	1,23
	GGG(RC(D))	0,71	0,52	1,23
	GGG(C(D))	0,71	0,52	1,23
	CCC(R(D))	0,52	0,71	1,23

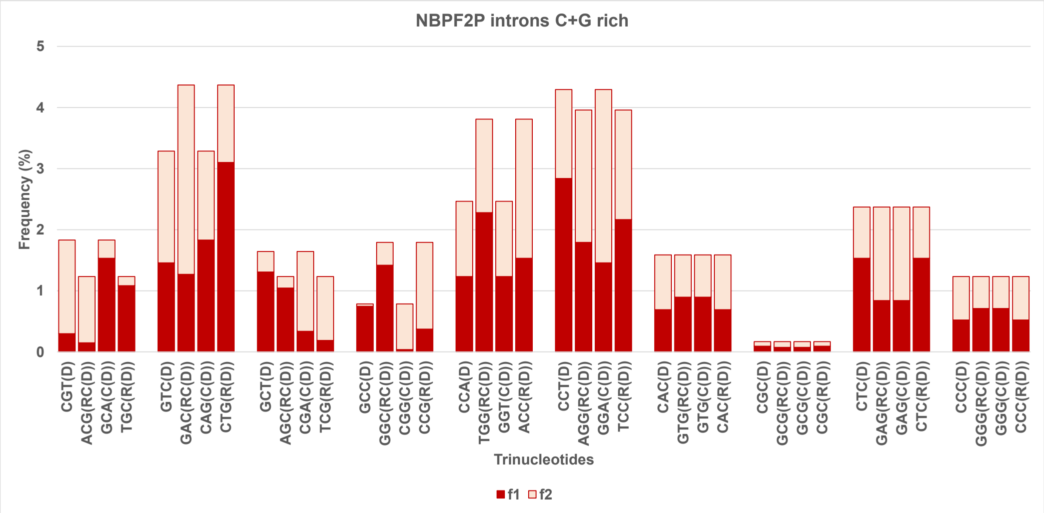

NBPF3 sequence

A-T rich												
**D**	**f**	**%**	**RC**	**f**	**%**	**C**	**f**	**%**	**R**	**f**	**%**	
**ATG**	899,00	1,95	CAT	873,00	1,89	TAC	436,00	0,95	GTA	505,00	1,10	Ia
**TGA**	1020,00	2,21	TCA	977,00	2,12	ACT	680,00	1,48	AGT	820,00	1,78	
**TAG**	580,00	1,26	CTA	489,00	1,06	ATC	606,00	1,32	GAT	619,00	1,34	
**TAA**	726,00	1,58	TTA	790,00	1,71	ATT	1014,00	2,20	AAT	984,00	2,14	Ib
**AAC**	592,00	1,28	GTT	771,00	1,67	TTG	931,00	2,02	CAA	741,00	1,61	
**AAG**	812,00	1,76	CTT	984,00	2,14	TTC	1048,00	2,27	GAA	921,00	2,00	
**ACA**	818,00	1,78	TGT	1149,00	2,49	TGT*			ACA*			Ic
**ATA**	743,00	1,61	TAT	786,00	1,71	TAT*			ATA*			
**AGA**	914,00	1,98	TCT	1177,00	2,55	TCT*			AGA*			
**AAA**	1293,00	2,81	TTT	1697,00	3,68	TTT*			AAA*			

Quadruplet		f1	f2	fu
ATG	ATG(D)	1,95	0,95	2,90
	CAT(RC(D))	1,89	1,10	2,99
	TAC(C(D))	0,95	1,95	2,90
	GTA(R(D))	1,10	1,89	2,99
TGA	TGA(D)	2,21	1,48	3,69
	TCA(RC(D))	2,12	1,78	3,90
	ACT(C(D))	1,48	2,21	3,69
	AGT(R(D))	1,78	2,12	3,90
TAG	TAG(D)	1,26	1,32	2,57
	CTA(RC(D))	1,06	1,34	2,40
	ATC(C(D))	1,32	1,26	2,57
	GAT(R(D))	1,34	1,06	2,40
TAA	TAA(D)	1,58	2,20	3,78
	TTA(RC(D))	1,71	2,14	3,85
	ATT(C(D))	2,20	1,58	3,78
	AAT(R(D))	2,14	1,71	3,85
AAC	AAC(D)	1,28	2,02	3,31
	GTT(RC(D))	1,67	1,61	3,28
	TTG(C(D))	2,02	1,28	3,31
	CAA(R(D))	1,61	1,67	3,28
AAG	AAG(D)	1,76	2,27	4,04
	CTT(RC(D))	2,14	2,00	4,13
	TTC(C(D))	2,27	1,76	4,04
	GAA(R(D))	2,00	2,14	4,13
ACA	ACA(D)	0,89	1,25	2,13
	TGT(RC(D))	1,25	0,89	2,13
	TGT(C(D))	1,25	0,89	2,13
	ACA(R(D))	0,89	1,25	2,13
ATA	ATA(D)	0,81	0,85	1,66
	TAT(RC(D))	0,85	0,81	1,66
	TAT(C(D))	0,85	0,81	1,66
	ATA(R(D))	0,81	0,81	1,61
AGA	AGA(D)	0,99	1,28	2,27
	TCT(RC(D))	1,28	0,99	2,27
	TCT(C(D))	1,28	0,99	2,27
	AGA(R(D))	0,99	1,28	2,27
AAA	AAA(D)	1,40	1,84	3,24
	TTT(RC(D))	1,84	1,40	3,24
	TTT(C(D))	1,84	1,40	3,24
	AAA(R(D))	1,40	1,84	3,24

Composition of DNA sequence in NBPF3

Base	No.of bases	%
A	12298	26,69%
C	9815	21,30%
G	10061	21,83%
T	13904	30,17%
N	0	0,00%
Sum	46078	100,00%

% of coding bases from NBPF3 (including N bases)

89,05%

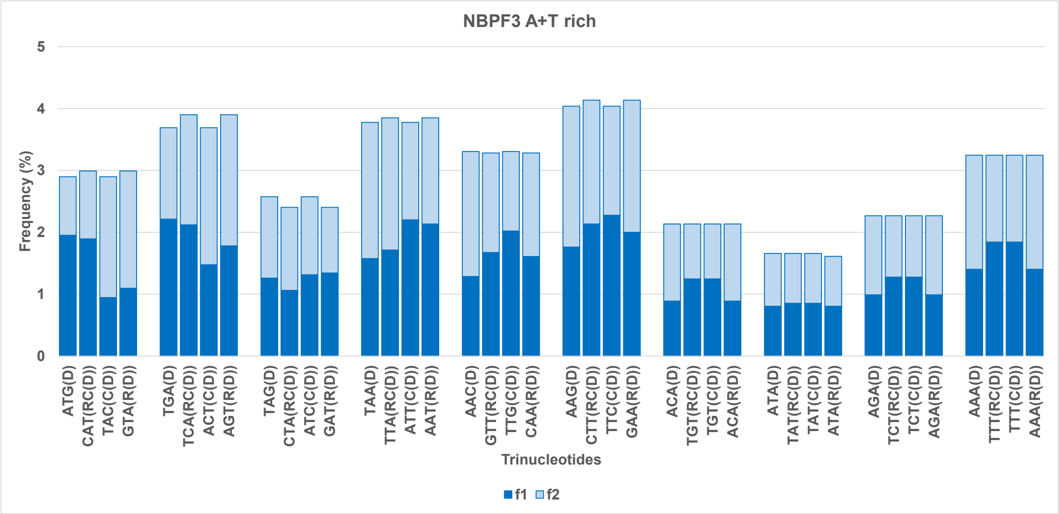

NBPF3 exons

A-T rich

D	f	%	RC	f	%	C	f	%	R	f	%	
**ATG**	66,00	1,78	CAT	64,00	1,73	TAC	32,00	0,86	GTA	33,00	0,89	Ia
**TGA**	89,00	2,40	TCA	95,00	2,57	ACT	65,00	1,76	AGT	66,00	1,78	
**TAG**	39,00	1,05	CTA	32,00	0,86	ATC	54,00	1,46	GAT	51,00	1,38	
**TAA**	26,00	0,70	TTA	47,00	1,27	ATT	56,00	1,51	AAT	46,00	1,24	Ib
**AAC**	50,00	1,35	GTT	57,00	1,54	TTG	75,00	2,03	CAA	60,00	1,62	
**AAG**	85,00	2,30	CTT	64,00	1,73	TTC	55,00	1,49	GAA	95,00	2,57	
**ACA**	63,00	1,70	TGT	70,00	1,89	TGT*			ACA*			Ic
**ATA**	31,00	0,84	TAT	46,00	1,24	TAT*			ATA*			
**AGA**	95,00	2,57	TCT	68,00	1,84	TCT*			AGA*			
**AAA**	74,00	2,00	TTT	84,00	2,27	TTT*			AAA*			

Quadruplet		f1	f2	fu
ATG	ATG(D)	1,78	0,86	2,65
	CAT(RC(D))	1,73	0,89	2,62
	TAC(C(D))	0,86	1,78	2,65
	GTA(R(D))	0,89	1,73	2,62
TGA	TGA(D)	2,40	1,76	4,16
	TCA(RC(D))	2,57	1,78	4,35
	ACT(C(D))	1,76	2,40	4,16
	AGT(R(D))	1,78	2,57	4,35
TAG	TAG(D)	1,05	1,46	2,51
	CTA(RC(D))	0,86	1,38	2,24
	ATC(C(D))	1,46	1,05	2,51
	GAT(R(D))	1,38	0,86	2,24
TAA	TAA(D)	0,70	1,51	2,22
	TTA(RC(D))	1,27	1,24	2,51
	ATT(C(D))	1,51	0,70	2,22
	AAT(R(D))	1,24	1,27	2,51
AAC	AAC(D)	1,35	2,03	3,38
	GTT(RC(D))	1,54	1,62	3,16
	TTG(C(D))	2,03	1,35	3,38
	CAA(R(D))	1,62	1,54	3,16
AAG	AAG(D)	2,30	1,49	3,78
	CTT(RC(D))	1,73	2,57	4,29
	TTC(C(D))	1,49	2,30	3,78
	GAA(R(D))	2,57	1,73	4,29
ACA	ACA(D)	0,85	0,95	1,80
	TGT(RC(D))	0,95	0,85	1,80
	TGT(C(D))	0,95	0,85	1,80
	ACA(R(D))	0,85	0,95	1,80
ATA	ATA(D)	0,42	0,62	1,04
	TAT(RC(D))	0,62	0,42	1,04
	TAT(C(D))	0,62	0,42	1,04
	ATA(R(D))	0,42	0,42	0,84
AGA	AGA(D)	1,28	0,92	2,20
	TCT(RC(D))	0,92	1,28	2,20
	TCT(C(D))	0,92	1,28	2,20
	AGA(R(D))	1,28	0,92	2,20
AAA	AAA(D)	1,00	1,13	2,13
	TTT(RC(D))	1,13	1,00	2,13
	TTT(C(D))	1,13	1,00	2,13
	AAA(R(D))	1,00	1,13	2,13

Composition of DNA sequence in NBPF3 exons

Base	No.of bases	%
A	983	26,54%
C	864	23,33%
G	923	24,92%
T	934	25,22%
N	0	0,00%
Sum	3704	100,00%

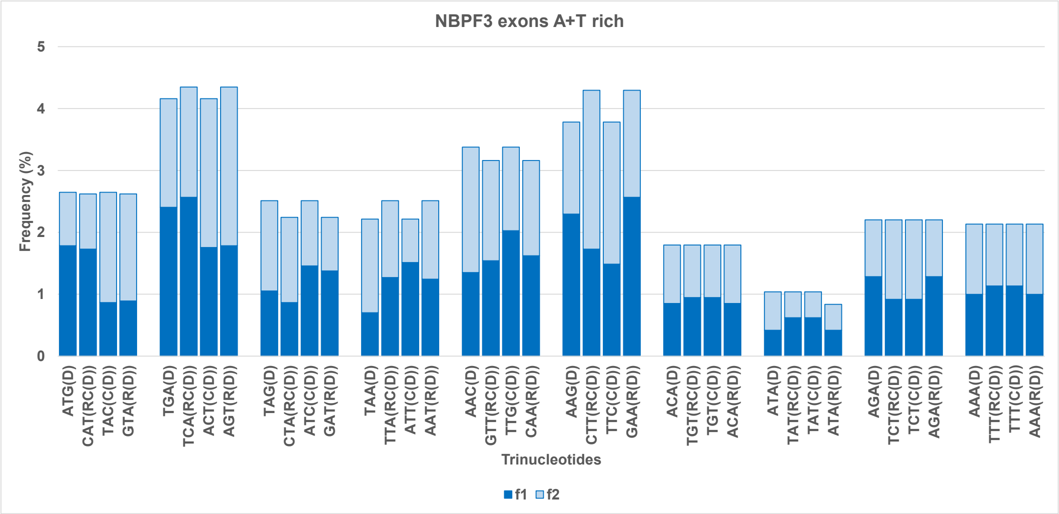

NBPF3 introns

A-T rich

D	f	%	RC	f	%	C	f	%	R	f	%	
**ATG**	811,00	1,98	CAT	780,00	1,90	TAC	386,00	0,94	GTA	461,00	1,12	Ia
**TGA**	908,00	2,21	TCA	858,00	2,09	ACT	597,00	1,46	AGT	740,00	1,80	
**TAG**	529,00	1,29	CTA	442,00	1,08	ATC	534,00	1,30	GAT	551,00	1,34	
**TAA**	677,00	1,65	TTA	718,00	1,75	ATT	927,00	2,26	AAT	912,00	2,22	Ib
**AAC**	529,00	1,29	GTT	686,00	1,67	TTG	826,00	2,01	CAA	675,00	1,65	
**AAG**	720,00	1,75	CTT	893,00	2,18	TTC	962,00	2,34	GAA	809,00	1,97	
**ACA**	732,00	1,78	TGT	1042,00	2,54	TGT*			ACA*			Ic
**ATA**	689,00	1,68	TAT	718,00	1,75	TAT*			ATA*			
**AGA**	799,00	1,95	TCT	1081,00	2,63	TCT*			AGA*			
**AAA**	1195,00	2,91	TTT	1539,00	3,75	TTT*			AAA*			

Quadruplet		f1	f2	fu
ATG	ATG(D)	1,98	0,94	2,92
	CAT(RC(D))	1,90	1,12	3,02
	TAC(C(D))	0,94	1,98	2,92
	GTA(R(D))	1,12	1,90	3,02
TGA	TGA(D)	2,21	1,46	3,67
	TCA(RC(D))	2,09	1,80	3,89
	ACT(C(D))	1,46	2,21	3,67
	AGT(R(D))	1,80	2,09	3,89
TAG	TAG(D)	1,29	1,30	2,59
	CTA(RC(D))	1,08	1,34	2,42
	ATC(C(D))	1,30	1,29	2,59
	GAT(R(D))	1,34	1,08	2,42
TAA	TAA(D)	1,65	2,26	3,91
	TTA(RC(D))	1,75	2,22	3,97
	ATT(C(D))	2,26	1,65	3,91
	AAT(R(D))	2,22	1,75	3,97
AAC	AAC(D)	1,29	2,01	3,30
	GTT(RC(D))	1,67	1,65	3,32
	TTG(C(D))	2,01	1,29	3,30
	CAA(R(D))	1,65	1,67	3,32
AAG	AAG(D)	1,75	2,34	4,10
	CTT(RC(D))	2,18	1,97	4,15
	TTC(C(D))	2,34	1,75	4,10
	GAA(R(D))	1,97	2,18	4,15
ACA	ACA(D)	0,89	1,27	2,16
	TGT(RC(D))	1,27	0,89	2,16
	TGT(C(D))	1,27	0,89	2,16
	ACA(R(D))	0,89	1,27	2,16
ATA	ATA(D)	0,84	0,87	1,71
	TAT(RC(D))	0,87	0,84	1,71
	TAT(C(D))	0,87	0,84	1,71
	ATA(R(D))	0,84	0,84	1,68
AGA	AGA(D)	0,97	1,32	2,29
	TCT(RC(D))	1,32	0,97	2,29
	TCT(C(D))	1,32	0,97	2,29
	AGA(R(D))	0,97	1,32	2,29
AAA	AAA(D)	1,46	1,88	3,33
	TTT(RC(D))	1,88	1,46	3,33
	TTT(C(D))	1,88	1,46	3,33
	AAA(R(D))	1,46	1,88	3,33

Composition of DNA sequence NBPF3 introns

Base	No.of bases	%
A	11019	26,85%
C	8656	21,10%
G	8818	21,49%
T	12540	30,56%
N	0	0,00%
Sum	41033	100,00%

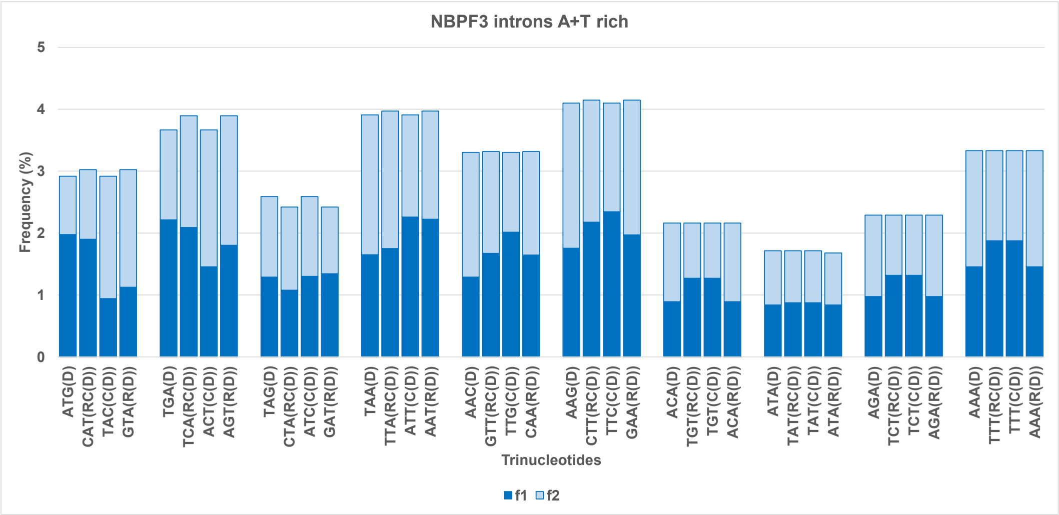

						C+G rich						
**D**	**f**	**%**	**RC**	**f**	**%**	**C**	**f**	**%**	**R**	**f**	**%**	
**CGT**	157,00	0,34	ACG	129,00	0,28	GCA	654,00	1,42	TGC	676,00	1,47	IIa
**GTC**	571,00	1,24	GAC	475,00	1,03	CAG	995,00	2,16	CTG	1094,00	2,37	
**GCT**	662,00	1,44	AGC	586,00	1,27	CGA	123,00	0,27	TCG	140,00	0,30	
**GCC**	537,00	1,17	GGC	605,00	1,31	CGG	139,00	0,30	CCG	136,00	0,30	IIb
**CCA**	834,00	1,81	TGG	933,00	2,02	GGT	576,00	1,25	ACC	550,00	1,19	
**CCT**	954,00	2,07	AGG	858,00	1,86	GGA	749,00	1,63	TCC	837,00	1,82	
**CAC**	674,00	1,46	GTG	855,00	1,86	GTG*			CAC*			IIc
**CGC**	117,00	0,25	GCG	131,00	0,28	GCG*			CGC*			
**CTC**	906,00	1,97	GAG	791,00	1,72	GAG*			CTC*			
**CCC**	599,00	1,30	GGG	638,00	1,38	GGG*			CCC*			

Quadruplet		f1	f2	fu
CGT	CGT(D)	0,34	1,42	1,76
	ACG(RC(D))	0,28	1,47	1,75
	GCA(C(D))	1,42	0,34	1,76
	TGC(R(D))	1,47	0,28	1,75
GTC	GTC(D)	1,24	2,16	3,40
	GAC(RC(D))	1,03	2,37	3,41
	CAG(C(D))	2,16	1,24	3,40
	CTG(R(D))	2,37	1,03	3,41
GCT	GCT(D)	1,44	0,27	1,70
	AGC(RC(D))	1,27	0,30	1,58
	CGA(C(D))	0,27	1,44	1,70
	TCG(R(D))	0,30	1,27	1,58
GCC	GCC(D)	1,17	0,30	1,47
	GGC(RC(D)	1,31	0,30	1,61
	CGG(C(D))	0,30	1,17	1,47
	CCG(R(D))	0,30	1,31	1,61
CCA	CCA(D)	1,81	1,25	3,06
	TGG(RC(D))	2,02	1,19	3,22
	GGT(C(D))	1,25	1,81	3,06
	ACC(R(D))	1,19	2,02	3,22
CCT	CCT(D)	2,07	1,63	3,70
	AGG(RC(D))	1,86	1,82	3,68
	GGA(C(D))	1,63	2,07	3,70
	TCC(R(D))	1,82	1,86	3,68
CAC	CAC(D)	0,73	0,93	1,66
	GTG(RC(D))	0,93	0,73	1,66
	GTG(C(D))	0,93	0,73	1,66
	CAC(R(D))	0,73	0,93	1,66
CGC	CGC(D)	0,13	0,14	0,27
	GCG(RC(D)	0,14	0,13	0,27
	GCG(C(D))	0,14	0,13	0,27
	CGC(R(D))	0,13	0,14	0,27
CTC	CTC(D)	0,98	0,86	1,84
	GAG(RC(D))	0,86	0,98	1,84
	GAG(C(D))	0,86	0,98	1,84
	CTC(R(D))	0,98	0,86	1,84
CCC	CCC(D)	0,65	0,69	1,34
	GGG(RC(D)	0,69	0,65	1,34
	GGG(C(D))	0,69	0,65	1,34
	CCC(R(D))	0,65	0,69	1,34

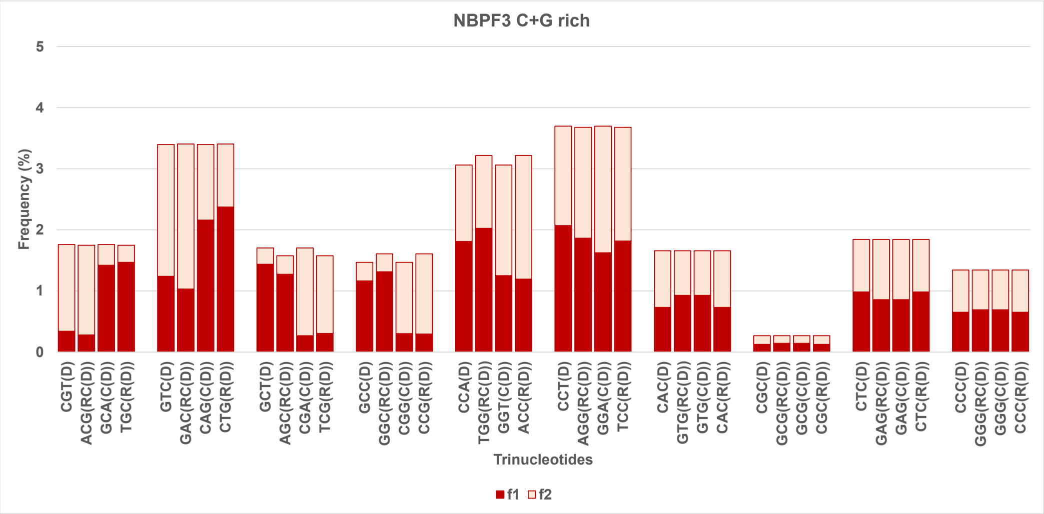

NBPF3 exons

C+G rich

D	f	%	RC	f	%	C	f	%	R	f	%	
**CGT**	15,00	0,41	ACG	24,00	0,65	GCA	47,00	1,27	TGC	63,00	1,70	IIa
**GTC**	52,00	1,40	GAC	61,00	1,65	CAG	107,00	2,89	CTG	104,00	2,81	
**GCT**	62,00	1,67	AGC	63,00	1,70	CGA	18,00	0,49	TCG	8,00	0,22	
**GCC**	68,00	1,84	GGC	59,00	1,59	CGG	24,00	0,65	CCG	17,00	0,46	IIb
**CCA**	82,00	2,22	TGG	73,00	1,97	GGT	41,00	1,11	ACC	46,00	1,24	
**CCT**	79,00	2,13	AGG	98,00	2,65	GGA	95,00	2,57	TCC	64,00	1,73	
**CAC**	55,00	1,49	GTG	50,00	1,35	GTG*			CAC*			IIc
**CGC**	12,00	0,32	GCG	20,00	0,54	GCG*			CGC*			
**CTC**	74,00	2,00	GAG	91,00	2,46	GAG*			CTC*			
**CCC**	56,00	1,51	GGG	41,00	1,11	GGG*			CCC*			

Quadruplet		f1	f2	fu
CGT	CGT(D)	0,41	1,27	1,67
	ACG(RC(D))	0,65	1,70	2,35
	GCA(C(D))	1,27	0,41	1,67
	TGC(R(D))	1,70	0,65	2,35
GTC	GTC(D)	1,40	2,89	4,29
	GAC(RC(D))	1,65	2,81	4,46
	CAG(C(D))	2,89	1,40	4,29
	CTG(R(D))	2,81	1,65	4,46
GCT	GCT(D)	1,67	0,49	2,16
	AGC(RC(D))	1,70	0,22	1,92
	CGA(C(D))	0,49	1,67	2,16
	TCG(R(D))	0,22	1,70	1,92
GCC	GCC(D)	1,84	0,65	2,49
	GGC(RC(D))	1,59	0,46	2,05
	CGG(C(D))	0,65	1,84	2,49
	CCG(R(D))	0,46	1,59	2,05
CCA	CCA(D)	2,22	1,11	3,32
	TGG(RC(D))	1,97	1,24	3,21
	GGT(C(D))	1,11	2,22	3,32
	ACC(R(D))	1,24	1,97	3,21
CCT	CCT(D)	2,13	2,57	4,70
	AGG(RC(D))	2,65	1,73	4,38
	GGA(C(D))	2,57	2,13	4,70
	TCC(R(D))	1,73	2,65	4,38
CAC	CAC(D)	0,74	0,68	1,42
	GTG(RC(D))	0,68	0,74	1,42
	GTG(C(D))	0,68	0,74	1,42
	CAC(R(D))	0,74	0,68	1,42
CGC	CGC(D)	0,16	0,27	0,43
	GCG(RC(D))	0,27	0,16	0,43
	GCG(C(D))	0,27	0,16	0,43
	CGC(R(D))	0,16	0,27	0,43
CTC	CTC(D)	1,00	1,23	2,23
	GAG(RC(D))	1,23	1,00	2,23
	GAG(C(D))	1,23	1,00	2,23
	CTC(R(D))	1,00	1,23	2,23
CCC	CCC(D)	0,76	0,55	1,31
	GGG(RC(D))	0,55	0,76	1,31
	GGG(C(D))	0,55	0,76	1,31
	CCC(R(D))	0,76	0,55	1,31

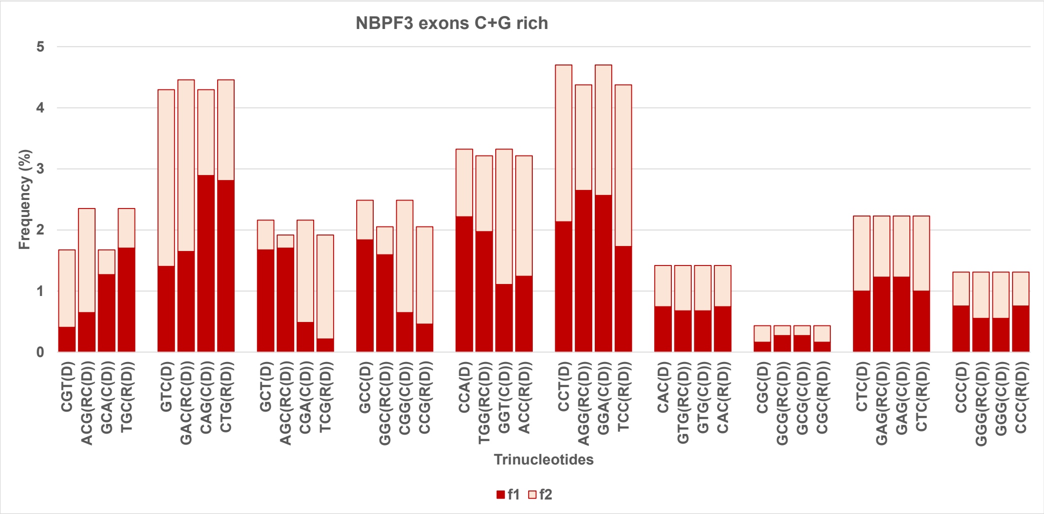

NBPF3 introns

C+G rich

D	f	%	RC	f	%	C	f	%	R	f	%	
**CGT**	135,00	0,33	ACG	97,00	0,24	GCA	589,00	1,44	TGC	594,00	1,45	IIa
**GTC**	505,00	1,23	GAC	402,00	0,98	CAG	861,00	2,10	CTG	962,00	2,34	
**GCT**	583,00	1,42	AGC	514,00	1,25	CGA	97,00	0,24	TCG	122,00	0,30	
**GCC**	450,00	1,10	GGC	521,00	1,27	CGG	102,00	0,25	CCG	108,00	0,26	IIb
**CCA**	736,00	1,79	TGG	831,00	2,03	GGT	510,00	1,24	ACC	490,00	1,19	
**CCT**	845,00	2,06	AGG	732,00	1,78	GGA	634,00	1,55	TCC	749,00	1,83	
**CAC**	599,00	1,46	GTG	776,00	1,89	GTG*			CAC*			IIc
**CGC**	89,00	0,22	GCG	96,00	0,23	GCG*			CGC*			
**CTC**	809,00	1,97	GAG	676,00	1,65	GAG*			CTC*			
**CCC**	523,00	1,27	GGG	568,00	1,38	GGG*			CCC*			

Quadruplet		f1	f2	fu
CGT	CGT(D)	0,33	1,44	1,76
	ACG(RC(D))	0,24	1,45	1,68
	GCA(C(D))	1,44	0,33	1,76
	TGC(R(D))	1,45	0,24	1,68
GTC	GTC(D)	1,23	2,10	3,33
	GAC(RC(D))	0,98	2,34	3,32
	CAG(C(D))	2,10	1,23	3,33
	CTG(R(D))	2,34	0,98	3,32
GCT	GCT(D)	1,42	0,24	1,66
	AGC(RC(D))	1,25	0,30	1,55
	CGA(C(D))	0,24	1,42	1,66
	TCG(R(D))	0,30	1,25	1,55
GCC	GCC(D)	1,10	0,25	1,35
	GGC(RC(D))	1,27	0,26	1,53
	CGG(C(D))	0,25	1,10	1,35
	CCG(R(D))	0,26	1,27	1,53
CCA	CCA(D)	1,79	1,24	3,04
	TGG(RC(D))	2,03	1,19	3,22
	GGT(C(D))	1,24	1,79	3,04
	ACC(R(D))	1,19	2,03	3,22
CCT	CCT(D)	2,06	1,55	3,60
	AGG(RC(D))	1,78	1,83	3,61
	GGA(C(D))	1,55	2,06	3,60
	TCC(R(D))	1,83	1,78	3,61
CAC	CAC(D)	0,73	0,95	1,68
	GTG(RC(D))	0,95	0,73	1,68
	GTG(C(D))	0,95	0,73	1,68
	CAC(R(D))	0,73	0,95	1,68
CGC	CGC(D)	0,11	0,12	0,23
	GCG(RC(D))	0,12	0,11	0,23
	GCG(C(D))	0,12	0,11	0,23
	CGC(R(D))	0,11	0,12	0,23
CTC	CTC(D)	0,99	0,82	1,81
	GAG(RC(D))	0,82	0,99	1,81
	GAG(C(D))	0,82	0,99	1,81
	CTC(R(D))	0,99	0,82	1,81
CCC	CCC(D)	0,64	0,69	1,33
	GGG(RC(D))	0,69	0,64	1,33
	GGG(C(D))	0,69	0,64	1,33
	CCC(R(D))	0,64	0,69	1,33

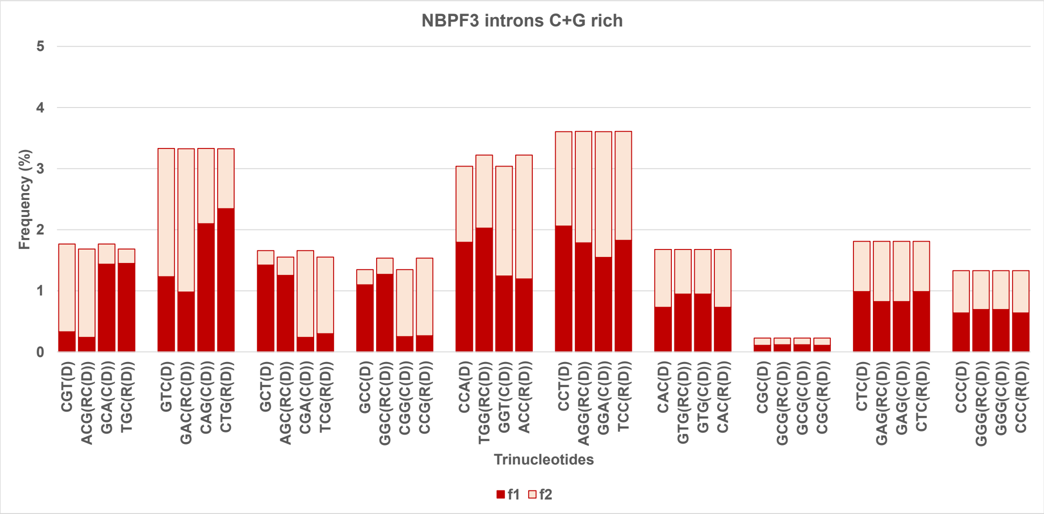

NBPF4 sequence

						A-T rich						
**D**	**f**	**%**	**RC**	**f**	**%**	**C**	**f**	**%**	**R**	**f**	**%**	
**ATG**	437,00	1,99	CAT	421,00	1,92	TAC	194,00	0,88	GTA	199,00	0,91	Ia
**TGA**	497,00	2,27	TCA	470,00	2,14	ACT	359,00	1,64	AGT	333,00	1,52	
**TAG**	226,00	1,03	CTA	242,00	1,10	ATC	297,00	1,35	GAT	306,00	1,39	
**TAA**	280,00	1,28	TTA	305,00	1,39	ATT	422,00	1,92	AAT	383,00	1,75	Ib
**AAC**	297,00	1,35	GTT	290,00	1,32	TTG	410,00	1,87	CAA	340,00	1,55	
**AAG**	404,00	1,84	CTT	480,00	2,19	TTC	477,00	2,17	GAA	464,00	2,11	
**ACA**	413,00	1,88	TGT	555,00	2,53	TGT*			ACA*			Ic
**ATA**	271,00	1,24	TAT	317,00	1,44	TAT*			ATA*			
**AGA**	500,00	2,28	TCT	624,00	2,84	TCT*			AGA*			
**AAA**	584,00	2,66	TTT	619,00	2,82	TTT*			AAA*			

Quadruplet		f1	f2	fu
ATG	ATG(D)	1,99	0,88	2,88
	CAT(RC(D))	1,92	0,91	2,83
	TAC(C(D))	0,88	1,99	2,88
	GTA(R(D))	0,91	1,92	2,83
TGA	TGA(D)	2,27	1,64	3,90
	TCA(RC(D))	2,14	1,52	3,66
	ACT(C(D))	1,64	2,27	3,90
	AGT(R(D))	1,52	2,14	3,66
TAG	TAG(D)	1,03	1,35	2,38
	CTA(RC(D))	1,10	1,39	2,50
	ATC(C(D))	1,35	1,03	2,38
	GAT(R(D))	1,39	1,10	2,50
TAA	TAA(D)	1,28	1,92	3,20
	TTA(RC(D))	1,39	1,75	3,14
	ATT(C(D))	1,92	1,28	3,20
	AAT(R(D))	1,75	1,39	3,14
AAC	AAC(D)	1,35	1,87	3,22
	GTT(RC(D))	1,32	1,55	2,87
	TTG(C(D))	1,87	1,35	3,22
	CAA(R(D))	1,55	1,32	2,87
AAG	AAG(D)	1,84	2,17	4,02
	CTT(RC(D))	2,19	2,11	4,30
	TTC(C(D))	2,17	1,84	4,02
	GAA(R(D))	2,11	2,19	4,30
ACA	ACA(D)	0,94	1,26	2,21
	TGT(RC(D))	1,26	0,94	2,21
	TGT(C(D))	1,26	0,94	2,21
	ACA(R(D))	0,94	1,26	2,21
ATA	ATA(D)	0,62	0,72	1,34
	TAT(RC(D))	0,72	0,62	1,34
	TAT(C(D))	0,72	0,62	1,34
	ATA(R(D))	0,62	0,62	1,24
AGA	AGA(D)	1,14	1,42	2,56
	TCT(RC(D))	1,42	1,14	2,56
	TCT(C(D))	1,42	1,14	2,56
	AGA(R(D))	1,14	1,42	2,56
AAA	AAA(D)	1,33	1,41	2,74
	TTT(RC(D))	1,41	1,33	2,74
	TTT(C(D))	1,41	1,33	2,74
	AAA(R(D))	1,33	1,41	2,74

Composition of DNA sequence in NBPF4

Base	No.of bases	%
A	5802	26,44%
C	5089	23,19%
G	4806	21,90%
T	6244	28,46%
N	0	0,00%
Sum	21941	100,00%

% of coding bases from NBPF4 (including N bases)

**83,14%**

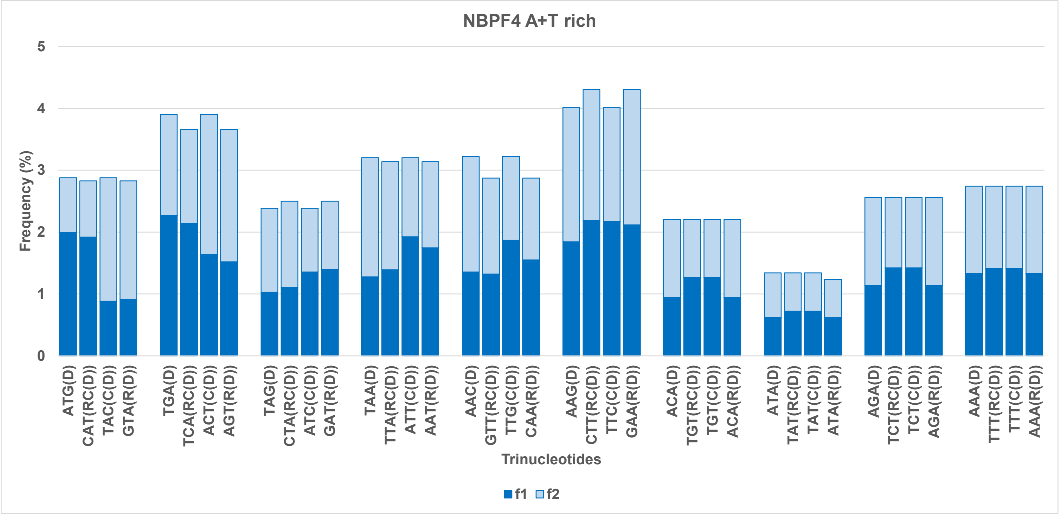

NBPF4 exons

A-T rich

D	f	%	RC	f	%	C	f	%	R	f	%	
**ATG**	45,00	1,81	CAT	29,00	1,17	TAC	24,00	0,97	GTA	11,00	0,44	Ia
**TGA**	65,00	2,62	TCA	58,00	2,34	ACT	48,00	1,93	AGT	38,00	1,53	
**TAG**	13,00	0,52	CTA	14,00	0,56	ATC	23,00	0,93	GAT	37,00	1,49	
**TAA**	11,00	0,44	TTA	15,00	0,60	ATT	21,00	0,85	AAT	30,00	1,21	Ib
**AAC**	31,00	1,25	GTT	28,00	1,13	TTG	42,00	1,69	CAA	61,00	2,46	
**AAG**	75,00	3,02	CTT	43,00	1,73	TTC	35,00	1,41	GAA	64,00	2,58	
**ACA**	43,00	1,73	TGT	32,00	1,29	TGT*			ACA*			Ic
**ATA**	15,00	0,60	TAT	7,00	0,28	TAT*			ATA*			
**AGA**	77,00	3,10	TCT	35,00	1,41	TCT*			AGA*			
**AAA**	56,00	2,26	TTT	25,00	1,01	TTT*			AAA*			

Quadruplet		f1	f2	fu
ATG	ATG(D)	1,81	0,97	2,78
	CAT(RC(D))	1,17	0,44	1,61
	TAC(C(D))	0,97	1,81	2,78
	GTA(R(D))	0,44	1,17	1,61
TGA	TGA(D)	2,62	1,93	4,55
	TCA(RC(D))	2,34	1,53	3,87
	ACT(C(D))	1,93	2,62	4,55
	AGT(R(D))	1,53	2,34	3,87
TAG	TAG(D)	0,52	0,93	1,45
	CTA(RC(D))	0,56	1,49	2,05
	ATC(C(D))	0,93	0,52	1,45
	GAT(R(D))	1,49	0,56	2,05
TAA	TAA(D)	0,44	0,85	1,29
	TTA(RC(D))	0,60	1,21	1,81
	ATT(C(D))	0,85	0,44	1,29
	AAT(R(D))	1,21	0,60	1,81
AAC	AAC(D)	1,25	1,69	2,94
	GTT(RC(D))	1,13	2,46	3,58
	TTG(C(D))	1,69	1,25	2,94
	CAA(R(D))	2,46	1,13	3,58
AAG	AAG(D)	3,02	1,41	4,43
	CTT(RC(D))	1,73	2,58	4,31
	TTC(C(D))	1,41	3,02	4,43
	GAA(R(D))	2,58	1,73	4,31
ACA	ACA(D)	0,87	0,64	1,51
	TGT(RC(D))	0,64	0,87	1,51
	TGT(C(D))	0,64	0,87	1,51
	ACA(R(D))	0,87	0,64	1,51
ATA	ATA(D)	0,30	0,14	0,44
	TAT(RC(D))	0,14	0,30	0,44
	TAT(C(D))	0,14	0,30	0,44
	ATA(R(D))	0,30	0,30	0,60
AGA	AGA(D)	1,55	0,70	2,26
	TCT(RC(D))	0,70	1,55	2,26
	TCT(C(D))	0,70	1,55	2,26
	AGA(R(D))	1,55	0,70	2,26
AAA	AAA(D)	1,13	0,50	1,63
	TTT(RC(D))	0,50	1,13	1,63
	TTT(C(D))	0,50	1,13	1,63
	AAA(R(D))	1,13	0,50	1,63

Composition of DNA sequence in NBPF4 exons

Base	No.of bases	%
A	687	27,65%
C	658	26,48%
G	626	25,19%
T	514	20,68%
N	0	0,00%
Sum	2485	100,00%

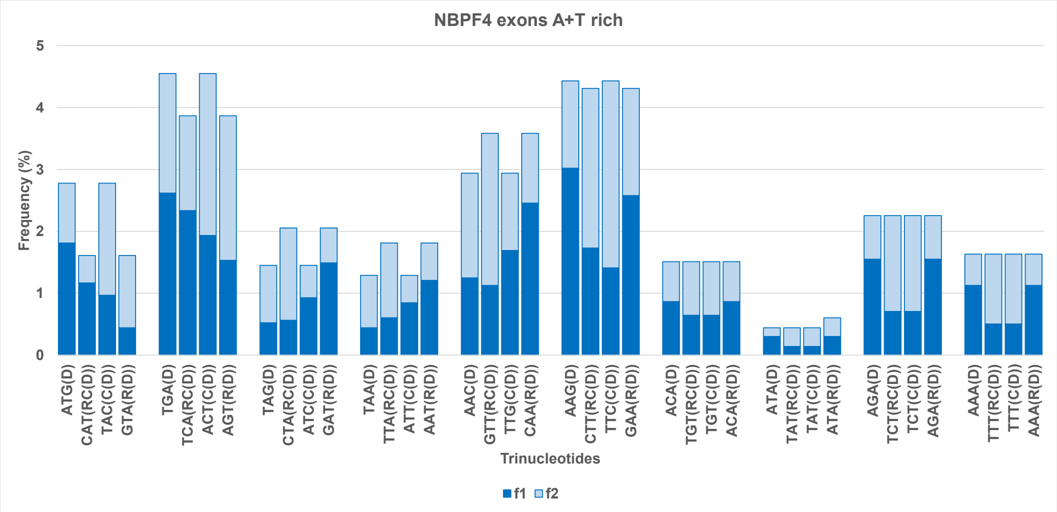

NBPF4 introns

A-T rich

D	f	%	RC	f	%	C	f	%	R	f	%	
**ATG**	367,00	2,01	CAT	365,00	2,00	TAC	160,00	0,88	GTA	174,00	0,95	Ia
**TGA**	397,00	2,18	TCA	384,00	2,11	ACT	294,00	1,61	AGT	270,00	1,48	
**TAG**	199,00	1,09	CTA	212,00	1,16	ATC	257,00	1,41	GAT	252,00	1,38	
**TAA**	245,00	1,34	TTA	271,00	1,49	ATT	376,00	2,06	AAT	330,00	1,81	Ib
**AAC**	244,00	1,34	GTT	249,00	1,37	TTG	345,00	1,89	CAA	262,00	1,44	
**AAG**	298,00	1,63	CTT	414,00	2,27	TTC	423,00	2,32	GAA	365,00	2,00	
**ACA**	340,00	1,86	TGT	496,00	2,72	TGT*			ACA*			Ic
**ATA**	238,00	1,30	TAT	291,00	1,60	TAT*			ATA*			
**AGA**	382,00	2,09	TCT	566,00	3,10	TCT*			AGA*			
**AAA**	474,00	2,60	TTT	559,00	3,06	TTT*			AAA*			

Quadruplet		f1	f2	fu
ATG	ATG(D)	2,01	0,88	2,89
	CAT(RC(D))	2,00	0,95	2,96
	TAC(C(D))	0,88	2,01	2,89
	GTA(R(D))	0,95	2,00	2,96
TGA	TGA(D)	2,18	1,61	3,79
	TCA(RC(D))	2,11	1,48	3,59
	ACT(C(D))	1,61	2,18	3,79
	AGT(R(D))	1,48	2,11	3,59
TAG	TAG(D)	1,09	1,41	2,50
	CTA(RC(D))	1,16	1,38	2,54
	ATC(C(D))	1,41	1,09	2,50
	GAT(R(D))	1,38	1,16	2,54
TAA	TAA(D)	1,34	2,06	3,40
	TTA(RC(D))	1,49	1,81	3,29
	ATT(C(D))	2,06	1,34	3,40
	AAT(R(D))	1,81	1,49	3,29
AAC	AAC(D)	1,34	1,89	3,23
	GTT(RC(D))	1,37	1,44	2,80
	TTG(C(D))	1,89	1,34	3,23
	CAA(R(D))	1,44	1,37	2,80
AAG	AAG(D)	1,63	2,32	3,95
	CTT(RC(D))	2,27	2,00	4,27
	TTC(C(D))	2,32	1,63	3,95
	GAA(R(D))	2,00	2,27	4,27
ACA	ACA(D)	0,93	1,36	2,29
	TGT(RC(D))	1,36	0,93	2,29
	TGT(C(D))	1,36	0,93	2,29
	ACA(R(D))	0,93	1,36	2,29
ATA	ATA(D)	0,65	0,80	1,45
	TAT(RC(D))	0,80	0,65	1,45
	TAT(C(D))	0,80	0,65	1,45
	ATA(R(D))	0,65	0,65	1,30
AGA	AGA(D)	1,05	1,55	2,60
	TCT(RC(D))	1,55	1,05	2,60
	TCT(C(D))	1,55	1,05	2,60
	AGA(R(D))	1,05	1,55	2,60
AAA	AAA(D)	1,30	1,53	2,83
	TTT(RC(D))	1,53	1,30	2,83
	TTT(C(D))	1,53	1,30	2,83
	AAA(R(D))	1,30	1,53	2,83

Composition of DNA sequence NBPF4 introns

Base	No.of bases	%
A	4729	25,92%
C	4192	22,98%
G	3917	21,47%
T	5404	29,62%
N	0	0,00%
Sum	18242	100,00%

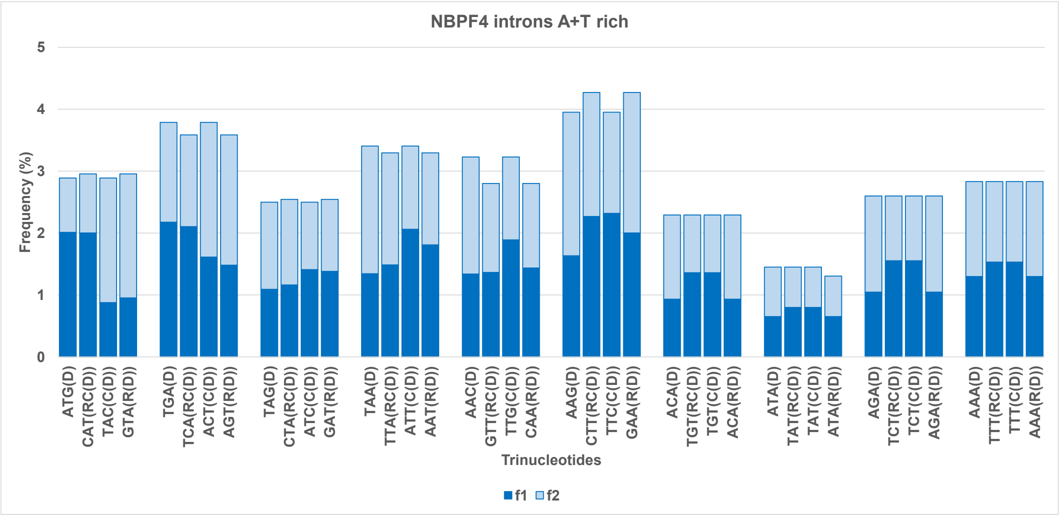

NBPF4 sequence

						C+G rich						
**D**	**f**	**%**	**RC**	**f**	**%**	**C**	**f**	**%**	**R**	**f**	**%**	
**CGT**	63,00	0,29	ACG	57,00	0,26	GCA	344,00	1,57	TGC	361,00	1,65	IIa
**GTC**	305,00	1,39	GAC	263,00	1,20	CAG	529,00	2,41	CTG	583,00	2,66	
**GCT**	305,00	1,39	AGC	314,00	1,43	CGA	49,00	0,22	TCG	48,00	0,22	
**GCC**	272,00	1,24	GGC	255,00	1,16	CGG	55,00	0,25	CCG	53,00	0,24	IIb
**CCA**	438,00	2,00	TGG	419,00	1,91	GGT	244,00	1,11	ACC	300,00	1,37	
**CCT**	522,00	2,38	AGG	431,00	1,96	GGA	406,00	1,85	TCC	441,00	2,01	
**CAC**	375,00	1,71	GTG	402,00	1,83	GTG*			CAC*			IIc
**CGC**	37,00	0,17	GCG	46,00	0,21	GCG*			CGC*			
**CTC**	504,00	2,30	GAG	419,00	1,91	GAG*			CTC*			
**CCC**	397,00	1,81	GGG	286,00	1,30	GGG*			CCC*			

Quadruplet		f1	f2	fu
CGT	CGT(D)	0,29	1,57	1,86
	ACG(RC(D))	0,26	1,65	1,91
	GCA(C(D))	1,57	0,29	1,86
	TGC(R(D))	1,65	0,26	1,91
GTC	GTC(D)	1,39	2,41	3,80
	GAC(RC(D))	1,20	2,66	3,86
	CAG(C(D))	2,41	1,39	3,80
	CTG(R(D))	2,66	1,20	3,86
GCT	GCT(D)	1,39	0,22	1,61
	AGC(RC(D))	1,43	0,22	1,65
	CGA(C(D))	0,22	1,39	1,61
	TCG(R(D))	0,22	1,43	1,65
GCC	GCC(D)	1,24	0,25	1,49
	GGC(RC(D))	1,16	0,24	1,40
	CGG(C(D))	0,25	1,24	1,49
	CCG(R(D))	0,24	1,16	1,40
CCA	CCA(D)	2,00	1,11	3,11
	TGG(RC(D))	1,91	1,37	3,28
	GGT(C(D))	1,11	2,00	3,11
	ACC(R(D))	1,37	1,91	3,28
CCT	CCT(D)	2,38	1,85	4,23
	AGG(RC(D))	1,96	2,01	3,97
	GGA(C(D))	1,85	2,38	4,23
	TCC(R(D))	2,01	1,96	3,97
CAC	CAC(D)	0,85	0,92	1,77
	GTG(RC(D))	0,92	0,85	1,77
	GTG(C(D))	0,92	0,85	1,77
	CAC(R(D))	0,85	0,92	1,77
CGC	CGC(D)	0,08	0,10	0,19
	GCG(RC(D))	0,10	0,08	0,19
	GCG(C(D))	0,10	0,08	0,19
	CGC(R(D))	0,08	0,10	0,19
CTC	CTC(D)	1,15	0,95	2,10
	GAG(RC(D))	0,95	1,15	2,10
	GAG(C(D))	0,95	1,15	2,10
	CTC(R(D))	1,15	0,95	2,10
CCC	CCC(D)	0,90	0,65	1,56
	GGG(RC(D))	0,65	0,90	1,56
	GGG(C(D))	0,65	0,90	1,56
	CCC(R(D))	0,90	0,65	1,56

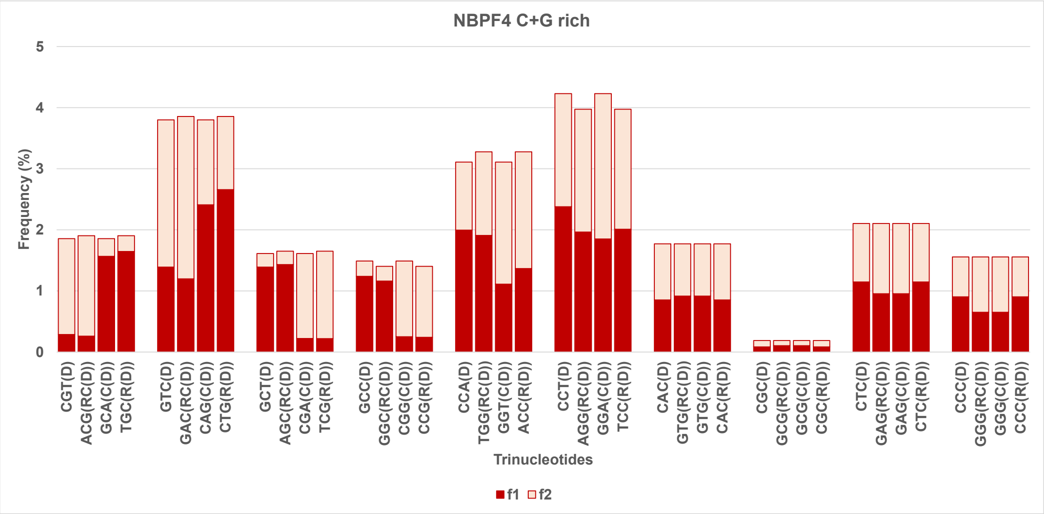

NBPF4 exons

C+G rich

D	f	%	RC	f	%	C	f	%	R	f	%	
**CGT**	10,00	0,40	ACG	10,00	0,40	GCA	55,00	2,22	TGC	43,00	1,73	IIa
**GTC**	31,00	1,25	GAC	46,00	1,85	CAG	85,00	3,42	CTG	73,00	2,94	
**GCT**	56,00	2,26	AGC	67,00	2,70	CGA	11,00	0,44	TCG	9,00	0,36	
**GCC**	39,00	1,57	GGC	38,00	1,53	CGG	12,00	0,48	CCG	16,00	0,64	IIb
**CCA**	64,00	2,58	TGG	46,00	1,85	GGT	17,00	0,68	ACC	45,00	1,81	
**CCT**	57,00	2,30	AGG	63,00	2,54	GGA	66,00	2,66	TCC	53,00	2,13	
**CAC**	45,00	1,81	GTG	27,00	1,09	GTG*			CAC*			IIc
**CGC**	12,00	0,48	GCG	10,00	0,40	GCG*			CGC*			
**CTC**	66,00	2,66	GAG	72,00	2,90	GAG*			CTC*			
**CCC**	60,00	2,42	GGG	28,00	1,13	GGG*			CCC*			

Quadruplet		f1	f2	fu
CGT	CGT(D)	0,40	2,22	2,62
	ACG(RC(D))	0,40	1,73	2,13
	GCA(C(D))	2,22	0,40	2,62
	TGC(R(D))	1,73	0,40	2,13
GTC	GTC(D)	1,25	3,42	4,67
	GAC(RC(D))	1,85	2,94	4,79
	CAG(C(D))	3,42	1,25	4,67
	CTG(R(D))	2,94	1,85	4,79
GCT	GCT(D)	2,26	0,44	2,70
	AGC(RC(D))	2,70	0,36	3,06
	CGA(C(D))	0,44	2,26	2,70
	TCG(R(D))	0,36	2,70	3,06
GCC	GCC(D)	1,57	0,48	2,05
	GGC(RC(D))	1,53	0,64	2,17
	CGG(C(D))	0,48	1,57	2,05
	CCG(R(D))	0,64	1,53	2,17
CCA	CCA(D)	2,58	0,68	3,26
	TGG(RC(D))	1,85	1,81	3,66
	GGT(C(D))	0,68	2,58	3,26
	ACC(R(D))	1,81	1,85	3,66
CCT	CCT(D)	2,30	2,66	4,95
	AGG(RC(D))	2,54	2,13	4,67
	GGA(C(D))	2,66	2,30	4,95
	TCC(R(D))	2,13	2,54	4,67
CAC	CAC(D)	0,91	0,54	1,45
	GTG(RC(D))	0,54	0,91	1,45
	GTG(C(D))	0,54	0,91	1,45
	CAC(R(D))	0,91	0,54	1,45
CGC	CGC(D)	0,24	0,20	0,44
	GCG(RC(D))	0,20	0,24	0,44
	GCG(C(D))	0,20	0,24	0,44
	CGC(R(D))	0,24	0,20	0,44
CTC	CTC(D)	1,33	1,45	2,78
	GAG(RC(D))	1,45	1,33	2,78
	GAG(C(D))	1,45	1,33	2,78
	CTC(R(D))	1,33	1,45	2,78
CCC	CCC(D)	1,21	0,56	1,77
	GGG(RC(D))	0,56	1,21	1,77
	GGG(C(D))	0,56	1,21	1,77
	CCC(R(D))	1,21	0,56	1,77

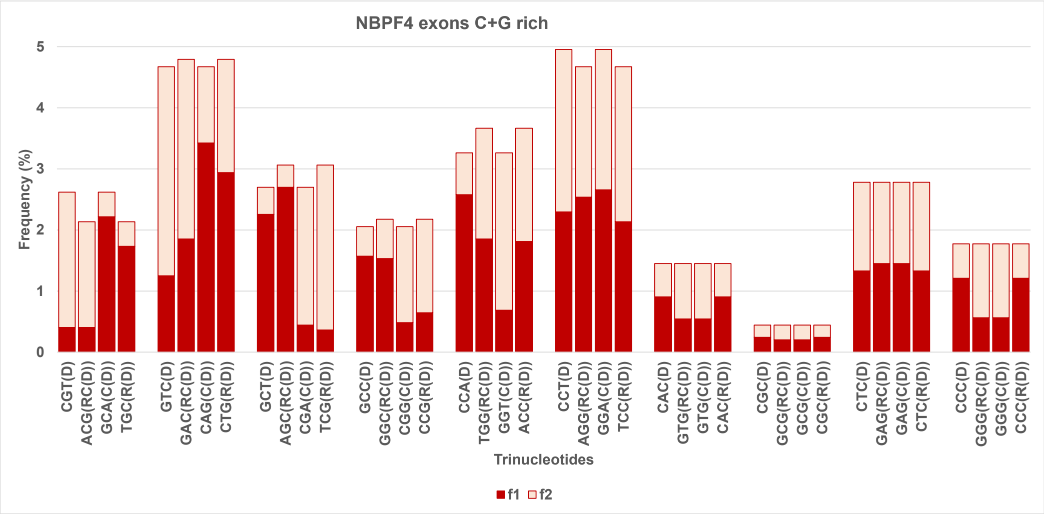

NBPF4 introns

						C+G rich						
**D**	**f**	**%**	**RC**	**f**	**%**	**C**	**f**	**%**	**R**	**f**	**%**	
**CGT**	50,00	0,27	ACG	45,00	0,25	GCA	273,00	1,50	TGC	301,00	1,65	IIa
**GTC**	253,00	1,39	GAC	198,00	1,09	CAG	411,00	2,25	CTG	484,00	2,65	
**GCT**	236,00	1,29	AGC	229,00	1,26	CGA	37,00	0,20	TCG	37,00	0,20	
**GCC**	221,00	1,21	GGC	210,00	1,15	CGG	40,00	0,22	CCG	35,00	0,19	IIb
**CCA**	354,00	1,94	TGG	354,00	1,94	GGT	211,00	1,16	ACC	236,00	1,29	
**CCT**	444,00	2,43	AGG	348,00	1,91	GGA	321,00	1,76	TCC	376,00	2,06	
**CAC**	313,00	1,72	GTG	352,00	1,93	GTG*			CAC*			IIc
**CGC**	24,00	0,13	GCG	34,00	0,19	GCG*			CGC*			
**CTC**	430,00	2,36	GAG	322,00	1,77	GAG*			CTC*			
**CCC**	317,00	1,74	GGG	245,00	1,34	GGG*			CCC*			

Quadruplet		f1	f2	fu
CGT	CGT(D)	0,27	1,50	1,77
	ACG(RC(D))	0,25	1,65	1,90
	GCA(C(D))	1,50	0,27	1,77
	TGC(R(D))	1,65	0,25	1,90
GTC	GTC(D)	1,39	2,25	3,64
	GAC(RC(D))	1,09	2,65	3,74
	CAG(C(D))	2,25	1,39	3,64
	CTG(R(D))	2,65	1,09	3,74
GCT	GCT(D)	1,29	0,20	1,50
	AGC(RC(D))	1,26	0,20	1,46
	CGA(C(D))	0,20	1,29	1,50
	TCG(R(D))	0,20	1,26	1,46
GCC	GCC(D)	1,21	0,22	1,43
	GGC(RC(D))	1,15	0,19	1,34
	CGG(C(D))	0,22	1,21	1,43
	CCG(R(D))	0,19	1,15	1,34
CCA	CCA(D)	1,94	1,16	3,10
	TGG(RC(D))	1,94	1,29	3,23
	GGT(C(D))	1,16	1,94	3,10
	ACC(R(D))	1,29	1,94	3,23
CCT	CCT(D)	2,43	1,76	4,19
	AGG(RC(D))	1,91	2,06	3,97
	GGA(C(D))	1,76	2,43	4,19
	TCC(R(D))	2,06	1,91	3,97
CAC	CAC(D)	0,86	0,96	1,82
	GTG(RC(D))	0,96	0,86	1,82
	GTG(C(D))	0,96	0,86	1,82
	CAC(R(D))	0,86	0,96	1,82
CGC	CGC(D)	0,07	0,09	0,16
	GCG(RC(D))	0,09	0,07	0,16
	GCG(C(D))	0,09	0,07	0,16
	CGC(R(D))	0,07	0,09	0,16
CTC	CTC(D)	1,18	0,88	2,06
	GAG(RC(D))	0,88	1,18	2,06
	GAG(C(D))	0,88	1,18	2,06
	CTC(R(D))	1,18	0,88	2,06
CCC	CCC(D)	0,87	0,67	1,54
	GGG(RC(D))	0,67	0,87	1,54
	GGG(C(D))	0,67	0,87	1,54
	CCC(R(D))	0,87	0,67	1,54

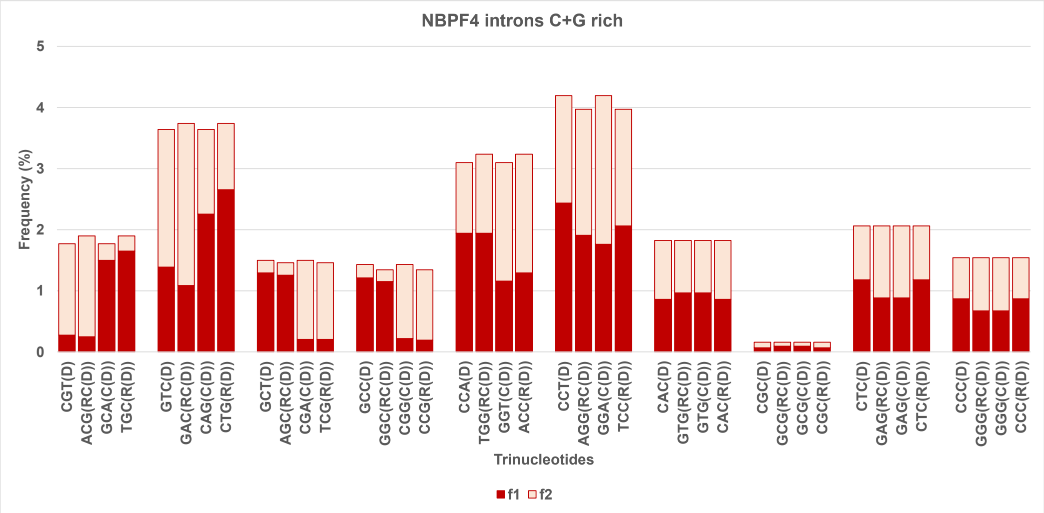

						A-T rich						
**D**	**f**	**%**	**RC**	**f**	**%**	**C**	**f**	**%**	**R**	**f**	**%**	
**ATG**	716,00	1,98	CAT	660,00	1,82	TAC	429,00	1,19	GTA	374,00	1,03	Ia
**TGA**	713,00	1,97	TCA	711,00	1,97	ACT	635,00	1,76	AGT	509,00	1,41	
**TAG**	476,00	1,32	CTA	510,00	1,41	ATC	507,00	1,40	GAT	496,00	1,37	
**TAA**	758,00	2,10	TTA	613,00	1,69	ATT	781,00	2,16	AAT	1014,00	2,80	Ib
**AAC**	686,00	1,90	GTT	413,00	1,14	TTG	593,00	1,64	CAA	851,00	2,35	
**AAG**	895,00	2,47	CTT	613,00	1,69	TTC	601,00	1,66	GAA	986,00	2,73	
**ACA**	873,00	2,41	TGT	668,00	1,85	TGT*			ACA*			Ic
**ATA**	859,00	2,37	TAT	693,00	1,92	TAT*			ATA*			
**AGA**	1124,00	3,11	TCT	676,00	1,87	TCT*			AGA*			
**AAA**	1855,00	5,13	TTT	923,00	2,55	TTT*			AAA*			

Quadruplet		f1	f2	fu
ATG	ATG(D)	1,98	1,19	3,17
	CAT(RC(D))	1,82	1,03	2,86
	TAC(C(D))	1,19	1,98	3,17
	GTA(R(D))	1,03	1,82	2,86
TGA	TGA(D)	1,97	1,76	3,73
	TCA(RC(D))	1,97	1,41	3,37
	ACT(C(D))	1,76	1,97	3,73
	AGT(R(D))	1,41	1,97	3,37
TAG	TAG(D)	1,32	1,40	2,72
	CTA(RC(D))	1,41	1,37	2,78
	ATC(C(D))	1,40	1,32	2,72
	GAT(R(D))	1,37	1,41	2,78
TAA	TAA(D)	2,10	2,16	4,25
	TTA(RC(D))	1,69	2,80	4,50
	ATT(C(D))	2,16	2,10	4,25
	AAT(R(D))	2,80	1,69	4,50
AAC	AAC(D)	1,90	1,64	3,54
	GTT(RC(D))	1,14	2,35	3,49
	TTG(C(D))	1,64	1,90	3,54
	CAA(R(D))	2,35	1,14	3,49
AAG	AAG(D)	2,47	1,66	4,14
	CTT(RC(D))	1,69	2,73	4,42
	TTC(C(D))	1,66	2,47	4,14
	GAA(R(D))	2,73	1,69	4,42
ACA	ACA(D)	1,21	0,92	2,13
	TGT(RC(D))	0,92	1,21	2,13
	TGT(C(D))	0,92	1,21	2,13
	ACA(R(D))	1,21	0,92	2,13
ATA	ATA(D)	1,19	0,96	2,15
	TAT(RC(D))	0,96	1,19	2,15
	TAT(C(D))	0,96	1,19	2,15
	ATA(R(D))	1,19	1,19	2,37
AGA	AGA(D)	1,55	0,93	2,49
	TCT(RC(D))	0,93	1,55	2,49
	TCT(C(D))	0,93	1,55	2,49
	AGA(R(D))	1,55	0,93	2,49
AAA	AAA(D)	2,56	1,28	3,84
	TTT(RC(D))	1,28	2,56	3,84
	TTT(C(D))	1,28	2,56	3,84
	AAA(R(D))	2,56	1,28	3,84

Composition of DNA sequence in NBPF5P

Base	No.of bases	%
A	12194	33,71%
C	7146	19,75%
G	7333	20,27%
T	9504	26,27%
N	0	0,00%
Sum	36177	100,00%

% of coding bases from NBPF5P (including N bases)

**92,24%**

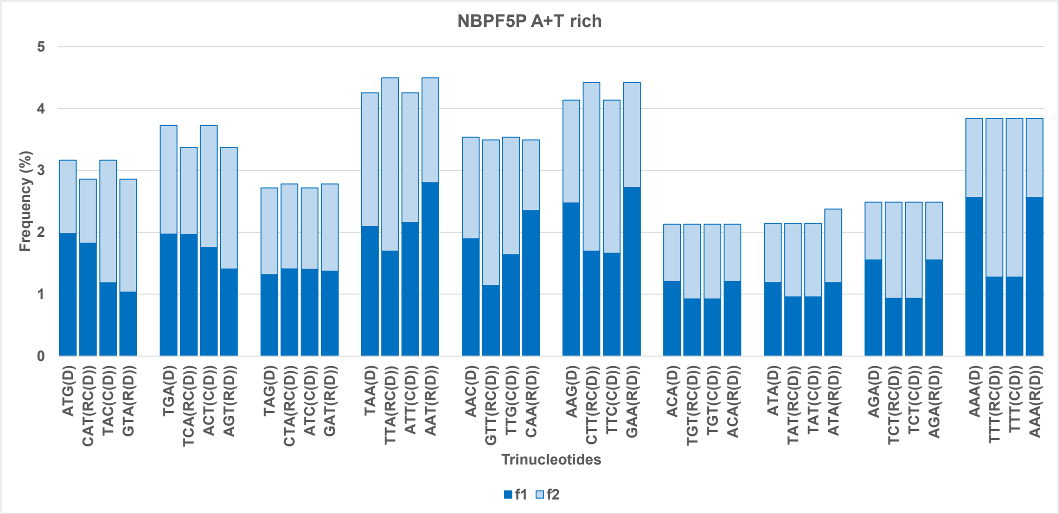

NBPF5P exons

A-T rich

D	f	%	RC	f	%	C	f	%	R	f	%	
**ATG**	30,00	1,87	CAT	25,00	1,56	TAC	15,00	0,94	GTA	11,00	0,69	Ia
**TGA**	46,00	2,87	TCA	41,00	2,56	ACT	24,00	1,50	AGT	29,00	1,81	
**TAG**	13,00	0,81	CTA	16,00	1,00	ATC	22,00	1,37	GAT	20,00	1,25	
**TAA**	16,00	1,00	TTA	14,00	0,87	ATT	17,00	1,06	AAT	26,00	1,62	Ib
**AAC**	25,00	1,56	GTT	20,00	1,25	TTG	26,00	1,62	CAA	31,00	1,93	
**AAG**	46,00	2,87	CTT	25,00	1,56	TTC	22,00	1,37	GAA	51,00	3,18	
**ACA**	27,00	1,68	TGT	25,00	1,56	TGT*			ACA*			Ic
**ATA**	17,00	1,06	TAT	15,00	0,94	TAT*			ATA*			
**AGA**	48,00	2,99	TCT	30,00	1,87	TCT*			AGA*			
**AAA**	38,00	2,37	TTT	26,00	1,62	TTT*			AAA*			

Quadruplet		f1	f2	fu
ATG	ATG(D)	1,87	0,94	2,81
	CAT(RC(D))	1,56	0,69	2,24
	TAC(C(D))	0,94	1,87	2,81
	GTA(R(D))	0,69	1,56	2,24
TGA	TGA(D)	2,87	1,50	4,36
	TCA(RC(D))	2,56	1,81	4,36
	ACT(C(D))	1,50	2,87	4,36
	AGT(R(D))	1,81	2,56	4,36
TAG	TAG(D)	0,81	1,37	2,18
	CTA(RC(D))	1,00	1,25	2,24
	ATC(C(D))	1,37	0,81	2,18
	GAT(R(D))	1,25	1,00	2,24
TAA	TAA(D)	1,00	1,06	2,06
	TTA(RC(D))	0,87	1,62	2,49
	ATT(C(D))	1,06	1,00	2,06
	AAT(R(D))	1,62	0,87	2,49
AAC	AAC(D)	1,56	1,62	3,18
	GTT(RC(D))	1,25	1,93	3,18
	TTG(C(D))	1,62	1,56	3,18
	CAA(R(D))	1,93	1,25	3,18
AAG	AAG(D)	2,87	1,37	4,24
	CTT(RC(D))	1,56	3,18	4,74
	TTC(C(D))	1,37	2,87	4,24
	GAA(R(D))	3,18	1,56	4,74
ACA	ACA(D)	0,84	0,78	1,62
	TGT(RC(D))	0,78	0,84	1,62
	TGT(C(D))	0,78	0,84	1,62
	ACA(R(D))	0,84	0,78	1,62
ATA	ATA(D)	0,53	0,47	1,00
	TAT(RC(D))	0,47	0,53	1,00
	TAT(C(D))	0,47	0,53	1,00
	ATA(R(D))	0,53	0,53	1,06
AGA	AGA(D)	1,50	0,94	2,43
	TCT(RC(D))	0,94	1,50	2,43
	TCT(C(D))	0,94	1,50	2,43
	AGA(R(D))	1,50	0,94	2,43
AAA	AAA(D)	1,18	0,81	2,00
	TTT(RC(D))	0,81	1,18	2,00
	TTT(C(D))	0,81	1,18	2,00
	AAA(R(D))	1,18	0,81	2,00

Composition of DNA sequence in NBPF5P exons

Base	No.of bases	%
A	457	28,46%
C	394	24,53%
G	380	23,66%
T	375	23,35%
N	0	0,00%
Sum	1606	100,00%

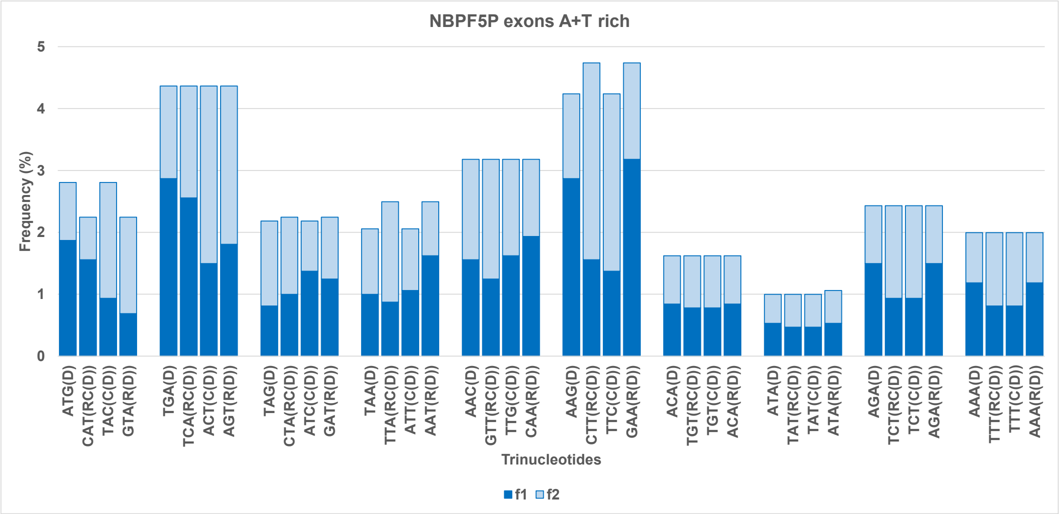

NBPF5P introns

A-T rich

D	f	%	RC	f	%	C	f	%	R	f	%	
**ATG**	667,00	2,00	CAT	621,00	1,86	TAC	399,00	1,20	GTA	348,00	1,04	Ia
**TGA**	637,00	1,91	TCA	651,00	1,95	ACT	587,00	1,76	AGT	461,00	1,38	
**TAG**	444,00	1,33	CTA	478,00	1,43	ATC	473,00	1,42	GAT	456,00	1,37	
**TAA**	715,00	2,14	TTA	569,00	1,71	ATT	728,00	2,18	AAT	954,00	2,86	Ib
**AAC**	641,00	1,92	GTT	380,00	1,14	TTG	546,00	1,64	CAA	795,00	2,38	
**AAG**	813,00	2,44	CTT	564,00	1,69	TTC	557,00	1,67	GAA	898,00	2,69	
**ACA**	821,00	2,46	TGT	626,00	1,88	TGT*			ACA*			Ic
**ATA**	819,00	2,45	TAT	656,00	1,97	TAT*			ATA*			
**AGA**	1033,00	3,10	TCT	625,00	1,87	TCT*			AGA*			
**AAA**	1753,00	5,25	TTT	844,00	2,53	TTT*			AAA*			

Quadruplet		f1	f2	fu
ATG	ATG(D)	2,00	1,20	3,19
	CAT(RC(D))	1,86	1,04	2,90
	TAC(C(D))	1,20	2,00	3,19
	GTA(R(D))	1,04	1,86	2,90
TGA	TGA(D)	1,91	1,76	3,67
	TCA(RC(D))	1,95	1,38	3,33
	ACT(C(D))	1,76	1,91	3,67
	AGT(R(D))	1,38	1,95	3,33
TAG	TAG(D)	1,33	1,42	2,75
	CTA(RC(D))	1,43	1,37	2,80
	ATC(C(D))	1,42	1,33	2,75
	GAT(R(D))	1,37	1,43	2,80
TAA	TAA(D)	2,14	2,18	4,32
	TTA(RC(D))	1,71	2,86	4,56
	ATT(C(D))	2,18	2,14	4,32
	AAT(R(D))	2,86	1,71	4,56
AAC	AAC(D)	1,92	1,64	3,56
	GTT(RC(D))	1,14	2,38	3,52
	TTG(C(D))	1,64	1,92	3,56
	CAA(R(D))	2,38	1,14	3,52
AAG	AAG(D)	2,44	1,67	4,11
	CTT(RC(D))	1,69	2,69	4,38
	TTC(C(D))	1,67	2,44	4,11
	GAA(R(D))	2,69	1,69	4,38
ACA	ACA(D)	1,23	0,94	2,17
	TGT(RC(D))	0,94	1,23	2,17
	TGT(C(D))	0,94	1,23	2,17
	ACA(R(D))	1,23	0,94	2,17
ATA	ATA(D)	1,23	0,98	2,21
	TAT(RC(D))	0,98	1,23	2,21
	TAT(C(D))	0,98	1,23	2,21
	ATA(R(D))	1,23	1,23	2,45
AGA	AGA(D)	1,55	0,94	2,48
	TCT(RC(D))	0,94	1,55	2,48
	TCT(C(D))	0,94	1,55	2,48
	AGA(R(D))	1,55	0,94	2,48
AAA	AAA(D)	2,63	1,26	3,89
	TTT(RC(D))	1,26	2,63	3,89
	TTT(C(D))	1,26	2,63	3,89
	AAA(R(D))	2,63	1,26	3,89

Composition of DNA sequence NBPF5P introns

Base	No.of bases	%
A	11331	33,955%
C	6545	19,613%
G	6711	20,110%
T	8784	26,322%
N	0	0,000%
Sum	33371	100,00%

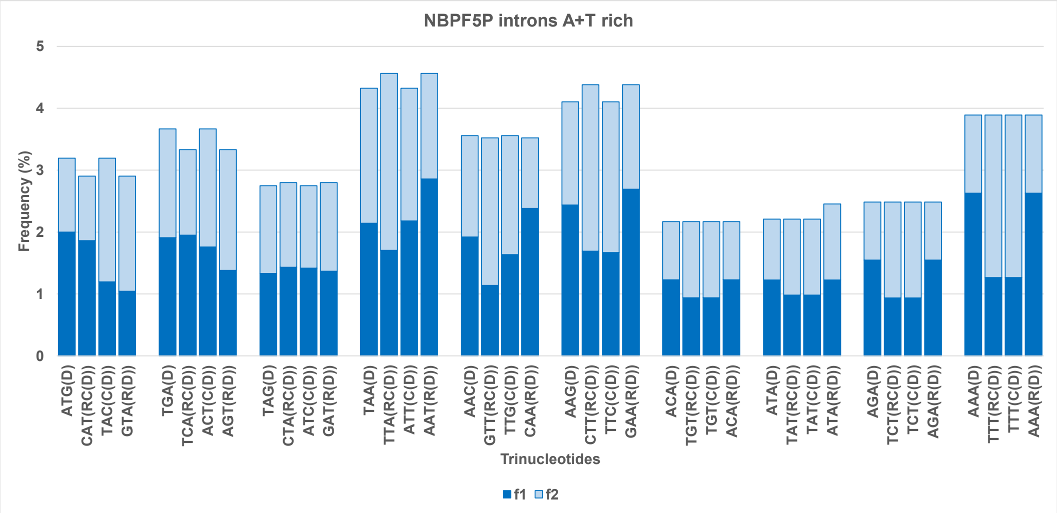

NBPF5P sequence

C+G rich												
**D**	**f**	**%**	**RC**	**f**	**%**	**C**	**f**	**%**	**R**	**f**	**%**	
**CGT**	58,00	0,16	ACG	75,00	0,21	GCA	572,00	1,58	TGC	461,00	1,27	IIa
**GTC**	287,00	0,79	GAC	421,00	1,16	CAG	795,00	2,20	CTG	687,00	1,90	
**GCT**	447,00	1,24	AGC	503,00	1,39	CGA	60,00	0,17	TCG	47,00	0,13	
**GCC**	339,00	0,94	GGC	396,00	1,09	CGG	57,00	0,16	CCG	51,00	0,14	IIb
**CCA**	692,00	1,91	TGG	669,00	1,85	GGT	354,00	0,98	ACC	495,00	1,37	
**CCT**	564,00	1,56	AGG	666,00	1,84	GGA	642,00	1,77	TCC	473,00	1,31	
**CAC**	542,00	1,50	GTG	515,00	1,42	GTG*			CAC*			IIc
**CGC**	44,00	0,12	GCG	46,00	0,13	GCG*			CGC*			
**CTC**	512,00	1,42	GAG	635,00	1,76	GAG*			CTC*			
**CCC**	450,00	1,24	GGG	409,00	1,13	GGG*			CCC*			

Quadruplet		f1	f2	fu
CGT	CGT(D)	0,16	1,58	1,74
	ACG(RC(D))	0,21	1,27	1,48
	GCA(C(D))	1,58	0,16	1,74
	TGC(R(D))	1,27	0,21	1,48
GTC	GTC(D)	0,79	2,20	2,99
	GAC(RC(D))	1,16	1,90	3,06
	CAG(C(D))	2,20	0,79	2,99
	CTG(R(D))	1,90	1,16	3,06
GCT	GCT(D)	1,24	0,17	1,40
	AGC(RC(D))	1,39	0,13	1,52
	CGA(C(D))	0,17	1,24	1,40
	TCG(R(D))	0,13	1,39	1,52
GCC	GCC(D)	0,94	0,16	1,09
	GGC(RC(D))	1,09	0,14	1,24
	CGG(C(D))	0,16	0,94	1,09
	CCG(R(D))	0,14	1,09	1,24
CCA	CCA(D)	1,91	0,98	2,89
	TGG(RC(D))	1,85	1,37	3,22
	GGT(C(D))	0,98	1,91	2,89
	ACC(R(D))	1,37	1,85	3,22
CCT	CCT(D)	1,56	1,77	3,33
	AGG(RC(D))	1,84	1,31	3,15
	GGA(C(D))	1,77	1,56	3,33
	TCC(R(D))	1,31	1,84	3,15
CAC	CAC(D)	0,75	0,71	1,46
	GTG(RC(D))	0,71	0,75	1,46
	GTG(C(D))	0,71	0,75	1,46
	CAC(R(D))	0,75	0,71	1,46
CGC	CGC(D)	0,06	0,06	0,12
	GCG(RC(D))	0,06	0,06	0,12
	GCG(C(D))	0,06	0,06	0,12
	CGC(R(D))	0,06	0,06	0,12
CTC	CTC(D)	0,71	0,88	1,59
	GAG(RC(D))	0,88	0,71	1,59
	GAG(C(D))	0,88	0,71	1,59
	CTC(R(D))	0,71	0,88	1,59
CCC	CCC(D)	0,62	0,57	1,19
	GGG(RC(D))	0,57	0,62	1,19
	GGG(C(D))	0,57	0,62	1,19
	CCC(R(D))	0,62	0,57	1,19

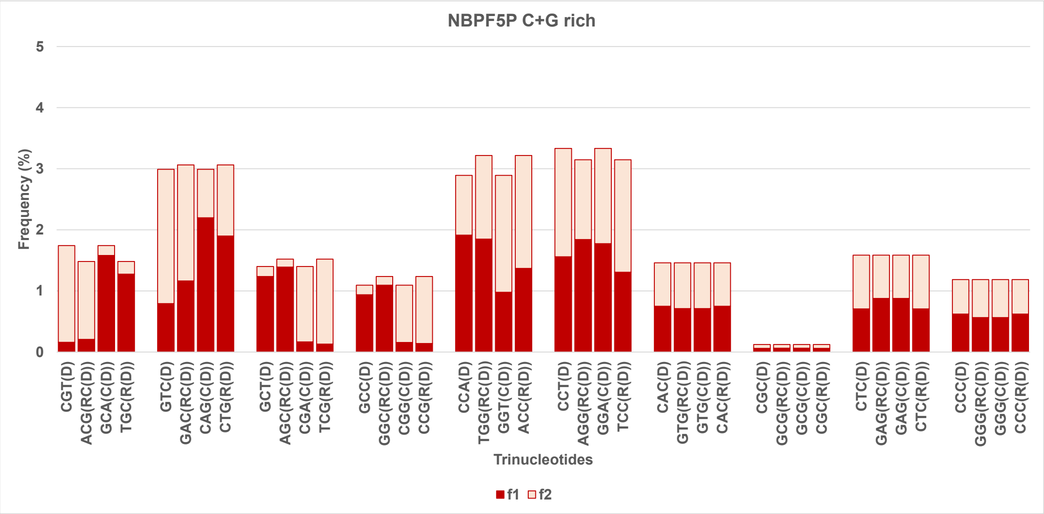

NBPF5P exons

C+G rich

D	f	%	RC	f	%	C	f	%	R	f	%	
**CGT**	3,00	0,19	ACG	7,00	0,44	GCA	27,00	1,68	TGC	22,00	1,37	IIa
**GTC**	19,00	1,18	GAC	21,00	1,31	CAG	45,00	2,81	CTG	42,00	2,62	
**GCT**	36,00	2,24	AGC	31,00	1,93	CGA	9,00	0,56	TCG	7,00	0,44	
**GCC**	18,00	1,12	GGC	26,00	1,62	CGG	7,00	0,44	CCG	7,00	0,44	IIb
**CCA**	30,00	1,87	TGG	25,00	1,56	GGT	13,00	0,81	ACC	27,00	1,68	
**CCT**	40,00	2,49	AGG	41,00	2,56	GGA	34,00	2,12	TCC	32,00	2,00	
**CAC**	24,00	1,50	GTG	20,00	1,25	GTG*			CAC*			IIc
**CGC**	6,00	0,37	GCG	4,00	0,25	GCG*			CGC*			
**CTC**	47,00	2,93	GAG	45,00	2,81	GAG*			CTC*			
**CCC**	37,00	2,31	GGG	15,00	0,94	GGG*			CCC*			

Quadruplet		f1	f2	fu
CGT	CGT(D)	0,19	1,68	1,87
	ACG(RC(D))	0,44	1,37	1,81
	GCA(C(D))	1,68	0,19	1,87
	TGC(R(D))	1,37	0,44	1,81
GTC	GTC(D)	1,18	2,81	3,99
	GAC(RC(D))	1,31	2,62	3,93
	CAG(C(D))	2,81	1,18	3,99
	CTG(R(D))	2,62	1,31	3,93
GCT	GCT(D)	2,24	0,56	2,81
	AGC(RC(D))	1,93	0,44	2,37
	CGA(C(D))	0,56	2,24	2,81
	TCG(R(D))	0,44	1,93	2,37
GCC	GCC(D)	1,12	0,44	1,56
	GGC(RC(D))	1,62	0,44	2,06
	CGG(C(D))	0,44	1,12	1,56
	CCG(R(D))	0,44	1,62	2,06
CCA	CCA(D)	1,87	0,81	2,68
	TGG(RC(D))	1,56	1,68	3,24
	GGT(C(D))	0,81	1,87	2,68
	ACC(R(D))	1,68	1,56	3,24
CCT	CCT(D)	2,49	2,12	4,61
	AGG(RC(D))	2,56	2,00	4,55
	GGA(C(D))	2,12	2,49	4,61
	TCC(R(D))	2,00	2,56	4,55
CAC	CAC(D)	0,75	0,62	1,37
	GTG(RC(D))	0,62	0,75	1,37
	GTG(C(D))	0,62	0,75	1,37
	CAC(R(D))	0,75	0,62	1,37
CGC	CGC(D)	0,19	0,12	0,31
	GCG(RC(D))	0,12	0,19	0,31
	GCG(C(D))	0,12	0,19	0,31
	CGC(R(D))	0,19	0,12	0,31
CTC	CTC(D)	1,47	1,40	2,87
	GAG(RC(D))	1,40	1,47	2,87
	GAG(C(D))	1,40	1,47	2,87
	CTC(R(D))	1,47	1,40	2,87
CCC	CCC(D)	1,15	0,47	1,62
	GGG(RC(D))	0,47	1,15	1,62
	GGG(C(D))	0,47	1,15	1,62
	CCC(R(D))	1,15	0,47	1,62

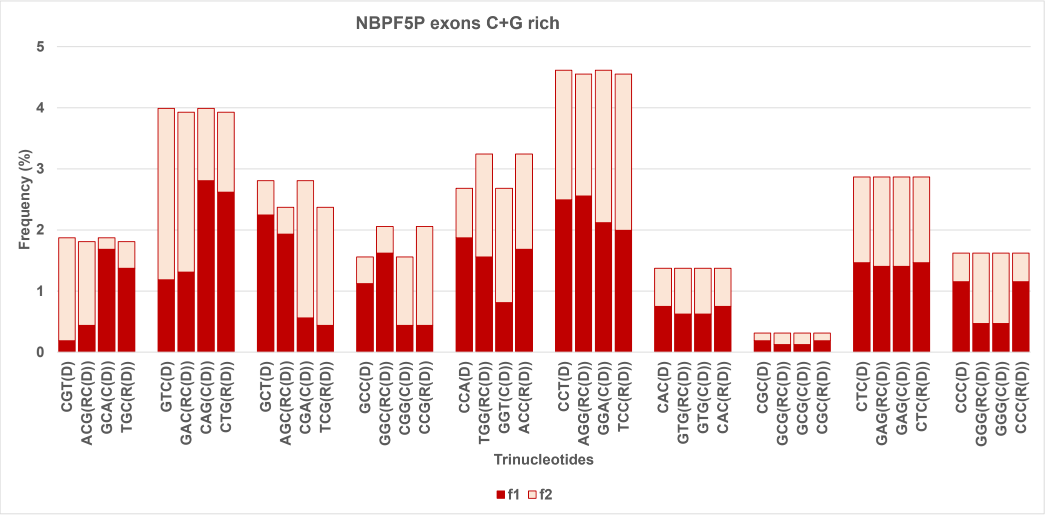

NBPF5P introns

C+G rich

D	f	%	RC	f	%	C	f	%	R	f	%	
**CGT**	51,00	0,15	ACG	66,00	0,20	GCA	530,00	1,59	TGC	423,00	1,27	IIa
**GTC**	260,00	0,78	GAC	383,00	1,15	CAG	727,00	2,18	CTG	621,00	1,86	
**GCT**	397,00	1,19	AGC	456,00	1,37	CGA	49,00	0,15	TCG	38,00	0,11	
**GCC**	311,00	0,93	GGC	363,00	1,09	CGG	49,00	0,15	CCG	41,00	0,12	IIb
**CCA**	645,00	1,93	TGG	625,00	1,87	GGT	326,00	0,98	ACC	453,00	1,36	
**CCT**	507,00	1,52	AGG	605,00	1,81	GGA	590,00	1,77	TCC	429,00	1,29	
**CAC**	504,00	1,51	GTG	477,00	1,43	GTG*			CAC*			IIc
**CGC**	38,00	0,11	GCG	42,00	0,13	GCG*			CGC*			
**CTC**	453,00	1,36	GAG	572,00	1,71	GAG*			CTC*			
**CCC**	402,00	1,20	GGG	377,00	1,13	GGG*			CCC*			

Quadruplet		f1	f2	fu
CGT	CGT(D)	0,15	1,59	1,74
	ACG(RC(D))	0,20	1,27	1,47
	GCA(C(D))	1,59	0,15	1,74
	TGC(R(D))	1,27	0,20	1,47
GTC	GTC(D)	0,78	2,18	2,96
	GAC(RC(D))	1,15	1,86	3,01
	CAG(C(D))	2,18	0,78	2,96
	CTG(R(D))	1,86	1,15	3,01
GCT	GCT(D)	1,19	0,15	1,34
	AGC(RC(D))	1,37	0,11	1,48
	CGA(C(D))	0,15	1,19	1,34
	TCG(R(D))	0,11	1,37	1,48
GCC	GCC(D)	0,93	0,15	1,08
	GGC(RC(D))	1,09	0,12	1,21
	CGG(C(D))	0,15	0,93	1,08
	CCG(R(D))	0,12	1,09	1,21
CCA	CCA(D)	1,93	0,98	2,91
	TGG(RC(D))	1,87	1,36	3,23
	GGT(C(D))	0,98	1,93	2,91
	ACC(R(D))	1,36	1,87	3,23
CCT	CCT(D)	1,52	1,77	3,29
	AGG(RC(D))	1,81	1,29	3,10
	GGA(C(D))	1,77	1,52	3,29
	TCC(R(D))	1,29	1,81	3,10
CAC	CAC(D)	0,76	0,71	1,47
	GTG(RC(D))	0,71	0,76	1,47
	GTG(C(D))	0,71	0,76	1,47
	CAC(R(D))	0,76	0,71	1,47
CGC	CGC(D)	0,06	0,06	0,12
	GCG(RC(D))	0,06	0,06	0,12
	GCG(C(D))	0,06	0,06	0,12
	CGC(R(D))	0,06	0,06	0,12
CTC	CTC(D)	0,68	0,86	1,54
	GAG(RC(D))	0,86	0,68	1,54
	GAG(C(D))	0,86	0,68	1,54
	CTC(R(D))	0,68	0,86	1,54
CCC	CCC(D)	0,60	0,56	1,17
	GGG(RC(D))	0,56	0,60	1,17
	GGG(C(D))	0,56	0,60	1,17
	CCC(R(D))	0,60	0,56	1,17

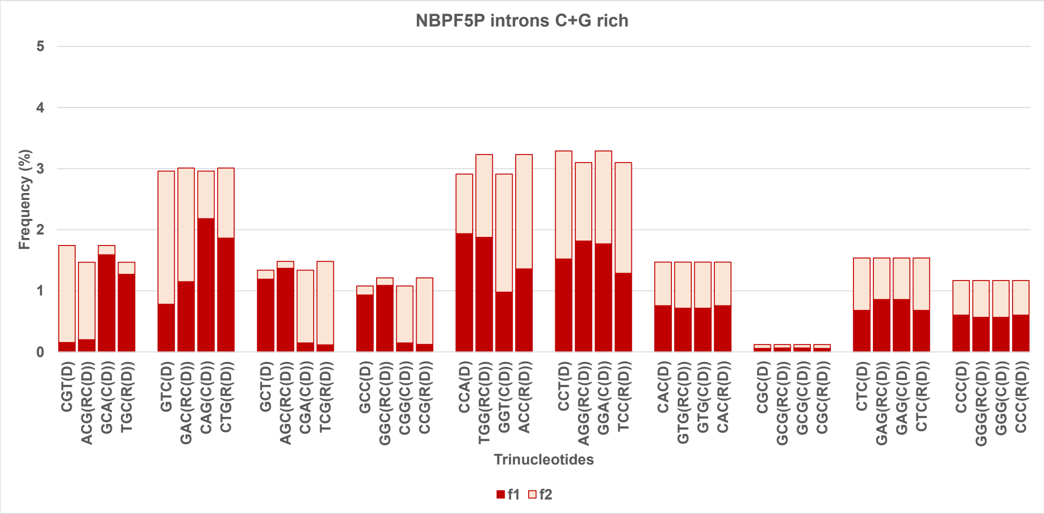

NBPF6 sequence

						A-T rich						
**D**	**f**	**%**	**RC**	**f**	**%**	**C**	**f**	**%**	**R**	**f**	**%**	
**ATG**	437,00	1,99	CAT	422,00	1,93	TAC	193,00	0,88	GTA	195,00	0,89	Ia
**TGA**	499,00	2,28	TCA	470,00	2,14	ACT	360,00	1,64	AGT	326,00	1,49	
**TAG**	229,00	1,04	CTA	243,00	1,11	ATC	299,00	1,36	GAT	310,00	1,41	
**TAA**	279,00	1,27	TTA	307,00	1,40	ATT	424,00	1,93	AAT	384,00	1,75	Ib
**AAC**	299,00	1,36	GTT	290,00	1,32	TTG	409,00	1,87	CAA	343,00	1,56	
**AAG**	402,00	1,83	CTT	480,00	2,19	TTC	478,00	2,18	GAA	463,00	2,11	
**ACA**	414,00	1,89	TGT	558,00	2,55	TGT*			ACA*			Ic
**ATA**	264,00	1,20	TAT	308,00	1,41	TAT*			ATA*			
**AGA**	498,00	2,27	TCT	624,00	2,85	TCT*			AGA*			
**AAA**	590,00	2,69	TTT	618,00	2,82	TTT*			AAA*			

Quadruplet		f1	f2	fu
ATG	ATG(D)	1,99	0,88	2,87
	CAT(RC(D))	1,93	0,89	2,81
	TAC(C(D))	0,88	1,99	2,87
	GTA(R(D))	0,89	1,93	2,81
TGA	TGA(D)	2,28	1,64	3,92
	TCA(RC(D))	2,14	1,49	3,63
	ACT(C(D))	1,64	2,28	3,92
	AGT(R(D))	1,49	2,14	3,63
TAG	TAG(D)	1,04	1,36	2,41
	CTA(RC(D))	1,11	1,41	2,52
	ATC(C(D))	1,36	1,04	2,41
	GAT(R(D))	1,41	1,11	2,52
TAA	TAA(D)	1,27	1,93	3,21
	TTA(RC(D))	1,40	1,75	3,15
	ATT(C(D))	1,93	1,27	3,21
	AAT(R(D))	1,75	1,40	3,15
AAC	AAC(D)	1,36	1,87	3,23
	GTT(RC(D))	1,32	1,56	2,89
	TTG(C(D))	1,87	1,36	3,23
	CAA(R(D))	1,56	1,32	2,89
AAG	AAG(D)	1,83	2,18	4,01
	CTT(RC(D))	2,19	2,11	4,30
	TTC(C(D))	2,18	1,83	4,01
	GAA(R(D))	2,11	2,19	4,30
ACA	ACA(D)	0,94	1,27	2,22
	TGT(RC(D))	1,27	0,94	2,22
	TGT(C(D))	1,27	0,94	2,22
	ACA(R(D))	0,94	1,27	2,22
ATA	ATA(D)	0,60	0,70	1,30
	TAT(RC(D))	0,70	0,60	1,30
	TAT(C(D))	0,70	0,60	1,30
	ATA(R(D))	0,60	0,60	1,20
AGA	AGA(D)	1,14	1,42	2,56
	TCT(RC(D))	1,42	1,14	2,56
	TCT(C(D))	1,42	1,14	2,56
	AGA(R(D))	1,14	1,42	2,56
AAA	AAA(D)	1,35	1,41	2,76
	TTT(RC(D))	1,41	1,35	2,76
	TTT(C(D))	1,41	1,35	2,76
	AAA(R(D))	1,35	1,41	2,76

Composition of DNA sequence in NBPF6

Base	No.of bases	%
A	5798	26,45%
C	5087	23,21%
G	4797	21,88%
T	6239	28,46%
N	0	0,00%
Sum	21921	100,00%

% of coding bases from NBPF6 (including N bases)

82,73%

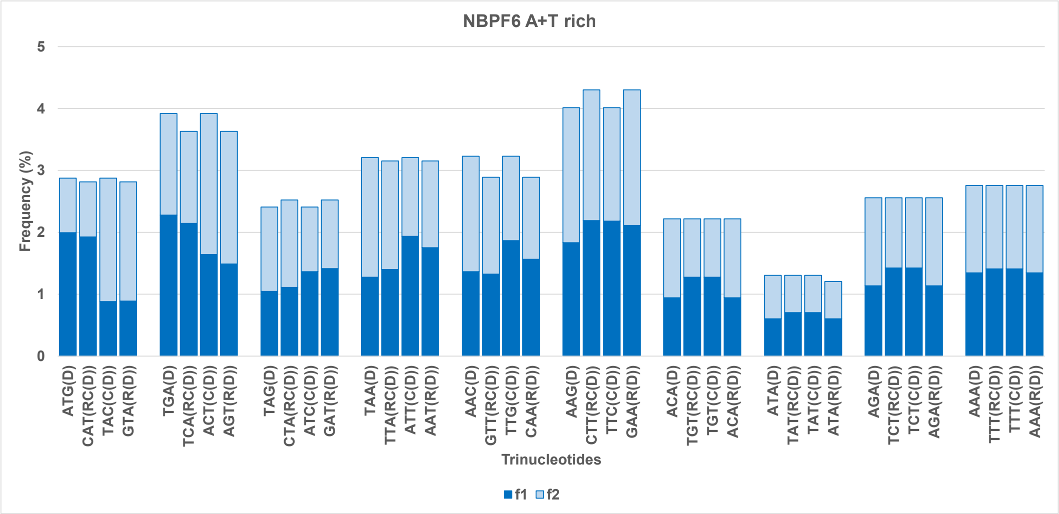

NBPF6 exons

A-T rich

D	f	%	RC	f	%	C	f	%	R	f	%	
**ATG**	38,00	1,71	CAT	24,00	1,08	TAC	21,00	0,95	GTA	9,00	0,41	Ia
**TGA**	61,00	2,75	TCA	55,00	2,48	ACT	42,00	1,89	AGT	33,00	1,49	
**TAG**	10,00	0,45	CTA	13,00	0,59	ATC	23,00	1,04	GAT	35,00	1,58	
**TAA**	10,00	0,45	TTA	14,00	0,63	ATT	17,00	0,77	AAT	24,00	1,08	Ib
**AAC**	30,00	1,35	GTT	25,00	1,13	TTG	35,00	1,58	CAA	50,00	2,25	
**AAG**	67,00	3,02	CTT	37,00	1,67	TTC	30,00	1,35	GAA	61,00	2,75	
**ACA**	34,00	1,53	TGT	29,00	1,31	TGT*			ACA*			Ic
**ATA**	11,00	0,50	TAT	6,00	0,27	TAT*			ATA*			
**AGA**	66,00	2,97	TCT	35,00	1,58	TCT*			AGA*			
**AAA**	46,00	2,07	TTT	19,00	0,86	TTT*			AAA*			

Quadruplet		f1	f2	fu
ATG	ATG(D)	1,71	0,95	2,66
	CAT(RC(D))	1,08	0,41	1,49
	TAC(C(D))	0,95	1,71	2,66
	GTA(R(D))	0,41	1,08	1,49
TGA	TGA(D)	2,75	1,89	4,64
	TCA(RC(D))	2,48	1,49	3,96
	ACT(C(D))	1,89	2,75	4,64
	AGT(R(D))	1,49	2,48	3,96
TAG	TAG(D)	0,45	1,04	1,49
	CTA(RC(D))	0,59	1,58	2,16
	ATC(C(D))	1,04	0,45	1,49
	GAT(R(D))	1,58	0,59	2,16
TAA	TAA(D)	0,45	0,77	1,22
	TTA(RC(D))	0,63	1,08	1,71
	ATT(C(D))	0,77	0,45	1,22
	AAT(R(D))	1,08	0,63	1,71
AAC	AAC(D)	1,35	1,58	2,93
	GTT(RC(D))	1,13	2,25	3,38
	TTG(C(D))	1,58	1,35	2,93
	CAA(R(D))	2,25	1,13	3,38
AAG	AAG(D)	3,02	1,35	4,37
	CTT(RC(D))	1,67	2,75	4,41
	TTC(C(D))	1,35	3,02	4,37
	GAA(R(D))	2,75	1,67	4,41
ACA	ACA(D)	0,77	0,65	1,42
	TGT(RC(D))	0,65	0,77	1,42
	TGT(C(D))	0,65	0,77	1,42
	ACA(R(D))	0,77	0,65	1,42
ATA	ATA(D)	0,25	0,14	0,38
	TAT(RC(D))	0,14	0,25	0,38
	TAT(C(D))	0,14	0,25	0,38
	ATA(R(D))	0,25	0,25	0,50
AGA	AGA(D)	1,49	0,79	2,27
	TCT(RC(D))	0,79	1,49	2,27
	TCT(C(D))	0,79	1,49	2,27
	AGA(R(D))	1,49	0,79	2,27
AAA	AAA(D)	1,04	0,43	1,46
	TTT(RC(D))	0,43	1,04	1,46
	TTT(C(D))	0,43	1,04	1,46
	AAA(R(D))	1,04	0,43	1,46

Composition of DNA sequence in NBPF6 exons

Base	No.of bases	%
A	603	27,14%
C	594	26,73%
G	564	25,38%
T	461	20,75%
N	0	0,00%
Sum	2222	100,00%

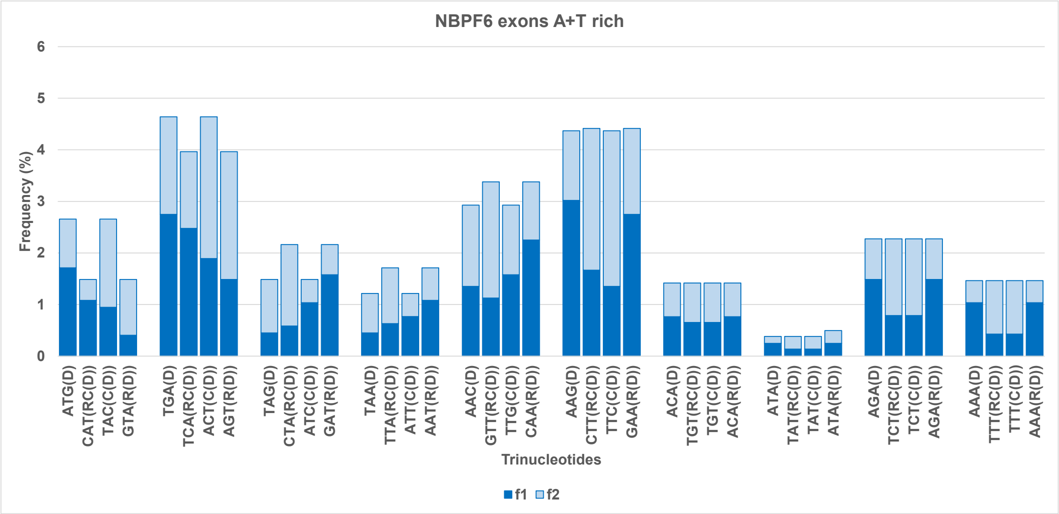

NBPF6 introns

A-T rich

D	f	%	RC	f	%	C	f	%	R	f	%	
**ATG**	363,00	2,00	CAT	365,00	2,01	TAC	160,00	0,88	GTA	172,00	0,95	Ia
**TGA**	394,00	2,17	TCA	379,00	2,09	ACT	292,00	1,61	AGT	265,00	1,46	
**TAG**	202,00	1,11	CTA	211,00	1,16	ATC	255,00	1,41	GAT	253,00	1,40	
**TAA**	243,00	1,34	TTA	271,00	1,49	ATT	376,00	2,07	AAT	327,00	1,80	Ib
**AAC**	241,00	1,33	GTT	248,00	1,37	TTG	345,00	1,90	CAA	261,00	1,44	
**AAG**	298,00	1,64	CTT	413,00	2,28	TTC	421,00	2,32	GAA	362,00	2,00	
**ACA**	339,00	1,87	TGT	500,00	2,76	TGT*			ACA*			Ic
**ATA**	229,00	1,26	TAT	278,00	1,53	TAT*			ATA*			
**AGA**	381,00	2,10	TCT	564,00	3,11	TCT*			AGA*			
**AAA**	475,00	2,62	TTT	557,00	3,07	TTT*			AAA*			

Quadruplet		f1	f2	fu
ATG	ATG(D)	2,00	0,88	2,88
	CAT(RC(D))	2,01	0,95	2,96
	TAC(C(D))	0,88	2,00	2,88
	GTA(R(D))	0,95	2,01	2,96
TGA	TGA(D)	2,17	1,61	3,78
	TCA(RC(D))	2,09	1,46	3,55
	ACT(C(D))	1,61	2,17	3,78
	AGT(R(D))	1,46	2,09	3,55
TAG	TAG(D)	1,11	1,41	2,52
	CTA(RC(D))	1,16	1,40	2,56
	ATC(C(D))	1,41	1,11	2,52
	GAT(R(D))	1,40	1,16	2,56
TAA	TAA(D)	1,34	2,07	3,41
	TTA(RC(D))	1,49	1,80	3,30
	ATT(C(D))	2,07	1,34	3,41
	AAT(R(D))	1,80	1,49	3,30
AAC	AAC(D)	1,33	1,90	3,23
	GTT(RC(D))	1,37	1,44	2,81
	TTG(C(D))	1,90	1,33	3,23
	CAA(R(D))	1,44	1,37	2,81
AAG	AAG(D)	1,64	2,32	3,97
	CTT(RC(D))	2,28	2,00	4,27
	TTC(C(D))	2,32	1,64	3,97
	GAA(R(D))	2,00	2,28	4,27
ACA	ACA(D)	0,93	1,38	2,31
	TGT(RC(D))	1,38	0,93	2,31
	TGT(C(D))	1,38	0,93	2,31
	ACA(R(D))	0,93	1,38	2,31
ATA	ATA(D)	0,63	0,77	1,40
	TAT(RC(D))	0,77	0,63	1,40
	TAT(C(D))	0,77	0,63	1,40
	ATA(R(D))	0,63	0,63	1,26
AGA	AGA(D)	1,05	1,56	2,61
	TCT(RC(D))	1,56	1,05	2,61
	TCT(C(D))	1,56	1,05	2,61
	AGA(R(D))	1,05	1,56	2,61
AAA	AAA(D)	1,31	1,54	2,85
	TTT(RC(D))	1,54	1,31	2,85
	TTT(C(D))	1,54	1,31	2,85
	AAA(R(D))	1,31	1,54	2,85

Composition of DNA sequence NBPF6 introns

Base	No.of bases	%
A	4694	25,88%
C	4167	22,98%
G	3899	21,50%
T	5375	29,64%
N	0	0,00%
Sum	18135	100,00%

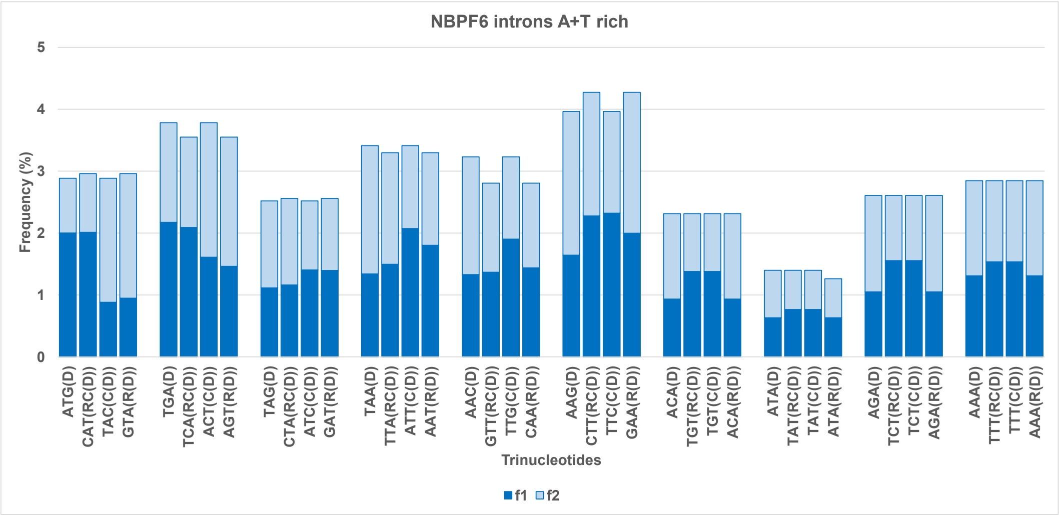

NBPF6 sequence

						C+G rich						
**D**	**f**	**%**	**RC**	**f**	**%**	**C**	**f**	**%**	**R**	**f**	**%**	
**CGT**	63,00	0,29	ACG	58,00	0,26	GCA	341,00	1,56	TGC	361,00	1,65	IIa
**GTC**	302,00	1,38	GAC	261,00	1,19	CAG	520,00	2,37	CTG	583,00	2,66	
**GCT**	304,00	1,39	AGC	314,00	1,43	CGA	48,00	0,22	TCG	49,00	0,22	
**GCC**	274,00	1,25	GGC	252,00	1,15	CGG	56,00	0,26	CCG	52,00	0,24	IIb
**CCA**	437,00	1,99	TGG	417,00	1,90	GGT	245,00	1,12	ACC	298,00	1,36	
**CCT**	522,00	2,38	AGG	431,00	1,97	GGA	407,00	1,86	TCC	440,00	2,01	
**CAC**	377,00	1,72	GTG	406,00	1,85	GTG*			CAC*			IIc
**CGC**	35,00	0,16	GCG	43,00	0,20	GCG*			CGC*			
**CTC**	504,00	2,30	GAG	418,00	1,91	GAG*			CTC*			
**CCC**	400,00	1,82	GGG	286,00	1,30	GGG*			CCC*			

Quadruplet		f1	f2	fu
CGT	CGT(D)	0,29	1,56	1,84
	ACG(RC(D))	0,26	1,65	1,91
	GCA(C(D))	1,56	0,29	1,84
	TGC(R(D))	1,65	0,26	1,91
GTC	GTC(D)	1,38	2,37	3,75
	GAC(RC(D))	1,19	2,66	3,85
	CAG(C(D))	2,37	1,38	3,75
	CTG(R(D))	2,66	1,19	3,85
GCT	GCT(D)	1,39	0,22	1,61
	AGC(RC(D))	1,43	0,22	1,66
	CGA(C(D))	0,22	1,39	1,61
	TCG(R(D))	0,22	1,43	1,66
GCC	GCC(D)	1,25	0,26	1,51
	GGC(RC(D))	1,15	0,24	1,39
	CGG(C(D))	0,26	1,25	1,51
	CCG(R(D))	0,24	1,15	1,39
CCA	CCA(D)	1,99	1,12	3,11
	TGG(RC(D))	1,90	1,36	3,26
	GGT(C(D))	1,12	1,99	3,11
	ACC(R(D))	1,36	1,90	3,26
CCT	CCT(D)	2,38	1,86	4,24
	AGG(RC(D))	1,97	2,01	3,97
	GGA(C(D))	1,86	2,38	4,24
	TCC(R(D))	2,01	1,97	3,97
CAC	CAC(D)	0,86	0,93	1,79
	GTG(RC(D))	0,93	0,86	1,79
	GTG(C(D))	0,93	0,86	1,79
	CAC(R(D))	0,86	0,93	1,79
CGC	CGC(D)	0,08	0,10	0,18
	GCG(RC(D))	0,10	0,08	0,18
	GCG(C(D))	0,10	0,08	0,18
	CGC(R(D))	0,08	0,10	0,18
CTC	CTC(D)	1,15	0,95	2,10
	GAG(RC(D))	0,95	1,15	2,10
	GAG(C(D))	0,95	1,15	2,10
	CTC(R(D))	1,15	0,95	2,10
CCC	CCC(D)	0,91	0,65	1,56
	GGG(RC(D))	0,65	0,91	1,56
	GGG(C(D))	0,65	0,91	1,56
	CCC(R(D))	0,91	0,65	1,56

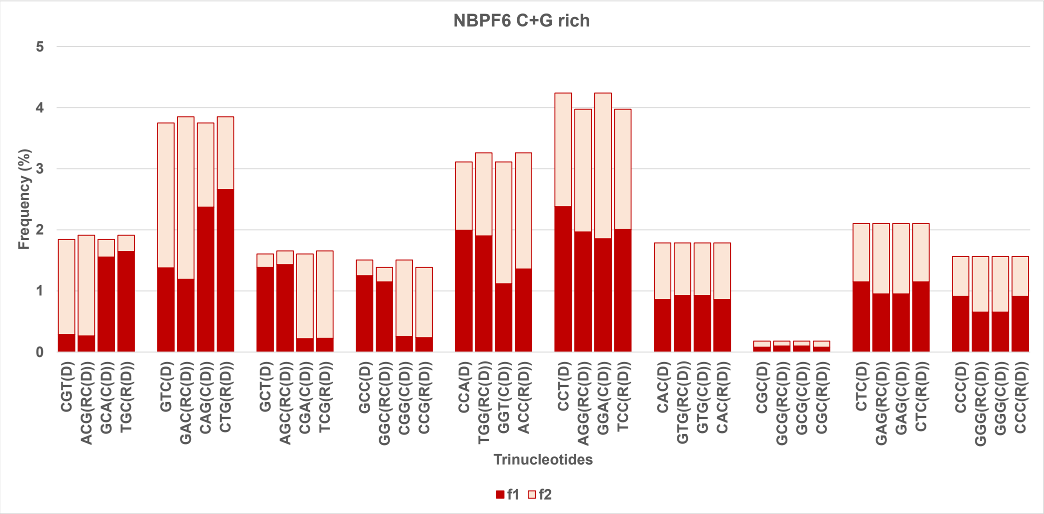

NBPF6 exons

C+G rich

D	f	%	RC	f	%	C	f	%	R	f	%	
**CGT**	8,00	0,36	ACG	8,00	0,36	GCA	43,00	1,94	TGC	37,00	1,67	IIa
**GTC**	27,00	1,22	GAC	40,00	1,80	CAG	73,00	3,29	CTG	70,00	3,15	
**GCT**	51,00	2,30	AGC	54,00	2,43	CGA	12,00	0,54	TCG	8,00	0,36	
**GCC**	32,00	1,44	GGC	35,00	1,58	CGG	11,00	0,50	CCG	15,00	0,68	IIb
**CCA**	56,00	2,52	TGG	44,00	1,98	GGT	19,00	0,86	ACC	48,00	2,16	
**CCT**	56,00	2,52	AGG	61,00	2,75	GGA	62,00	2,79	TCC	47,00	2,12	
**CAC**	41,00	1,85	GTG	28,00	1,26	GTG*			CAC*			IIc
**CGC**	8,00	0,36	GCG	8,00	0,36	GCG*			CGC*			
**CTC**	65,00	2,93	GAG	64,00	2,88	GAG*			CTC*			
**CCC**	55,00	2,48	GGG	24,00	1,08	GGG*			CCC*			

Quadruplet		f1	f2	fu
CGT	CGT(D)	0,36	1,94	2,30
	ACG(RC(D))	0,36	1,67	2,03
	GCA(C(D))	1,94	0,36	2,30
	TGC(R(D))	1,67	0,36	2,03
GTC	GTC(D)	1,22	3,29	4,50
	GAC(RC(D))	1,80	3,15	4,95
	CAG(C(D))	3,29	1,22	4,50
	CTG(R(D))	3,15	1,80	4,95
GCT	GCT(D)	2,30	0,54	2,84
	AGC(RC(D))	2,43	0,36	2,79
	CGA(C(D))	0,54	2,30	2,84
	TCG(R(D))	0,36	2,43	2,79
GCC	GCC(D)	1,44	0,50	1,94
	GGC(RC(D))	1,58	0,68	2,25
	CGG(C(D))	0,50	1,44	1,94
	CCG(R(D))	0,68	1,58	2,25
CCA	CCA(D)	2,52	0,86	3,38
	TGG(RC(D))	1,98	2,16	4,14
	GGT(C(D))	0,86	2,52	3,38
	ACC(R(D))	2,16	1,98	4,14
CCT	CCT(D)	2,52	2,79	5,32
	AGG(RC(D))	2,75	2,12	4,86
	GGA(C(D))	2,79	2,52	5,32
	TCC(R(D))	2,12	2,75	4,86
CAC	CAC(D)	0,92	0,63	1,55
	GTG(RC(D))	0,63	0,92	1,55
	GTG(C(D))	0,63	0,92	1,55
	CAC(R(D))	0,92	0,63	1,55
CGC	CGC(D)	0,18	0,18	0,36
	GCG(RC(D))	0,18	0,18	0,36
	GCG(C(D))	0,18	0,18	0,36
	CGC(R(D))	0,18	0,18	0,36
CTC	CTC(D)	1,46	1,44	2,91
	GAG(RC(D))	1,44	1,46	2,91
	GAG(C(D))	1,44	1,46	2,91
	CTC(R(D))	1,46	1,44	2,91
CCC	CCC(D)	1,24	0,54	1,78
	GGG(RC(D))	0,54	1,24	1,78
	GGG(C(D))	0,54	1,24	1,78
	CCC(R(D))	1,24	0,54	1,78

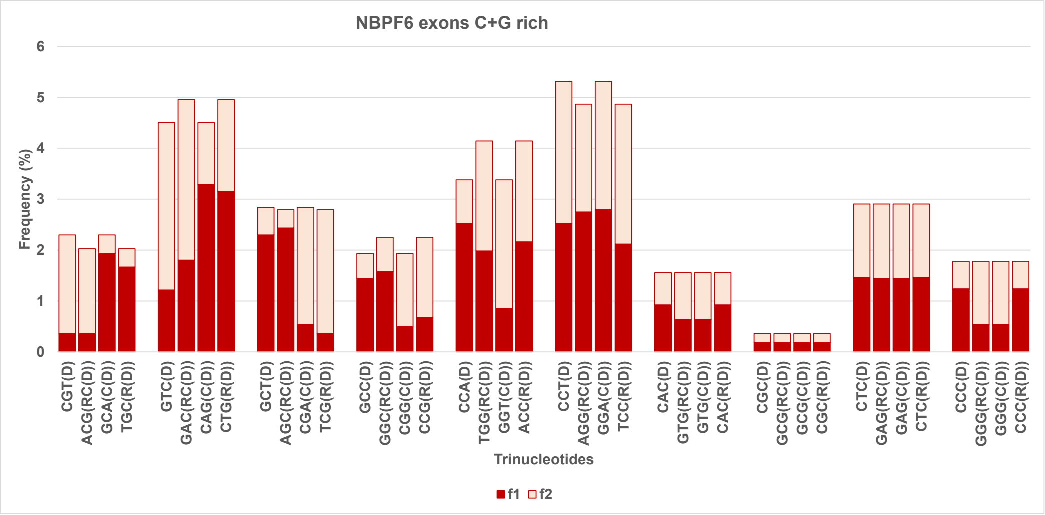

NBPF6 introns

C+G rich

D	f	%	RC	f	%	C	f	%	R	f	%	
**CGT**	50,00	0,28	ACG	47,00	0,26	GCA	272,00	1,50	TGC	299,00	1,65	IIa
**GTC**	251,00	1,38	GAC	195,00	1,08	CAG	403,00	2,22	CTG	482,00	2,66	
**GCT**	236,00	1,30	AGC	230,00	1,27	CGA	34,00	0,19	TCG	38,00	0,21	
**GCC**	222,00	1,22	GGC	208,00	1,15	CGG	41,00	0,23	CCG	33,00	0,18	IIb
**CCA**	351,00	1,94	TGG	352,00	1,94	GGT	210,00	1,16	ACC	230,00	1,27	
**CCT**	440,00	2,43	AGG	345,00	1,90	GGA	320,00	1,76	TCC	372,00	2,05	
**CAC**	312,00	1,72	GTG	355,00	1,96	GTG*			CAC*			IIc
**CGC**	26,00	0,14	GCG	33,00	0,18	GCG*			CGC*			
**CTC**	426,00	2,35	GAG	319,00	1,76	GAG*			CTC*			
**CCC**	319,00	1,76	GGG	242,00	1,33	GGG*			CCC*			

Quadruplet		f1	f2	fu
CGT	CGT(D)	0,28	1,50	1,78
	ACG(RC(D))	0,26	1,65	1,91
	GCA(C(D))	1,50	0,28	1,78
	TGC(R(D))	1,65	0,26	1,91
GTC	GTC(D)	1,38	2,22	3,61
	GAC(RC(D))	1,08	2,66	3,73
	CAG(C(D))	2,22	1,38	3,61
	CTG(R(D))	2,66	1,08	3,73
GCT	GCT(D)	1,30	0,19	1,49
	AGC(RC(D))	1,27	0,21	1,48
	CGA(C(D))	0,19	1,30	1,49
	TCG(R(D))	0,21	1,27	1,48
GCC	GCC(D)	1,22	0,23	1,45
	GGC(RC(D))	1,15	0,18	1,33
	CGG(C(D))	0,23	1,22	1,45
	CCG(R(D))	0,18	1,15	1,33
CCA	CCA(D)	1,94	1,16	3,09
	TGG(RC(D))	1,94	1,27	3,21
	GGT(C(D))	1,16	1,94	3,09
	ACC(R(D))	1,27	1,94	3,21
CCT	CCT(D)	2,43	1,76	4,19
	AGG(RC(D))	1,90	2,05	3,95
	GGA(C(D))	1,76	2,43	4,19
	TCC(R(D))	2,05	1,90	3,95
CAC	CAC(D)	0,86	0,98	1,84
	GTG(RC(D))	0,98	0,86	1,84
	GTG(C(D))	0,98	0,86	1,84
	CAC(R(D))	0,86	0,98	1,84
CGC	CGC(D)	0,07	0,09	0,16
	GCG(RC(D))	0,09	0,07	0,16
	GCG(C(D))	0,09	0,07	0,16
	CGC(R(D))	0,07	0,09	0,16
CTC	CTC(D)	1,17	0,88	2,05
	GAG(RC(D))	0,88	1,17	2,05
	GAG(C(D))	0,88	1,17	2,05
	CTC(R(D))	1,17	0,88	2,05
CCC	CCC(D)	0,88	0,67	1,55
	GGG(RC(D))	0,67	0,88	1,55
	GGG(C(D))	0,67	0,88	1,55
	CCC(R(D))	0,88	0,67	1,55

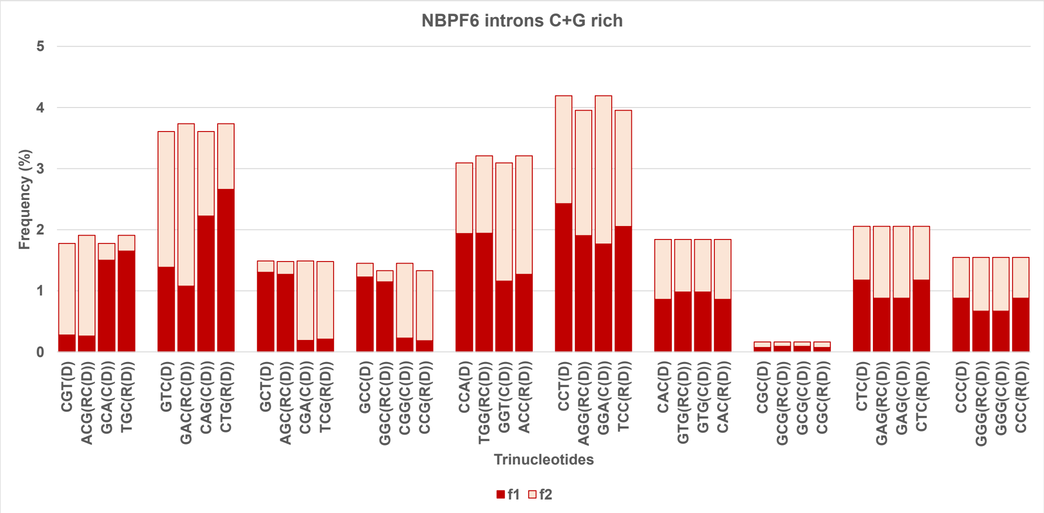

NBPF7 sequence

A-T rich												
**D**	**f**	**%**	**RC**	**f**	**%**	**C**	**f**	**%**	**R**	**f**	**%**	
**ATG**	201,00	1,85	CAT	184,00	1,70	TAC	77,00	0,71	GTA	111,00	1,02	Ia
**TGA**	235,00	2,17	TCA	249,00	2,30	ACT	152,00	1,40	AGT	175,00	1,61	
**TAG**	120,00	1,11	CTA	99,00	0,91	ATC	164,00	1,51	GAT	159,00	1,47	
**TAA**	127,00	1,17	TTA	127,00	1,17	ATT	180,00	1,66	AAT	189,00	1,74	Ib
**AAC**	135,00	1,25	GTT	153,00	1,41	TTG	202,00	1,86	CAA	200,00	1,84	
**AAG**	236,00	2,18	CTT	230,00	2,12	TTC	234,00	2,16	GAA	233,00	2,15	
**ACA**	215,00	1,98	TGT	258,00	2,38	TGT*			ACA*			Ic
**ATA**	116,00	1,07	TAT	129,00	1,19	TAT*			ATA*			
**AGA**	283,00	2,61	TCT	308,00	2,84	TCT*			AGA*			
**AAA**	297,00	2,74	TTT	345,00	3,18	TTT*			AAA*			

Quadruplet		f1	f2	fu
	ATG(D)	1,85	0,71	2,56
ATG	CAT(RC(D))	1,70	1,02	2,72
	TAC(C(D))	0,71	1,85	2,56
	GTA(R(D))	1,02	1,70	2,72
	TGA(D)	2,17	1,40	3,57
TGA	TCA(RC(D))	2,30	1,61	3,91
	ACT(C(D))	1,40	2,17	3,57
	AGT(R(D))	1,61	2,30	3,91
	TAG(D)	1,11	1,51	2,62
TAG	CTA(RC(D))	0,91	1,47	2,38
	ATC(C(D))	1,51	1,11	2,62
	GAT(R(D))	1,47	0,91	2,38
	TAA(D)	1,17	1,66	2,83
TAA	TTA(RC(D))	1,17	1,74	2,91
	ATT(C(D))	1,66	1,17	2,83
	AAT(R(D))	1,74	1,17	2,91
	AAC(D)	1,25	1,86	3,11
AAC	GTT(RC(D))	1,41	1,84	3,26
	TTG(C(D))	1,86	1,25	3,11
	CAA(R(D))	1,84	1,41	3,26
	AAG(D)	2,18	2,16	4,33
AAG	CTT(RC(D))	2,12	2,15	4,27
	TTC(C(D))	2,16	2,18	4,33
	GAA(R(D))	2,15	2,12	4,27
	ACA(D)	0,99	1,19	2,18
ACA	TGT(RC(D))	1,19	0,99	2,18
	TGT(C(D))	1,19	0,99	2,18
	ACA(R(D))	0,99	1,19	2,18
	ATA(D)	0,53	0,59	1,13
ATA	TAT(RC(D))	0,59	0,53	1,13
	TAT(C(D))	0,59	0,53	1,13
	ATA(R(D))	0,53	0,53	1,07
	AGA(D)	1,30	1,42	2,73
AGA	TCT(RC(D))	1,42	1,30	2,73
	TCT(C(D))	1,42	1,30	2,73
	AGA(R(D))	1,30	1,42	2,73
	AAA(D)	1,37	1,59	2,96
AAA	TTT(RC(D))	1,59	1,37	2,96
	TTT(C(D))	1,59	1,37	2,96
	AAA(R(D))	1,37	1,59	2,96

Composition of DNA sequence in NBPF7

Base	No.of bases	%
A	2911	26,84%
C	2438	22,48%
G	2474	22,81%
T	3022	27,87%
N	0	0,00%
Sum	10845	100,00%

% of coding bases from NBPF7 (including N bases)

76,71%

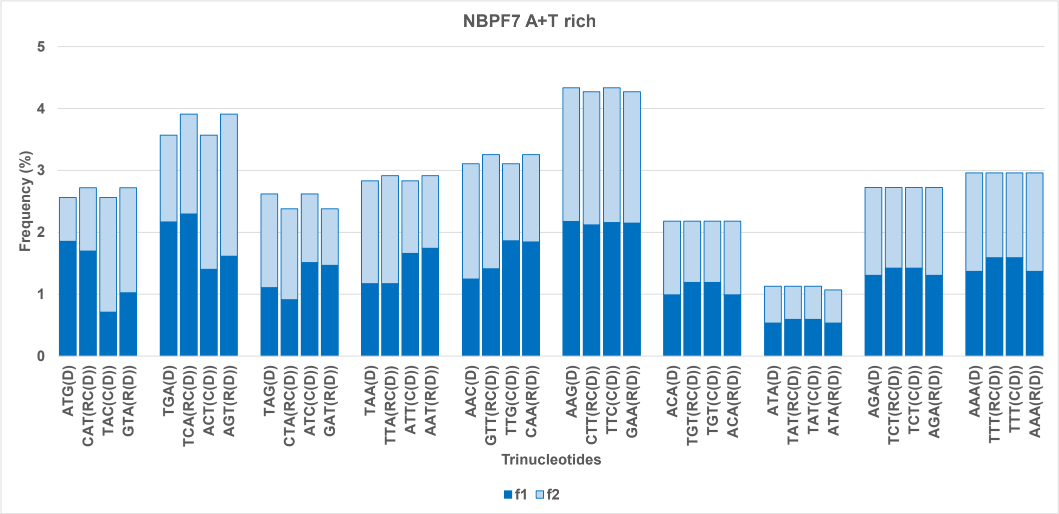

NBPF7 exons

A-T rich

D	f	%	RC	f	%	C	f	%	R	f	%	
**ATG**	31,00	2,34	CAT	17,00	1,28	TAC	7,00	0,53	GTA	8,00	0,60	Ia
**TGA**	37,00	2,79	TCA	33,00	2,49	ACT	22,00	1,66	AGT	22,00	1,66	
**TAG**	9,00	0,68	CTA	5,00	0,38	ATC	17,00	1,28	GAT	21,00	1,59	
**TAA**	9,00	0,68	TTA	7,00	0,53	ATT	10,00	0,76	AAT	25,00	1,89	Ib
**AAC**	24,00	1,81	GTT	12,00	0,91	TTG	15,00	1,13	CAA	38,00	2,87	
**AAG**	47,00	3,55	CTT	24,00	1,81	TTC	24,00	1,81	GAA	50,00	3,78	
**ACA**	27,00	2,04	TGT	11,00	0,83	TGT*			ACA*			Ic
**ATA**	10,00	0,76	TAT	5,00	0,38	TAT*			ATA*			
**AGA**	39,00	2,95	TCT	26,00	1,96	TCT*			AGA*			
**AAA**	48,00	3,63	TTT	4,00	0,30	TTT*			AAA*			

Quadruplet		f1	f2	fu
ATG	ATG(D)	2,34	0,53	2,87
	CAT(RC(D))	1,28	0,60	1,89
	TAC(C(D))	0,53	2,34	2,87
	GTA(R(D))	0,60	1,28	1,89
TGA	TGA(D)	2,79	1,66	4,46
	TCA(RC(D))	2,49	1,66	4,15
	ACT(C(D))	1,66	2,79	4,46
	AGT(R(D))	1,66	2,49	4,15
TAG	TAG(D)	0,68	1,28	1,96
	CTA(RC(D))	0,38	1,59	1,96
	ATC(C(D))	1,28	0,68	1,96
	GAT(R(D))	1,59	0,38	1,96
TAA	TAA(D)	0,68	0,76	1,44
	TTA(RC(D))	0,53	1,89	2,42
	ATT(C(D))	0,76	0,68	1,44
	AAT(R(D))	1,89	0,53	2,42
AAC	AAC(D)	1,81	1,13	2,95
	GTT(RC(D))	0,91	2,87	3,78
	TTG(C(D))	1,13	1,81	2,95
	CAA(R(D))	2,87	0,91	3,78
AAG	AAG(D)	3,55	1,81	5,36
	CTT(RC(D))	1,81	3,78	5,59
	TTC(C(D))	1,81	3,55	5,36
	GAA(R(D))	3,78	1,81	5,59
ACA	ACA(D)	1,02	0,42	1,44
	TGT(RC(D))	0,42	1,02	1,44
	TGT(C(D))	0,42	1,02	1,44
	ACA(R(D))	1,02	0,42	1,44
ATA	ATA(D)	0,38	0,19	0,57
	TAT(RC(D))	0,19	0,38	0,57
	TAT(C(D))	0,19	0,38	0,57
	ATA(R(D))	0,38	0,38	0,76
AGA	AGA(D)	1,47	0,98	2,45
	TCT(RC(D))	0,98	1,47	2,45
	TCT(C(D))	0,98	1,47	2,45
	AGA(R(D))	1,47	0,98	2,45
AAA	AAA(D)	1,81	0,15	1,96
	TTT(RC(D))	0,15	1,81	1,96
	TTT(C(D))	0,15	1,81	1,96
	AAA(R(D))	1,81	0,15	1,96

Composition of DNA sequence in NBPF7 exons

Base	No.of bases	%
A	415	31,30%
C	320	24,13%
G	331	24,96%
T	260	19,61%
N	0	0,00%
Sum	1326	100,00%

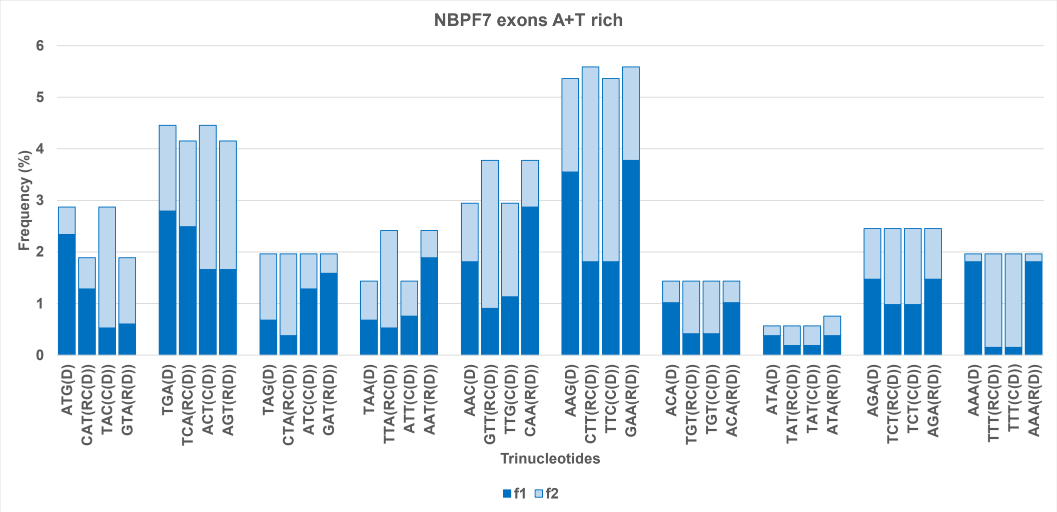

NBPF7 introns

A-T rich

D	f	%	RC	f	%	C	f	%	R	f	%	
**ATG**	145,00	1,74	CAT	149,00	1,79	TAC	59,00	0,71	GTA	93,00	1,12	Ia
**TGA**	163,00	1,96	TCA	185,00	2,22	ACT	110,00	1,32	AGT	132,00	1,59	
**TAG**	99,00	1,19	CTA	85,00	1,02	ATC	135,00	1,62	GAT	116,00	1,39	
**TAA**	104,00	1,25	TTA	104,00	1,25	ATT	144,00	1,73	AAT	140,00	1,68	Ib
**AAC**	86,00	1,03	GTT	121,00	1,45	TTG	160,00	1,92	CAA	133,00	1,60	
**AAG**	166,00	2,00	CTT	182,00	2,19	TTC	183,00	2,20	GAA	155,00	1,86	
**ACA**	153,00	1,84	TGT	221,00	2,66	TGT*			ACA*			Ic
**ATA**	96,00	1,15	TAT	115,00	1,38	TAT*			ATA*			
**AGA**	209,00	2,51	TCT	260,00	3,13	TCT*			AGA*			
**AAA**	214,00	2,57	TTT	309,00	3,72	TTT*			AAA*			

Quadruplet		f1	f2	fu
ATG	ATG(D)	1,74	0,71	2,45
	CAT(RC(D))	1,79	1,12	2,91
	TAC(C(D))	0,71	1,74	2,45
	GTA(R(D))	1,12	1,79	2,91
TGA	TGA(D)	1,96	1,32	3,28
	TCA(RC(D))	2,22	1,59	3,81
	ACT(C(D))	1,32	1,96	3,28
	AGT(R(D))	1,59	2,22	3,81
TAG	TAG(D)	1,19	1,62	2,81
	CTA(RC(D))	1,02	1,39	2,42
	ATC(C(D))	1,62	1,19	2,81
	GAT(R(D))	1,39	1,02	2,42
TAA	TAA(D)	1,25	1,73	2,98
	TTA(RC(D))	1,25	1,68	2,93
	ATT(C(D))	1,73	1,25	2,98
	AAT(R(D))	1,68	1,25	2,93
AAC	AAC(D)	1,03	1,92	2,96
	GTT(RC(D))	1,45	1,60	3,05
	TTG(C(D))	1,92	1,03	2,96
	CAA(R(D))	1,60	1,45	3,05
AAG	AAG(D)	2,00	2,20	4,20
	CTT(RC(D))	2,19	1,86	4,05
	TTC(C(D))	2,20	2,00	4,20
	GAA(R(D))	1,86	2,19	4,05
ACA	ACA(D)	0,92	1,33	2,25
	TGT(RC(D))	1,33	0,92	2,25
	TGT(C(D))	1,33	0,92	2,25
	ACA(R(D))	0,92	1,33	2,25
ATA	ATA(D)	0,58	0,69	1,27
	TAT(RC(D))	0,69	0,58	1,27
	TAT(C(D))	0,69	0,58	1,27
	ATA(R(D))	0,58	0,58	1,15
AGA	AGA(D)	1,26	1,56	2,82
	TCT(RC(D))	1,56	1,26	2,82
	TCT(C(D))	1,56	1,26	2,82
	AGA(R(D))	1,26	1,56	2,82
AAA	AAA(D)	1,29	1,86	3,14
	TTT(RC(D))	1,86	1,29	3,14
	TTT(C(D))	1,86	1,29	3,14
	AAA(R(D))	1,29	1,86	3,14

Composition of DNA sequence NBPF7 introns

Base	No.of bases	%
A	2145	25,78%
C	1849	22,23%
G	1884	22,65%
T	2441	29,34%
N	0	0,00%
Sum	8319	100,00%

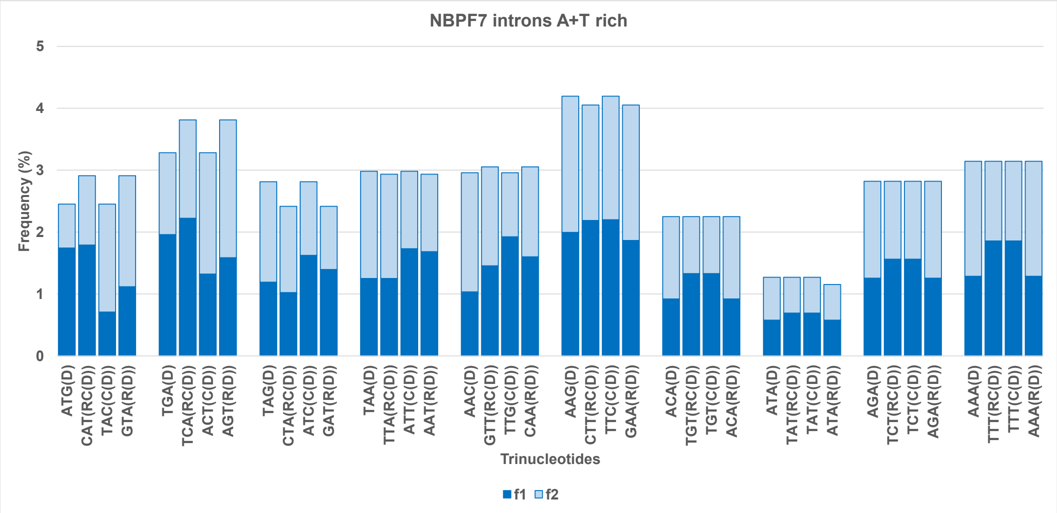

C+G rich												
**D**	**f**	**%**	**RC**	**f**	**%**	**C**	**f**	**%**	**R**	**f**	**%**	
**CGT**	28,00	0,26	ACG	21,00	0,19	GCA	176,00	1,62	TGC	179,00	1,65	IIa
**GTC**	130,00	1,20	GAC	121,00	1,12	CAG	278,00	2,56	CTG	290,00	2,67	
**GCT**	180,00	1,66	AGC	161,00	1,48	CGA	22,00	0,20	TCG	20,00	0,18	
**GCC**	134,00	1,24	GGC	153,00	1,41	CGG	23,00	0,21	CCG	29,00	0,27	IIb
**CCA**	215,00	1,98	TGG	214,00	1,97	GGT	127,00	1,17	ACC	138,00	1,27	
**CCT**	225,00	2,08	AGG	248,00	2,29	GGA	205,00	1,89	TCC	197,00	1,82	
**CAC**	193,00	1,78	GTG	193,00	1,78	GTG*			CAC*			IIc
**CGC**	19,00	0,18	GCG	22,00	0,20	GCG*			CGC*			
**CTC**	246,00	2,27	GAG	233,00	2,15	GAG*			CTC*			
**CCC**	157,00	1,45	GGG	143,00	1,32	GGG*			CCC*			

Quadruplet		f1	f2	fu
CGT	CGT(D)	0,26	1,62	1,88
	ACG(RC(D))	0,19	1,65	1,84
	GCA(C(D))	1,62	0,26	1,88
	TGC(R(D))	1,65	0,19	1,84
GTC	GTC(D)	1,20	2,56	3,76
	GAC(RC(D))	1,12	2,67	3,79
	CAG(C(D))	2,56	1,20	3,76
	CTG(R(D))	2,67	1,12	3,79
GCT	GCT(D)	1,66	0,20	1,86
	AGC(RC(D))	1,48	0,18	1,67
	CGA(C(D))	0,20	1,66	1,86
	TCG(R(D))	0,18	1,48	1,67
GCC	GCC(D)	1,24	0,21	1,45
	GGC(RC(D))	1,41	0,27	1,68
	CGG(C(D))	0,21	1,24	1,45
	CCG(R(D))	0,27	1,41	1,68
CCA	CCA(D)	1,98	1,17	3,15
	TGG(RC(D))	1,97	1,27	3,25
	GGT(C(D))	1,17	1,98	3,15
	ACC(R(D))	1,27	1,97	3,25
CCT	CCT(D)	2,08	1,89	3,97
	AGG(RC(D))	2,29	1,82	4,10
	GGA(C(D))	1,89	2,08	3,97
	TCC(R(D))	1,82	2,29	4,10
CAC	CAC(D)	0,89	0,89	1,78
	GTG(RC(D))	0,89	0,89	1,78
	GTG(C(D))	0,89	0,89	1,78
	CAC(R(D))	0,89	0,89	1,78
CGC	CGC(D)	0,09	0,10	0,19
	GCG(RC(D))	0,10	0,09	0,19
	GCG(C(D))	0,10	0,09	0,19
	CGC(R(D))	0,09	0,10	0,19
CTC	CTC(D)	1,13	1,07	2,21
	GAG(RC(D))	1,07	1,13	2,21
	GAG(C(D))	1,07	1,13	2,21
	CTC(R(D))	1,13	1,07	2,21
CCC	CCC(D)	0,72	0,66	1,38
	GGG(RC(D))	0,66	0,72	1,38
	GGG(C(D))	0,66	0,72	1,38
	CCC(R(D))	0,72	0,66	1,38

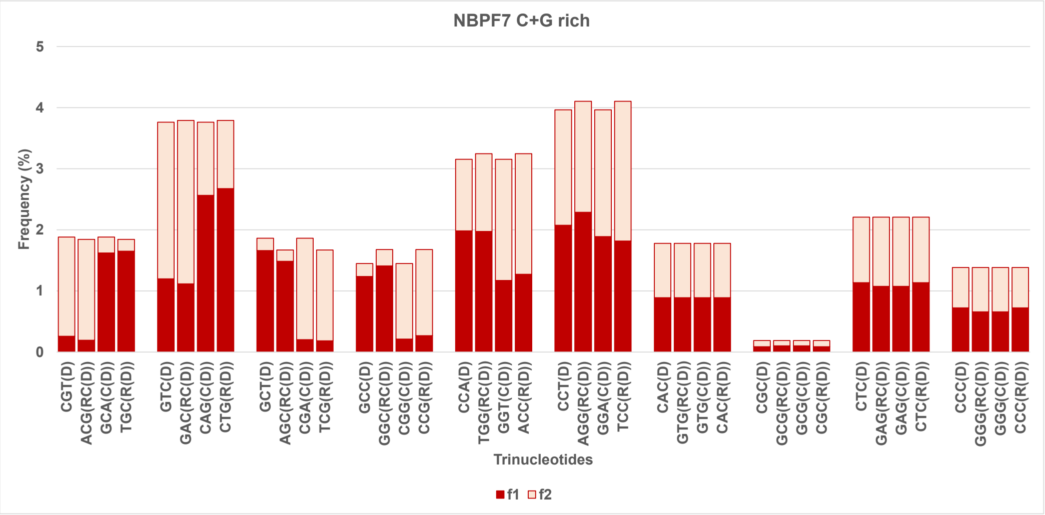

NBPF7 exons

C+G rich

D	f	%	RC	f	%	C	f	%	R	f	%	
**CGT**	2,00	0,15	ACG	6,00	0,45	GCA	28,00	2,11	TGC	22,00	1,66	IIa
**GTC**	12,00	0,91	GAC	13,00	0,98	CAG	42,00	3,17	CTG	36,00	2,72	
**GCT**	29,00	2,19	AGC	31,00	2,34	CGA	4,00	0,30				
**GCC**	20,00	1,51	GGC	23,00	1,74	CGG	6,00	0,45	CCG	7,00	0,53	IIb
**CCA**	33,00	2,49	TGG	25,00	1,89	GGT	10,00	0,76	ACC	13,00	0,98	
**CCT**	20,00	1,51	AGG	41,00	3,10	GGA	39,00	2,95	TCC	26,00	1,96	
**CAC**	24,00	1,81	GTG	13,00	0,98	GTG*			CAC*			IIc
**CGC**	4,00	0,30	GCG	3,00	0,23	GCG*			CGC*			
**CTC**	32,00	2,42	GAG	35,00	2,64	GAG*			CTC*			
**CCC**	26,00	1,96	GGG	15,00	1,13	GGG*			CCC*			

Quadruplet		f1	f2	fu
CGT	CGT(D)	0,15	2,11	2,27
	ACG(RC(D))	0,45	1,66	2,11
	GCA(C(D))	2,11	0,15	2,27
	TGC(R(D))	1,66	0,45	2,11
GTC	GTC(D)	0,91	3,17	4,08
	GAC(RC(D))	0,98	2,72	3,70
	CAG(C(D))	3,17	0,91	4,08
	CTG(R(D))	2,72	0,98	3,70
GCT	GCT(D)	2,19	0,30	2,49
	AGC(RC(D))	2,34	0,00	2,34
	CGA(C(D))	0,30	2,19	2,49
	TCG(R(D))	0,00	2,34	2,34
GCC	GCC(D)	1,51	0,45	1,96
	GGC(RC(D))	1,74	0,53	2,27
	CGG(C(D))	0,45	1,51	1,96
	CCG(R(D))	0,53	1,74	2,27
CCA	CCA(D)	2,49	0,76	3,25
	TGG(RC(D))	1,89	0,98	2,87
	GGT(C(D))	0,76	2,49	3,25
	ACC(R(D))	0,98	1,89	2,87
CCT	CCT(D)	1,51	2,95	4,46
	AGG(RC(D))	3,10	1,96	5,06
	GGA(C(D))	2,95	1,51	4,46
	TCC(R(D))	1,96	3,10	5,06
CAC	CAC(D)	0,91	0,49	1,40
	GTG(RC(D))	0,49	0,91	1,40
	GTG(C(D))	0,49	0,91	1,40
	CAC(R(D))	0,91	0,49	1,40
CGC	CGC(D)	0,15	0,11	0,26
	GCG(RC(D))	0,11	0,15	0,26
	GCG(C(D))	0,11	0,15	0,26
	CGC(R(D))	0,15	0,11	0,26
CTC	CTC(D)	1,21	1,32	2,53
	GAG(RC(D))	1,32	1,21	2,53
	GAG(C(D))	1,32	1,21	2,53
	CTC(R(D))	1,21	1,32	2,53
CCC	CCC(D)	0,98	0,57	1,55
	GGG(RC(D))	0,57	0,98	1,55
	GGG(C(D))	0,57	0,98	1,55
	CCC(R(D))	0,98	0,57	1,55

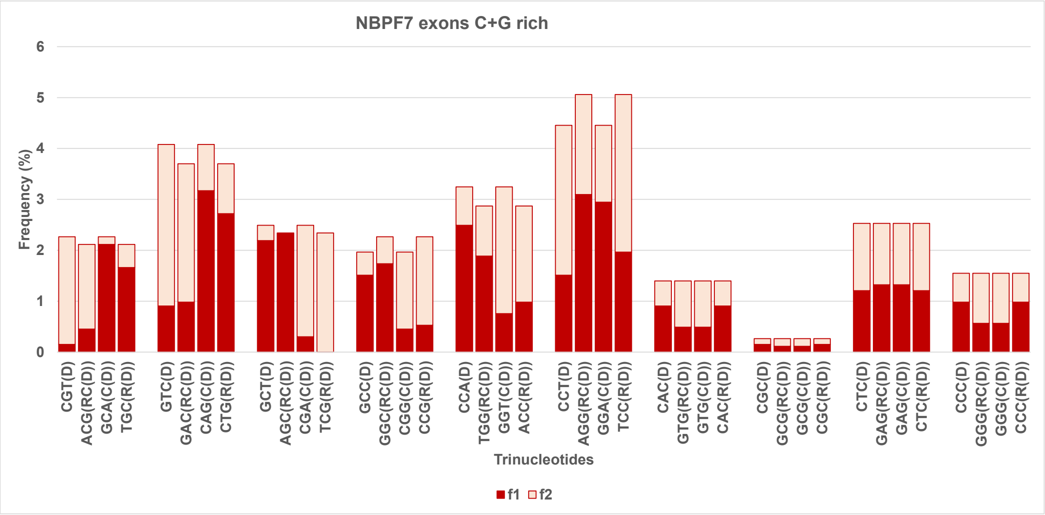

NBPF7 introns

C+G rich

D	f	%	RC	f	%	C	f	%	R	f	%	
**CGT**	23,00	0,28	ACG	13,00	0,16	GCA	129,00	1,55	TGC	137,00	1,65	IIa
**GTC**	107,00	1,29	GAC	91,00	1,09	CAG	204,00	2,45	CTG	225,00	2,71	
**GCT**	132,00	1,59	AGC	114,00	1,37	CGA	14,00	0,17	TCG	17,00	0,20	
**GCC**	104,00	1,25	GGC	118,00	1,42	CGG	15,00	0,18	CCG	18,00	0,22	IIb
**CCA**	160,00	1,92	TGG	171,00	2,06	GGT	106,00	1,27	ACC	101,00	1,21	
**CCT**	180,00	2,16	AGG	186,00	2,24	GGA	148,00	1,78	TCC	153,00	1,84	
**CAC**	141,00	1,70	GTG	162,00	1,95	GTG*			CAC*			IIc
**CGC**	13,00	0,16	GCG	17,00	0,20	GCG*			CGC*			
**CTC**	190,00	2,28	GAG	172,00	2,07	GAG*			CTC*			
**CCC**	117,00	1,41	GGG	113,00	1,36	GGG*			CCC*			

Quadruplet		f1	f2	fu
CGT	CGT(D)	0,28	1,55	1,83
	ACG(RC(D))	0,16	1,65	1,80
	GCA(C(D))	1,55	0,28	1,83
	TGC(R(D))	1,65	0,16	1,80
GTC	GTC(D)	1,29	2,45	3,74
	GAC(RC(D))	1,09	2,71	3,80
	CAG(C(D))	2,45	1,29	3,74
	CTG(R(D))	2,71	1,09	3,80
GCT	GCT(D)	1,59	0,17	1,76
	AGC(RC(D))	1,37	0,20	1,58
	CGA(C(D))	0,17	1,59	1,76
	TCG(R(D))	0,20	1,37	1,58
GCC	GCC(D)	1,25	0,18	1,43
	GGC(RC(D))	1,42	0,22	1,64
	CGG(C(D))	0,18	1,25	1,43
	CCG(R(D))	0,22	1,42	1,64
CCA	CCA(D)	1,92	1,27	3,20
	TGG(RC(D))	2,06	1,21	3,27
	GGT(C(D))	1,27	1,92	3,20
	ACC(R(D))	1,21	2,06	3,27
CCT	CCT(D)	2,16	1,78	3,94
	AGG(RC(D))	2,24	1,84	4,08
	GGA(C(D))	1,78	2,16	3,94
	TCC(R(D))	1,84	2,24	4,08
CAC	CAC(D)	0,85	0,97	1,82
	GTG(RC(D))	0,97	0,85	1,82
	GTG(C(D))	0,97	0,85	1,82
	CAC(R(D))	0,85	0,97	1,82
CGC	CGC(D)	0,08	0,10	0,18
	GCG(RC(D))	0,10	0,08	0,18
	GCG(C(D))	0,10	0,08	0,18
	CGC(R(D))	0,08	0,10	0,18
CTC	CTC(D)	1,14	1,03	2,18
	GAG(RC(D))	1,03	1,14	2,18
	GAG(C(D))	1,03	1,14	2,18
	CTC(R(D))	1,14	1,03	2,18
CCC	CCC(D)	0,70	0,68	1,38
	GGG(RC(D))	0,68	0,70	1,38
	GGG(C(D))	0,68	0,70	1,38
	CCC(R(D))	0,70	0,68	1,38

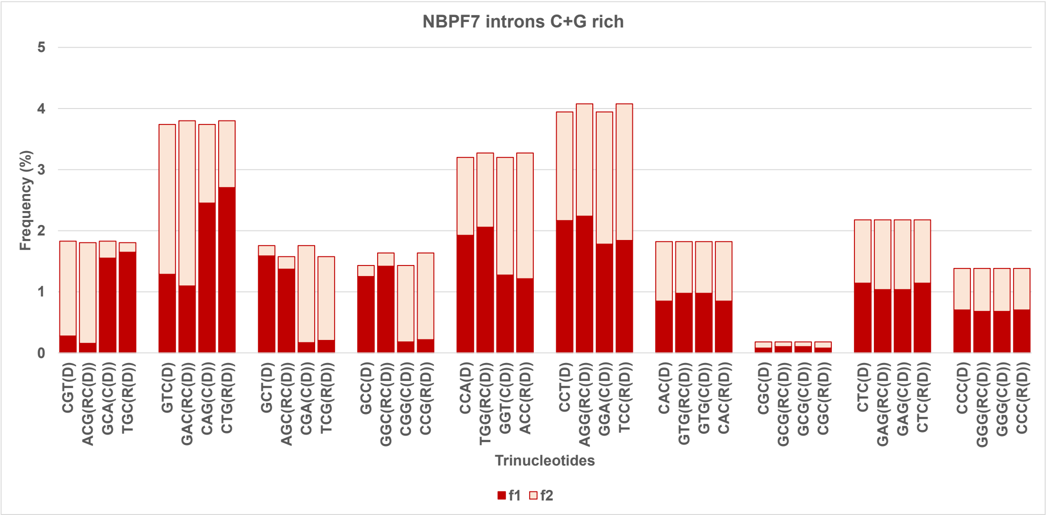

NBPF8 sequence

A-T rich												
**D**	**f**	**%**	**RC**	**f**	**%**	**C**	**f**	**%**	**R**	**f**	**%**	
**ATG**	676,00	2,07	CAT	645,00	1,98	TAC	289,00	0,89	GTA	309,00	0,95	Ia
**TGA**	767,00	2,35	TCA	725,00	2,22	ACT	487,00	1,49	AGT	546,00	1,68	
**TAG**	344,00	1,06	CTA	361,00	1,11	ATC	448,00	1,37	GAT	427,00	1,31	
**TAA**	426,00	1,31	TTA	436,00	1,34	ATT	615,00	1,89	AAT	620,00	1,90	Ib
**AAC**	424,00	1,30	GTT	518,00	1,59	TTG	696,00	2,14	CAA	531,00	1,63	
**AAG**	607,00	1,86	CTT	688,00	2,11	TTC	689,00	2,11	GAA	694,00	2,13	
**ACA**	601,00	1,84	TGT	915,00	2,81	TGT*			ACA*			Ic
**ATA**	396,00	1,22	TAT	443,00	1,36	TAT*			ATA*			
**AGA**	713,00	2,19	TCT	949,00	2,91	TCT*			AGA*			
**AAA**	875,00	2,69	TTT	1029,00	3,16	TTT*			AAA*			

Quadruplet		f1	f2	fu
ATG	ATG(D)	2,07	0,89	2,96
	CAT(RC(D))	1,98	0,95	2,93
	TAC(C(D))	0,89	2,07	2,96
	GTA(R(D))	0,95	1,98	2,93
TGA	TGA(D)	2,35	1,49	3,85
	TCA(RC(D))	2,22	1,68	3,90
	ACT(C(D))	1,49	2,35	3,85
	AGT(R(D))	1,68	2,22	3,90
TAG	TAG(D)	1,06	1,37	2,43
	CTA(RC(D))	1,11	1,31	2,42
	ATC(C(D))	1,37	1,06	2,43
	GAT(R(D))	1,31	1,11	2,42
TAA	TAA(D)	1,31	1,89	3,19
	TTA(RC(D))	1,34	1,90	3,24
	ATT(C(D))	1,89	1,31	3,19
	AAT(R(D))	1,90	1,34	3,24
AAC	AAC(D)	1,30	2,14	3,44
	GTT(RC(D))	1,59	1,63	3,22
	TTG(C(D))	2,14	1,30	3,44
	CAA(R(D))	1,63	1,59	3,22
AAG	AAG(D)	1,86	2,11	3,98
	CTT(RC(D))	2,11	2,13	4,24
	TTC(C(D))	2,11	1,86	3,98
	GAA(R(D))	2,13	2,11	4,24
ACA	ACA(D)	0,92	1,40	2,33
	TGT(RC(D))	1,40	0,92	2,33
	TGT(C(D))	1,40	0,92	2,33
	ACA(R(D))	0,92	1,40	2,33
ATA	ATA(D)	0,61	0,68	1,29
	TAT(RC(D))	0,68	0,61	1,29
	TAT(C(D))	0,68	0,61	1,29
	ATA(R(D))	0,61	0,61	1,22
AGA	AGA(D)	1,09	1,46	2,55
	TCT(RC(D))	1,46	1,09	2,55
	TCT(C(D))	1,46	1,09	2,55
	AGA(R(D))	1,09	1,46	2,55
AAA	AAA(D)	1,34	1,58	2,92
	TTT(RC(D))	1,58	1,34	2,92
	TTT(C(D))	1,58	1,34	2,92
	AAA(R(D))	1,34	1,58	2,92

Composition of DNA sequence in NBPF8

Base	No.of bases	%
A	8608	26,42%
C	7044	21,62%
G	7448	22,86%
T	9487	29,11%
N	0	0,00%
Sum	32587	100,00%

% of coding bases from NBPF8 (including N bases)

79,96%

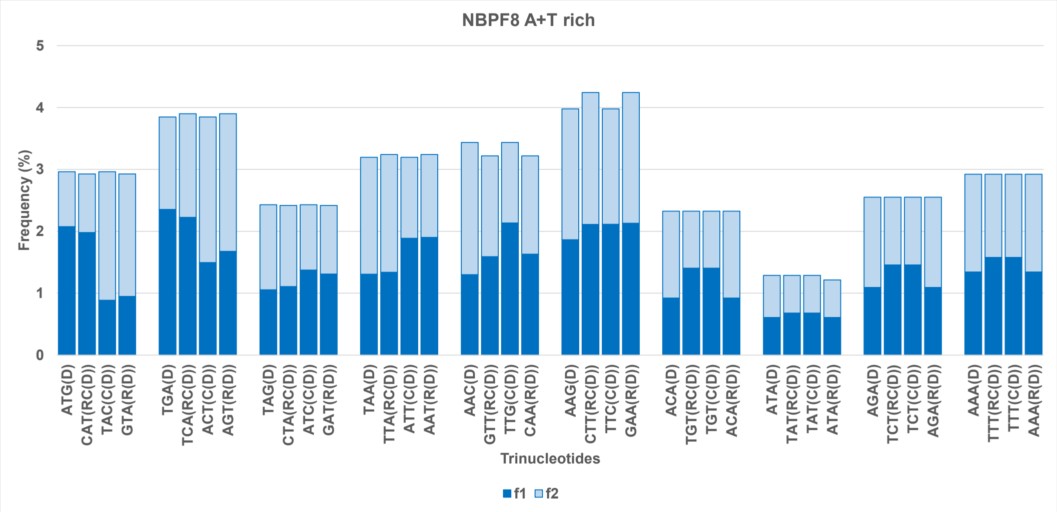

NBPF8 exons

A-T rich												
**D**	**f**	**%**	**RC**	**f**	**%**	**C**	**f**	**%**	**R**	**f**	**%**	
**ATG**	111,00	2,08	CAT	96,00	1,80	TAC	34,00	0,64	GTA	37,00	0,69	Ia
**TGA**	132,00	2,48	TCA	152,00	2,85	ACT	79,00	1,48	AGT	92,00	1,73	
**TAG**	45,00	0,84	CTA	36,00	0,68	ATC	65,00	1,22	GAT	69,00	1,30	
**TAA**	47,00	0,88	TTA	61,00	1,14	ATT	81,00	1,52	AAT	84,00	1,58	Ib
**AAC**	85,00	1,60	GTT	80,00	1,50	TTG	93,00	1,75	CAA	112,00	2,10	
**AAG**	160,00	3,00	CTT	73,00	1,37	TTC	80,00	1,50	GAA	170,00	3,19	
**ACA**	111,00	2,08	TGT	88,00	1,65	TGT*			ACA*			Ic
**ATA**	48,00	0,90	TAT	56,00	1,05	TAT*			ATA*			
**AGA**	178,00	3,34	TCT	98,00	1,84	TCT*			AGA*			
**AAA**	138,00	2,59	TTT	95,00	1,78	TTT*			AAA*			

Quadruplet		f1	f2	fu
ATG	ATG(D)	2,08	0,64	2,72
	CAT(RC(D))	1,80	0,69	2,50
	TAC(C(D))	0,64	2,08	2,72
	GTA(R(D))	0,69	1,80	2,50
TGA	TGA(D)	2,48	1,48	3,96
	TCA(RC(D))	2,85	1,73	4,58
	ACT(C(D))	1,48	2,48	3,96
	AGT(R(D))	1,73	2,85	4,58
TAG	TAG(D)	0,84	1,22	2,06
	CTA(RC(D))	0,68	1,30	1,97
	ATC(C(D))	1,22	0,84	2,06
	GAT(R(D))	1,30	0,68	1,97
TAA	TAA(D)	0,88	1,52	2,40
	TTA(RC(D))	1,14	1,58	2,72
	ATT(C(D))	1,52	0,88	2,40
	AAT(R(D))	1,58	1,14	2,72
AAC	AAC(D)	1,60	1,75	3,34
	GTT(RC(D))	1,50	2,10	3,60
	TTG(C(D))	1,75	1,60	3,34
	CAA(R(D))	2,10	1,50	3,60
AAG	AAG(D)	3,00	1,50	4,50
	CTT(RC(D))	1,37	3,19	4,56
	TTC(C(D))	1,50	3,00	4,50
	GAA(R(D))	3,19	1,37	4,56
ACA	ACA(D)	1,04	0,83	1,87
	TGT(RC(D))	0,83	1,04	1,87
	TGT(C(D))	0,83	1,04	1,87
	ACA(R(D))	1,04	0,83	1,87
ATA	ATA(D)	0,45	0,53	0,98
	TAT(RC(D))	0,53	0,45	0,98
	TAT(C(D))	0,53	0,45	0,98
	ATA(R(D))	0,45	0,45	0,90
AGA	AGA(D)	1,67	0,92	2,59
	TCT(RC(D))	0,92	1,67	2,59
	TCT(C(D))	0,92	1,67	2,59
	AGA(R(D))	1,67	0,92	2,59
AAA	AAA(D)	1,30	0,89	2,19
	TTT(RC(D))	0,89	1,30	2,19
	TTT(C(D))	0,89	1,30	2,19
	AAA(R(D))	1,30	0,89	2,19

Composition of DNA sequence in NBPF8 exons

Base	No.of bases	%
A	1576	29,57%
C	1202	22,55%
G	1304	24,47%
T	1248	23,41%
N	0	0,00%
Sum	5330	100,00%

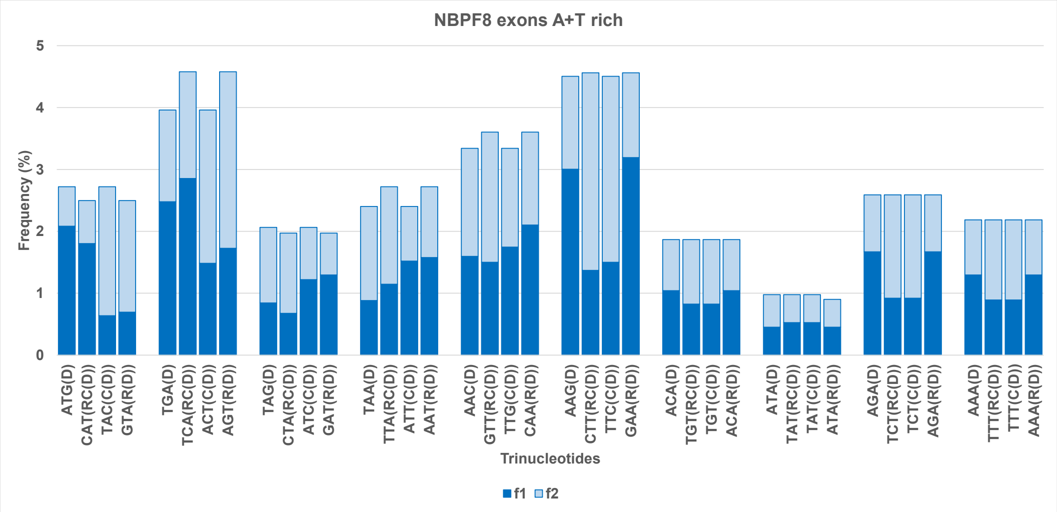

NBPF8 introns

A-T rich

D	f	%	RC	f	%	C	f	%	R	f	%	
**ATG**	111,00	2,08	CAT	96,00	1,80	TAC	34,00	0,64	GTA	37,00	0,69	Ia
**TGA**	132,00	2,48	TCA	152,00	2,85	ACT	79,00	1,48	AGT	92,00	1,73	
**TAG**	45,00	0,84	CTA	36,00	0,68	ATC	65,00	1,22	GAT	69,00	1,30	
**TAA**	47,00	0,88	TTA	61,00	1,14	ATT	81,00	1,52	AAT	84,00	1,58	Ib
**AAC**	85,00	1,60	GTT	80,00	1,50	TTG	93,00	1,75	CAA	112,00	2,10	
**AAG**	160,00	3,00	CTT	73,00	1,37	TTC	80,00	1,50	GAA	170,00	3,19	
**ACA**	111,00	2,08	TGT	88,00	1,65	TGT*			ACA*			Ic
**ATA**	48,00	0,90	TAT	56,00	1,05	TAT*			ATA*			
**AGA**	178,00	3,34	TCT	98,00	1,84	TCT*			AGA*			
**AAA**	138,00	2,59	TTT	95,00	1,78	TTT*			AAA*			

Quadruplet		f1	f2	fu
ATG	ATG(D)	2,08	0,64	2,72
	CAT(RC(D))	1,80	0,69	2,50
	TAC(C(D))	0,64	2,08	2,72
	GTA(R(D))	0,69	1,80	2,50
TGA	TGA(D)	2,48	1,48	3,96
	TCA(RC(D))	2,85	1,73	4,58
	ACT(C(D))	1,48	2,48	3,96
	AGT(R(D))	1,73	2,85	4,58
TAG	TAG(D)	0,84	1,22	2,06
	CTA(RC(D))	0,68	1,30	1,97
	ATC(C(D))	1,22	0,84	2,06
	GAT(R(D))	1,30	0,68	1,97
TAA	TAA(D)	0,88	1,52	2,40
	TTA(RC(D))	1,14	1,58	2,72
	ATT(C(D))	1,52	0,88	2,40
	AAT(R(D))	1,58	1,14	2,72
AAC	AAC(D)	1,60	1,75	3,34
	GTT(RC(D))	1,50	2,10	3,60
	TTG(C(D))	1,75	1,60	3,34
	CAA(R(D))	2,10	1,50	3,60
AAG	AAG(D)	3,00	1,50	4,50
	CTT(RC(D))	1,37	3,19	4,56
	TTC(C(D))	1,50	3,00	4,50
	GAA(R(D))	3,19	1,37	4,56
ACA	ACA(D)	1,04	0,83	1,87
	TGT(RC(D))	0,83	1,04	1,87
	TGT(C(D))	0,83	1,04	1,87
	ACA(R(D))	1,04	0,83	1,87
ATA	ATA(D)	0,45	0,53	0,98
	TAT(RC(D))	0,53	0,45	0,98
	TAT(C(D))	0,53	0,45	0,98
	ATA(R(D))	0,45	0,45	0,90
AGA	AGA(D)	1,67	0,92	2,59
	TCT(RC(D))	0,92	1,67	2,59
	TCT(C(D))	0,92	1,67	2,59
	AGA(R(D))	1,67	0,92	2,59
AAA	AAA(D)	1,30	0,89	2,19
	TTT(RC(D))	0,89	1,30	2,19
	TTT(C(D))	0,89	1,30	2,19
	AAA(R(D))	1,30	0,89	2,19

Composition of DNA sequence NBPF8 introns

Base	No.of bases	%
A	6716	25,77%
C	5616	21,55%
G	5885	22,59%
T	7840	30,09%
N	0	0,00%
Sum	26057	100,00%

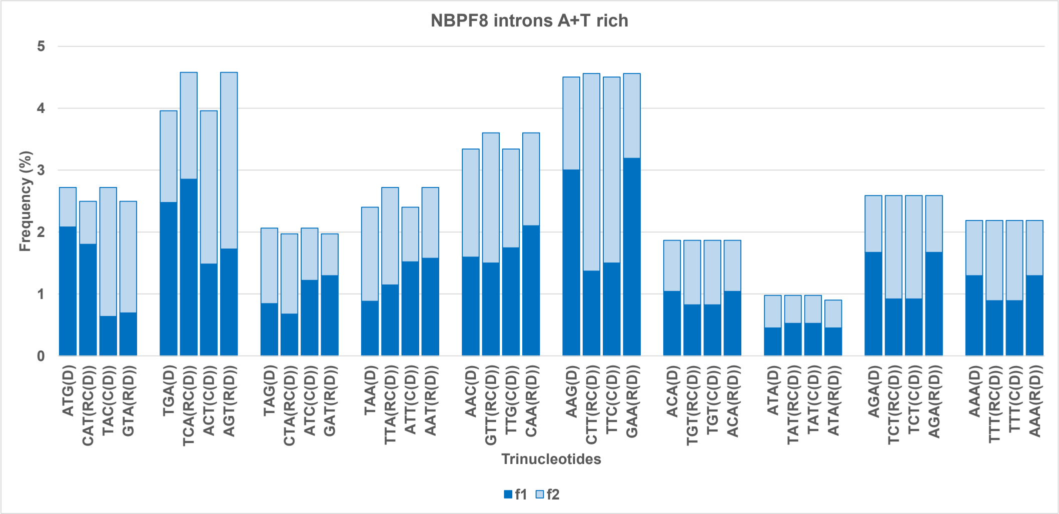

NBPF8 sequence

						C+G rich						
**D**	**f**	**%**	**RC**	**f**	**%**	**C**	**f**	**%**	**R**	**f**	**%**	
**CGT**	87,00	0,27	ACG	84,00	0,26	GCA	520,00	1,60	TGC	469,00	1,44	IIa
**GTC**	460,00	1,41	GAC	397,00	1,22	CAG	774,00	2,38	CTG	825,00	2,53	
**GCT**	484,00	1,49	AGC	434,00	1,33	CGA	102,00	0,31	TCG	83,00	0,25	
**GCC**	366,00	1,12	GGC	478,00	1,47	CGG	76,00	0,23	CCG	87,00	0,27	IIb
**CCA**	560,00	1,72	TGG	674,00	2,07	GGT	367,00	1,13	ACC	394,00	1,21	
**CCT**	666,00	2,04	AGG	688,00	2,11	GGA	592,00	1,82	TCC	553,00	1,70	
**CAC**	456,00	1,40	GTG	628,00	1,93	GTG*			CAC*			IIc
**CGC**	74,00	0,23	GCG	85,00	0,26	GCG*			CGC*			
**CTC**	713,00	2,19	GAG	656,00	2,01	GAG*			CTC*			
**CCC**	399,00	1,22	GGG	465,00	1,43	GGG*			CCC*			

Quadruplet		f1	f2	fu
	CGT(D)	0,27	1,60	1,86
CGT	ACG(RC(D))	0,26	1,44	1,70
	GCA(C(D))	1,60	0,27	1,86
	TGC(R(D))	1,44	0,26	1,70
	GTC(D)	1,41	2,38	3,79
GTC	GAC(RC(D))	1,22	2,53	3,75
	CAG(C(D))	2,38	1,41	3,79
	CTG(R(D))	2,53	1,22	3,75
	GCT(D)	1,49	0,31	1,80
GCT	AGC(RC(D))	1,33	0,25	1,59
	CGA(C(D))	0,31	1,49	1,80
	TCG(R(D))	0,25	1,33	1,59
	GCC(D)	1,12	0,23	1,36
GCC	GGC(RC(D))	1,47	0,27	1,73
	CGG(C(D))	0,23	1,12	1,36
	CCG(R(D))	0,27	1,47	1,73
	CCA(D)	1,72	1,13	2,84
CCA	TGG(RC(D))	2,07	1,21	3,28
	GGT(C(D))	1,13	1,72	2,84
	ACC(R(D))	1,21	2,07	3,28
	CCT(D)	2,04	1,82	3,86
CCT	AGG(RC(D))	2,11	1,70	3,81
	GGA(C(D))	1,82	2,04	3,86
	TCC(R(D))	1,70	2,11	3,81
	CAC(D)	0,70	0,96	1,66
CAC	GTG(RC(D))	0,96	0,70	1,66
	GTG(C(D))	0,96	0,70	1,66
	CAC(R(D))	0,70	0,96	1,66
	CGC(D)	0,11	0,13	0,24
CGC	GCG(RC(D))	0,13	0,11	0,24
	GCG(C(D))	0,13	0,11	0,24
	CGC(R(D))	0,11	0,13	0,24
	CTC(D)	1,09	1,01	2,10
CTC	GAG(RC(D))	1,01	1,09	2,10
	GAG(C(D))	1,01	1,09	2,10
	CTC(R(D))	1,09	1,01	2,10
	CCC(D)	0,61	0,71	1,33
CCC	GGG(RC(D))	0,71	0,61	1,33
	GGG(C(D))	0,71	0,61	1,33
	CCC(R(D))	0,61	0,71	1,33

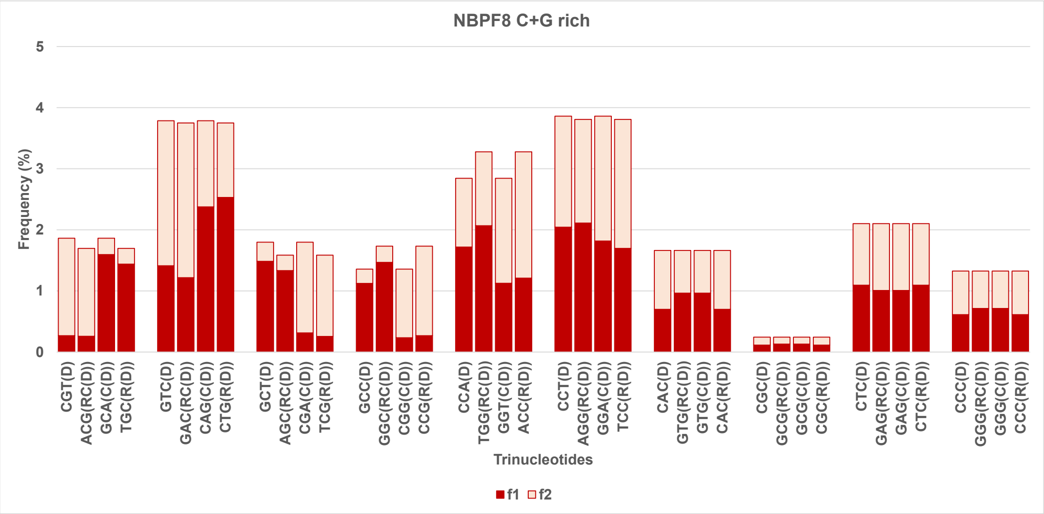

NBPF8 exons

C+G rich

D	f	%	RC	f	%	C	f	%	R	f	%	
**CGT**	18,00	0,34	ACG	26,00	0,49	GCA	91,00	1,71	TGC	79,00	1,48	IIa
**GTC**	70,00	1,31	GAC	86,00	1,61	CAG	174,00	3,27	CTG	128,00	2,40	
**GCT**	84,00	1,58	AGC	112,00	2,10	CGA	24,00	0,45	TCG	11,00	0,21	
**GCC**	84,00	1,58	GGC	72,00	1,35	CGG	21,00	0,39	CCG	22,00	0,41	IIb
**CCA**	100,00	1,88	TGG	93,00	1,75	GGT	49,00	0,92	ACC	60,00	1,13	
**CCT**	106,00	1,99	AGG	145,00	2,72	GGA	138,00	2,59	TCC	84,00	1,58	
**CAC**	71,00	1,33	GTG	60,00	1,13	GTG*			CAC*			IIc
**CGC**	9,00	0,17	GCG	13,00	0,24	GCG*			CGC*			
**CTC**	130,00	2,44	GAG	147,00	2,76	GAG*			CTC*			
**CCC**	81,00	1,52	GGG	54,00	1,01	GGG*			CCC*			

Quadruplet		f1	f2	fu
	CGT(D)	0,34	1,71	2,05
CGT	ACG(RC(D))	0,49	1,48	1,97
	GCA(C(D))	1,71	0,34	2,05
	TGC(R(D))	1,48	0,49	1,97
	GTC(D)	1,31	3,27	4,58
GTC	GAC(RC(D))	1,61	2,40	4,02
	CAG(C(D))	3,27	1,31	4,58
	CTG(R(D))	2,40	1,61	4,02
	GCT(D)	1,58	0,45	2,03
GCT	AGC(RC(D))	2,10	0,21	2,31
	CGA(C(D))	0,45	1,58	2,03
	TCG(R(D))	0,21	2,10	2,31
	GCC(D)	1,58	0,39	1,97
GCC	GGC(RC(D)	1,35	0,41	1,76
	CGG(C(D))	0,39	1,58	1,97
	CCG(R(D))	0,41	1,35	1,76
	CCA(D)	1,88	0,92	2,80
CCA	TGG(RC(D))	1,75	1,13	2,87
	GGT(C(D))	0,92	1,88	2,80
	ACC(R(D))	1,13	1,75	2,87
	CCT(D)	1,99	2,59	4,58
CCT	AGG(RC(D))	2,72	1,58	4,30
	GGA(C(D))	2,59	1,99	4,58
	TCC(R(D))	1,58	2,72	4,30
	CAC(D)	0,67	0,56	1,23
CAC	GTG(RC(D))	0,56	0,67	1,23
	GTG(C(D))	0,56	0,67	1,23
	CAC(R(D))	0,67	0,56	1,23
	CGC(D)	0,08	0,12	0,21
CGC	GCG(RC(D)	0,12	0,08	0,21
	GCG(C(D))	0,12	0,08	0,21
	CGC(R(D))	0,08	0,12	0,21
	CTC(D)	1,22	1,38	2,60
CTC	GAG(RC(D))	1,38	1,22	2,60
	GAG(C(D))	1,38	1,22	2,60
	CTC(R(D))	1,22	1,38	2,60
	CCC(D)	0,76	0,51	1,27
CCC	GGG(RC(D)	0,51	0,76	1,27
	GGG(C(D))	0,51	0,76	1,27
	CCC(R(D))	0,76	0,51	1,27

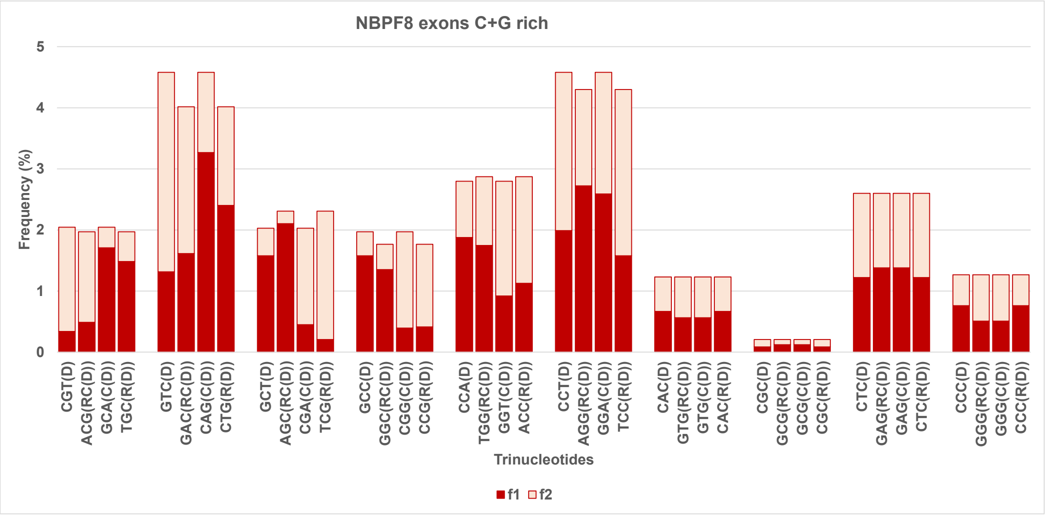

NBPF8 introns

C+G rich												
**D**	**f**	**%**	**RC**	**f**	**%**	**C**	**f**	**%**	**R**	**f**	**%**	
**CGT**	18,00	0,34	ACG	26,00	0,49	GCA	91,00	1,71	TGC	79,00	1,48	IIa
**GTC**	70,00	1,31	GAC	86,00	1,61	CAG	174,00	3,27	CTG	128,00	2,40	
**GCT**	84,00	1,58	AGC	112,00	2,10	CGA	24,00	0,45	TCG	11,00	0,21	
**GCC**	84,00	1,58	GGC	72,00	1,35	CGG	21,00	0,39	CCG	22,00	0,41	IIb
**CCA**	100,00	1,88	TGG	93,00	1,75	GGT	49,00	0,92	ACC	60,00	1,13	
**CCT**	106,00	1,99	AGG	145,00	2,72	GGA	138,00	2,59	TCC	84,00	1,58	
**CAC**	71,00	1,33	GTG	60,00	1,13	GTG*			CAC*			IIc
**CGC**	9,00	0,17	GCG	13,00	0,24	GCG*			CGC*			
**CTC**	130,00	2,44	GAG	147,00	2,76	GAG*			CTC*			
**CCC**	81,00	1,52	GGG	54,00	1,01	GGG*			CCC*			

Quadruplet		f1	f2	fu
CGT	CGT(D)	0,34	1,71	2,05
	ACG(RC(D))	0,49	1,48	1,97
	GCA(C(D))	1,71	0,34	2,05
	TGC(R(D))	1,48	0,49	1,97
GTC	GTC(D)	1,31	3,27	4,58
	GAC(RC(D))	1,61	2,40	4,02
	CAG(C(D))	3,27	1,31	4,58
	CTG(R(D))	2,40	1,61	4,02
GCT	GCT(D)	1,58	0,45	2,03
	AGC(RC(D))	2,10	0,21	2,31
	CGA(C(D))	0,45	1,58	2,03
	TCG(R(D))	0,21	2,10	2,31
GCC	GCC(D)	1,58	0,39	1,97
	GGC(RC(D)	1,35	0,41	1,76
	CGG(C(D))	0,39	1,58	1,97
	CCG(R(D))	0,41	1,35	1,76
CCA	CCA(D)	1,88	0,92	2,80
	TGG(RC(D))	1,75	1,13	2,87
	GGT(C(D))	0,92	1,88	2,80
	ACC(R(D))	1,13	1,75	2,87
CCT	CCT(D)	1,99	2,59	4,58
	AGG(RC(D))	2,72	1,58	4,30
	GGA(C(D))	2,59	1,99	4,58
	TCC(R(D))	1,58	2,72	4,30
CAC	CAC(D)	0,67	0,56	1,23
	GTG(RC(D))	0,56	0,67	1,23
	GTG(C(D))	0,56	0,67	1,23
	CAC(R(D))	0,67	0,56	1,23
CGC	CGC(D)	0,08	0,12	0,21
	GCG(RC(D)	0,12	0,08	0,21
	GCG(C(D))	0,12	0,08	0,21
	CGC(R(D))	0,08	0,12	0,21
CTC	CTC(D)	1,22	1,38	2,60
	GAG(RC(D))	1,38	1,22	2,60
	GAG(C(D))	1,38	1,22	2,60
	CTC(R(D))	1,22	1,38	2,60
CCC	CCC(D)	0,76	0,51	1,27
	GGG(RC(D)	0,51	0,76	1,27
	GGG(C(D))	0,51	0,76	1,27
	CCC(R(D))	0,76	0,51	1,27

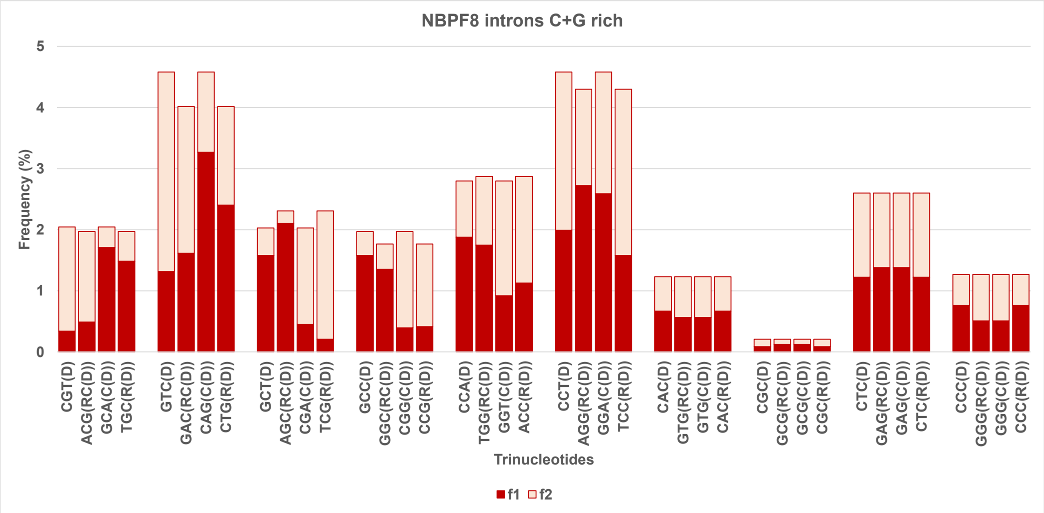

NBPF9 sequence

A-T rich												
**D**	**f**	**%**	**RC**	**f**	**%**	**C**	**f**	**%**	**R**	**f**	**%**	
**ATG**	1005,00	1,98	CAT	970,00	1,91	TAC	519,00	1,02	GTA	536,00	1,06	Ia
**TGA**	1105,00	2,18	TCA	1055,00	2,08	ACT	738,00	1,45	AGT	847,00	1,67	
**TAG**	597,00	1,18	CTA	584,00	1,15	ATC	654,00	1,29	GAT	681,00	1,34	
**TAA**	738,00	1,45	TTA	844,00	1,66	ATT	1113,00	2,19	AAT	1012,00	1,99	Ib
**AAC**	622,00	1,23	GTT	854,00	1,68	TTG	1053,00	2,08	CAA	766,00	1,51	
**AAG**	876,00	1,73	CTT	1064,00	2,10	TTC	1134,00	2,24	GAA	1006,00	1,98	
**ACA**	876,00	1,73	TGT	1406,00	2,77	TGT*			ACA*			Ic
**ATA**	764,00	1,51	TAT	874,00	1,72	TAT*			ATA*			
**AGA**	996,00	1,96	TCT	1349,00	2,66	TCT*			AGA*			
**AAA**	1408,00	2,78	TTT	1862,00	3,67	TTT*			AAA*			

Quadruplet		f1	f2	fu
ATG	ATG(D)	1,98	1,02	3,00
	CAT(RC(D))	1,91	1,06	2,97
	TAC(C(D))	1,02	1,98	3,00
	GTA(R(D))	1,06	1,91	2,97
TGA	TGA(D)	2,18	1,45	3,63
	TCA(RC(D))	2,08	1,67	3,75
	ACT(C(D))	1,45	2,18	3,63
	AGT(R(D))	1,67	2,08	3,75
TAG	TAG(D)	1,18	1,29	2,47
	CTA(RC(D))	1,15	1,34	2,49
	ATC(C(D))	1,29	1,18	2,47
	GAT(R(D))	1,34	1,15	2,49
TAA	TAA(D)	1,45	2,19	3,65
	TTA(RC(D))	1,66	1,99	3,66
	ATT(C(D))	2,19	1,45	3,65
	AAT(R(D))	1,99	1,66	3,66
AAC	AAC(D)	1,23	2,08	3,30
	GTT(RC(D))	1,68	1,51	3,19
	TTG(C(D))	2,08	1,23	3,30
	CAA(R(D))	1,51	1,68	3,19
AAG	AAG(D)	1,73	2,24	3,96
	CTT(RC(D))	2,10	1,98	4,08
	TTC(C(D))	2,24	1,73	3,96
	GAA(R(D))	1,98	2,10	4,08
ACA	ACA(D)	0,86	1,39	2,25
	TGT(RC(D))	1,39	0,86	2,25
	TGT(C(D))	1,39	0,86	2,25
	ACA(R(D))	0,86	1,39	2,25
ATA	ATA(D)	0,75	0,86	1,61
	TAT(RC(D))	0,86	0,75	1,61
	TAT(C(D))	0,86	0,75	1,61
	ATA(R(D))	0,75	0,75	1,51
AGA	AGA(D)	0,98	1,33	2,31
	TCT(RC(D))	1,33	0,98	2,31
	TCT(C(D))	1,33	0,98	2,31
	AGA(R(D))	0,98	1,33	2,31
AAA	AAA(D)	1,39	1,84	3,22
	TTT(RC(D))	1,84	1,39	3,22
	TTT(C(D))	1,84	1,39	3,22
	AAA(R(D))	1,39	1,84	3,22

Composition of DNA sequence in NBPF9

Base	No.of bases	%
A	13278	26,17%
C	10855	21,40%
G	11247	22,17%
T	15355	30,27%
N	0	0,00%
Sum	50735	100,00%

% of coding bases from NBPF9 (including N bases)

**86,09%**

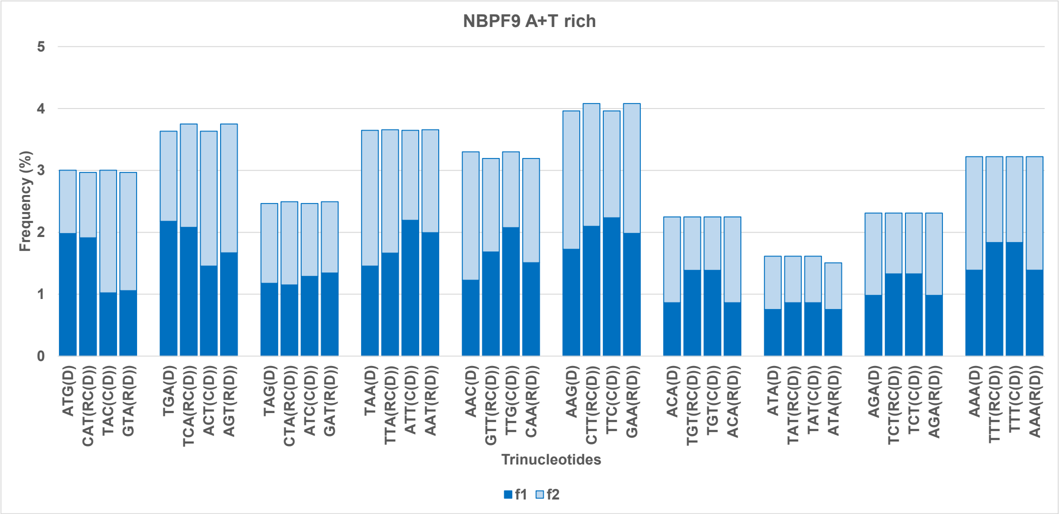

NBPF9 exons

A-T rich

D	f	%	RC	f	%	C	f	%	R	f	%	
**ATG**	111,00	1,90	CAT	92,00	1,58	TAC	49,00	0,84	GTA	41,00	0,70	Ia
**TGA**	136,00	2,33	TCA	142,00	2,43	ACT	87,00	1,49	AGT	93,00	1,59	
**TAG**	48,00	0,82	CTA	39,00	0,67	ATC	66,00	1,13	GAT	76,00	1,30	
**TAA**	44,00	0,75	TTA	77,00	1,32	ATT	90,00	1,54	AAT	84,00	1,44	Ib
**AAC**	86,00	1,47	GTT	89,00	1,53	TTG	110,00	1,89	CAA	110,00	1,89	
**AAG**	175,00	3,00	CTT	93,00	1,59	TTC	85,00	1,46	GAA	191,00	3,27	
**ACA**	105,00	1,80	TGT	96,00	1,65	TGT*			ACA*			Ic
**ATA**	46,00	0,79	TAT	61,00	1,05	TAT*			ATA*			
**AGA**	175,00	3,00	TCT	94,00	1,61	TCT*			AGA*			
**AAA**	155,00	2,66	TTT	114,00	1,95	TTT*			AAA*			

Quadruplet		f1	f2	fu
ATG	ATG(D)	1,90	0,84	2,74
	CAT(RC(D))	1,58	0,70	2,28
	TAC(C(D))	0,84	1,90	2,74
	GTA(R(D))	0,70	1,58	2,28
TGA	TGA(D)	2,33	1,49	3,82
	TCA(RC(D))	2,43	1,59	4,03
	ACT(C(D))	1,49	2,33	3,82
	AGT(R(D))	1,59	2,43	4,03
TAG	TAG(D)	0,82	1,13	1,95
	CTA(RC(D))	0,67	1,30	1,97
	ATC(C(D))	1,13	0,82	1,95
	GAT(R(D))	1,30	0,67	1,97
TAA	TAA(D)	0,75	1,54	2,30
	TTA(RC(D))	1,32	1,44	2,76
	ATT(C(D))	1,54	0,75	2,30
	AAT(R(D))	1,44	1,32	2,76
AAC	AAC(D)	1,47	1,89	3,36
	GTT(RC(D))	1,53	1,89	3,41
	TTG(C(D))	1,89	1,47	3,36
	CAA(R(D))	1,89	1,53	3,41
AAG	AAG(D)	3,00	1,46	4,46
	CTT(RC(D))	1,59	3,27	4,87
	TTC(C(D))	1,46	3,00	4,46
	GAA(R(D))	3,27	1,59	4,87
ACA	ACA(D)	0,90	0,82	1,72
	TGT(RC(D))	0,82	0,90	1,72
	TGT(C(D))	0,82	0,90	1,72
	ACA(R(D))	0,90	0,82	1,72
ATA	ATA(D)	0,39	0,52	0,92
	TAT(RC(D))	0,52	0,39	0,92
	TAT(C(D))	0,52	0,39	0,92
	ATA(R(D))	0,39	0,39	0,79
AGA	AGA(D)	1,50	0,81	2,31
	TCT(RC(D))	0,81	1,50	2,31
	TCT(C(D))	0,81	1,50	2,31
	AGA(R(D))	1,50	0,81	2,31
AAA	AAA(D)	1,33	0,98	2,31
	TTT(RC(D))	0,98	1,33	2,31
	TTT(C(D))	0,98	1,33	2,31
	AAA(R(D))	1,33	0,98	2,31

Composition of DNA sequence in NBPF9 exons

Base	No.of bases	%
A	1652	28,31%
C	1307	22,40%
G	1496	25,64%
T	1380	23,65%
N	0	0,00%
Sum	5835	100,00%

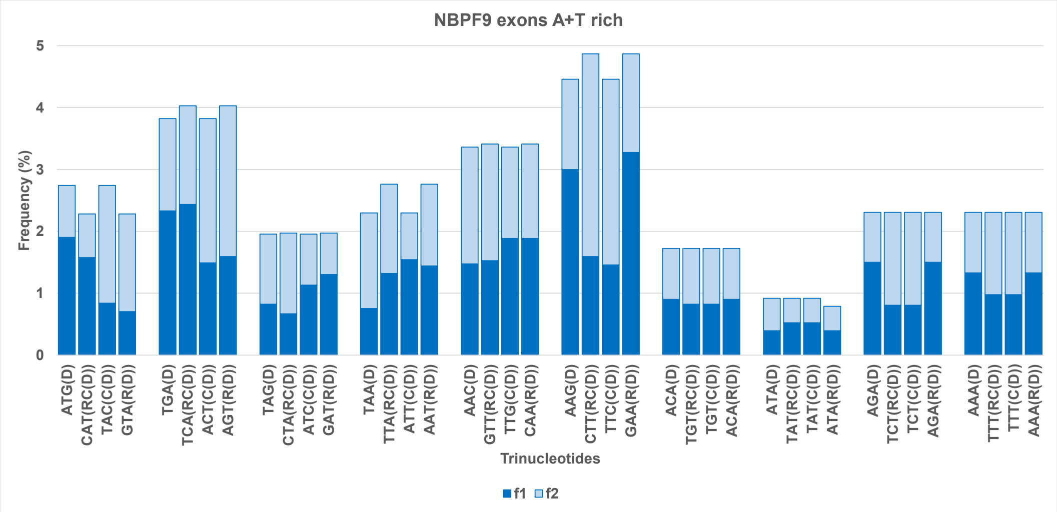

NBPF9 introns

A-T rich												
**D**	**f**	**%**	**RC**	**f**	**%**	**C**	**f**	**%**	**R**	**f**	**%**	
**ATG**	876,00	2,01	CAT	855,00	1,96	TAC	456,00	1,04	GTA	485,00	1,11	Ia
**TGA**	953,00	2,18	TCA	889,00	2,04	ACT	638,00	1,46	AGT	727,00	1,66	
**TAG**	535,00	1,22	CTA	529,00	1,21	ATC	574,00	1,31	GAT	596,00	1,36	
**TAA**	673,00	1,54	TTA	739,00	1,69	ATT	996,00	2,28	AAT	906,00	2,07	Ib
**AAC**	528,00	1,21	GTT	742,00	1,70	TTG	921,00	2,11	CAA	646,00	1,48	
**AAG**	688,00	1,58	CTT	942,00	2,16	TTC	1020,00	2,34	GAA	803,00	1,84	
**ACA**	756,00	1,73	TGT	1266,00	2,90	TGT*			ACA*			Ic
**ATA**	701,00	1,61	TAT	790,00	1,81	TAT*			ATA*			
**AGA**	809,00	1,85	TCT	1221,00	2,80	TCT*			AGA*			
**AAA**	1228,00	2,81	TTT	1689,00	3,87	TTT*			AAA*			

Quadruplet		f1	f2	fu
ATG	ATG(D)	2,01	1,04	3,05
	CAT(RC(D))	1,96	1,11	3,07
	TAC(C(D))	1,04	2,01	3,05
	GTA(R(D))	1,11	1,96	3,07
TGA	TGA(D)	2,18	1,46	3,64
	TCA(RC(D))	2,04	1,66	3,70
	ACT(C(D))	1,46	2,18	3,64
	AGT(R(D))	1,66	2,04	3,70
TAG	TAG(D)	1,22	1,31	2,54
	CTA(RC(D))	1,21	1,36	2,58
	ATC(C(D))	1,31	1,22	2,54
	GAT(R(D))	1,36	1,21	2,58
TAA	TAA(D)	1,54	2,28	3,82
	TTA(RC(D))	1,69	2,07	3,77
	ATT(C(D))	2,28	1,54	3,82
	AAT(R(D))	2,07	1,69	3,77
AAC	AAC(D)	1,21	2,11	3,32
	GTT(RC(D))	1,70	1,48	3,18
	TTG(C(D))	2,11	1,21	3,32
	CAA(R(D))	1,48	1,70	3,18
AAG	AAG(D)	1,58	2,34	3,91
	CTT(RC(D))	2,16	1,84	4,00
	TTC(C(D))	2,34	1,58	3,91
	GAA(R(D))	1,84	2,16	4,00
ACA	ACA(D) c		1,45	##########
	TGT(RC(D))	1,45	0,87	2,31
	TGT(C(D))	1,45	0,87	2,31
	ACA(R(D))	0,87	1,45	2,31
ATA	ATA(D)	0,80	0,90	1,71
	TAT(RC(D))	0,90	0,80	1,71
	TAT(C(D))	0,90	0,80	1,71
	ATA(R(D))	0,80	0,80	1,61
AGA	AGA(D)	0,93	1,40	2,32
	TCT(RC(D))	1,40	0,93	2,32
	TCT(C(D))	1,40	0,93	2,32
	AGA(R(D))	0,93	1,40	2,32
AAA	AAA(D)	1,41	1,93	3,34
	TTT(RC(D))	1,93	1,41	3,34
	TTT(C(D))	1,93	1,41	3,34
	AAA(R(D))	1,41	1,93	3,34

Composition of DNA sequence NBPF9 introns

Base	No.of bases	%
A	11371	26,03%
C	9271	21,23%
G	9462	21,66%
T	13574	31,08%
N	0	0,00%
Sum	43678	100,00%

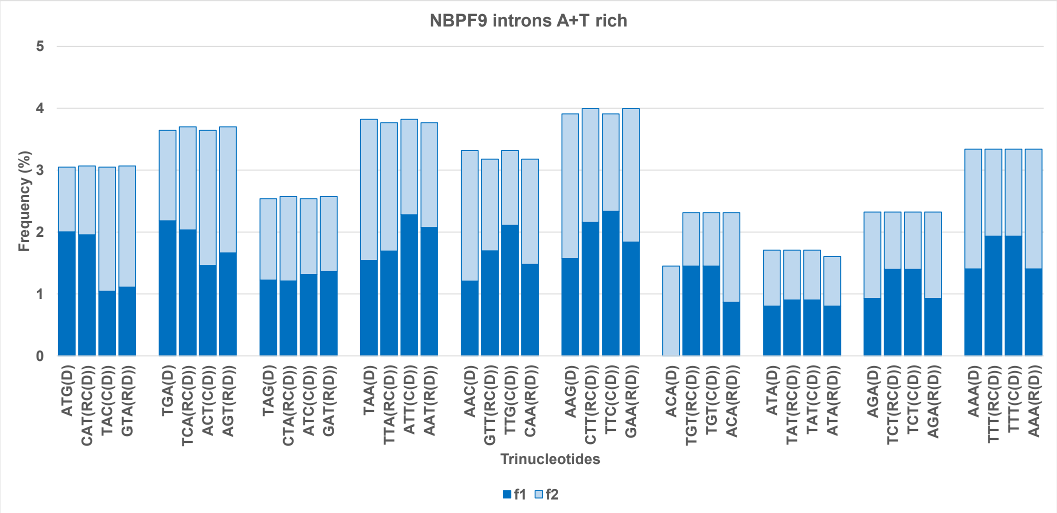

NBPF9 sequence

						C+G rich						
**D**	**f**	**%**	**RC**	**f**	**%**	**C**	**f**	**%**	**R**	**f**	**%**	
**CGT**	177,00	0,35	ACG	143,00	0,28	GCA	708,00	1,40	TGC	716,00	1,41	IIa
**GTC**	657,00	1,30	GAC	529,00	1,04	CAG	1079,00	2,13	CTG	1236,00	2,44	
**GCT**	726,00	1,43	AGC	636,00	1,25	CGA	153,00	0,30	TCG	129,00	0,25	
**GCC**	590,00	1,16	GGC	708,00	1,40	CGG	147,00	0,29	CCG	168,00	0,33	IIb
**CCA**	891,00	1,76	TGG	1056,00	2,08	GGT	606,00	1,19	ACC	628,00	1,24	
**CCT**	1076,00	2,12	AGG	959,00	1,89	GGA	848,00	1,67	TCC	917,00	1,81	
**CAC**	715,00	1,41	GTG	989,00	1,95	GTG*			CAC*			IIc
**CGC**	127,00	0,25	GCG	164,00	0,32	GCG*			CGC*			
**CTC**	1005,00	1,98	GAG	886,00	1,75	GAG*			CTC*			
**CCC**	697,00	1,37	GGG	759,00	1,50	GGG*			CCC*			

Quadruplet		f1	f2	fu
CGT	CGT(D)	0,35	1,40	1,74
	ACG(RC(D))	0,28	1,41	1,69
	GCA(C(D))	1,40	0,35	1,74
	TGC(R(D))	1,41	0,28	1,69
GTC	GTC(D)	1,30	2,13	3,42
	GAC(RC(D))	1,04	2,44	3,48
	CAG(C(D))	2,13	1,30	3,42
	CTG(R(D))	2,44	1,04	3,48
GCT	GCT(D)	1,43	0,30	1,73
	AGC(RC(D))	1,25	0,25	1,51
	CGA(C(D))	0,30	1,43	1,73
	TCG(R(D))	0,25	1,25	1,51
GCC	GCC(D)	1,16	0,29	1,45
	GGC(RC(D))	1,40	0,33	1,73
	CGG(C(D))	0,29	1,16	1,45
	CCG(R(D))	0,33	1,40	1,73
CCA	CCA(D)	1,76	1,19	2,95
	TGG(RC(D))	2,08	1,24	3,32
	GGT(C(D))	1,19	1,76	2,95
	ACC(R(D))	1,24	2,08	3,32
CCT	CCT(D)	2,12	1,67	3,79
	AGG(RC(D))	1,89	1,81	3,70
	GGA(C(D))	1,67	2,12	3,79
	TCC(R(D))	1,81	1,89	3,70
CAC	CAC(D)	0,70	0,97	1,68
	GTG(RC(D))	0,97	0,70	1,68
	GTG(C(D))	0,97	0,70	1,68
	CAC(R(D))	0,70	0,97	1,68
CGC	CGC(D)	0,13	0,16	0,29
	GCG(RC(D))	0,16	0,13	0,29
	GCG(C(D))	0,16	0,13	0,29
	CGC(R(D))	0,13	0,16	0,29
CTC	CTC(D)	0,99	0,87	1,86
	GAG(RC(D))	0,87	0,99	1,86
	GAG(C(D))	0,87	0,99	1,86
	CTC(R(D))	0,99	0,87	1,86
CCC	CCC(D)	0,69	0,75	1,43
	GGG(RC(D))	0,75	0,69	1,43
	GGG(C(D))	0,75	0,69	1,43
	CCC(R(D))	0,69	0,75	1,43

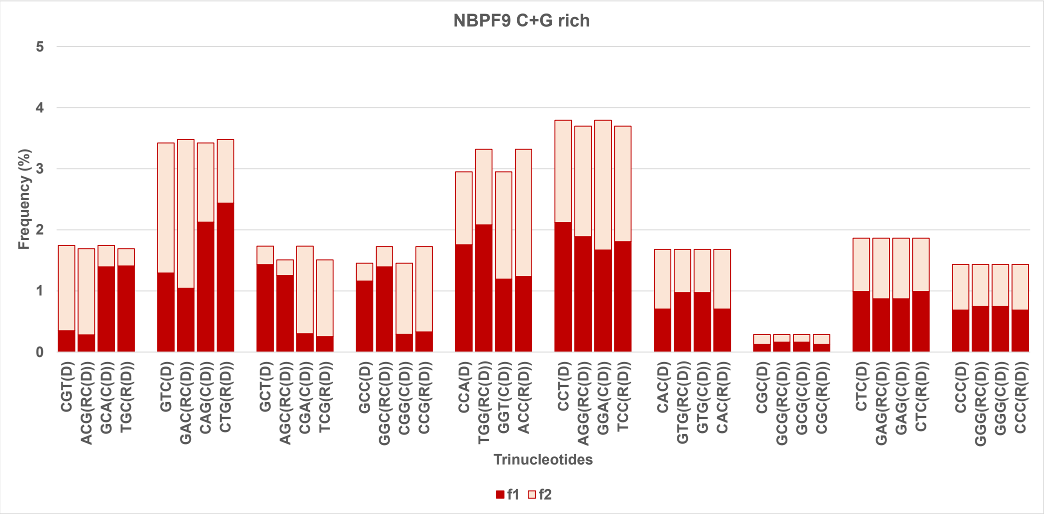

NBPF9 exons

C+G rich

D	f	%	RC	f	%	C	f	%	R	f	%	
**CGT**	24,00	0,41	ACG	35,00	0,60	GCA	87,00	1,49	TGC	88,00	1,51	IIa
**GTC**	72,00	1,23	GAC	88,00	1,51	CAG	175,00	3,00	CTG	150,00	2,57	
**GCT**	102,00	1,75	AGC	117,00	2,01	CGA	26,00	0,45	TCG	13,00	0,22	
**GCC**	98,00	1,68	GGC	93,00	1,59	CGG	35,00	0,60	CCG	26,00	0,45	IIb
**CCA**	118,00	2,02	TGG	127,00	2,18	GGT	65,00	1,11	ACC	71,00	1,22	
**CCT**	120,00	2,06	AGG	155,00	2,66	GGA	160,00	2,74	TCC	95,00	1,63	
**CAC**	75,00	1,29	GTG	76,00	1,30	GTG*			CAC*			IIc
**CGC**	21,00	0,36	GCG	32,00	0,55	GCG*			CGC*			
**CTC**	121,00	2,07	GAG	142,00	2,43	GAG*			CTC*			
**CCC**	82,00	1,41	GGG	84,00	1,44	GGG*			CCC*			

Quadruplet		f1	f2	fu
CGT	CGT(D)	0,41	1,49	1,90
	ACG(RC(D))	0,60	1,51	2,11
	GCA(C(D))	1,49	0,41	1,90
	TGC(R(D))	1,51	0,60	2,11
GTC	GTC(D)	1,23	3,00	4,23
	GAC(RC(D))	1,51	2,57	4,08
	CAG(C(D))	3,00	1,23	4,23
	CTG(R(D))	2,57	1,51	4,08
GCT	GCT(D)	1,75	0,45	2,19
	AGC(RC(D))	2,01	0,22	2,23
	CGA(C(D))	0,45	1,75	2,19
	TCG(R(D))	0,22	2,01	2,23
GCC	GCC(D)	1,68	0,60	2,28
	GGC(RC(D))	1,59	0,45	2,04
	CGG(C(D))	0,60	1,68	2,28
	CCG(R(D))	0,45	1,59	2,04
CCA	CCA(D)	2,02	1,11	3,14
	TGG(RC(D))	2,18	1,22	3,39
	GGT(C(D))	1,11	2,02	3,14
	ACC(R(D))	1,22	2,18	3,39
CCT	CCT(D)	2,06	2,74	4,80
	AGG(RC(D))	2,66	1,63	4,29
	GGA(C(D))	2,74	2,06	4,80
	TCC(R(D))	1,63	2,66	4,29
CAC	CAC(D)	0,64	0,65	1,29
	GTG(RC(D))	0,65	0,64	1,29
	GTG(C(D))	0,65	0,64	1,29
	CAC(R(D))	0,64	0,65	1,29
CGC	CGC(D)	0,18	0,27	0,45
	GCG(RC(D))	0,27	0,18	0,45
	GCG(C(D))	0,27	0,18	0,45
	CGC(R(D))	0,18	0,27	0,45
CTC	CTC(D)	1,04	1,22	2,25
	GAG(RC(D))	1,22	1,04	2,25
	GAG(C(D))	1,22	1,04	2,25
	CTC(R(D))	1,04	1,22	2,25
CCC	CCC(D)	0,70	0,72	1,42
	GGG(RC(D))	0,72	0,70	1,42
	GGG(C(D))	0,72	0,70	1,42
	CCC(R(D))	0,70	0,72	1,42

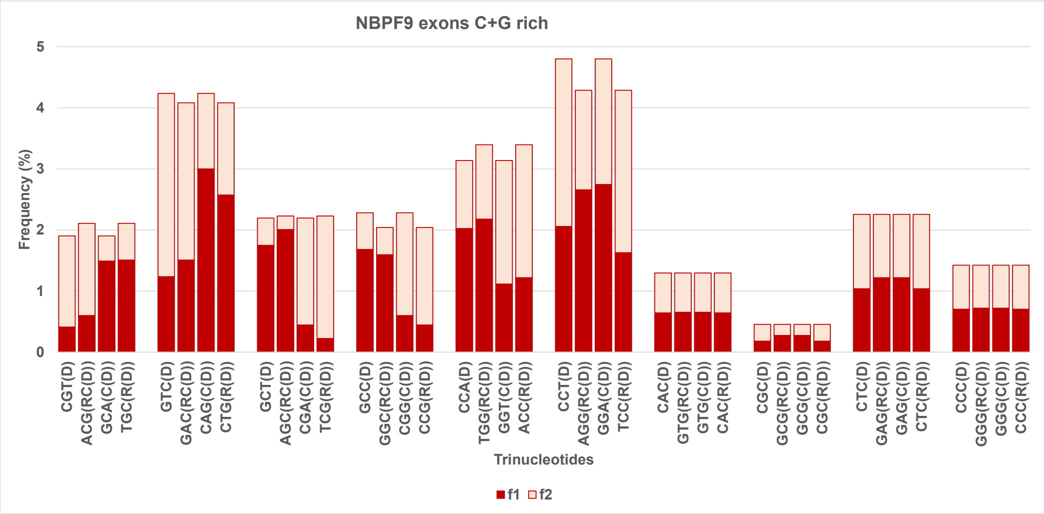

NBPF9 introns

D	f	%	RC	f	%	C	f	%	R	f	%	
						C+G rich						
**CGT**	149,00	0,34	ACG	103,00	0,24	GCA	605,00	1,39	TGC	607,00	1,39	IIa
**GTC**	563,00	1,29	GAC	433,00	0,99	CAG	886,00	2,03	CTG	1057,00	2,42	
**GCT**	604,00	1,38	AGC	506,00	1,16	CGA	121,00	0,28	TCG	107,00	0,24	
**GCC**	472,00	1,08	GGC	595,00	1,36	CGG	97,00	0,22	CCG	130,00	0,30	IIb
**CCA**	761,00	1,74	TGG	906,00	2,07	GGT	525,00	1,20	ACC	544,00	1,25	
**CCT**	927,00	2,12	AGG	790,00	1,81	GGA	673,00	1,54	TCC	802,00	1,84	
**CAC**	624,00	1,43	GTG	878,00	2,01	GTG*			CAC*			IIc
**CGC**	90,00	0,21	GCG	117,00	0,27	GCG*			CGC*			
**CTC**	862,00	1,97	GAG	724,00	1,66	GAG*			CTC*			
**CCC**	595,00	1,36	GGG	646,00	1,48	GGG*			CCC*			

Quadruplet		f1	f2	fu
CGT	CGT(D)	0,34	1,39	1,73
	ACG(RC(D))	0,24	1,39	1,63
	GCA(C(D))	1,39	0,34	1,73
	TGC(R(D))	1,39	0,24	1,63
GTC	GTC(D)	1,29	2,03	3,32
	GAC(RC(D))	0,99	2,42	3,41
	CAG(C(D))	2,03	1,29	3,32
	CTG(R(D))	2,42	0,99	3,41
GCT	GCT(D)	1,38	0,28	1,66
	AGC(RC(D))	1,16	0,24	1,40
	CGA(C(D))	0,28	1,38	1,66
	TCG(R(D))	0,24	1,16	1,40
GCC	GCC(D)	1,08	0,22	1,30
	GGC(RC(D))	1,36	0,30	1,66
	CGG(C(D))	0,22	1,08	1,30
	CCG(R(D))	0,30	1,36	1,66
CCA	CCA(D)	1,74	1,20	2,94
	TGG(RC(D))	2,07	1,25	3,32
	GGT(C(D))	1,20	1,74	2,94
	ACC(R(D))	1,25	2,07	3,32
CCT	CCT(D)	2,12	1,54	3,66
	AGG(RC(D))	1,81	1,84	3,65
	GGA(C(D))	1,54	2,12	3,66
	TCC(R(D))	1,84	1,81	3,65
CAC	CAC(D)	0,71	1,01	1,72
	GTG(RC(D))	1,01	0,71	1,72
	GTG(C(D))	1,01	0,71	1,72
	CAC(R(D))	0,71	1,01	1,72
CGC	CGC(D)	0,10	0,13	0,24
	GCG(RC(D))	0,13	0,10	0,24
	GCG(C(D))	0,13	0,10	0,24
	CGC(R(D))	0,10	0,13	0,24
CTC	CTC(D)	0,99	0,83	1,82
	GAG(RC(D))	0,83	0,99	1,82
	GAG(C(D))	0,83	0,99	1,82
	CTC(R(D))	0,99	0,83	1,82
CCC	CCC(D)	0,68	0,74	1,42
	GGG(RC(D))	0,74	0,68	1,42
	GGG(C(D))	0,74	0,68	1,42
	CCC(R(D))	0,68	0,74	1,42

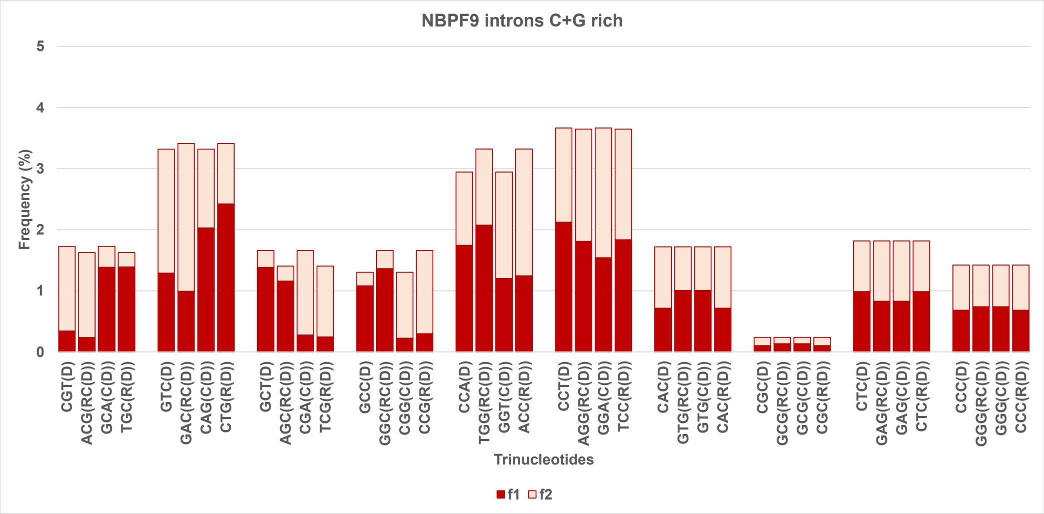

NBPF10 sequence

						A-T rich						
**D**	**f**	**%**	**RC**	**f**	**%**	**C**	**f**	**%**	**R**	**f**	**%**	
**ATG**	1688,00	2,08	CAT	1645,00	2,02	TAC	837,00	1,03	GTA	717,00	0,88	Ia
**TGA**	2146,00	2,64	TCA	1855,00	2,28	ACT	1490,00	1,83	AGT	1234,00	1,52	
**TAG**	903,00	1,11	CTA	1040,00	1,28	ATC	969,00	1,19	GAT	1267,00	1,56	
**TAA**	897,00	1,10	TTA	1257,00	1,55	ATT	1968,00	2,42	AAT	1336,00	1,64	Ib
**AAC**	1057,00	1,30	GTT	1322,00	1,63	TTG	1779,00	2,19	CAA	1120,00	1,38	
**AAG**	1365,00	1,68	CTT	1788,00	2,20	TTC	2041,00	2,51	GAA	1741,00	2,14	
**ACA**	1200,00	1,48	TGT	2589,00	3,18	TGT*			ACA*			Ic
**ATA**	910,00	1,12	TAT	1287,00	1,58	TAT*			ATA*			
**AGA**	1489,00	1,83	TCT	2451,00	3,01	TCT*			AGA*			
**AAA**	1692,00	2,08	TTT	2802,00	3,45	TTT*			AAA*			

Quadruplet		f1	f2	fu
ATG	ATG(D)	2,08	1,03	3,11
	CAT(RC(D))	2,02	0,88	2,91
	TAC(C(D))	1,03	2,08	3,11
	GTA(R(D))	0,88	2,02	2,91
TGA	TGA(D)	2,64	1,83	4,47
	TCA(RC(D))	2,28	1,52	3,80
	ACT(C(D))	1,83	2,64	4,47
	AGT(R(D))	1,52	2,28	3,80
TAG	TAG(D)	1,11	1,19	2,30
	CTA(RC(D))	1,28	1,56	2,84
	ATC(C(D))	1,19	1,11	2,30
	GAT(R(D))	1,56	1,28	2,84
TAA	TAA(D)	1,10	2,42	3,52
	TTA(RC(D))	1,55	1,64	3,19
	ATT(C(D))	2,42	1,10	3,52
	AAT(R(D))	1,64	1,55	3,19
AAC	AAC(D)	1,30	2,19	3,49
	GTT(RC(D))	1,63	1,38	3,00
	TTG(C(D))	2,19	1,30	3,49
	CAA(R(D))	1,38	1,63	3,00
AAG	AAG(D)	1,68	2,51	4,19
	CTT(RC(D))	2,20	2,14	4,34
	TTC(C(D))	2,51	1,68	4,19
	GAA(R(D))	2,14	2,20	4,34
ACA	ACA(D)	0,74	1,59	2,33
	TGT(RC(D))	1,59	0,74	2,33
	TGT(C(D))	1,59	0,74	2,33
	ACA(R(D))	0,74	1,59	2,33
ATA	ATA(D)	0,56	0,79	1,35
	TAT(RC(D))	0,79	0,56	1,35
	TAT(C(D))	0,79	0,56	1,35
	ATA(R(D))	0,56	0,56	1,12
AGA	AGA(D)	0,92	1,51	2,42
	TCT(RC(D))	1,51	0,92	2,42
	TCT(C(D))	1,51	0,92	2,42
	AGA(R(D))	0,92	1,51	2,42
AAA	AAA(D)	1,04	1,72	2,76
	TTT(RC(D))	1,72	1,04	2,76
	TTT(C(D))	1,72	1,04	2,76
	AAA(R(D))	1,04	1,72	2,76

Composition of DNA sequence in NBPF10

Base	No.of bases	%
A	20295	24,96%
C	17613	21,66%
G	18164	22,34%
T	25234	31,04%
N	0	0,00%
Sum	81306	100,00%

% of coding bases from NBPF10 (including N bases)

82,48%

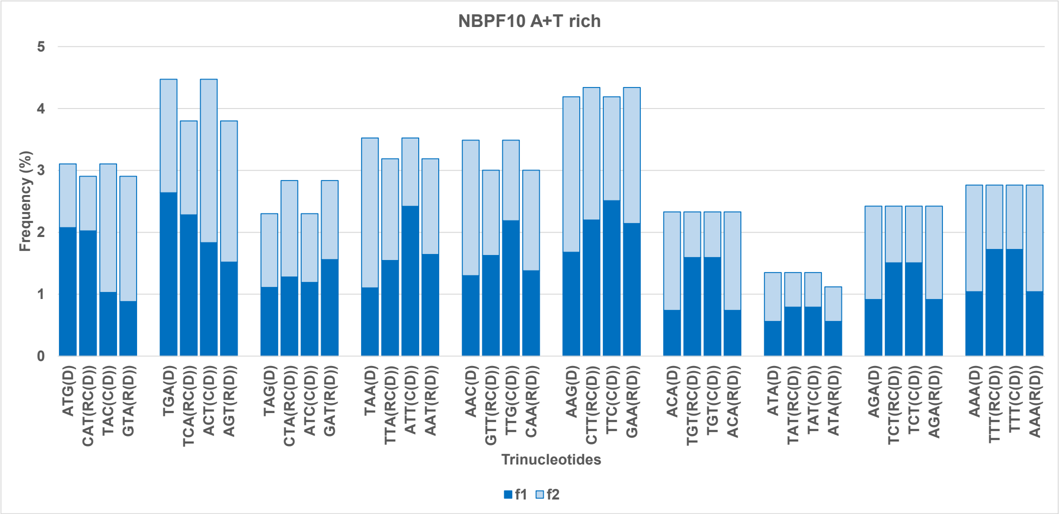

NBPF10 exons

A-T rich

D	f	%	RC	f	%	C	f	%	R	f	%	
**ATG**	272,00	2,09	CAT	197,00	1,51	TAC	97,00	0,74	GTA	91,00	0,70	Ia
**TGA**	317,00	2,43	TCA	298,00	2,29	ACT	256,00	1,96	AGT	204,00	1,56	
**TAG**	112,00	0,86	CTA	67,00	0,51	ATC	100,00	0,77	GAT	182,00	1,40	
**TAA**	46,00	0,35	TTA	167,00	1,28	ATT	185,00	1,42	AAT	108,00	0,83	Ib
**AAC**	172,00	1,32	GTT	193,00	1,48	TTG	325,00	2,49	CAA	194,00	1,49	
**AAG**	569,00	4,36	CTT	276,00	2,12	TTC	162,00	1,24	GAA	609,00	4,67	
**ACA**	185,00	1,42	TGT	196,00	1,50	TGT*			ACA*			Ic
**ATA**	77,00	0,59	TAT	147,00	1,13	TAT*			ATA*			
**AGA**	500,00	3,83	TCT	181,00	1,39	TCT*			AGA*			
**AAA**	380,00	2,91	TTT	176,00	1,35	TTT*			AAA*			

Quadruplet		f1	f2	fu
	ATG(D)	2,09	0,74	2,83
ATG	CAT(RC(D))	1,51	0,70	2,21
	TAC(C(D))	0,74	2,09	2,83
	GTA(R(D))	0,70	1,51	2,21
	TGA(D)	2,43	1,96	4,39
TGA	TCA(RC(D))	2,29	1,56	3,85
	ACT(C(D))	1,96	2,43	4,39
	AGT(R(D))	1,56	2,29	3,85
	TAG(D)	0,86	0,77	1,63
TAG	CTA(RC(D))	0,51	1,40	1,91
	ATC(C(D))	0,77	0,86	1,63
	GAT(R(D))	1,40	0,51	1,91
	TAA(D)	0,35	1,42	1,77
TAA	TTA(RC(D))	1,28	0,83	2,11
	ATT(C(D))	1,42	0,35	1,77
	AAT(R(D))	0,83	1,28	2,11
	AAC(D)	1,32	2,49	3,81
AAC	GTT(RC(D))	1,48	1,49	2,97
	TTG(C(D))	2,49	1,32	3,81
	CAA(R(D))	1,49	1,48	2,97
	AAG(D)	4,36	1,24	5,61
AAG	CTT(RC(D))	2,12	4,67	6,79
	TTC(C(D))	1,24	4,36	5,61
	GAA(R(D))	4,67	2,12	6,79
	ACA(D)	0,71	0,75	1,46
ACA	TGT(RC(D))	0,75	0,71	1,46
	TGT(C(D))	0,75	0,71	1,46
	ACA(R(D))	0,71	0,75	1,46
	ATA(D)	0,30	0,56	0,86
ATA	TAT(RC(D))	0,56	0,30	0,86
	TAT(C(D))	0,56	0,30	0,86
	ATA(R(D))	0,30	0,30	0,59
	AGA(D)	1,92	0,69	2,61
AGA	TCT(RC(D))	0,69	1,92	2,61
	TCT(C(D))	0,69	1,92	2,61
	AGA(R(D))	1,92	0,69	2,61
	AAA(D)	1,46	0,67	2,13
AAA	TTT(RC(D))	0,67	1,46	2,13
	TTT(C(D))	0,67	1,46	2,13
	AAA(R(D))	1,46	0,67	2,13

Composition of DNA sequence in NBPF10 exons

Base	No.of bases	%
A	3884	29,78%
C	2741	21,02%
G	3485	26,72%
T	2932	22,48%
N	0	0,00%
Sum	13042	100,00%

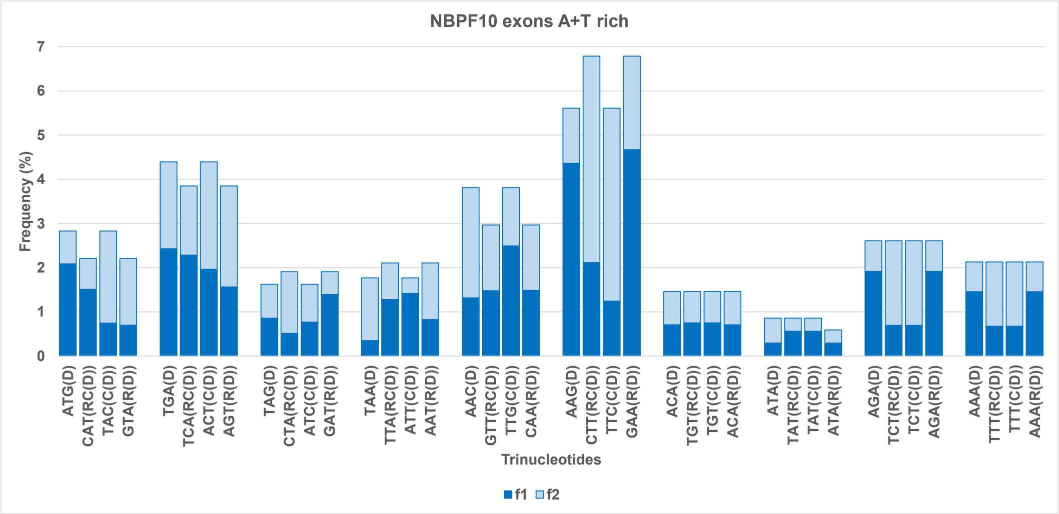

NBPF10 introns

A-T rich

D	f	%	RC	f	%	C	f	%	R	f	%	
**ATG**	1392,00	2,08	CAT	1416,00	2,11	TAC	723,00	1,08	GTA	614,00	0,92	Ia
**TGA**	1809,00	2,70	TCA	1528,00	2,28	ACT	1218,00	1,82	AGT	989,00	1,47	
**TAG**	779,00	1,16	CTA	958,00	1,43	ATC	854,00	1,27	GAT	1069,00	1,59	
**TAA**	827,00	1,23	TTA	1063,00	1,59	ATT	1750,00	2,61	AAT	1205,00	1,80	Ib
**AAC**	870,00	1,30	GTT	1101,00	1,64	TTG	1426,00	2,13	CAA	919,00	1,37	
**AAG**	783,00	1,17	CTT	1488,00	2,22	TTC	1850,00	2,76	GAA	1112,00	1,66	
**ACA**	990,00	1,48	TGT	2347,00	3,50	TGT*			ACA*			Ic
**ATA**	811,00	1,21	TAT	1117,00	1,67	TAT*			ATA*			
**AGA**	968,00	1,44	TCT	2242,00	3,34	TCT*			AGA*			
**AAA**	1296,00	1,93	TTT	2571,00	3,83	TTT*			AAA*			

Quadruplet		f1	f2	fu
ATG	ATG(D)	2,08	1,08	3,15
	CAT(RC(D))	2,11	0,92	3,03
	TAC(C(D))	1,08	2,08	3,15
	GTA(R(D))	0,92	2,11	3,03
TGA	TGA(D)	2,70	1,82	4,51
	TCA(RC(D))	2,28	1,47	3,75
	ACT(C(D))	1,82	2,70	4,51
	AGT(R(D))	1,47	2,28	3,75
TAG	TAG(D)	1,16	1,27	2,44
	CTA(RC(D))	1,43	1,59	3,02
	ATC(C(D))	1,27	1,16	2,44
	GAT(R(D))	1,59	1,43	3,02
TAA	TAA(D)	1,23	2,61	3,84
	TTA(RC(D))	1,59	1,80	3,38
	ATT(C(D))	2,61	1,23	3,84
	AAT(R(D))	1,80	1,59	3,38
AAC	AAC(D)	1,30	2,13	3,42
	GTT(RC(D))	1,64	1,37	3,01
	TTG(C(D))	2,13	1,30	3,42
	CAA(R(D))	1,37	1,64	3,01
AAG	AAG(D)	1,17	2,76	3,93
	CTT(RC(D))	2,22	1,66	3,88
	TTC(C(D))	2,76	1,17	3,93
	GAA(R(D))	1,66	2,22	3,88
ACA	ACA(D)	0,74	1,75	2,49
	TGT(RC(D))	1,75	0,74	2,49
	TGT(C(D))	1,75	0,74	2,49
	ACA(R(D))	0,74	1,75	2,49
ATA	ATA(D)	0,60	0,83	1,44
	TAT(RC(D))	0,83	0,60	1,44
	TAT(C(D))	0,83	0,60	1,44
	ATA(R(D))	0,60	0,60	1,21
AGA	AGA(D)	0,72	1,67	2,39
	TCT(RC(D))	1,67	0,72	2,39
	TCT(C(D))	1,67	0,72	2,39
	AGA(R(D))	0,72	1,67	2,39
AAA	AAA(D)	0,97	1,92	2,88
	TTT(RC(D))	1,92	0,97	2,88
	TTT(C(D))	1,92	0,97	2,88
	AAA(R(D))	0,97	1,92	2,88

Composition of DNA sequence NBPF10 introns

Base	No.of bases	%
A	16115	24,03%
C	14634	21,82%
G	14426	21,51%
T	21889	32,64%
N	1	0,00%
Sum	67065	100,00%

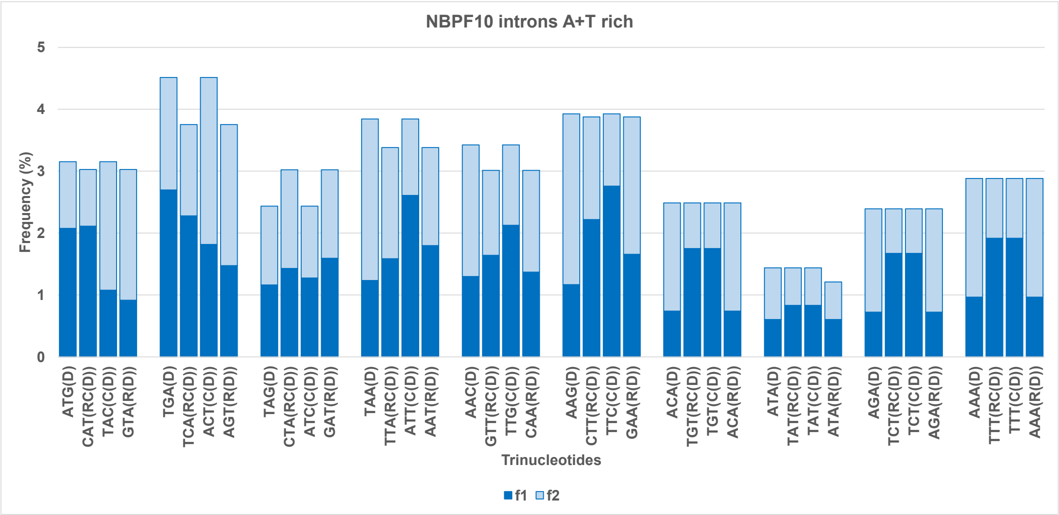

NBPF10 sequence

C+G rich

D	f	%	RC	f	%	C	f	%	R	f	%	
**CGT**	192,00	0,24	ACG	202,00	0,25	GCA	1099,00	1,35	TGC	1002,00	1,23	IIa
**GTC**	1144,00	1,41	GAC	1027,00	1,26	CAG	1770,00	2,18	CTG	2295,00	2,82	
**GCT**	1075,00	1,32	AGC	1025,00	1,26	CGA	244,00	0,30	TCG	156,00	0,19	
**GCC**	914,00	1,12	GGC	1072,00	1,32	CGG	81,00	0,10	CCG	147,00	0,18	IIb
**CCA**	1348,00	1,66	TGG	1698,00	2,09	GGT	841,00	1,03	ACC	996,00	1,23	
**CCT**	1947,00	2,39	AGG	1674,00	2,06	GGA	1540,00	1,89	TCC	1532,00	1,88	
**CAC**	967,00	1,19	GTG	1673,00	2,06	GTG*			CAC*			IIc
**CGC**	141,00	0,17	GCG	153,00	0,19	GCG*			CGC*			
**CTC**	1840,00	2,26	GAG	1384,00	1,70	GAG*			CTC*			
**CCC**	1048,00	1,29	GGG	1195,00	1,47	GGG*			CCC*			

Quadruplet		f1	f2	fu
	CGT(D)	0,24	1,35	1,59
CGT	ACG(RC(D))	0,25	1,23	1,48
	GCA(C(D))	1,35	0,24	1,59
	TGC(R(D))	1,23	0,25	1,48
	GTC(D)	1,41	2,18	3,58
GTC	GAC(RC(D))	1,26	2,82	4,09
	CAG(C(D))	2,18	1,41	3,58
	CTG(R(D))	2,82	1,26	4,09
	GCT(D)	1,32	0,30	1,62
GCT	AGC(RC(D))	1,26	0,19	1,45
	CGA(C(D))	0,30	1,32	1,62
	TCG(R(D))	0,19	1,26	1,45
	GCC(D)	1,12	0,10	1,22
GCC	GGC(RC(D))	1,32	0,18	1,50
	CGG(C(D))	0,10	1,12	1,22
	CCG(R(D))	0,18	1,32	1,50
	CCA(D)	1,66	1,03	2,69
CCA	TGG(RC(D))	2,09	1,23	3,31
	GGT(C(D))	1,03	1,66	2,69
	ACC(R(D))	1,23	2,09	3,31
	CCT(D)	2,39	1,89	4,29
CCT	AGG(RC(D))	2,06	1,88	3,94
	GGA(C(D))	1,89	2,39	4,29
	TCC(R(D))	1,88	2,06	3,94
	CAC(D)	0,59	1,03	1,62
CAC	GTG(RC(D))	1,03	0,59	1,62
	GTG(C(D))	1,03	0,59	1,62
	CAC(R(D))	0,59	1,03	1,62
	CGC(D)	0,09	0,09	0,18
CGC	GCG(RC(D))	0,09	0,09	0,18
	GCG(C(D))	0,09	0,09	0,18
	CGC(R(D))	0,09	0,09	0,18
	CTC(D)	1,13	0,85	1,98
CTC	GAG(RC(D))	0,85	1,13	1,98
	GAG(C(D))	0,85	1,13	1,98
	CTC(R(D))	1,13	0,85	1,98
	CCC(D)	0,64	0,73	1,38
CCC	GGG(RC(D))	0,73	0,64	1,38
	GGG(C(D))	0,73	0,64	1,38
	CCC(R(D))	0,64	0,73	1,38

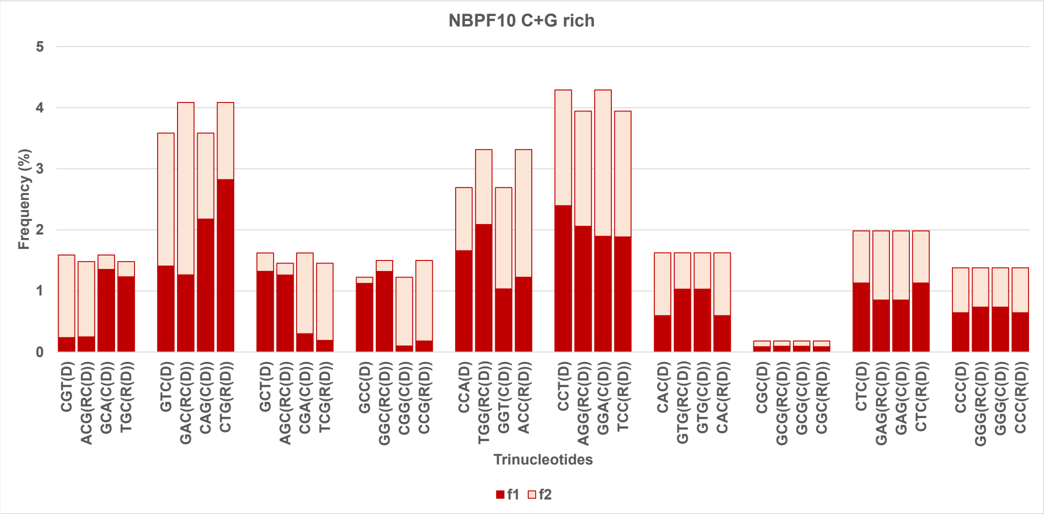

NBPF10 exons

C+G rich												
**D**	**f**	**%**	**RC**	**f**	**%**	**C**	**f**	**%**	**R**	**f**	**%**	
**CGT**	42,00	0,32	ACG	50,00	0,38	GCA	183,00	1,40	TGC	240,00	1,84	IIa
**GTC**	117,00	0,90	GAC	266,00	2,04	CAG	432,00	3,31	CTG	339,00	2,60	
**GCT**	248,00	1,90	AGC	262,00	2,01	CGA	33,00	0,25	TCG	22,00	0,17	
**GCC**	249,00	1,91	GGC	180,00	1,38	CGG	16,00	0,12	CCG	17,00	0,13	IIb
**CCA**	271,00	2,08	TGG	303,00	2,32	GGT	79,00	0,61	ACC	158,00	1,21	
**CCT**	261,00	2,00	AGG	406,00	3,11	GGA	466,00	3,57	TCC	143,00	1,10	
**CAC**	114,00	0,87	GTG	120,00	0,92	GTG*			CAC*			IIc
**CGC**	13,00	0,10	GCG	15,00	0,12	GCG*			CGC*			
**CTC**	264,00	2,02	GAG	259,00	1,99	GAG*			CTC*			
**CCC**	203,00	1,56	GGG	228,00	1,75	GGG*			CCC*			

Quadruplet		f1	f2	fu
CGT	CGT(D)	0,32	1,40	1,73
	ACG(RC(D))	0,38	1,84	2,22
	GCA(C(D))	1,40	0,32	1,73
	TGC(R(D))	1,84	0,38	2,22
GTC	GTC(D)	0,90	3,31	4,21
	GAC(RC(D))	2,04	2,60	4,64
	CAG(C(D))	3,31	0,90	4,21
	CTG(R(D))	2,60	2,04	4,64
GCT	GCT(D)	1,90	0,25	2,15
	AGC(RC(D))	2,01	0,17	2,18
	CGA(C(D))	0,25	1,90	2,15
	TCG(R(D))	0,17	2,01	2,18
GCC	GCC(D)	1,91	0,12	2,03
	GGC(RC(D))	1,38	0,13	1,51
	CGG(C(D))	0,12	1,91	2,03
	CCG(R(D))	0,13	1,38	1,51
CCA	CCA(D)	2,08	0,61	2,68
	TGG(RC(D))	2,32	1,21	3,54
	GGT(C(D))	0,61	2,08	2,68
	ACC(R(D))	1,21	2,32	3,54
CCT	CCT(D)	2,00	3,57	5,58
	AGG(RC(D))	3,11	1,10	4,21
	GGA(C(D))	3,57	2,00	5,58
	TCC(R(D))	1,10	3,11	4,21
CAC	CAC(D)	0,44	0,46	0,90
	GTG(RC(D))	0,46	0,44	0,90
	GTG(C(D))	0,46	0,44	0,90
	CAC(R(D))	0,44	0,46	0,90
CGC	CGC(D)	0,05	0,06	0,11
	GCG(RC(D))	0,06	0,05	0,11
	GCG(C(D))	0,06	0,05	0,11
	CGC(R(D))	0,05	0,06	0,11
CTC	CTC(D)	1,01	0,99	2,01
	GAG(RC(D))	0,99	1,01	2,01
	GAG(C(D))	0,99	1,01	2,01
	CTC(R(D))	1,01	0,99	2,01
CCC	CCC(D)	0,78	0,87	1,65
	GGG(RC(D))	0,87	0,78	1,65
	GGG(C(D))	0,87	0,78	1,65
	CCC(R(D))	0,78	0,87	1,65

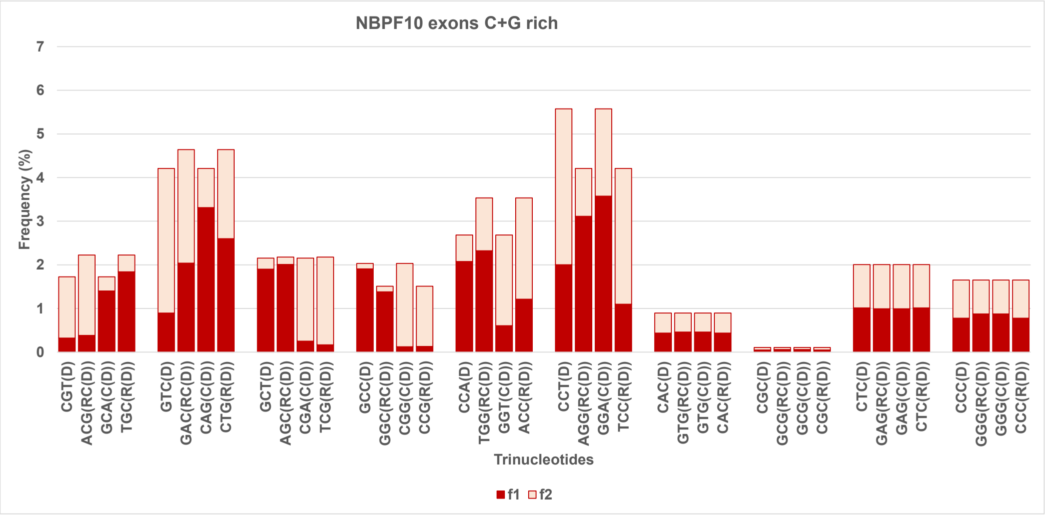

NBPF10 introns

C+G rich

D	f	%	RC	f	%	C	f	%	R	f	%	
**CGT**	149,00	0,22	ACG	146,00	0,22	GCA	900,00	1,34	TGC	747,00	1,11	IIa
**GTC**	1012,00	1,51	GAC	750,00	1,12	CAG	1314,00	1,96	CTG	1930,00	2,88	
**GCT**	812,00	1,21	AGC	751,00	1,12	CGA	207,00	0,31	TCG	128,00	0,19	
**GCC**	656,00	0,98	GGC	885,00	1,32	CGG	61,00	0,09	CCG	128,00	0,19	IIb
**CCA**	1063,00	1,59	TGG	1359,00	2,03	GGT	754,00	1,12	ACC	821,00	1,22	
**CCT**	1659,00	2,47	AGG	1269,00	1,89	GGA	1050,00	1,57	TCC	1373,00	2,05	
**CAC**	832,00	1,24	GTG	1514,00	2,26	GTG*			CAC*			IIc
**CGC**	123,00	0,18	GCG	138,00	0,21	GCG*			CGC*			
**CTC**	1555,00	2,32	GAG	1103,00	1,64	GAG*			CTC*			
**CCC**	832,00	1,24	GGG	954,00	1,42	GGG*			CCC*			

Quadruplet		f1	f2	fu
CGT	CGT(D)	0,22	1,34	1,56
	ACG(RC(D))	0,22	1,11	1,33
	GCA(C(D))	1,34	0,22	1,56
	TGC(R(D))	1,11	0,22	1,33
GTC	GTC(D)	1,51	1,96	3,47
	GAC(RC(D))	1,12	2,88	4,00
	CAG(C(D))	1,96	1,51	3,47
	CTG(R(D))	2,88	1,12	4,00
GCT	GCT(D)	1,21	0,31	1,52
	AGC(RC(D))	1,12	0,19	1,31
	CGA(C(D))	0,31	1,21	1,52
	TCG(R(D))	0,19	1,12	1,31
GCC	GCC(D)	0,98	0,09	1,07
	GGC(RC(D)	1,32	0,19	1,51
	CGG(C(D))	0,09	0,98	1,07
	CCG(R(D))	0,19	1,32	1,51
CCA	CCA(D)	1,59	1,12	2,71
	TGG(RC(D))	2,03	1,22	3,25
	GGT(C(D))	1,12	1,59	2,71
	ACC(R(D))	1,22	2,03	3,25
CCT	CCT(D)	2,47	1,57	4,04
	AGG(RC(D))	1,89	2,05	3,94
	GGA(C(D))	1,57	2,47	4,04
	TCC(R(D))	2,05	1,89	3,94
CAC	CAC(D)	0,62	1,13	1,75
	GTG(RC(D))	1,13	0,62	1,75
	GTG(C(D))	1,13	0,62	1,75
	CAC(R(D))	0,62	1,13	1,75
CGC	CGC(D)	0,09	0,10	0,19
	GCG(RC(D)	0,10	0,09	0,19
	GCG(C(D))	0,10	0,09	0,19
	CGC(R(D))	0,09	0,10	0,19
CTC	CTC(D)	1,16	0,82	1,98
	GAG(RC(D))	0,82	1,16	1,98
	GAG(C(D))	0,82	1,16	1,98
	CTC(R(D))	1,16	0,82	1,98
CCC	CCC(D)	0,62	0,71	1,33
	GGG(RC(D)	0,71	0,62	1,33
	GGG(C(D))	0,71	0,62	1,33
	CCC(R(D))	0,62	0,71	1,33

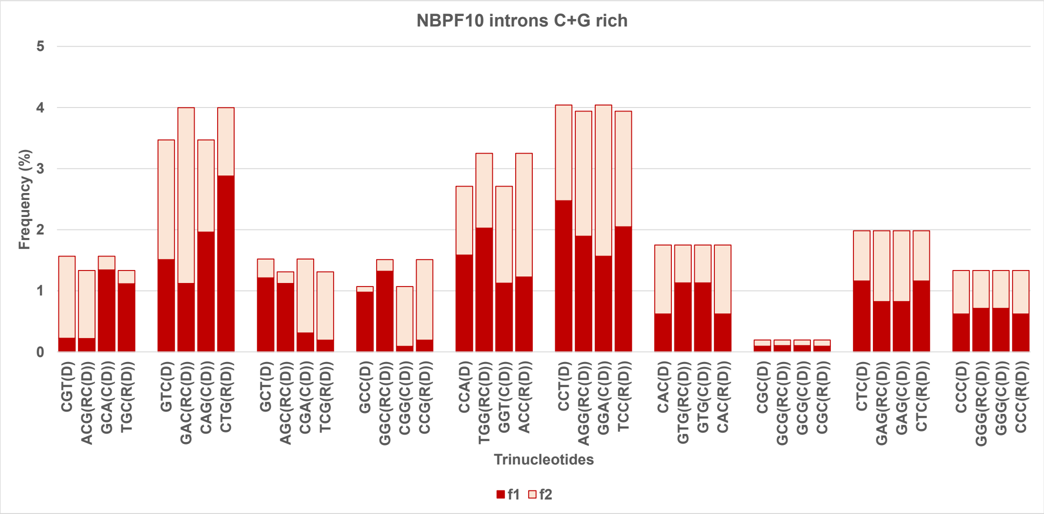

NBPF11 sequence

						A-T rich						
**D**	**f**	**%**	**RC**	**f**	**%**	**C**	**f**	**%**	**R**	**f**	**%**	
**ATG**	985,00	1,91	CAT	940,00	1,83	TAC	483,00	0,94	GTA	531,00	1,03	Ia
**TGA**	1050,00	2,04	TCA	1029,00	2,00	ACT	719,00	1,40	AGT	838,00	1,63	
**TAG**	599,00	1,16	CTA	549,00	1,07	ATC	638,00	1,24	GAT	662,00	1,29	
**TAA**	721,00	1,40	TTA	787,00	1,53	ATT	1033,00	2,01	AAT	989,00	1,92	Ib
**AAC**	597,00	1,16	GTT	799,00	1,55	TTG	1009,00	1,96	CAA	756,00	1,47	
**AAG**	873,00	1,70	CTT	1025,00	1,99	TTC	1061,00	2,06	GAA	982,00	1,91	
**ACA**	865,00	1,68	TGT	1322,00	2,57	TGT*			ACA*			Ic
**ATA**	768,00	1,49	TAT	832,00	1,62	TAT*			ATA*			
**AGA**	998,00	1,94	TCT	1302,00	2,53	TCT*			AGA*			
**AAA**	1417,00	2,75	TTT	1748,00	3,40	TTT*			AAA*			

Quadruplet		f1	f2	fu
ATG	ATG(D)	1,91	0,94	2,85
	CAT(RC(D))	1,83	1,03	2,86
	TAC(C(D))	0,94	1,91	2,85
	GTA(R(D))	1,03	1,83	2,86
TGA	TGA(D)	2,04	1,40	3,44
	TCA(RC(D))	2,00	1,63	3,63
	ACT(C(D))	1,40	2,04	3,44
	AGT(R(D))	1,63	2,00	3,63
TAG	TAG(D)	1,16	1,24	2,40
	CTA(RC(D))	1,07	1,29	2,35
	ATC(C(D))	1,24	1,16	2,40
	GAT(R(D))	1,29	1,07	2,35
TAA	TAA(D)	1,40	2,01	3,41
	TTA(RC(D))	1,53	1,92	3,45
	ATT(C(D))	2,01	1,40	3,41
	AAT(R(D))	1,92	1,53	3,45
AAC	AAC(D)	1,16	1,96	3,12
	GTT(RC(D))	1,55	1,47	3,02
	TTG(C(D))	1,96	1,16	3,12
	CAA(R(D))	1,47	1,55	3,02
AAG	AAG(D)	1,70	2,06	3,76
	CTT(RC(D))	1,99	1,91	3,90
	TTC(C(D))	2,06	1,70	3,76
	GAA(R(D))	1,91	1,99	3,90
ACA	ACA(D)	0,84	1,28	2,12
	TGT(RC(D))	1,28	0,84	2,12
	TGT(C(D))	1,28	0,84	2,12
	ACA(R(D))	0,84	1,28	2,12
ATA	ATA(D)	0,75	0,81	1,55
	TAT(RC(D))	0,81	0,75	1,55
	TAT(C(D))	0,81	0,75	1,55
	ATA(R(D))	0,75	0,75	1,49
AGA	AGA(D)	0,97	1,26	2,23
	TCT(RC(D))	1,26	0,97	2,23
	TCT(C(D))	1,26	0,97	2,23
	AGA(R(D))	0,97	1,26	2,23
AAA	AAA(D)	1,38	1,70	3,07
	TTT(RC(D))	1,70	1,38	3,07
	TTT(C(D))	1,70	1,38	3,07
	AAA(R(D))	1,38	1,70	3,07

Composition of DNA sequence in NBPF11

Base	No.of bases	%
A	13262	25,76%
C	11533	22,40%
G	11761	22,85%
T	14921	28,99%
N	0	0,00%
Sum	51477	100,00%

% of coding bases from NBPF11 (including N bases)

86,79%

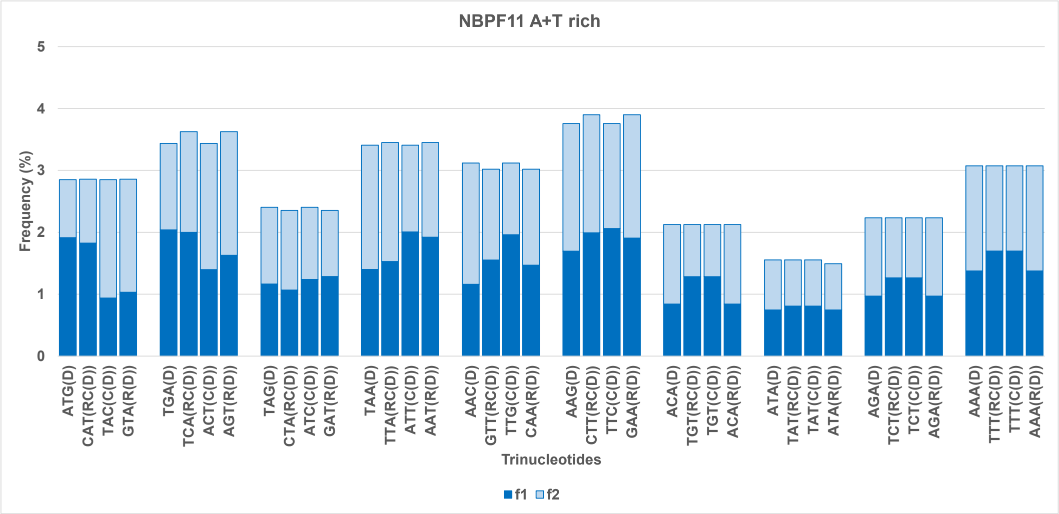

NBPF11 exons

A-T rich

D	f	%	RC	f	%	C	f	%	R	f	%	
**ATG**	95,00	1,73	CAT	90,00	1,64	TAC	41,00	0,75	GTA	37,00	0,67	Ia
**TGA**	124,00	2,26	TCA	134,00	2,44	ACT	78,00	1,42	AGT	92,00	1,68	
**TAG**	47,00	0,86	CTA	35,00	0,64	ATC	64,00	1,17	GAT	68,00	1,24	
**TAA**	45,00	0,82	TTA	65,00	1,18	ATT	80,00	1,46	AAT	77,00	1,40	Ib
**AAC**	73,00	1,33	GTT	73,00	1,33	TTG	96,00	1,75	CAA	104,00	1,89	
**AAG**	158,00	2,88	CTT	85,00	1,55	TTC	77,00	1,40	GAA	159,00	2,90	
**ACA**	95,00	1,73	TGT	90,00	1,64	TGT*			ACA*			Ic
**ATA**	50,00	0,91	TAT	54,00	0,98	TAT*			ATA*			
**AGA**	161,00	2,93	TCT	94,00	1,71	TCT*			AGA*			
**AAA**	134,00	2,44	TTT	98,00	1,78	TTT*			AAA*			

Quadruplet		f1	f2	fu
ATG	ATG(D)	1,73	0,75	2,48
	CAT(RC(D))	1,64	0,67	2,31
	TAC(C(D))	0,75	1,73	2,48
	GTA(R(D))	0,67	1,64	2,31
TGA	TGA(D)	2,26	1,42	3,68
	TCA(RC(D))	2,44	1,68	4,12
	ACT(C(D))	1,42	2,26	3,68
	AGT(R(D))	1,68	2,44	4,12
TAG	TAG(D)	0,86	1,17	2,02
	CTA(RC(D))	0,64	1,24	1,88
	ATC(C(D))	1,17	0,86	2,02
	GAT(R(D))	1,24	0,64	1,88
TAA	TAA(D)	0,82	1,46	2,28
	TTA(RC(D))	1,18	1,40	2,59
	ATT(C(D))	1,46	0,82	2,28
	AAT(R(D))	1,40	1,18	2,59
AAC	AAC(D)	1,33	1,75	3,08
	GTT(RC(D))	1,33	1,89	3,22
	TTG(C(D))	1,75	1,33	3,08
	CAA(R(D))	1,89	1,33	3,22
AAG	AAG(D)	2,88	1,40	4,28
	CTT(RC(D))	1,55	2,90	4,44
	TTC(C(D))	1,40	2,88	4,28
	GAA(R(D))	2,90	1,55	4,44
ACA	ACA(D)	0,86	0,82	1,68
	TGT(RC(D))	0,82	0,86	1,68
	TGT(C(D))	0,82	0,86	1,68
	ACA(R(D))	0,86	0,82	1,68
ATA	ATA(D)	0,46	0,49	0,95
	TAT(RC(D))	0,49	0,46	0,95
	TAT(C(D))	0,49	0,46	0,95
	ATA(R(D))	0,46	0,46	0,91
AGA	AGA(D)	1,47	0,86	2,32
	TCT(RC(D))	0,86	1,47	2,32
	TCT(C(D))	0,86	1,47	2,32
	AGA(R(D))	1,47	0,86	2,32
AAA	AAA(D)	1,22	0,89	2,11
	TTT(RC(D))	0,89	1,22	2,11
	TTT(C(D))	0,89	1,22	2,11
	AAA(R(D))	1,22	0,89	2,11

Composition of DNA sequence in NBPF11 exons

Base	No.of bases	%
A	1513	27,54%
C	1273	23,17%
G	1436	26,14%
T	1272	23,15%
N	0	0,00%
Sum	5494	100,00%

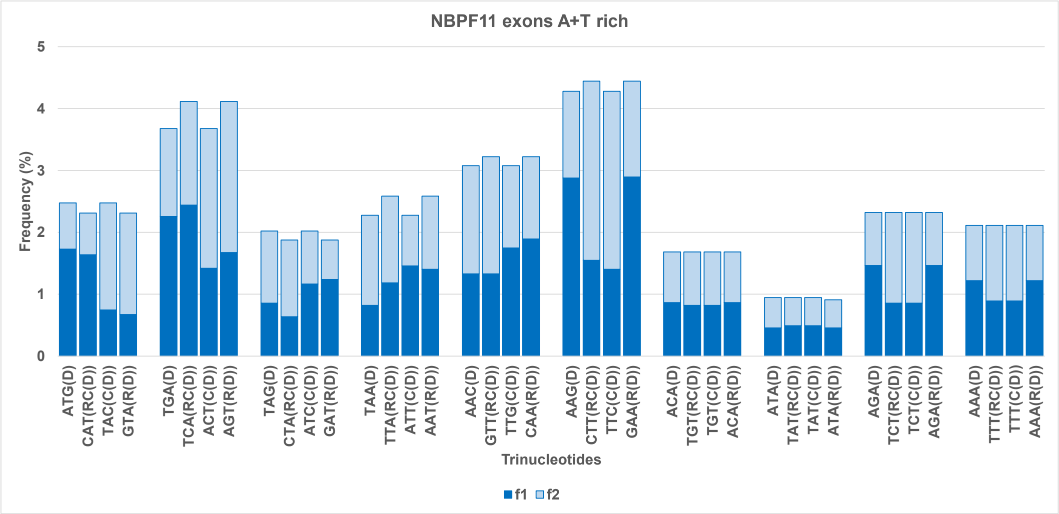

NBPF11 introns

A-T rich

D	f	%	RC	f	%	C	f	%	R	f	%	
**ATG**	864,00	1,93	CAT	820,00	1,84	TAC	427,00	0,96	GTA	475,00	1,06	Ia
**TGA**	904,00	2,02	TCA	868,00	1,94	ACT	623,00	1,39	AGT	721,00	1,61	
**TAG**	525,00	1,18	CTA	499,00	1,12	ATC	558,00	1,25	GAT	573,00	1,28	
**TAA**	646,00	1,45	TTA	683,00	1,53	ATT	908,00	2,03	AAT	878,00	1,97	Ib
**AAC**	513,00	1,15	GTT	698,00	1,56	TTG	882,00	1,97	CAA	641,00	1,43	
**AAG**	699,00	1,56	CTT	913,00	2,04	TTC	954,00	2,14	GAA	803,00	1,80	
**ACA**	752,00	1,68	TGT	1191,00	2,67	TGT*			ACA*			Ic
**ATA**	687,00	1,54	TAT	746,00	1,67	TAT*			ATA*			
**AGA**	819,00	1,83	TCT	1181,00	2,64	TCT*			AGA*			
**AAA**	1241,00	2,78	TTT	1576,00	3,53	TTT*			AAA*			

Quadruplet		f1	f2	fu
ATG	ATG(D)	1,93	0,96	2,89
	CAT(RC(D))	1,84	1,06	2,90
	TAC(C(D))	0,96	1,93	2,89
	GTA(R(D))	1,06	1,84	2,90
TGA	TGA(D)	2,02	1,39	3,42
	TCA(RC(D))	1,94	1,61	3,56
	ACT(C(D))	1,39	2,02	3,42
	AGT(R(D))	1,61	1,94	3,56
TAG	TAG(D)	1,18	1,25	2,42
	CTA(RC(D))	1,12	1,28	2,40
	ATC(C(D))	1,25	1,18	2,42
	GAT(R(D))	1,28	1,12	2,40
TAA	TAA(D)	1,45	2,03	3,48
	TTA(RC(D))	1,53	1,97	3,49
	ATT(C(D))	2,03	1,45	3,48
	AAT(R(D))	1,97	1,53	3,49
AAC	AAC(D)	1,15	1,97	3,12
	GTT(RC(D))	1,56	1,43	3,00
	TTG(C(D))	1,97	1,15	3,12
	CAA(R(D))	1,43	1,56	3,00
AAG	AAG(D)	1,56	2,14	3,70
	CTT(RC(D))	2,04	1,80	3,84
	TTC(C(D))	2,14	1,56	3,70
	GAA(R(D))	1,80	2,04	3,84
ACA	ACA(D)	0,84	1,33	2,17
	TGT(RC(D))	1,33	0,84	2,17
	TGT(C(D))	1,33	0,84	2,17
	ACA(R(D))	0,84	1,33	2,17
ATA	ATA(D)	0,77	0,83	1,60
	TAT(RC(D))	0,83	0,77	1,60
	TAT(C(D))	0,83	0,77	1,60
	ATA(R(D))	0,77	0,77	1,54
AGA	AGA(D)	0,92	1,32	2,24
	TCT(RC(D))	1,32	0,92	2,24
	TCT(C(D))	1,32	0,92	2,24
	AGA(R(D))	0,92	1,32	2,24
AAA	AAA(D)	1,39	1,76	3,15
	TTT(RC(D))	1,76	1,39	3,15
	TTT(C(D))	1,76	1,39	3,15
	AAA(R(D))	1,39	1,76	3,15

Composition of DNA sequence NBPF11 introns

Base	No.of bases	%
A	11401	25,52%
C	10038	22,47%
G	10052	22,50%
T	13187	29,52%
N	0	0,00%
Sum	44678	100,00%

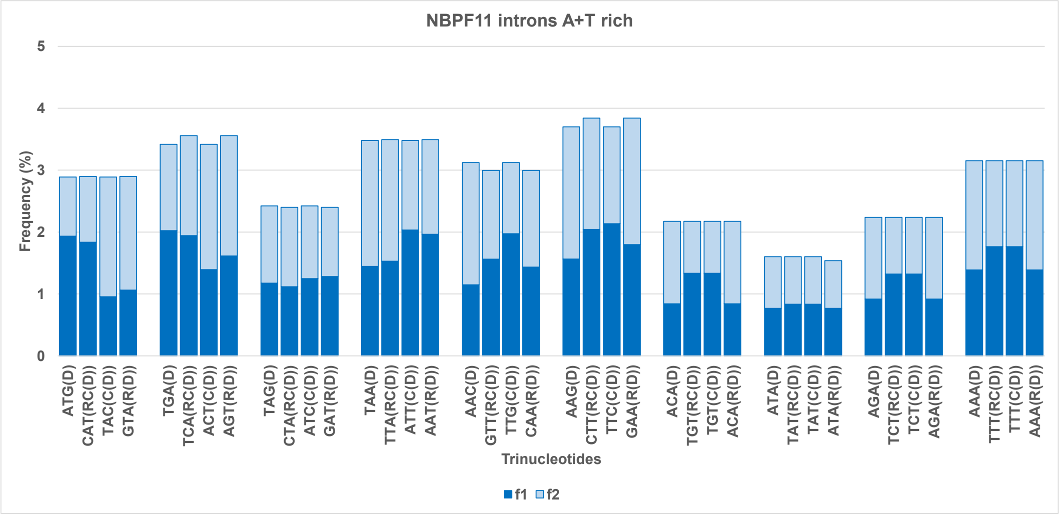

NBPF11 sequence

D	f	%	RC	f	%	C	f	%	R	f	%	
						C+G rich						
**CGT**	195,00	0,38	ACG	163,00	0,32	GCA	777,00	1,51	TGC	799,00	1,55	IIa
**GTC**	656,00	1,27	GAC	532,00	1,03	CAG	1186,00	2,30	CTG	1246,00	2,42	
**GCT**	775,00	1,51	AGC	712,00	1,38	CGA	175,00	0,34	TCG	153,00	0,30	
**GCC**	742,00	1,44	GGC	820,00	1,59	CGG	225,00	0,44	CCG	242,00	0,47	IIb
**CCA**	982,00	1,91	TGG	1076,00	2,09	GGT	638,00	1,24	ACC	635,00	1,23	
**CCT**	1103,00	2,14	AGG	1031,00	2,00	GGA	874,00	1,70	TCC	950,00	1,85	
**CAC**	770,00	1,50	GTG	1007,00	1,96	GTG*			CAC*			IIc
**CGC**	189,00	0,37	GCG	226,00	0,44	GCG*			CGC*			
**CTC**	1079,00	2,10	GAG	921,00	1,79	GAG*			CTC*			
**CCC**	870,00	1,69	GGG	819,00	1,59	GGG*			CCC*			

Quadruplet		f1	f2	fu
CGT	CGT(D)	0,38	1,51	1,89
	ACG(RC(D))	0,32	1,55	1,87
	GCA(C(D))	1,51	0,38	1,89
	TGC(R(D))	1,55	0,32	1,87
GTC	GTC(D)	1,27	2,30	3,58
	GAC(RC(D))	1,03	2,42	3,45
	CAG(C(D))	2,30	1,27	3,58
	CTG(R(D))	2,42	1,03	3,45
GCT	GCT(D)	1,51	0,34	1,85
	AGC(RC(D))	1,38	0,30	1,68
	CGA(C(D))	0,34	1,51	1,85
	TCG(R(D))	0,30	1,38	1,68
GCC	GCC(D)	1,44	0,44	1,88
	GGC(RC(D))	1,59	0,47	2,06
	CGG(C(D))	0,44	1,44	1,88
	CCG(R(D))	0,47	1,59	2,06
CCA	CCA(D)	1,91	1,24	3,15
	TGG(RC(D))	2,09	1,23	3,32
	GGT(C(D))	1,24	1,91	3,15
	ACC(R(D))	1,23	2,09	3,32
CCT	CCT(D)	2,14	1,70	3,84
	AGG(RC(D))	2,00	1,85	3,85
	GGA(C(D))	1,70	2,14	3,84
	TCC(R(D))	1,85	2,00	3,85
CAC	CAC(D)	0,75	0,98	1,73
	GTG(RC(D))	0,98	0,75	1,73
	GTG(C(D))	0,98	0,75	1,73
	CAC(R(D))	0,75	0,98	1,73
CGC	CGC(D)	0,18	0,22	0,40
	GCG(RC(D))	0,22	0,18	0,40
	GCG(C(D))	0,22	0,18	0,40
	CGC(R(D))	0,18	0,22	0,40
CTC	CTC(D)	1,05	0,89	1,94
	GAG(RC(D))	0,89	1,05	1,94
	GAG(C(D))	0,89	1,05	1,94
	CTC(R(D))	1,05	0,89	1,94
CCC	CCC(D)	0,85	0,80	1,64
	GGG(RC(D))	0,80	0,85	1,64
	GGG(C(D))	0,80	0,85	1,64
	CCC(R(D))	0,85	0,80	1,64

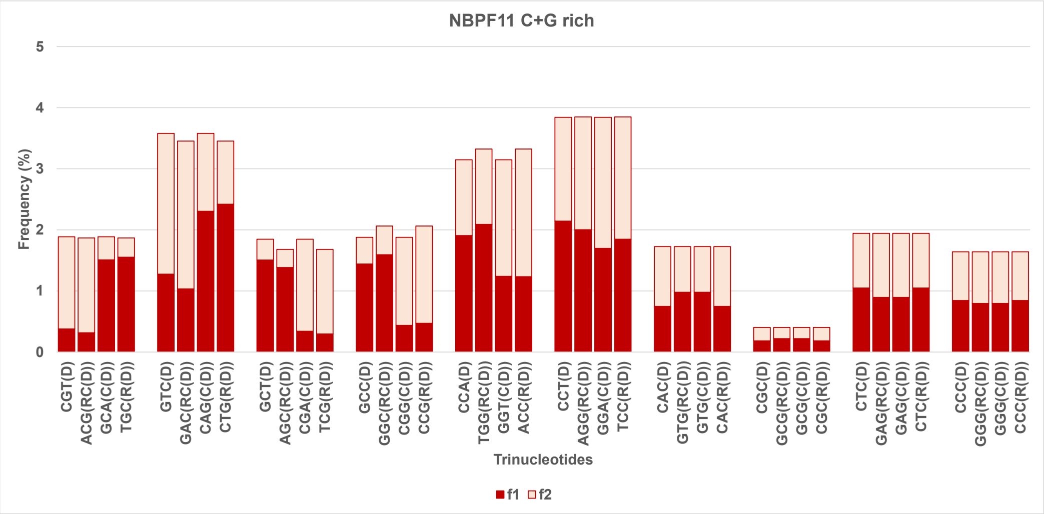

NBPF11 exons

C+G rich

D	f	%	RC	f	%	C	f	%	R	f	%	
**CGT**	15,00	0,27	ACG	28,00	0,51	GCA	91,00	1,66	TGC	88,00	1,60	IIa
**GTC**	74,00	1,35	GAC	81,00	1,47	CAG	167,00	3,04	CTG	142,00	2,59	
**GCT**	95,00	1,73	AGC	118,00	2,15	CGA	27,00	0,49	TCG	10,00	0,18	
**GCC**	101,00	1,84	GGC	96,00	1,75	CGG	47,00	0,86	CCG	36,00	0,66	IIb
**CCA**	110,00	2,00	TGG	110,00	2,00	GGT	66,00	1,20	ACC	62,00	1,13	
**CCT**	117,00	2,13	AGG	147,00	2,68	GGA	142,00	2,59	TCC	99,00	1,80	
**CAC**	68,00	1,24	GTG	79,00	1,44	GTG*			CAC*			IIc
**CGC**	22,00	0,40	GCG	37,00	0,67	GCG*			CGC*			
**CTC**	122,00	2,22	GAG	146,00	2,66	GAG*			CTC*			
**CCC**	85,00	1,55	GGG	91,00	1,66	GGG*			CCC*			

Quadruplet		f1	f2	fu
CGT	CGT(D)	0,27	1,66	1,93
	ACG(RC(D))	0,51	1,60	2,11
	GCA(C(D))	1,66	0,27	1,93
	TGC(R(D))	1,60	0,51	2,11
GTC	GTC(D)	1,35	3,04	4,39
	GAC(RC(D))	1,47	2,59	4,06
	CAG(C(D))	3,04	1,35	4,39
	CTG(R(D))	2,59	1,47	4,06
GCT	GCT(D)	1,73	0,49	2,22
	AGC(RC(D))	2,15	0,18	2,33
	CGA(C(D))	0,49	1,73	2,22
	TCG(R(D))	0,18	2,15	2,33
GCC	GCC(D)	1,84	0,86	2,69
	GGC(RC(D))	1,75	0,66	2,40
	CGG(C(D))	0,86	1,84	2,69
	CCG(R(D))	0,66	1,75	2,40
CCA	CCA(D)	2,00	1,20	3,20
	TGG(RC(D))	2,00	1,13	3,13
	GGT(C(D))	1,20	2,00	3,20
	ACC(R(D))	1,13	2,00	3,13
CCT	CCT(D)	2,13	2,59	4,72
	AGG(RC(D))	2,68	1,80	4,48
	GGA(C(D))	2,59	2,13	4,72
	TCC(R(D))	1,80	2,68	4,48
CAC	CAC(D)	0,62	0,72	1,34
	GTG(RC(D))	0,72	0,62	1,34
	GTG(C(D))	0,72	0,62	1,34
	CAC(R(D))	0,62	0,72	1,34
CGC	CGC(D)	0,20	0,34	0,54
	GCG(RC(D))	0,34	0,20	0,54
	GCG(C(D))	0,34	0,20	0,54
	CGC(R(D))	0,20	0,34	0,54
CTC	CTC(D)	1,11	1,33	2,44
	GAG(RC(D))	1,33	1,11	2,44
	GAG(C(D))	1,33	1,11	2,44
	CTC(R(D))	1,11	1,33	2,44
CCC	CCC(D)	0,77	0,83	1,60
	GGG(RC(D))	0,83	0,77	1,60
	GGG(C(D))	0,83	0,77	1,60
	CCC(R(D))	0,77	0,83	1,60

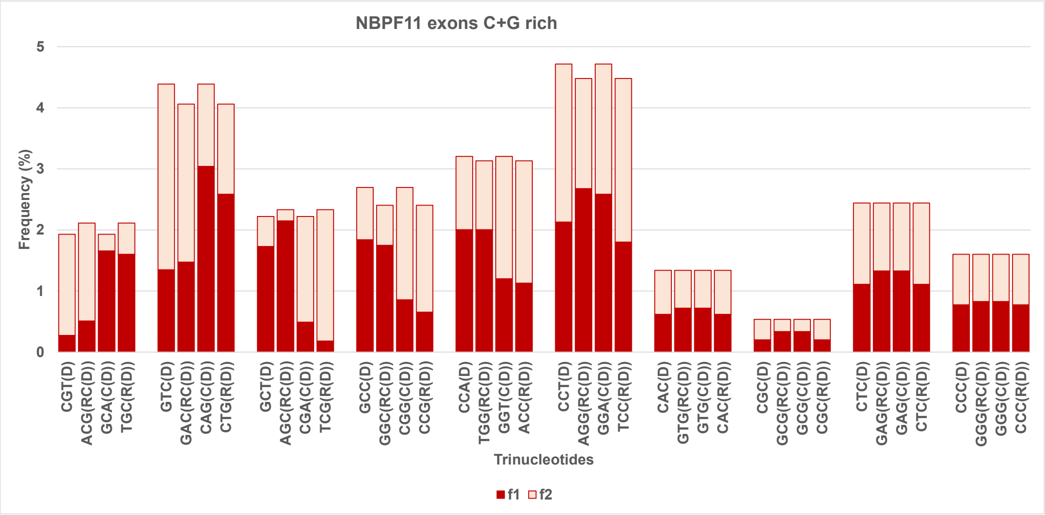

NBPF11 introns

C+G rich

D	f	%	RC	f	%	C	f	%	R	f	%	
**CGT**	176,00	0,39	ACG	129,00	0,29	GCA	676,00	1,51	TGC	693,00	1,55	IIa
**GTC**	570,00	1,28	GAC	439,00	0,98	CAG	1005,00	2,25	CTG	1085,00	2,43	
**GCT**	667,00	1,49	AGC	584,00	1,31	CGA	140,00	0,31	TCG	139,00	0,31	
**GCC**	629,00	1,41	GGC	714,00	1,60	CGG	173,00	0,39	CCG	202,00	0,45	IIb
**CCA**	857,00	1,92	TGG	939,00	2,10	GGT	550,00	1,23	ACC	562,00	1,26	
**CCT**	965,00	2,16	AGG	862,00	1,93	GGA	710,00	1,59	TCC	833,00	1,86	
**CAC**	687,00	1,54	GTG	896,00	2,01	GTG*			CAC*			IIc
**CGC**	162,00	0,36	GCG	181,00	0,41	GCG*			CGC*			
**CTC**	939,00	2,10	GAG	758,00	1,70	GAG*			CTC*			
**CCC**	774,00	1,73	GGG	712,00	1,59	GGG*			CCC*			

Quadruplet		f1	f2	fu
CGT	CGT(D)	0,39	1,51	1,91
	ACG(RC(D))	0,29	1,55	1,84
	GCA(C(D))	1,51	0,39	1,91
	TGC(R(D))	1,55	0,29	1,84
GTC	GTC(D)	1,28	2,25	3,53
	GAC(RC(D))	0,98	2,43	3,41
	CAG(C(D))	2,25	1,28	3,53
	CTG(R(D))	2,43	0,98	3,41
GCT	GCT(D)	1,49	0,31	1,81
	AGC(RC(D))	1,31	0,31	1,62
	CGA(C(D))	0,31	1,49	1,81
	TCG(R(D))	0,31	1,31	1,62
GCC	GCC(D)	1,41	0,39	1,80
	GGC(RC(D))	1,60	0,45	2,05
	CGG(C(D))	0,39	1,41	1,80
	CCG(R(D))	0,45	1,60	2,05
CCA	CCA(D)	1,92	1,23	3,15
	TGG(RC(D))	2,10	1,26	3,36
	GGT(C(D))	1,23	1,92	3,15
	ACC(R(D))	1,26	2,10	3,36
CCT	CCT(D)	2,16	1,59	3,75
	AGG(RC(D))	1,93	1,86	3,79
	GGA(C(D))	1,59	2,16	3,75
	TCC(R(D))	1,86	1,93	3,79
CAC	CAC(D)	0,77	1,00	1,77
	GTG(RC(D))	1,00	0,77	1,77
	GTG(C(D))	1,00	0,77	1,77
	CAC(R(D))	0,77	1,00	1,77
CGC	CGC(D)	0,18	0,20	0,38
	GCG(RC(D))	0,20	0,18	0,38
	GCG(C(D))	0,20	0,18	0,38
	CGC(R(D))	0,18	0,20	0,38
CTC	CTC(D)	1,05	0,85	1,90
	GAG(RC(D))	0,85	1,05	1,90
	GAG(C(D))	0,85	1,05	1,90
	CTC(R(D))	1,05	0,85	1,90
CCC	CCC(D)	0,87	0,80	1,66
	GGG(RC(D))	0,80	0,87	1,66
	GGG(C(D))	0,80	0,87	1,66
	CCC(R(D))	0,87	0,80	1,66

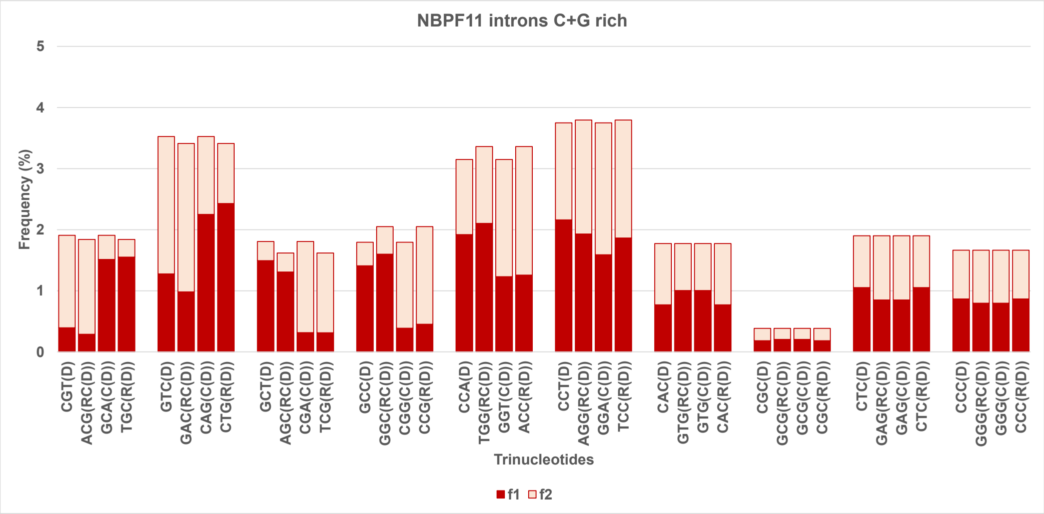

					A-T rich							
**D**	**f**	**%**	**RC**	**f**	**%**	**C**	**f**	**%**	**R**	**f**	**%**	
**ATG**	1147,00	1,96	CAT	1131,00	1,93	TAC	587,00	1,00	GTA	596,00	1,02	Ia
**TGA**	1298,00	2,21	TCA	1225,00	2,09	ACT	893,00	1,52	AGT	977,00	1,67	
**TAG**	674,00	1,15	CTA	669,00	1,14	ATC	760,00	1,30	GAT	770,00	1,31	
**TAA**	836,00	1,43	TTA	902,00	1,54	ATT	1216,00	2,07	AAT	1152,00	1,96	Ib
**AAC**	725,00	1,24	GTT	940,00	1,60	TTG	1206,00	2,06	CAA	875,00	1,49	
**AAG**	1040,00	1,77	CTT	1234,00	2,10	TTC	1282,00	2,19	GAA	1206,00	2,06	
**ACA**	1000,00	1,70	TGT	1580,00	2,69	TGT*			ACA*			Ic
**ATA**	885,00	1,51	TAT	955,00	1,63	TAT*			ATA*			
**AGA**	1211,00	2,06	TCT	1591,00	2,71	TCT*			AGA*			
**AAA**	1689,00	2,88	TTT	1982,00	3,38	TTT*			AAA*			

Quadruplet		f1	f2	fu
ATG	ATG(D)	1,96	1,00	2,96
	CAT(RC(D))	1,93	1,02	2,94
	TAC(C(D))	1,00	1,96	2,96
	GTA(R(D))	1,02	1,93	2,94
TGA	TGA(D)	2,21	1,52	3,74
	TCA(RC(D))	2,09	1,67	3,75
	ACT(C(D))	1,52	2,21	3,74
	AGT(R(D))	1,67	2,09	3,75
TAG	TAG(D)	1,15	1,30	2,44
	CTA(RC(D))	1,14	1,31	2,45
	ATC(C(D))	1,30	1,15	2,44
	GAT(R(D))	1,31	1,14	2,45
TAA	TAA(D)	1,43	2,07	3,50
	TTA(RC(D))	1,54	1,96	3,50
	ATT(C(D))	2,07	1,43	3,50
	AAT(R(D))	1,96	1,54	3,50
AAC	AAC(D)	1,24	2,06	3,29
	GTT(RC(D))	1,60	1,49	3,09
	TTG(C(D))	2,06	1,24	3,29
	CAA(R(D))	1,49	1,60	3,09
AAG	AAG(D)	1,77	2,19	3,96
	CTT(RC(D))	2,10	2,06	4,16
	TTC(C(D))	2,19	1,77	3,96
	GAA(R(D))	2,06	2,10	4,16
ACA	ACA(D)	0,85	1,35	2,20
	TGT(RC(D))	1,35	0,85	2,20
	TGT(C(D))	1,35	0,85	2,20
	ACA(R(D))	0,85	1,35	2,20
ATA	ATA(D)	0,75	0,81	1,57
	TAT(RC(D))	0,81	0,75	1,57
	TAT(C(D))	0,81	0,75	1,57
	ATA(R(D))	0,75	0,75	1,51
AGA	AGA(D)	1,03	1,36	2,39
	TCT(RC(D))	1,36	1,03	2,39
	TCT(C(D))	1,36	1,03	2,39
	AGA(R(D))	1,03	1,36	2,39
AAA	AAA(D)	1,44	1,69	3,13
	TTT(RC(D))	1,69	1,44	3,13
	TTT(C(D))	1,69	1,44	3,13
	AAA(R(D))	1,44	1,69	3,13

Composition of DNA sequence in NBPF12

Base	No.of bases	%
A	15465	26,364%
C	12717	21,680%
G	13082	22,302%
T	17395	29,654%
N	0	0,000%
Sum	58659	100,00%

% of coding bases from NBPF12 (including N bases)

**85,92%**

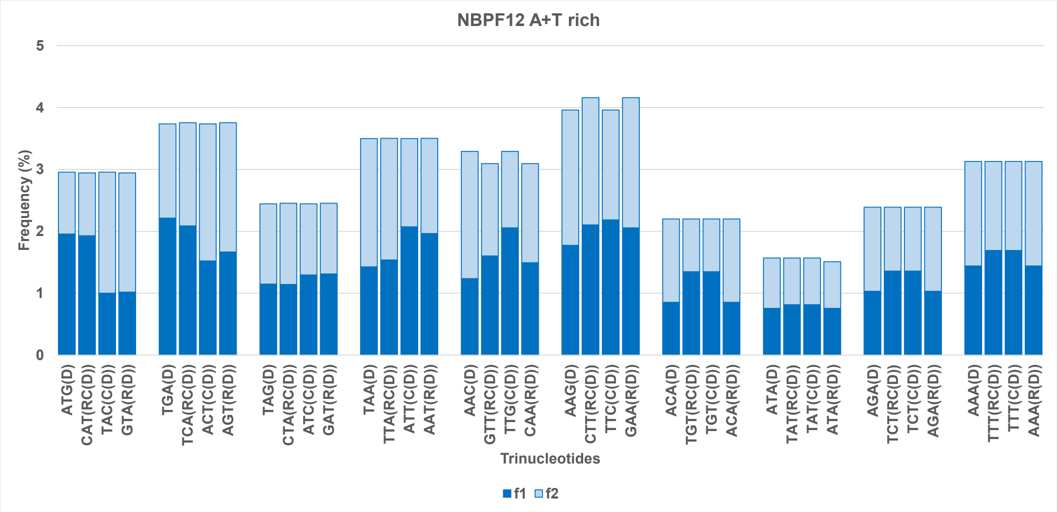

NBPF12 exons

A-T rich

D	f	%	RC	f	%	C	f	%	R	f	%	
**ATG**	138,00	1,95	CAT	119,00	1,69	TAC	57,00	0,81	GTA	45,00	0,64	Ia
**TGA**	177,00	2,51	TCA	177,00	2,51	ACT	109,00	1,54	AGT	122,00	1,73	
**TAG**	60,00	0,85	CTA	45,00	0,64	ATC	85,00	1,20	GAT	95,00	1,35	
**TAA**	48,00	0,68	TTA	81,00	1,15	ATT	97,00	1,37	AAT	100,00	1,42	Ib
**AAC**	101,00	1,43	GTT	107,00	1,52	TTG	135,00	1,91	CAA	143,00	2,03	
**AAG**	222,00	3,14	CTT	109,00	1,54	TTC	96,00	1,36	GAA	232,00	3,29	
**ACA**	133,00	1,88	TGT	113,00	1,60	TGT*			ACA*			Ic
**ATA**	63,00	0,89	TAT	69,00	0,98	TAT*			ATA*			
**AGA**	234,00	3,31	TCT	114,00	1,61	TCT*			AGA*			
**AAA**	192,00	2,72	TTT	126,00	1,78	TTT*			AAA*			

Quadruplet		f1	f2	fu
ATG	ATG(D)	1,95	0,81	2,76
	CAT(RC(D))	1,69	0,64	2,32
	TAC(C(D))	0,81	1,95	2,76
	GTA(R(D))	0,64	1,69	2,32
TGA	TGA(D)	2,51	1,54	4,05
	TCA(RC(D))	2,51	1,73	4,24
	ACT(C(D))	1,54	2,51	4,05
	AGT(R(D))	1,73	2,51	4,24
TAG	TAG(D)	0,85	1,20	2,05
	CTA(RC(D))	0,64	1,35	1,98
	ATC(C(D))	1,20	0,85	2,05
	GAT(R(D))	1,35	0,64	1,98
TAA	TAA(D)	0,68	1,37	2,05
	TTA(RC(D))	1,15	1,42	2,56
	ATT(C(D))	1,37	0,68	2,05
	AAT(R(D))	1,42	1,15	2,56
AAC	AAC(D)	1,43	1,91	3,34
	GTT(RC(D))	1,52	2,03	3,54
	TTG(C(D))	1,91	1,43	3,34
	CAA(R(D))	2,03	1,52	3,54
AAG	AAG(D)	3,14	1,36	4,50
	CTT(RC(D))	1,54	3,29	4,83
	TTC(C(D))	1,36	3,14	4,50
	GAA(R(D))	3,29	1,54	4,83
ACA	ACA(D)	0,94	0,80	1,74
	TGT(RC(D))	0,80	0,94	1,74
	TGT(C(D))	0,80	0,94	1,74
	ACA(R(D))	0,94	0,80	1,74
ATA	ATA(D)	0,45	0,49	0,93
	TAT(RC(D))	0,49	0,45	0,93
	TAT(C(D))	0,49	0,45	0,93
	ATA(R(D))	0,45	0,45	0,89
AGA	AGA(D)	1,66	0,81	2,46
	TCT(RC(D))	0,81	1,66	2,46
	TCT(C(D))	0,81	1,66	2,46
	AGA(R(D))	1,66	0,81	2,46
AAA	AAA(D)	1,36	0,89	2,25
	TTT(RC(D))	0,89	1,36	2,25
	TTT(C(D))	0,89	1,36	2,25
	AAA(R(D))	1,36	0,89	2,25

Composition of DNA sequence in NBPF12 exons

Base	No.of bases	%
A	2049	29,02%
C	1581	22,39%
G	1797	25,45%
T	1634	23,14%
N	0	0,00%
Sum	7061	100,00%

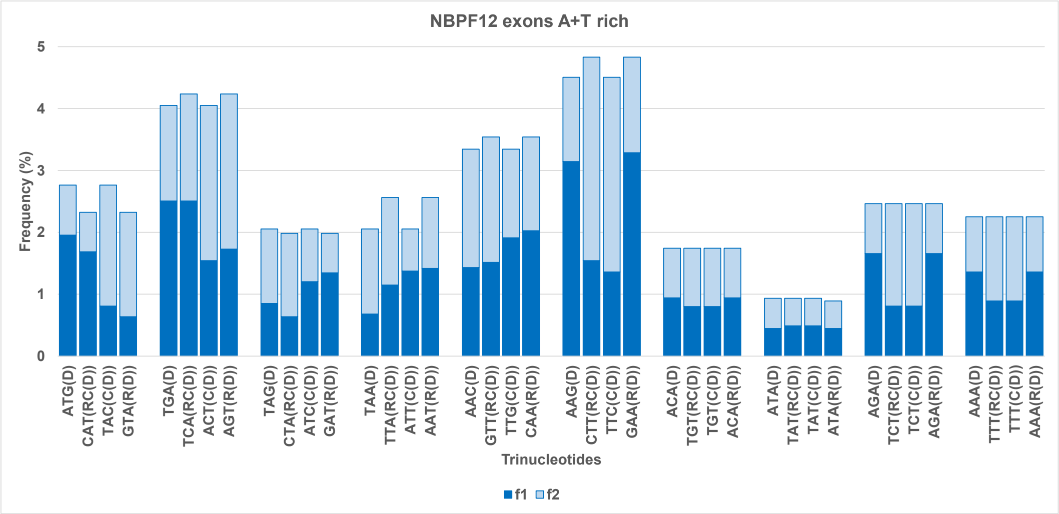

NBPF12 introns

A-T rich												
**D**	**f**	**%**	**RC**	**f**	**%**	**C**	**f**	**%**	**R**	**f**	**%**	
**ATG**	995,00	1,97	CAT	986,00	1,96	TAC	515,00	1,02	GTA	542,00	1,08	Ia
**TGA**	1106,00	2,19	TCA	1026,00	2,04	ACT	772,00	1,53	AGT	831,00	1,65	
**TAG**	600,00	1,19	CTA	610,00	1,21	ATC	661,00	1,31	GAT	667,00	1,32	
**TAA**	766,00	1,52	TTA	793,00	1,57	ATT	1091,00	2,16	AAT	1030,00	2,04	Ib
**AAC**	614,00	1,22	GTT	812,00	1,61	TTG	1049,00	2,08	CAA	727,00	1,44	
**AAG**	804,00	1,60	CTT	1102,00	2,19	TTC	1163,00	2,31	GAA	955,00	1,89	
**ACA**	851,00	1,69	TGT	1435,00	2,85	TGT*			ACA*			Ic
**ATA**	800,00	1,59	TAT	864,00	1,71	TAT*			ATA*			
**AGA**	962,00	1,91	TCT	1453,00	2,88	TCT*			AGA*			
**AAA**	1479,00	2,93	TTT	1805,00	3,58	TTT*			AAA*			

Quadruplet		f1	f2	fu
ATG	ATG(D)	1,97	1,02	3,00
	CAT(RC(D))	1,96	1,08	3,03
	TAC(C(D))	1,02	1,97	3,00
	GTA(R(D))	1,08	1,96	3,03
TGA	TGA(D)	2,19	1,53	3,73
	TCA(RC(D))	2,04	1,65	3,68
	ACT(C(D))	1,53	2,19	3,73
	AGT(R(D))	1,65	2,04	3,68
TAG	TAG(D)	1,19	1,31	2,50
	CTA(RC(D))	1,21	1,32	2,53
	ATC(C(D))	1,31	1,19	2,50
	GAT(R(D))	1,32	1,21	2,53
TAA	TAA(D)	1,52	2,16	3,68
	TTA(RC(D))	1,57	2,04	3,62
	ATT(C(D))	2,16	1,52	3,68
	AAT(R(D))	2,04	1,57	3,62
AAC	AAC(D)	1,22	2,08	3,30
	GTT(RC(D))	1,61	1,44	3,05
	TTG(C(D))	2,08	1,22	3,30
	CAA(R(D))	1,44	1,61	3,05
AAG	AAG(D)	1,60	2,31	3,90
	CTT(RC(D))	2,19	1,89	4,08
	TTC(C(D))	2,31	1,60	3,90
	GAA(R(D))	1,89	2,19	4,08
ACA	ACA(D)	0,84	1,42	2,27
	TGT(RC(D))	1,42	0,84	2,27
	TGT(C(D))	1,42	0,84	2,27
	ACA(R(D))	0,84	1,42	2,27
ATA	ATA(D)	0,79	0,86	1,65
	TAT(RC(D))	0,86	0,79	1,65
	TAT(C(D))	0,86	0,79	1,65
	ATA(R(D))	0,79	0,79	1,59
AGA	AGA(D)	0,95	1,44	2,40
	TCT(RC(D))	1,44	0,95	2,40
	TCT(C(D))	1,44	0,95	2,40
	AGA(R(D))	0,95	1,44	2,40
AAA	AAA(D)	1,47	1,79	3,26
	TTT(RC(D))	1,79	1,47	3,26
	TTT(C(D))	1,79	1,47	3,26
	AAA(R(D))	1,47	1,79	3,26

Composition of DNA sequence NBPF12 introns

Base	No.of bases	%
A	13162	26,12%
C	10845	21,52%
G	10997	21,82%
T	15394	30,54%
N	0	0,00%
Sum	50398	100,00%

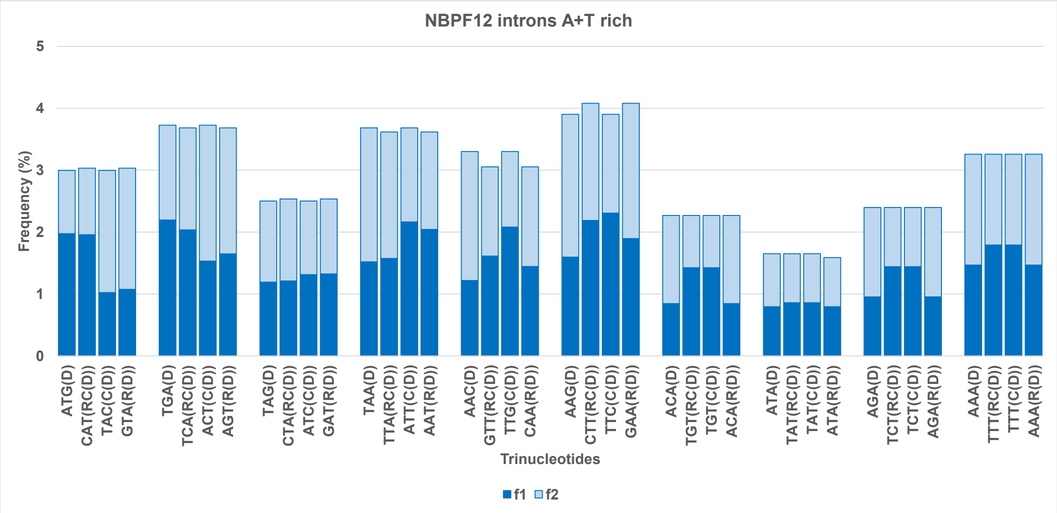

						C+G rich						
**D**	**f**	**%**	**RC**	**f**	**%**	**C**	**f**	**%**	**R**	**f**	**%**	
**CGT**	184,00	0,31	ACG	170,00	0,29	GCA	853,00	1,45	TGC	860,00	1,47	IIa
**GTC**	738,00	1,26	GAC	618,00	1,05	CAG	1296,00	2,21	CTG	1433,00	2,44	
**GCT**	855,00	1,46	AGC	766,00	1,31	CGA	177,00	0,30	TCG	136,00	0,23	
**GCC**	714,00	1,22	GGC	844,00	1,44	CGG	182,00	0,31	CCG	189,00	0,32	IIb
**CCA**	1049,00	1,79	TGG	1194,00	2,04	GGT	680,00	1,16	ACC	692,00	1,18	
**CCT**	1254,00	2,14	AGG	1142,00	1,95	GGA	994,00	1,69	TCC	1086,00	1,85	
**CAC**	825,00	1,41	GTG	1146,00	1,95	GTG*			CAC*			IIc
**CGC**	137,00	0,23	GCG	185,00	0,32	GCG*			CGC*			
**CTC**	1258,00	2,14	GAG	1086,00	1,85	GAG*			CTC*			
**CCC**	824,00	1,40	GGG	856,00	1,46	GGG*			CCC*			

Quadruplet		f1	f2	fu
CGT	CGT(D)	0,31	1,45	1,77
	ACG(RC(D))	0,29	1,47	1,76
	GCA(C(D))	1,45	0,31	1,77
	TGC(R(D))	1,47	0,29	1,76
GTC	GTC(D)	1,26	2,21	3,47
	GAC(RC(D))	1,05	2,44	3,50
	CAG(C(D))	2,21	1,26	3,47
	CTG(R(D))	2,44	1,05	3,50
GCT	GCT(D)	1,46	0,30	1,76
	AGC(RC(D))	1,31	0,23	1,54
	CGA(C(D))	0,30	1,46	1,76
	TCG(R(D))	0,23	1,31	1,54
GCC	GCC(D)	1,22	0,31	1,53
	GGC(RC(D))	1,44	0,32	1,76
	CGG(C(D))	0,31	1,22	1,53
	CCG(R(D))	0,32	1,44	1,76
CCA	CCA(D)	1,79	1,16	2,95
	TGG(RC(D))	2,04	1,18	3,22
	GGT(C(D))	1,16	1,79	2,95
	ACC(R(D))	1,18	2,04	3,22
CCT	CCT(D)	2,14	1,69	3,83
	AGG(RC(D))	1,95	1,85	3,80
	GGA(C(D))	1,69	2,14	3,83
	TCC(R(D))	1,85	1,95	3,80
CAC	CAC(D)	0,70	0,98	1,68
	GTG(RC(D))	0,98	0,70	1,68
	GTG(C(D))	0,98	0,70	1,68
	CAC(R(D))	0,70	0,98	1,68
CGC	CGC(D)	0,12	0,16	0,27
	GCG(RC(D))	0,16	0,12	0,27
	GCG(C(D))	0,16	0,12	0,27
	CGC(R(D))	0,12	0,16	0,27
CTC	CTC(D)	1,07	0,93	2,00
	GAG(RC(D))	0,93	1,07	2,00
	GAG(C(D))	0,93	1,07	2,00
	CTC(R(D))	1,07	0,93	2,00
CCC	CCC(D)	0,70	0,73	1,43
	GGG(RC(D))	0,73	0,70	1,43
	GGG(C(D))	0,73	0,70	1,43
	CCC(R(D))	0,70	0,73	1,43

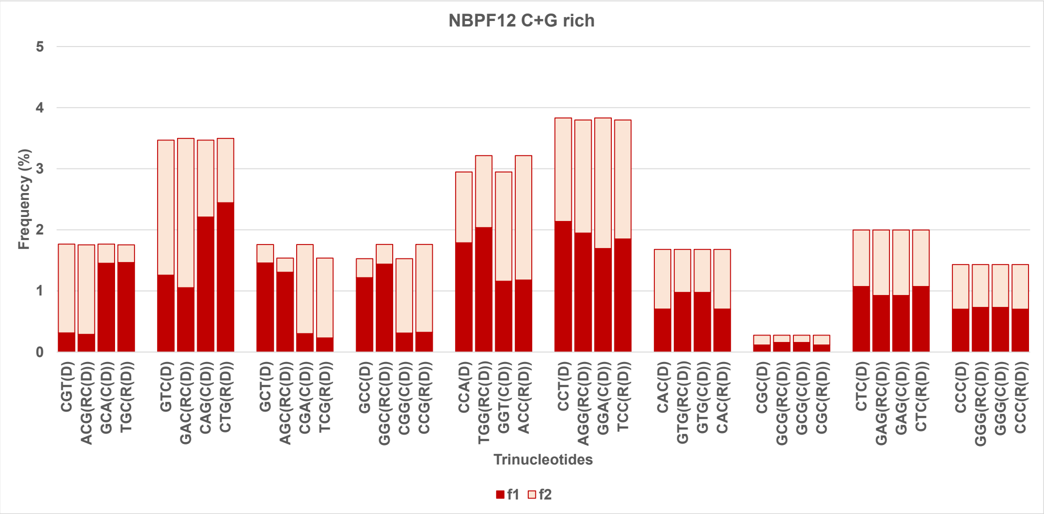

NBPF12 exons

C+G rich

D	f	%	RC	f	%	C	f	%	R	f	%	
**CGT**	16,00	0,23	ACG	31,00	0,44	GCA	114,00	1,61	TGC	111,00	1,57	IIa
**GTC**	80,00	1,13	GAC	108,00	1,53	CAG	222,00	3,14	CTG	184,00	2,61	
**GCT**	124,00	1,76	AGC	155,00	2,20	CGA	26,00	0,37	TCG	10,00	0,14	
**GCC**	124,00	1,76	GGC	108,00	1,53	CGG	37,00	0,52	CCG	26,00	0,37	IIb
**CCA**	150,00	2,12	TGG	146,00	2,07	GGT	71,00	1,01	ACC	83,00	1,18	
**CCT**	143,00	2,03	AGG	184,00	2,61	GGA	189,00	2,68	TCC	112,00	1,59	
**CAC**	90,00	1,27	GTG	90,00	1,27	GTG*			CAC*			IIc
**CGC**	14,00	0,20	GCG	26,00	0,37	GCG*			CGC*			
**CTC**	152,00	2,15	GAG	191,00	2,71	GAG*			CTC*			
**CCC**	105,00	1,49	GGG	93,00	1,32	GGG*			CCC*			

Quadruplet		f1	f2	fu
CGT	CGT(D)	0,23	1,61	1,84
	ACG(RC(D))	0,44	1,57	2,01
	GCA(C(D))	1,61	0,23	1,84
	TGC(R(D))	1,57	0,44	2,01
GTC	GTC(D)	1,13	3,14	4,28
	GAC(RC(D))	1,53	2,61	4,14
	CAG(C(D))	3,14	1,13	4,28
	CTG(R(D))	2,61	1,53	4,14
GCT	GCT(D)	1,76	0,37	2,12
	AGC(RC(D))	2,20	0,14	2,34
	CGA(C(D))	0,37	1,76	2,12
	TCG(R(D))	0,14	2,20	2,34
GCC	GCC(D)	1,76	0,52	2,28
	GGC(RC(D))	1,53	0,37	1,90
	CGG(C(D))	0,52	1,76	2,28
	CCG(R(D))	0,37	1,53	1,90
CCA	CCA(D)	2,12	1,01	3,13
	TGG(RC(D))	2,07	1,18	3,24
	GGT(C(D))	1,01	2,12	3,13
	ACC(R(D))	1,18	2,07	3,24
CCT	CCT(D)	2,03	2,68	4,70
	AGG(RC(D))	2,61	1,59	4,19
	GGA(C(D))	2,68	2,03	4,70
	TCC(R(D))	1,59	2,61	4,19
CAC	CAC(D)	0,64	0,64	1,27
	GTG(RC(D))	0,64	0,64	1,27
	GTG(C(D))	0,64	0,64	1,27
	CAC(R(D))	0,64	0,64	1,27
CGC	CGC(D)	0,10	0,18	0,28
	GCG(RC(D))	0,18	0,10	0,28
	GCG(C(D))	0,18	0,10	0,28
	CGC(R(D))	0,10	0,18	0,28
CTC	CTC(D)	1,08	1,35	2,43
	GAG(RC(D))	1,35	1,08	2,43
	GAG(C(D))	1,35	1,08	2,43
	CTC(R(D))	1,08	1,35	2,43
CCC	CCC(D)	0,74	0,66	1,40
	GGG(RC(D))	0,66	0,74	1,40
	GGG(C(D))	0,66	0,74	1,40
	CCC(R(D))	0,74	0,66	1,40

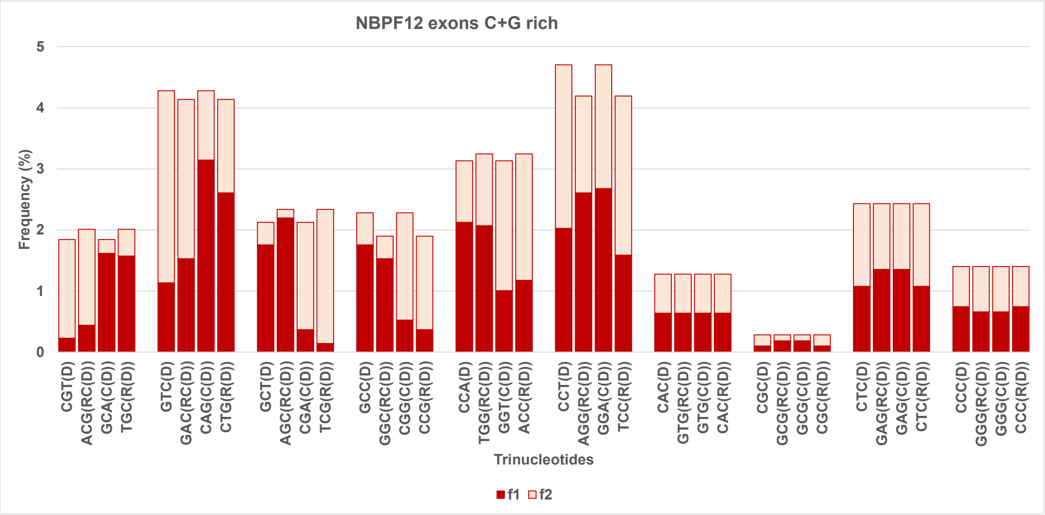

NBPF12 introns

C+G rich

D	f	%	RC	f	%	C	f	%	R	f	%	
**CGT**	158,00	0,31	ACG	134,00	0,27	GCA	728,00	1,44	TGC	728,00	1,44	IIa
**GTC**	638,00	1,27	GAC	503,00	1,00	CAG	1053,00	2,09	CTG	1229,00	2,44	
**GCT**	712,00	1,41	AGC	597,00	1,18	CGA	142,00	0,28	TCG	119,00	0,24	
**GCC**	567,00	1,13	GGC	712,00	1,41	CGG	124,00	0,25	CCG	141,00	0,28	IIb
**CCA**	884,00	1,75	TGG	1030,00	2,04	GGT	593,00	1,18	ACC	598,00	1,19	
**CCT**	1082,00	2,15	AGG	942,00	1,87	GGA	791,00	1,57	TCC	942,00	1,87	
**CAC**	723,00	1,43	GTG	1026,00	2,04	GTG*			CAC*			IIc
**CGC**	107,00	0,21	GCG	137,00	0,27	GCG*			CGC*			
**CTC**	1078,00	2,14	GAG	876,00	1,74	GAG*			CTC*			
**CCC**	699,00	1,39	GGG	737,00	1,46	GGG*			CCC*			

Quadruplet		f1	f2	fu
CGT	CGT(D)	0,31	1,44	1,76
	ACG(RC(D))	0,27	1,44	1,71
	GCA(C(D))	1,44	0,31	1,76
	TGC(R(D))	1,44	0,27	1,71
GTC	GTC(D)	1,27	2,09	3,36
	GAC(RC(D))	1,00	2,44	3,44
	CAG(C(D))	2,09	1,27	3,36
	CTG(R(D))	2,44	1,00	3,44
GCT	GCT(D)	1,41	0,28	1,69
	AGC(RC(D))	1,18	0,24	1,42
	CGA(C(D))	0,28	1,41	1,69
	TCG(R(D))	0,24	1,18	1,42
GCC	GCC(D)	1,13	0,25	1,37
	GGC(RC(D))	1,41	0,28	1,69
	CGG(C(D))	0,25	1,13	1,37
	CCG(R(D))	0,28	1,41	1,69
CCA	CCA(D)	1,75	1,18	2,93
	TGG(RC(D))	2,04	1,19	3,23
	GGT(C(D))	1,18	1,75	2,93
	ACC(R(D))	1,19	2,04	3,23
CCT	CCT(D)	2,15	1,57	3,72
	AGG(RC(D))	1,87	1,87	3,74
	GGA(C(D))	1,57	2,15	3,72
	TCC(R(D))	1,87	1,87	3,74
CAC	CAC(D)	0,72	1,02	1,74
	GTG(RC(D))	1,02	0,72	1,74
	GTG(C(D))	1,02	0,72	1,74
	CAC(R(D))	0,72	1,02	1,74
CGC	CGC(D)	0,11	0,14	0,24
	GCG(RC(D))	0,14	0,11	0,24
	GCG(C(D))	0,14	0,11	0,24
	CGC(R(D))	0,11	0,14	0,24
CTC	CTC(D)	1,07	0,87	1,94
	GAG(RC(D))	0,87	1,07	1,94
	GAG(C(D))	0,87	1,07	1,94
	CTC(R(D))	1,07	0,87	1,94
CCC	CCC(D)	0,69	0,73	1,42
	GGG(RC(D))	0,73	0,69	1,42
	GGG(C(D))	0,73	0,69	1,42
	CCC(R(D))	0,69	0,73	1,42

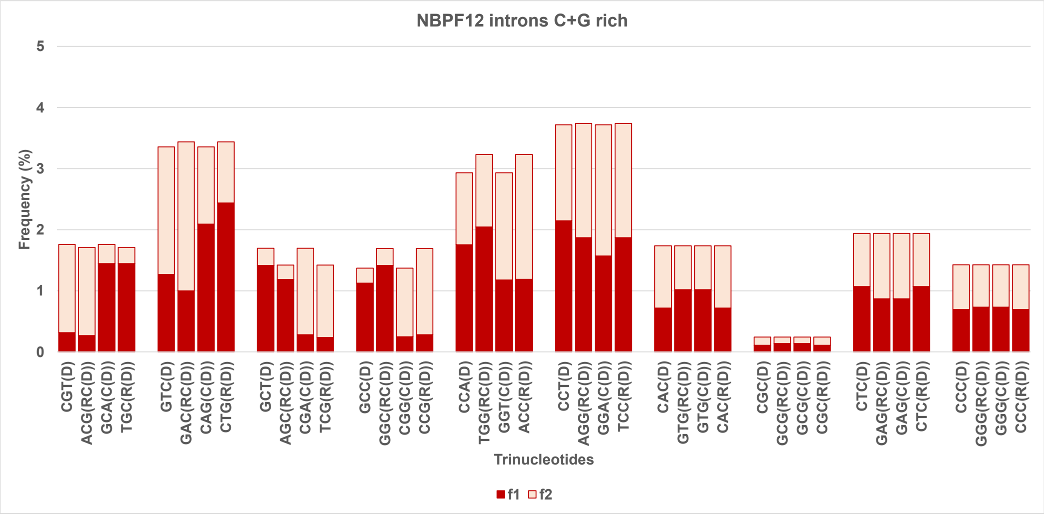

NBPF13P sequence

						A-T rich						
**D**	**f**	**%**	**RC**	**f**	**%**	**C**	**f**	**%**	**R**	**f**	**%**	
**ATG**	670,00	1,76	CAT	739,00	1,94	TAC	403,00	1,06	GTA	468,00	1,23	Ia
**TGA**	756,00	1,98	TCA	801,00	2,10	ACT	600,00	1,57	AGT	730,00	1,91	
**TAG**	579,00	1,52	CTA	500,00	1,31	ATC	512,00	1,34	GAT	551,00	1,44	
**TAA**	770,00	2,02	TTA	819,00	2,15	ATT	953,00	2,50	AAT	810,00	2,12	Ib
**AAC**	515,00	1,35	GTT	623,00	1,63	TTG	768,00	2,01	CAA	614,00	1,61	
**AAG**	784,00	2,06	CTT	962,00	2,52	TTC	951,00	2,49	GAA	725,00	1,90	
**ACA**	645,00	1,69	TGT	801,00	2,10	TGT*			ACA*			Ic
**ATA**	739,00	1,94	TAT	774,00	2,03	TAT*			ATA*			
**AGA**	884,00	2,32	TCT	958,00	2,51	TCT*			AGA*			
**AAA**	1209,00	3,17	TTT	1642,00	4,31	TTT*			AAA*			

Quadruplet		f1	f2	fu
ATG	ATG(D)	1,76	1,06	2,81
	CAT(RC(D))	1,94	1,23	3,16
	TAC(C(D))	1,06	1,76	2,81
	GTA(R(D))	1,23	1,94	3,16
TGA	TGA(D)	1,98	1,57	3,56
	TCA(RC(D))	2,10	1,91	4,01
	ACT(C(D))	1,57	1,98	3,56
	AGT(R(D))	1,91	2,10	4,01
TAG	TAG(D)	1,52	1,34	2,86
	CTA(RC(D))	1,31	1,44	2,76
	ATC(C(D))	1,34	1,52	2,86
	GAT(R(D))	1,44	1,31	2,76
TAA	TAA(D)	2,02	2,50	4,52
	TTA(RC(D))	2,15	2,12	4,27
	ATT(C(D))	2,50	2,02	4,52
	AAT(R(D))	2,12	2,15	4,27
AAC	AAC(D)	1,35	2,01	3,36
	GTT(RC(D))	1,63	1,61	3,24
	TTG(C(D))	2,01	1,35	3,36
	CAA(R(D))	1,61	1,63	3,24
AAG	AAG(D)	2,06	2,49	4,55
	CTT(RC(D))	2,52	1,90	4,42
	TTC(C(D))	2,49	2,06	4,55
	GAA(R(D))	1,90	2,52	4,42
ACA	ACA(D)	0,85	1,05	1,90
	TGT(RC(D))	1,05	0,85	1,90
	TGT(C(D))	1,05	0,85	1,90
	ACA(R(D))	0,85	1,05	1,90
ATA	ATA(D)	0,97	1,01	1,98
	TAT(RC(D))	1,01	0,97	1,98
	TAT(C(D))	1,01	0,97	1,98
	ATA(R(D))	0,97	0,97	1,94
AGA	AGA(D)	1,16	1,26	2,42
	TCT(RC(D))	1,26	1,16	2,42
	TCT(C(D))	1,26	1,16	2,42
	AGA(R(D))	1,16	1,26	2,42
AAA	AAA(D)	1,59	2,15	3,74
	TTT(RC(D))	2,15	1,59	3,74
	TTT(C(D))	2,15	1,59	3,74
	AAA(R(D))	1,59	2,15	3,74

Composition of DNA sequence in NBPF13P

Base	No.of bases	%
A	10684	28,01%
C	7780	20,40%
G	7670	20,11%
T	12004	31,48%
N	0	0,00%
Sum	38138	100,00%

% of coding bases from NBPF13P (including N bases)

**92,18%**

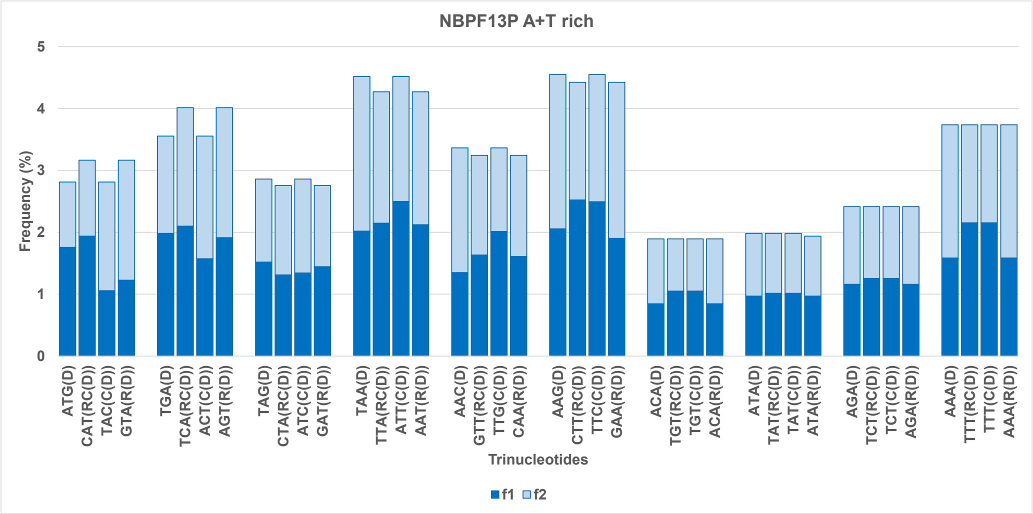

NBPF13P exons

A-T rich												
**D**	**f**	**%**	**RC**	**f**	**%**	**C**	**f**	**%**	**R**	**f**	**%**	
**ATG**	41,00	2,30	CAT	30,00	1,68	TAC	13,00	0,73	GTA	11,00	0,62	Ia
**TGA**	38,00	2,13	TCA	25,00	1,40	ACT	26,00	1,46	AGT	37,00	2,08	
**TAG**	21,00	1,18	CTA	23,00	1,29	ATC	19,00	1,07	GAT	31,00	1,74	
**TAA**	15,00	0,84	TTA	12,00	0,67	ATT	19,00	1,07	AAT	21,00	1,18	Ib
**AAC**	29,00	1,63	GTT	14,00	0,79	TTG	26,00	1,46	CAA	41,00	2,30	
**AAG**	58,00	3,26	CTT	31,00	1,74	TTC	26,00	1,46	GAA	52,00	2,92	
**ACA**	33,00	1,85	TGT	15,00	0,84	TGT*			ACA*			Ic
**ATA**	13,00	0,73	TAT	10,00	0,56	TAT*			ATA*			
**AGA**	75,00	4,21	TCT	30,00	1,68	TCT*			AGA*			
**AAA**	42,00	2,36	TTT	10,00	0,56	TTT*			AAA*			

Quadruplet		f1	f2	fu
ATG	ATG(D)	2,30	0,73	3,03
	CAT(RC(D))	1,68	0,62	2,30
	TAC(C(D))	0,73	2,30	3,03
	GTA(R(D))	0,62	1,68	2,30
TGA	TGA(D)	2,13	1,46	3,59
	TCA(RC(D))	1,40	2,08	3,48
	ACT(C(D))	1,46	2,13	3,59
	AGT(R(D))	2,08	1,40	3,48
TAG	TAG(D)	1,18	1,07	2,25
	CTA(RC(D))	1,29	1,74	3,03
	ATC(C(D))	1,07	1,18	2,25
	GAT(R(D))	1,74	1,29	3,03
TAA	TAA(D)	0,84	1,07	1,91
	TTA(RC(D))	0,67	1,18	1,85
	ATT(C(D))	1,07	0,84	1,91
	AAT(R(D))	1,18	0,67	1,85
AAC	AAC(D)	1,63	1,46	3,09
	GTT(RC(D))	0,79	2,30	3,09
	TTG(C(D))	1,46	1,63	3,09
	CAA(R(D))	2,30	0,79	3,09
AAG	AAG(D)	3,26	1,46	4,72
	CTT(RC(D))	1,74	2,92	4,66
	TTC(C(D))	1,46	3,26	4,72
	GAA(R(D))	2,92	1,74	4,66
ACA	ACA(D)	0,93	0,42	1,35
	TGT(RC(D))	0,42	0,93	1,35
	TGT(C(D))	0,42	0,93	1,35
	ACA(R(D))	0,93	0,42	1,35
ATA	ATA(D)	0,36	0,28	0,65
	TAT(RC(D))	0,28	0,36	0,65
	TAT(C(D))	0,28	0,36	0,65
	ATA(R(D))	0,36	0,36	0,73
AGA	AGA(D)	2,11	0,84	2,95
	TCT(RC(D))	0,84	2,11	2,95
	TCT(C(D))	0,84	2,11	2,95
	AGA(R(D))	2,11	0,84	2,95
AAA	AAA(D)	1,18	0,28	1,46
	TTT(RC(D))	0,28	1,18	1,46
	TTT(C(D))	0,28	1,18	1,46
	AAA(R(D))	1,18	0,28	1,46

Composition of DNA sequence in NBPF13P exons

Base	No.of bases	%
A	525	29,44%
C	422	23,67%
G	468	26,25%
T	368	20,64%
N	0	0,00%
Sum	1783	100,00%

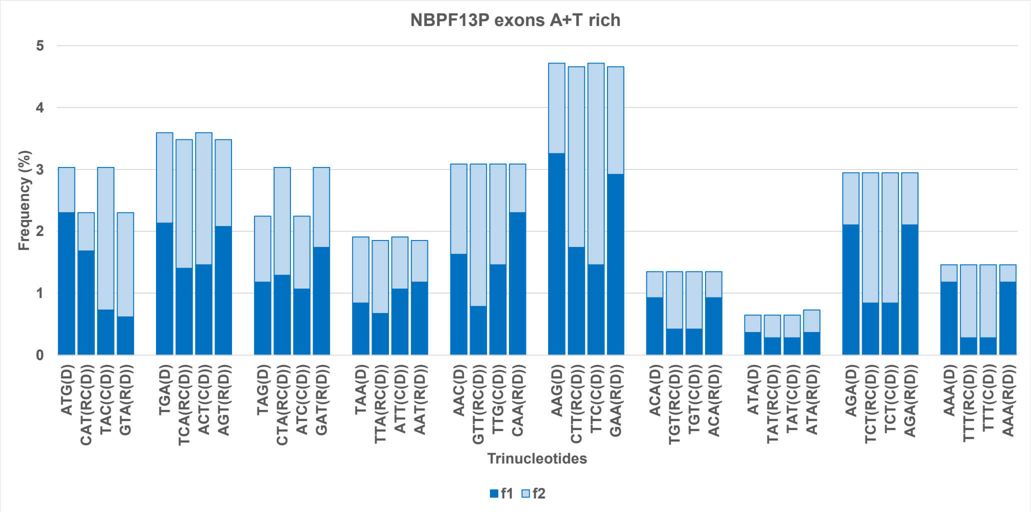

NBPF13P introns

A-T rich

D	f	%	RC	f	%	C	f	%	R	f	%	
**ATG**	610,00	1,74	CAT	688,00	1,96	TAC	380,00	1,08	GTA	441,00	1,25	Ia
**TGA**	701,00	1,99	TCA	754,00	2,14	ACT	553,00	1,57	AGT	666,00	1,89	
**TAG**	538,00	1,53	CTA	456,00	1,30	ATC	478,00	1,36	GAT	505,00	1,44	
**TAA**	735,00	2,09	TTA	787,00	2,24	ATT	915,00	2,60	AAT	779,00	2,22	Ib
**AAC**	467,00	1,33	GTT	588,00	1,67	TTG	725,00	2,06	CAA	561,00	1,60	
**AAG**	699,00	1,99	CTT	906,00	2,58	TTC	897,00	2,55	GAA	649,00	1,85	
**ACA**	587,00	1,67	TGT	761,00	2,16	TGT*			ACA*			Ic
**ATA**	706,00	2,01	TAT	737,00	2,10	TAT*			ATA*			
**AGA**	779,00	2,22	TCT	906,00	2,58	TCT*			AGA*			
**AAA**	1133,00	3,22	TTT	1605,00	4,57	TTT*			AAA*			

Quadruplet		f1	f2	fu
ATG	ATG(D)	1,74	1,08	2,82
	CAT(RC(D))	1,96	1,25	3,21
	TAC(C(D))	1,08	1,74	2,82
	GTA(R(D))	1,25	1,96	3,21
TGA	TGA(D)	1,99	1,57	3,57
	TCA(RC(D))	2,14	1,89	4,04
	ACT(C(D))	1,57	1,99	3,57
	AGT(R(D))	1,89	2,14	4,04
TAG	TAG(D)	1,53	1,36	2,89
	CTA(RC(D))	1,30	1,44	2,73
	ATC(C(D))	1,36	1,53	2,89
	GAT(R(D))	1,44	1,30	2,73
TAA	TAA(D)	2,09	2,60	4,69
	TTA(RC(D))	2,24	2,22	4,45
	ATT(C(D))	2,60	2,09	4,69
	AAT(R(D))	2,22	2,24	4,45
AAC	AAC(D)	1,33	2,06	3,39
	GTT(RC(D))	1,67	1,60	3,27
	TTG(C(D))	2,06	1,33	3,39
	CAA(R(D))	1,60	1,67	3,27
AAG	AAG(D)	1,99	2,55	4,54
	CTT(RC(D))	2,58	1,85	4,42
	TTC(C(D))	2,55	1,99	4,54
	GAA(R(D))	1,85	2,58	4,42
ACA	ACA(D)	0,83	1,08	1,92
	TGT(RC(D))	1,08	0,83	1,92
	TGT(C(D))	1,08	0,83	1,92
	ACA(R(D))	0,83	1,08	1,92
ATA	ATA(D)	1,00	1,05	2,05
	TAT(RC(D))	1,05	1,00	2,05
	TAT(C(D))	1,05	1,00	2,05
	ATA(R(D))	1,00	1,00	2,01
AGA	AGA(D)	1,11	1,29	2,40
	TCT(RC(D))	1,29	1,11	2,40
	TCT(C(D))	1,29	1,11	2,40
	AGA(R(D))	1,11	1,29	2,40
AAA	AAA(D)	1,61	2,28	3,89
	TTT(RC(D))	2,28	1,61	3,89
	TTT(C(D))	2,28	1,61	3,89
	AAA(R(D))	1,61	2,28	3,89

Composition of DNA sequence NBPF13P introns

Base	No.of bases	%
A	9831	27,96%
C	7083	20,15%
G	6938	19,74%
T	11303	32,15%
N	0	0,00%
Sum	35155	100,00%

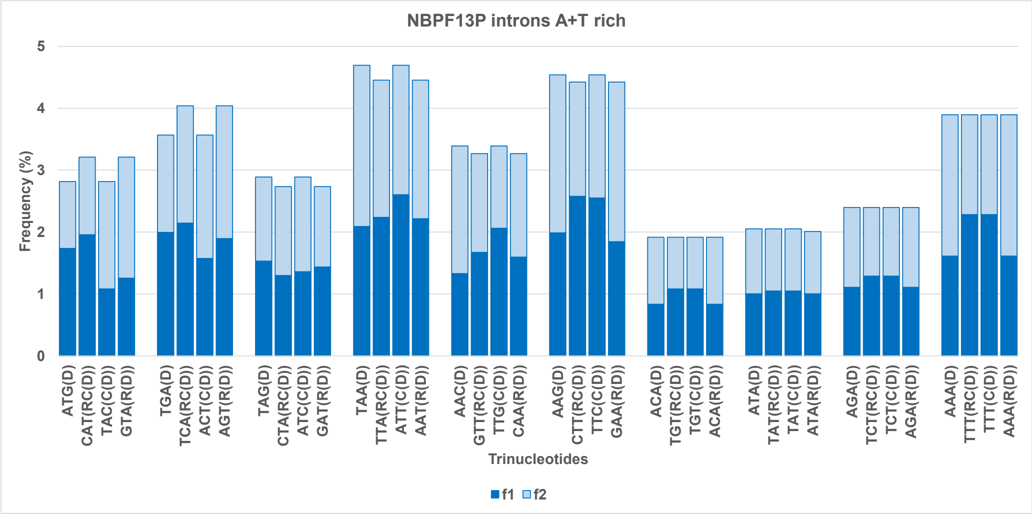

NBPF13P sequence

D	f	%	RC	f	%	C	f	%	R	f	%	
						C+G rich						
**CGT**	77,00	0,20	ACG	65,00	0,17	GCA	530,00	1,39	TGC	565,00	1,48	IIa
**GTC**	369,00	0,97	GAC	314,00	0,82	CAG	745,00	1,95	CTG	782,00	2,05	
**GCT**	571,00	1,50	AGC	544,00	1,43	CGA	75,00	0,20	TCG	78,00	0,20	
**GCC**	418,00	1,10	GGC	413,00	1,08	CGG	91,00	0,24	CCG	97,00	0,25	IIb
**CCA**	610,00	1,60	TGG	676,00	1,77	GGT	429,00	1,12	ACC	409,00	1,07	
**CCT**	783,00	2,05	AGG	614,00	1,61	GGA	539,00	1,41	TCC	663,00	1,74	
**CAC**	487,00	1,28	GTG	578,00	1,52	GTG*			CAC*			IIc
**CGC**	61,00	0,16	GCG	64,00	0,17	GCG*			CGC*			
**CTC**	668,00	1,75	GAG	664,00	1,74	GAG*			CTC*			
**CCC**	488,00	1,28	GGG	414,00	1,09	GGG*			CCC*			

Quadruplet		f1	f2	fu
CGT	CGT(D)	0,20	1,39	1,59
	ACG(RC(D))	0,17	1,48	1,65
	GCA(C(D))	1,39	0,20	1,59
	TGC(R(D))	1,48	0,17	1,65
GTC	GTC(D)	0,97	1,95	2,92
	GAC(RC(D))	0,82	2,05	2,87
	CAG(C(D))	1,95	0,97	2,92
	CTG(R(D))	2,05	0,82	2,87
GCT	GCT(D)	1,50	0,20	1,69
	AGC(RC(D))	1,43	0,20	1,63
	CGA(C(D))	0,20	1,50	1,69
	TCG(R(D))	0,20	1,43	1,63
GCC	GCC(D)	1,10	0,24	1,33
	GGC(RC(D))	1,08	0,25	1,34
	CGG(C(D))	0,24	1,10	1,33
	CCG(R(D))	0,25	1,08	1,34
CCA	CCA(D)	1,60	1,12	2,72
	TGG(RC(D))	1,77	1,07	2,85
	GGT(C(D))	1,12	1,60	2,72
	ACC(R(D))	1,07	1,77	2,85
CCT	CCT(D)	2,05	1,41	3,47
	AGG(RC(D))	1,61	1,74	3,35
	GGA(C(D))	1,41	2,05	3,47
	TCC(R(D))	1,74	1,61	3,35
CAC	CAC(D)	0,64	0,76	1,40
	GTG(RC(D))	0,76	0,64	1,40
	GTG(C(D))	0,76	0,64	1,40
	CAC(R(D))	0,64	0,76	1,40
CGC	CGC(D)	0,08	0,08	0,16
	GCG(RC(D))	0,08	0,08	0,16
	GCG(C(D))	0,08	0,08	0,16
	CGC(R(D))	0,08	0,08	0,16
CTC	CTC(D)	0,88	0,87	1,75
	GAG(RC(D))	0,87	0,88	1,75
	GAG(C(D))	0,87	0,88	1,75
	CTC(R(D))	0,88	0,87	1,75
CCC	CCC(D)	0,64	0,54	1,18
	GGG(RC(D))	0,54	0,64	1,18
	GGG(C(D))	0,54	0,64	1,18
	CCC(R(D))	0,64	0,54	1,18

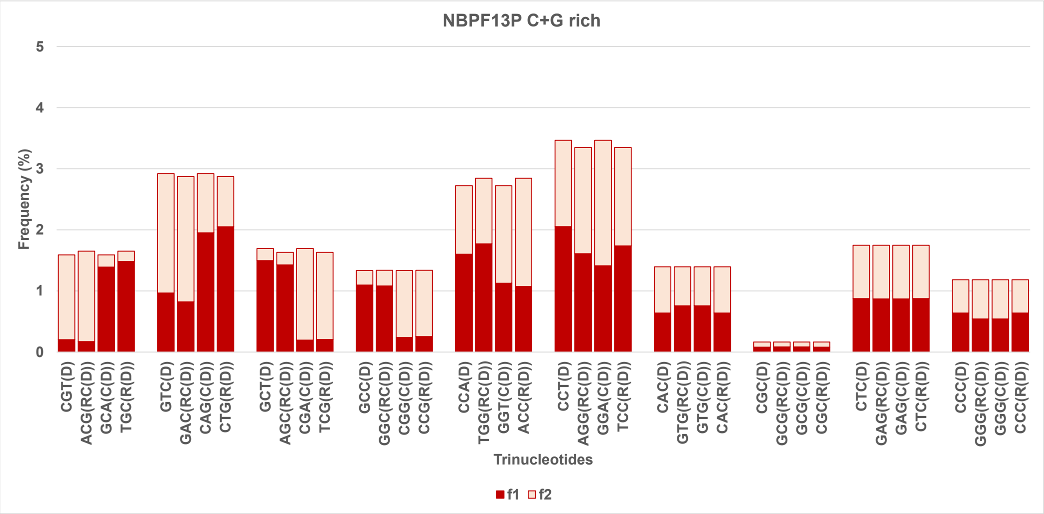

NBPF13P exons

C+G rich

D	f	%	RC	f	%	C	f	%	R	f	%		
**CGT**	2,00	0,11	ACG	3,00	0,17	GCA	49,00	2,75	TGC	51,00	2,86	IIa	86356
**GTC**	20,00	1,12	GAC	24,00	1,35	CAG	62,00	3,48	CTG	50,00	2,81		80741
**GCT**	42,00	2,36	AGC	43,00	2,41	CGA	5,00	0,28	TCG	8,00	0,45		44919
**GCC**	30,00	1,68	GGC	29,00	1,63	CGG	10,00	0,56	CCG	4,00	0,22	IIb	22459
**CCA**	48,00	2,70	TGG	39,00	2,19	GGT	18,00	1,01	ACC	26,00	1,46		45985
**CCT**	32,00	1,80	AGG	40,00	2,25	GGA	42,00	2,36	TCC	28,00	1,57		57215
**CAC**	22,00	1,24	GTG	27,00	1,52	GTG*			CAC*			IIc	
**CGC**	4,00	0,22	GCG	6,00	0,34	GCG*			CGC*				
**CTC**	26,00	1,46	GAG	54,00	3,03	GAG*			CTC*				
**CCC**	32,00	1,80	GGG	18,00	1,01	GGG*			CCC*				

Quadruplet		f1	f2	fu
CGT	CGT(D)	0,11	2,75	2,86
	ACG(RC(D))	0,17	2,86	3,03
	GCA(C(D))	2,75	0,11	2,86
	TGC(R(D))	2,86	0,17	3,03
GTC	GTC(D)	1,12	3,48	4,60
	GAC(RC(D))	1,35	2,81	4,15
	CAG(C(D))	3,48	1,12	4,60
	CTG(R(D))	2,81	1,35	4,15
GCT	GCT(D)	2,36	0,28	2,64
	AGC(RC(D))	2,41	0,45	2,86
	CGA(C(D))	0,28	2,36	2,64
	TCG(R(D))	0,45	2,41	2,86
GCC	GCC(D)	1,68	0,56	2,25
	GGC(RC(D))	1,63	0,22	1,85
	CGG(C(D))	0,56	1,68	2,25
	CCG(R(D))	0,22	1,63	1,85
CCA	CCA(D)	2,70	1,01	3,71
	TGG(RC(D))	2,19	1,46	3,65
	GGT(C(D))	1,01	2,70	3,71
	ACC(R(D))	1,46	2,19	3,65
CCT	CCT(D)	1,80	2,36	4,15
	AGG(RC(D))	2,25	1,57	3,82
	GGA(C(D))	2,36	1,80	4,15
	TCC(R(D))	1,57	2,25	3,82
CAC	CAC(D)	0,62	0,76	1,38
	GTG(RC(D))	0,76	0,62	1,38
	GTG(C(D))	0,76	0,62	1,38
	CAC(R(D))	0,62	0,76	1,38
CGC	CGC(D)	0,11	0,17	0,28
	GCG(RC(D))	0,17	0,11	0,28
	GCG(C(D))	0,17	0,11	0,28
	CGC(R(D))	0,11	0,17	0,28
CTC	CTC(D)	0,73	1,52	2,25
	GAG(RC(D))	1,52	0,73	2,25
	GAG(C(D))	1,52	0,73	2,25
	CTC(R(D))	0,73	1,52	2,25
CCC	CCC(D)	0,90	0,51	1,40
	GGG(RC(D))	0,51	0,90	1,40
	GGG(C(D))	0,51	0,90	1,40
	CCC(R(D))	0,90	0,51	1,40

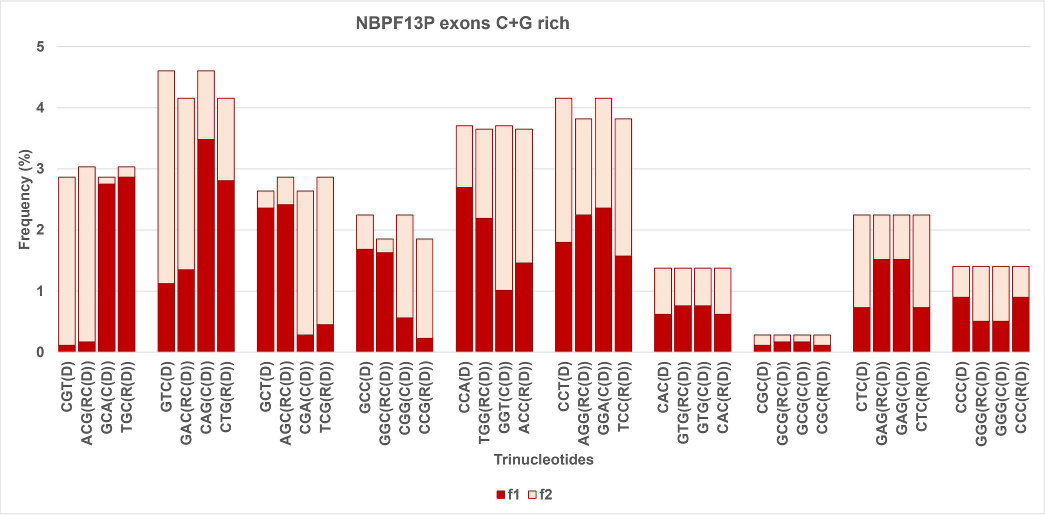

NBPF13P introns

C+G rich

D	f	%	RC	f	%	C	f	%	R	f	%	
**CGT**	71,00	0,20	ACG	58,00	0,16	GCA	455,00	1,29	TGC	494,00	1,41	IIa
**GTC**	335,00	0,95	GAC	281,00	0,80	CAG	647,00	1,84	CTG	701,00	1,99	
**GCT**	508,00	1,45	AGC	473,00	1,35	CGA	67,00	0,19	TCG	67,00	0,19	
**GCC**	371,00	1,06	GGC	369,00	1,05	CGG	77,00	0,22	CCG	88,00	0,25	IIb
**CCA**	542,00	1,54	TGG	611,00	1,74	GGT	396,00	1,13	ACC	372,00	1,06	
**CCT**	718,00	2,04	AGG	555,00	1,58	GGA	478,00	1,36	TCC	605,00	1,72	
**CAC**	442,00	1,26	GTG	531,00	1,51	GTG*			CAC*			IIc
**CGC**	56,00	0,16	GCG	58,00	0,16	GCG*			CGC*			
**CTC**	622,00	1,77	GAG	590,00	1,68	GAG*			CTC*			
**CCC**	441,00	1,25	GGG	382,00	1,09	GGG*			CCC*			

Quadruplet		f1	f2	fu
CGT	CGT(D)	0,20	1,29	1,50
	ACG(RC(D))	0,16	1,41	1,57
	GCA(C(D))	1,29	0,20	1,50
	TGC(R(D))	1,41	0,16	1,57
GTC	GTC(D)	0,95	1,84	2,79
	GAC(RC(D))	0,80	1,99	2,79
	CAG(C(D))	1,84	0,95	2,79
	CTG(R(D))	1,99	0,80	2,79
GCT	GCT(D)	1,45	0,19	1,64
	AGC(RC(D))	1,35	0,19	1,54
	CGA(C(D))	0,19	1,45	1,64
	TCG(R(D))	0,19	1,35	1,54
GCC	GCC(D)	1,06	0,22	1,27
	GGC(RC(D))	1,05	0,25	1,30
	CGG(C(D))	0,22	1,06	1,27
	CCG(R(D))	0,25	1,05	1,30
CCA	CCA(D)	1,54	1,13	2,67
	TGG(RC(D))	1,74	1,06	2,80
	GGT(C(D))	1,13	1,54	2,67
	ACC(R(D))	1,06	1,74	2,80
CCT	CCT(D)	2,04	1,36	3,40
	AGG(RC(D))	1,58	1,72	3,30
	GGA(C(D))	1,36	2,04	3,40
	TCC(R(D))	1,72	1,58	3,30
CAC	CAC(D)	0,63	0,76	1,38
	GTG(RC(D))	0,76	0,63	1,38
	GTG(C(D))	0,76	0,63	1,38
	CAC(R(D))	0,63	0,76	1,38
CGC	CGC(D)	0,08	0,08	0,16
	GCG(RC(D))	0,08	0,08	0,16
	GCG(C(D))	0,08	0,08	0,16
	CGC(R(D))	0,08	0,08	0,16
CTC	CTC(D)	0,88	0,84	1,72
	GAG(RC(D))	0,84	0,88	1,72
	GAG(C(D))	0,84	0,88	1,72
	CTC(R(D))	0,88	0,84	1,72
CCC	CCC(D)	0,63	0,54	1,17
	GGG(RC(D))	0,54	0,63	1,17
	GGG(C(D))	0,54	0,63	1,17
	CCC(R(D))	0,63	0,54	1,17

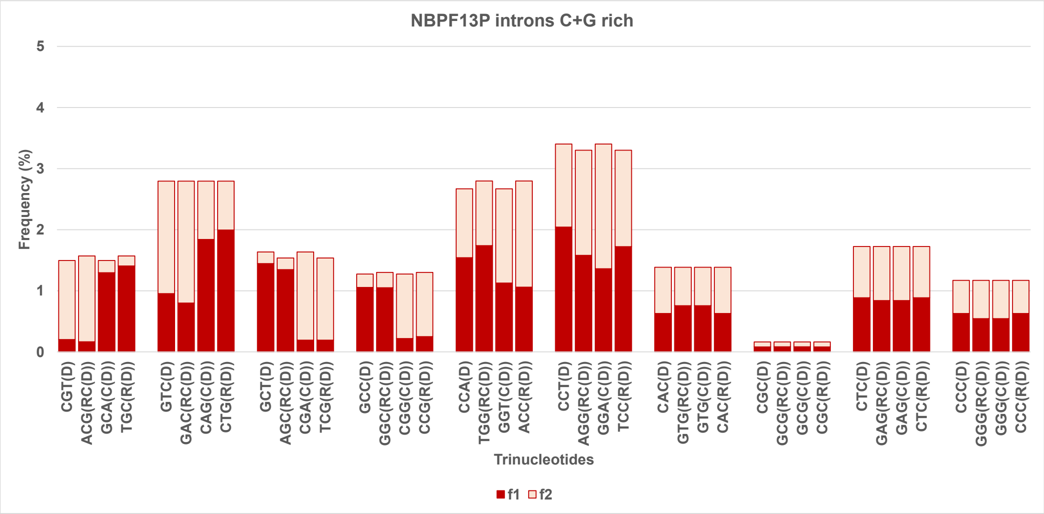

NBPF14 sequence

						A-T rich						
**D**	**f**	**%**	**RC**	**f**	**%**	**C**	**f**	**%**	**R**	**f**	**%**	
**ATG**	2929,00	1,96	CAT	2786,00	1,86	TAC	1630,00	1,09	GTA	1734,00	1,16	Ia
**TGA**	3421,00	2,29	TCA	3044,00	2,04	ACT	2553,00	1,71	AGT	2585,00	1,73	
**TAG**	2099,00	1,40	CTA	1885,00	1,26	ATC	1816,00	1,21	GAT	2246,00	1,50	
**TAA**	2474,00	1,65	TTA	3050,00	2,04	ATT	3826,00	2,56	AAT	3073,00	2,05	Ib
**AAC**	1894,00	1,27	GTT	2590,00	1,73	TTG	3320,00	2,22	CAA	2160,00	1,44	
**AAG**	2726,00	1,82	CTT	3449,00	2,31	TTC	3495,00	2,34	GAA	3059,00	2,05	
**ACA**	2436,00	1,63	TGT	4077,00	2,73	TGT*			ACA*			Ic
**ATA**	2442,00	1,63	TAT	2908,00	1,94	TAT*			ATA*			
**AGA**	3037,00	2,03	TCT	4017,00	2,69	TCT*			AGA*			
**AAA**	4100,00	2,74	TTT	6382,00	4,27	TTT*			AAA*			

Quadruplet		f1	f2	fu
ATG	ATG(D)	1,96	1,09	3,05
	CAT(RC(D))	1,86	1,16	3,02
	TAC(C(D))	1,09	1,96	3,05
	GTA(R(D))	1,16	1,86	3,02
TGA	TGA(D)	2,29	1,71	3,99
	TCA(RC(D))	2,04	1,73	3,76
	ACT(C(D))	1,71	2,29	3,99
	AGT(R(D))	1,73	2,04	3,76
TAG	TAG(D)	1,40	1,21	2,62
	CTA(RC(D))	1,26	1,50	2,76
	ATC(C(D))	1,21	1,40	2,62
	GAT(R(D))	1,50	1,26	2,76
TAA	TAA(D)	1,65	2,56	4,21
	TTA(RC(D))	2,04	2,05	4,09
	ATT(C(D))	2,56	1,65	4,21
	AAT(R(D))	2,05	2,04	4,09
AAC	AAC(D)	1,27	2,22	3,49
	GTT(RC(D))	1,73	1,44	3,18
	TTG(C(D))	2,22	1,27	3,49
	CAA(R(D))	1,44	1,73	3,18
AAG	AAG(D)	1,82	2,34	4,16
	CTT(RC(D))	2,31	2,05	4,35
	TTC(C(D))	2,34	1,82	4,16
	GAA(R(D))	2,05	2,31	4,35
ACA	ACA(D)	0,81	1,36	2,18
	TGT(RC(D))	1,36	0,81	2,18
	TGT(C(D))	1,36	0,81	2,18
	ACA(R(D))	0,81	1,36	2,18
ATA	ATA(D)	0,82	0,97	1,79
	TAT(RC(D))	0,97	0,82	1,79
	TAT(C(D))	0,97	0,82	1,79
	ATA(R(D))	0,82	0,82	1,63
AGA	AGA(D)	1,02	1,34	2,36
	TCT(RC(D))	1,34	1,02	2,36
	TCT(C(D))	1,34	1,02	2,36
	AGA(R(D))	1,02	1,34	2,36
AAA	AAA(D)	1,37	2,13	3,50
	TTT(RC(D))	2,13	1,37	3,50
	TTT(C(D))	2,13	1,37	3,50
	AAA(R(D))	1,37	2,13	3,50

Composition of DNA sequence in NBPF14

Base	No.of bases	%
A	40027	26,76%
C	29696	19,85%
G	32244	21,56%
T	47600	31,83%
N	0	0,00%
Sum	149567	100,00%

% of coding bases from NBPF14 (including N bases)

36,00%

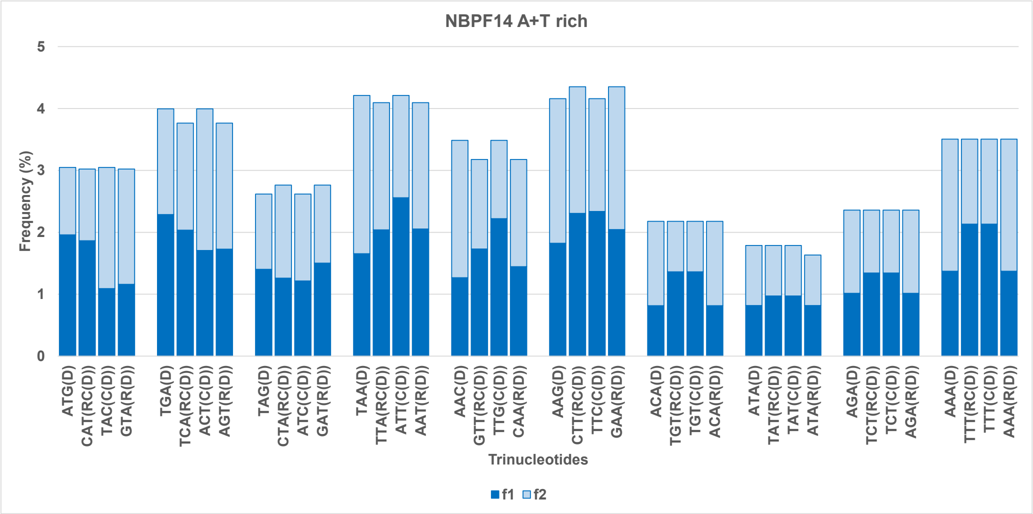

NBPF14 exons

A-T rich

D	f	%	RC	f	%	C	f	%	R	f	%	
**ATG**	216,00	2,00	CAT	161,00	1,49	TAC	80,00	0,74	GTA	74,00	0,69	Ia
**TGA**	264,00	2,45	TCA	250,00	2,32	ACT	206,00	1,91	AGT	172,00	1,60	
**TAG**	93,00	0,86	CTA	58,00	0,54	ATC	90,00	0,84	GAT	153,00	1,42	
**TAA**	44,00	0,41	TTA	138,00	1,28	ATT	156,00	1,45	AAT	95,00	0,88	Ib
**AAC**	144,00	1,34	GTT	161,00	1,49	TTG	262,00	2,43	CAA	160,00	1,48	
**AAG**	448,00	4,16	CTT	219,00	2,03	TTC	136,00	1,26	GAA	483,00	4,48	
**ACA**	164,00	1,52	TGT	167,00	1,55	TGT*			ACA*			Ic
**ATA**	68,00	0,63	TAT	121,00	1,12	TAT*			ATA*			
**AGA**	402,00	3,73	TCT	157,00	1,46	TCT*			AGA*			
**AAA**	317,00	2,94	TTT	159,00	1,48	TTT*			AAA*			

Quadruplet		f1	f2	fu
ATG	ATG(D)	2,00	0,74	2,75
	CAT(RC(D))	1,49	0,69	2,18
	TAC(C(D))	0,74	2,00	2,75
	GTA(R(D))	0,69	1,49	2,18
TGA	TGA(D)	2,45	1,91	4,36
	TCA(RC(D))	2,32	1,60	3,92
	ACT(C(D))	1,91	2,45	4,36
	AGT(R(D))	1,60	2,32	3,92
TAG	TAG(D)	0,86	0,84	1,70
	CTA(RC(D))	0,54	1,42	1,96
	ATC(C(D))	0,84	0,86	1,70
	GAT(R(D))	1,42	0,54	1,96
TAA	TAA(D)	0,41	1,45	1,86
	TTA(RC(D))	1,28	0,88	2,16
	ATT(C(D))	1,45	0,41	1,86
	AAT(R(D))	0,88	1,28	2,16
AAC	AAC(D)	1,34	2,43	3,77
	GTT(RC(D))	1,49	1,48	2,98
	TTG(C(D))	2,43	1,34	3,77
	CAA(R(D))	1,48	1,49	2,98
AAG	AAG(D)	4,16	1,26	5,42
	CTT(RC(D))	2,03	4,48	6,51
	TTC(C(D))	1,26	4,16	5,42
	GAA(R(D))	4,48	2,03	6,51
ACA	ACA(D)	0,76	0,77	1,54
	TGT(RC(D))	0,77	0,76	1,54
	TGT(C(D))	0,77	0,76	1,54
	ACA(R(D))	0,76	0,77	1,54
ATA	ATA(D)	0,32	0,56	0,88
	TAT(RC(D))	0,56	0,32	0,88
	TAT(C(D))	0,56	0,32	0,88
	ATA(R(D))	0,32	0,32	0,63
AGA	AGA(D)	1,87	0,73	2,59
	TCT(RC(D))	0,73	1,87	2,59
	TCT(C(D))	0,73	1,87	2,59
	AGA(R(D))	1,87	0,73	2,59
AAA	AAA(D)	1,47	0,74	2,21
	TTT(RC(D))	0,74	1,47	2,21
	TTT(C(D))	0,74	1,47	2,21
	AAA(R(D))	1,47	0,74	2,21

Composition of DNA sequence in NBPF14 exons

Base	No.of bases	%
A	3188	29,58%
C	2268	21,04%
G	2864	26,57%
T	2459	22,81%
N	0	0,00%
Sum	10779	100,00%

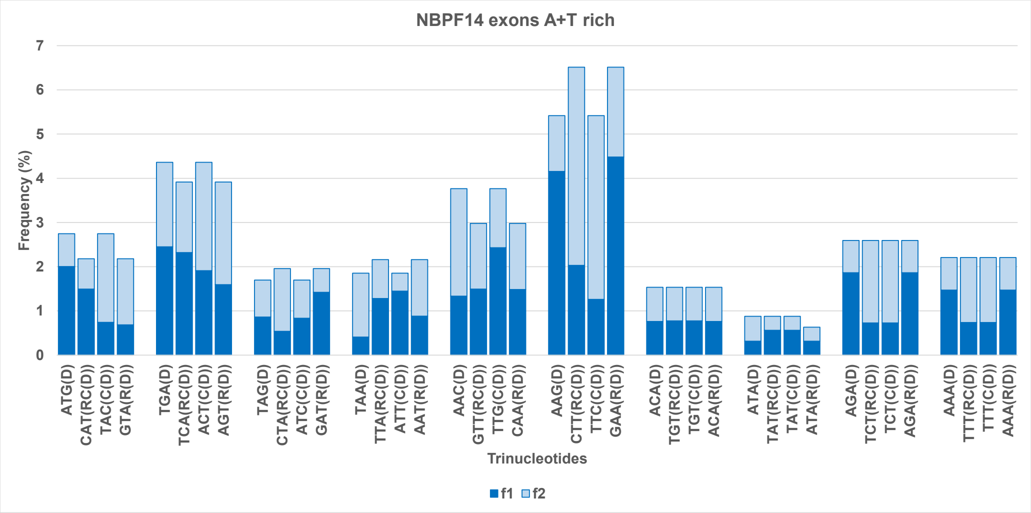

NBPF14 introns

A-T rich												
**D**	**f**	**%**	**RC**	**f**	**%**	**C**	**f**	**%**	**R**	**f**	**%**	
**ATG**	1107,00	2,06	CAT	1124,00	2,09	TAC	571,00	1,06	GTA	501,00	0,93	Ia
**TGA**	1421,00	2,64	TCA	1223,00	2,27	ACT	949,00	1,76	AGT	809,00	1,50	
**TAG**	630,00	1,17	CTA	761,00	1,41	ATC	708,00	1,31	GAT	848,00	1,57	
**TAA**	664,00	1,23	TTA	849,00	1,58	ATT	1378,00	2,56	AAT	975,00	1,81	Ib
**AAC**	692,00	1,29	GTT	889,00	1,65	TTG	1149,00	2,13	CAA	747,00	1,39	
**AAG**	647,00	1,20	CTT	1190,00	2,21	TTC	1459,00	2,71	GAA	903,00	1,68	
**ACA**	804,00	1,49	TGT	1838,00	3,41	TGT*			ACA*			Ic
**ATA**	650,00	1,21	TAT	896,00	1,66	TAT*			ATA*			
**AGA**	814,00	1,51	TCT	1807,00	3,36	TCT*			AGA*			
**AAA**	1080,00	2,01	TTT	2044,00	3,80	TTT*			AAA*			

Quadruplet		f1	f2	fu
ATG	ATG(D)	2,06	1,06	3,12
	CAT(RC(D))	2,09	0,93	3,02
	TAC(C(D))	1,06	2,06	3,12
	GTA(R(D))	0,93	2,09	3,02
TGA	TGA(D)	2,64	1,76	4,40
	TCA(RC(D))	2,27	1,50	3,77
	ACT(C(D))	1,76	2,64	4,40
	AGT(R(D))	1,50	2,27	3,77
TAG	TAG(D)	1,17	1,31	2,48
	CTA(RC(D))	1,41	1,57	2,99
	ATC(C(D))	1,31	1,17	2,48
	GAT(R(D))	1,57	1,41	2,99
TAA	TAA(D)	1,23	2,56	3,79
	TTA(RC(D))	1,58	1,81	3,39
	ATT(C(D))	2,56	1,23	3,79
	AAT(R(D))	1,81	1,58	3,39
AAC	AAC(D)	1,29	2,13	3,42
	GTT(RC(D))	1,65	1,39	3,04
	TTG(C(D))	2,13	1,29	3,42
	CAA(R(D))	1,39	1,65	3,04
AAG	AAG(D)	1,20	2,71	3,91
	CTT(RC(D))	2,21	1,68	3,89
	TTC(C(D))	2,71	1,20	3,91
	GAA(R(D))	1,68	2,21	3,89
ACA	ACA(D)	0,75	1,71	2,45
	TGT(RC(D))	1,71	0,75	2,45
	TGT(C(D))	1,71	0,75	2,45
	ACA(R(D))	0,75	1,71	2,45
ATA	ATA(D)	0,60	0,83	1,44
	TAT(RC(D))	0,83	0,60	1,44
	TAT(C(D))	0,83	0,60	1,44
	ATA(R(D))	0,60	0,60	1,21
AGA	AGA(D)	0,76	1,68	2,43
	TCT(RC(D))	1,68	0,76	2,43
	TCT(C(D))	1,68	0,76	2,43
	AGA(R(D))	0,76	1,68	2,43
AAA	AAA(D)	1,00	1,90	2,90
	TTT(RC(D))	1,90	1,00	2,90
	TTT(C(D))	1,90	1,00	2,90
	AAA(R(D))	1,00	1,90	2,90

Composition of DNA sequence NBPF14 introns

Base	No.of bases	%
A	13019	24,177%
C	11720	21,765%
G	11634	21,605%
T	17475	32,452%
N	1	0,002%
Sum	53849	100,00%

4

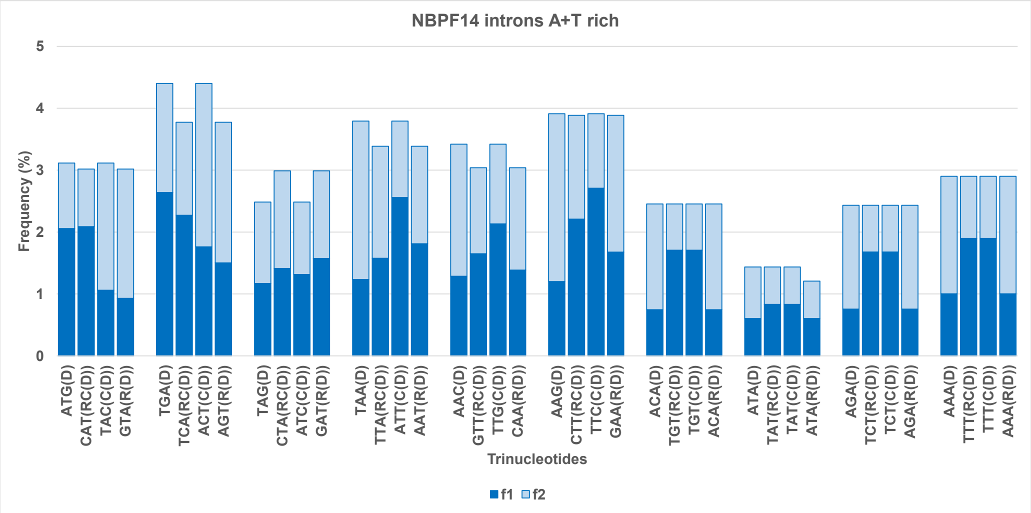

NBPF14 sequence

C+G rich												
**D**	**f**	**%**	**RC**	**f**	**%**	**C**	**f**	**%**	**R**	**f**	**%**	
**CGT**	344,00	0,23	ACG	304,00	0,20	GCA	1964,00	1,31	TGC	2002,00	1,34	IIa
**GTC**	1700,00	1,14	GAC	1548,00	1,04	CAG	2948,00	1,97	CTG	3553,00	2,38	
**GCT**	2050,00	1,37	AGC	1895,00	1,27	CGA	329,00	0,22	TCG	298,00	0,20	
**GCC**	1579,00	1,06	GGC	1733,00	1,16	CGG	259,00	0,17	CCG	293,00	0,20	IIb
**CCA**	2293,00	1,53	TGG	3053,00	2,04	GGT	1769,00	1,18	ACC	1623,00	1,09	
**CCT**	2945,00	1,97	AGG	2788,00	1,86	GGA	2598,00	1,74	TCC	2330,00	1,56	
**CAC**	1844,00	1,23	GTG	2751,00	1,84	GTG*			CAC*			IIc
**CGC**	280,00	0,19	GCG	317,00	0,21	GCG*			CGC*			
**CTC**	2678,00	1,79	GAG	2532,00	1,69	GAG*			CTC*			
**CCC**	1648,00	1,10	GGG	2074,00	1,39	GGG*			CCC*			

Quadruplet		f1	f2	fu
CGT	CGT(D)	0,23	1,31	1,54
	ACG(RC(D))	0,20	1,34	1,54
	GCA(C(D))	1,31	0,23	1,54
	TGC(R(D))	1,34	0,20	1,54
GTC	GTC(D)	1,14	1,97	3,11
	GAC(RC(D))	1,04	2,38	3,41
	CAG(C(D))	1,97	1,14	3,11
	CTG(R(D))	2,38	1,04	3,41
GCT	GCT(D)	1,37	0,22	1,59
	AGC(RC(D))	1,27	0,20	1,47
	CGA(C(D))	0,22	1,37	1,59
	TCG(R(D))	0,20	1,27	1,47
GCC	GCC(D)	1,06	0,17	1,23
	GGC(RC(D))	1,16	0,20	1,35
	CGG(C(D))	0,17	1,06	1,23
	CCG(R(D))	0,20	1,16	1,35
CCA	CCA(D)	1,53	1,18	2,72
	TGG(RC(D))	2,04	1,09	3,13
	GGT(C(D))	1,18	1,53	2,72
	ACC(R(D))	1,09	2,04	3,13
CCT	CCT(D)	1,97	1,74	3,71
	AGG(RC(D))	1,86	1,56	3,42
	GGA(C(D))	1,74	1,97	3,71
	TCC(R(D))	1,56	1,86	3,42
CAC	CAC(D)	0,62	0,92	1,54
	GTG(RC(D))	0,92	0,62	1,54
	GTG(C(D))	0,92	0,62	1,54
	CAC(R(D))	0,62	0,92	1,54
CGC	CGC(D)	0,09	0,11	0,20
	GCG(RC(D))	0,11	0,09	0,20
	GCG(C(D))	0,11	0,09	0,20
	CGC(R(D))	0,09	0,11	0,20
CTC	CTC(D)	0,90	0,85	1,74
	GAG(RC(D))	0,85	0,90	1,74
	GAG(C(D))	0,85	0,90	1,74
	CTC(R(D))	0,90	0,85	1,74
CCC	CCC(D)	0,55	0,69	1,24
	GGG(RC(D))	0,69	0,55	1,24
	GGG(C(D))	0,69	0,55	1,24
	CCC(R(D))	0,55	0,69	1,24

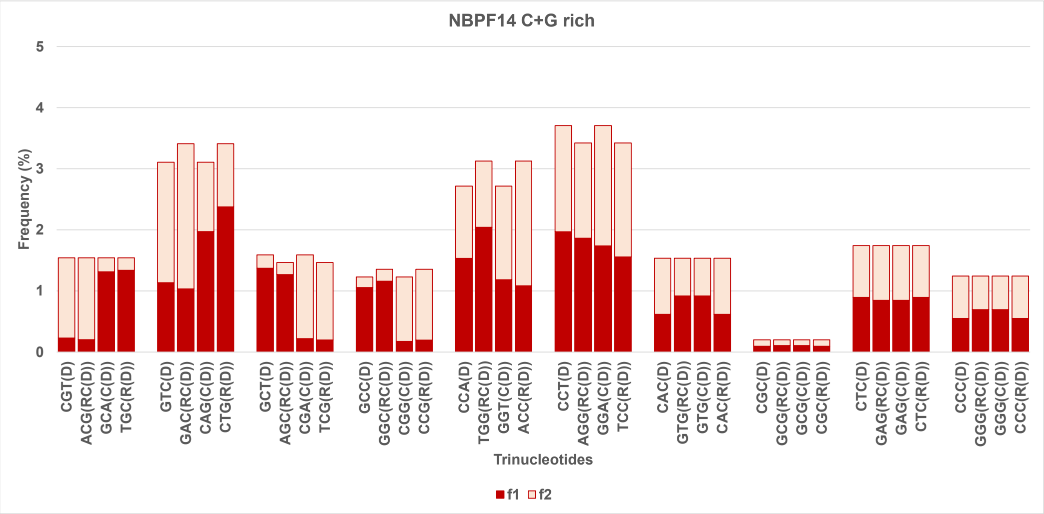

NBPF14 exons

C+G rich

D	f	%	RC	f	%	C	f	%	R	f	%		
**CGT**	39,00	0,36	ACG	44,00	0,41	GCA	148,00	1,37	TGC	194,00	1,80	IIa	86356
**GTC**	105,00	0,97	GAC	211,00	1,96	CAG	350,00	3,25	CTG	284,00	2,64		80741
**GCT**	202,00	1,87	AGC	212,00	1,97	CGA	31,00	0,29	TCG	19,00	0,18		44919
**GCC**	195,00	1,81	GGC	149,00	1,38	CGG	18,00	0,17	CCG	15,00	0,14	IIb	22459
**CCA**	214,00	1,99	TGG	248,00	2,30	GGT	73,00	0,68	ACC	125,00	1,16		45985
**CCT**	218,00	2,02	AGG	328,00	3,04	GGA	372,00	3,45	TCC	127,00	1,18		57215
**CAC**	104,00	0,97	GTG	111,00	1,03	GTG*			CAC*			IIc	
**CGC**	11,00	0,10	GCG	21,00	0,19	GCG*			CGC*				
**CTC**	222,00	2,06	GAG	222,00	2,06	GAG*			CTC*				
**CCC**	163,00	1,51	GGG	184,00	1,71	GGG*			CCC*				

Quadruplet		f1	f2	fu
CGT	CGT(D)	0,36	1,37	1,74
	ACG(RC(D))	0,41	1,80	2,21
	GCA(C(D))	1,37	0,36	1,74
	TGC(R(D))	1,80	0,41	2,21
GTC	GTC(D)	0,97	3,25	4,22
	GAC(RC(D))	1,96	2,64	4,59
	CAG(C(D))	3,25	0,97	4,22
	CTG(R(D))	2,64	1,96	4,59
GCT	GCT(D)	1,87	0,29	2,16
	AGC(RC(D))	1,97	0,18	2,14
	CGA(C(D))	0,29	1,87	2,16
	TCG(R(D))	0,18	1,97	2,14
GCC	GCC(D)	1,81	0,17	1,98
	GGC(RC(D))	1,38	0,14	1,52
	CGG(C(D))	0,17	1,81	1,98
	CCG(R(D))	0,14	1,38	1,52
CCA	CCA(D)	1,99	0,68	2,66
	TGG(RC(D))	2,30	1,16	3,46
	GGT(C(D))	0,68	1,99	2,66
	ACC(R(D))	1,16	2,30	3,46
CCT	CCT(D)	2,02	3,45	5,47
	AGG(RC(D))	3,04	1,18	4,22
	GGA(C(D))	3,45	2,02	5,47
	TCC(R(D))	1,18	3,04	4,22
CAC	CAC(D)	0,48	0,51	1,00
	GTG(RC(D))	0,51	0,48	1,00
	GTG(C(D))	0,51	0,48	1,00
	CAC(R(D))	0,48	0,51	1,00
CGC	CGC(D)	0,05	0,10	0,15
	GCG(RC(D))	0,10	0,05	0,15
	GCG(C(D))	0,10	0,05	0,15
	CGC(R(D))	0,05	0,10	0,15
CTC	CTC(D)	1,03	1,03	2,06
	GAG(RC(D))	1,03	1,03	2,06
	GAG(C(D))	1,03	1,03	2,06
	CTC(R(D))	1,03	1,03	2,06
CCC	CCC(D)	0,76	0,85	1,61
	GGG(RC(D))	0,85	0,76	1,61
	GGG(C(D))	0,85	0,76	1,61
	CCC(R(D))	0,76	0,85	1,61

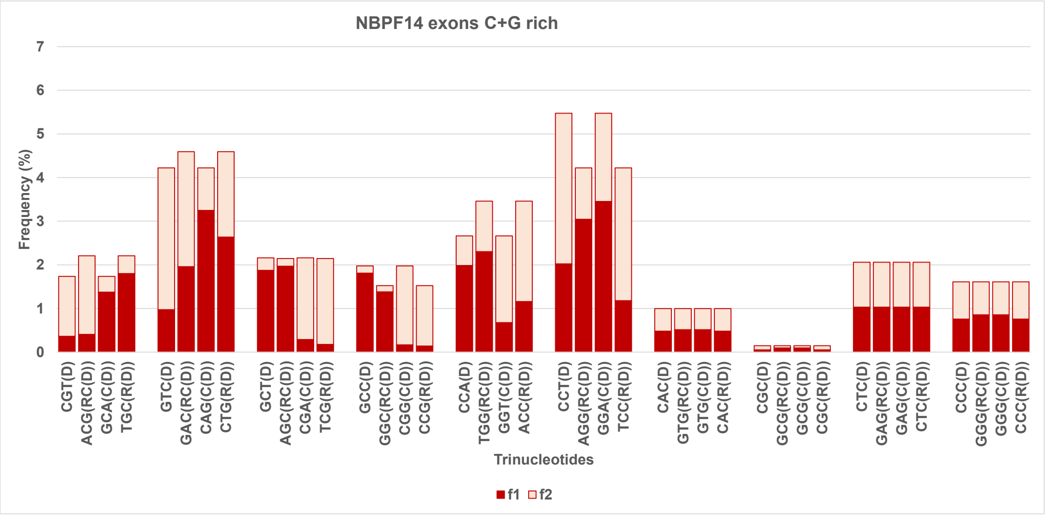

NBPF14 introns

C+G rich

D	f	%	RC	f	%	C	f	%	R	f	%	
**CGT**	124,00	0,23	ACG	110,00	0,20	GCA	734,00	1,36	TGC	613,00	1,14	IIa
**GTC**	806,00	1,50	GAC	591,00	1,10	CAG	1080,00	2,01	CTG	1544,00	2,87	
**GCT**	679,00	1,26	AGC	616,00	1,14	CGA	155,00	0,29	TCG	114,00	0,21	
**GCC**	527,00	0,98	GGC	719,00	1,34	CGG	50,00	0,09	CCG	97,00	0,18	IIb
**CCA**	859,00	1,60	TGG	1116,00	2,07	GGT	611,00	1,13	ACC	660,00	1,23	
**CCT**	1312,00	2,44	AGG	1018,00	1,89	GGA	854,00	1,59	TCC	1081,00	2,01	
**CAC**	669,00	1,24	GTG	1188,00	2,21	GTG*			CAC*			IIc
**CGC**	97,00	0,18	GCG	105,00	0,20	GCG*			CGC*			
**CTC**	1252,00	2,33	GAG	902,00	1,68	GAG*			CTC*			
**CCC**	659,00	1,22	GGG	775,00	1,44	GGG*			CCC*			

Quadruplet		f1	f2	fu
CGT	CGT(D)	0,23	1,36	1,59
	ACG(RC(D))	0,20	1,14	1,34
	GCA(C(D))	1,36	0,23	1,59
	TGC(R(D))	1,14	0,20	1,34
GTC	GTC(D)	1,50	2,01	3,50
	GAC(RC(D))	1,10	2,87	3,97
	CAG(C(D))	2,01	1,50	3,50
	CTG(R(D))	2,87	1,10	3,97
GCT	GCT(D)	1,26	0,29	1,55
	AGC(RC(D))	1,14	0,21	1,36
	CGA(C(D))	0,29	1,26	1,55
	TCG(R(D))	0,21	1,14	1,36
GCC	GCC(D)	0,98	0,09	1,07
	GGC(RC(D)	1,34	0,18	1,52
	CGG(C(D))	0,09	0,98	1,07
	CCG(R(D))	0,18	1,34	1,52
CCA	CCA(D)	1,60	1,13	2,73
	TGG(RC(D))	2,07	1,23	3,30
	GGT(C(D))	1,13	1,60	2,73
	ACC(R(D))	1,23	2,07	3,30
CCT	CCT(D)	2,44	1,59	4,02
	AGG(RC(D))	1,89	2,01	3,90
	GGA(C(D))	1,59	2,44	4,02
	TCC(R(D))	2,01	1,89	3,90
CAC	CAC(D)	0,62	1,10	1,72
	GTG(RC(D))	1,10	0,62	1,72
	GTG(C(D))	1,10	0,62	1,72
	CAC(R(D))	0,62	1,10	1,72
CGC	CGC(D)	0,09	0,10	0,19
	GCG(RC(D)	0,10	0,09	0,19
	GCG(C(D))	0,10	0,09	0,19
	CGC(R(D))	0,09	0,10	0,19
CTC	CTC(D)	1,16	0,84	2,00
	GAG(RC(D))	0,84	1,16	2,00
	GAG(C(D))	0,84	1,16	2,00
	CTC(R(D))	1,16	0,84	2,00
CCC	CCC(D)	0,61	0,72	1,33
	GGG(RC(D)	0,72	0,61	1,33
	GGG(C(D))	0,72	0,61	1,33
	CCC(R(D))	0,61	0,72	1,33

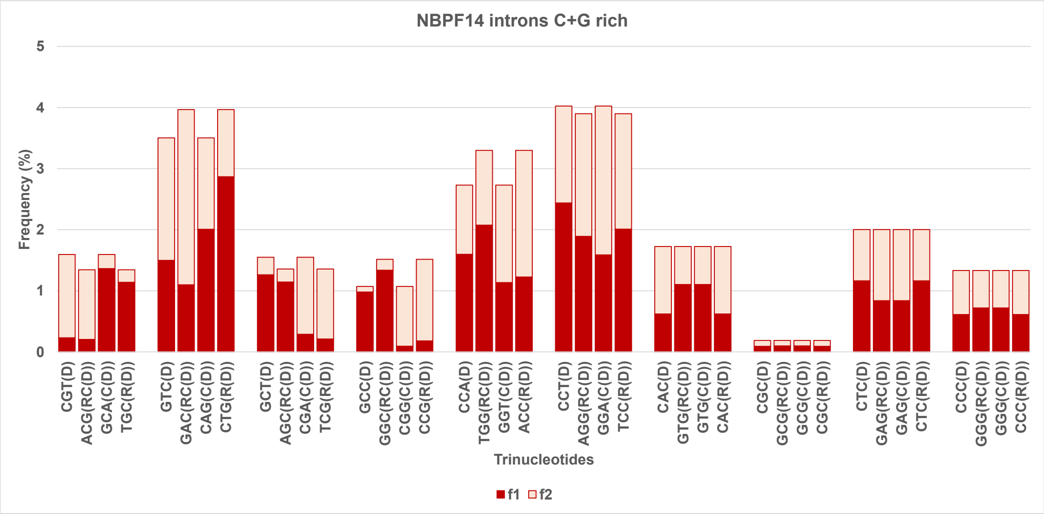

NBPF15 sequence

						A-T rich						
**D**	**f**	**%**	**RC**	**f**	**%**	**C**	**f**	**%**	**R**	**f**	**%**	
**ATG**	811,00	1,95	CAT	796,00	1,92	TAC	443,00	1,07	GTA	457,00	1,10	Ia
**TGA**	880,00	2,12	TCA	839,00	2,02	ACT	603,00	1,45	AGT	685,00	1,65	
**TAG**	498,00	1,20	CTA	470,00	1,13	ATC	528,00	1,27	GAT	560,00	1,35	
**TAA**	635,00	1,53	TTA	739,00	1,78	ATT	961,00	2,32	AAT	855,00	2,06	Ib
**AAC**	494,00	1,19	GTT	717,00	1,73	TTG	892,00	2,15	CAA	618,00	1,49	
**AAG**	693,00	1,67	CTT	888,00	2,14	TTC	935,00	2,25	GAA	789,00	1,90	
**ACA**	722,00	1,74	TGT	1174,00	2,83	TGT*			ACA*			Ic
**ATA**	710,00	1,71	TAT	800,00	1,93	TAT*			ATA*			
**AGA**	792,00	1,91	TCT	1099,00	2,65	TCT*			AGA*			
**AAA**	1131,00	2,73	TTT	1614,00	3,89	TTT*			AAA*			

Quadruplet		f1	f2	fu
ATG	ATG(D)	1,95	1,07	3,02
	CAT(RC(D))	1,92	1,10	3,02
	TAC(C(D))	1,07	1,95	3,02
	GTA(R(D))	1,10	1,92	3,02
TGA	TGA(D)	2,12	1,45	3,57
	TCA(RC(D))	2,02	1,65	3,67
	ACT(C(D))	1,45	2,12	3,57
	AGT(R(D))	1,65	2,02	3,67
TAG	TAG(D)	1,20	1,27	2,47
	CTA(RC(D))	1,13	1,35	2,48
	ATC(C(D))	1,27	1,20	2,47
	GAT(R(D))	1,35	1,13	2,48
TAA	TAA(D)	1,53	2,32	3,85
	TTA(RC(D))	1,78	2,06	3,84
	ATT(C(D))	2,32	1,53	3,85
	AAT(R(D))	2,06	1,78	3,84
AAC	AAC(D)	1,19	2,15	3,34
	GTT(RC(D))	1,73	1,49	3,22
	TTG(C(D))	2,15	1,19	3,34
	CAA(R(D))	1,49	1,73	3,22
AAG	AAG(D)	1,67	2,25	3,92
	CTT(RC(D))	2,14	1,90	4,04
	TTC(C(D))	2,25	1,67	3,92
	GAA(R(D))	1,90	2,14	4,04
ACA	ACA(D)	0,87	1,41	2,29
	TGT(RC(D))	1,41	0,87	2,29
	TGT(C(D))	1,41	0,87	2,29
	ACA(R(D))	0,87	1,41	2,29
ATA	ATA(D)	0,86	0,96	1,82
	TAT(RC(D))	0,96	0,86	1,82
	TAT(C(D))	0,96	0,86	1,82
	ATA(R(D))	0,86	0,86	1,71
AGA	AGA(D)	0,95	1,32	2,28
	TCT(RC(D))	1,32	0,95	2,28
	TCT(C(D))	1,32	0,95	2,28
	AGA(R(D))	0,95	1,32	2,28
AAA	AAA(D)	1,36	1,95	3,31
	TTT(RC(D))	1,95	1,36	3,31
	TTT(C(D))	1,95	1,36	3,31
	AAA(R(D))	1,36	1,95	3,31

Composition of DNA sequence in NBPF15

Base	No.of bases	%
A	10836	26,12%
C	8782	21,17%
G	9017	21,73%
T	12854	30,98%
N	0	0,00%
Sum	41489	100,00%

% of coding bases from NBPF15 (including N bases)

74,04%

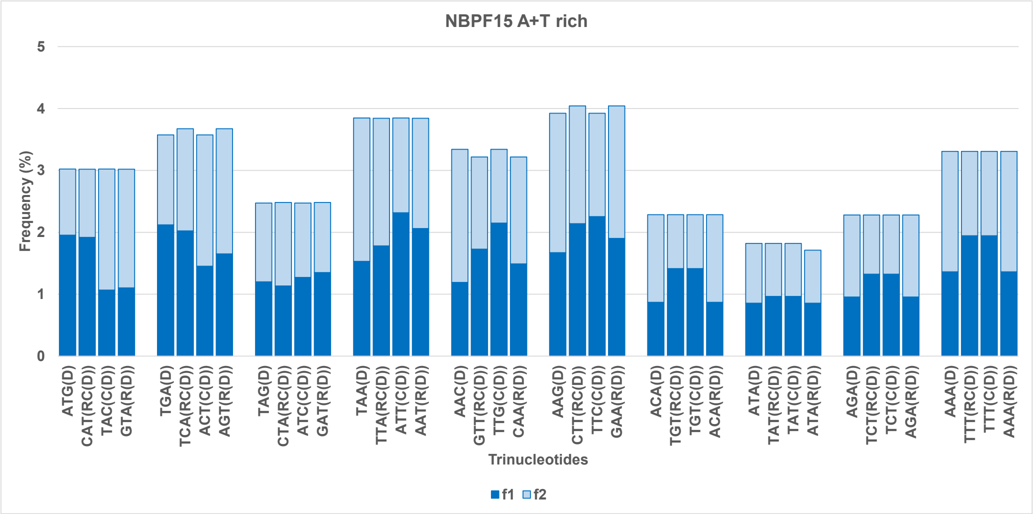

NBPF15 exons

A-T rich

D	f	%	RC	f	%	C	f	%	R	f	%	
**ATG**	98,00	2,08	CAT	87,00	1,85	TAC	45,00	0,96	GTA	32,00	0,68	Ia
**TGA**	119,00	2,53	TCA	113,00	2,40	ACT	70,00	1,49	AGT	77,00	1,64	
**TAG**	41,00	0,87	CTA	33,00	0,70	ATC	57,00	1,21	GAT	71,00	1,51	
**TAA**	39,00	0,83	TTA	66,00	1,40	ATT	85,00	1,81	AAT	75,00	1,59	Ib
**AAC**	66,00	1,40	GTT	74,00	1,57	TTG	95,00	2,02	CAA	91,00	1,93	
**AAG**	127,00	2,70	CTT	74,00	1,57	TTC	71,00	1,51	GAA	138,00	2,93	
**ACA**	97,00	2,06	TGT	91,00	1,93	TGT*			ACA*			Ic
**ATA**	51,00	1,08	TAT	57,00	1,21	TAT*			ATA*			
**AGA**	133,00	2,83	TCT	85,00	1,81	TCT*			AGA*			
**AAA**	115,00	2,44	TTT	104,00	2,21	TTT*			AAA*			

Quadruplet		f1	f2	fu
	ATG(D)	2,08	0,96	3,04
ATG	CAT(RC(D))	1,85	0,68	2,53
	TAC(C(D))	0,96	2,08	3,04
	GTA(R(D))	0,68	1,85	2,53
	TGA(D)	2,53	1,49	4,02
TGA	TCA(RC(D))	2,40	1,64	4,04
	ACT(C(D))	1,49	2,53	4,02
	AGT(R(D))	1,64	2,40	4,04
	TAG(D)	0,87	1,21	2,08
TAG	CTA(RC(D))	0,70	1,51	2,21
	ATC(C(D))	1,21	0,87	2,08
	GAT(R(D))	1,51	0,70	2,21
	TAA(D)	0,83	1,81	2,64
TAA	TTA(RC(D))	1,40	1,59	3,00
	ATT(C(D))	1,81	0,83	2,64
	AAT(R(D))	1,59	1,40	3,00
	AAC(D)	1,40	2,02	3,42
AAC	GTT(RC(D))	1,57	1,93	3,51
	TTG(C(D))	2,02	1,40	3,42
	CAA(R(D))	1,93	1,57	3,51
	AAG(D)	2,70	1,51	4,21
AAG	CTT(RC(D))	1,57	2,93	4,51
	TTC(C(D))	1,51	2,70	4,21
	GAA(R(D))	2,93	1,57	4,51
	ACA(D)	1,03	0,97	2,00
ACA	TGT(RC(D))	0,97	1,03	2,00
	TGT(C(D))	0,97	1,03	2,00
	ACA(R(D))	1,03	0,97	2,00
	ATA(D)	0,54	0,61	1,15
ATA	TAT(RC(D))	0,61	0,54	1,15
	TAT(C(D))	0,61	0,54	1,15
	ATA(R(D))	0,54	0,54	1,08
	AGA(D)	1,41	0,90	2,32
AGA	TCT(RC(D))	0,90	1,41	2,32
	TCT(C(D))	0,90	1,41	2,32
	AGA(R(D))	1,41	0,90	2,32
	AAA(D)	1,22	1,11	2,33
AAA	TTT(RC(D))	1,11	1,22	2,33
	TTT(C(D))	1,11	1,22	2,33
	AAA(R(D))	1,22	1,11	2,33

Composition of DNA sequence in NBPF15 exons

Base	No.of bases	%
A	1329	28,23%
C	1041	22,12%
G	1143	24,28%
T	1194	25,37%
N	0	0,00%
Sum	4707	100,00%

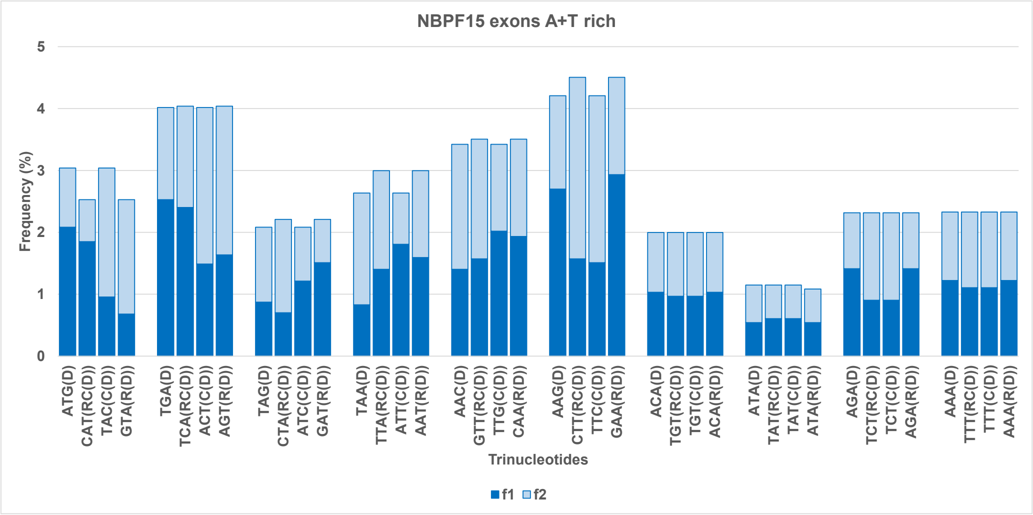

NBPF15 introns

A-T rich												
**D**	**f**	**%**	**RC**	**f**	**%**	**C**	**f**	**%**	**R**	**f**	**%**	
**ATG**	628,00	2,04	CAT	620,00	2,02	TAC	330,00	1,07	GTA	346,00	1,13	Ia
**TGA**	657,00	2,14	TCA	616,00	2,01	ACT	462,00	1,50	AGT	497,00	1,62	
**TAG**	380,00	1,24	CTA	373,00	1,21	ATC	397,00	1,29	GAT	412,00	1,34	
**TAA**	508,00	1,65	TTA	551,00	1,79	ATT	733,00	2,39	AAT	674,00	2,19	Ib
**AAC**	377,00	1,23	GTT	530,00	1,73	TTG	660,00	2,15	CAA	449,00	1,46	
**AAG**	481,00	1,57	CTT	686,00	2,23	TTC	738,00	2,40	GAA	575,00	1,87	
**ACA**	548,00	1,78	TGT	924,00	3,01	TGT*			ACA*			Ic
**ATA**	586,00	1,91	TAT	638,00	2,08	TAT*			ATA*			
**AGA**	571,00	1,86	TCT	873,00	2,84	TCT*			AGA*			
**AAA**	871,00	2,84	TTT	1189,00	3,87	TTT*			AAA*			

Quadruplet		f1	f2	fu
ATG	ATG(D)	2,04	1,07	3,12
	CAT(RC(D))	2,02	1,13	3,15
	TAC(C(D))	1,07	2,04	3,12
	GTA(R(D))	1,13	2,02	3,15
TGA	TGA(D)	2,14	1,50	3,64
	TCA(RC(D))	2,01	1,62	3,62
	ACT(C(D))	1,50	2,14	3,64
	AGT(R(D))	1,62	2,01	3,62
TAG	TAG(D)	1,24	1,29	2,53
	CTA(RC(D))	1,21	1,34	2,56
	ATC(C(D))	1,29	1,24	2,53
	GAT(R(D))	1,34	1,21	2,56
TAA	TAA(D)	1,65	2,39	4,04
	TTA(RC(D))	1,79	2,19	3,99
	ATT(C(D))	2,39	1,65	4,04
	AAT(R(D))	2,19	1,79	3,99
AAC	AAC(D)	1,23	2,15	3,38
	GTT(RC(D))	1,73	1,46	3,19
	TTG(C(D))	2,15	1,23	3,38
	CAA(R(D))	1,46	1,73	3,19
AAG	AAG(D)	1,57	2,40	3,97
	CTT(RC(D))	2,23	1,87	4,11
	TTC(C(D))	2,40	1,57	3,97
	GAA(R(D))	1,87	2,23	4,11
ACA	ACA(D)	0,89	1,50	2,40
	TGT(RC(D))	1,50	0,89	2,40
	TGT(C(D))	1,50	0,89	2,40
	ACA(R(D))	0,89	1,50	2,40
ATA	ATA(D)	0,95	1,04	1,99
	TAT(RC(D))	1,04	0,95	1,99
	TAT(C(D))	1,04	0,95	1,99
	ATA(R(D))	0,95	0,95	1,91
AGA	AGA(D)	0,93	1,42	2,35
	TCT(RC(D))	1,42	0,93	2,35
	TCT(C(D))	1,42	0,93	2,35
	AGA(R(D))	0,93	1,42	2,35
AAA	AAA(D)	1,42	1,94	3,35
	TTT(RC(D))	1,94	1,42	3,35
	TTT(C(D))	1,94	1,42	3,35
	AAA(R(D))	1,42	1,94	3,35

Composition of DNA sequence NBPF15 introns

Base	No.of bases	%
A	8111	26,41%
C	6416	20,89%
G	6440	20,97%
T	9750	31,74%
N	0	0,00%
Sum	30717	100,00%

4

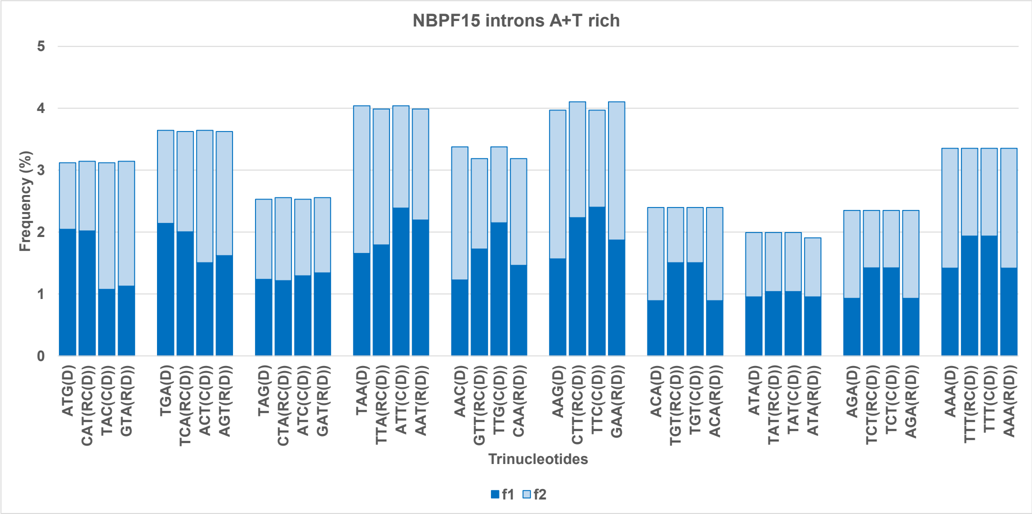

NBPF15 sequence

C+G rich												
**D**	**f**	**%**	**RC**	**f**	**%**	**C**	**f**	**%**	**R**	**f**	**%**	
**CGT**	143,00	0,34	ACG	105,00	0,25	GCA	561,00	1,35	TGC	603,00	1,45	IIa
**GTC**	518,00	1,25	GAC	413,00	1,00	CAG	845,00	2,04	CTG	990,00	2,39	
**GCT**	585,00	1,41	AGC	502,00	1,21	CGA	111,00	0,27	TCG	96,00	0,23	
**GCC**	470,00	1,13	GGC	550,00	1,33	CGG	126,00	0,30	CCG	140,00	0,34	IIb
**CCA**	723,00	1,74	TGG	849,00	2,05	GGT	503,00	1,21	ACC	507,00	1,22	
**CCT**	871,00	2,10	AGG	736,00	1,77	GGA	658,00	1,59	TCC	757,00	1,82	
**CAC**	587,00	1,41	GTG	813,00	1,96	GTG*			CAC*			IIc
**CGC**	100,00	0,24	GCG	139,00	0,34	GCG*			CGC*			
**CTC**	810,00	1,95	GAG	679,00	1,64	GAG*			CTC*			
**CCC**	564,00	1,36	GGG	605,00	1,46	GGG*			CCC*			

Quadruplet		f1	f2	fu
	CGT(D)	0,34	1,35	1,70
CGT	ACG(RC(D))	0,25	1,45	1,71
	GCA(C(D))	1,35	0,34	1,70
	TGC(R(D))	1,45	0,25	1,71
	GTC(D)	1,25	2,04	3,29
GTC	GAC(RC(D))	1,00	2,39	3,38
	CAG(C(D))	2,04	1,25	3,29
	CTG(R(D))	2,39	1,00	3,38
	GCT(D)	1,41	0,27	1,68
GCT	AGC(RC(D))	1,21	0,23	1,44
	CGA(C(D))	0,27	1,41	1,68
	TCG(R(D))	0,23	1,21	1,44
	GCC(D)	1,13	0,30	1,44
GCC	GGC(RC(D)	1,33	0,34	1,66
	CGG(C(D))	0,30	1,13	1,44
	CCG(R(D))	0,34	1,33	1,66
	CCA(D)	1,74	1,21	2,96
CCA	TGG(RC(D))	2,05	1,22	3,27
	GGT(C(D))	1,21	1,74	2,96
	ACC(R(D))	1,22	2,05	3,27
	CCT(D)	2,10	1,59	3,69
CCT	AGG(RC(D))	1,77	1,82	3,60
	GGA(C(D))	1,59	2,10	3,69
	TCC(R(D))	1,82	1,77	3,60
	CAC(D)	0,71	0,98	1,69
CAC	GTG(RC(D))	0,98	0,71	1,69
	GTG(C(D))	0,98	0,71	1,69
	CAC(R(D))	0,71	0,98	1,69
	CGC(D)	0,12	0,17	0,29
CGC	GCG(RC(D)	0,17	0,12	0,29
	GCG(C(D))	0,17	0,12	0,29
	CGC(R(D))	0,12	0,17	0,29
	CTC(D)	0,98	0,82	1,79
CTC	GAG(RC(D))	0,82	0,98	1,79
	GAG(C(D))	0,82	0,98	1,79
	CTC(R(D))	0,98	0,82	1,79
	CCC(D)	0,68	0,73	1,41
CCC	GGG(RC(D)	0,73	0,68	1,41
	GGG(C(D))	0,73	0,68	1,41
	CCC(R(D))	0,68	0,73	1,41

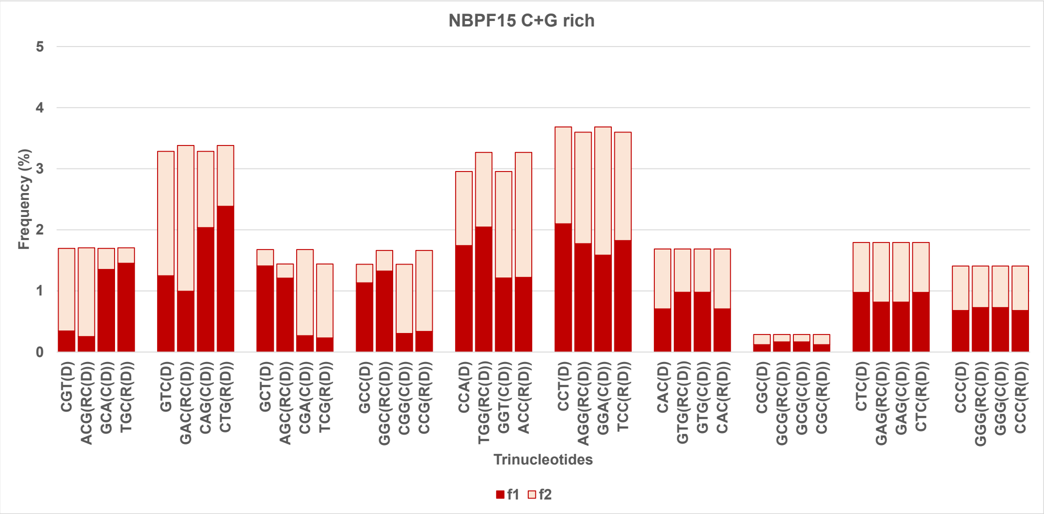

NBPF15 exons

C+G rich

D	f	%	RC	f	%	C	f	%	R	f	%		
**CGT**	17,00	0,36	ACG	22,00	0,47	GCA	67,00	1,42	TGC	72,00	1,53	IIa	86356
**GTC**	65,00	1,38	GAC	77,00	1,64	CAG	135,00	2,87	CTG	122,00	2,59		80741
**GCT**	75,00	1,59	AGC	80,00	1,70	CGA	18,00	0,38	TCG	10,00	0,21		44919
**GCC**	69,00	1,47	GGC	62,00	1,32	CGG	18,00	0,38	CCG	18,00	0,38	IIb	22459
**CCA**	100,00	2,13	TGG	103,00	2,19	GGT	56,00	1,19	ACC	63,00	1,34		45985
**CCT**	95,00	2,02	AGG	113,00	2,40	GGA	116,00	2,47	TCC	81,00	1,72		57215
**CAC**	64,00	1,36	GTG	70,00	1,49	GTG*			CAC*			IIc	
**CGC**	10,00	0,21	GCG	13,00	0,28	GCG*			CGC*				
**CTC**	96,00	2,04	GAG	100,00	2,13	GAG*			CTC*				
**CCC**	63,00	1,34	GGG	58,00	1,23	GGG*			CCC*				

Quadruplet		f1	f2	fu
CGT	CGT(D)	0,36	1,42	1,79
	ACG(RC(D))	0,47	1,53	2,00
	GCA(C(D))	1,42	0,36	1,79
	TGC(R(D))	1,53	0,47	2,00
GTC	GTC(D)	1,38	2,87	4,25
	GAC(RC(D))	1,64	2,59	4,23
	CAG(C(D))	2,87	1,38	4,25
	CTG(R(D))	2,59	1,64	4,23
GCT	GCT(D)	1,59	0,38	1,98
	AGC(RC(D))	1,70	0,21	1,91
	CGA(C(D))	0,38	1,59	1,98
	TCG(R(D))	0,21	1,70	1,91
GCC	GCC(D)	1,47	0,38	1,85
	GGC(RC(D))	1,32	0,38	1,70
	CGG(C(D))	0,38	1,47	1,85
	CCG(R(D))	0,38	1,32	1,70
CCA	CCA(D)	2,13	1,19	3,32
	TGG(RC(D))	2,19	1,34	3,53
	GGT(C(D))	1,19	2,13	3,32
	ACC(R(D))	1,34	2,19	3,53
CCT	CCT(D)	2,02	2,47	4,48
	AGG(RC(D))	2,40	1,72	4,12
	GGA(C(D))	2,47	2,02	4,48
	TCC(R(D))	1,72	2,40	4,12
CAC	CAC(D)	0,68	0,74	1,42
	GTG(RC(D))	0,74	0,68	1,42
	GTG(C(D))	0,74	0,68	1,42
	CAC(R(D))	0,68	0,74	1,42
CGC	CGC(D)	0,11	0,14	0,24
	GCG(RC(D))	0,14	0,11	0,24
	GCG(C(D))	0,14	0,11	0,24
	CGC(R(D))	0,11	0,14	0,24
CTC	CTC(D)	1,02	1,06	2,08
	GAG(RC(D))	1,06	1,02	2,08
	GAG(C(D))	1,06	1,02	2,08
	CTC(R(D))	1,02	1,06	2,08
CCC	CCC(D)	0,67	0,62	1,29
	GGG(RC(D))	0,62	0,67	1,29
	GGG(C(D))	0,62	0,67	1,29
	CCC(R(D))	0,67	0,62	1,29

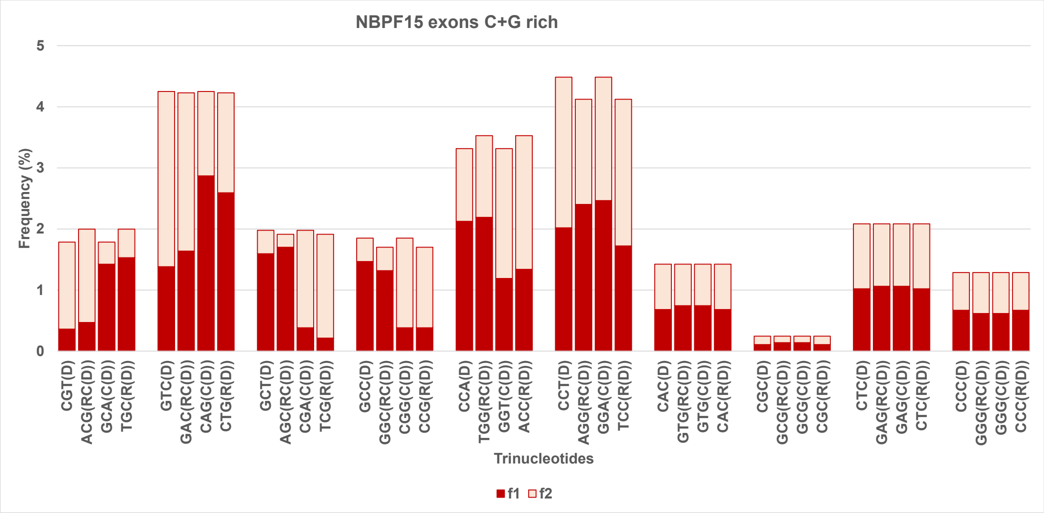

NBPF15 introns

C+G rich

D	f	%	RC	f	%	C	f	%	R	f	%	
**CGT**	100,00	0,33	ACG	61,00	0,20	GCA	415,00	1,35	TGC	429,00	1,40	IIa
**GTC**	388,00	1,26	GAC	283,00	0,92	CAG	591,00	1,92	CTG	729,00	2,37	
**GCT**	410,00	1,33	AGC	339,00	1,10	CGA	60,00	0,20	TCG	61,00	0,20	
**GCC**	309,00	1,01	GGC	378,00	1,23	CGG	51,00	0,17	CCG	77,00	0,25	IIb
**CCA**	520,00	1,69	TGG	616,00	2,01	GGT	351,00	1,14	ACC	358,00	1,17	
**CCT**	650,00	2,12	AGG	527,00	1,72	GGA	465,00	1,51	TCC	580,00	1,89	
**CAC**	439,00	1,43	GTG	609,00	1,98	GTG*			CAC*			IIc
**CGC**	47,00	0,15	GCG	59,00	0,19	GCG*			CGC*			
**CTC**	607,00	1,98	GAG	483,00	1,57	GAG*			CTC*			
**CCC**	417,00	1,36	GGG	426,00	1,39	GGG*			CCC*			

Quadruplet		f1	f2	fu
CGT	CGT(D)	0,33	1,35	1,68
	ACG(RC(D))	0,20	1,40	1,60
	GCA(C(D))	1,35	0,33	1,68
	TGC(R(D))	1,40	0,20	1,60
GTC	GTC(D)	1,26	1,92	3,19
	GAC(RC(D))	0,92	2,37	3,29
	CAG(C(D))	1,92	1,26	3,19
	CTG(R(D))	2,37	0,92	3,29
GCT	GCT(D)	1,33	0,20	1,53
	AGC(RC(D))	1,10	0,20	1,30
	CGA(C(D))	0,20	1,33	1,53
	TCG(R(D))	0,20	1,10	1,30
GCC	GCC(D)	1,01	0,17	1,17
	GGC(RC(D)	1,23	0,25	1,48
	CGG(C(D))	0,17	1,01	1,17
	CCG(R(D))	0,25	1,23	1,48
CCA	CCA(D)	1,69	1,14	2,84
	TGG(RC(D))	2,01	1,17	3,17
	GGT(C(D))	1,14	1,69	2,84
	ACC(R(D))	1,17	2,01	3,17
CCT	CCT(D)	2,12	1,51	3,63
	AGG(RC(D))	1,72	1,89	3,60
	GGA(C(D))	1,51	2,12	3,63
	TCC(R(D))	1,89	1,72	3,60
CAC	CAC(D)	0,71	0,99	1,71
	GTG(RC(D))	0,99	0,71	1,71
	GTG(C(D))	0,99	0,71	1,71
	CAC(R(D))	0,71	0,99	1,71
CGC	CGC(D)	0,08	0,10	0,17
	GCG(RC(D)	0,10	0,08	0,17
	GCG(C(D))	0,10	0,08	0,17
	CGC(R(D))	0,08	0,10	0,17
CTC	CTC(D)	0,99	0,79	1,77
	GAG(RC(D))	0,79	0,99	1,77
	GAG(C(D))	0,79	0,99	1,77
	CTC(R(D))	0,99	0,79	1,77
CCC	CCC(D)	0,68	0,69	1,37
	GGG(RC(D)	0,69	0,68	1,37
	GGG(C(D))	0,69	0,68	1,37
	CCC(R(D))	0,68	0,69	1,37

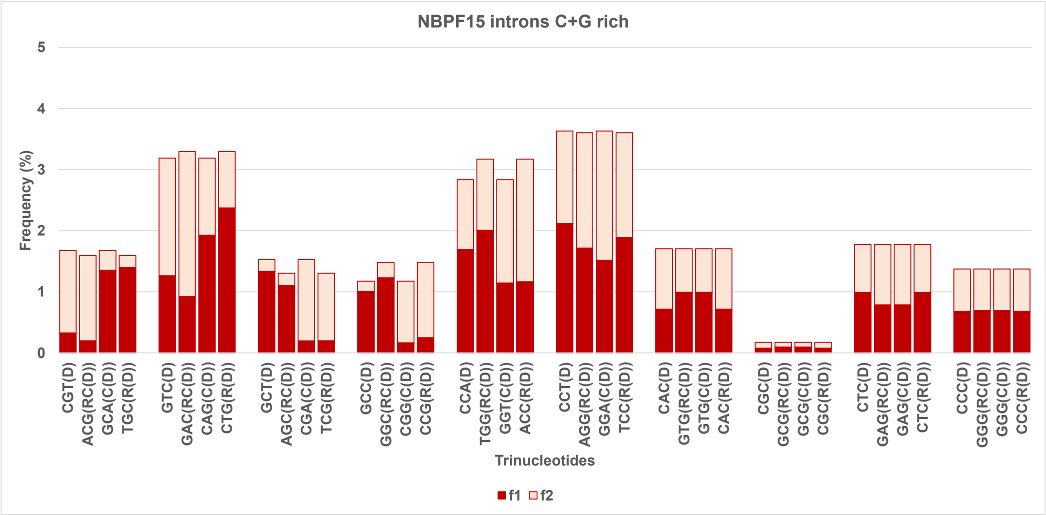

NBPF17P sequence

A-T rich												
**D**	**f**	**%**	**RC**	**f**	**%**	**C**	**f**	**%**	**R**	**f**	**%**	
**ATG**	816,00	1,96	CAT	844,00	2,03	TAC	459,00	1,10	GTA	440,00	1,06	Ia
**TGA**	926,00	2,22	TCA	908,00	2,18	ACT	670,00	1,61	AGT	740,00	1,78	
**TAG**	534,00	1,28	CTA	529,00	1,27	ATC	568,00	1,36	GAT	569,00	1,37	
**TAA**	705,00	1,69	TTA	734,00	1,76	ATT	984,00	2,36	AAT	950,00	2,28	Ib
**AAC**	558,00	1,34	GTT	678,00	1,63	TTG	849,00	2,04	CAA	707,00	1,70	
**AAG**	787,00	1,89	CTT	905,00	2,17	TTC	984,00	2,36	GAA	883,00	2,12	
**ACA**	790,00	1,90	TGT	996,00	2,39	TGT*			ACA*			Ic
**ATA**	794,00	1,91	TAT	799,00	1,92	TAT*			ATA*			
**AGA**	848,00	2,04	TCT	1128,00	2,71	TCT*			AGA*			
**AAA**	1295,00	3,11	TTT	1497,00	3,60	TTT*			AAA*			

Quadruplet		f1	f2	fu
ATG	ATG(D)	1,96	1,10	3,06
	CAT(RC(D))	2,03	1,06	3,08
	TAC(C(D))	1,10	1,96	3,06
	GTA(R(D))	1,06	2,03	3,08
TGA	TGA(D)	2,22	1,61	3,83
	TCA(RC(D))	2,18	1,78	3,96
	ACT(C(D))	1,61	2,22	3,83
	AGT(R(D))	1,78	2,18	3,96
TAG	TAG(D)	1,28	1,36	2,65
	CTA(RC(D))	1,27	1,37	2,64
	ATC(C(D))	1,36	1,28	2,65
	GAT(R(D))	1,37	1,27	2,64
TAA	TAA(D)	1,69	2,36	4,06
	TTA(RC(D))	1,76	2,28	4,05
	ATT(C(D))	2,36	1,69	4,06
	AAT(R(D))	2,28	1,76	4,05
AAC	AAC(D)	1,34	2,04	3,38
	GTT(RC(D))	1,63	1,70	3,33
	TTG(C(D))	2,04	1,34	3,38
	CAA(R(D))	1,70	1,63	3,33
AAG	AAG(D)	1,89	2,36	4,25
	CTT(RC(D))	2,17	2,12	4,30
	TTC(C(D))	2,36	1,89	4,25
	GAA(R(D))	2,12	2,17	4,30
ACA	ACA(D)	0,95	1,20	2,15
	TGT(RC(D))	1,20	0,95	2,15
	TGT(C(D))	1,20	0,95	2,15
	ACA(R(D))	0,95	1,20	2,15
ATA	ATA(D)	0,95	0,96	1,91
	TAT(RC(D))	0,96	0,95	1,91
	TAT(C(D))	0,96	0,95	1,91
	ATA(R(D))	0,95	0,95	1,91
AGA	AGA(D)	1,02	1,35	2,37
	TCT(RC(D))	1,35	1,02	2,37
	TCT(C(D))	1,35	1,02	2,37
	AGA(R(D))	1,02	1,35	2,37
AAA	AAA(D)	1,56	1,80	3,35
	TTT(RC(D))	1,80	1,56	3,35
	TTT(C(D))	1,80	1,56	3,35
	AAA(R(D))	1,56	1,80	3,35

Composition of DNA sequence in NBPF17P

Base	No.of bases	%
A	11631	27,94%
C	8652	20,79%
G	8607	20,68%
T	12736	30,60%
N	0	0,00%
Sum	41626	100,00%

% of coding bases from NBPF17P (including N bases)

92,03%

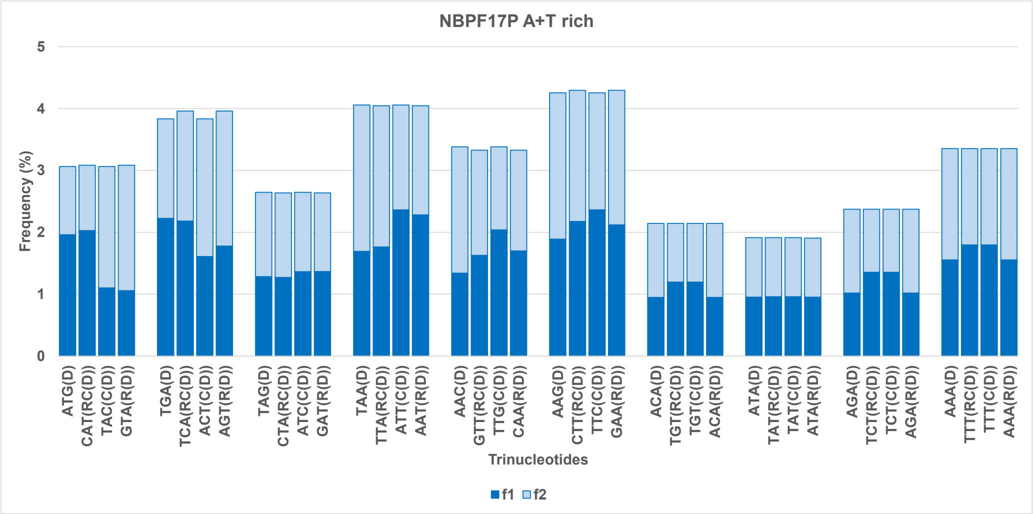

NBPF17P exons

A-T rich

D	f	%	RC	f	%	C	f	%	R	f	%	
**ATG**	41,00	1,94	CAT	36,00	1,70	TAC	18,00	0,85	GTA	11,00	0,52	Ia
**TGA**	62,00	2,93	TCA	53,00	2,50	ACT	46,00	2,17	AGT	45,00	2,13	
**TAG**	20,00	0,94	CTA	11,00	0,52	ATC	23,00	1,09	GAT	39,00	1,84	
**TAA**	11,00	0,52	TTA	20,00	0,94	ATT	26,00	1,23	AAT	22,00	1,04	Ib
**AAC**	30,00	1,42	GTT	21,00	0,99	TTG	41,00	1,94	CAA	37,00	1,75	
**AAG**	72,00	3,40	CTT	40,00	1,89	TTC	26,00	1,23	GAA	77,00	3,64	
**ACA**	36,00	1,70	TGT	24,00	1,13	TGT*			ACA*			Ic
**ATA**	19,00	0,90	TAT	12,00	0,57	TAT*			ATA*			
**AGA**	67,00	3,16	TCT	35,00	1,65	TCT*			AGA*			
**AAA**	43,00	2,03	TTT	26,00	1,23	TTT*			AAA*			

Quadruplet		f1	f2	fu
ATG	ATG(D)	1,94	0,85	2,79
	CAT(RC(D))	1,70	0,52	2,22
	TAC(C(D))	0,85	1,94	2,79
	GTA(R(D))	0,52	1,70	2,22
TGA	TGA(D)	2,93	2,17	5,10
	TCA(RC(D))	2,50	2,13	4,63
	ACT(C(D))	2,17	2,93	5,10
	AGT(R(D))	2,13	2,50	4,63
TAG	TAG(D)	0,94	1,09	2,03
	CTA(RC(D))	0,52	1,84	2,36
	ATC(C(D))	1,09	0,94	2,03
	GAT(R(D))	1,84	0,52	2,36
TAA	TAA(D)	0,52	1,23	1,75
	TTA(RC(D))	0,94	1,04	1,98
	ATT(C(D))	1,23	0,52	1,75
	AAT(R(D))	1,04	0,94	1,98
AAC	AAC(D)	1,42	1,94	3,35
	GTT(RC(D))	0,99	1,75	2,74
	TTG(C(D))	1,94	1,42	3,35
	CAA(R(D))	1,75	0,99	2,74
AAG	AAG(D)	3,40	1,23	4,63
	CTT(RC(D))	1,89	3,64	5,53
	TTC(C(D))	1,23	3,40	4,63
	GAA(R(D))	3,64	1,89	5,53
ACA	ACA(D)	0,85	0,57	1,42
	TGT(RC(D))	0,57	0,85	1,42
	TGT(C(D))	0,57	0,85	1,42
	ACA(R(D))	0,85	0,57	1,42
ATA	ATA(D)	0,45	0,28	0,73
	TAT(RC(D))	0,28	0,45	0,73
	TAT(C(D))	0,28	0,45	0,73
	ATA(R(D))	0,45	0,45	0,90
AGA	AGA(D)	1,58	0,83	2,41
	TCT(RC(D))	0,83	1,58	2,41
	TCT(C(D))	0,83	1,58	2,41
	AGA(R(D))	1,58	0,83	2,41
AAA	AAA(D)	1,02	0,61	1,63
	TTT(RC(D))	0,61	1,02	1,63
	TTT(C(D))	0,61	1,02	1,63
	AAA(R(D))	1,02	0,61	1,63

Composition of DNA sequence in NBPF17P exons

Base	No.of bases	%
A	612	28,88%
C	498	23,50%
G	538	25,39%
T	471	22,23%
N	0	0,00%
Sum	2119	100,00%

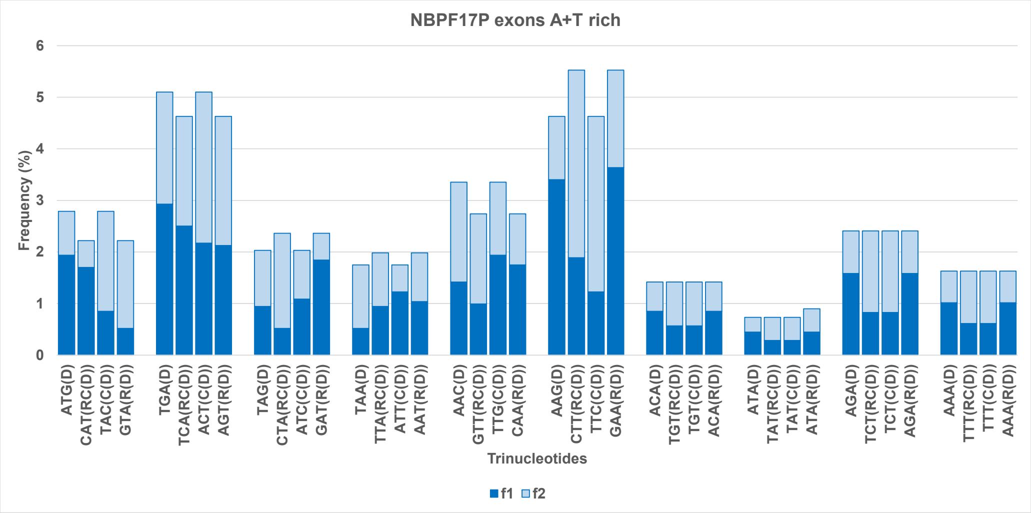

NBPF17P introns

A-T rich

D	f	%	RC	f	%	C	f	%	R	f	%	
**ATG**	757,00	1,98	CAT	784,00	2,05	TAC	431,00	1,13	GTA	419,00	1,09	Ia
**TGA**	839,00	2,19	TCA	826,00	2,16	ACT	609,00	1,59	AGT	668,00	1,74	
**TAG**	493,00	1,29	CTA	498,00	1,30	ATC	528,00	1,38	GAT	512,00	1,34	
**TAA**	683,00	1,78	TTA	699,00	1,82	ATT	936,00	2,44	AAT	916,00	2,39	Ib
**AAC**	510,00	1,33	GTT	637,00	1,66	TTG	787,00	2,05	CAA	648,00	1,69	
**AAG**	697,00	1,82	CTT	842,00	2,20	TTC	929,00	2,43	GAA	792,00	2,07	
**ACA**	735,00	1,92	TGT	947,00	2,47	TGT*			ACA*			Ic
**ATA**	762,00	1,99	TAT	771,00	2,01	TAT*			ATA*			
**AGA**	766,00	2,00	TCT	1058,00	2,76	TCT*			AGA*			
**AAA**	1240,00	3,24	TTT	1421,00	3,71	TTT*			AAA*			

Quadruplet		f1	f2	fu
ATG	ATG(D)	1,98	1,13	3,10
	CAT(RC(D))	2,05	1,09	3,14
	TAC(C(D))	1,13	1,98	3,10
	GTA(R(D))	1,09	2,05	3,14
TGA	TGA(D)	2,19	1,59	3,78
	TCA(RC(D))	2,16	1,74	3,90
	ACT(C(D))	1,59	2,19	3,78
	AGT(R(D))	1,74	2,16	3,90
TAG	TAG(D)	1,29	1,38	2,67
	CTA(RC(D))	1,30	1,34	2,64
	ATC(C(D))	1,38	1,29	2,67
	GAT(R(D))	1,34	1,30	2,64
TAA	TAA(D)	1,78	2,44	4,23
	TTA(RC(D))	1,82	2,39	4,22
	ATT(C(D))	2,44	1,78	4,23
	AAT(R(D))	2,39	1,82	4,22
AAC	AAC(D)	1,33	2,05	3,39
	GTT(RC(D))	1,66	1,69	3,35
	TTG(C(D))	2,05	1,33	3,39
	CAA(R(D))	1,69	1,66	3,35
AAG	AAG(D)	1,82	2,43	4,24
	CTT(RC(D))	2,20	2,07	4,27
	TTC(C(D))	2,43	1,82	4,24
	GAA(R(D))	2,07	2,20	4,27
ACA	ACA(D)	0,96	1,24	2,20
	TGT(RC(D))	1,24	0,96	2,20
	TGT(C(D))	1,24	0,96	2,20
	ACA(R(D))	0,96	1,24	2,20
ATA	ATA(D)	0,99	1,01	2,00
	TAT(RC(D))	1,01	0,99	2,00
	TAT(C(D))	1,01	0,99	2,00
	ATA(R(D))	0,99	0,99	1,99
AGA	AGA(D)	1,00	1,38	2,38
	TCT(RC(D))	1,38	1,00	2,38
	TCT(C(D))	1,38	1,00	2,38
	AGA(R(D))	1,00	1,38	2,38
AAA	AAA(D)	1,62	1,85	3,47
	TTT(RC(D))	1,85	1,62	3,47
	TTT(C(D))	1,85	1,62	3,47
	AAA(R(D))	1,62	1,85	3,47

Composition of DNA sequence NBPF17P introns

Base	No.of bases	%
A	10743	28,04%
C	7844	20,48%
G	7817	20,41%
T	11903	31,07%
N	0	0,00%
Sum	38307	100,00%

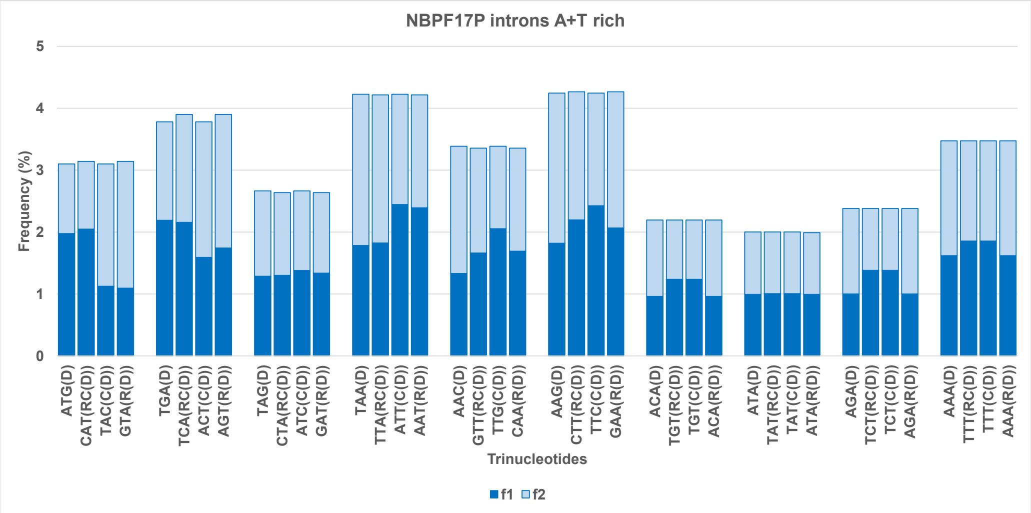

						C+G rich						
**D**	**f**	**%**	**RC**	**f**	**%**	**C**	**f**	**%**	**R**	**f**	**%**	
**CGT**	75,00	0,18	ACG	77,00	0,18	GCA	587,00	1,41	TGC	579,00	1,39	IIa
**GTC**	479,00	1,15	GAC	417,00	1,00	CAG	871,00	2,09	CTG	971,00	2,33	
**GCT**	568,00	1,36	AGC	520,00	1,25	CGA	75,00	0,18	TCG	81,00	0,19	
**GCC**	424,00	1,02	GGC	474,00	1,14	CGG	61,00	0,15	CCG	59,00	0,14	IIb
**CCA**	724,00	1,74	TGG	823,00	1,98	GGT	473,00	1,14	ACC	484,00	1,16	
**CCT**	859,00	2,06	AGG	749,00	1,80	GGA	686,00	1,65	TCC	734,00	1,76	
**CAC**	587,00	1,41	GTG	688,00	1,65	GTG*			CAC*			IIc
**CGC**	52,00	0,12	GCG	46,00	0,11	GCG*			CGC*			
**CTC**	820,00	1,97	GAG	665,00	1,60	GAG*			CTC*			
**CCC**	513,00	1,23	GGG	529,00	1,27	GGG*			CCC*			

Quadruplet		f1	f2	fu
CGT	CGT(D)	0,18	1,41	1,59
	ACG(RC(D))	0,18	1,39	1,58
	GCA(C(D))	1,41	0,18	1,59
	TGC(R(D))	1,39	0,18	1,58
GTC	GTC(D)	1,15	2,09	3,24
	GAC(RC(D))	1,00	2,33	3,33
	CAG(C(D))	2,09	1,15	3,24
	CTG(R(D))	2,33	1,00	3,33
GCT	GCT(D)	1,36	0,18	1,54
	AGC(RC(D))	1,25	0,19	1,44
	CGA(C(D))	0,18	1,36	1,54
	TCG(R(D))	0,19	1,25	1,44
GCC	GCC(D)	1,02	0,15	1,17
	GGC(RC(D))	1,14	0,14	1,28
	CGG(C(D))	0,15	1,02	1,17
	CCG(R(D))	0,14	1,14	1,28
CCA	CCA(D)	1,74	1,14	2,88
	TGG(RC(D))	1,98	1,16	3,14
	GGT(C(D))	1,14	1,74	2,88
	ACC(R(D))	1,16	1,98	3,14
CCT	CCT(D)	2,06	1,65	3,71
	AGG(RC(D))	1,80	1,76	3,56
	GGA(C(D))	1,65	2,06	3,71
	TCC(R(D))	1,76	1,80	3,56
CAC	CAC(D)	0,71	0,83	1,53
	GTG(RC(D))	0,83	0,71	1,53
	GTG(C(D))	0,83	0,71	1,53
	CAC(R(D))	0,71	0,83	1,53
CGC	CGC(D)	0,06	0,06	0,12
	GCG(RC(D))	0,06	0,06	0,12
	GCG(C(D))	0,06	0,06	0,12
	CGC(R(D))	0,06	0,06	0,12
CTC	CTC(D)	0,99	0,80	1,78
	GAG(RC(D))	0,80	0,99	1,78
	GAG(C(D))	0,80	0,99	1,78
	CTC(R(D))	0,99	0,80	1,78
CCC	CCC(D)	0,62	0,64	1,25
	GGG(RC(D))	0,64	0,62	1,25
	GGG(C(D))	0,64	0,62	1,25
	CCC(R(D))	0,62	0,64	1,25

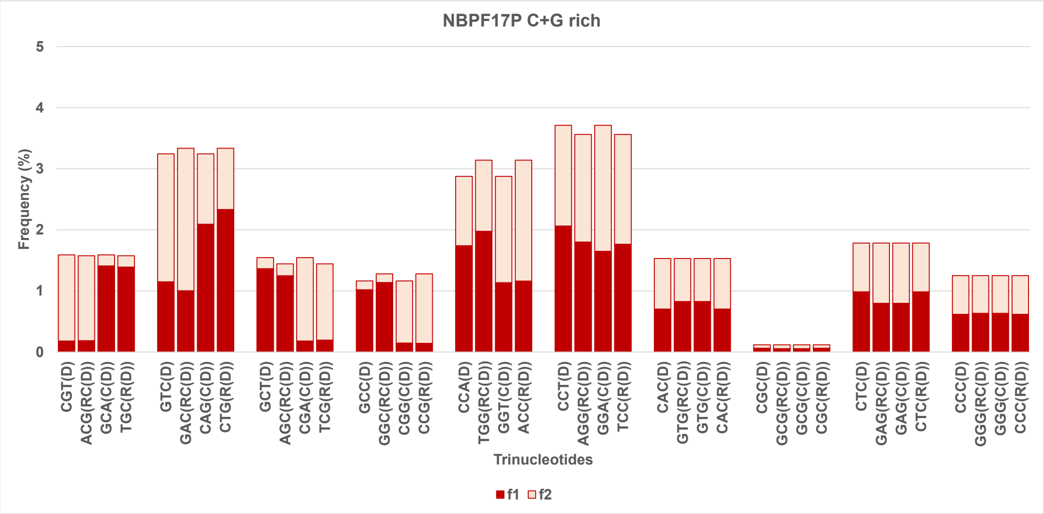

NBPF17P exons

C+G rich

D	f	%	RC	f	%	C	f	%	R	f	%		
**CGT**	5,00	0,24	ACG	5,00	0,24	GCA	37,00	1,75	TGC	36,00	1,70	IIa	86356
**GTC**	24,00	1,13	GAC	30,00	1,42	CAG	74,00	3,50	CTG	50,00	2,36		80741
**GCT**	38,00	1,79	AGC	46,00	2,17	CGA	7,00	0,33	TCG	6,00	0,28		44919
**GCC**	38,00	1,79	GGC	32,00	1,51	CGG	5,00	0,24	CCG	5,00	0,24	IIb	22459
**CCA**	54,00	2,55	TGG	41,00	1,94	GGT	12,00	0,57	ACC	24,00	1,13		45985
**CCT**	43,00	2,03	AGG	65,00	3,07	GGA	67,00	3,16	TCC	40,00	1,89		57215
**CAC**	33,00	1,56	GTG	31,00	1,46	GTG*			CAC*			IIc	
**CGC**	2,00	0,09	GCG	3,00	0,14	GCG*			CGC*				
**CTC**	61,00	2,88	GAG	57,00	2,69	GAG*			CTC*				
**CCC**	35,00	1,65	GGG	21,00	0,99	GGG*			CCC*				

Quadruplet		f1	f2	fu
CGT	CGT(D)	0,24	1,75	1,98
	ACG(RC(D))	0,24	1,70	1,94
	GCA(C(D))	1,75	0,24	1,98
	TGC(R(D))	1,70	0,24	1,94
GTC	GTC(D)	1,13	3,50	4,63
	GAC(RC(D))	1,42	2,36	3,78
	CAG(C(D))	3,50	1,13	4,63
	CTG(R(D))	2,36	1,42	3,78
GCT	GCT(D)	1,79	0,33	2,13
	AGC(RC(D))	2,17	0,28	2,46
	CGA(C(D))	0,33	1,79	2,13
	TCG(R(D))	0,28	2,17	2,46
GCC	GCC(D)	1,79	0,24	2,03
	GGC(RC(D)	1,51	0,24	1,75
	CGG(C(D))	0,24	1,79	2,03
	CCG(R(D))	0,24	1,51	1,75
CCA	CCA(D)	2,55	0,57	3,12
	TGG(RC(D))	1,94	1,13	3,07
	GGT(C(D))	0,57	2,55	3,12
	ACC(R(D))	1,13	1,94	3,07
CCT	CCT(D)	2,03	3,16	5,20
	AGG(RC(D))	3,07	1,89	4,96
	GGA(C(D))	3,16	2,03	5,20
	TCC(R(D))	1,89	3,07	4,96
CAC	CAC(D)	0,78	0,73	1,51
	GTG(RC(D))	0,73	0,78	1,51
	GTG(C(D))	0,73	0,78	1,51
	CAC(R(D))	0,78	0,73	1,51
CGC	CGC(D)	0,05	0,07	0,12
	GCG(RC(D)	0,07	0,05	0,12
	GCG(C(D))	0,07	0,05	0,12
	CGC(R(D))	0,05	0,07	0,12
CTC	CTC(D)	1,44	1,35	2,79
	GAG(RC(D))	1,35	1,44	2,79
	GAG(C(D))	1,35	1,44	2,79
	CTC(R(D))	1,44	1,35	2,79
CCC	CCC(D)	0,83	0,50	1,32
	GGG(RC(D)	0,50	0,83	1,32
	GGG(C(D))	0,50	0,83	1,32
	CCC(R(D))	0,83	0,50	1,32

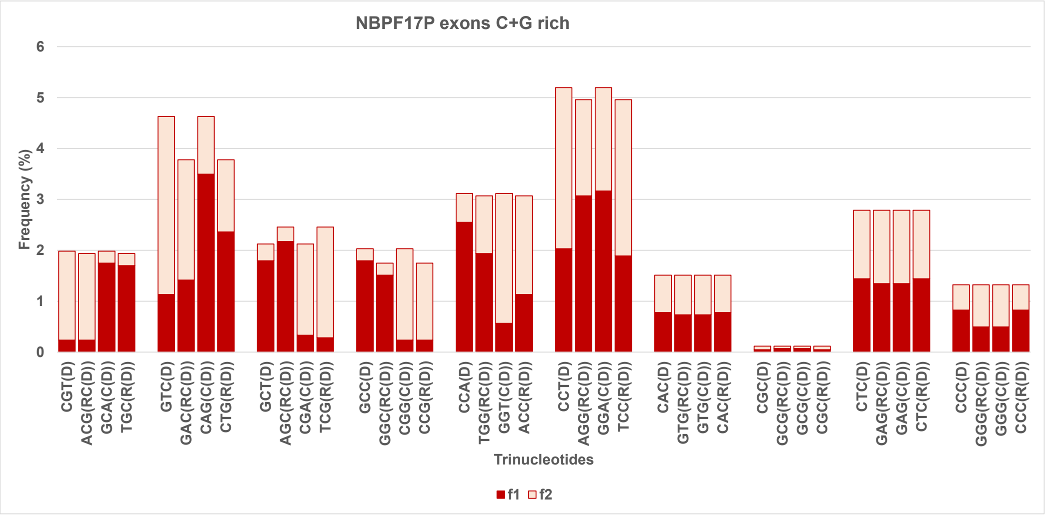

NBPF17P introns

C+G rich												
**D**	**f**	**%**	**RC**	**f**	**%**	**C**	**f**	**%**	**R**	**f**	**%**	
**CGT**	66,00	0,17	ACG	70,00	0,18	GCA	529,00	1,38	TGC	522,00	1,36	IIa
**GTC**	437,00	1,14	GAC	370,00	0,97	CAG	763,00	1,99	CTG	889,00	2,32	
**GCT**	508,00	1,33	AGC	460,00	1,20	CGA	66,00	0,17	TCG	71,00	0,19	
**GCC**	372,00	0,97	GGC	423,00	1,10	CGG	54,00	0,14	CCG	49,00	0,13	IIb
**CCA**	639,00	1,67	TGG	759,00	1,98	GGT	445,00	1,16	ACC	431,00	1,13	
**CCT**	782,00	2,04	AGG	657,00	1,72	GGA	602,00	1,57	TCC	667,00	1,74	
**CAC**	534,00	1,39	GTG	634,00	1,66	GTG*			CAC*			IIc
**CGC**	47,00	0,12	GCG	43,00	0,11	GCG*			CGC*			
**CTC**	728,00	1,90	GAG	599,00	1,56	GAG*			CTC*			
**CCC**	455,00	1,19	GGG	494,00	1,29	GGG*			CCC*			

Quadruplet		f1	f2	fu
CGT	CGT(D)	0,17	1,38	1,55
	ACG(RC(D))	0,18	1,36	1,55
	GCA(C(D))	1,38	0,17	1,55
	TGC(R(D))	1,36	0,18	1,55
GTC	GTC(D)	1,14	1,99	3,13
	GAC(RC(D))	0,97	2,32	3,29
	CAG(C(D))	1,99	1,14	3,13
	CTG(R(D))	2,32	0,97	3,29
GCT	GCT(D)	1,33	0,17	1,50
	AGC(RC(D))	1,20	0,19	1,39
	CGA(C(D))	0,17	1,33	1,50
	TCG(R(D))	0,19	1,20	1,39
GCC	GCC(D)	0,97	0,14	1,11
	GGC(RC(D))	1,10	0,13	1,23
	CGG(C(D))	0,14	0,97	1,11
	CCG(R(D))	0,13	1,10	1,23
CCA	CCA(D)	1,67	1,16	2,83
	TGG(RC(D))	1,98	1,13	3,11
	GGT(C(D))	1,16	1,67	2,83
	ACC(R(D))	1,13	1,98	3,11
CCT	CCT(D)	2,04	1,57	3,61
	AGG(RC(D))	1,72	1,74	3,46
	GGA(C(D))	1,57	2,04	3,61
	TCC(R(D))	1,74	1,72	3,46
CAC	CAC(D)	0,70	0,83	1,52
	GTG(RC(D))	0,83	0,70	1,52
	GTG(C(D))	0,83	0,70	1,52
	CAC(R(D))	0,70	0,83	1,52
CGC	CGC(D)	0,06	0,06	0,12
	GCG(RC(D))	0,06	0,06	0,12
	GCG(C(D))	0,06	0,06	0,12
	CGC(R(D))	0,06	0,06	0,12
CTC	CTC(D)	0,95	0,78	1,73
	GAG(RC(D))	0,78	0,95	1,73
	GAG(C(D))	0,78	0,95	1,73
	CTC(R(D))	0,95	0,78	1,73
CCC	CCC(D)	0,59	0,64	1,24
	GGG(RC(D))	0,64	0,59	1,24
	GGG(C(D))	0,64	0,59	1,24
	CCC(R(D))	0,59	0,64	1,24

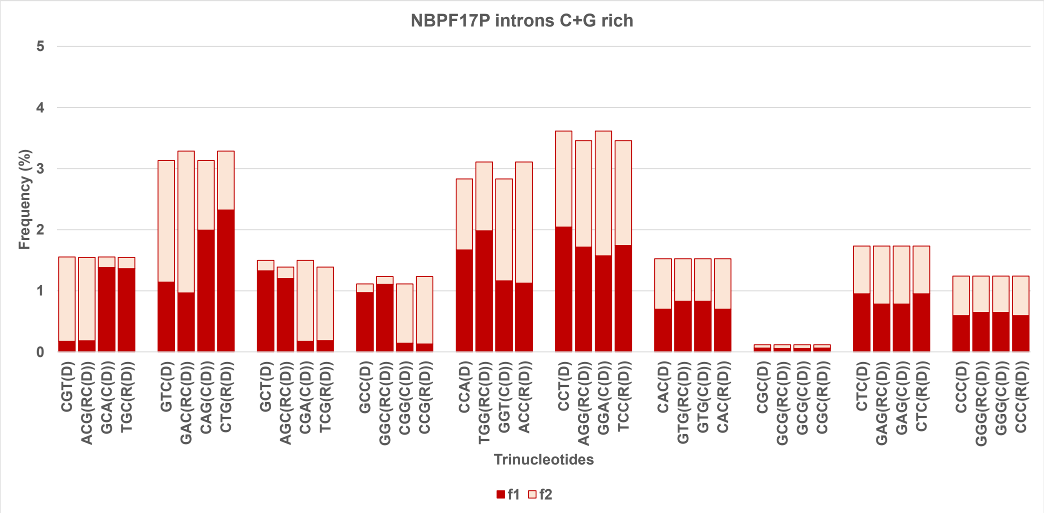

NBPF19 sequence

A-T rich

D	f	%	RC	f	%	C	f	%	R	f	%	
**ATG**	3299,00	1,98	CAT	3209,00	1,92	TAC	1884,00	1,13	GTA	1816,00	1,09	Ia
**TGA**	3873,00	2,32	TCA	3442,00	2,06	ACT	2956,00	1,77	AGT	2818,00	1,69	
**TAG**	2281,00	1,37	CTA	2192,00	1,31	ATC	1974,00	1,18	GAT	2536,00	1,52	
**TAA**	2668,00	1,60	TTA	3365,00	2,02	ATT	4316,00	2,59	AAT	3304,00	1,98	Ib
**AAC**	2190,00	1,31	GTT	2851,00	1,71	TTG	3659,00	2,19	CAA	2374,00	1,42	
**AAG**	2936,00	1,76	CTT	3846,00	2,30	TTC	3989,00	2,39	GAA	3388,00	2,03	
**ACA**	2634,00	1,58	TGT	4640,00	2,78	TGT*			ACA*			Ic
**ATA**	2666,00	1,60	TAT	3206,00	1,92	TAT*			ATA*			
**AGA**	3246,00	1,94	TCT	4743,00	2,84	TCT*			AGA*			
**AAA**	4354,00	2,61	TTT	7078,00	4,24	TTT*			AAA*			

Quadruplet		f1	f2	fu
ATG	ATG(D)	1,98	1,13	3,10
	CAT(RC(D))	1,92	1,09	3,01
	TAC(C(D))	1,13	1,98	3,10
	GTA(R(D))	1,09	1,92	3,01
TGA	TGA(D)	2,32	1,77	4,09
	TCA(RC(D))	2,06	1,69	3,75
	ACT(C(D))	1,77	2,32	4,09
	AGT(R(D))	1,69	2,06	3,75
TAG	TAG(D)	1,37	1,18	2,55
	CTA(RC(D))	1,31	1,52	2,83
	ATC(C(D))	1,18	1,37	2,55
	GAT(R(D))	1,52	1,31	2,83
TAA	TAA(D)	1,60	2,59	4,18
	TTA(RC(D))	2,02	1,98	3,99
	ATT(C(D))	2,59	1,60	4,18
	AAT(R(D))	1,98	2,02	3,99
AAC	AAC(D)	1,31	2,19	3,50
	GTT(RC(D))	1,71	1,42	3,13
	TTG(C(D))	2,19	1,31	3,50
	CAA(R(D))	1,42	1,71	3,13
AAG	AAG(D)	1,76	2,39	4,15
	CTT(RC(D))	2,30	2,03	4,33
	TTC(C(D))	2,39	1,76	4,15
	GAA(R(D))	2,03	2,30	4,33
ACA	ACA(D)	0,79	1,39	2,18
	TGT(RC(D))	1,39	0,79	2,18
	TGT(C(D))	1,39	0,79	2,18
	ACA(R(D))	0,79	1,39	2,18
ATA	ATA(D)	0,80	0,96	1,76
	TAT(RC(D))	0,96	0,80	1,76
	TAT(C(D))	0,96	0,80	1,76
	ATA(R(D))	0,80	0,80	1,60
AGA	AGA(D)	0,97	1,42	2,39
	TCT(RC(D))	1,42	0,97	2,39
	TCT(C(D))	1,42	0,97	2,39
	AGA(R(D))	0,97	1,42	2,39
AAA	AAA(D)	1,30	2,12	3,42
	TTT(RC(D))	2,12	1,30	3,42
	TTT(C(D))	2,12	1,30	3,42
	AAA(R(D))	1,30	2,12	3,42

Composition of DNA sequence in NBPF19

Base	No.of bases	%
A	44074	26,40%
C	33679	20,17%
G	35761	21,42%
T	53425	32,00%
N	0	0,00%
Sum	166939	100,00%

% of coding bases from NBPF19 (including N bases)

90,64%

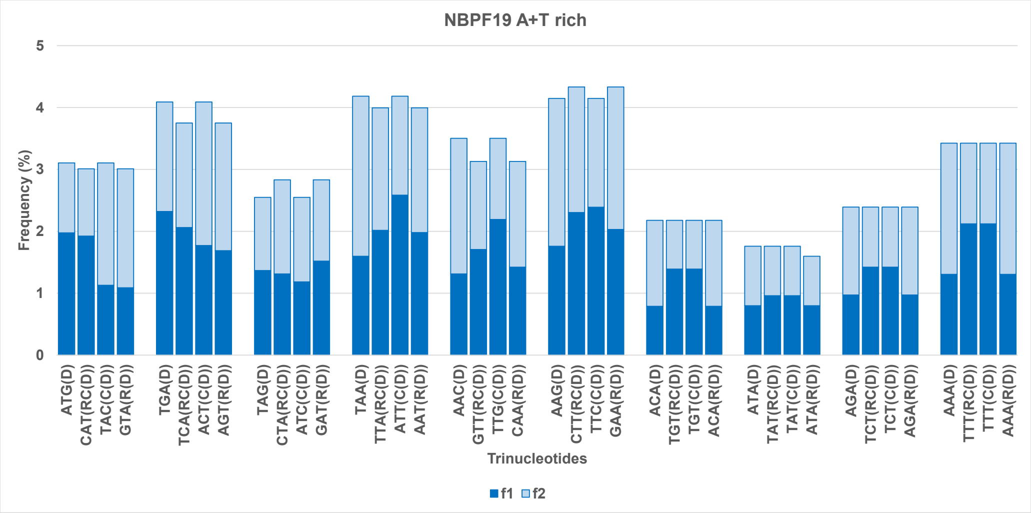

NBPF19 exons

A-T rich

D	f	%	RC	f	%	C	f	%	R	f	%	
**ATG**	279,00	1,93	CAT	220,00	1,53	TAC	127,00	0,88	GTA	87,00	0,60	Ia
**TGA**	344,00	2,39	TCA	308,00	2,14	ACT	276,00	1,91	AGT	243,00	1,68	
**TAG**	117,00	0,81	CTA	75,00	0,52	ATC	104,00	0,72	GAT	207,00	1,44	
**TAA**	50,00	0,35	TTA	187,00	1,30	ATT	206,00	1,43	AAT	107,00	0,74	Ib
**AAC**	182,00	1,26	GTT	213,00	1,48	TTG	361,00	2,50	CAA	197,00	1,37	
**AAG**	597,00	4,14	CTT	314,00	2,18	TTC	185,00	1,28	GAA	638,00	4,42	
**ACA**	208,00	1,44	TGT	235,00	1,63	TGT*			ACA*			Ic
**ATA**	94,00	0,65	TAT	149,00	1,03	TAT*			ATA*			
**AGA**	517,00	3,58	TCT	199,00	1,38	TCT*			AGA*			
**AAA**	392,00	2,72	TTT	207,00	1,44	TTT*			AAA*			

Quadruplet		f1	f2	fu
ATG	ATG(D)	1,93	0,88	2,81
	CAT(RC(D))	1,53	0,60	2,13
	TAC(C(D))	0,88	1,93	2,81
	GTA(R(D))	0,60	1,53	2,13
TGA	TGA(D)	2,39	1,91	4,30
	TCA(RC(D))	2,14	1,68	3,82
	ACT(C(D))	1,91	2,39	4,30
	AGT(R(D))	1,68	2,14	3,82
TAG	TAG(D)	0,81	0,72	1,53
	CTA(RC(D))	0,52	1,44	1,96
	ATC(C(D))	0,72	0,81	1,53
	GAT(R(D))	1,44	0,52	1,96
TAA	TAA(D)	0,35	1,43	1,77
	TTA(RC(D))	1,30	0,74	2,04
	ATT(C(D))	1,43	0,35	1,77
	AAT(R(D))	0,74	1,30	2,04
AAC	AAC(D)	1,26	2,50	3,76
	GTT(RC(D))	1,48	1,37	2,84
	TTG(C(D))	2,50	1,26	3,76
	CAA(R(D))	1,37	1,48	2,84
AAG	AAG(D)	4,14	1,28	5,42
	CTT(RC(D))	2,18	4,42	6,60
	TTC(C(D))	1,28	4,14	5,42
	GAA(R(D))	4,42	2,18	6,60
ACA	ACA(D)	0,72	0,81	1,54
	TGT(RC(D))	0,81	0,72	1,54
	TGT(C(D))	0,81	0,72	1,54
	ACA(R(D))	0,72	0,81	1,54
ATA	ATA(D)	0,33	0,52	0,84
	TAT(RC(D))	0,52	0,33	0,84
	TAT(C(D))	0,52	0,33	0,84
	ATA(R(D))	0,33	0,33	0,65
AGA	AGA(D)	1,79	0,69	2,48
	TCT(RC(D))	0,69	1,79	2,48
	TCT(C(D))	0,69	1,79	2,48
	AGA(R(D))	1,79	0,69	2,48
AAA	AAA(D)	1,36	0,72	2,08
	TTT(RC(D))	0,72	1,36	2,08
	TTT(C(D))	0,72	1,36	2,08
	AAA(R(D))	1,36	0,72	2,08

Composition of DNA sequence in NBPF19 exons

Base	No.of bases	%
A	4149	28,76%
C	3076	21,32%
G	3911	27,11%
T	3289	22,80%
N	0	0,00%
Sum	14425	100,00%

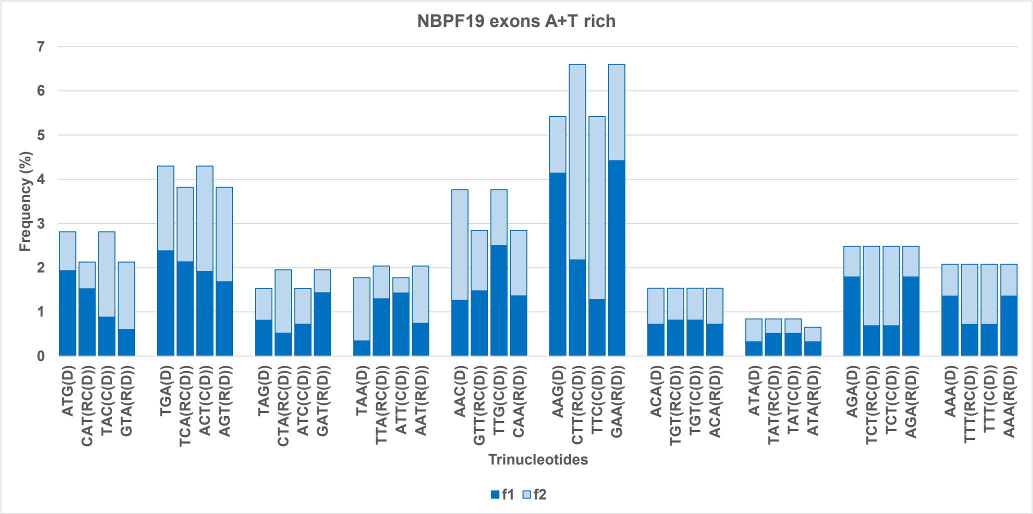

NBPF19 introns

A-T rich

D	f	%	RC	f	%	C	f	%	R	f	%	
**ATG**	3000,00	1,98	CAT	2974,00	1,97	TAC	1743,00	1,15	GTA	1716,00	1,13	Ia
**TGA**	3525,00	2,33	TCA	3119,00	2,06	ACT	2662,00	1,76	AGT	2541,00	1,68	
**TAG**	2151,00	1,42	CTA	2101,00	1,39	ATC	1860,00	1,23	GAT	2315,00	1,53	
**TAA**	2603,00	1,72	TTA	3162,00	2,09	ATT	4091,00	2,70	AAT	3180,00	2,10	Ib
**AAC**	1999,00	1,32	GTT	2621,00	1,73	TTG	3275,00	2,16	CAA	2164,00	1,43	
**AAG**	2321,00	1,53	CTT	3501,00	2,31	TTC	3776,00	2,50	GAA	2733,00	1,81	
**ACA**	2406,00	1,59	TGT	4368,00	2,89	TGT*			ACA*			Ic
**ATA**	2558,00	1,69	TAT	3040,00	2,01	TAT*			ATA*			
**AGA**	2715,00	1,79	TCT	4523,00	2,99	TCT*			AGA*			
**AAA**	3948,00	2,61	TTT	6824,00	4,51	TTT*			AAA*			

Quadruplet		f1	f2	fu
ATG	ATG(D)	1,98	1,15	3,13
	CAT(RC(D))	1,97	1,13	3,10
	TAC(C(D))	1,15	1,98	3,13
	GTA(R(D))	1,13	1,97	3,10
TGA	TGA(D)	2,33	1,76	4,09
	TCA(RC(D))	2,06	1,68	3,74
	ACT(C(D))	1,76	2,33	4,09
	AGT(R(D))	1,68	2,06	3,74
TAG	TAG(D)	1,42	1,23	2,65
	CTA(RC(D))	1,39	1,53	2,92
	ATC(C(D))	1,23	1,42	2,65
	GAT(R(D))	1,53	1,39	2,92
TAA	TAA(D)	1,72	2,70	4,42
	TTA(RC(D))	2,09	2,10	4,19
	ATT(C(D))	2,70	1,72	4,42
	AAT(R(D))	2,10	2,09	4,19
AAC	AAC(D)	1,32	2,16	3,49
	GTT(RC(D))	1,73	1,43	3,16
	TTG(C(D))	2,16	1,32	3,49
	CAA(R(D))	1,43	1,73	3,16
AAG	AAG(D)	1,53	2,50	4,03
	CTT(RC(D))	2,31	1,81	4,12
	TTC(C(D))	2,50	1,53	4,03
	GAA(R(D))	1,81	2,31	4,12
ACA	ACA(D)	0,80	1,44	2,24
	TGT(RC(D))	1,44	0,80	2,24
	TGT(C(D))	1,44	0,80	2,24
	ACA(R(D))	0,80	1,44	2,24
ATA	ATA(D)	0,85	1,00	1,85
	TAT(RC(D))	1,00	0,85	1,85
	TAT(C(D))	1,00	0,85	1,85
	ATA(R(D))	0,85	0,85	1,69
AGA	AGA(D)	0,90	1,49	2,39
	TCT(RC(D))	1,49	0,90	2,39
	TCT(C(D))	1,49	0,90	2,39
	AGA(R(D))	0,90	1,49	2,39
AAA	AAA(D)	1,30	2,25	3,56
	TTT(RC(D))	2,25	1,30	3,56
	TTT(C(D))	2,25	1,30	3,56
	AAA(R(D))	1,30	2,25	3,56

Composition of DNA sequence NBPF19 introns

Base	No.of bases	%
A	39669	26,22%
C	30309	20,03%
G	31541	20,84%
T	49795	32,91%
N	0	0,00%
Sum	151314	100,00%

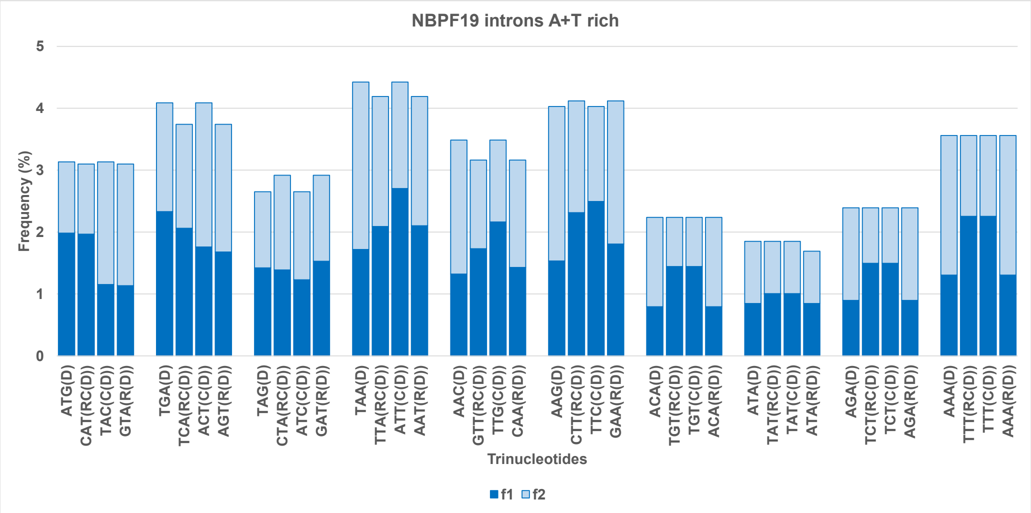

NBPF19 sequence

						C+G rich						
**D**	**f**	**%**	**RC**	**f**	**%**	**C**	**f**	**%**	**R**	**f**	**%**	
**CGT**	336,00	0,20	ACG	343,00	0,21	GCA	2227,00	1,33	TGC	2171,00	1,30	IIa
**GTC**	1918,00	1,15	GAC	1732,00	1,04	CAG	3278,00	1,96	CTG	4026,00	2,41	
**GCT**	2256,00	1,35	AGC	2079,00	1,25	CGA	379,00	0,23	TCG	326,00	0,20	
**GCC**	1754,00	1,05	GGC	1985,00	1,19	CGG	283,00	0,17	CCG	331,00	0,20	IIb
**CCA**	2564,00	1,54	TGG	3439,00	2,06	GGT	1930,00	1,16	ACC	1879,00	1,13	
**CCT**	3399,00	2,04	AGG	3079,00	1,84	GGA	2886,00	1,73	TCC	2661,00	1,59	
**CAC**	2006,00	1,20	GTG	3139,00	1,88	GTG*			CAC*			IIc
**CGC**	322,00	0,19	GCG	320,00	0,19	GCG*			CGC*			
**CTC**	3291,00	1,97	GAG	2727,00	1,63	GAG*			CTC*			
**CCC**	1843,00	1,10	GGG	2295,00	1,37	GGG*			CCC*			

Quadruplet		f1	f2	fu
CGT	CGT(D)	0,20	1,33	1,54
	ACG(RC(D))	0,21	1,30	1,51
	GCA(C(D))	1,33	0,20	1,54
	TGC(R(D))	1,30	0,21	1,51
GTC	GTC(D)	1,15	1,96	3,11
	GAC(RC(D))	1,04	2,41	3,45
	CAG(C(D))	1,96	1,15	3,11
	CTG(R(D))	2,41	1,04	3,45
GCT	GCT(D)	1,35	0,23	1,58
	AGC(RC(D))	1,25	0,20	1,44
	CGA(C(D))	0,23	1,35	1,58
	TCG(R(D))	0,20	1,25	1,44
GCC	GCC(D)	1,05	0,17	1,22
	GGC(RC(D))	1,19	0,20	1,39
	CGG(C(D))	0,17	1,05	1,22
	CCG(R(D))	0,20	1,19	1,39
CCA	CCA(D)	1,54	1,16	2,69
	TGG(RC(D))	2,06	1,13	3,19
	GGT(C(D))	1,16	1,54	2,69
	ACC(R(D))	1,13	2,06	3,19
CCT	CCT(D)	2,04	1,73	3,76
	AGG(RC(D))	1,84	1,59	3,44
	GGA(C(D))	1,73	2,04	3,76
	TCC(R(D))	1,59	1,84	3,44
CAC	CAC(D)	0,60	0,94	1,54
	GTG(RC(D))	0,94	0,60	1,54
	GTG(C(D))	0,94	0,60	1,54
	CAC(R(D))	0,60	0,94	1,54
CGC	CGC(D)	0,10	0,10	0,19
	GCG(RC(D))	0,10	0,10	0,19
	GCG(C(D))	0,10	0,10	0,19
	CGC(R(D))	0,10	0,10	0,19
CTC	CTC(D)	0,99	0,82	1,80
	GAG(RC(D))	0,82	0,99	1,80
	GAG(C(D))	0,82	0,99	1,80
	CTC(R(D))	0,99	0,82	1,80
CCC	CCC(D)	0,55	0,69	1,24
	GGG(RC(D))	0,69	0,55	1,24
	GGG(C(D))	0,69	0,55	1,24
	CCC(R(D))	0,55	0,69	1,24

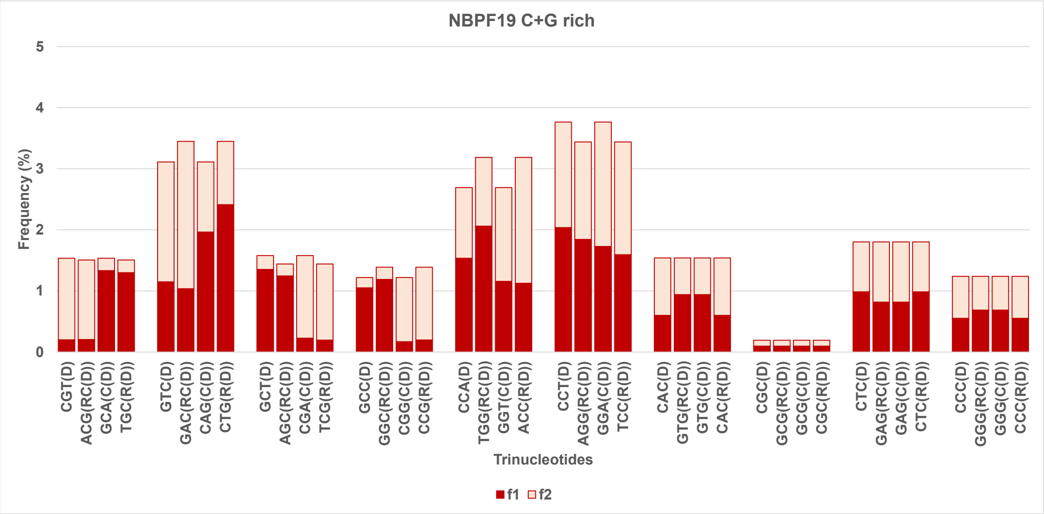

NBPF19 exons

C+G rich

D	f	%	RC	f	%	C	f	%	R	f	%		
**CGT**	42,00	0,29	ACG	46,00	0,32	GCA	192,00	1,33	TGC	285,00	1,98	IIa	86356
**GTC**	129,00	0,89	GAC	288,00	2,00	CAG	469,00	3,25	CTG	394,00	2,73		80741
**GCT**	275,00	1,91	AGC	257,00	1,78	CGA	37,00	0,26	TCG	35,00	0,24		44919
**GCC**	293,00	2,03	GGC	230,00	1,59	CGG	38,00	0,26	CCG	24,00	0,17	IIb	22459
**CCA**	308,00	2,14	TGG	351,00	2,43	GGT	90,00	0,62	ACC	196,00	1,36		45985
**CCT**	306,00	2,12	AGG	444,00	3,08	GGA	513,00	3,56	TCC	149,00	1,03		57215
**CAC**	129,00	0,89	GTG	181,00	1,25	GTG*			CAC*			IIc	
**CGC**	22,00	0,15	GCG	34,00	0,24	GCG*			CGC*				
**CTC**	273,00	1,89	GAG	278,00	1,93	GAG*			CTC*				
**CCC**	227,00	1,57	GGG	263,00	1,82	GGG*			CCC*				

Quadruplet		f1	f2	fu
CGT	CGT(D)	0,29	1,33	1,62
	ACG(RC(D))	0,32	1,98	2,29
	GCA(C(D))	1,33	0,29	1,62
	TGC(R(D))	1,98	0,32	2,29
GTC	GTC(D)	0,89	3,25	4,15
	GAC(RC(D))	2,00	2,73	4,73
	CAG(C(D))	3,25	0,89	4,15
	CTG(R(D))	2,73	2,00	4,73
GCT	GCT(D)	1,91	0,26	2,16
	AGC(RC(D))	1,78	0,24	2,02
	CGA(C(D))	0,26	1,91	2,16
	TCG(R(D))	0,24	1,78	2,02
GCC	GCC(D)	2,03	0,26	2,29
	GGC(RC(D))	1,59	0,17	1,76
	CGG(C(D))	0,26	2,03	2,29
	CCG(R(D))	0,17	1,59	1,76
CCA	CCA(D)	2,14	0,62	2,76
	TGG(RC(D))	2,43	1,36	3,79
	GGT(C(D))	0,62	2,14	2,76
	ACC(R(D))	1,36	2,43	3,79
CCT	CCT(D)	2,12	3,56	5,68
	AGG(RC(D))	3,08	1,03	4,11
	GGA(C(D))	3,56	2,12	5,68
	TCC(R(D))	1,03	3,08	4,11
CAC	CAC(D)	0,45	0,63	1,07
	GTG(RC(D))	0,63	0,45	1,07
	GTG(C(D))	0,63	0,45	1,07
	CAC(R(D))	0,45	0,63	1,07
CGC	CGC(D)	0,08	0,12	0,19
	GCG(RC(D))	0,12	0,08	0,19
	GCG(C(D))	0,12	0,08	0,19
	CGC(R(D))	0,08	0,12	0,19
CTC	CTC(D)	0,95	0,96	1,91
	GAG(RC(D))	0,96	0,95	1,91
	GAG(C(D))	0,96	0,95	1,91
	CTC(R(D))	0,95	0,96	1,91
CCC	CCC(D)	0,79	0,91	1,70
	GGG(RC(D))	0,91	0,79	1,70
	GGG(C(D))	0,91	0,79	1,70
	CCC(R(D))	0,79	0,91	1,70

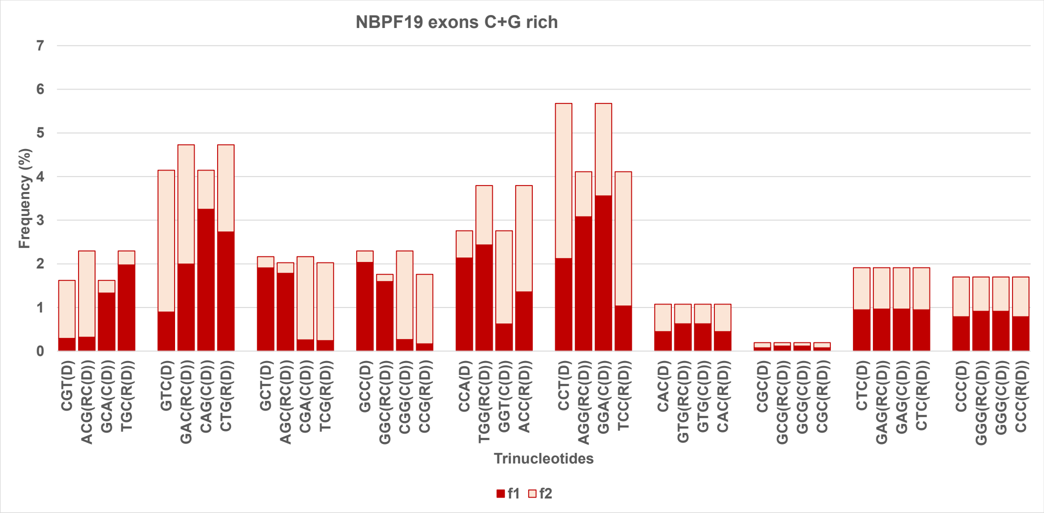

NBPF19 introns

C+G rich												
**D**	**f**	**%**	**RC**	**f**	**%**	**C**	**f**	**%**	**R**	**f**	**%**	
**CGT**	290,00	0,19	ACG	289,00	0,19	GCA	2013,00	1,33	TGC	1867,00	1,23	IIa
**GTC**	1780,00	1,18	GAC	1433,00	0,95	CAG	2787,00	1,84	CTG	3609,00	2,39	
**GCT**	1960,00	1,30	AGC	1801,00	1,19	CGA	331,00	0,22	TCG	283,00	0,19	
**GCC**	1440,00	0,95	GGC	1722,00	1,14	CGG	224,00	0,15	CCG	296,00	0,20	IIb
**CCA**	2236,00	1,48	TGG	3046,00	2,01	GGT	1839,00	1,22	ACC	1667,00	1,10	
**CCT**	3065,00	2,03	AGG	2630,00	1,74	GGA	2339,00	1,55	TCC	2490,00	1,65	
**CAC**	1849,00	1,22	GTG	2922,00	1,93	GTG*			CAC*			IIc
**CGC**	288,00	0,19	GCG	265,00	0,18	GCG*			CGC*			
**CTC**	2999,00	1,98	GAG	2429,00	1,61	GAG*			CTC*			
**CCC**	1595,00	1,05	GGG	2013,00	1,33	GGG*			CCC*			

Quadruplet		f1	f2	fu
CGT	CGT(D)	0,19	1,33	1,52
	ACG(RC(D))	0,19	1,23	1,42
	GCA(C(D))	1,33	0,19	1,52
	TGC(R(D))	1,23	0,19	1,42
GTC	GTC(D)	1,18	1,84	3,02
	GAC(RC(D))	0,95	2,39	3,33
	CAG(C(D))	1,84	1,18	3,02
	CTG(R(D))	2,39	0,95	3,33
GCT	GCT(D)	1,30	0,22	1,51
	AGC(RC(D))	1,19	0,19	1,38
	CGA(C(D))	0,22	1,30	1,51
	TCG(R(D))	0,19	1,19	1,38
GCC	GCC(D)	0,95	0,15	1,10
	GGC(RC(D))	1,14	0,20	1,33
	CGG(C(D))	0,15	0,95	1,10
	CCG(R(D))	0,20	1,14	1,33
CCA	CCA(D)	1,48	1,22	2,69
	TGG(RC(D))	2,01	1,10	3,11
	GGT(C(D))	1,22	1,48	2,69
	ACC(R(D))	1,10	2,01	3,11
CCT	CCT(D)	2,03	1,55	3,57
	AGG(RC(D))	1,74	1,65	3,38
	GGA(C(D))	1,55	2,03	3,57
	TCC(R(D))	1,65	1,74	3,38
CAC	CAC(D)	0,61	0,97	1,58
	GTG(RC(D))	0,97	0,61	1,58
	GTG(C(D))	0,97	0,61	1,58
	CAC(R(D))	0,61	0,97	1,58
CGC	CGC(D)	0,10	0,09	0,18
	GCG(RC(D))	0,09	0,10	0,18
	GCG(C(D))	0,09	0,10	0,18
	CGC(R(D))	0,10	0,09	0,18
CTC	CTC(D)	0,99	0,80	1,79
	GAG(RC(D))	0,80	0,99	1,79
	GAG(C(D))	0,80	0,99	1,79
	CTC(R(D))	0,99	0,80	1,79
CCC	CCC(D)	0,53	0,67	1,19
	GGG(RC(D))	0,67	0,53	1,19
	GGG(C(D))	0,67	0,53	1,19
	CCC(R(D))	0,53	0,67	1,19

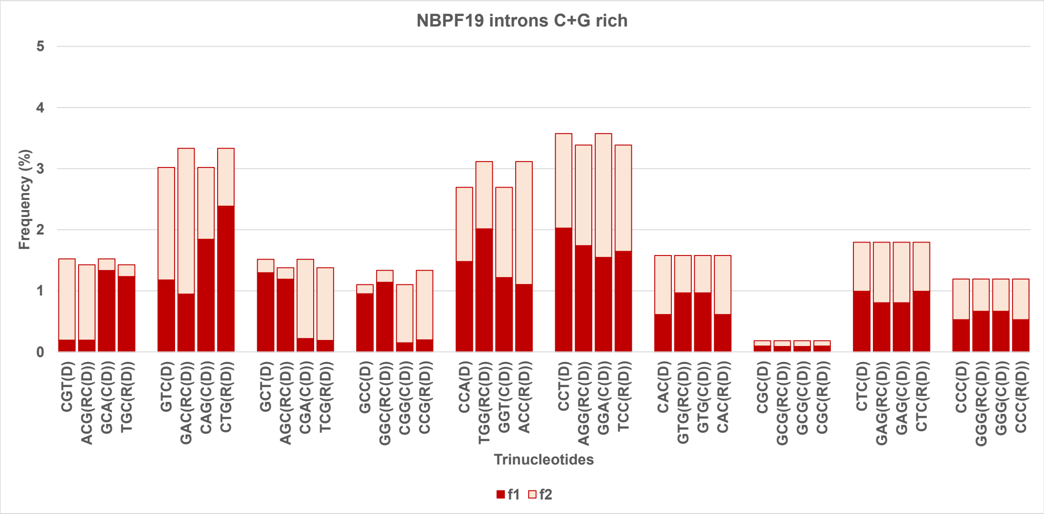

NBPF20 sequence

						A-T rich						
**D**	**f**	**%**	**RC**	**f**	**%**	**C**	**f**	**%**	**R**	**f**	**%**	
**ATG**	2366,00	2,02	CAT	2362,00	2,02	TAC	1270,00	1,08	GTA	960,00	0,82	Ia
**TGA**	3067,00	2,62	TCA	2585,00	2,21	ACT	2210,00	1,89	AGT	1677,00	1,43	
**TAG**	1306,00	1,12	CTA	1624,00	1,39	ATC	1374,00	1,17	GAT	1883,00	1,61	
**TAA**	1255,00	1,07	TTA	1842,00	1,57	ATT	2900,00	2,48	AAT	1800,00	1,54	Ib
**AAC**	1584,00	1,35	GTT	1884,00	1,61	TTG	2537,00	2,17	CAA	1547,00	1,32	
**AAG**	1914,00	1,63	CTT	2565,00	2,19	TTC	2970,00	2,54	GAA	2496,00	2,13	
**ACA**	1578,00	1,35	TGT	3863,00	3,30	TGT*			ACA*			Ic
**ATA**	1292,00	1,10	TAT	1887,00	1,61	TAT*			ATA*			
**AGA**	2039,00	1,74	TCT	3746,00	3,20	TCT*			AGA*			
**AAA**	2288,00	1,95	TTT	4089,00	3,49	TTT*			AAA*			

Quadruplet		f1	f2	fu
ATG	ATG(D)	2,02	1,08	3,11
	CAT(RC(D))	2,02	0,82	2,84
	TAC(C(D))	1,08	2,02	3,11
	GTA(R(D))	0,82	2,02	2,84
TGA	TGA(D)	2,62	1,89	4,51
	TCA(RC(D))	2,21	1,43	3,64
	ACT(C(D))	1,89	2,62	4,51
	AGT(R(D))	1,43	2,21	3,64
TAG	TAG(D)	1,12	1,17	2,29
	CTA(RC(D))	1,39	1,61	3,00
	ATC(C(D))	1,17	1,12	2,29
	GAT(R(D))	1,61	1,39	3,00
TAA	TAA(D)	1,07	2,48	3,55
	TTA(RC(D))	1,57	1,54	3,11
	ATT(C(D))	2,48	1,07	3,55
	AAT(R(D))	1,54	1,57	3,11
AAC	AAC(D)	1,35	2,17	3,52
	GTT(RC(D))	1,61	1,32	2,93
	TTG(C(D))	2,17	1,35	3,52
	CAA(R(D))	1,32	1,61	2,93
AAG	AAG(D)	1,63	2,54	4,17
	CTT(RC(D))	2,19	2,13	4,32
	TTC(C(D))	2,54	1,63	4,17
	GAA(R(D))	2,13	2,19	4,32
ACA	ACA(D)	0,67	1,65	2,32
	TGT(RC(D))	1,65	0,67	2,32
	TGT(C(D))	1,65	0,67	2,32
	ACA(R(D))	0,67	1,65	2,32
ATA	ATA(D)	0,55	0,81	1,36
	TAT(RC(D))	0,81	0,55	1,36
	TAT(C(D))	0,81	0,55	1,36
	ATA(R(D))	0,55	0,55	1,10
AGA	AGA(D)	0,87	1,60	2,47
	TCT(RC(D))	1,60	0,87	2,47
	TCT(C(D))	1,60	0,87	2,47
	AGA(R(D))	0,87	1,60	2,47
AAA	AAA(D)	0,98	1,75	2,72
	TTT(RC(D))	1,75	0,98	2,72
	TTT(C(D))	1,75	0,98	2,72
	AAA(R(D))	0,98	1,75	2,72

Composition of DNA sequence in NBPF20

Base	No.of bases	%
A	28637	24,46%
C	25586	21,85%
G	26037	22,24%
T	36819	31,45%
N	0	0,00%
Sum	117079	100,00%

% of coding bases from NBPF20 (including N bases)

**82,77%**

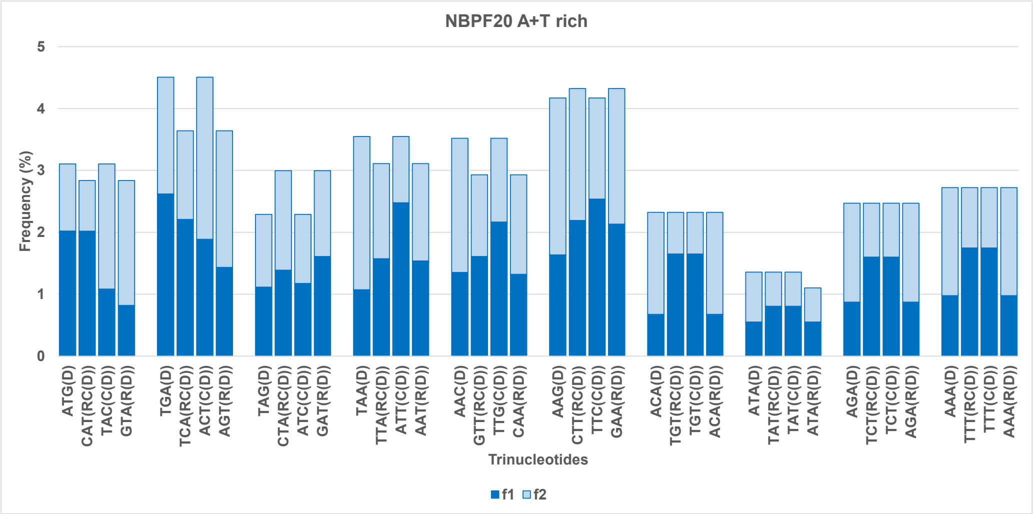

NBPF20 exons

A-T rich

D	f	%	RC	f	%	C	f	%	R	f	%	
**ATG**	345,00	1,84	CAT	252,00	1,34	TAC	158,00	0,84	GTA	130,00	0,69	Ia
**TGA**	444,00	2,37	TCA	400,00	2,13	ACT	405,00	2,16	AGT	298,00	1,59	
**TAG**	160,00	0,85	CTA	113,00	0,60	ATC	128,00	0,68	GAT	265,00	1,41	
**TAA**	53,00	0,28	TTA	223,00	1,19	ATT	235,00	1,25	AAT	96,00	0,51	Ib
**AAC**	263,00	1,40	GTT	286,00	1,52	TTG	496,00	2,64	CAA	253,00	1,35	
**AAG**	872,00	4,65	CTT	422,00	2,25	TTC	224,00	1,19	GAA	925,00	4,93	
**ACA**	242,00	1,29	TGT	242,00	1,29	TGT*			ACA*			Ic
**ATA**	97,00	0,52	TAT	192,00	1,02	TAT*			ATA*			
**AGA**	724,00	3,86	TCT	256,00	1,36	TCT*			AGA*			
**AAA**	551,00	2,94	TTT	237,00	1,26	TTT*			AAA*			

Quadruplet		f1	f2	fu
ATG	ATG(D)	1,84	0,84	2,68
	CAT(RC(D))	1,34	0,69	2,04
	TAC(C(D))	0,84	1,84	2,68
	GTA(R(D))	0,69	1,34	2,04
TGA	TGA(D)	2,37	2,16	4,53
	TCA(RC(D))	2,13	1,59	3,72
	ACT(C(D))	2,16	2,37	4,53
	AGT(R(D))	1,59	2,13	3,72
TAG	TAG(D)	0,85	0,68	1,54
	CTA(RC(D))	0,60	1,41	2,02
	ATC(C(D))	0,68	0,85	1,54
	GAT(R(D))	1,41	0,60	2,02
TAA	TAA(D)	0,28	1,25	1,54
	TTA(RC(D))	1,19	0,51	1,70
	ATT(C(D))	1,25	0,28	1,54
	AAT(R(D))	0,51	1,19	1,70
AAC	AAC(D)	1,40	2,64	4,05
	GTT(RC(D))	1,52	1,35	2,87
	TTG(C(D))	2,64	1,40	4,05
	CAA(R(D))	1,35	1,52	2,87
AAG	AAG(D)	4,65	1,19	5,84
	CTT(RC(D))	2,25	4,93	7,18
	TTC(C(D))	1,19	4,65	5,84
	GAA(R(D))	4,93	2,25	7,18
ACA	ACA(D)	0,65	0,65	1,29
	TGT(RC(D))	0,65	0,65	1,29
	TGT(C(D))	0,65	0,65	1,29
	ACA(R(D))	0,65	0,65	1,29
ATA	ATA(D)	0,26	0,51	0,77
	TAT(RC(D))	0,51	0,26	0,77
	TAT(C(D))	0,51	0,26	0,77
	ATA(R(D))	0,26	0,26	0,52
AGA	AGA(D)	1,93	0,68	2,61
	TCT(RC(D))	0,68	1,93	2,61
	TCT(C(D))	0,68	1,93	2,61
	AGA(R(D))	1,93	0,68	2,61
AAA	AAA(D)	1,47	0,63	2,10
	TTT(RC(D))	0,63	1,47	2,10
	TTT(C(D))	0,63	1,47	2,10
	AAA(R(D))	1,47	0,63	2,10

Composition of DNA sequence in NBPF20 exons

Base	No.of bases	%
A	5555	29,61%
C	3942	21,01%
G	5129	27,34%
T	4134	22,04%
N	0	0,00%
Sum	18760	100,00%

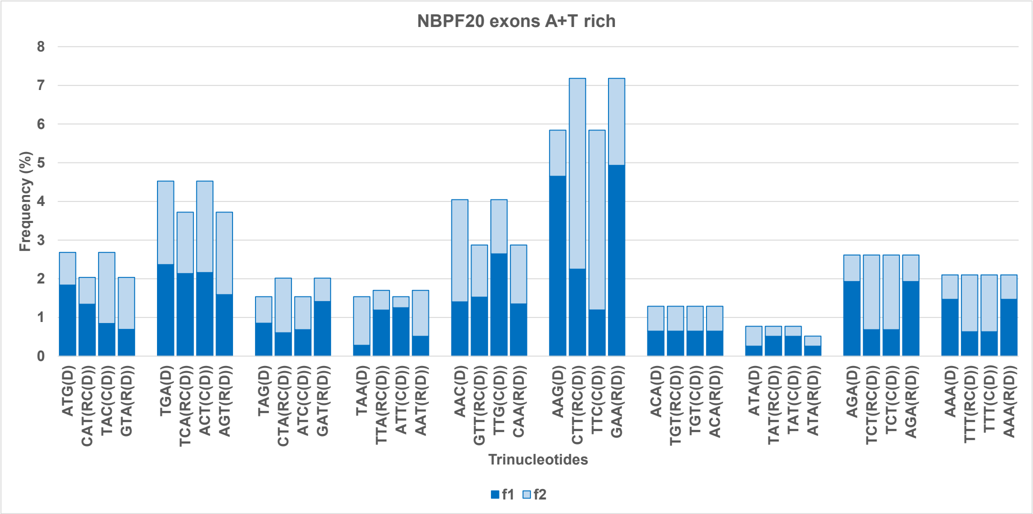

NBPF20 introns

A-T rich

D	f	%	RC	f	%	C	f	%	R	f	%	
**ATG**	1998,00	2,06	CAT	2086,00	2,15	TAC	1092,00	1,13	GTA	815,00	0,84	Ia
**TGA**	2615,00	2,70	TCA	2163,00	2,23	ACT	1784,00	1,84	AGT	1335,00	1,38	
**TAG**	1129,00	1,17	CTA	1492,00	1,54	ATC	1230,00	1,27	GAT	1601,00	1,65	
**TAA**	1180,00	1,22	TTA	1597,00	1,65	ATT	2633,00	2,72	AAT	1671,00	1,72	Ib
**AAC**	1302,00	1,34	GTT	1579,00	1,63	TTG	2010,00	2,07	CAA	1273,00	1,31	
**AAG**	1025,00	1,06	CTT	2118,00	2,19	TTC	2723,00	2,81	GAA	1545,00	1,59	
**ACA**	1307,00	1,35	TGT	3575,00	3,69	TGT*			ACA*			Ic
**ATA**	1166,00	1,20	TAT	1669,00	1,72	TAT*			ATA*			
**AGA**	1288,00	1,33	TCT	3464,00	3,57	TCT*			AGA*			
**AAA**	1667,00	1,72	TTT	3802,00	3,92	TTT*			AAA*			

Quadruplet		f1	f2	fu
ATG	ATG(D)	2,06	1,13	3,19
	CAT(RC(D))	2,15	0,84	2,99
	TAC(C(D))	1,13	2,06	3,19
	GTA(R(D))	0,84	2,15	2,99
TGA	TGA(D)	2,70	1,84	4,54
	TCA(RC(D))	2,23	1,38	3,61
	ACT(C(D))	1,84	2,70	4,54
	AGT(R(D))	1,38	2,23	3,61
TAG	TAG(D)	1,17	1,27	2,43
	CTA(RC(D))	1,54	1,65	3,19
	ATC(C(D))	1,27	1,17	2,43
	GAT(R(D))	1,65	1,54	3,19
TAA	TAA(D)	1,22	2,72	3,93
	TTA(RC(D))	1,65	1,72	3,37
	ATT(C(D))	2,72	1,22	3,93
	AAT(R(D))	1,72	1,65	3,37
AAC	AAC(D)	1,34	2,07	3,42
	GTT(RC(D))	1,63	1,31	2,94
	TTG(C(D))	2,07	1,34	3,42
	CAA(R(D))	1,31	1,63	2,94
AAG	AAG(D)	1,06	2,81	3,87
	CTT(RC(D))	2,19	1,59	3,78
	TTC(C(D))	2,81	1,06	3,87
	GAA(R(D))	1,59	2,19	3,78
ACA	ACA(D)	0,67	1,84	2,52
	TGT(RC(D))	1,84	0,67	2,52
	TGT(C(D))	1,84	0,67	2,52
	ACA(R(D))	0,67	1,84	2,52
ATA	ATA(D)	0,60	0,86	1,46
	TAT(RC(D))	0,86	0,60	1,46
	TAT(C(D))	0,86	0,60	1,46
	ATA(R(D))	0,60	0,60	1,20
AGA	AGA(D)	0,66	1,79	2,45
	TCT(RC(D))	1,79	0,66	2,45
	TCT(C(D))	1,79	0,66	2,45
	AGA(R(D))	0,66	1,79	2,45
AAA	AAA(D)	0,86	1,96	2,82
	TTT(RC(D))	1,96	0,86	2,82
	TTT(C(D))	1,96	0,86	2,82
	AAA(R(D))	0,86	1,96	2,82

Composition of DNA sequence NBPF20 introns

Base	No.of bases	%
A	22682	23,41%
C	21366	22,05%
G	20581	21,24%
T	32279	33,31%
N	0	0,00%
Sum	96908	100,00%

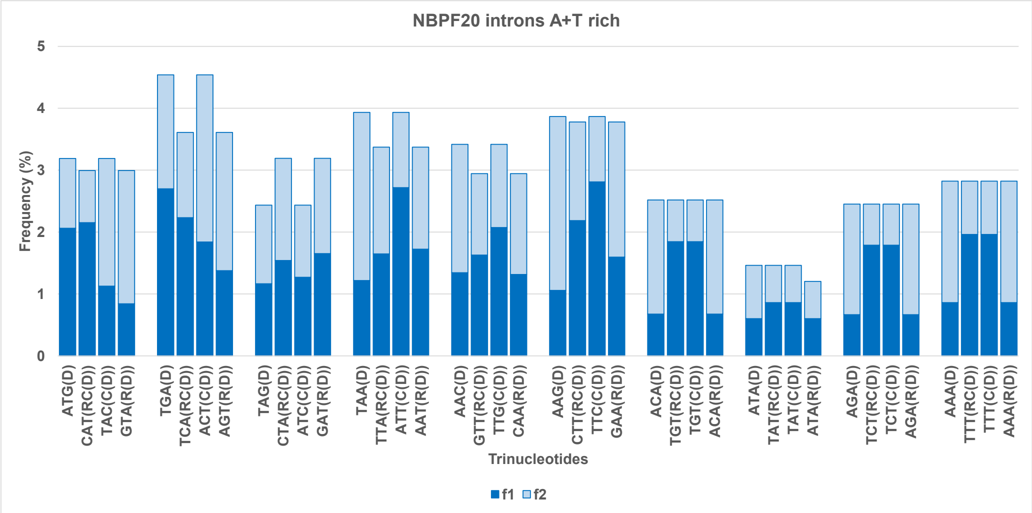

NBPF20 sequence

						C+G rich						
**D**	**f**	**%**	**RC**	**f**	**%**	**C**	**f**	**%**	**R**	**f**	**%**	
**CGT**	264,00	0,23	ACG	315,00	0,27	GCA	1602,00	1,37	TGC	1376,00	1,18	IIa
**GTC**	1670,00	1,43	GAC	1395,00	1,19	CAG	2478,00	2,12	CTG	3393,00	2,90	
**GCT**	1548,00	1,32	AGC	1454,00	1,24	CGA	355,00	0,30	TCG	230,00	0,20	
**GCC**	1297,00	1,11	GGC	1605,00	1,37	CGG	110,00	0,09	CCG	196,00	0,17	IIb
**CCA**	1895,00	1,62	TGG	2473,00	2,11	GGT	1192,00	1,02	ACC	1419,00	1,21	
**CCT**	2948,00	2,52	AGG	2426,00	2,07	GGA	2212,00	1,89	TCC	2323,00	1,98	
**CAC**	1273,00	1,09	GTG	2482,00	2,12	GTG*			CAC*			IIc
**CGC**	195,00	0,17	GCG	183,00	0,16	GCG*			CGC*			
**CTC**	2870,00	2,45	GAG	1898,00	1,62	GAG*			CTC*			
**CCC**	1511,00	1,29	GGG	1729,00	1,48	GGG*			CCC*			

Quadruplet		f1	f2	fu
CGT	CGT(D)	0,23	1,37	1,59
	ACG(RC(D))	0,27	1,18	1,44
	GCA(C(D))	1,37	0,23	1,59
	TGC(R(D))	1,18	0,27	1,44
GTC	GTC(D)	1,43	2,12	3,54
	GAC(RC(D))	1,19	2,90	4,09
	CAG(C(D))	2,12	1,43	3,54
	CTG(R(D))	2,90	1,19	4,09
GCT	GCT(D)	1,32	0,30	1,63
	AGC(RC(D))	1,24	0,20	1,44
	CGA(C(D))	0,30	1,32	1,63
	TCG(R(D))	0,20	1,24	1,44
GCC	GCC(D)	1,11	0,09	1,20
	GGC(RC(D)	1,37	0,17	1,54
	CGG(C(D))	0,09	1,11	1,20
	CCG(R(D))	0,17	1,37	1,54
CCA	CCA(D)	1,62	1,02	2,64
	TGG(RC(D))	2,11	1,21	3,32
	GGT(C(D))	1,02	1,62	2,64
	ACC(R(D))	1,21	2,11	3,32
CCT	CCT(D)	2,52	1,89	4,41
	AGG(RC(D))	2,07	1,98	4,06
	GGA(C(D))	1,89	2,52	4,41
	TCC(R(D))	1,98	2,07	4,06
CAC	CAC(D)	0,54	1,06	1,60
	GTG(RC(D))	1,06	0,54	1,60
	GTG(C(D))	1,06	0,54	1,60
	CAC(R(D))	0,54	1,06	1,60
CGC	CGC(D)	0,08	0,08	0,16
	GCG(RC(D)	0,08	0,08	0,16
	GCG(C(D))	0,08	0,08	0,16
	CGC(R(D))	0,08	0,08	0,16
CTC	CTC(D)	1,23	0,81	2,04
	GAG(RC(D))	0,81	1,23	2,04
	GAG(C(D))	0,81	1,23	2,04
	CTC(R(D))	1,23	0,81	2,04
CCC	CCC(D)	0,65	0,74	1,38
	GGG(RC(D)	0,74	0,65	1,38
	GGG(C(D))	0,74	0,65	1,38
	CCC(R(D))	0,65	0,74	1,38

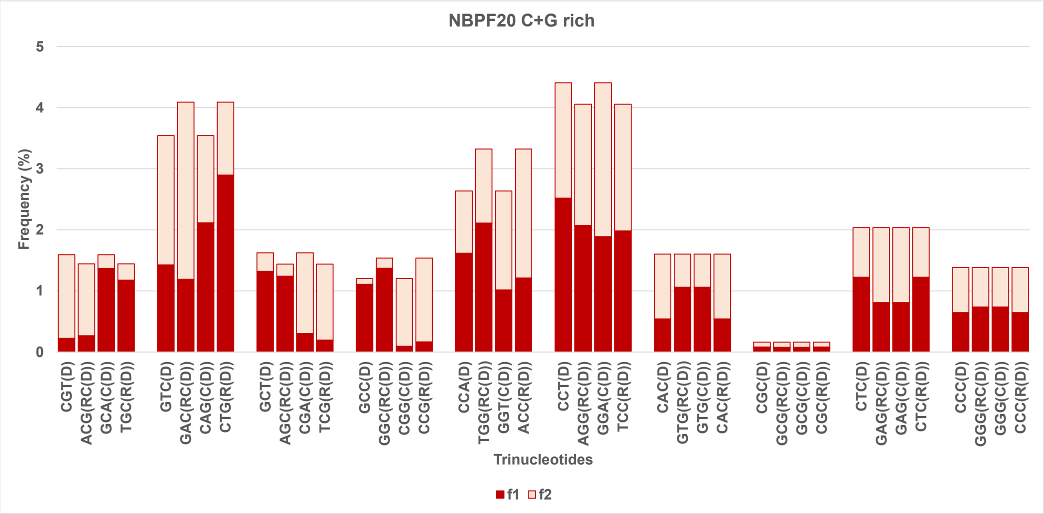

NBPF20 exons

C+G rich

D	f	%	RC	f	%	C	f	%	R	f	%		
**CGT**	96,00	0,51	ACG	82,00	0,44	GCA	241,00	1,28	TGC	365,00	1,95	IIa	86356
**GTC**	153,00	0,82	GAC	385,00	2,05	CAG	623,00	3,32	CTG	504,00	2,69		80741
**GCT**	366,00	1,95	AGC	372,00	1,98	CGA	42,00	0,22	TCG	39,00	0,21		44919
**GCC**	382,00	2,04	GGC	272,00	1,45	CGG	15,00	0,08	CCG	12,00	0,06	IIb	22459
**CCA**	399,00	2,13	TGG	463,00	2,47	GGT	102,00	0,54	ACC	231,00	1,23		45985
**CCT**	384,00	2,05	AGG	613,00	3,27	GGA	717,00	3,82	TCC	182,00	0,97		57215
**CAC**	154,00	0,82	GTG	169,00	0,90	GTG*			CAC*			IIc	
**CGC**	8,00	0,04	GCG	28,00	0,15	GCG*			CGC*				
**CTC**	372,00	1,98	GAG	352,00	1,88	GAG*			CTC*				
**CCC**	292,00	1,56	GGG	356,00	1,90	GGG*			CCC*				

Quadruplet		f1	f2	fu
CGT	CGT(D)	0,51	1,28	1,80
	ACG(RC(D))	0,44	1,95	2,38
	GCA(C(D))	1,28	0,51	1,80
	TGC(R(D))	1,95	0,44	2,38
GTC	GTC(D)	0,82	3,32	4,14
	GAC(RC(D))	2,05	2,69	4,74
	CAG(C(D))	3,32	0,82	4,14
	CTG(R(D))	2,69	2,05	4,74
GCT	GCT(D)	1,95	0,22	2,18
	AGC(RC(D))	1,98	0,21	2,19
	CGA(C(D))	0,22	1,95	2,18
	TCG(R(D))	0,21	1,98	2,19
GCC	GCC(D)	2,04	0,08	2,12
	GGC(RC(D))	1,45	0,06	1,51
	CGG(C(D))	0,08	2,04	2,12
	CCG(R(D))	0,06	1,45	1,51
CCA	CCA(D)	2,13	0,54	2,67
	TGG(RC(D))	2,47	1,23	3,70
	GGT(C(D))	0,54	2,13	2,67
	ACC(R(D))	1,23	2,47	3,70
CCT	CCT(D)	2,05	3,82	5,87
	AGG(RC(D))	3,27	0,97	4,24
	GGA(C(D))	3,82	2,05	5,87
	TCC(R(D))	0,97	3,27	4,24
CAC	CAC(D)	0,41	0,45	0,86
	GTG(RC(D))	0,45	0,41	0,86
	GTG(C(D))	0,45	0,41	0,86
	CAC(R(D))	0,41	0,45	0,86
CGC	CGC(D)	0,02	0,07	0,10
	GCG(RC(D))	0,07	0,02	0,10
	GCG(C(D))	0,07	0,02	0,10
	CGC(R(D))	0,02	0,07	0,10
CTC	CTC(D)	0,99	0,94	1,93
	GAG(RC(D))	0,94	0,99	1,93
	GAG(C(D))	0,94	0,99	1,93
	CTC(R(D))	0,99	0,94	1,93
CCC	CCC(D)	0,78	0,95	1,73
	GGG(RC(D))	0,95	0,78	1,73
	GGG(C(D))	0,95	0,78	1,73
	CCC(R(D))	0,78	0,95	1,73

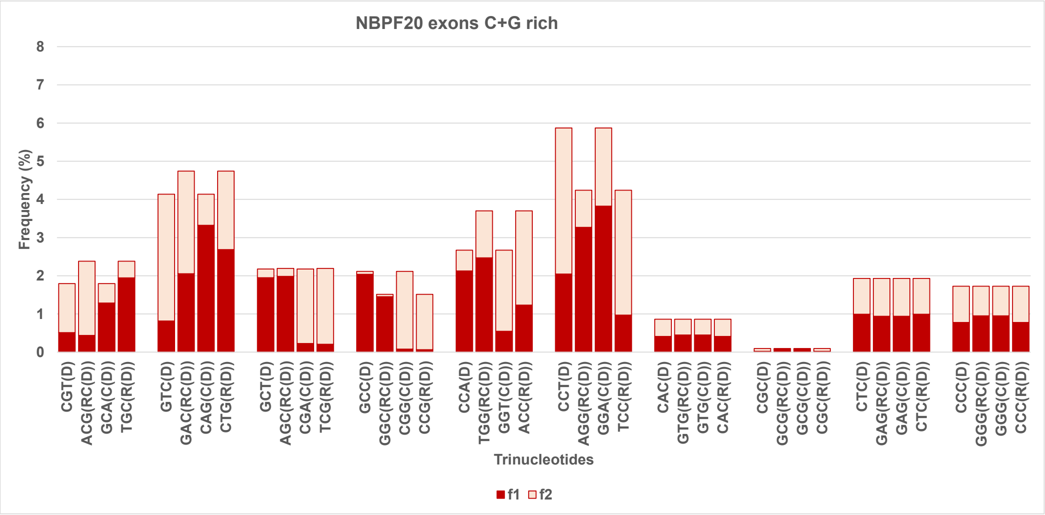

NBPF20 introns

C+G rich

D	f	%	RC	f	%	C	f	%	R	f	%	
**CGT**	161,00	0,17	ACG	227,00	0,23	GCA	1341,00	1,38	TGC	992,00	1,02	IIa
**GTC**	1499,00	1,55	GAC	997,00	1,03	CAG	1829,00	1,89	CTG	2862,00	2,95	
**GCT**	1169,00	1,21	AGC	1067,00	1,10	CGA	305,00	0,31	TCG	185,00	0,19	
**GCC**	897,00	0,93	GGC	1306,00	1,35	CGG	82,00	0,08	CCG	178,00	0,18	IIb
**CCA**	1474,00	1,52	TGG	1959,00	2,02	GGT	1092,00	1,13	ACC	1170,00	1,21	
**CCT**	2539,00	2,62	AGG	1811,00	1,87	GGA	1454,00	1,50	TCC	2124,00	2,19	
**CAC**	1097,00	1,13	GTG	2271,00	2,34	GTG*			CAC*			IIc
**CGC**	183,00	0,19	GCG	141,00	0,15	GCG*			CGC*			
**CTC**	2484,00	2,56	GAG	1519,00	1,57	GAG*			CTC*			
**CCC**	1203,00	1,24	GGG	1354,00	1,40	GGG*			CCC*			

Quadruplet		f1	f2	fu
CGT	CGT(D)	0,17	1,38	1,55
	ACG(RC(D))	0,23	1,02	1,26
	GCA(C(D))	1,38	0,17	1,55
	TGC(R(D))	1,02	0,23	1,26
GTC	GTC(D)	1,55	1,89	3,43
	GAC(RC(D))	1,03	2,95	3,98
	CAG(C(D))	1,89	1,55	3,43
	CTG(R(D))	2,95	1,03	3,98
GCT	GCT(D)	1,21	0,31	1,52
	AGC(RC(D))	1,10	0,19	1,29
	CGA(C(D))	0,31	1,21	1,52
	TCG(R(D))	0,19	1,10	1,29
GCC	GCC(D)	0,93	0,08	1,01
	GGC(RC(D))	1,35	0,18	1,53
	CGG(C(D))	0,08	0,93	1,01
	CCG(R(D))	0,18	1,35	1,53
CCA	CCA(D)	1,52	1,13	2,65
	TGG(RC(D))	2,02	1,21	3,23
	GGT(C(D))	1,13	1,52	2,65
	ACC(R(D))	1,21	2,02	3,23
CCT	CCT(D)	2,62	1,50	4,12
	AGG(RC(D))	1,87	2,19	4,06
	GGA(C(D))	1,50	2,62	4,12
	TCC(R(D))	2,19	1,87	4,06
CAC	CAC(D)	0,57	1,17	1,74
	GTG(RC(D))	1,17	0,57	1,74
	GTG(C(D))	1,17	0,57	1,74
	CAC(R(D))	0,57	1,17	1,74
CGC	CGC(D)	0,09	0,07	0,17
	GCG(RC(D))	0,07	0,09	0,17
	GCG(C(D))	0,07	0,09	0,17
	CGC(R(D))	0,09	0,07	0,17
CTC	CTC(D)	1,28	0,78	2,07
	GAG(RC(D))	0,78	1,28	2,07
	GAG(C(D))	0,78	1,28	2,07
	CTC(R(D))	1,28	0,78	2,07
CCC	CCC(D)	0,62	0,70	1,32
	GGG(RC(D))	0,70	0,62	1,32
	GGG(C(D))	0,70	0,62	1,32
	CCC(R(D))	0,62	0,70	1,32

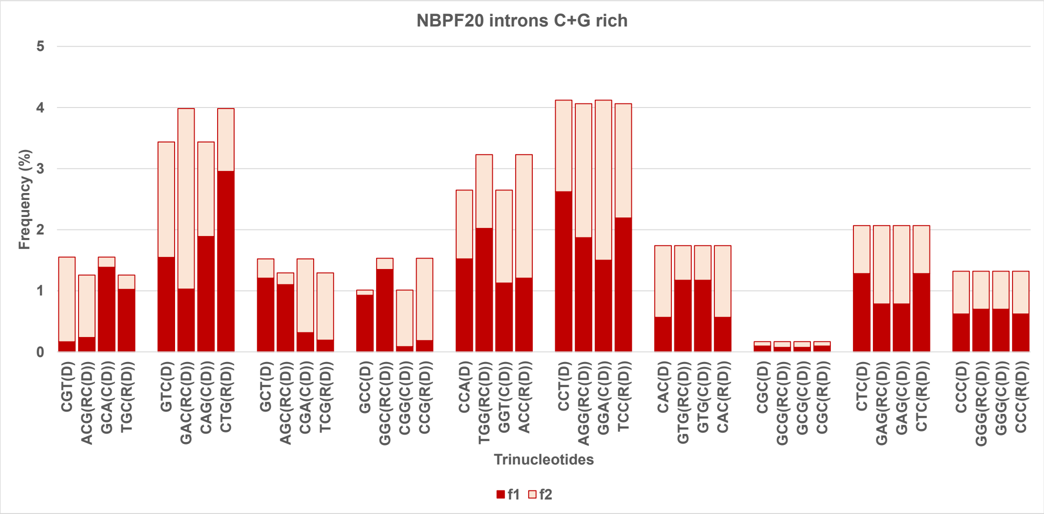

						A-T rich						
**D**	**f**	**%**	**RC**	**f**	**%**	**C**	**f**	**%**	**R**	**f**	**%**	
**ATG**	452,00	2,00	CAT	421,00	1,86	TAC	227,00	1,00	GTA	234,00	1,03	Ia
**TGA**	472,00	2,08	TCA	478,00	2,11	ACT	334,00	1,47	AGT	364,00	1,61	
**TAG**	275,00	1,21	CTA	265,00	1,17	ATC	318,00	1,40	GAT	300,00	1,32	
**TAA**	341,00	1,51	TTA	339,00	1,50	ATT	436,00	1,92	AAT	491,00	2,17	Ib
**AAC**	321,00	1,42	GTT	289,00	1,28	TTG	376,00	1,66	CAA	453,00	2,00	
**AAG**	442,00	1,95	CTT	461,00	2,04	TTC	471,00	2,08	GAA	460,00	2,03	
**ACA**	483,00	2,13	TGT	497,00	2,19	TGT*			ACA*			Ic
**ATA**	345,00	1,52	TAT	340,00	1,50	TAT*			ATA*			
**AGA**	513,00	2,26	TCT	558,00	2,46	TCT*			AGA*			
**AAA**	688,00	3,04	TTT	644,00	2,84	TTT*			AAA*			

Quadruplet		f1	f2	fu
ATG	ATG(D)	2,00	1,00	3,00
	CAT(RC(D))	1,86	1,03	2,89
	TAC(C(D))	1,00	2,00	3,00
	GTA(R(D))	1,03	1,86	2,89
TGA	TGA(D)	2,08	1,47	3,56
	TCA(RC(D))	2,11	1,61	3,72
	ACT(C(D))	1,47	2,08	3,56
	AGT(R(D))	1,61	2,11	3,72
TAG	TAG(D)	1,21	1,40	2,62
	CTA(RC(D))	1,17	1,32	2,49
	ATC(C(D))	1,40	1,21	2,62
	GAT(R(D))	1,32	1,17	2,49
TAA	TAA(D)	1,51	1,92	3,43
	TTA(RC(D))	1,50	2,17	3,66
	ATT(C(D))	1,92	1,51	3,43
	AAT(R(D))	2,17	1,50	3,66
AAC	AAC(D)	1,42	1,66	3,08
	GTT(RC(D))	1,28	2,00	3,28
	TTG(C(D))	1,66	1,42	3,08
	CAA(R(D))	2,00	1,28	3,28
AAG	AAG(D)	1,95	2,08	4,03
	CTT(RC(D))	2,04	2,03	4,07
	TTC(C(D))	2,08	1,95	4,03
	GAA(R(D))	2,03	2,04	4,07
ACA	ACA(D)	1,07	1,10	2,16
	TGT(RC(D))	1,10	1,07	2,16
	TGT(C(D))	1,10	1,07	2,16
	ACA(R(D))	1,07	1,10	2,16
ATA	ATA(D)	0,76	0,75	1,51
	TAT(RC(D))	0,75	0,76	1,51
	TAT(C(D))	0,75	0,76	1,51
	ATA(R(D))	0,76	0,76	1,52
AGA	AGA(D)	1,13	1,23	2,36
	TCT(RC(D))	1,23	1,13	2,36
	TCT(C(D))	1,23	1,13	2,36
	AGA(R(D))	1,13	1,23	2,36
AAA	AAA(D)	1,52	1,42	2,94
	TTT(RC(D))	1,42	1,52	2,94
	TTT(C(D))	1,42	1,52	2,94
	AAA(R(D))	1,52	1,42	2,94

Composition of DNA sequence in NBPF21P

Base	No.of bases	%
A	6340	27,99%
C	5040	22,25%
G	4987	22,02%
T	6285	27,75%
N	0	0,00%
Sum	22652	100,00%

% of coding bases from NBPF21P (including N bases)

**88,30%**

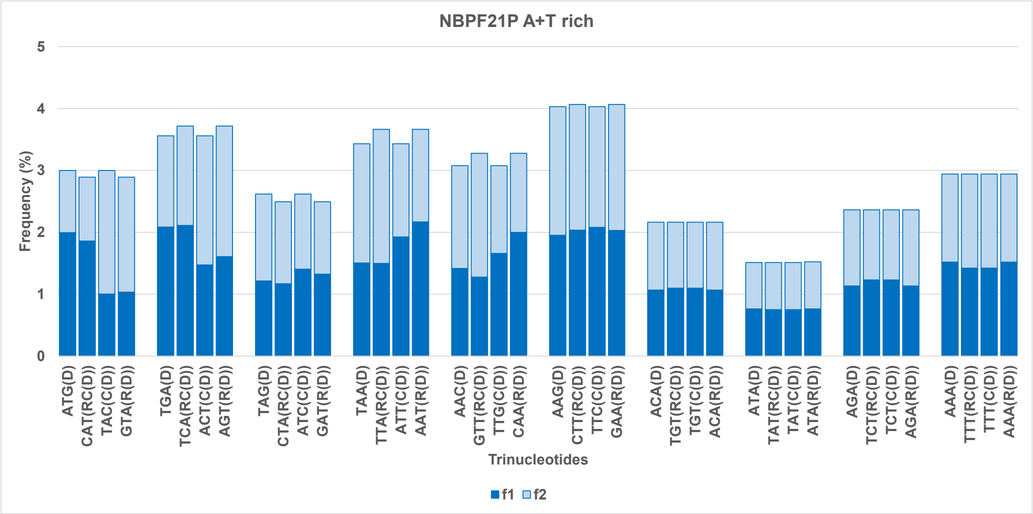

NBPF21P exons

A-T rich

D	f	%	RC	f	%	C	f	%	R	f	%	
**ATG**	36,00	2,48	CAT	14,00	0,97	TAC	6,00	0,41	GTA	8,00	0,55	Ia
**TGA**	46,00	3,17	TCA	37,00	2,55	ACT	18,00	1,24	AGT	29,00	2,00	
**TAG**	9,00	0,62	CTA	7,00	0,48	ATC	14,00	0,97	GAT	23,00	1,59	
**TAA**	6,00	0,41	TTA	13,00	0,90	ATT	16,00	1,10	AAT	23,00	1,59	Ib
**AAC**	16,00	1,10	GTT	14,00	0,97	TTG	24,00	1,66	CAA	28,00	1,93	
**AAG**	43,00	2,97	CTT	29,00	2,00	TTC	22,00	1,52	GAA	48,00	3,31	
**ACA**	22,00	1,52	TGT	21,00	1,45	TGT*			ACA*			Ic
**ATA**	5,00	0,35	TAT	11,00	0,76	TAT*			ATA*			
**AGA**	41,00	2,83	TCT	25,00	1,73	TCT*			AGA*			
**AAA**	35,00	2,42	TTT	8,00	0,55	TTT*			AAA*			

Quadruplet		f1	f2	fu
ATG	ATG(D)	2,48	0,41	2,90
	CAT(RC(D))	0,97	0,55	1,52
	TAC(C(D))	0,41	2,48	2,90
	GTA(R(D))	0,55	0,97	1,52
TGA	TGA(D)	3,17	1,24	4,42
	TCA(RC(D))	2,55	2,00	4,55
	ACT(C(D))	1,24	3,17	4,42
	AGT(R(D))	2,00	2,55	4,55
TAG	TAG(D)	0,62	0,97	1,59
	CTA(RC(D))	0,48	1,59	2,07
	ATC(C(D))	0,97	0,62	1,59
	GAT(R(D))	1,59	0,48	2,07
TAA	TAA(D)	0,41	1,10	1,52
	TTA(RC(D))	0,90	1,59	2,48
	ATT(C(D))	1,10	0,41	1,52
	AAT(R(D))	1,59	0,90	2,48
AAC	AAC(D)	1,10	1,66	2,76
	GTT(RC(D))	0,97	1,93	2,90
	TTG(C(D))	1,66	1,10	2,76
	CAA(R(D))	1,93	0,97	2,90
AAG	AAG(D)	2,97	1,52	4,49
	CTT(RC(D))	2,00	3,31	5,31
	TTC(C(D))	1,52	2,97	4,49
	GAA(R(D))	3,31	2,00	5,31
ACA	ACA(D)	0,76	0,72	1,48
	TGT(RC(D))	0,72	0,76	1,48
	TGT(C(D))	0,72	0,76	1,48
	ACA(R(D))	0,76	0,72	1,48
ATA	ATA(D)	0,17	0,38	0,55
	TAT(RC(D))	0,38	0,17	0,55
	TAT(C(D))	0,38	0,17	0,55
	ATA(R(D))	0,17	0,17	0,35
AGA	AGA(D)	1,41	0,86	2,28
	TCT(RC(D))	0,86	1,41	2,28
	TCT(C(D))	0,86	1,41	2,28
	AGA(R(D))	1,41	0,86	2,28
AAA	AAA(D)	1,21	0,28	1,48
	TTT(RC(D))	0,28	1,21	1,48
	TTT(C(D))	0,28	1,21	1,48
	AAA(R(D))	1,21	0,28	1,48

Composition of DNA sequence in NBPF21P exons

Base	No.of bases	%
A	408	28,12%
C	331	22,81%
G	400	27,57%
T	312	21,50%
N	0	0,00%
Sum	1451	100,00%

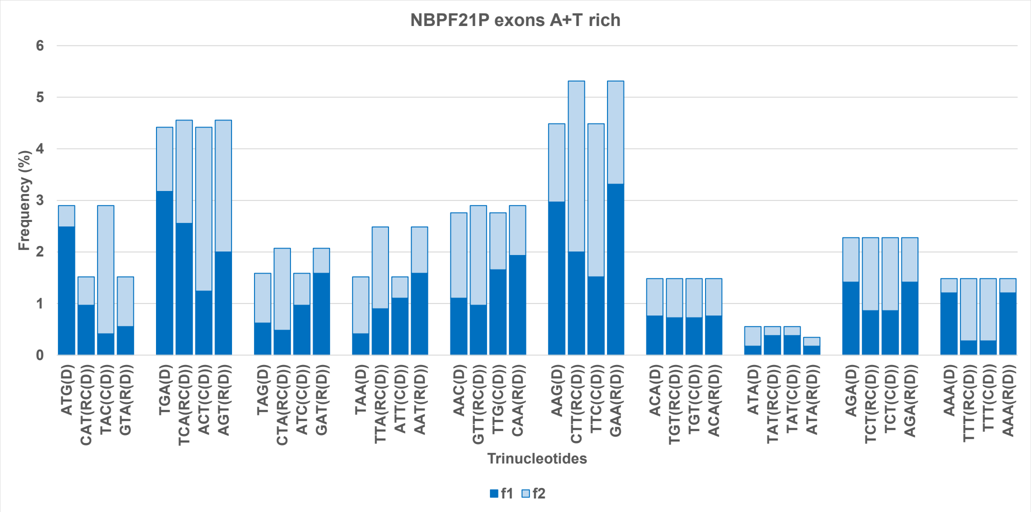

NBPF21P introns

A-T rich

D	f	%	RC	f	%	C	f	%	R	f	%	
**ATG**	398,00	1,99	CAT	384,00	1,92	TAC	202,00	1,01	GTA	216,00	1,08	Ia
**TGA**	400,00	2,00	TCA	419,00	2,10	ACT	297,00	1,49	AGT	317,00	1,59	
**TAG**	250,00	1,25	CTA	245,00	1,23	ATC	288,00	1,44	GAT	262,00	1,31	
**TAA**	318,00	1,59	TTA	302,00	1,51	ATT	394,00	1,97	AAT	441,00	2,21	Ib
**AAC**	291,00	1,46	GTT	258,00	1,29	TTG	333,00	1,67	CAA	400,00	2,00	
**AAG**	381,00	1,91	CTT	403,00	2,02	TTC	420,00	2,10	GAA	395,00	1,98	
**ACA**	434,00	2,17	TGT	454,00	2,27	TGT*			ACA*			Ic
**ATA**	325,00	1,63	TAT	318,00	1,59	TAT*			ATA*			
**AGA**	447,00	2,24	TCT	495,00	2,48	TCT*			AGA*			
**AAA**	630,00	3,15	TTT	590,00	2,95	TTT*			AAA*			

Quadruplet		f1	f2	fu
ATG	ATG(D)	1,99	1,01	3,00
	CAT(RC(D))	1,92	1,08	3,00
	TAC(C(D))	1,01	1,99	3,00
	GTA(R(D))	1,08	1,92	3,00
TGA	TGA(D)	2,00	1,49	3,49
	TCA(RC(D))	2,10	1,59	3,68
	ACT(C(D))	1,49	2,00	3,49
	AGT(R(D))	1,59	2,10	3,68
TAG	TAG(D)	1,25	1,44	2,69
	CTA(RC(D))	1,23	1,31	2,54
	ATC(C(D))	1,44	1,25	2,69
	GAT(R(D))	1,31	1,23	2,54
TAA	TAA(D)	1,59	1,97	3,56
	TTA(RC(D))	1,51	2,21	3,72
	ATT(C(D))	1,97	1,59	3,56
	AAT(R(D))	2,21	1,51	3,72
AAC	AAC(D)	1,46	1,67	3,12
	GTT(RC(D))	1,29	2,00	3,29
	TTG(C(D))	1,67	1,46	3,12
	CAA(R(D))	2,00	1,29	3,29
AAG	AAG(D)	1,91	2,10	4,01
	CTT(RC(D))	2,02	1,98	3,99
	TTC(C(D))	2,10	1,91	4,01
	GAA(R(D))	1,98	2,02	3,99
ACA	ACA(D)	1,09	1,14	2,22
	TGT(RC(D))	1,14	1,09	2,22
	TGT(C(D))	1,14	1,09	2,22
	ACA(R(D))	1,09	1,14	2,22
ATA	ATA(D)	0,81	0,80	1,61
	TAT(RC(D))	0,80	0,81	1,61
	TAT(C(D))	0,80	0,81	1,61
	ATA(R(D))	0,81	0,81	1,63
AGA	AGA(D)	1,12	1,24	2,36
	TCT(RC(D))	1,24	1,12	2,36
	TCT(C(D))	1,24	1,12	2,36
	AGA(R(D))	1,12	1,24	2,36
AAA	AAA(D)	1,58	1,48	3,05
	TTT(RC(D))	1,48	1,58	3,05
	TTT(C(D))	1,48	1,58	3,05
	AAA(R(D))	1,58	1,48	3,05

Composition of DNA sequence NBPF21P introns

Base	No.of bases	%
A	5633	28,16%
C	4412	22,06%
G	4348	21,74%
T	5608	28,04%
N	0	0,00%
Sum	20001	100,00%

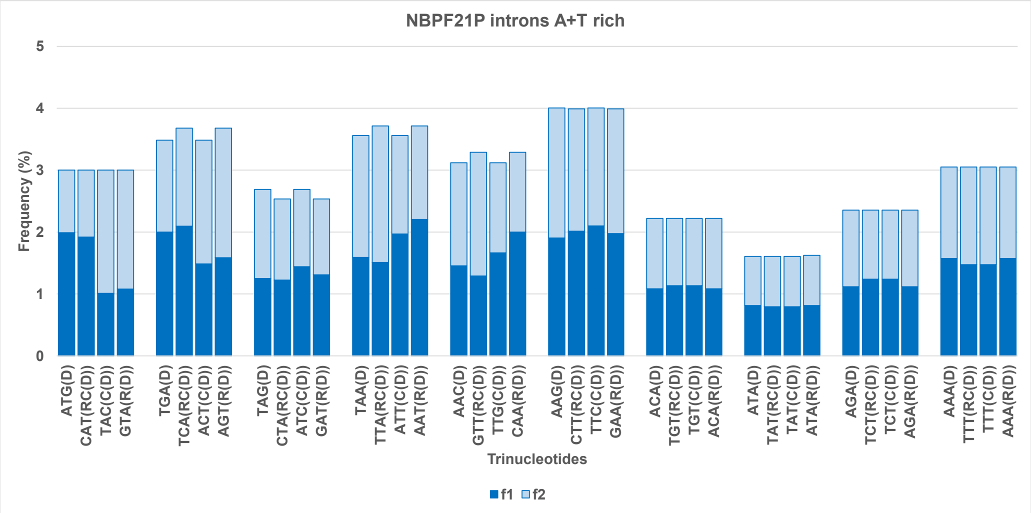

NBPF21P sequence

						C+G rich						
**D**	**f**	**%**	**RC**	**f**	**%**	**C**	**f**	**%**	**R**	**f**	**%**	
**CGT**	39,00	0,17	ACG	44,00	0,19	GCA	366,00	1,62	TGC	341,00	1,51	IIa
**GTC**	239,00	1,06	GAC	249,00	1,10	CAG	503,00	2,22	CTG	515,00	2,27	
**GCT**	339,00	1,50	AGC	334,00	1,47	CGA	54,00	0,24	TCG	59,00	0,26	
**GCC**	267,00	1,18	GGC	284,00	1,25	CGG	74,00	0,33	CCG	77,00	0,34	IIb
**CCA**	430,00	1,90	TGG	448,00	1,98	GGT	277,00	1,22	ACC	316,00	1,40	
**CCT**	494,00	2,18	AGG	458,00	2,02	GGA	419,00	1,85	TCC	418,00	1,85	
**CAC**	380,00	1,68	GTG	415,00	1,83	GTG*			CAC*			IIc
**CGC**	66,00	0,29	GCG	53,00	0,23	GCG*			CGC*			
**CTC**	484,00	2,14	GAG	449,00	1,98	GAG*			CTC*			
**CCC**	324,00	1,43	GGG	347,00	1,53	GGG*			CCC*			

Quadruplet		f1	f2	fu
CGT	CGT(D)	0,17	1,62	1,79
	ACG(RC(D))	0,19	1,51	1,70
	GCA(C(D))	1,62	0,17	1,79
	TGC(R(D))	1,51	0,19	1,70
GTC	GTC(D)	1,06	2,22	3,28
	GAC(RC(D))	1,10	2,27	3,37
	CAG(C(D))	2,22	1,06	3,28
	CTG(R(D))	2,27	1,10	3,37
GCT	GCT(D)	1,50	0,24	1,74
	AGC(RC(D))	1,47	0,26	1,74
	CGA(C(D))	0,24	1,50	1,74
	TCG(R(D))	0,26	1,47	1,74
GCC	GCC(D)	1,18	0,33	1,51
	GGC(RC(D))	1,25	0,34	1,59
	CGG(C(D))	0,33	1,18	1,51
	CCG(R(D))	0,34	1,25	1,59
CCA	CCA(D)	1,90	1,22	3,12
	TGG(RC(D))	1,98	1,40	3,37
	GGT(C(D))	1,22	1,90	3,12
	ACC(R(D))	1,40	1,98	3,37
CCT	CCT(D)	2,18	1,85	4,03
	AGG(RC(D))	2,02	1,85	3,87
	GGA(C(D))	1,85	2,18	4,03
	TCC(R(D))	1,85	2,02	3,87
CAC	CAC(D)	0,84	0,92	1,75
	GTG(RC(D))	0,92	0,84	1,75
	GTG(C(D))	0,92	0,84	1,75
	CAC(R(D))	0,84	0,92	1,75
CGC	CGC(D)	0,15	0,12	0,26
	GCG(RC(D))	0,12	0,15	0,26
	GCG(C(D))	0,12	0,15	0,26
	CGC(R(D))	0,15	0,12	0,26
CTC	CTC(D)	1,07	0,99	2,06
	GAG(RC(D))	0,99	1,07	2,06
	GAG(C(D))	0,99	1,07	2,06
	CTC(R(D))	1,07	0,99	2,06
CCC	CCC(D)	0,72	0,77	1,48
	GGG(RC(D))	0,77	0,72	1,48
	GGG(C(D))	0,77	0,72	1,48
	CCC(R(D))	0,72	0,77	1,48

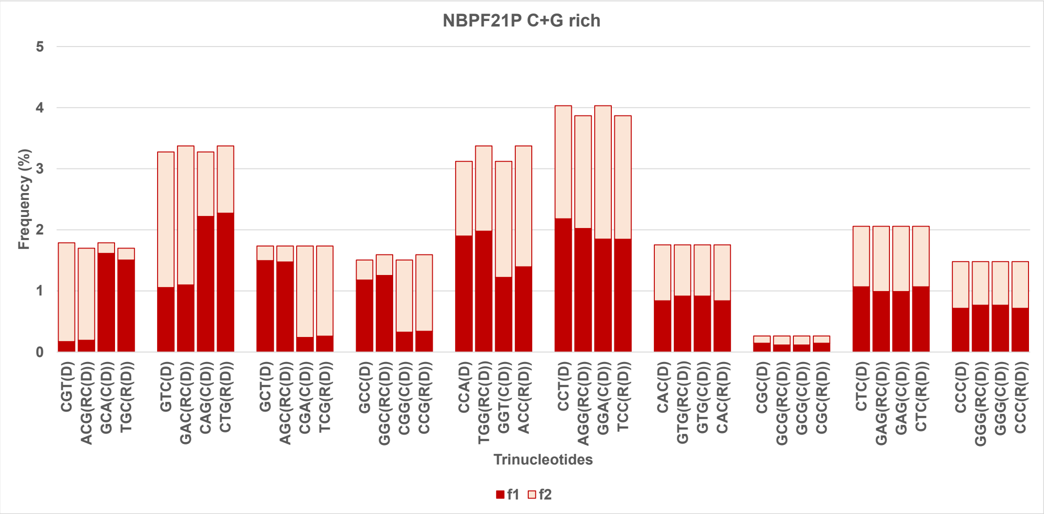

NBPF21P exons

C+G rich

D	f	%	RC	f	%	C	f	%	R	f	%		
			ACG	3,00	0,21	GCA	31,00	2,14	TGC	29,00	2,00	IIa	86356
**GTC**	18,00	1,24	GAC	18,00	1,24	CAG	52,00	3,59	CTG	40,00	2,76		80741
**GCT**	33,00	2,28	AGC	36,00	2,48	CGA	8,00	0,55	TCG	4,00	0,28		44919
**GCC**	24,00	1,66	GGC	23,00	1,59	CGG	5,00	0,35	CCG	6,00	0,41	IIb	22459
**CCA**	30,00	2,07	TGG	31,00	2,14	GGT	17,00	1,17	ACC	23,00	1,59		45985
**CCT**	30,00	2,07	AGG	47,00	3,24	GGA	43,00	2,97	TCC	19,00	1,31		57215
**CAC**	26,00	1,79	GTG	27,00	1,86	GTG*			CAC*			IIc	
**CGC**	4,00	0,28	GCG	4,00	0,28	GCG*			CGC*				
**CTC**	30,00	2,07	GAG	49,00	3,38	GAG*			CTC*				
**CCC**	22,00	1,52	GGG	20,00	1,38	GGG*			CCC*				

Quadruplet		f1	f2	fu
CGT	CGT(D)	0,00	2,14	2,14
	ACG(RC(D))	0,21	2,00	2,21
	GCA(C(D))	2,14	0,00	2,14
	TGC(R(D))	2,00	0,21	2,21
GTC	GTC(D)	1,24	3,59	4,83
	GAC(RC(D))	1,24	2,76	4,00
	CAG(C(D))	3,59	1,24	4,83
	CTG(R(D))	2,76	1,24	4,00
GCT	GCT(D)	2,28	0,55	2,83
	AGC(RC(D))	2,48	0,28	2,76
	CGA(C(D))	0,55	2,28	2,83
	TCG(R(D))	0,28	2,48	2,76
GCC	GCC(D)	1,66	0,35	2,00
	GGC(RC(D))	1,59	0,41	2,00
	CGG(C(D))	0,35	1,66	2,00
	CCG(R(D))	0,41	1,59	2,00
CCA	CCA(D)	2,07	1,17	3,24
	TGG(RC(D))	2,14	1,59	3,73
	GGT(C(D))	1,17	2,07	3,24
	ACC(R(D))	1,59	2,14	3,73
CCT	CCT(D)	2,07	2,97	5,04
	AGG(RC(D))	3,24	1,31	4,55
	GGA(C(D))	2,97	2,07	5,04
	TCC(R(D))	1,31	3,24	4,55
CAC	CAC(D)	0,90	0,93	1,83
	GTG(RC(D))	0,93	0,90	1,83
	GTG(C(D))	0,93	0,90	1,83
	CAC(R(D))	0,90	0,93	1,83
CGC	CGC(D)	0,14	0,14	0,28
	GCG(RC(D))	0,14	0,14	0,28
	GCG(C(D))	0,14	0,14	0,28
	CGC(R(D))	0,14	0,14	0,28
CTC	CTC(D)	1,04	1,69	2,73
	GAG(RC(D))	1,69	1,04	2,73
	GAG(C(D))	1,69	1,04	2,73
	CTC(R(D))	1,04	1,69	2,73
CCC	CCC(D)	0,76	0,69	1,45
	GGG(RC(D))	0,69	0,76	1,45
	GGG(C(D))	0,69	0,76	1,45
	CCC(R(D))	0,76	0,69	1,45

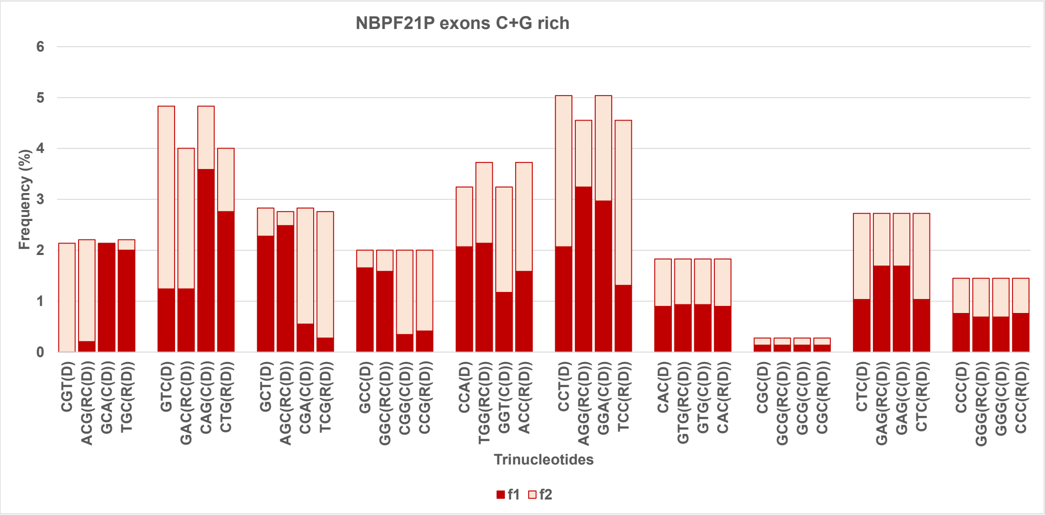

NBPF21P introns

C+G rich

D	f	%	RC	f	%	C	f	%	R	f	%	
**CGT**	37,00	0,19	ACG	40,00	0,20	GCA	318,00	1,59	TGC	295,00	1,48	IIa
**GTC**	202,00	1,01	GAC	219,00	1,10	CAG	425,00	2,13	CTG	440,00	2,20	
**GCT**	291,00	1,46	AGC	284,00	1,42	CGA	46,00	0,23	TCG	53,00	0,27	
**GCC**	230,00	1,15	GGC	245,00	1,23	CGG	66,00	0,33	CCG	71,00	0,36	IIb
**CCA**	373,00	1,87	TGG	393,00	1,97	GGT	239,00	1,20	ACC	276,00	1,38	
**CCT**	428,00	2,14	AGG	389,00	1,95	GGA	364,00	1,82	TCC	366,00	1,83	
**CAC**	335,00	1,68	GTG	371,00	1,86	GTG*			CAC*			IIc
**CGC**	62,00	0,31	GCG	47,00	0,24	GCG*			CGC*			
**CTC**	423,00	2,12	GAG	381,00	1,91	GAG*			CTC*			
**CCC**	274,00	1,37	GGG	309,00	1,55	GGG*			CCC*			

Quadruplet		f1	f2	fu
CGT	CGT(D)	0,19	1,59	1,78
	ACG(RC(D))	0,20	1,48	1,68
	GCA(C(D))	1,59	0,19	1,78
	TGC(R(D))	1,48	0,20	1,68
GTC	GTC(D)	1,01	2,13	3,14
	GAC(RC(D))	1,10	2,20	3,30
	CAG(C(D))	2,13	1,01	3,14
	CTG(R(D))	2,20	1,10	3,30
GCT	GCT(D)	1,46	0,23	1,69
	AGC(RC(D))	1,42	0,27	1,69
	CGA(C(D))	0,23	1,46	1,69
	TCG(R(D))	0,27	1,42	1,69
GCC	GCC(D)	1,15	0,33	1,48
	GGC(RC(D))	1,23	0,36	1,58
	CGG(C(D))	0,33	1,15	1,48
	CCG(R(D))	0,36	1,23	1,58
CCA	CCA(D)	1,87	1,20	3,06
	TGG(RC(D))	1,97	1,38	3,35
	GGT(C(D))	1,20	1,87	3,06
	ACC(R(D))	1,38	1,97	3,35
CCT	CCT(D)	2,14	1,82	3,96
	AGG(RC(D))	1,95	1,83	3,78
	GGA(C(D))	1,82	2,14	3,96
	TCC(R(D))	1,83	1,95	3,78
CAC	CAC(D)	0,84	0,93	1,77
	GTG(RC(D))	0,93	0,84	1,77
	GTG(C(D))	0,93	0,84	1,77
	CAC(R(D))	0,84	0,93	1,77
CGC	CGC(D)	0,16	0,12	0,27
	GCG(RC(D))	0,12	0,16	0,27
	GCG(C(D))	0,12	0,16	0,27
	CGC(R(D))	0,16	0,12	0,27
CTC	CTC(D)	1,06	0,95	2,01
	GAG(RC(D))	0,95	1,06	2,01
	GAG(C(D))	0,95	1,06	2,01
	CTC(R(D))	1,06	0,95	2,01
CCC	CCC(D)	0,69	0,77	1,46
	GGG(RC(D))	0,77	0,69	1,46
	GGG(C(D))	0,77	0,69	1,46
	CCC(R(D))	0,69	0,77	1,46

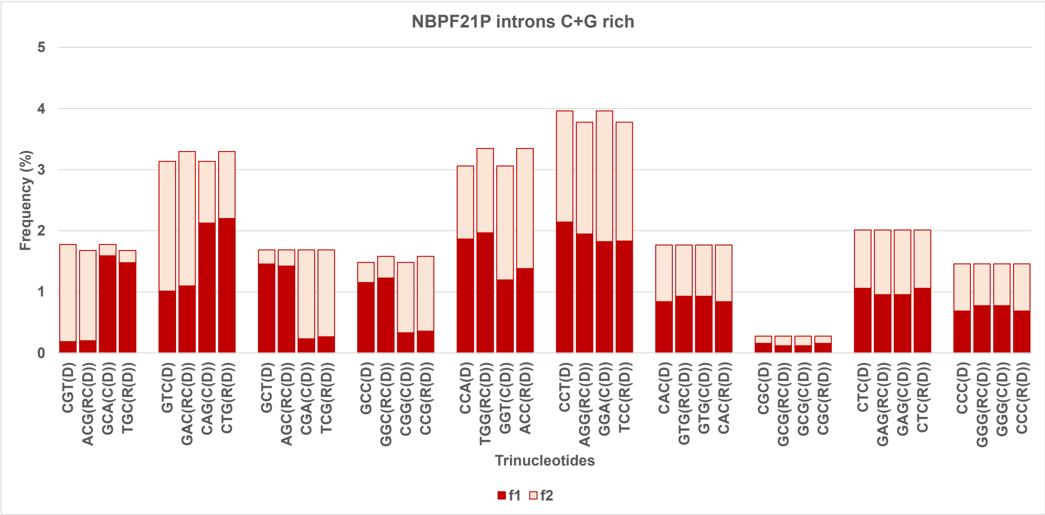

NBPF22P sequence

						A-T rich						
**D**	**f**	**%**	**RC**	**f**	**%**	**C**	**f**	**%**	**R**	**f**	**%**	
**ATG**	313,00	2,06	CAT	301,00	1,98	TAC	143,00	0,94	GTA	145,00	0,95	Ia
**TGA**	340,00	2,24	TCA	340,00	2,24	ACT	265,00	1,74	AGT	241,00	1,59	
**TAG**	154,00	1,01	CTA	180,00	1,18	ATC	208,00	1,37	GAT	213,00	1,40	
**TAA**	209,00	1,37	TTA	219,00	1,44	ATT	307,00	2,02	AAT	276,00	1,82	Ib
**AAC**	206,00	1,36	GTT	203,00	1,34	TTG	284,00	1,87	CAA	246,00	1,62	
**AAG**	262,00	1,72	CTT	340,00	2,24	TTC	347,00	2,28	GAA	288,00	1,89	
**ACA**	270,00	1,78	TGT	364,00	2,39	TGT*			ACA*			Ic
**ATA**	197,00	1,30	TAT	235,00	1,55	TAT*			ATA*			
**AGA**	313,00	2,06	TCT	440,00	2,89	TCT*			AGA*			
**AAA**	385,00	2,53	TTT	460,00	3,03	TTT*			AAA*			

Quadruplet		f1	f2	fu
ATG	ATG(D)	2,06	0,94	3,00
	CAT(RC(D))	1,98	0,95	2,93
	TAC(C(D))	0,94	2,06	3,00
	GTA(R(D))	0,95	1,98	2,93
TGA	TGA(D)	2,24	1,74	3,98
	TCA(RC(D))	2,24	1,59	3,82
	ACT(C(D))	1,74	2,24	3,98
	AGT(R(D))	1,59	2,24	3,82
TAG	TAG(D)	1,01	1,37	2,38
	CTA(RC(D))	1,18	1,40	2,59
	ATC(C(D))	1,37	1,01	2,38
	GAT(R(D))	1,40	1,18	2,59
TAA	TAA(D)	1,37	2,02	3,39
	TTA(RC(D))	1,44	1,82	3,26
	ATT(C(D))	2,02	1,37	3,39
	AAT(R(D))	1,82	1,44	3,26
AAC	AAC(D)	1,36	1,87	3,22
	GTT(RC(D))	1,34	1,62	2,95
	TTG(C(D))	1,87	1,36	3,22
	CAA(R(D))	1,62	1,34	2,95
AAG	AAG(D)	1,72	2,28	4,01
	CTT(RC(D))	2,24	1,89	4,13
	TTC(C(D))	2,28	1,72	4,01
	GAA(R(D))	1,89	2,24	4,13
ACA	ACA(D)	0,89	1,20	2,09
	TGT(RC(D))	1,20	0,89	2,09
	TGT(C(D))	1,20	0,89	2,09
	ACA(R(D))	0,89	1,20	2,09
ATA	ATA(D)	0,65	0,77	1,42
	TAT(RC(D))	0,77	0,65	1,42
	TAT(C(D))	0,77	0,65	1,42
	ATA(R(D))	0,65	0,65	1,30
AGA	AGA(D)	1,03	1,45	2,48
	TCT(RC(D))	1,45	1,03	2,48
	TCT(C(D))	1,45	1,03	2,48
	AGA(R(D))	1,03	1,45	2,48
AAA	AAA(D)	1,27	1,51	2,78
	TTT(RC(D))	1,51	1,27	2,78
	TTT(C(D))	1,51	1,27	2,78
	AAA(R(D))	1,27	1,51	2,78

Composition of DNA sequence in NBPF22P

Base	No.of bases	%
A	3965	26,08%
C	3583	23,57%
G	3206	21,09%
T	4450	29,27%
N	0	0,00%
Sum	15204	100,00%

% of coding bases from NBPF22P (including N bases)

81,72%

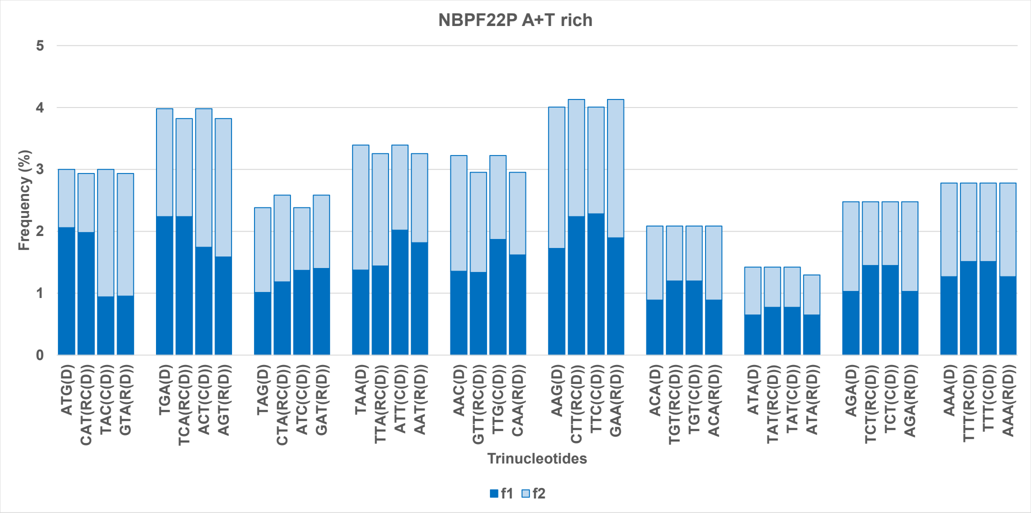

NBPF22P exons

A-T rich

D	f	%	RC	f	%	C	f	%	R	f	%	
**ATG**	27,00	1,71	CAT	18,00	1,14	TAC	17,00	1,08	GTA	8,00	0,51	Ia
**TGA**	37,00	2,34	TCA	44,00	2,79	ACT	33,00	2,09	AGT	26,00	1,65	
**TAG**	10,00	0,63	CTA	13,00	0,82	ATC	16,00	1,01	GAT	27,00	1,71	
**TAA**	10,00	0,63	TTA	11,00	0,70	ATT	19,00	1,20	AAT	21,00	1,33	Ib
**AAC**	18,00	1,14	GTT	20,00	1,27	TTG	33,00	2,09	CAA	43,00	2,72	
**AAG**	44,00	2,79	CTT	34,00	2,15	TTC	29,00	1,84	GAA	30,00	1,90	
**ACA**	22,00	1,39	TGT	21,00	1,33	TGT*			ACA*			Ic
**ATA**	12,00	0,76	TAT	7,00	0,44	TAT*			ATA*			
**AGA**	40,00	2,53	TCT	25,00	1,58	TCT*			AGA*			
**AAA**	36,00	2,28	TTT	25,00	1,58	TTT*			AAA*			

Quadruplet		f1	f2	fu
ATG	ATG(D)	1,71	1,08	2,79
	CAT(RC(D))	1,14	0,51	1,65
	TAC(C(D))	1,08	1,71	2,79
	GTA(R(D))	0,51	1,14	1,65
TGA	TGA(D)	2,34	2,09	4,44
	TCA(RC(D))	2,79	1,65	4,44
	ACT(C(D))	2,09	2,34	4,44
	AGT(R(D))	1,65	2,79	4,44
TAG	TAG(D)	0,63	1,01	1,65
	CTA(RC(D))	0,82	1,71	2,53
	ATC(C(D))	1,01	0,63	1,65
	GAT(R(D))	1,71	0,82	2,53
TAA	TAA(D)	0,63	1,20	1,84
	TTA(RC(D))	0,70	1,33	2,03
	ATT(C(D))	1,20	0,63	1,84
	AAT(R(D))	1,33	0,70	2,03
AAC	AAC(D)	1,14	2,09	3,23
	GTT(RC(D))	1,27	2,72	3,99
	TTG(C(D))	2,09	1,14	3,23
	CAA(R(D))	2,72	1,27	3,99
AAG	AAG(D)	2,79	1,84	4,63
	CTT(RC(D))	2,15	1,90	4,06
	TTC(C(D))	1,84	2,79	4,63
	GAA(R(D))	1,90	2,15	4,06
ACA	ACA(D)	0,70	0,67	1,36
	TGT(RC(D))	0,67	0,70	1,36
	TGT(C(D))	0,67	0,70	1,36
	ACA(R(D))	0,70	0,67	1,36
ATA	ATA(D)	0,38	0,22	0,60
	TAT(RC(D))	0,22	0,38	0,60
	TAT(C(D))	0,22	0,38	0,60
	ATA(R(D))	0,38	0,38	0,76
AGA	AGA(D)	1,27	0,79	2,06
	TCT(RC(D))	0,79	1,27	2,06
	TCT(C(D))	0,79	1,27	2,06
	AGA(R(D))	1,27	0,79	2,06
AAA	AAA(D)	1,14	0,79	1,93
	TTT(RC(D))	0,79	1,14	1,93
	TTT(C(D))	0,79	1,14	1,93
	AAA(R(D))	1,14	0,79	1,93

Composition of DNA sequence in NBPF22P exons

Base	No.of bases	%
A	420	26,58%
C	425	26,90%
G	364	23,04%
T	371	23,48%
N	0	0,00%
Sum	1580	100,00%

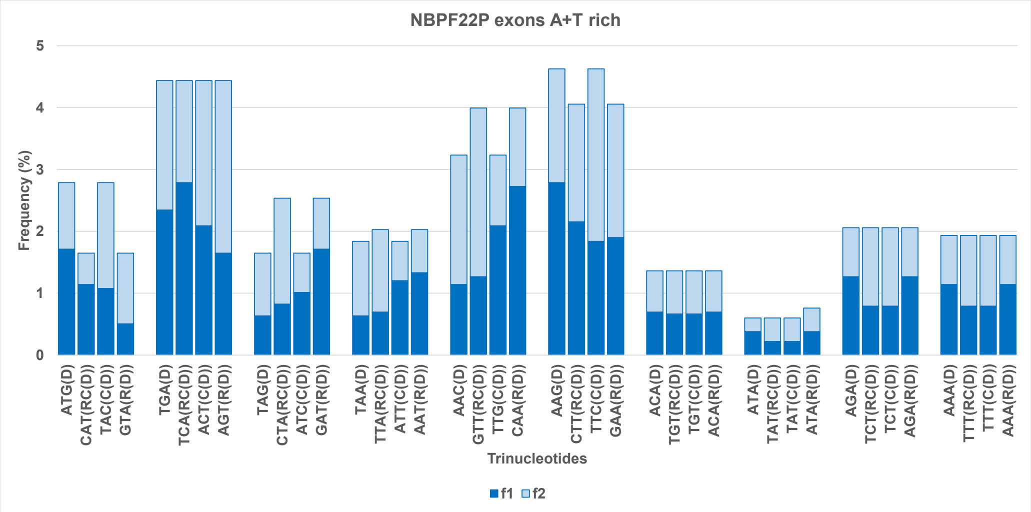

NBPF22P introns

A-T rich

D	f	%	RC	f	%	C	f	%	R	f	%	
**ATG**	255,00	2,05	CAT	253,00	2,04	TAC	116,00	0,93	GTA	124,00	1,00	Ia
**TGA**	269,00	2,17	TCA	269,00	2,17	ACT	216,00	1,74	AGT	196,00	1,58	
**TAG**	128,00	1,03	CTA	153,00	1,23	ATC	178,00	1,43	GAT	169,00	1,36	
**TAA**	182,00	1,47	TTA	193,00	1,55	ATT	264,00	2,13	AAT	231,00	1,86	Ib
**AAC**	165,00	1,33	GTT	171,00	1,38	TTG	231,00	1,86	CAA	179,00	1,44	
**AAG**	189,00	1,52	CTT	287,00	2,31	TTC	298,00	2,40	GAA	224,00	1,80	
**ACA**	215,00	1,73	TGT	318,00	2,56	TGT*			ACA*			Ic
**ATA**	168,00	1,35	TAT	212,00	1,71	TAT*			ATA*			
**AGA**	231,00	1,86	TCT	398,00	3,20	TCT*			AGA*			
**AAA**	300,00	2,42	TTT	403,00	3,24	TTT*			AAA*			

Quadruplet		f1	f2	fu
ATG	ATG(D)	2,05	0,93	2,99
	CAT(RC(D))	2,04	1,00	3,04
	TAC(C(D))	0,93	2,05	2,99
	GTA(R(D))	1,00	2,04	3,04
TGA	TGA(D)	2,17	1,74	3,90
	TCA(RC(D))	2,17	1,58	3,74
	ACT(C(D))	1,74	2,17	3,90
	AGT(R(D))	1,58	2,17	3,74
TAG	TAG(D)	1,03	1,43	2,46
	CTA(RC(D))	1,23	1,36	2,59
	ATC(C(D))	1,43	1,03	2,46
	GAT(R(D))	1,36	1,23	2,59
TAA	TAA(D)	1,47	2,13	3,59
	TTA(RC(D))	1,55	1,86	3,41
	ATT(C(D))	2,13	1,47	3,59
	AAT(R(D))	1,86	1,55	3,41
AAC	AAC(D)	1,33	1,86	3,19
	GTT(RC(D))	1,38	1,44	2,82
	TTG(C(D))	1,86	1,33	3,19
	CAA(R(D))	1,44	1,38	2,82
AAG	AAG(D)	1,52	2,40	3,92
	CTT(RC(D))	2,31	1,80	4,11
	TTC(C(D))	2,40	1,52	3,92
	GAA(R(D))	1,80	2,31	4,11
ACA	ACA(D)	0,87	1,28	2,15
	TGT(RC(D))	1,28	0,87	2,15
	TGT(C(D))	1,28	0,87	2,15
	ACA(R(D))	0,87	1,28	2,15
ATA	ATA(D)	0,68	0,85	1,53
	TAT(RC(D))	0,85	0,68	1,53
	TAT(C(D))	0,85	0,68	1,53
	ATA(R(D))	0,68	0,68	1,35
AGA	AGA(D)	0,93	1,60	2,53
	TCT(RC(D))	1,60	0,93	2,53
	TCT(C(D))	1,60	0,93	2,53
	AGA(R(D))	0,93	1,60	2,53
AAA	AAA(D)	1,21	1,62	2,83
	TTT(RC(D))	1,62	1,21	2,83
	TTT(C(D))	1,62	1,21	2,83
	AAA(R(D))	1,21	1,62	2,83

Composition of DNA sequence NBPF22P introns

Base	No.of bases	%
A	3158	25,42%
C	2904	23,37%
G	2584	20,80%
T	3778	30,41%
N	1	0,01%
Sum	12425	100,00%

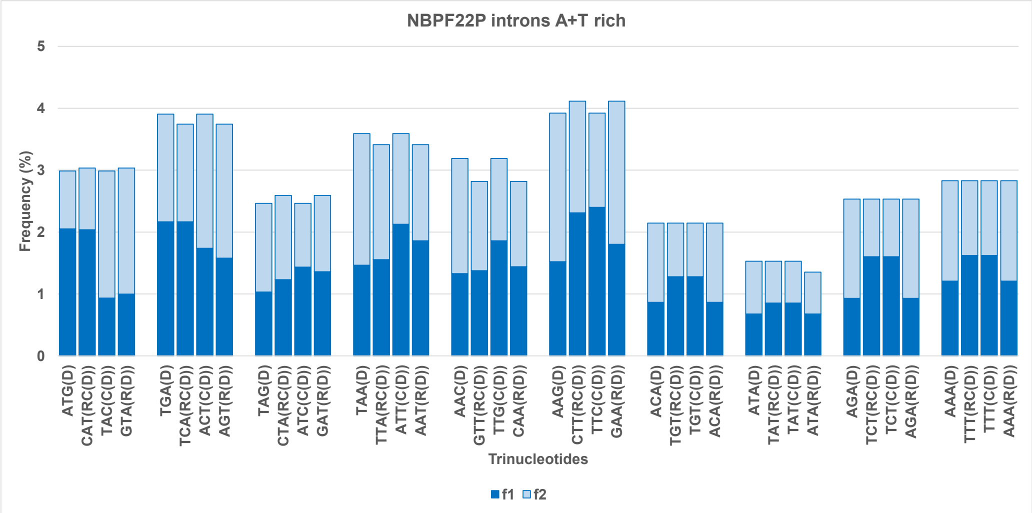

NBPF22P sequence

						C+G rich						
**D**	**f**	**%**	**RC**	**f**	**%**	**C**	**f**	**%**	**R**	**f**	**%**	
**CGT**	42,00	0,28	ACG	31,00	0,20	GCA	223,00	1,47	TGC	265,00	1,74	IIa
**GTC**	214,00	1,41	GAC	174,00	1,14	CAG	356,00	2,34	CTG	409,00	2,69	
**GCT**	209,00	1,37	AGC	213,00	1,40	CGA	28,00	0,18	TCG	25,00	0,16	
**GCC**	198,00	1,30	GGC	160,00	1,05	CGG	27,00	0,18	CCG	33,00	0,22	IIb
**CCA**	320,00	2,10	TGG	293,00	1,93	GGT	171,00	1,12	ACC	207,00	1,36	
**CCT**	383,00	2,52	AGG	271,00	1,78	GGA	260,00	1,71	TCC	332,00	2,18	
**CAC**	250,00	1,64	GTG	256,00	1,68	GTG*			CAC*			IIc
**CGC**	18,00	0,12	GCG	26,00	0,17	GCG*			CGC*			
**CTC**	368,00	2,42	GAG	266,00	1,75	GAG*			CTC*			
**CCC**	280,00	1,84	GGG	200,00	1,32	GGG*			CCC*			

Quadruplet		f1	f2	fu
CGT	CGT(D)	0,28	1,47	1,74
	ACG(RC(D))	0,20	1,74	1,95
	GCA(C(D))	1,47	0,28	1,74
	TGC(R(D))	1,74	0,20	1,95
GTC	GTC(D)	1,41	2,34	3,75
	GAC(RC(D))	1,14	2,69	3,84
	CAG(C(D))	2,34	1,41	3,75
	CTG(R(D))	2,69	1,14	3,84
GCT	GCT(D)	1,37	0,18	1,56
	AGC(RC(D))	1,40	0,16	1,57
	CGA(C(D))	0,18	1,37	1,56
	TCG(R(D))	0,16	1,40	1,57
GCC	GCC(D)	1,30	0,18	1,48
	GGC(RC(D))	1,05	0,22	1,27
	CGG(C(D))	0,18	1,30	1,48
	CCG(R(D))	0,22	1,05	1,27
CCA	CCA(D)	2,10	1,12	3,23
	TGG(RC(D))	1,93	1,36	3,29
	GGT(C(D))	1,12	2,10	3,23
	ACC(R(D))	1,36	1,93	3,29
CCT	CCT(D)	2,52	1,71	4,23
	AGG(RC(D))	1,78	2,18	3,97
	GGA(C(D))	1,71	2,52	4,23
	TCC(R(D))	2,18	1,78	3,97
CAC	CAC(D)	0,82	0,84	1,66
	GTG(RC(D))	0,84	0,82	1,66
	GTG(C(D))	0,84	0,82	1,66
	CAC(R(D))	0,82	0,84	1,66
CGC	CGC(D)	0,06	0,09	0,14
	GCG(RC(D))	0,09	0,06	0,14
	GCG(C(D))	0,09	0,06	0,14
	CGC(R(D))	0,06	0,09	0,14
CTC	CTC(D)	1,21	0,87	2,09
	GAG(RC(D))	0,87	1,21	2,09
	GAG(C(D))	0,87	1,21	2,09
	CTC(R(D))	1,21	0,87	2,09
CCC	CCC(D)	0,92	0,66	1,58
	GGG(RC(D))	0,66	0,92	1,58
	GGG(C(D))	0,66	0,92	1,58
	CCC(R(D))	0,92	0,66	1,58

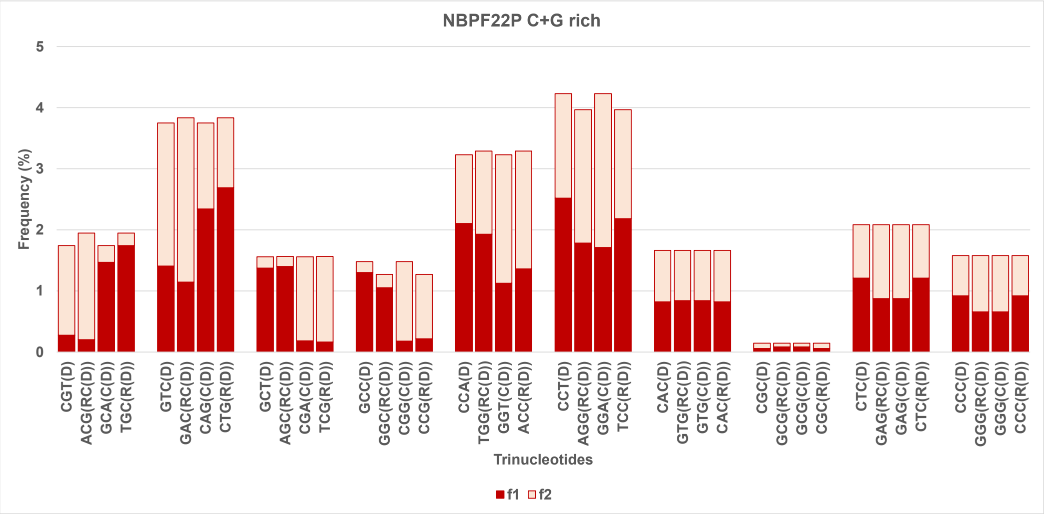

NBPF22P exons

C+G rich

D	f	%	RC	f	%	C	f	%	R	f	%		
**CGT**	6,00	0,38	ACG	4,00	0,25	GCA	32,00	2,03	TGC	31,00	1,96	IIa	86356
**GTC**	23,00	1,46	GAC	26,00	1,65	CAG	51,00	3,23	CTG	45,00	2,85		80741
**GCT**	35,00	2,22	AGC	41,00	2,60	CGA	5,00	0,32	TCG	3,00	0,19		44919
**GCC**	25,00	1,58	GGC	21,00	1,33	CGG	7,00	0,44	CCG	10,00	0,63	IIb	22459
**CCA**	42,00	2,66	TGG	30,00	1,90	GGT	13,00	0,82	ACC	30,00	1,90		45985
**CCT**	40,00	2,53	AGG	31,00	1,96	GGA	34,00	2,15	TCC	37,00	2,34		57215
**CAC**	28,00	1,77	GTG	15,00	0,95	GTG*			CAC*			IIc	
**CGC**	5,00	0,32	GCG	6,00	0,38	GCG*			CGC*				
**CTC**	41,00	2,60	GAG	33,00	2,09	GAG*			CTC*				
**CCC**	37,00	2,34	GGG	15,00	0,95	GGG*			CCC*				

Quadruplet		f1	f2	fu
CGT	CGT(D)	0,38	2,03	2,41
	ACG(RC(D))	0,25	1,96	2,22
	GCA(C(D))	2,03	0,38	2,41
	TGC(R(D))	1,96	0,25	2,22
GTC	GTC(D)	1,46	3,23	4,69
	GAC(RC(D))	1,65	2,85	4,50
	CAG(C(D))	3,23	1,46	4,69
	CTG(R(D))	2,85	1,65	4,50
GCT	GCT(D)	2,22	0,32	2,53
	AGC(RC(D))	2,60	0,19	2,79
	CGA(C(D))	0,32	2,22	2,53
	TCG(R(D))	0,19	2,60	2,79
GCC	GCC(D)	1,58	0,44	2,03
	GGC(RC(D))	1,33	0,63	1,96
	CGG(C(D))	0,44	1,58	2,03
	CCG(R(D))	0,63	1,33	1,96
CCA	CCA(D)	2,66	0,82	3,49
	TGG(RC(D))	1,90	1,90	3,80
	GGT(C(D))	0,82	2,66	3,49
	ACC(R(D))	1,90	1,90	3,80
CCT	CCT(D)	2,53	2,15	4,69
	AGG(RC(D))	1,96	2,34	4,31
	GGA(C(D))	2,15	2,53	4,69
	TCC(R(D))	2,34	1,96	4,31
CAC	CAC(D)	0,89	0,48	1,36
	GTG(RC(D))	0,48	0,89	1,36
	GTG(C(D))	0,48	0,89	1,36
	CAC(R(D))	0,89	0,48	1,36
CGC	CGC(D)	0,16	0,19	0,35
	GCG(RC(D))	0,19	0,16	0,35
	GCG(C(D))	0,19	0,16	0,35
	CGC(R(D))	0,16	0,19	0,35
CTC	CTC(D)	1,30	1,05	2,34
	GAG(RC(D))	1,05	1,30	2,34
	GAG(C(D))	1,05	1,30	2,34
	CTC(R(D))	1,30	1,05	2,34
CCC	CCC(D)	1,17	0,48	1,65
	GGG(RC(D))	0,48	1,17	1,65
	GGG(C(D))	0,48	1,17	1,65
	CCC(R(D))	1,17	0,48	1,65

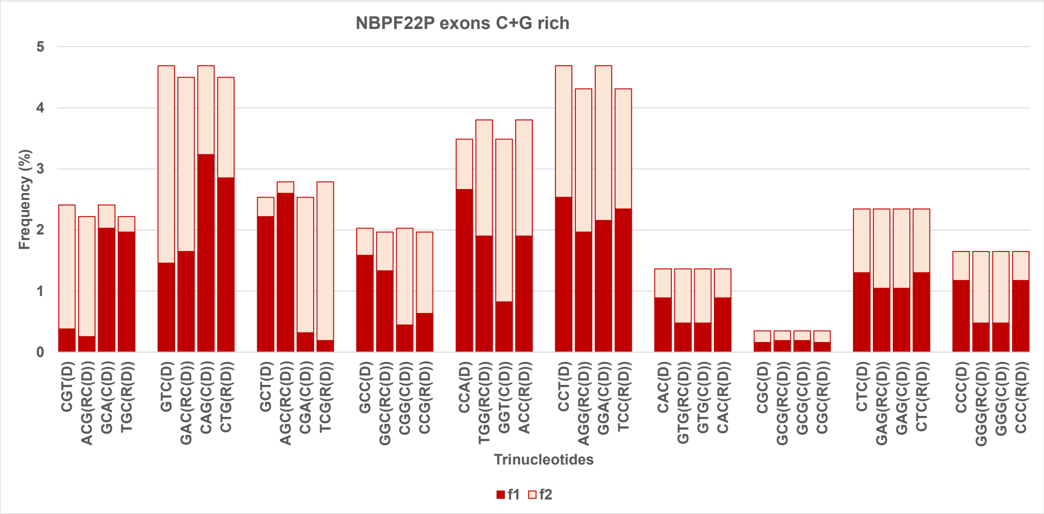

NBPF22P introns

C+G rich

D	f	%	RC	f	%	C	f	%	R	f	%	
**CGT**	32,00	0,26	ACG	25,00	0,20	GCA	171,00	1,38	TGC	215,00	1,73	IIa
**GTC**	173,00	1,39	GAC	127,00	1,02	CAG	270,00	2,17	CTG	337,00	2,71	
**GCT**	160,00	1,29	AGC	148,00	1,19	CGA	22,00	0,18	TCG	20,00	0,16	
**GCC**	157,00	1,26	GGC	132,00	1,06	CGG	19,00	0,15	CCG	21,00	0,17	IIb
**CCA**	253,00	2,04	TGG	247,00	1,99	GGT	146,00	1,18	ACC	158,00	1,27	
**CCT**	320,00	2,58	AGG	217,00	1,75	GGA	205,00	1,65	TCC	279,00	2,25	
**CAC**	206,00	1,66	GTG	226,00	1,82	GTG*			CAC*			IIc
**CGC**	11,00	0,09	GCG	18,00	0,14	GCG*			CGC*			
**CTC**	317,00	2,55	GAG	207,00	1,67	GAG*			CTC*			
**CCC**	224,00	1,80	GGG	172,00	1,38	GGG*			CCC*			

Quadruplet		f1	f2	fu
CGT	CGT(D)	0,26	1,38	1,63
	ACG(RC(D))	0,20	1,73	1,93
	GCA(C(D))	1,38	0,26	1,63
	TGC(R(D))	1,73	0,20	1,93
GTC	GTC(D)	1,39	2,17	3,57
	GAC(RC(D))	1,02	2,71	3,74
	CAG(C(D))	2,17	1,39	3,57
	CTG(R(D))	2,71	1,02	3,74
GCT	GCT(D)	1,29	0,18	1,47
	AGC(RC(D))	1,19	0,16	1,35
	CGA(C(D))	0,18	1,29	1,47
	TCG(R(D))	0,16	1,19	1,35
GCC	GCC(D)	1,26	0,15	1,42
	GGC(RC(D))	1,06	0,17	1,23
	CGG(C(D))	0,15	1,26	1,42
	CCG(R(D))	0,17	1,06	1,23
CCA	CCA(D)	2,04	1,18	3,21
	TGG(RC(D))	1,99	1,27	3,26
	GGT(C(D))	1,18	2,04	3,21
	ACC(R(D))	1,27	1,99	3,26
CCT	CCT(D)	2,58	1,65	4,23
	AGG(RC(D))	1,75	2,25	3,99
	GGA(C(D))	1,65	2,58	4,23
	TCC(R(D))	2,25	1,75	3,99
CAC	CAC(D)	0,83	0,91	1,74
	GTG(RC(D))	0,91	0,83	1,74
	GTG(C(D))	0,91	0,83	1,74
	CAC(R(D))	0,83	0,91	1,74
CGC	CGC(D)	0,04	0,07	0,12
	GCG(RC(D))	0,07	0,04	0,12
	GCG(C(D))	0,07	0,04	0,12
	CGC(R(D))	0,04	0,07	0,12
CTC	CTC(D)	1,28	0,83	2,11
	GAG(RC(D))	0,83	1,28	2,11
	GAG(C(D))	0,83	1,28	2,11
	CTC(R(D))	1,28	0,83	2,11
CCC	CCC(D)	0,90	0,69	1,59
	GGG(RC(D))	0,69	0,90	1,59
	GGG(C(D))	0,69	0,90	1,59
	CCC(R(D))	0,90	0,69	1,59

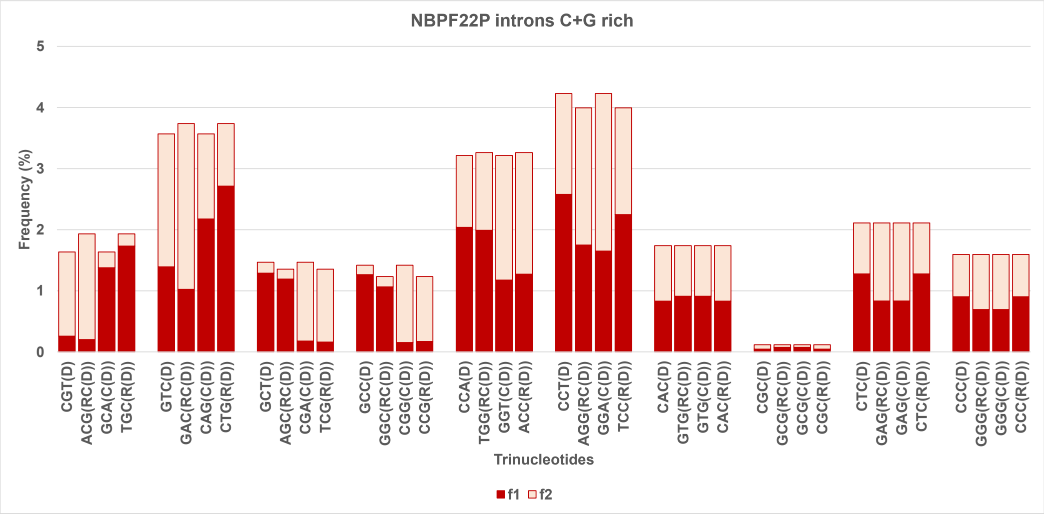

A-T rich												
**D**	**f**	**%**	**RC**	**f**	**%**	**C**	**f**	**%**	**R**	**f**	**%**	
**ATG**	728,00	1,98	CAT	721,00	1,96	TAC	368,00	1,00	GTA	370,00	1,01	Ia
**TGA**	857,00	2,33	TCA	798,00	2,17	ACT	609,00	1,66	AGT	621,00	1,69	
**TAG**	446,00	1,21	CTA	453,00	1,23	ATC	510,00	1,39	GAT	513,00	1,40	
**TAA**	579,00	1,58	TTA	627,00	1,71	ATT	847,00	2,31	AAT	794,00	2,16	Ib
**AAC**	493,00	1,34	GTT	589,00	1,60	TTG	799,00	2,18	CAA	612,00	1,67	
**AAG**	655,00	1,78	CTT	779,00	2,12	TTC	789,00	2,15	GAA	751,00	2,05	
**ACA**	659,00	1,80	TGT	926,00	2,52	TGT*			ACA*			Ic
**ATA**	620,00	1,69	TAT	677,00	1,84	TAT*			ATA*			
**AGA**	741,00	2,02	TCT	1002,00	2,73	TCT*			AGA*			
**AAA**	1161,00	3,16	TTT	1367,00	3,72	TTT*			AAA*			

Quadruplet		f1	f2	fu
ATG	ATG(D)	1,98	1,00	2,99
	CAT(RC(D))	1,96	1,01	2,97
	TAC(C(D))	1,00	1,98	2,99
	GTA(R(D))	1,01	1,96	2,97
TGA	TGA(D)	2,33	1,66	3,99
	TCA(RC(D))	2,17	1,69	3,87
	ACT(C(D))	1,66	2,33	3,99
	AGT(R(D))	1,69	2,17	3,87
TAG	TAG(D)	1,21	1,39	2,60
	CTA(RC(D))	1,23	1,40	2,63
	ATC(C(D))	1,39	1,21	2,60
	GAT(R(D))	1,40	1,23	2,63
TAA	TAA(D)	1,58	2,31	3,88
	TTA(RC(D))	1,71	2,16	3,87
	ATT(C(D))	2,31	1,58	3,88
	AAT(R(D))	2,16	1,71	3,87
AAC	AAC(D)	1,34	2,18	3,52
	GTT(RC(D))	1,60	1,67	3,27
	TTG(C(D))	2,18	1,34	3,52
	CAA(R(D))	1,67	1,60	3,27
AAG	AAG(D)	1,78	2,15	3,93
	CTT(RC(D))	2,12	2,05	4,17
	TTC(C(D))	2,15	1,78	3,93
	GAA(R(D))	2,05	2,12	4,17
ACA	ACA(D)	0,90	1,26	2,16
	TGT(RC(D))	1,26	0,90	2,16
	TGT(C(D))	1,26	0,90	2,16
	ACA(R(D))	0,90	1,26	2,16
ATA	ATA(D)	0,84	0,92	1,77
	TAT(RC(D))	0,92	0,84	1,77
	TAT(C(D))	0,92	0,84	1,77
	ATA(R(D))	0,84	0,84	1,69
AGA	AGA(D)	1,01	1,36	2,37
	TCT(RC(D))	1,36	1,01	2,37
	TCT(C(D))	1,36	1,01	2,37
	AGA(R(D))	1,01	1,36	2,37
AAA	AAA(D)	1,58	1,86	3,44
	TTT(RC(D))	1,86	1,58	3,44
	TTT(C(D))	1,86	1,58	3,44
	AAA(R(D))	1,58	1,86	3,44

Composition of DNA sequence in NBPF25P

Base	No.of bases	%
A	10095	27,50%
C	7592	20,68%
G	7867	21,43%
T	11160	30,40%
N	0	0,00%
Sum	36714	100,00%

% of coding bases from NBPF25P (including N bases)

**86,31%**

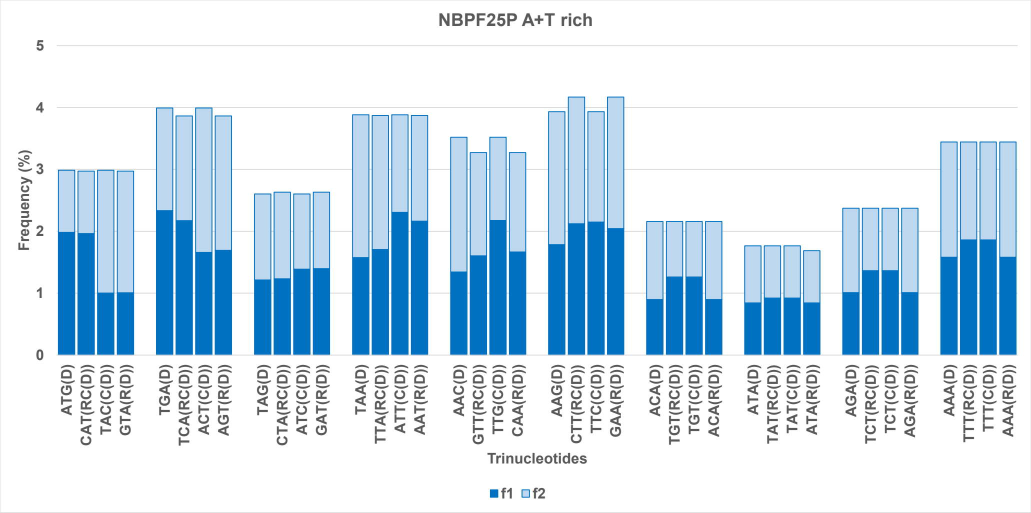

NBPF25P exons

A-T rich

D	f	%	RC	f	%	C	f	%	R	f	%	
**ATG**	75,00	1,96	CAT	69,00	1,80	TAC	39,00	1,02	GTA	23,00	0,60	Ia
**TGA**	89,00	2,33	TCA	97,00	2,54	ACT	62,00	1,62	AGT	60,00	1,57	
**TAG**	36,00	0,94	CTA	33,00	0,86	ATC	43,00	1,12	GAT	50,00	1,31	
**TAA**	33,00	0,86	TTA	60,00	1,57	ATT	73,00	1,91	AAT	64,00	1,67	Ib
**AAC**	55,00	1,44	GTT	58,00	1,52	TTG	75,00	1,96	CAA	72,00	1,88	
**AAG**	116,00	3,03	CTT	62,00	1,62	TTC	58,00	1,52	GAA	129,00	3,37	
**ACA**	73,00	1,91	TGT	69,00	1,80	TGT*			ACA*			Ic
**ATA**	39,00	1,02	TAT	47,00	1,23	TAT*			ATA*			
**AGA**	113,00	2,95	TCT	64,00	1,67	TCT*			AGA*			
**AAA**	95,00	2,48	TTT	85,00	2,22	TTT*			AAA*			

Quadruplet		f1	f2	fu
ATG	ATG(D)	1,96	1,02	2,98
	CAT(RC(D))	1,80	0,60	2,41
	TAC(C(D))	1,02	1,96	2,98
	GTA(R(D))	0,60	1,80	2,41
TGA	TGA(D)	2,33	1,62	3,95
	TCA(RC(D))	2,54	1,57	4,10
	ACT(C(D))	1,62	2,33	3,95
	AGT(R(D))	1,57	2,54	4,10
TAG	TAG(D)	0,94	1,12	2,07
	CTA(RC(D))	0,86	1,31	2,17
	ATC(C(D))	1,12	0,94	2,07
	GAT(R(D))	1,31	0,86	2,17
TAA	TAA(D)	0,86	1,91	2,77
	TTA(RC(D))	1,57	1,67	3,24
	ATT(C(D))	1,91	0,86	2,77
	AAT(R(D))	1,67	1,57	3,24
AAC	AAC(D)	1,44	1,96	3,40
	GTT(RC(D))	1,52	1,88	3,40
	TTG(C(D))	1,96	1,44	3,40
	CAA(R(D))	1,88	1,52	3,40
AAG	AAG(D)	3,03	1,52	4,55
	CTT(RC(D))	1,62	3,37	4,99
	TTC(C(D))	1,52	3,03	4,55
	GAA(R(D))	3,37	1,62	4,99
ACA	ACA(D)	0,95	0,90	1,86
	TGT(RC(D))	0,90	0,95	1,86
	TGT(C(D))	0,90	0,95	1,86
	ACA(R(D))	0,95	0,90	1,86
ATA	ATA(D)	0,51	0,61	1,12
	TAT(RC(D))	0,61	0,51	1,12
	TAT(C(D))	0,61	0,51	1,12
	ATA(R(D))	0,51	0,51	1,02
AGA	AGA(D)	1,48	0,84	2,31
	TCT(RC(D))	0,84	1,48	2,31
	TCT(C(D))	0,84	1,48	2,31
	AGA(R(D))	1,48	0,84	2,31
AAA	AAA(D)	1,24	1,11	2,35
	TTT(RC(D))	1,11	1,24	2,35
	TTT(C(D))	1,11	1,24	2,35
	AAA(R(D))	1,24	1,11	2,35

Composition of DNA sequence in NBPF25P exons

Base	No.of bases	%
A	1102	28,80%
C	829	21,66%
G	937	24,48%
T	959	25,06%
N	0	0,00%
Sum	3827	100,00%

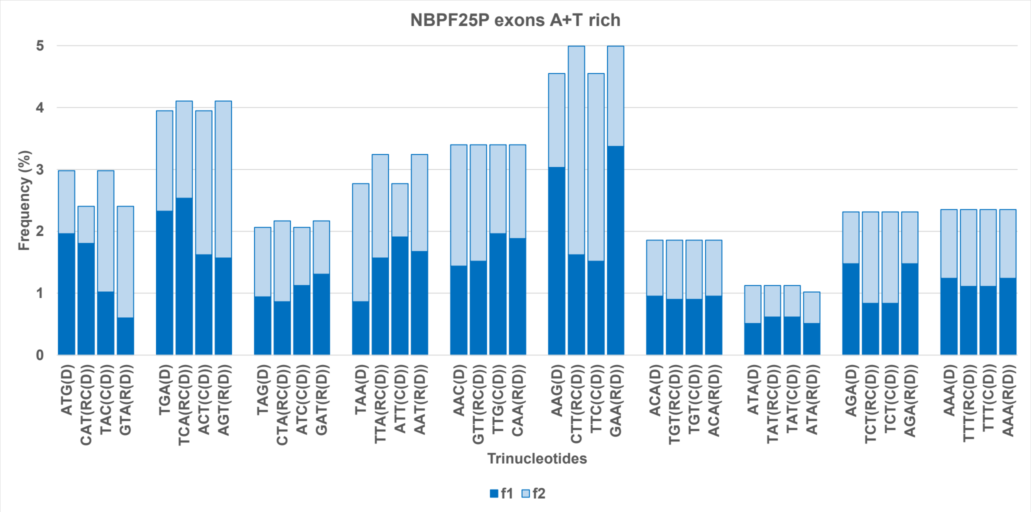

NBPF25 introns

A-T rich

D	f	%	RC	f	%	C	f	%	R	f	%	
**ATG**	633,00	2,00	CAT	628,00	1,98	TAC	317,00	1,00	GTA	336,00	1,06	Ia
**TGA**	749,00	2,36	TCA	679,00	2,14	ACT	534,00	1,69	AGT	543,00	1,71	
**TAG**	396,00	1,25	CTA	406,00	1,28	ATC	454,00	1,43	GAT	454,00	1,43	
**TAA**	529,00	1,67	TTA	548,00	1,73	ATT	754,00	2,38	AAT	711,00	2,24	Ib
**AAC**	428,00	1,35	GTT	517,00	1,63	TTG	709,00	2,24	CAA	531,00	1,68	
**AAG**	530,00	1,67	CTT	696,00	2,20	TTC	710,00	2,24	GAA	609,00	1,92	
**ACA**	565,00	1,78	TGT	818,00	2,58	TGT*			ACA*			Ic
**ATA**	565,00	1,78	TAT	613,00	1,93	TAT*			ATA*			
**AGA**	620,00	1,96	TCT	913,00	2,88	TCT*			AGA*			
**AAA**	1049,00	3,31	TTT	1244,00	3,93	TTT*			AAA*			

Quadruplet		f1	f2	fu
ATG	ATG(D)	2,00	1,00	3,00
	CAT(RC(D))	1,98	1,06	3,04
	TAC(C(D))	1,00	2,00	3,00
	GTA(R(D))	1,06	1,98	3,04
TGA	TGA(D)	2,36	1,69	4,05
	TCA(RC(D))	2,14	1,71	3,86
	ACT(C(D))	1,69	2,36	4,05
	AGT(R(D))	1,71	2,14	3,86
TAG	TAG(D)	1,25	1,43	2,68
	CTA(RC(D))	1,28	1,43	2,71
	ATC(C(D))	1,43	1,25	2,68
	GAT(R(D))	1,43	1,28	2,71
TAA	TAA(D)	1,67	2,38	4,05
	TTA(RC(D))	1,73	2,24	3,97
	ATT(C(D))	2,38	1,67	4,05
	AAT(R(D))	2,24	1,73	3,97
AAC	AAC(D)	1,35	2,24	3,59
	GTT(RC(D))	1,63	1,68	3,31
	TTG(C(D))	2,24	1,35	3,59
	CAA(R(D))	1,68	1,63	3,31
AAG	AAG(D)	1,67	2,24	3,91
	CTT(RC(D))	2,20	1,92	4,12
	TTC(C(D))	2,24	1,67	3,91
	GAA(R(D))	1,92	2,20	4,12
ACA	ACA(D)	0,89	1,29	2,18
	TGT(RC(D))	1,29	0,89	2,18
	TGT(C(D))	1,29	0,89	2,18
	ACA(R(D))	0,89	1,29	2,18
ATA	ATA(D)	0,89	0,97	1,86
	TAT(RC(D))	0,97	0,89	1,86
	TAT(C(D))	0,97	0,89	1,86
	ATA(R(D))	0,89	0,89	1,78
AGA	AGA(D)	0,98	1,44	2,42
	TCT(RC(D))	1,44	0,98	2,42
	TCT(C(D))	1,44	0,98	2,42
	AGA(R(D))	0,98	1,44	2,42
AAA	AAA(D)	1,66	1,96	3,62
	TTT(RC(D))	1,96	1,66	3,62
	TTT(C(D))	1,96	1,66	3,62
	AAA(R(D))	1,66	1,96	3,62

Composition of DNA sequence NBPF25 introns

Base	No.of bases	%
A	8738	27,58%
C	6466	20,41%
G	6604	20,84%
T	9879	31,18%
N	0	0,00%
Sum	31687	100,00%

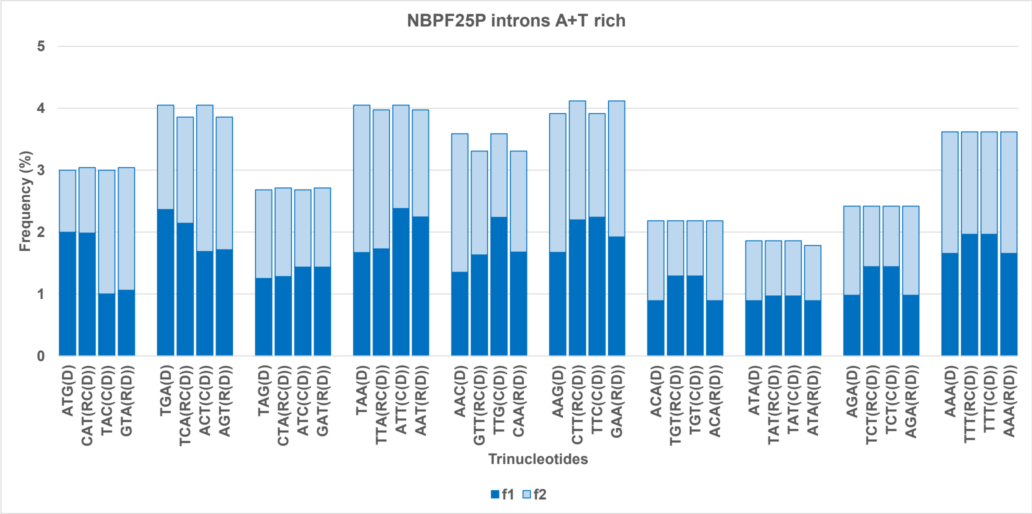

NBPF25P sequence

						C+G rich						
**D**	**f**	**%**	**RC**	**f**	**%**	**C**	**f**	**%**	**R**	**f**	**%**	
**CGT**	83,00	0,23	ACG	88,00	0,24	GCA	525,00	1,43	TGC	510,00	1,39	IIa
**GTC**	455,00	1,24	GAC	400,00	1,09	CAG	755,00	2,06	CTG	851,00	2,32	
**GCT**	511,00	1,39	AGC	465,00	1,27	CGA	92,00	0,25	TCG	79,00	0,22	
**GCC**	391,00	1,07	GGC	439,00	1,20	CGG	83,00	0,23	CCG	104,00	0,28	IIb
**CCA**	616,00	1,68	TGG	737,00	2,01	GGT	436,00	1,19	ACC	415,00	1,13	
**CCT**	685,00	1,87	AGG	688,00	1,87	GGA	634,00	1,73	TCC	599,00	1,63	
**CAC**	510,00	1,39	GTG	652,00	1,78	GTG*			CAC*			IIc
**CGC**	86,00	0,23	GCG	73,00	0,20	GCG*			CGC*			
**CTC**	724,00	1,97	GAG	659,00	1,80	GAG*			CTC*			
**CCC**	438,00	1,19	GGG	468,00	1,27	GGG*			CCC*			

Quadruplet		f1	f2	fu
	CGT(D)	0,23	1,43	1,66
CGT	ACG(RC(D))	0,24	1,39	1,63
	GCA(C(D))	1,43	0,23	1,66
	TGC(R(D))	1,39	0,24	1,63
	GTC(D)	1,24	2,06	3,30
GTC	GAC(RC(D))	1,09	2,32	3,41
	CAG(C(D))	2,06	1,24	3,30
	CTG(R(D))	2,32	1,09	3,41
	GCT(D)	1,39	0,25	1,64
GCT	AGC(RC(D))	1,27	0,22	1,48
	CGA(C(D))	0,25	1,39	1,64
	TCG(R(D))	0,22	1,27	1,48
	GCC(D)	1,07	0,23	1,29
GCC	GGC(RC(D)	1,20	0,28	1,48
	CGG(C(D))	0,23	1,07	1,29
	CCG(R(D))	0,28	1,20	1,48
	CCA(D)	1,68	1,19	2,87
CCA	TGG(RC(D))	2,01	1,13	3,14
	GGT(C(D))	1,19	1,68	2,87
	ACC(R(D))	1,13	2,01	3,14
	CCT(D)	1,87	1,73	3,59
CCT	AGG(RC(D))	1,87	1,63	3,51
	GGA(C(D))	1,73	1,87	3,59
	TCC(R(D))	1,63	1,87	3,51
	CAC(D)	0,69	0,89	1,58
CAC	GTG(RC(D))	0,89	0,69	1,58
	GTG(C(D))	0,89	0,69	1,58
	CAC(R(D))	0,69	0,89	1,58
	CGC(D)	0,12	0,10	0,22
CGC	GCG(RC(D)	0,10	0,12	0,22
	GCG(C(D))	0,10	0,12	0,22
	CGC(R(D))	0,12	0,10	0,22
	CTC(D)	0,99	0,90	1,88
CTC	GAG(RC(D))	0,90	0,99	1,88
	GAG(C(D))	0,90	0,99	1,88
	CTC(R(D))	0,99	0,90	1,88
	CCC(D)	0,60	0,64	1,23
CCC	GGG(RC(D)	0,64	0,60	1,23
	GGG(C(D))	0,64	0,60	1,23
	CCC(R(D))	0,60	0,64	1,23

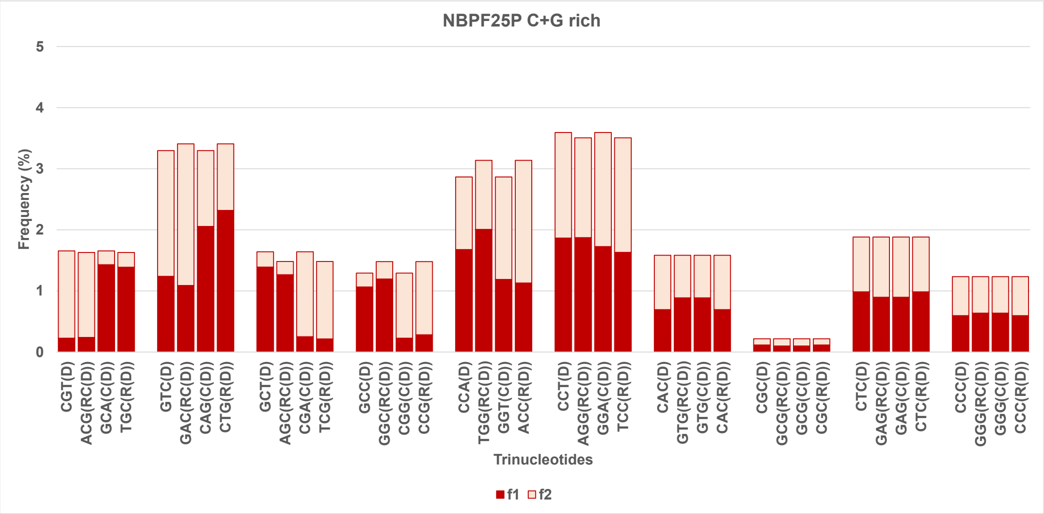

NBPF25P exons

C+G rich

D	f	%	RC	f	%	C	f	%	R	f	%		
**CGT**	18,00	0,47	ACG	18,00	0,47	GCA	59,00	1,54	TGC	59,00	1,54	IIa	86356
**GTC**	53,00	1,39	GAC	55,00	1,44	CAG	112,00	2,93	CTG	95,00	2,48		80741
**GCT**	66,00	1,73	AGC	75,00	1,96	CGA	14,00	0,37	TCG	8,00	0,21		44919
**GCC**	58,00	1,52	GGC	52,00	1,36	CGG	13,00	0,34	CCG	16,00	0,42	IIb	22459
**CCA**	69,00	1,80	TGG	78,00	2,04	GGT	37,00	0,97	ACC	41,00	1,07		45985
**CCT**	75,00	1,96	AGG	100,00	2,61	GGA	102,00	2,67	TCC	62,00	1,62		57215
**CAC**	45,00	1,18	GTG	50,00	1,31	GTG*			CAC*			IIc	
**CGC**	7,00	0,18	GCG	10,00	0,26	GCG*			CGC*				
**CTC**	77,00	2,01	GAG	84,00	2,20	GAG*			CTC*				
**CCC**	50,00	1,31	GGG	51,00	1,33	GGG*			CCC*				

Quadruplet		f1	f2	fu
	CGT(D)	0,47	1,54	2,01
CGT	ACG(RC(D))	0,47	1,54	2,01
	GCA(C(D))	1,54	0,47	2,01
	TGC(R(D))	1,54	0,47	2,01
	GTC(D)	1,39	2,93	4,31
GTC	GAC(RC(D))	1,44	2,48	3,92
	CAG(C(D))	2,93	1,39	4,31
	CTG(R(D))	2,48	1,44	3,92
	GCT(D)	1,73	0,37	2,09
GCT	AGC(RC(D))	1,96	0,21	2,17
	CGA(C(D))	0,37	1,73	2,09
	TCG(R(D))	0,21	1,96	2,17
	GCC(D)	1,52	0,34	1,86
GCC	GGC(RC(D))	1,36	0,42	1,78
	CGG(C(D))	0,34	1,52	1,86
	CCG(R(D))	0,42	1,36	1,78
	CCA(D)	1,80	0,97	2,77
CCA	TGG(RC(D))	2,04	1,07	3,11
	GGT(C(D))	0,97	1,80	2,77
	ACC(R(D))	1,07	2,04	3,11
	CCT(D)	1,96	2,67	4,63
CCT	AGG(RC(D))	2,61	1,62	4,24
	GGA(C(D))	2,67	1,96	4,63
	TCC(R(D))	1,62	2,61	4,24
	CAC(D)	0,59	0,65	1,24
CAC	GTG(RC(D))	0,65	0,59	1,24
	GTG(C(D))	0,65	0,59	1,24
	CAC(R(D))	0,59	0,65	1,24
	CGC(D)	0,09	0,13	0,22
CGC	GCG(RC(D))	0,13	0,09	0,22
	GCG(C(D))	0,13	0,09	0,22
	CGC(R(D))	0,09	0,13	0,22
	CTC(D)	1,01	1,10	2,10
CTC	GAG(RC(D))	1,10	1,01	2,10
	GAG(C(D))	1,10	1,01	2,10
	CTC(R(D))	1,01	1,10	2,10
	CCC(D)	0,65	0,67	1,32
CCC	GGG(RC(D))	0,67	0,65	1,32
	GGG(C(D))	0,67	0,65	1,32
	CCC(R(D))	0,65	0,67	1,32

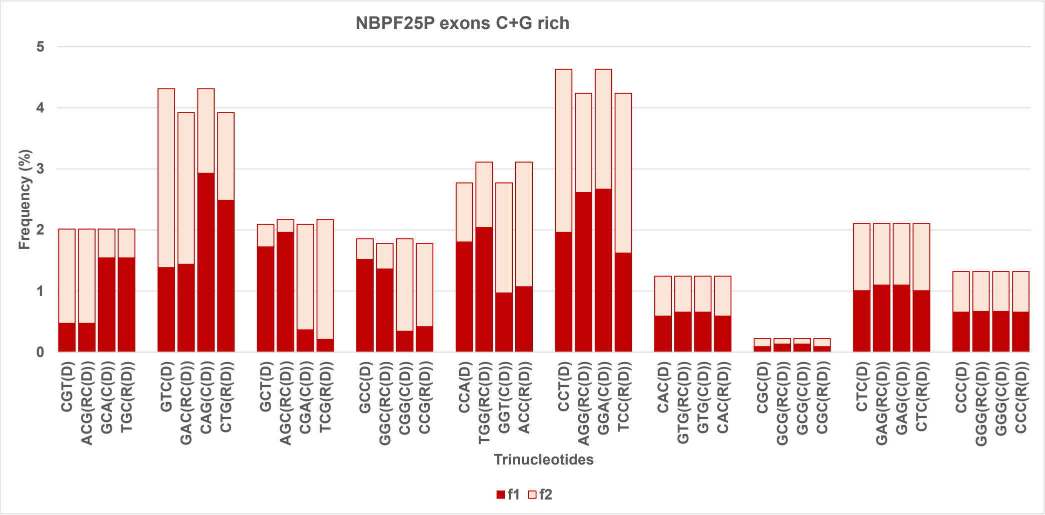

NBPF25 introns

C+G rich

D	f	%	RC	f	%	C	f	%	R	f	%	
**CGT**	60,00	0,19	ACG	58,00	0,18	GCA	451,00	1,42	TGC	436,00	1,38	IIa
**GTC**	384,00	1,21	GAC	328,00	1,04	CAG	618,00	1,95	CTG	737,00	2,33	
**GCT**	425,00	1,34	AGC	369,00	1,16	CGA	71,00	0,22	TCG	61,00	0,19	
**GCC**	308,00	0,97	GGC	363,00	1,15	CGG	41,00	0,13	CCG	69,00	0,22	IIb
**CCA**	526,00	1,66	TGG	640,00	2,02	GGT	379,00	1,20	ACC	359,00	1,13	
**CCT**	589,00	1,86	AGG	565,00	1,78	GGA	504,00	1,59	TCC	517,00	1,63	
**CAC**	444,00	1,40	GTG	564,00	1,78	GTG*			CAC*			IIc
**CGC**	61,00	0,19	GCG	45,00	0,14	GCG*			CGC*			
**CTC**	622,00	1,96	GAG	553,00	1,75	GAG*			CTC*			
**CCC**	366,00	1,16	GGG	384,00	1,21	GGG*			CCC*			

Quadruplet		f1	f2	fu
CGT	CGT(D)	0,19	1,42	1,61
	ACG(RC(D))	0,18	1,38	1,56
	GCA(C(D))	1,42	0,19	1,61
	TGC(R(D))	1,38	0,18	1,56
GTC	GTC(D)	1,21	1,95	3,16
	GAC(RC(D))	1,04	2,33	3,36
	CAG(C(D))	1,95	1,21	3,16
	CTG(R(D))	2,33	1,04	3,36
GCT	GCT(D)	1,34	0,22	1,57
	AGC(RC(D))	1,16	0,19	1,36
	CGA(C(D))	0,22	1,34	1,57
	TCG(R(D))	0,19	1,16	1,36
GCC	GCC(D)	0,97	0,13	1,10
	GGC(RC(D))	1,15	0,22	1,36
	CGG(C(D))	0,13	0,97	1,10
	CCG(R(D))	0,22	1,15	1,36
CCA	CCA(D)	1,66	1,20	2,86
	TGG(RC(D))	2,02	1,13	3,15
	GGT(C(D))	1,20	1,66	2,86
	ACC(R(D))	1,13	2,02	3,15
CCT	CCT(D)	1,86	1,59	3,45
	AGG(RC(D))	1,78	1,63	3,41
	GGA(C(D))	1,59	1,86	3,45
	TCC(R(D))	1,63	1,78	3,41
CAC	CAC(D)	0,70	0,89	1,59
	GTG(RC(D))	0,89	0,70	1,59
	GTG(C(D))	0,89	0,70	1,59
	CAC(R(D))	0,70	0,89	1,59
CGC	CGC(D)	0,10	0,07	0,17
	GCG(RC(D))	0,07	0,10	0,17
	GCG(C(D))	0,07	0,10	0,17
	CGC(R(D))	0,10	0,07	0,17
CTC	CTC(D)	0,98	0,87	1,85
	GAG(RC(D))	0,87	0,98	1,85
	GAG(C(D))	0,87	0,98	1,85
	CTC(R(D))	0,98	0,87	1,85
CCC	CCC(D)	0,58	0,61	1,18
	GGG(RC(D))	0,61	0,58	1,18
	GGG(C(D))	0,61	0,58	1,18
	CCC(R(D))	0,58	0,61	1,18

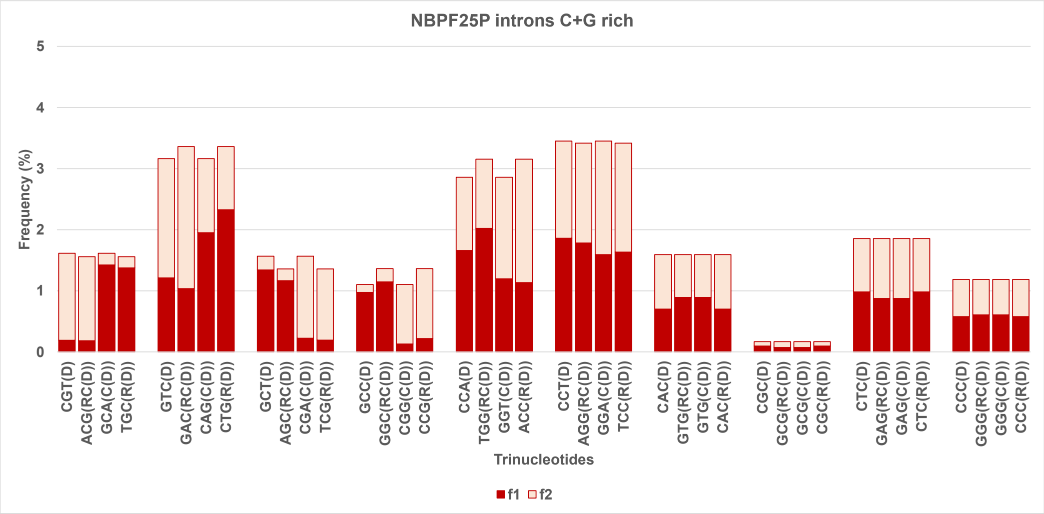

NBPF26 sequence

D	f	%	RC	f	%	C	f	%	R	f	%	
						A-T rich						
**ATG**	2294,00	1,93	CAT	2181,00	1,84	TAC	1323,00	1,11	GTA	1469,00	1,24	Ia
**TGA**	2577,00	2,17	TCA	2346,00	1,98	ACT	1965,00	1,65	AGT	2169,00	1,83	
**TAG**	1724,00	1,45	CTA	1482,00	1,25	ATC	1479,00	1,25	GAT	1752,00	1,48	
**TAA**	2173,00	1,83	TTA	2525,00	2,13	ATT	3016,00	2,54	AAT	2600,00	2,19	Ib
**AAC**	1505,00	1,27	GTT	2080,00	1,75	TTG	2638,00	2,22	CAA	1796,00	1,51	
**AAG**	2255,00	1,90	CTT	2748,00	2,31	TTC	2681,00	2,26	GAA	2391,00	2,01	
**ACA**	2032,00	1,71	TGT	3049,00	2,57	TGT*			ACA*			Ic
**ATA**	2110,00	1,78	TAT	2366,00	1,99	TAT*			ATA*			
**AGA**	2489,00	2,10	TCT	3074,00	2,59	TCT*			AGA*			
**AAA**	3458,00	2,91	TTT	5266,00	4,43	TTT*			AAA*			

Quadruplet		f1	f2	fu
ATG	ATG(D)	1,93	1,11	3,05
	CAT(RC(D))	1,84	1,24	3,07
	TAC(C(D))	1,11	1,93	3,05
	GTA(R(D))	1,24	1,84	3,07
TGA	TGA(D)	2,17	1,65	3,82
	TCA(RC(D))	1,98	1,83	3,80
	ACT(C(D))	1,65	2,17	3,82
	AGT(R(D))	1,83	1,98	3,80
TAG	TAG(D)	1,45	1,25	2,70
	CTA(RC(D))	1,25	1,48	2,72
	ATC(C(D))	1,25	1,45	2,70
	GAT(R(D))	1,48	1,25	2,72
TAA	TAA(D)	1,83	2,54	4,37
	TTA(RC(D))	2,13	2,19	4,32
	ATT(C(D))	2,54	1,83	4,37
	AAT(R(D))	2,19	2,13	4,32
AAC	AAC(D)	1,27	2,22	3,49
	GTT(RC(D))	1,75	1,51	3,26
	TTG(C(D))	2,22	1,27	3,49
	CAA(R(D))	1,51	1,75	3,26
AAG	AAG(D)	1,90	2,26	4,16
	CTT(RC(D))	2,31	2,01	4,33
	TTC(C(D))	2,26	1,90	4,16
	GAA(R(D))	2,01	2,31	4,33
ACA	ACA(D)	0,86	1,28	2,14
	TGT(RC(D))	1,28	0,86	2,14
	TGT(C(D))	1,28	0,86	2,14
	ACA(R(D))	0,86	1,28	2,14
ATA	ATA(D)	0,89	1,00	1,88
	TAT(RC(D))	1,00	0,89	1,88
	TAT(C(D))	1,00	0,89	1,88
	ATA(R(D))	0,89	0,89	1,78
AGA	AGA(D)	1,05	1,29	2,34
	TCT(RC(D))	1,29	1,05	2,34
	TCT(C(D))	1,29	1,05	2,34
	AGA(R(D))	1,05	1,29	2,34
AAA	AAA(D)	1,46	2,22	3,67
	TTT(RC(D))	2,22	1,46	3,67
	TTT(C(D))	2,22	1,46	3,67
	AAA(R(D))	1,46	2,22	3,67

Composition of DNA sequence in NBPF26

Base	No.of bases	%
A	32530	27,39%
C	23099	19,45%
G	25363	21,36%
T	37767	31,80%
N	0	0,00%
Sum	118759	100,00%

% of coding bases from NBPF26 (including N bases)

**94,37%**

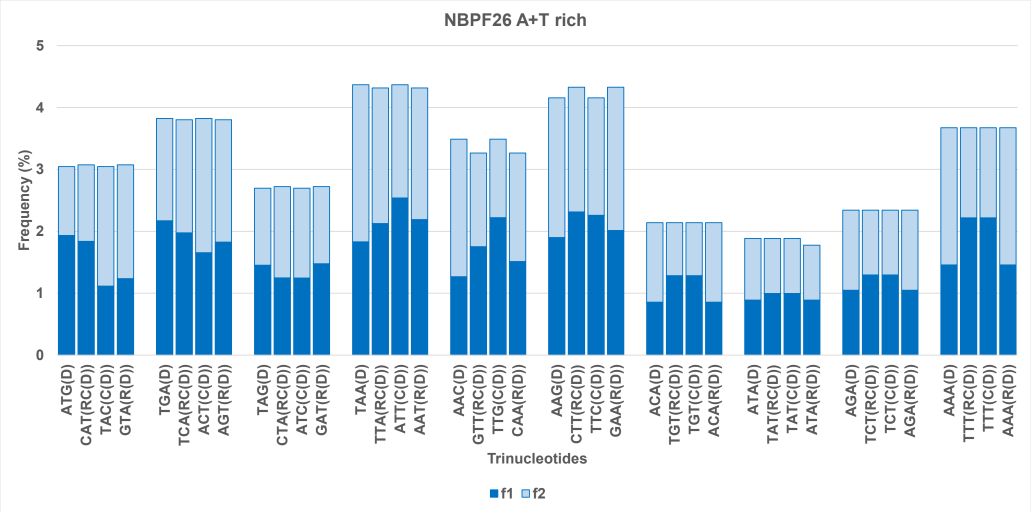

NBPF26 exons

A-T rich												
**D**	**f**	**%**	**RC**	**f**	**%**	**C**	**f**	**%**	**R**	**f**	**%**	
**ATG**	101,00	2,20	CAT	68,00	1,48	TAC	37,00	0,80	GTA	30,00	0,65	Ia
**TGA**	110,00	2,39	TCA	105,00	2,28	ACT	84,00	1,83	AGT	77,00	1,67	
**TAG**	25,00	0,54	CTA	20,00	0,43	ATC	35,00	0,76	GAT	52,00	1,13	
**TAA**	18,00	0,39	TTA	36,00	0,78	ATT	44,00	0,96	AAT	54,00	1,17	Ib
**AAC**	59,00	1,28	GTT	54,00	1,17	TTG	79,00	1,72	CAA	83,00	1,80	
**AAG**	155,00	3,37	CTT	71,00	1,54	TTC	54,00	1,17	GAA	166,00	3,61	
**ACA**	74,00	1,61	TGT	78,00	1,70	TGT*			ACA*			Ic
**ATA**	30,00	0,65	TAT	36,00	0,78	TAT*			ATA*			
**AGA**	151,00	3,28	TCT	58,00	1,26	TCT*			AGA*			
**AAA**	111,00	2,41	TTT	40,00	0,87	TTT*			AAA*			

Quadruplet		f1	f2	fu
ATG	ATG(D)	2,20	0,80	3,00
	CAT(RC(D))	1,48	0,65	2,13
	TAC(C(D))	0,80	2,20	3,00
	GTA(R(D))	0,65	1,48	2,13
TGA	TGA(D)	2,39	1,83	4,22
	TCA(RC(D))	2,28	1,67	3,96
	ACT(C(D))	1,83	2,39	4,22
	AGT(R(D))	1,67	2,28	3,96
TAG	TAG(D)	0,54	0,76	1,30
	CTA(RC(D))	0,43	1,13	1,57
	ATC(C(D))	0,76	0,54	1,30
	GAT(R(D))	1,13	0,43	1,57
TAA	TAA(D)	0,39	0,96	1,35
	TTA(RC(D))	0,78	1,17	1,96
	ATT(C(D))	0,96	0,39	1,35
	AAT(R(D))	1,17	0,78	1,96
AAC	AAC(D)	1,28	1,72	3,00
	GTT(RC(D))	1,17	1,80	2,98
	TTG(C(D))	1,72	1,28	3,00
	CAA(R(D))	1,80	1,17	2,98
AAG	AAG(D)	3,37	1,17	4,54
	CTT(RC(D))	1,54	3,61	5,15
	TTC(C(D))	1,17	3,37	4,54
	GAA(R(D))	3,61	1,54	5,15
ACA	ACA(D)	0,80	0,85	1,65
	TGT(RC(D))	0,85	0,80	1,65
	TGT(C(D))	0,85	0,80	1,65
	ACA(R(D))	0,80	0,85	1,65
ATA	ATA(D)	0,33	0,39	0,72
	TAT(RC(D))	0,39	0,33	0,72
	TAT(C(D))	0,39	0,33	0,72
	ATA(R(D))	0,33	0,33	0,65
AGA	AGA(D)	1,64	0,63	2,27
	TCT(RC(D))	0,63	1,64	2,27
	TCT(C(D))	0,63	1,64	2,27
	AGA(R(D))	1,64	0,63	2,27
AAA	AAA(D)	1,21	0,43	1,64
	TTT(RC(D))	0,43	1,21	1,64
	TTT(C(D))	0,43	1,21	1,64
	AAA(R(D))	1,21	0,43	1,64

Composition of DNA sequence in NBPF26 exons

Base	No.of bases	%
A	1289	28,01%
C	1080	23,47%
G	1286	27,94%
T	947	20,58%
N	0	0,00%
Sum	4602	100,00%

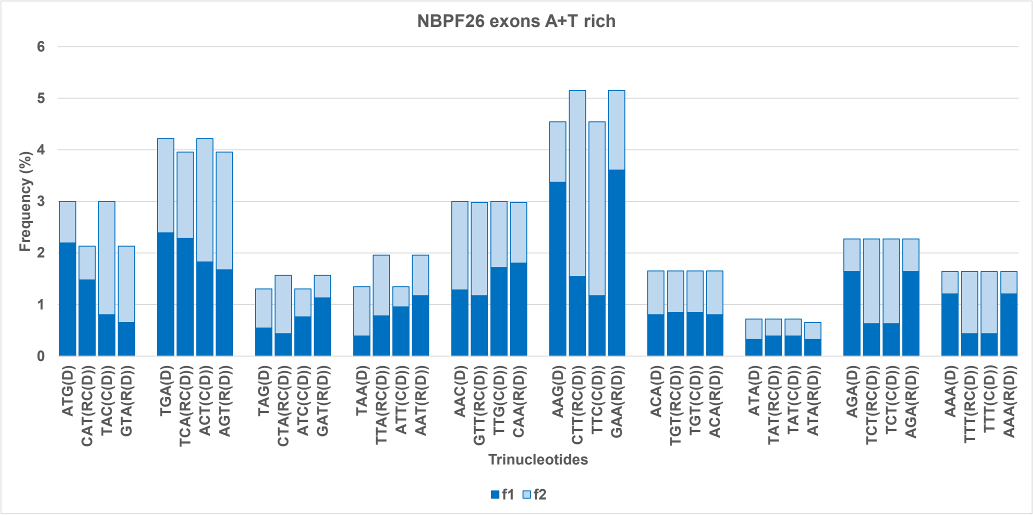

NBPF26 introns

A-T rich

D	f	%	RC	f	%	C	f	%	R	f	%	
**ATG**	2157,00	1,92	CAT	2075,00	1,85	TAC	1269,00	1,13	GTA	1421,00	1,27	Ia
**TGA**	2431,00	2,17	TCA	2193,00	1,96	ACT	1854,00	1,65	AGT	2052,00	1,83	
**TAG**	1673,00	1,49	CTA	1438,00	1,28	ATC	1416,00	1,26	GAT	1675,00	1,49	
**TAA**	2131,00	1,90	TTA	2456,00	2,19	ATT	2932,00	2,62	AAT	2513,00	2,24	Ib
**AAC**	1419,00	1,27	GTT	1993,00	1,78	TTG	2522,00	2,25	CAA	1679,00	1,50	
**AAG**	2066,00	1,84	CTT	2641,00	2,36	TTC	2588,00	2,31	GAA	2188,00	1,95	
**ACA**	1914,00	1,71	TGT	2925,00	2,61	TGT*			ACA*			Ic
**ATA**	2059,00	1,84	TAT	2301,00	2,05	TAT*			ATA*			
**AGA**	2297,00	2,05	TCT	2973,00	2,65	TCT*			AGA*			
**AAA**	3307,00	2,95	TTT	5165,00	4,61	TTT*			AAA*			

Quadruplet		f1	f2	fu
ATG	ATG(D)	1,92	1,13	3,06
	CAT(RC(D))	1,85	1,27	3,12
	TAC(C(D))	1,13	1,92	3,06
	GTA(R(D))	1,27	1,85	3,12
TGA	TGA(D)	2,17	1,65	3,82
	TCA(RC(D))	1,96	1,83	3,79
	ACT(C(D))	1,65	2,17	3,82
	AGT(R(D))	1,83	1,96	3,79
TAG	TAG(D)	1,49	1,26	2,76
	CTA(RC(D))	1,28	1,49	2,78
	ATC(C(D))	1,26	1,49	2,76
	GAT(R(D))	1,49	1,28	2,78
TAA	TAA(D)	1,90	2,62	4,52
	TTA(RC(D))	2,19	2,24	4,43
	ATT(C(D))	2,62	1,90	4,52
	AAT(R(D))	2,24	2,19	4,43
AAC	AAC(D)	1,27	2,25	3,52
	GTT(RC(D))	1,78	1,50	3,28
	TTG(C(D))	2,25	1,27	3,52
	CAA(R(D))	1,50	1,78	3,28
AAG	AAG(D)	1,84	2,31	4,15
	CTT(RC(D))	2,36	1,95	4,31
	TTC(C(D))	2,31	1,84	4,15
	GAA(R(D))	1,95	2,36	4,31
ACA	ACA(D)	0,85	1,31	2,16
	TGT(RC(D))	1,31	0,85	2,16
	TGT(C(D))	1,31	0,85	2,16
	ACA(R(D))	0,85	1,31	2,16
ATA	ATA(D)	0,92	1,03	1,95
	TAT(RC(D))	1,03	0,92	1,95
	TAT(C(D))	1,03	0,92	1,95
	ATA(R(D))	0,92	0,92	1,84
AGA	AGA(D)	1,02	1,33	2,35
	TCT(RC(D))	1,33	1,02	2,35
	TCT(C(D))	1,33	1,02	2,35
	AGA(R(D))	1,02	1,33	2,35
AAA	AAA(D)	1,48	2,30	3,78
	TTT(RC(D))	2,30	1,48	3,78
	TTT(C(D))	2,30	1,48	3,78
	AAA(R(D))	1,48	2,30	3,78

Composition of DNA sequence NBPF26 introns

Base	No.of bases	%
A	30716	27,41%
C	21511	19,19%
G	23582	21,04%
T	36260	32,36%
N	0	0,00%
Sum	112069	100,00%

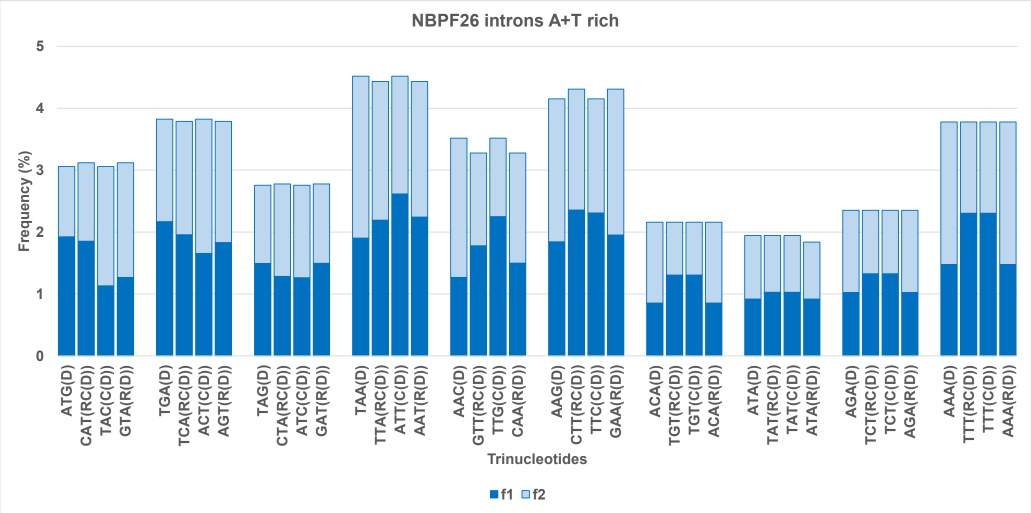

NBPF26 sequence

C+G rich												
**D**	**f**	**%**	**RC**	**f**	**%**	**C**	**f**	**%**	**R**	**f**	**%**	
**CGT**	252,00	0,21	ACG	237,00	0,20	GCA	1569,00	1,32	TGC	1635,00	1,38	IIa
**GTC**	1274,00	1,07	GAC	1159,00	0,98	CAG	2288,00	1,93	CTG	2633,00	2,22	
**GCT**	1641,00	1,38	AGC	1487,00	1,25	CGA	249,00	0,21	TCG	250,00	0,21	
**GCC**	1224,00	1,03	GGC	1329,00	1,12	CGG	243,00	0,20	CCG	240,00	0,20	IIb
**CCA**	1831,00	1,54	TGG	2395,00	2,02	GGT	1444,00	1,22	ACC	1266,00	1,07	
**CCT**	2163,00	1,82	AGG	2167,00	1,82	GGA	2032,00	1,71	TCC	1744,00	1,47	
**CAC**	1513,00	1,27	GTG	2091,00	1,76	GTG*			CAC*			IIc
**CGC**	240,00	0,20	GCG	257,00	0,22	GCG*			CGC*			
**CTC**	1980,00	1,67	GAG	2045,00	1,72	GAG*			CTC*			
**CCC**	1260,00	1,06	GGG	1606,00	1,35	GGG*			CCC*			

Quadruplet		f1	f2	fu
CGT	CGT(D)	0,21	1,32	1,53
	ACG(RC(D))	0,20	1,38	1,58
	GCA(C(D))	1,32	0,21	1,53
	TGC(R(D))	1,38	0,20	1,58
GTC	GTC(D)	1,07	1,93	3,00
	GAC(RC(D))	0,98	2,22	3,19
	CAG(C(D))	1,93	1,07	3,00
	CTG(R(D))	2,22	0,98	3,19
GCT	GCT(D)	1,38	0,21	1,59
	AGC(RC(D))	1,25	0,21	1,46
	CGA(C(D))	0,21	1,38	1,59
	TCG(R(D))	0,21	1,25	1,46
GCC	GCC(D)	1,03	0,20	1,24
	GGC(RC(D))	1,12	0,20	1,32
	CGG(C(D))	0,20	1,03	1,24
	CCG(R(D))	0,20	1,12	1,32
CCA	CCA(D)	1,54	1,22	2,76
	TGG(RC(D))	2,02	1,07	3,08
	GGT(C(D))	1,22	1,54	2,76
	ACC(R(D))	1,07	2,02	3,08
CCT	CCT(D)	1,82	1,71	3,53
	AGG(RC(D))	1,82	1,47	3,29
	GGA(C(D))	1,71	1,82	3,53
	TCC(R(D))	1,47	1,82	3,29
CAC	CAC(D)	0,64	0,88	1,52
	GTG(RC(D))	0,88	0,64	1,52
	GTG(C(D))	0,88	0,64	1,52
	CAC(R(D))	0,64	0,88	1,52
CGC	CGC(D)	0,10	0,11	0,21
	GCG(RC(D))	0,11	0,10	0,21
	GCG(C(D))	0,11	0,10	0,21
	CGC(R(D))	0,10	0,11	0,21
CTC	CTC(D)	0,83	0,86	1,69
	GAG(RC(D))	0,86	0,83	1,69
	GAG(C(D))	0,86	0,83	1,69
	CTC(R(D))	0,83	0,86	1,69
CCC	CCC(D)	0,53	0,68	1,21
	GGG(RC(D))	0,68	0,53	1,21
	GGG(C(D))	0,68	0,53	1,21
	CCC(R(D))	0,53	0,68	1,21

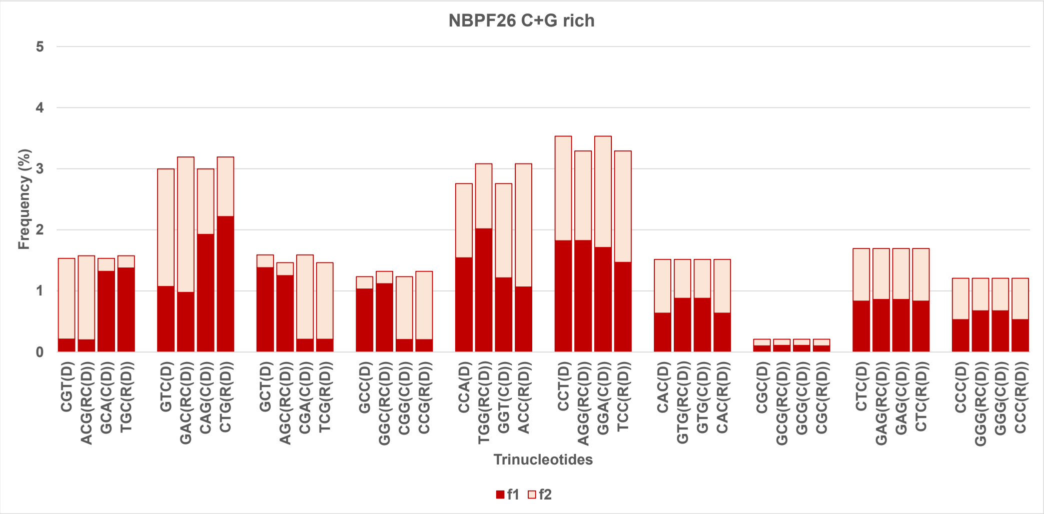

NBPF26 exons

C+G rich												
**D**	**f**	**%**	**RC**	**f**	**%**	**C**	**f**	**%**	**R**	**f**	**%**	
**CGT**	16,00	0,35	ACG	22,00	0,48	GCA	80,00	1,74	TGC	92,00	2,00	IIa
**GTC**	51,00	1,11	GAC	85,00	1,85	CAG	151,00	3,28	CTG	125,00	2,72	
**GCT**	82,00	1,78	AGC	93,00	2,02	CGA	28,00	0,61	TCG	20,00	0,43	
**GCC**	101,00	2,20	GGC	94,00	2,04	CGG	38,00	0,83	CCG	24,00	0,52	IIb
**CCA**	99,00	2,15	TGG	95,00	2,07	GGT	35,00	0,76	ACC	57,00	1,24	
**CCT**	98,00	2,13	AGG	142,00	3,09	GGA	146,00	3,17	TCC	63,00	1,37	
**CAC**	56,00	1,22	GTG	70,00	1,52	GTG*			CAC*			IIc
**CGC**	17,00	0,37	GCG	33,00	0,72	GCG*			CGC*			
**CTC**	106,00	2,30	GAG	132,00	2,87	GAG*			CTC*			
**CCC**	80,00	1,74	GGG	74,00	1,61	GGG*			CCC*			

Quadruplet		f1	f2	fu
CGT	CGT(D)	0,35	1,74	2,09
	ACG(RC(D))	0,48	2,00	2,48
	GCA(C(D))	1,74	0,35	2,09
	TGC(R(D))	2,00	0,48	2,48
GTC	GTC(D)	1,11	3,28	4,39
	GAC(RC(D))	1,85	2,72	4,57
	CAG(C(D))	3,28	1,11	4,39
	CTG(R(D))	2,72	1,85	4,57
GCT	GCT(D)	1,78	0,61	2,39
	AGC(RC(D))	2,02	0,43	2,46
	CGA(C(D))	0,61	1,78	2,39
	TCG(R(D))	0,43	2,02	2,46
GCC	GCC(D)	2,20	0,83	3,02
	GGC(RC(D))	2,04	0,52	2,57
	CGG(C(D))	0,83	2,20	3,02
	CCG(R(D))	0,52	2,04	2,57
CCA	CCA(D)	2,15	0,76	2,91
	TGG(RC(D))	2,07	1,24	3,30
	GGT(C(D))	0,76	2,15	2,91
	ACC(R(D))	1,24	2,07	3,30
CCT	CCT(D)	2,13	3,17	5,30
	AGG(RC(D))	3,09	1,37	4,46
	GGA(C(D))	3,17	2,13	5,30
	TCC(R(D))	1,37	3,09	4,46
CAC	CAC(D)	0,61	0,76	1,37
	GTG(RC(D))	0,76	0,61	1,37
	GTG(C(D))	0,76	0,61	1,37
	CAC(R(D))	0,61	0,76	1,37
CGC	CGC(D)	0,18	0,36	0,54
	GCG(RC(D))	0,36	0,18	0,54
	GCG(C(D))	0,36	0,18	0,54
	CGC(R(D))	0,18	0,36	0,54
CTC	CTC(D)	1,15	1,43	2,59
	GAG(RC(D))	1,43	1,15	2,59
	GAG(C(D))	1,43	1,15	2,59
	CTC(R(D))	1,15	1,43	2,59
CCC	CCC(D)	0,87	0,80	1,67
	GGG(RC(D))	0,80	0,87	1,67
	GGG(C(D))	0,80	0,87	1,67
	CCC(R(D))	0,87	0,80	1,67

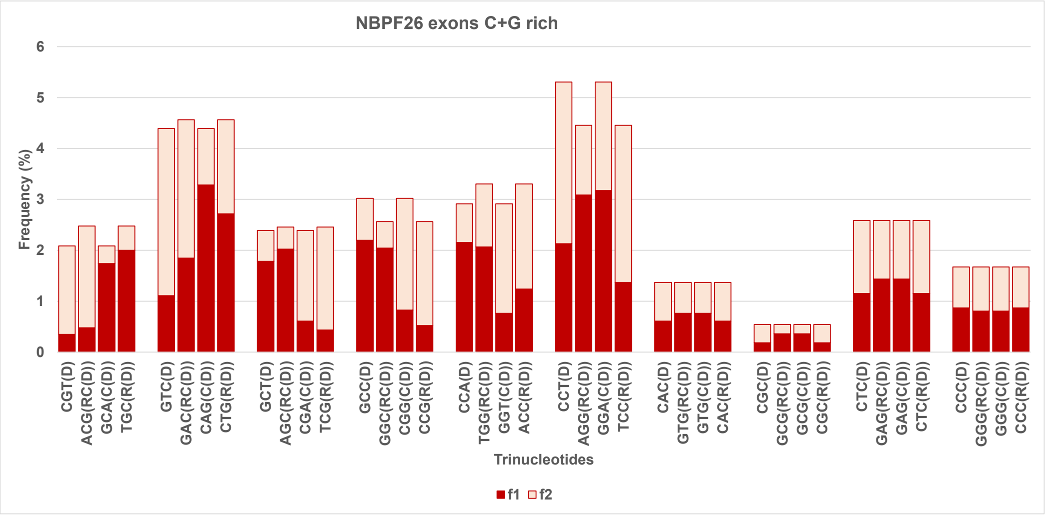

NBPF26 introns

C+G rich

D	f	%	RC	f	%	C	f	%	R	f	%	
**CGT**	229,00	0,20	ACG	203,00	0,18	GCA	1452,00	1,30	TGC	1508,00	1,35	IIa
**GTC**	1193,00	1,06	GAC	1046,00	0,93	CAG	2084,00	1,86	CTG	2454,00	2,19	
**GCT**	1525,00	1,36	AGC	1359,00	1,21	CGA	213,00	0,19	TCG	224,00	0,20	
**GCC**	1090,00	0,97	GGC	1197,00	1,07	CGG	182,00	0,16	CCG	200,00	0,18	IIb
**CCA**	1695,00	1,51	TGG	2258,00	2,01	GGT	1389,00	1,24	ACC	1179,00	1,05	
**CCT**	2017,00	1,80	AGG	1988,00	1,77	GGA	1842,00	1,64	TCC	1643,00	1,47	
**CAC**	1416,00	1,26	GTG	1989,00	1,77	GTG*			CAC*			IIc
**CGC**	207,00	0,18	GCG	204,00	0,18	GCG*			CGC*			
**CTC**	1836,00	1,64	GAG	1874,00	1,67	GAG*			CTC*			
**CCC**	1145,00	1,02	GGG	1503,00	1,34	GGG*			CCC*			

Quadruplet		f1	f2	fu
CGT	CGT(D)	0,20	1,30	1,50
	ACG(RC(D))	0,18	1,35	1,53
	GCA(C(D))	1,30	0,20	1,50
	TGC(R(D))	1,35	0,18	1,53
GTC	GTC(D)	1,06	1,86	2,92
	GAC(RC(D))	0,93	2,19	3,12
	CAG(C(D))	1,86	1,06	2,92
	CTG(R(D))	2,19	0,93	3,12
GCT	GCT(D)	1,36	0,19	1,55
	AGC(RC(D))	1,21	0,20	1,41
	CGA(C(D))	0,19	1,36	1,55
	TCG(R(D))	0,20	1,21	1,41
GCC	GCC(D)	0,97	0,16	1,14
	GGC(RC(D)	1,07	0,18	1,25
	CGG(C(D))	0,16	0,97	1,14
	CCG(R(D))	0,18	1,07	1,25
CCA	CCA(D)	1,51	1,24	2,75
	TGG(RC(D))	2,01	1,05	3,07
	GGT(C(D))	1,24	1,51	2,75
	ACC(R(D))	1,05	2,01	3,07
CCT	CCT(D)	1,80	1,64	3,44
	AGG(RC(D))	1,77	1,47	3,24
	GGA(C(D))	1,64	1,80	3,44
	TCC(R(D))	1,47	1,77	3,24
CAC	CAC(D)	0,63	0,89	1,52
	GTG(RC(D))	0,89	0,63	1,52
	GTG(C(D))	0,89	0,63	1,52
	CAC(R(D))	0,63	0,89	1,52
CGC	CGC(D)	0,09	0,09	0,18
	GCG(RC(D)	0,09	0,09	0,18
	GCG(C(D))	0,09	0,09	0,18
	CGC(R(D))	0,09	0,09	0,18
CTC	CTC(D)	0,82	0,84	1,66
	GAG(RC(D))	0,84	0,82	1,66
	GAG(C(D))	0,84	0,82	1,66
	CTC(R(D))	0,82	0,84	1,66
CCC	CCC(D)	0,51	0,67	1,18
	GGG(RC(D)	0,67	0,51	1,18
	GGG(C(D))	0,67	0,51	1,18
	CCC(R(D))	0,51	0,67	1,18

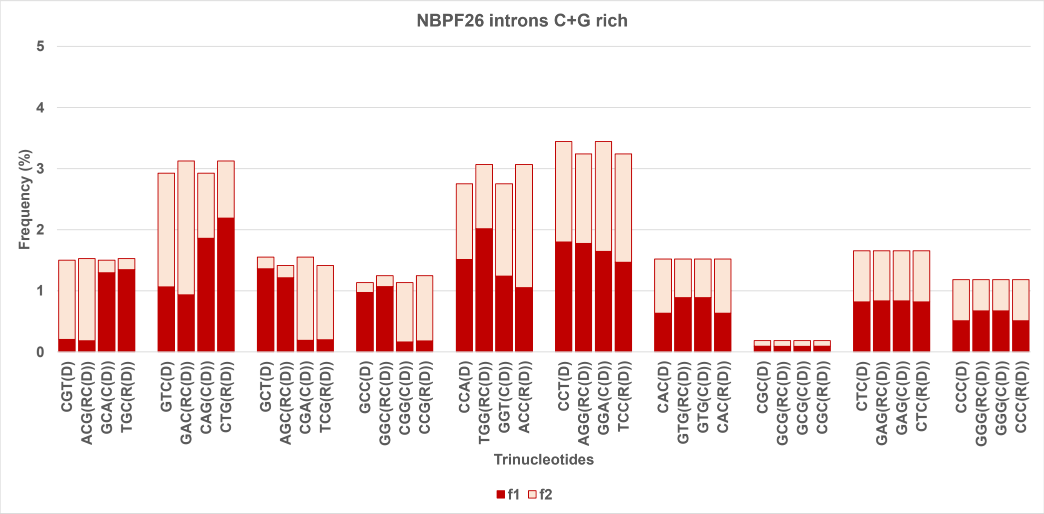

Supplement: Supplementary file 1 [file genes-13-01929-s001.zip › Supplemetary Table S10.pdf]
