# Supplementary material for: An Explanation of Exceptions from Chargaff’s Second Parity Rule/Strand Symmetry of DNA Molecules
Source: Genes (Basel). 2022 Oct 23;13(11):1929. doi: 10.3390/genes13111929 (PMC9689577; doi:10.3390/genes13111929)

Human assembled chromomome 1

A-T rich												
**D**	**f**	**%**	**RC**	**f**	**%**	**C**	**f**	**%**	**R**	**f**	**%**	
**ATG**	4110209,00	1,78	CAT	4120946,00	1,79	TAC	2542958,00	1,10	GTA	2566721,00	1,11	Ia
**TGA**	4486632,00	1,95	TCA	4522569,00	1,96	ACT	3732934,00	1,62	AGT	3719675,00	1,61	
**TAG**	2960898,00	1,28	CTA	2941433,00	1,28	ATC	3035996,00	1,32	GAT	3056974,00	1,33	
**TAA**	4577976,00	1,99	TTA	4571528,00	1,98	ATT	5500468,00	2,39	AAT	5470905,00	2,37	Ib
**AAC**	3332435,00	1,45	GTT	3329970,00	1,44	TTG	4344678,00	1,89	CAA	4288540,00	1,86	
**AAG**	4581648,00	1,99	CTT	4634644,00	2,01	TTC	4548877,00	1,97	GAA	4518460,00	1,96	
**ACA**	4553751,00	1,98	TGT	4584113,00	1,99	TGT*			ACA*			Ic
**ATA**	4475100,00	1,94	TAT	4472951,00	1,94	TAT*			ATA*			
**AGA**	5150760,00	2,23	TCT	5129424,00	2,23	TCT*			AGA*			
**AAA**	8516543,00	3,70	TTT	8583142,00	3,72	TTT*			AAA*			

Quadruplet		f1	f2	fu
ATG	ATG(D)	1,78	1,10	2,89
	CAT(RC(D))	1,79	1,11	2,90
	TAC(C(D))	1,10	1,78	2,89
	GTA(R(D))	1,11	1,79	2,90
TGA	TGA(D)	1,95	1,62	3,57
	TCA(RC(D))	1,96	1,61	3,58
	ACT(C(D))	1,62	1,95	3,57
	AGT(R(D))	1,61	1,96	3,58
TAG	TAG(D)	1,28	1,32	2,60
	CTA(RC(D))	1,28	1,33	2,60
	ATC(C(D))	1,32	1,28	2,60
	GAT(R(D))	1,33	1,28	2,60
TAA	TAA(D)	1,99	2,39	4,37
	TTA(RC(D))	1,98	2,37	4,36
	ATT(C(D))	2,39	1,99	4,37
	AAT(R(D))	2,37	1,98	4,36
AAC	AAC(D)	1,45	1,89	3,33
	GTT(RC(D))	1,44	1,86	3,31
	TTG(C(D))	1,89	1,45	3,33
	CAA(R(D))	1,86	1,44	3,31
AAG	AAG(D)	1,99	1,97	3,96
	CTT(RC(D))	2,01	1,96	3,97
	TTC(C(D))	1,97	1,99	3,96
	GAA(R(D))	1,96	2,01	3,97
ACA	ACA(D)	0,99	0,99	1,98
	TGT(RC(D))	0,99	0,99	1,98
	TGT(C(D))	0,99	0,99	1,98
	ACA(R(D))	0,99	0,99	1,98
ATA	ATA(D)	0,97	0,97	1,94
	TAT(RC(D))	0,97	0,97	1,94
	TAT(C(D))	0,97	0,97	1,94
	ATA(R(D))	0,97	0,97	1,94
AGA	AGA(D)	1,12	1,11	2,23
	TCT(RC(D))	1,11	1,12	2,23
	TCT(C(D))	1,11	1,12	2,23
	AGA(R(D))	1,12	1,11	2,23
AAA	AAA(D)	1,85	1,86	3,71
	TTT(RC(D))	1,86	1,85	3,71
	TTT(C(D))	1,86	1,85	3,71
	AAA(R(D))	1,85	1,86	3,71

Composition of DNA sequence in assembled chromosome 1

Base	No.of bases	%
A	67070277	26,94%
C	48055043	19,30%
G	48111528	19,33%
T	67244164	27,01%
N	18475410	7,42%
Sum	248956422	100,00%

% of coding bases from complete assembled chromosome 1 (including N bases)

6,91%

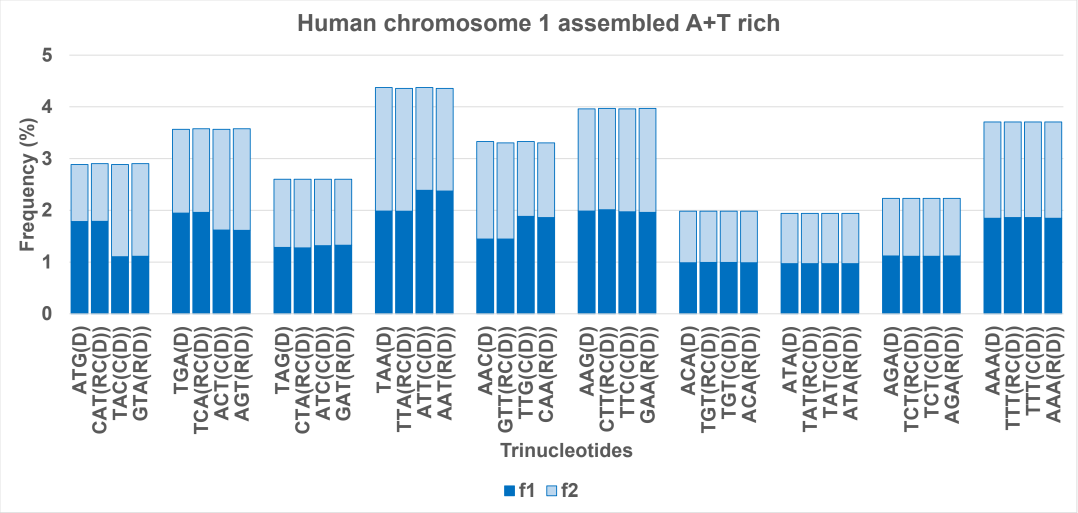

Human random chromomome 1

						A-T rich						
**D**	**f**	**%**	**RC**	**f**	**%**	**C**	**f**	**%**	**R**	**f**	**%**	
**ATG**	3313,00	1,66	CAT	3383,00	1,70	TAC	2115,00	1,06	GTA	2048,00	1,03	Ia
**TGA**	3810,00	1,91	TCA	3910,00	1,96	ACT	3098,00	1,56	AGT	3141,00	1,58	
**TAG**	2356,00	1,18	CTA	2479,00	1,24	ATC	2602,00	1,31	GAT	2482,00	1,25	
**TAA**	3285,00	1,65	TTA	3375,00	1,69	ATT	4157,00	2,09	AAT	4061,00	2,04	Ib
**AAC**	2631,00	1,32	GTT	2600,00	1,31	TTG	3544,00	1,78	CAA	3584,00	1,80	
**AAG**	3742,00	1,88	CTT	3715,00	1,87	TTC	3553,00	1,78	GAA	3565,00	1,79	
**ACA**	3765,00	1,89	TGT	3697,00	1,86	TGT*			ACA*			Ic
**ATA**	3101,00	1,56	TAT	3248,00	1,63	TAT*			ATA*			
**AGA**	4219,00	2,12	TCT	4308,00	2,16	TCT*			AGA*			
**AAA**	6714,00	3,37	TTT	6473,00	3,25	TTT*			AAA*			

Quadruplet		f1	f2	fu
ATG	ATG(D)	1,66	1,06	2,73
	CAT(RC(D))	1,70	1,03	2,73
	TAC(C(D))	1,06	1,66	2,73
	GTA(R(D))	1,03	1,70	2,73
TGA	TGA(D)	1,91	1,56	3,47
	TCA(RC(D))	1,96	1,58	3,54
	ACT(C(D))	1,56	1,91	3,47
	AGT(R(D))	1,58	1,96	3,54
TAG	TAG(D)	1,18	1,31	2,49
	CTA(RC(D))	1,24	1,25	2,49
	ATC(C(D))	1,31	1,18	2,49
	GAT(R(D))	1,25	1,24	2,49
TAA	TAA(D)	1,65	2,09	3,74
	TTA(RC(D))	1,69	2,04	3,73
	ATT(C(D))	2,09	1,65	3,74
	AAT(R(D))	2,04	1,69	3,73
AAC	AAC(D)	1,32	1,78	3,10
	GTT(RC(D))	1,31	1,80	3,10
	TTG(C(D))	1,78	1,32	3,10
	CAA(R(D))	1,80	1,31	3,10
AAG	AAG(D)	1,88	1,78	3,66
	CTT(RC(D))	1,87	1,79	3,65
	TTC(C(D))	1,78	1,88	3,66
	GAA(R(D))	1,79	1,87	3,65
ACA	ACA(D)	0,95	0,93	1,87
	TGT(RC(D))	0,93	0,95	1,87
	TGT(C(D))	0,93	0,95	1,87
	ACA(R(D))	0,95	0,93	1,87
ATA	ATA(D)	0,78	0,82	1,59
	TAT(RC(D))	0,82	0,78	1,59
	TAT(C(D))	0,82	0,78	1,59
	ATA(R(D))	0,78	0,78	1,56
AGA	AGA(D)	1,06	1,08	2,14
	TCT(RC(D))	1,08	1,06	2,14
	TCT(C(D))	1,08	1,06	2,14
	AGA(R(D))	1,06	1,08	2,14
AAA	AAA(D)	1,69	1,62	3,31
	TTT(RC(D))	1,62	1,69	3,31
	TTT(C(D))	1,62	1,69	3,31
	AAA(R(D))	1,69	1,62	3,31

Composition of DNA sequence in random chromosome 1

Base	No.of bases	%
A	55030	27,52%
C	44870	22,44%
G	44403	22,20%
T	54897	27,45%
N	800	0,40%
Sum	200000	100,00%

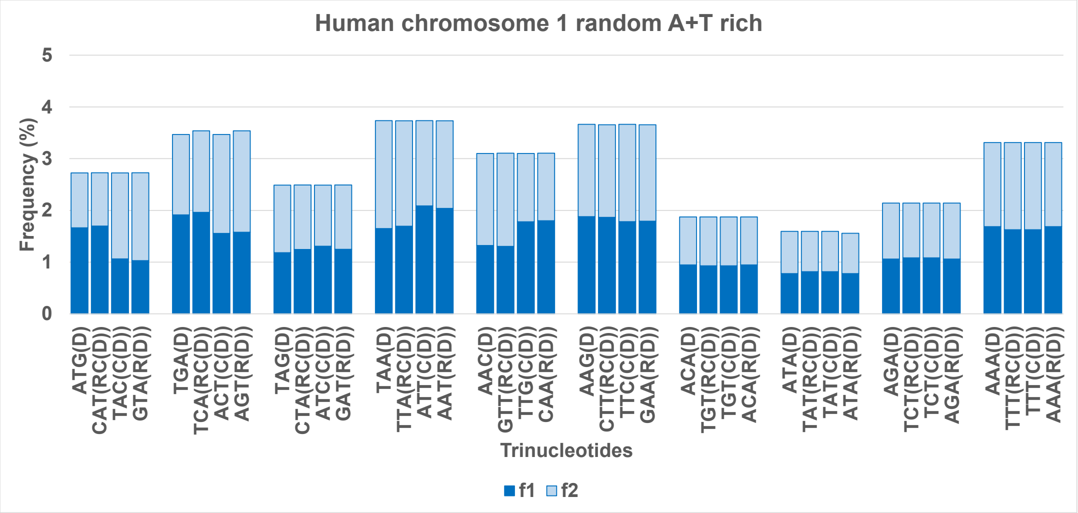

Human coding chromomome 1

A-T rich												
**D**	**f**	**%**	**RC**	**f**	**%**	**C**	**f**	**%**	**R**	**f**	**%**	
**ATG**	298293,00	1,73	CAT	262940,00	1,53	TAC	165107,00	0,96	GTA	141268,00	0,82	Ia
**TGA**	333511,00	1,94	TCA	301920,00	1,76	ACT	247324,00	1,44	AGT	247875,00	1,44	
**TAG**	137339,00	0,80	CTA	171408,00	1,00	ATC	211863,00	1,23	GAT	229766,00	1,34	
**TAA**	199316,00	1,16	TTA	207699,00	1,21	ATT	267785,00	1,56	AAT	266622,00	1,55	Ib
**AAC**	224977,00	1,31	GTT	205537,00	1,19	TTG	278640,00	1,62	CAA	293767,00	1,71	
**AAG**	372789,00	2,17	CTT	305835,00	1,78	TTC	292818,00	1,70	GAA	371305,00	2,16	
**ACA**	293253,00	1,70	TGT	301144,00	1,75	TGT*			ACA*			Ic
**ATA**	187216,00	1,09	TAT	205829,00	1,20	TAT*			ATA*			
**AGA**	400504,00	2,33	TCT	315917,00	1,84	TCT*			AGA*			
**AAA**	431811,00	2,51	TTT	403803,00	2,35	TTT*			AAA*			

Quadruplet		f1	f2	fu
ATG	ATG(D)	1,73	0,96	2,69
	CAT(RC(D))	1,53	0,82	2,35
	TAC(C(D))	0,96	1,73	2,69
	GTA(R(D))	0,82	1,53	2,35
TGA	TGA(D)	1,94	1,44	3,38
	TCA(RC(D))	1,76	1,44	3,20
	ACT(C(D))	1,44	1,94	3,38
	AGT(R(D))	1,44	1,76	3,20
TAG	TAG(D)	0,80	1,23	2,03
	CTA(RC(D))	1,00	1,34	2,33
	ATC(C(D))	1,23	0,80	2,03
	GAT(R(D))	1,34	1,00	2,33
TAA	TAA(D)	1,16	1,56	2,72
	TTA(RC(D))	1,21	1,55	2,76
	ATT(C(D))	1,56	1,16	2,72
	AAT(R(D))	1,55	1,21	2,76
AAC	AAC(D)	1,31	1,62	2,93
	GTT(RC(D))	1,19	1,71	2,90
	TTG(C(D))	1,62	1,31	2,93
	CAA(R(D))	1,71	1,19	2,90
AAG	AAG(D)	2,17	1,70	3,87
	CTT(RC(D))	1,78	2,16	3,94
	TTC(C(D))	1,70	2,17	3,87
	GAA(R(D))	2,16	1,78	3,94
ACA	ACA(D)	0,85	0,88	1,73
	TGT(RC(D))	0,88	0,85	1,73
	TGT(C(D))	0,88	0,85	1,73
	ACA(R(D))	0,85	0,88	1,73
ATA	ATA(D)	0,54	0,60	1,14
	TAT(RC(D))	0,60	0,54	1,14
	TAT(C(D))	0,60	0,54	1,14
	ATA(R(D))	0,54	0,54	1,09
AGA	AGA(D)	1,16	0,92	2,08
	TCT(RC(D))	0,92	1,16	2,08
	TCT(C(D))	0,92	1,16	2,08
	AGA(R(D))	1,16	0,92	2,08
AAA	AAA(D)	1,26	1,17	2,43
	TTT(RC(D))	1,17	1,26	2,43
	TTT(C(D))	1,17	1,26	2,43
	AAA(R(D))	1,26	1,17	2,43

Composition of DNA sequence in coding chromosome 1

Base	No.of bases	%
A	4475849	26,02%
C	4208900	24,47%
G	4288999	24,93%
T	4228820	24,58%
N	0	0,00%
Sum	17202568	100,00%

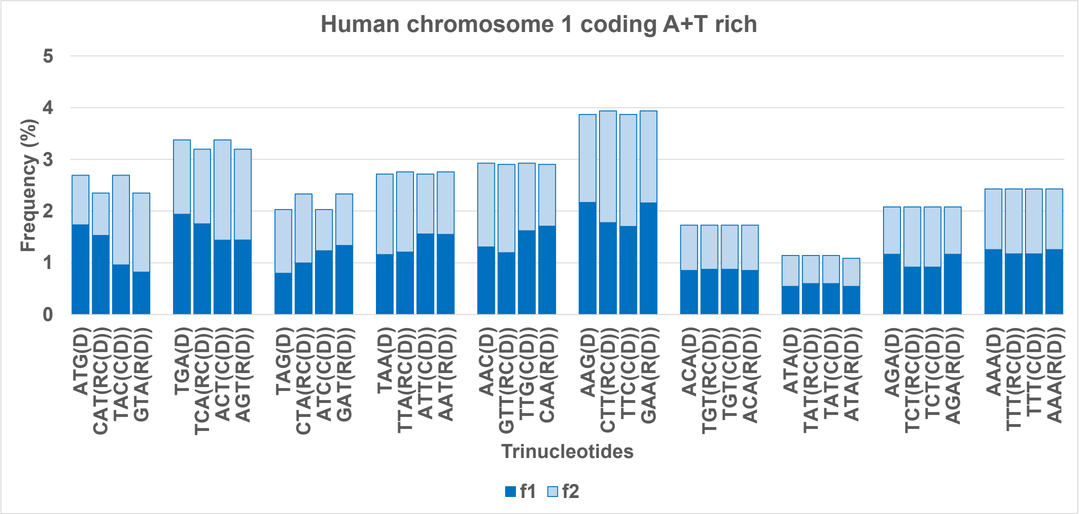

						C+G rich						
**D**	**f**	**%**	**RC**	**f**	**%**	**C**	**f**	**%**	**R**	**f**	**%**	
**CGT**	597422,00	0,26	ACG	586276,00	0,25	GCA	3361131,00	1,46	TGC	3357313,00	1,46	IIa
**GTC**	2202280,00	0,96	GAC	2216474,00	0,96	CAG	4852390,00	2,11	CTG	4811169,00	2,09	
**GCT**	3309131,00	1,44	AGC	3317232,00	1,44	CGA	523798,00	0,23	TCG	535651,00	0,23	
**GCC**	2891387,00	1,25	GGC	2891408,00	1,25	CGG	674618,00	0,29	CCG	669612,00	0,29	IIb
**CCA**	4330820,00	1,88	TGG	4368306,00	1,90	GGT	2728078,00	1,18	ACC	2725309,00	1,18	
**CCT**	4273302,00	1,85	AGG	4260968,00	1,85	GGA	3684403,00	1,60	TCC	3657040,00	1,59	
**CAC**	3506405,00	1,52	GTG	3530308,00	1,53	GTG*			CAC*			IIc
**CGC**	579316,00	0,25	GCG	583618,00	0,25	GCG*			CGC*			
**CTC**	4057534,00	1,76	GAG	4053693,00	1,76	GAG*			CTC*			
**CCC**	3193020,00	1,39	GGG	3187415,00	1,38	GGG*			CCC*			

Quadruplet		f1	f2	fu
CGT	CGT(D)	0,26	1,46	1,72
	ACG(RC(D))	0,25	1,46	1,71
	GCA(C(D))	1,46	0,26	1,72
	TGC(R(D))	1,46	0,25	1,71
GTC	GTC(D)	0,96	2,11	3,06
	GAC(RC(D))	0,96	2,09	3,05
	CAG(C(D))	2,11	0,96	3,06
	CTG(R(D))	2,09	0,96	3,05
GCT	GCT(D)	1,44	0,23	1,66
	AGC(RC(D))	1,44	0,23	1,67
	CGA(C(D))	0,23	1,44	1,66
	TCG(R(D))	0,23	1,44	1,67
GCC	GCC(D)	1,25	0,29	1,55
	GGC(RC(D))	1,25	0,29	1,55
	CGG(C(D))	0,29	1,25	1,55
	CCG(R(D))	0,29	1,25	1,55
CCA	CCA(D)	1,88	1,18	3,06
	TGG(RC(D))	1,90	1,18	3,08
	GGT(C(D))	1,18	1,88	3,06
	ACC(R(D))	1,18	1,90	3,08
CCT	CCT(D)	1,85	1,60	3,45
	AGG(RC(D))	1,85	1,59	3,44
	GGA(C(D))	1,60	1,85	3,45
	TCC(R(D))	1,59	1,85	3,44
CAC	CAC(D)	0,76	0,77	1,53
	GTG(RC(D))	0,77	0,76	1,53
	GTG(C(D))	0,77	0,76	1,53
	CAC(R(D))	0,76	0,77	1,53
CGC	CGC(D)	0,13	0,13	0,25
	GCG(RC(D))	0,13	0,13	0,25
	GCG(C(D))	0,13	0,13	0,25
	CGC(R(D))	0,13	0,13	0,25
CTC	CTC(D)	0,88	0,88	1,76
	GAG(RC(D))	0,88	0,88	1,76
	GAG(C(D))	0,88	0,88	1,76
	CTC(R(D))	0,88	0,88	1,76
CCC	CCC(D)	0,69	0,69	1,38
	GGG(RC(D))	0,69	0,69	1,38
	GGG(C(D))	0,69	0,69	1,38
	CCC(R(D))	0,69	0,69	1,38

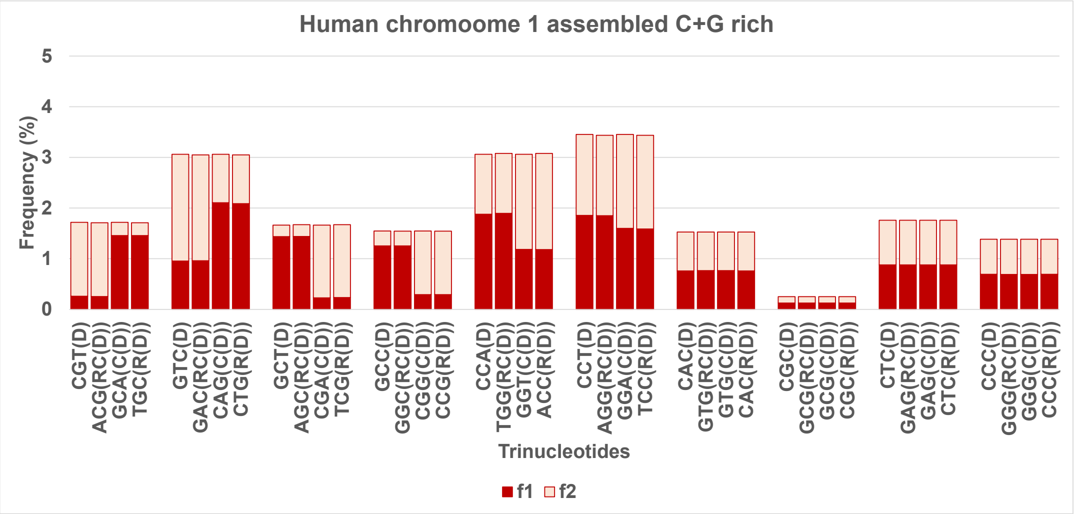

Human random chromomome 1

C+G rich												
**D**	**f**	**%**	**RC**	**f**	**%**	**C**	**f**	**%**	**R**	**f**	**%**	
**CGT**	575,00	0,29	ACG	584,00	0,29	GCA	3104,00	1,56	TGC	3111,00	1,56	IIa
**GTC**	2143,00	1,08	GAC	1983,00	1,00	CAG	4738,00	2,38	CTG	4638,00	2,33	
**GCT**	3158,00	1,59	AGC	3255,00	1,63	CGA	531,00	0,27	TCG	505,00	0,25	
**GCC**	3110,00	1,56	GGC	3042,00	1,53	CGG	790,00	0,40	CCG	772,00	0,39	IIb
**CCA**	4225,00	2,12	TGG	4149,00	2,08	GGT	2651,00	1,33	ACC	2581,00	1,30	
**CCT**	4149,00	2,08	AGG	4064,00	2,04	GGA	3312,00	1,66	TCC	3455,00	1,73	
**CAC**	3299,00	1,66	GTG	3273,00	1,64	GTG*			CAC*			IIc
**CGC**	660,00	0,33	GCG	695,00	0,35	GCG*			CGC*			
**CTC**	3881,00	1,95	GAG	3843,00	1,93	GAG*			CTC*			
**CCC**	3449,00	1,73	GGG	3391,00	1,70	GGG*			CCC*			

Quadruplet		f1	f2	fu
CGT	CGT(D)	0,29	1,56	1,85
	ACG(RC(D))	0,29	1,56	1,86
	GCA(C(D))	1,56	0,29	1,85
	TGC(R(D))	1,56	0,29	1,86
GTC	GTC(D)	1,08	2,38	3,45
	GAC(RC(D))	1,00	2,33	3,32
	CAG(C(D))	2,38	1,08	3,45
	CTG(R(D))	2,33	1,00	3,32
GCT	GCT(D)	1,59	0,27	1,85
	AGC(RC(D))	1,63	0,25	1,89
	CGA(C(D))	0,27	1,59	1,85
	TCG(R(D))	0,25	1,63	1,89
GCC	GCC(D)	1,56	0,40	1,96
	GGC(RC(D))	1,53	0,39	1,91
	CGG(C(D))	0,40	1,56	1,96
	CCG(R(D))	0,39	1,53	1,91
CCA	CCA(D)	2,12	1,33	3,45
	TGG(RC(D))	2,08	1,30	3,38
	GGT(C(D))	1,33	2,12	3,45
	ACC(R(D))	1,30	2,08	3,38
CCT	CCT(D)	2,08	1,66	3,75
	AGG(RC(D))	2,04	1,73	3,77
	GGA(C(D))	1,66	2,08	3,75
	TCC(R(D))	1,73	2,04	3,77
CAC	CAC(D)	0,83	0,82	1,65
	GTG(RC(D))	0,82	0,83	1,65
	GTG(C(D))	0,82	0,83	1,65
	CAC(R(D))	0,83	0,82	1,65
CGC	CGC(D)	0,17	0,17	0,34
	GCG(RC(D))	0,17	0,17	0,34
	GCG(C(D))	0,17	0,17	0,34
	CGC(R(D))	0,17	0,17	0,34
CTC	CTC(D)	0,97	0,96	1,94
	GAG(RC(D))	0,96	0,97	1,94
	GAG(C(D))	0,96	0,97	1,94
	CTC(R(D))	0,97	0,96	1,94
CCC	CCC(D)	0,87	0,85	1,72
	GGG(RC(D))	0,85	0,87	1,72
	GGG(C(D))	0,85	0,87	1,72
	CCC(R(D))	0,87	0,85	1,72

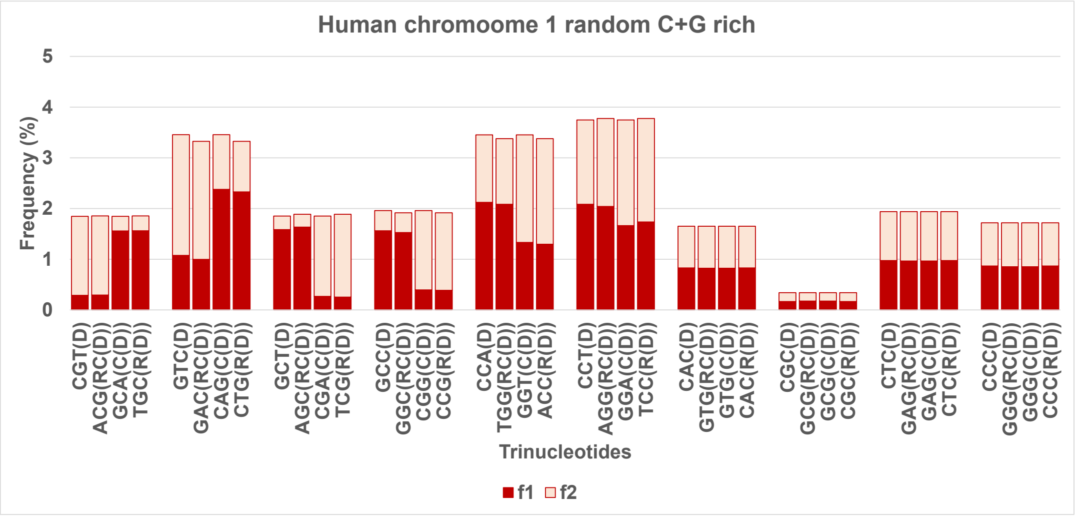

Human coding chromomome 1

						C+G rich						
**D**	**f**	**%**	**RC**	**f**	**%**	**C**	**f**	**%**	**R**	**f**	**%**	
**CGT**	77861,00	0,45	ACG	84674,00	0,49	GCA	298670,00	1,74	TGC	307274,00	1,79	IIa
**GTC**	185039,00	1,08	GAC	226267,00	1,32	CAG	456348,00	2,65	CTG	461006,00	2,68	
**GCT**	314762,00	1,83	AGC	333696,00	1,94	CGA	88722,00	0,52	TCG	79043,00	0,46	
**GCC**	337090,00	1,96	GGC	313432,00	1,82	CGG	142587,00	0,83	CCG	141572,00	0,82	IIb
**CCA**	383181,00	2,23	TGG	392533,00	2,28	GGT	201487,00	1,17	ACC	255069,00	1,48	
**CCT**	374333,00	2,18	AGG	352097,00	2,05	GGA	372298,00	2,16	TCC	306927,00	1,78	
**CAC**	263969,00	1,53	GTG	296523,00	1,72	GTG*			CAC*			IIc
**CGC**	117916,00	0,69	GCG	121796,00	0,71	GCG*			CGC*			
**CTC**	314087,00	1,83	GAG	367696,00	2,14	GAG*			CTC*			
**CCC**	353368,00	2,05	GGG	306062,00	1,78	GGG*			CCC*			

Quadruplet		f1	f2	fu
CGT	CGT(D)	0,45	1,74	2,19
	ACG(RC(D))	0,49	1,79	2,28
	GCA(C(D))	1,74	0,45	2,19
	TGC(R(D))	1,79	0,49	2,28
GTC	GTC(D)	1,08	2,65	3,73
	GAC(RC(D))	1,32	2,68	4,00
	CAG(C(D))	2,65	1,08	3,73
	CTG(R(D))	2,68	1,32	4,00
GCT	GCT(D)	1,83	0,52	2,35
	AGC(RC(D))	1,94	0,46	2,40
	CGA(C(D))	0,52	1,83	2,35
	TCG(R(D))	0,46	1,94	2,40
GCC	GCC(D)	1,96	0,83	2,79
	GGC(RC(D))	1,82	0,82	2,64
	CGG(C(D))	0,83	1,96	2,79
	CCG(R(D))	0,82	1,82	2,64
CCA	CCA(D)	2,23	1,17	3,40
	TGG(RC(D))	2,28	1,48	3,76
	GGT(C(D))	1,17	2,23	3,40
	ACC(R(D))	1,48	2,28	3,76
CCT	CCT(D)	2,18	2,16	4,34
	AGG(RC(D))	2,05	1,78	3,83
	GGA(C(D))	2,16	2,18	4,34
	TCC(R(D))	1,78	2,05	3,83
CAC	CAC(D)	0,77	0,86	1,63
	GTG(RC(D))	0,86	0,77	1,63
	GTG(C(D))	0,86	0,77	1,63
	CAC(R(D))	0,77	0,86	1,63
CGC	CGC(D)	0,34	0,35	0,70
	GCG(RC(D))	0,35	0,34	0,70
	GCG(C(D))	0,35	0,34	0,70
	CGC(R(D))	0,34	0,35	0,70
CTC	CTC(D)	0,91	1,07	1,98
	GAG(RC(D))	1,07	0,91	1,98
	GAG(C(D))	1,07	0,91	1,98
	CTC(R(D))	0,91	1,07	1,98
CCC	CCC(D)	1,03	0,89	1,92
	GGG(RC(D))	0,89	1,03	1,92
	GGG(C(D))	0,89	1,03	1,92
	CCC(R(D))	1,03	0,89	1,92

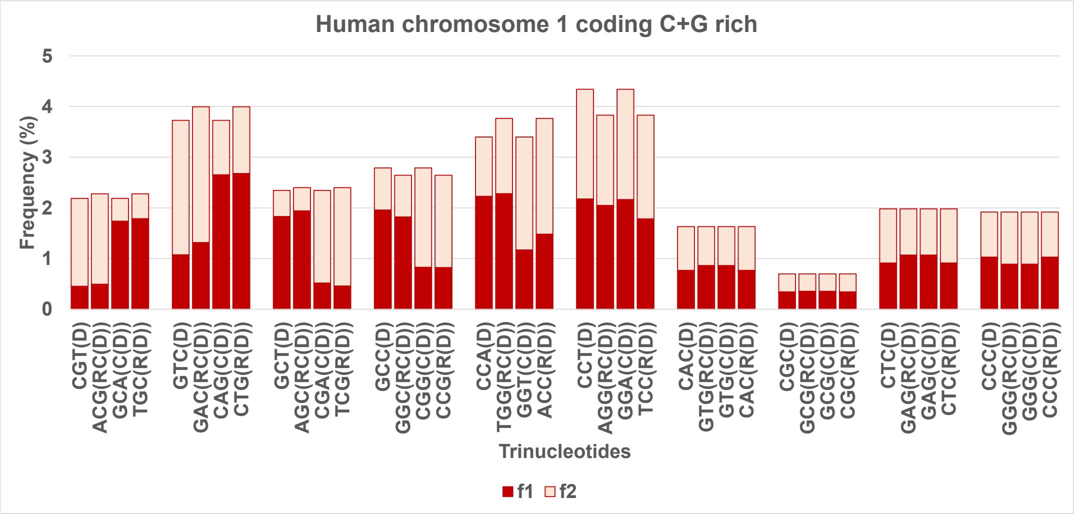

						A-T rich						
**D**	**f**	**%**	**RC**	**f**	**%**	**C**	**f**	**%**	**R**	**f**	**%**	
**ATG**	4465442,00	1,86	CAT	4482218,00	1,86	TAC	2757264,00	1,15	GTA	2767750,00	1,15	Ia
**TGA**	4752502,00	1,98	TCA	4739981,00	1,97	ACT	3894571,00	1,62	AGT	3908159,00	1,62	
**TAG**	3156134,00	1,31	CTA	3144525,00	1,31	ATC	3216224,00	1,34	GAT	3243804,00	1,35	
**TAA**	5147915,00	2,14	TTA	5145518,00	2,14	ATT	6182527,00	2,57	AAT	6141693,00	2,55	Ib
**AAC**	3559599,00	1,48	GTT	3565015,00	1,48	TTG	4651511,00	1,93	CAA	4569131,00	1,90	
**AAG**	4859279,00	2,02	CTT	4892402,00	2,03	TTC	4842914,00	2,01	GAA	4843524,00	2,01	
**ACA**	4893627,00	2,03	TGT	4916964,00	2,04	TGT*			ACA*			Ic
**ATA**	5123234,00	2,13	TAT	5119713,00	2,13	TAT*			ATA*			
**AGA**	5353864,00	2,23	TCT	5370890,00	2,23	TCT*			AGA*			
**AAA**	9416331,00	3,91	TTT	9508514,00	3,95	TTT*			AAA*			

Quadruplet		f1	f2	fu
ATG	ATG(D)	1,86	1,15	3,00
	CAT(RC(D))	1,86	1,15	3,01
	TAC(C(D))	1,15	1,86	3,00
	GTA(R(D))	1,15	1,86	3,01
TGA	TGA(D)	1,98	1,62	3,59
	TCA(RC(D))	1,97	1,62	3,60
	ACT(C(D))	1,62	1,98	3,59
	AGT(R(D))	1,62	1,97	3,60
TAG	TAG(D)	1,31	1,34	2,65
	CTA(RC(D))	1,31	1,35	2,66
	ATC(C(D))	1,34	1,31	2,65
	GAT(R(D))	1,35	1,31	2,66
TAA	TAA(D)	2,14	2,57	4,71
	TTA(RC(D))	2,14	2,55	4,69
	ATT(C(D))	2,57	2,14	4,71
	AAT(R(D))	2,55	2,14	4,69
AAC	AAC(D)	1,48	1,93	3,41
	GTT(RC(D))	1,48	1,90	3,38
	TTG(C(D))	1,93	1,48	3,41
	CAA(R(D))	1,90	1,48	3,38
AAG	AAG(D)	2,02	2,01	4,03
	CTT(RC(D))	2,03	2,01	4,05
	TTC(C(D))	2,01	2,02	4,03
	GAA(R(D))	2,01	2,03	4,05
ACA	ACA(D)	1,02	1,02	2,04
	TGT(RC(D))	1,02	1,02	2,04
	TGT(C(D))	1,02	1,02	2,04
	ACA(R(D))	1,02	1,02	2,04
ATA	ATA(D)	1,06	1,06	2,13
	TAT(RC(D))	1,06	1,06	2,13
	TAT(C(D))	1,06	1,06	2,13
	ATA(R(D))	1,06	1,06	2,13
AGA	AGA(D)	1,11	1,12	2,23
	TCT(RC(D))	1,12	1,11	2,23
	TCT(C(D))	1,12	1,11	2,23
	AGA(R(D))	1,11	1,12	2,23
AAA	AAA(D)	1,96	1,98	3,93
	TTT(RC(D))	1,98	1,96	3,93
	TTT(C(D))	1,98	1,96	3,93
	AAA(R(D))	1,96	1,98	3,93

Composition of DNA sequence in assembled chromosome 2

Base	No.of bases	%
A	71791213	29,64%
C	48318180	19,95%
G	48450903	20,01%
T	71987932	29,72%
N	1645301	0,68%
Sum	242193529	100,00%

% of coding bases from complete assembled chromosome 1 (including N bases)

5,47%

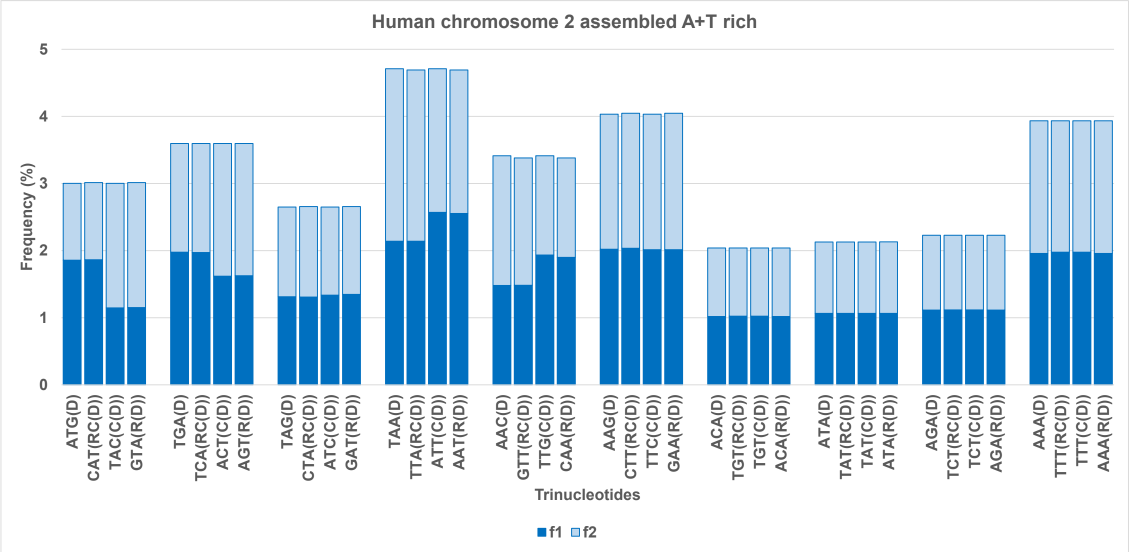

Human random chromomome 2

A-T rich

D	f	%	RC	f	%	C	f	%	R	f	%	
**ATG**	3612,00	1,81	CAT	3710,00	1,86	TAC	2270,00	1,14	GTA	2244,00	1,12	Ia
**TGA**	3906,00	1,95	TCA	3908,00	1,95	ACT	3280,00	1,64	AGT	3138,00	1,57	
**TAG**	2555,00	1,28	CTA	2596,00	1,30	ATC	2742,00	1,37	GAT	2691,00	1,35	
**TAA**	4083,00	2,04	TTA	4057,00	2,03	ATT	4917,00	2,46	AAT	4881,00	2,44	Ib
**AAC**	2899,00	1,45	GTT	2843,00	1,42	TTG	3782,00	1,89	CAA	3801,00	1,90	
**AAG**	3936,00	1,97	CTT	3953,00	1,98	TTC	3874,00	1,94	GAA	3833,00	1,92	
**ACA**	4066,00	2,03	TGT	4040,00	2,02	TGT*			ACA*			Ic
**ATA**	4009,00	2,00	TAT	3998,00	2,00	TAT*			ATA*			
**AGA**	4302,00	2,15	TCT	4444,00	2,22	TCT*			AGA*			
**AAA**	7735,00	3,87	TTT	7435,00	3,72	TTT*			AAA*			

Quadruplet		f1	f2	fu
ATG	ATG(D)	1,81	1,14	2,94
	CAT(RC(D))	1,86	1,12	2,98
	TAC(C(D))	1,14	1,81	2,94
	GTA(R(D))	1,12	1,86	2,98
TGA	TGA(D)	1,95	1,64	3,59
	TCA(RC(D))	1,95	1,57	3,52
	ACT(C(D))	1,64	1,95	3,59
	AGT(R(D))	1,57	1,95	3,52
TAG	TAG(D)	1,28	1,37	2,65
	CTA(RC(D))	1,30	1,35	2,64
	ATC(C(D))	1,37	1,28	2,65
	GAT(R(D))	1,35	1,30	2,64
TAA	TAA(D)	2,04	2,46	4,50
	TTA(RC(D))	2,03	2,44	4,47
	ATT(C(D))	2,46	2,04	4,50
	AAT(R(D))	2,44	2,03	4,47
AAC	AAC(D)	1,45	1,89	3,34
	GTT(RC(D))	1,42	1,90	3,32
	TTG(C(D))	1,89	1,45	3,34
	CAA(R(D))	1,90	1,42	3,32
AAG	AAG(D)	1,97	1,94	3,91
	CTT(RC(D))	1,98	1,92	3,89
	TTC(C(D))	1,94	1,97	3,91
	GAA(R(D))	1,92	1,98	3,89
ACA	ACA(D)	1,02	1,01	2,03
	TGT(RC(D))	1,01	1,02	2,03
	TGT(C(D))	1,01	1,02	2,03
	ACA(R(D))	1,02	1,01	2,03
ATA	ATA(D)	1,00	1,00	2,00
	TAT(RC(D))	1,00	1,00	2,00
	TAT(C(D))	1,00	1,00	2,00
	ATA(R(D))	1,00	1,00	2,00
AGA	AGA(D)	1,08	1,11	2,19
	TCT(RC(D))	1,11	1,08	2,19
	TCT(C(D))	1,11	1,08	2,19
	AGA(R(D))	1,08	1,11	2,19
AAA	AAA(D)	1,93	1,86	3,79
	TTT(RC(D))	1,86	1,93	3,79
	TTT(C(D))	1,86	1,93	3,79
	AAA(R(D))	1,93	1,86	3,79

Composition of DNA sequence in random chromosome 2

Base	No.of bases	%
A	58628	29,31%
C	41645	20,82%
G	40952	20,48%
T	58775	29,39%
N	0	0,00%
Sum	200000	100,00%

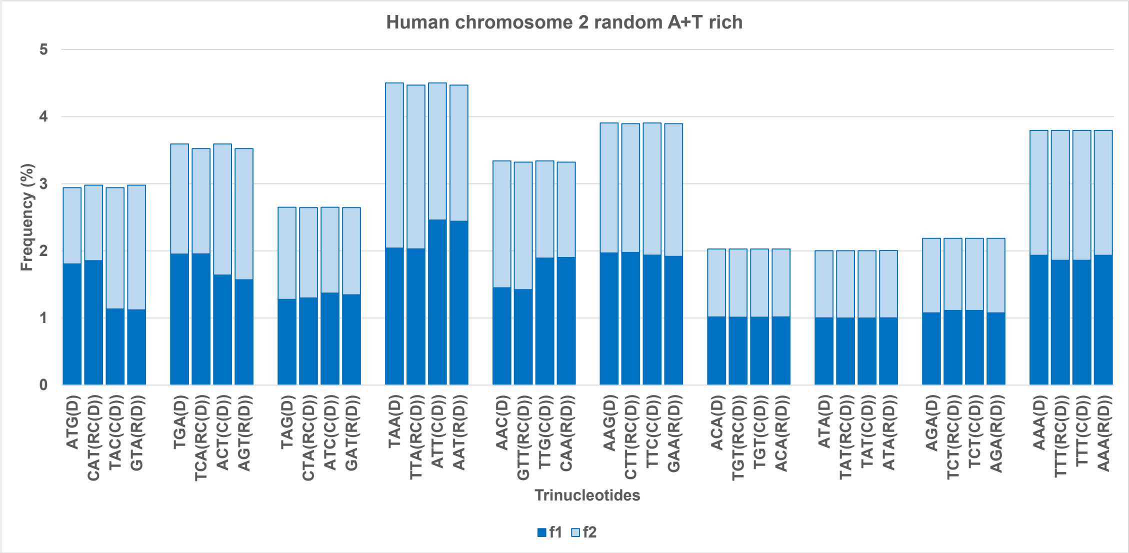

Human coding chromomome 2

A-T rich

D	f	%	RC	f	%	C	f	%	R	f	%	
**ATG**	249665,00	1,88	CAT	211830,00	1,60	TAC	138017,00	1,04	GTA	121758,00	0,92	Ia
**TGA**	275604,00	2,08	TCA	238383,00	1,80	ACT	197372,00	1,49	AGT	203115,00	1,53	
**TAG**	113804,00	0,86	CTA	134544,00	1,01	ATC	169208,00	1,28	GAT	192015,00	1,45	
**TAA**	183679,00	1,39	TTA	188501,00	1,42	ATT	241524,00	1,82	AAT	247249,00	1,86	Ib
**AAC**	190689,00	1,44	GTT	173418,00	1,31	TTG	232037,00	1,75	CAA	248116,00	1,87	
**AAG**	310456,00	2,34	CTT	235936,00	1,78	TTC	230340,00	1,74	GAA	316599,00	2,39	
**ACA**	244153,00	1,84	TGT	242302,00	1,83	TGT*			ACA*			Ic
**ATA**	174272,00	1,31	TAT	183575,00	1,38	TAT*			ATA*			
**AGA**	327290,00	2,47	TCT	239044,00	1,80	TCT*			AGA*			
**AAA**	405684,00	3,06	TTT	340924,00	2,57	TTT*			AAA*			

Quadruplet		f1	f2	fu
ATG	ATG(D)	1,88	1,04	2,92
	CAT(RC(D))	1,60	0,92	2,52
	TAC(C(D))	1,04	1,88	2,92
	GTA(R(D))	0,92	1,60	2,52
TGA	TGA(D)	2,08	1,49	3,57
	TCA(RC(D))	1,80	1,53	3,33
	ACT(C(D))	1,49	2,08	3,57
	AGT(R(D))	1,53	1,80	3,33
TAG	TAG(D)	0,86	1,28	2,13
	CTA(RC(D))	1,01	1,45	2,46
	ATC(C(D))	1,28	0,86	2,13
	GAT(R(D))	1,45	1,01	2,46
TAA	TAA(D)	1,39	1,82	3,21
	TTA(RC(D))	1,42	1,86	3,29
	ATT(C(D))	1,82	1,39	3,21
	AAT(R(D))	1,86	1,42	3,29
AAC	AAC(D)	1,44	1,75	3,19
	GTT(RC(D))	1,31	1,87	3,18
	TTG(C(D))	1,75	1,44	3,19
	CAA(R(D))	1,87	1,31	3,18
AAG	AAG(D)	2,34	1,74	4,08
	CTT(RC(D))	1,78	2,39	4,17
	TTC(C(D))	1,74	2,34	4,08
	GAA(R(D))	2,39	1,78	4,17
ACA	ACA(D)	0,92	0,91	1,83
	TGT(RC(D))	0,91	0,92	1,83
	TGT(C(D))	0,91	0,92	1,83
	ACA(R(D))	0,92	0,91	1,83
ATA	ATA(D)	0,66	0,69	1,35
	TAT(RC(D))	0,69	0,66	1,35
	TAT(C(D))	0,69	0,66	1,35
	ATA(R(D))	0,66	0,66	1,31
AGA	AGA(D)	1,23	0,90	2,14
	TCT(RC(D))	0,90	1,23	2,14
	TCT(C(D))	0,90	1,23	2,14
	AGA(R(D))	1,23	0,90	2,14
AAA	AAA(D)	1,53	1,29	2,82
	TTT(RC(D))	1,29	1,53	2,82
	TTT(C(D))	1,29	1,53	2,82
	AAA(R(D))	1,53	1,29	2,82

Composition of DNA sequence in coding chromosome 2

Base	No.of bases	%
A	3688330	27,82%
C	3023986	22,81%
G	3158909	23,82%
T	3388500	25,55%
N	0	0,00%
Sum	13259725	100,00%

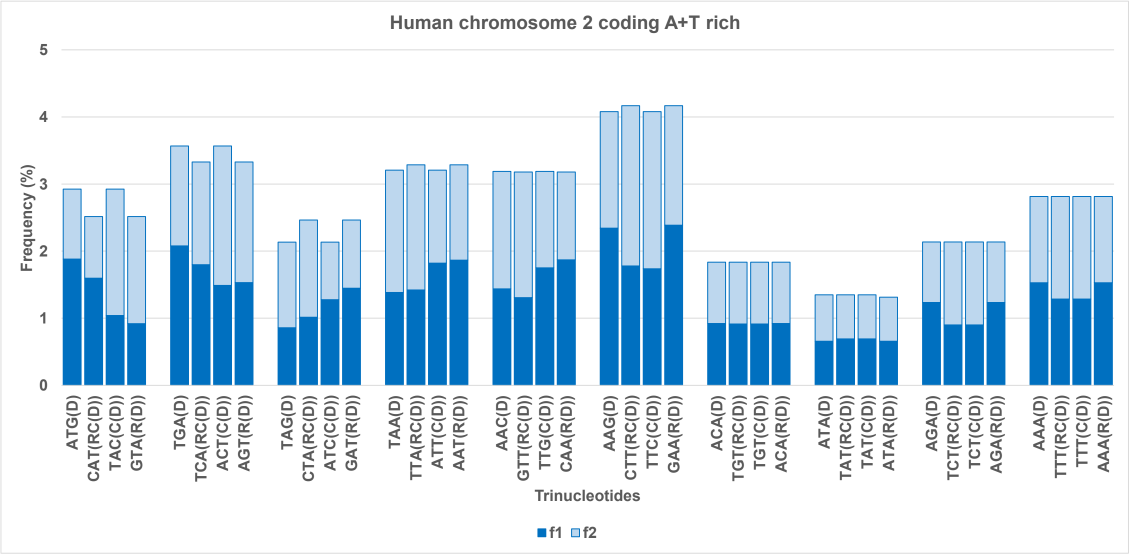

Human assembled chromomome 2

						C+G rich						
**D**	**f**	**%**	**RC**	**f**	**%**	**C**	**f**	**%**	**R**	**f**	**%**	
**CGT**	580544,00	0,24	ACG	578594,00	0,24	GCA	3432049,00	1,43	TGC	3415212,00	1,42	IIa
**GTC**	2243312,00	0,93	GAC	2238154,00	0,93	CAG	4786374,00	1,99	CTG	4750184,00	1,97	
**GCT**	3300818,00	1,37	AGC	3324050,00	1,38	CGA	495083,00	0,21	TCG	501563,00	0,21	
**GCC**	2706366,00	1,13	GGC	2703741,00	1,12	CGG	600010,00	0,25	CCG	591710,00	0,25	IIb
**CCA**	4297836,00	1,79	TGG	4337725,00	1,80	GGT	2725676,00	1,13	ACC	2713998,00	1,13	
**CCT**	4154410,00	1,73	AGG	4160006,00	1,73	GGA	3668322,00	1,52	TCC	3623593,00	1,51	
**CAC**	3525774,00	1,47	GTG	3555265,00	1,48	GTG*			CAC*			IIc
**CGC**	517032,00	0,21	GCG	520801,00	0,22	GCG*			CGC*			
**CTC**	3933579,00	1,64	GAG	3944289,00	1,64	GAG*			CTC*			
**CCC**	2997357,00	1,25	GGG	2991994,00	1,24	GGG*			CCC*			

Quadruplet		f1	f2	fu
CGT	CGT(D)	0,24	1,43	1,67
	ACG(RC(D))	0,24	1,42	1,66
	GCA(C(D))	1,43	0,24	1,67
	TGC(R(D))	1,42	0,24	1,66
GTC	GTC(D)	0,93	1,99	2,92
	GAC(RC(D))	0,93	1,97	2,91
	CAG(C(D))	1,99	0,93	2,92
	CTG(R(D))	1,97	0,93	2,91
GCT	GCT(D)	1,37	0,21	1,58
	AGC(RC(D))	1,38	0,21	1,59
	CGA(C(D))	0,21	1,37	1,58
	TCG(R(D))	0,21	1,38	1,59
GCC	GCC(D)	1,13	0,25	1,37
	GGC(RC(D))	1,12	0,25	1,37
	CGG(C(D))	0,25	1,13	1,37
	CCG(R(D))	0,25	1,12	1,37
CCA	CCA(D)	1,79	1,13	2,92
	TGG(RC(D))	1,80	1,13	2,93
	GGT(C(D))	1,13	1,79	2,92
	ACC(R(D))	1,13	1,80	2,93
CCT	CCT(D)	1,73	1,52	3,25
	AGG(RC(D))	1,73	1,51	3,24
	GGA(C(D))	1,52	1,73	3,25
	TCC(R(D))	1,51	1,73	3,24
CAC	CAC(D)	0,73	0,74	1,47
	GTG(RC(D))	0,74	0,73	1,47
	GTG(C(D))	0,74	0,73	1,47
	CAC(R(D))	0,73	0,74	1,47
CGC	CGC(D)	0,11	0,11	0,22
	GCG(RC(D))	0,11	0,11	0,22
	GCG(C(D))	0,11	0,11	0,22
	CGC(R(D))	0,11	0,11	0,22
CTC	CTC(D)	0,82	0,82	1,64
	GAG(RC(D))	0,82	0,82	1,64
	GAG(C(D))	0,82	0,82	1,64
	CTC(R(D))	0,82	0,82	1,64
CCC	CCC(D)	0,62	0,62	1,24
	GGG(RC(D))	0,62	0,62	1,24
	GGG(C(D))	0,62	0,62	1,24
	CCC(R(D))	0,62	0,62	1,24

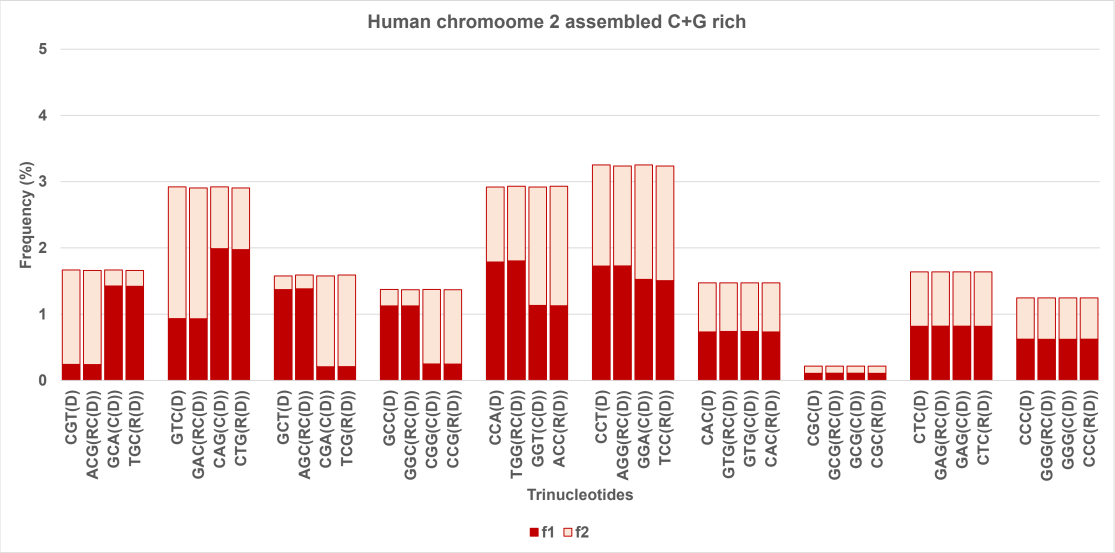

Human random chromomome 2

C+G rich												
**D**	**f**	**%**	**RC**	**f**	**%**	**C**	**f**	**%**	**R**	**f**	**%**	
**CGT**	554,00	0,28	ACG	538,00	0,27	GCA	2906,00	1,45	TGC	2995,00	1,50	IIa
**GTC**	1916,00	0,96	GAC	1948,00	0,97	CAG	4017,00	2,01	CTG	4182,00	2,09	
**GCT**	2821,00	1,41	AGC	2778,00	1,39	CGA	434,00	0,22	TCG	461,00	0,23	
**GCC**	2457,00	1,23	GGC	2410,00	1,21	CGG	585,00	0,29	CCG	575,00	0,29	IIb
**CCA**	3722,00	1,86	TGG	3727,00	1,86	GGT	2362,00	1,18	ACC	2307,00	1,15	
**CCT**	3707,00	1,85	AGG	3486,00	1,74	GGA	3026,00	1,51	TCC	3240,00	1,62	
**CAC**	3074,00	1,54	GTG	3092,00	1,55	GTG*			CAC*			IIc
**CGC**	501,00	0,25	GCG	500,00	0,25	GCG*			CGC*			
**CTC**	3521,00	1,76	GAG	3196,00	1,60	GAG*			CTC*			
**CCC**	2713,00	1,36	GGG	2707,00	1,35	GGG*			CCC*			

Quadruplet		f1	f2	fu
	CGT(D)	0,28	1,45	1,73
CGT	ACG(RC(D))	0,27	1,50	1,77
	GCA(C(D))	1,45	0,28	1,73
	TGC(R(D))	1,50	0,27	1,77
	GTC(D)	0,96	2,01	2,97
GTC	GAC(RC(D))	0,97	2,09	3,07
	CAG(C(D))	2,01	0,96	2,97
	CTG(R(D))	2,09	0,97	3,07
	GCT(D)	1,41	0,22	1,63
GCT	AGC(RC(D))	1,39	0,23	1,62
	CGA(C(D))	0,22	1,41	1,63
	TCG(R(D))	0,23	1,39	1,62
	GCC(D)	1,23	0,29	1,52
GCC	GGC(RC(D))	1,21	0,29	1,49
	CGG(C(D))	0,29	1,23	1,52
	CCG(R(D))	0,29	1,21	1,49
	CCA(D)	1,86	1,18	3,04
CCA	TGG(RC(D))	1,86	1,15	3,02
	GGT(C(D))	1,18	1,86	3,04
	ACC(R(D))	1,15	1,86	3,02
	CCT(D)	1,85	1,51	3,37
CCT	AGG(RC(D))	1,74	1,62	3,36
	GGA(C(D))	1,51	1,85	3,37
	TCC(R(D))	1,62	1,74	3,36
	CAC(D)	0,77	0,77	1,54
CAC	GTG(RC(D))	0,77	0,77	1,54
	GTG(C(D))	0,77	0,77	1,54
	CAC(R(D))	0,77	0,77	1,54
	CGC(D)	0,13	0,13	0,25
CGC	GCG(RC(D))	0,13	0,13	0,25
	GCG(C(D))	0,13	0,13	0,25
	CGC(R(D))	0,13	0,13	0,25
	CTC(D)	0,88	0,80	1,68
CTC	GAG(RC(D))	0,80	0,88	1,68
	GAG(C(D))	0,80	0,88	1,68
	CTC(R(D))	0,88	0,80	1,68
	CCC(D)	0,68	0,68	1,36
CCC	GGG(RC(D))	0,68	0,68	1,36
	GGG(C(D))	0,68	0,68	1,36
	CCC(R(D))	0,68	0,68	1,36

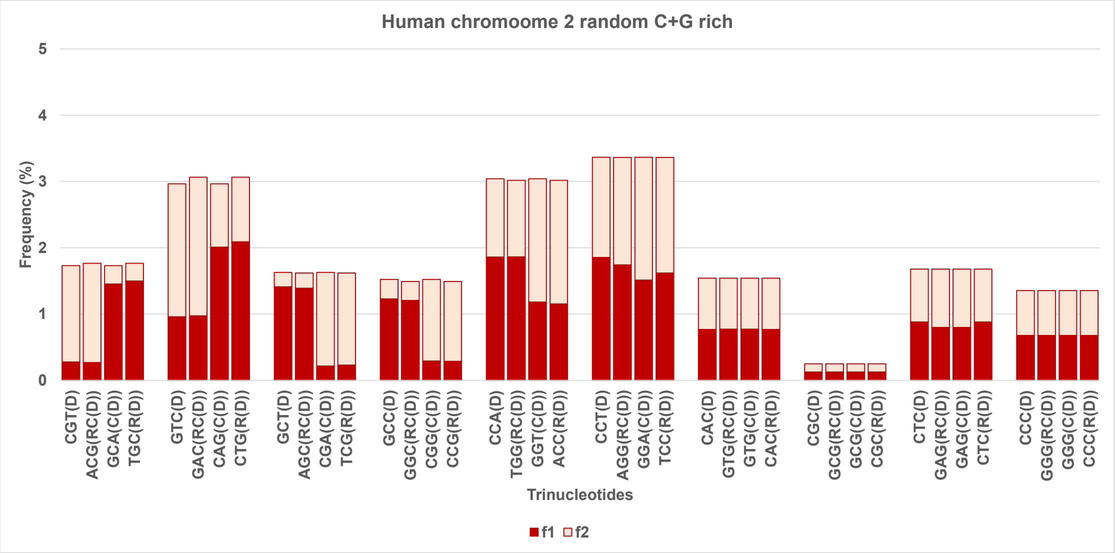

Human coding chromomome 2

C+G rich

D	f	%	RC	f	%	C	f	%	R	f	%	
**CGT**	58993,00	0,44	ACG	64763,00	0,49	GCA	221526,00	1,67	TGC	224643,00	1,69	IIa
**GTC**	135937,00	1,03	GAC	170384,00	1,28	CAG	323062,00	2,44	CTG	323202,00	2,44	
**GCT**	222291,00	1,68	AGC	237123,00	1,79	CGA	69856,00	0,53	TCG	59898,00	0,45	
**GCC**	220389,00	1,66	GGC	204716,00	1,54	CGG	95517,00	0,72	CCG	97430,00	0,73	IIb
**CCA**	270191,00	2,04	TGG	283983,00	2,14	GGT	148331,00	1,12	ACC	184047,00	1,39	
**CCT**	250581,00	1,89	AGG	241720,00	1,82	GGA	268173,00	2,02	TCC	213766,00	1,61	
**CAC**	191245,00	1,44	GTG	221628,00	1,67	GTG*			CAC*			IIc
**CGC**	83194,00	0,63	GCG	85470,00	0,64	GCG*			CGC*			
**CTC**	215606,00	1,63	GAG	261925,00	1,98	GAG*			CTC*			
**CCC**	214682,00	1,62	GGG	194348,00	1,47	GGG*			CCC*			

Quadruplet		f1	f2	fu
CGT	CGT(D)	0,44	1,67	2,12
	ACG(RC(D))	0,49	1,69	2,18
	GCA(C(D))	1,67	0,44	2,12
	TGC(R(D))	1,69	0,49	2,18
GTC	GTC(D)	1,03	2,44	3,46
	GAC(RC(D))	1,28	2,44	3,72
	CAG(C(D))	2,44	1,03	3,46
	CTG(R(D))	2,44	1,28	3,72
GCT	GCT(D)	1,68	0,53	2,20
	AGC(RC(D))	1,79	0,45	2,24
	CGA(C(D))	0,53	1,68	2,20
	TCG(R(D))	0,45	1,79	2,24
GCC	GCC(D)	1,66	0,72	2,38
	GGC(RC(D))	1,54	0,73	2,28
	CGG(C(D))	0,72	1,66	2,38
	CCG(R(D))	0,73	1,54	2,28
CCA	CCA(D)	2,04	1,12	3,16
	TGG(RC(D))	2,14	1,39	3,53
	GGT(C(D))	1,12	2,04	3,16
	ACC(R(D))	1,39	2,14	3,53
CCT	CCT(D)	1,89	2,02	3,91
	AGG(RC(D))	1,82	1,61	3,44
	GGA(C(D))	2,02	1,89	3,91
	TCC(R(D))	1,61	1,82	3,44
CAC	CAC(D)	0,72	0,84	1,56
	GTG(RC(D))	0,84	0,72	1,56
	GTG(C(D))	0,84	0,72	1,56
	CAC(R(D))	0,72	0,84	1,56
CGC	CGC(D)	0,31	0,32	0,64
	GCG(RC(D))	0,32	0,31	0,64
	GCG(C(D))	0,32	0,31	0,64
	CGC(R(D))	0,31	0,32	0,64
CTC	CTC(D)	0,81	0,99	1,80
	GAG(RC(D))	0,99	0,81	1,80
	GAG(C(D))	0,99	0,81	1,80
	CTC(R(D))	0,81	0,99	1,80
CCC	CCC(D)	0,81	0,73	1,54
	GGG(RC(D))	0,73	0,81	1,54
	GGG(C(D))	0,73	0,81	1,54
	CCC(R(D))	0,81	0,73	1,54

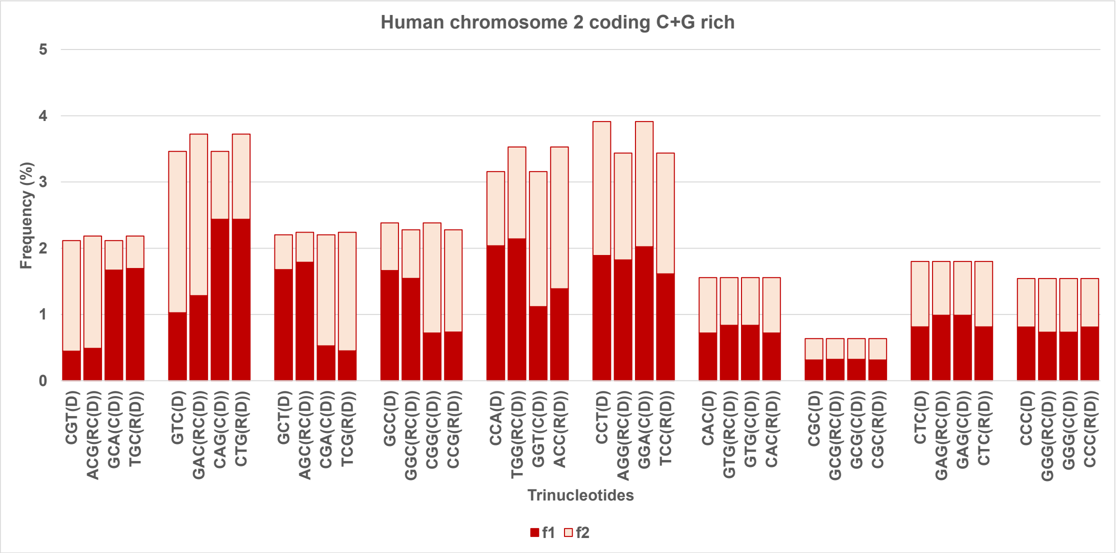

						A-T rich						
**D**	**f**	**%**	**RC**	**f**	**%**	**C**	**f**	**%**	**R**	**f**	**%**	
**ATG**	3687185,00	1,86	CAT	3687126,00	1,86	TAC	2316453,00	1,17	GTA	2338910,00	1,18	Ia
**TGA**	3915605,00	1,98	TCA	3940456,00	1,99	ACT	3261152,00	1,65	AGT	3267576,00	1,65	
**TAG**	2658975,00	1,34	CTA	2642160,00	1,33	ATC	2663071,00	1,34	GAT	2681109,00	1,35	
**TAA**	4339323,00	2,19	TTA	4335812,00	2,19	ATT	5193723,00	2,62	AAT	5173612,00	2,61	Ib
**AAC**	2989340,00	1,51	GTT	2977237,00	1,50	TTG	3872943,00	1,96	CAA	3829913,00	1,93	
**AAG**	4045631,00	2,04	CTT	4083610,00	2,06	TTC	4045812,00	2,04	GAA	4039346,00	2,04	
**ACA**	4030962,00	2,03	TGT	4055372,00	2,05	TGT*			ACA*			Ic
**ATA**	4327885,00	2,18	TAT	4330015,00	2,19	TAT*			ATA*			
**AGA**	4464341,00	2,25	TCT	4459517,00	2,25	TCT*			AGA*			
**AAA**	7897046,00	3,99	TTT	7973816,00	4,03	TTT*			AAA*			

Quadruplet		f1	f2	fu
ATG	ATG(D)	1,86	1,17	3,03
	CAT(RC(D))	1,86	1,18	3,04
	TAC(C(D))	1,17	1,86	3,03
	GTA(R(D))	1,18	1,86	3,04
TGA	TGA(D)	1,98	1,65	3,62
	TCA(RC(D))	1,99	1,65	3,64
	ACT(C(D))	1,65	1,98	3,62
	AGT(R(D))	1,65	1,99	3,64
TAG	TAG(D)	1,34	1,34	2,69
	CTA(RC(D))	1,33	1,35	2,69
	ATC(C(D))	1,34	1,34	2,69
	GAT(R(D))	1,35	1,33	2,69
TAA	TAA(D)	2,19	2,62	4,81
	TTA(RC(D))	2,19	2,61	4,80
	ATT(C(D))	2,62	2,19	4,81
	AAT(R(D))	2,61	2,19	4,80
AAC	AAC(D)	1,51	1,96	3,46
	GTT(RC(D))	1,50	1,93	3,44
	TTG(C(D))	1,96	1,51	3,46
	CAA(R(D))	1,93	1,50	3,44
AAG	AAG(D)	2,04	2,04	4,08
	CTT(RC(D))	2,06	2,04	4,10
	TTC(C(D))	2,04	2,04	4,08
	GAA(R(D))	2,04	2,06	4,10
ACA	ACA(D)	1,02	1,02	2,04
	TGT(RC(D))	1,02	1,02	2,04
	TGT(C(D))	1,02	1,02	2,04
	ACA(R(D))	1,02	1,02	2,04
ATA	ATA(D)	1,09	1,09	2,19
	TAT(RC(D))	1,09	1,09	2,19
	TAT(C(D))	1,09	1,09	2,19
	ATA(R(D))	1,09	1,09	2,18
AGA	AGA(D)	1,13	1,13	2,25
	TCT(RC(D))	1,13	1,13	2,25
	TCT(C(D))	1,13	1,13	2,25
	AGA(R(D))	1,13	1,13	2,25
AAA	AAA(D)	1,99	2,01	4,01
	TTT(RC(D))	2,01	1,99	4,01
	TTT(C(D))	2,01	1,99	4,01
	AAA(R(D))	1,99	2,01	4,01

Composition of DNA sequence in assembled chromosome 3

Base	No.of bases	%
A	59689091	30,10%
C	39233483	19,79%
G	39344259	19,84%
T	59833302	30,17%
N	195424	0,10%
Sum	198295559	100,00%

% of coding bases from complete assembled chromosome 1 (including N bases)

**5,32%**

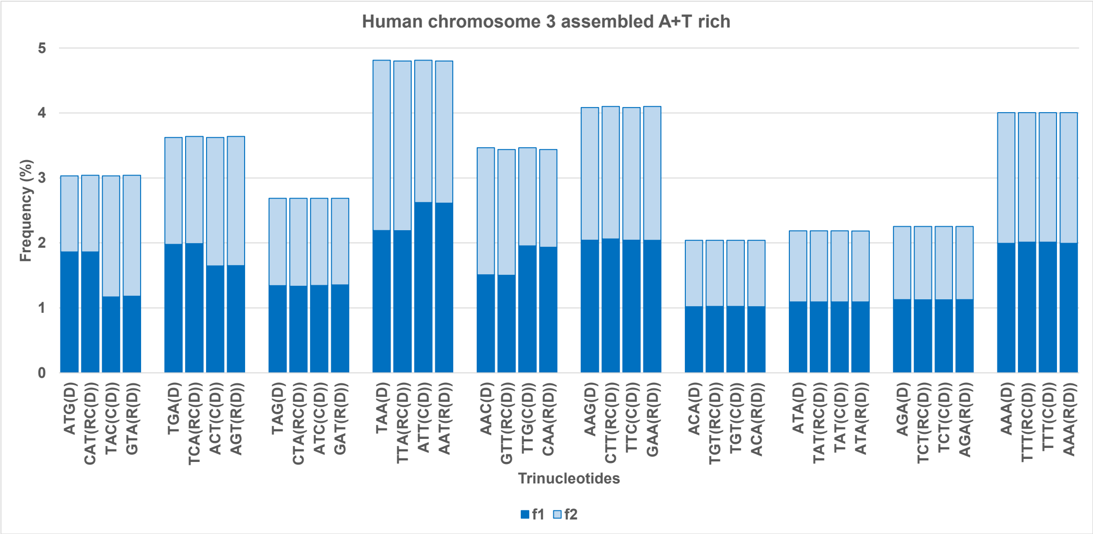

Human random chromomome 3

A-T rich

D	f	%	RC	f	%	C	f	%	R	f	%	
**ATG**	3673,00	1,84	CAT	3768,00	1,88	TAC	2286,00	1,14	GTA	2250,00	1,13	Ia
**TGA**	3945,00	1,97	TCA	3981,00	1,99	ACT	3248,00	1,62	AGT	3248,00	1,62	
**TAG**	2591,00	1,30	CTA	2677,00	1,34	ATC	2846,00	1,42	GAT	2606,00	1,30	
**TAA**	4150,00	2,08	TTA	4251,00	2,13	ATT	5042,00	2,52	AAT	5036,00	2,52	Ib
**AAC**	2892,00	1,45	GTT	2983,00	1,49	TTG	3847,00	1,92	CAA	3854,00	1,93	
**AAG**	4026,00	2,01	CTT	4061,00	2,03	TTC	3988,00	1,99	GAA	3950,00	1,98	
**ACA**	3898,00	1,95	TGT	4023,00	2,01	TGT*			ACA*			Ic
**ATA**	3998,00	2,00	TAT	4149,00	2,07	TAT*			ATA*			
**AGA**	4401,00	2,20	TCT	4497,00	2,25	TCT*			AGA*			
**AAA**	7494,00	3,75	TTT	7540,00	3,77	TTT*			AAA*			

Quadruplet		f1	f2	fu
ATG	ATG(D)	1,84	1,14	2,98
	CAT(RC(D))	1,88	1,13	3,01
	TAC(C(D))	1,14	1,84	2,98
	GTA(R(D))	1,13	1,88	3,01
TGA	TGA(D)	1,97	1,62	3,60
	TCA(RC(D))	1,99	1,62	3,61
	ACT(C(D))	1,62	1,97	3,60
	AGT(R(D))	1,62	1,99	3,61
TAG	TAG(D)	1,30	1,42	2,72
	CTA(RC(D))	1,34	1,30	2,64
	ATC(C(D))	1,42	1,30	2,72
	GAT(R(D))	1,30	1,34	2,64
TAA	TAA(D)	2,08	2,52	4,60
	TTA(RC(D))	2,13	2,52	4,64
	ATT(C(D))	2,52	2,08	4,60
	AAT(R(D))	2,52	2,13	4,64
AAC	AAC(D)	1,45	1,92	3,37
	GTT(RC(D))	1,49	1,93	3,42
	TTG(C(D))	1,92	1,45	3,37
	CAA(R(D))	1,93	1,49	3,42
AAG	AAG(D)	2,01	1,99	4,01
	CTT(RC(D))	2,03	1,98	4,01
	TTC(C(D))	1,99	2,01	4,01
	GAA(R(D))	1,98	2,03	4,01
ACA	ACA(D)	0,97	1,01	1,98
	TGT(RC(D))	1,01	0,97	1,98
	TGT(C(D))	1,01	0,97	1,98
	ACA(R(D))	0,97	1,01	1,98
ATA	ATA(D)	1,00	1,04	2,04
	TAT(RC(D))	1,04	1,00	2,04
	TAT(C(D))	1,04	1,00	2,04
	ATA(R(D))	1,00	1,00	2,00
AGA	AGA(D)	1,10	1,12	2,22
	TCT(RC(D))	1,12	1,10	2,22
	TCT(C(D))	1,12	1,10	2,22
	AGA(R(D))	1,10	1,12	2,22
AAA	AAA(D)	1,87	1,89	3,76
	TTT(RC(D))	1,89	1,87	3,76
	TTT(C(D))	1,89	1,87	3,76
	AAA(R(D))	1,87	1,89	3,76

Composition of DNA sequence in random chromosome 3

Base	No.of bases	%
A	58957	29,48%
C	41202	20,60%
G	40421	20,21%
T	59420	29,71%
N	0	0,00%
Sum	200000	100,00%

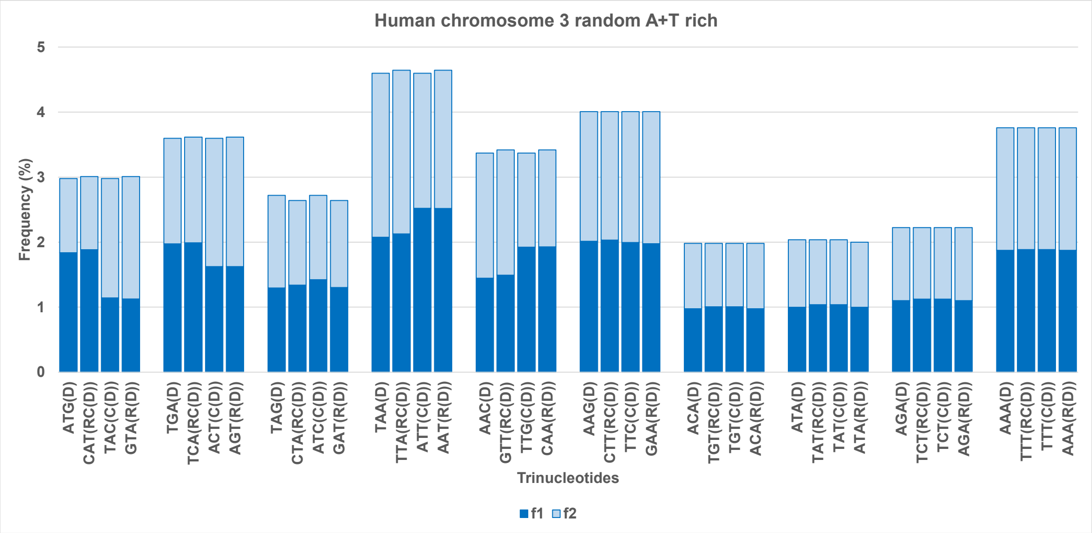

Human coding chromomome 3

A-T rich

D	f	%	RC	f	%	C	f	%	R	f	%	
**ATG**	195580,00	1,85	CAT	172058,00	1,63	TAC	105941,00	1,00	GTA	93093,00	0,88	Ia
**TGA**	212485,00	2,01	TCA	192255,00	1,82	ACT	155410,00	1,47	AGT	157994,00	1,50	
**TAG**	88823,00	0,84	CTA	106801,00	1,01	ATC	135925,00	1,29	GAT	146791,00	1,39	
**TAA**	135826,00	1,29	TTA	142801,00	1,35	ATT	182191,00	1,73	AAT	182742,00	1,73	Ib
**AAC**	147793,00	1,40	GTT	133077,00	1,26	TTG	180262,00	1,71	CAA	190941,00	1,81	
**AAG**	233822,00	2,21	CTT	190884,00	1,81	TTC	183089,00	1,73	GAA	237590,00	2,25	
**ACA**	191326,00	1,81	TGT	191889,00	1,82	TGT*			ACA*			Ic
**ATA**	128441,00	1,22	TAT	140546,00	1,33	TAT*			ATA*			
**AGA**	250038,00	2,37	TCT	191234,00	1,81	TCT*			AGA*			
**AAA**	295393,00	2,80	TTT	269641,00	2,55	TTT*			AAA*			

Quadruplet		f1	f2	fu
ATG	ATG(D)	1,85	1,00	2,86
	CAT(RC(D))	1,63	0,88	2,51
	TAC(C(D))	1,00	1,85	2,86
	GTA(R(D))	0,88	1,63	2,51
TGA	TGA(D)	2,01	1,47	3,48
	TCA(RC(D))	1,82	1,50	3,32
	ACT(C(D))	1,47	2,01	3,48
	AGT(R(D))	1,50	1,82	3,32
TAG	TAG(D)	0,84	1,29	2,13
	CTA(RC(D))	1,01	1,39	2,40
	ATC(C(D))	1,29	0,84	2,13
	GAT(R(D))	1,39	1,01	2,40
TAA	TAA(D)	1,29	1,73	3,01
	TTA(RC(D))	1,35	1,73	3,08
	ATT(C(D))	1,73	1,29	3,01
	AAT(R(D))	1,73	1,35	3,08
AAC	AAC(D)	1,40	1,71	3,11
	GTT(RC(D))	1,26	1,81	3,07
	TTG(C(D))	1,71	1,40	3,11
	CAA(R(D))	1,81	1,26	3,07
AAG	AAG(D)	2,21	1,73	3,95
	CTT(RC(D))	1,81	2,25	4,06
	TTC(C(D))	1,73	2,21	3,95
	GAA(R(D))	2,25	1,81	4,06
ACA	ACA(D)	0,91	0,91	1,81
	TGT(RC(D))	0,91	0,91	1,81
	TGT(C(D))	0,91	0,91	1,81
	ACA(R(D))	0,91	0,91	1,81
ATA	ATA(D)	0,61	0,67	1,27
	TAT(RC(D))	0,67	0,61	1,27
	TAT(C(D))	0,67	0,61	1,27
	ATA(R(D))	0,61	0,61	1,22
AGA	AGA(D)	1,18	0,91	2,09
	TCT(RC(D))	0,91	1,18	2,09
	TCT(C(D))	0,91	1,18	2,09
	AGA(R(D))	1,18	0,91	2,09
AAA	AAA(D)	1,40	1,28	2,68
	TTT(RC(D))	1,28	1,40	2,68
	TTT(C(D))	1,28	1,40	2,68
	AAA(R(D))	1,40	1,28	2,68

Composition of DNA sequence in coding chromosome 3

Base	No.of bases	%
A	2853802	27,03%
C	2477993	23,47%
G	2550327	24,15%
T	2676904	25,35%
N	0	0,00%
Sum	10559026	100,00%

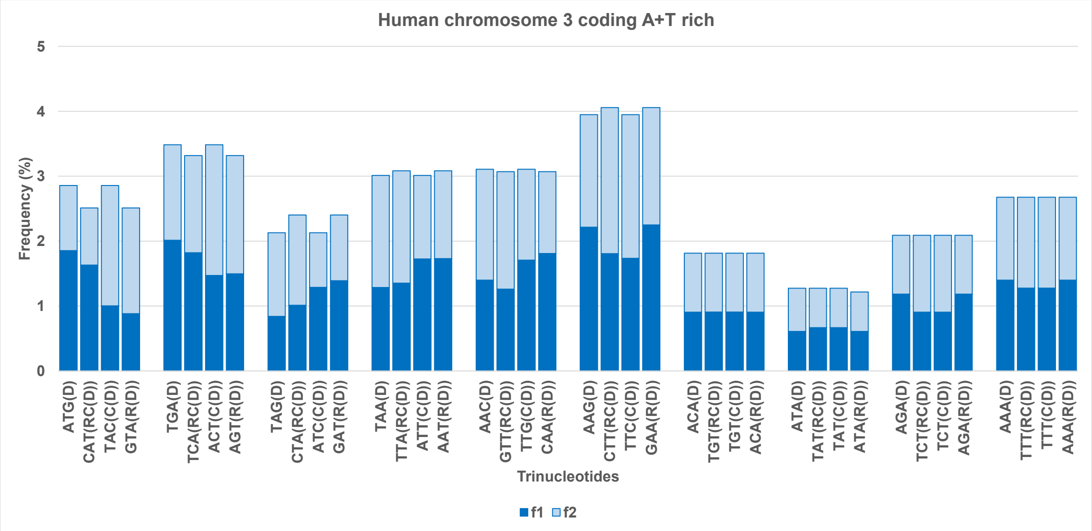

C+G rich												
**D**	**f**	**%**	**RC**	**f**	**%**	**C**	**f**	**%**	**R**	**f**	**%**	
**CGT**	456761,00	0,23	ACG	454381,00	0,23	GCA	2765835,00	1,40	TGC	2762407,00	1,39	IIa
**GTC**	1807639,00	0,91	GAC	1819863,00	0,92	CAG	3859694,00	1,95	CTG	3815695,00	1,93	
**GCT**	2659504,00	1,34	AGC	2666163,00	1,35	CGA	383345,00	0,19	TCG	395507,00	0,20	
**GCC**	2138195,00	1,08	GGC	2138261,00	1,08	CGG	450706,00	0,23	CCG	437626,00	0,22	IIb
**CCA**	3476623,00	1,75	TGG	3515174,00	1,77	GGT	2216810,00	1,12	ACC	2216301,00	1,12	
**CCT**	3356344,00	1,69	AGG	3350704,00	1,69	GGA	2961514,00	1,49	TCC	2916095,00	1,47	
**CAC**	2837142,00	1,43	GTG	2872733,00	1,45	GTG*			CAC*			IIc
**CGC**	382481,00	0,19	GCG	385779,00	0,19	GCG*			CGC*			
**CTC**	3195052,00	1,61	GAG	3184482,00	1,61	GAG*			CTC*			
**CCC**	2339195,00	1,18	GGG	2357034,00	1,19	GGG*			CCC*			

Quadruplet		f1	f2	fu
CGT	CGT(D)	0,23	1,40	1,63
	ACG(RC(D))	0,23	1,39	1,62
	GCA(C(D))	1,40	0,23	1,63
	TGC(R(D))	1,39	0,23	1,62
GTC	GTC(D)	0,91	1,95	2,86
	GAC(RC(D))	0,92	1,93	2,84
	CAG(C(D))	1,95	0,91	2,86
	CTG(R(D))	1,93	0,92	2,84
GCT	GCT(D)	1,34	0,19	1,54
	AGC(RC(D))	1,35	0,20	1,55
	CGA(C(D))	0,19	1,34	1,54
	TCG(R(D))	0,20	1,35	1,55
GCC	GCC(D)	1,08	0,23	1,31
	GGC(RC(D))	1,08	0,22	1,30
	CGG(C(D))	0,23	1,08	1,31
	CCG(R(D))	0,22	1,08	1,30
CCA	CCA(D)	1,75	1,12	2,87
	TGG(RC(D))	1,77	1,12	2,89
	GGT(C(D))	1,12	1,75	2,87
	ACC(R(D))	1,12	1,77	2,89
CCT	CCT(D)	1,69	1,49	3,19
	AGG(RC(D))	1,69	1,47	3,16
	GGA(C(D))	1,49	1,69	3,19
	TCC(R(D))	1,47	1,69	3,16
CAC	CAC(D)	0,72	0,73	1,44
	GTG(RC(D))	0,73	0,72	1,44
	GTG(C(D))	0,73	0,72	1,44
	CAC(R(D))	0,72	0,73	1,44
CGC	CGC(D)	0,10	0,10	0,19
	GCG(RC(D))	0,10	0,10	0,19
	GCG(C(D))	0,10	0,10	0,19
	CGC(R(D))	0,10	0,10	0,19
CTC	CTC(D)	0,81	0,80	1,61
	GAG(RC(D))	0,80	0,81	1,61
	GAG(C(D))	0,80	0,81	1,61
	CTC(R(D))	0,81	0,80	1,61
CCC	CCC(D)	0,59	0,59	1,19
	GGG(RC(D))	0,59	0,59	1,19
	GGG(C(D))	0,59	0,59	1,19
	CCC(R(D))	0,59	0,59	1,19

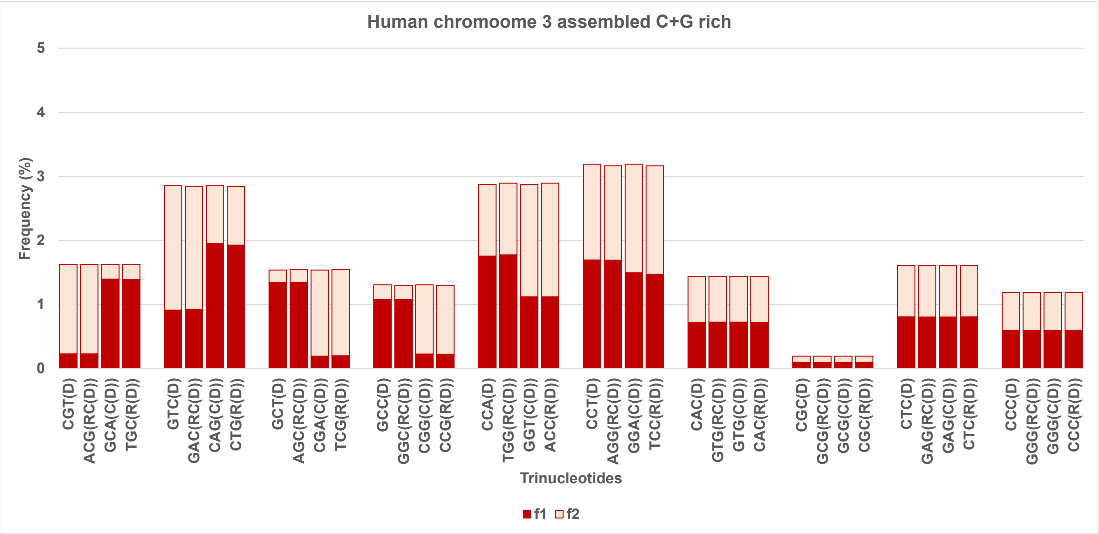

Human random chromomome 3

C+G rich

D	f	%	RC	f	%	C	f	%	R	f	%	
**CGT**	465,00	0,23	ACG	505,00	0,25	GCA	2850,00	1,43	TGC	2877,00	1,44	IIa
**GTC**	1899,00	0,95	GAC	1877,00	0,94	CAG	4034,00	2,02	CTG	4123,00	2,06	
**GCT**	2840,00	1,42	AGC	2816,00	1,41	CGA	376,00	0,19	TCG	406,00	0,20	
**GCC**	2404,00	1,20	GGC	2363,00	1,18	CGG	486,00	0,24	CCG	454,00	0,23	IIb
**CCA**	3878,00	1,94	TGG	3691,00	1,85	GGT	2289,00	1,14	ACC	2354,00	1,18	
**CCT**	3624,00	1,81	AGG	3479,00	1,74	GGA	3004,00	1,50	TCC	3198,00	1,60	
**CAC**	2951,00	1,48	GTG	2893,00	1,45	GTG*			CAC*			IIc
**CGC**	404,00	0,20	GCG	366,00	0,18	GCG*			CGC*			
**CTC**	3348,00	1,67	GAG	3293,00	1,65	GAG*			CTC*			
**CCC**	2698,00	1,35	GGG	2554,00	1,28	GGG*			CCC*			

Quadruplet		f1	f2	fu
CGT	CGT(D)	0,23	1,43	1,66
	ACG(RC(D))	0,25	1,44	1,69
	GCA(C(D))	1,43	0,23	1,66
	TGC(R(D))	1,44	0,25	1,69
GTC	GTC(D)	0,95	2,02	2,97
	GAC(RC(D))	0,94	2,06	3,00
	CAG(C(D))	2,02	0,95	2,97
	CTG(R(D))	2,06	0,94	3,00
GCT	GCT(D)	1,42	0,19	1,61
	AGC(RC(D))	1,41	0,20	1,61
	CGA(C(D))	0,19	1,42	1,61
	TCG(R(D))	0,20	1,41	1,61
GCC	GCC(D)	1,20	0,24	1,45
	GGC(RC(D))	1,18	0,23	1,41
	CGG(C(D))	0,24	1,20	1,45
	CCG(R(D))	0,23	1,18	1,41
CCA	CCA(D)	1,94	1,14	3,08
	TGG(RC(D))	1,85	1,18	3,02
	GGT(C(D))	1,14	1,94	3,08
	ACC(R(D))	1,18	1,85	3,02
CCT	CCT(D)	1,81	1,50	3,31
	AGG(RC(D))	1,74	1,60	3,34
	GGA(C(D))	1,50	1,81	3,31
	TCC(R(D))	1,60	1,74	3,34
CAC	CAC(D)	0,74	0,72	1,46
	GTG(RC(D))	0,72	0,74	1,46
	GTG(C(D))	0,72	0,74	1,46
	CAC(R(D))	0,74	0,72	1,46
CGC	CGC(D)	0,10	0,09	0,19
	GCG(RC(D))	0,09	0,10	0,19
	GCG(C(D))	0,09	0,10	0,19
	CGC(R(D))	0,10	0,09	0,19
CTC	CTC(D)	0,84	0,82	1,66
	GAG(RC(D))	0,82	0,84	1,66
	GAG(C(D))	0,82	0,84	1,66
	CTC(R(D))	0,84	0,82	1,66
CCC	CCC(D)	0,67	0,64	1,31
	GGG(RC(D))	0,64	0,67	1,31
	GGG(C(D))	0,64	0,67	1,31
	CCC(R(D))	0,67	0,64	1,31

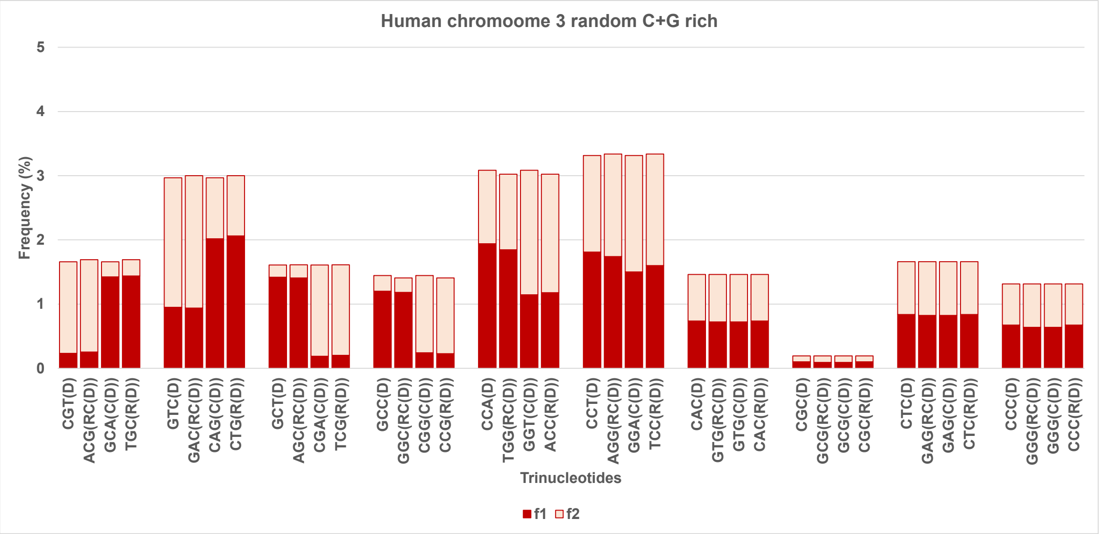

Human coding chromomome 3

C+G rich												
**D**	**f**	**%**	**RC**	**f**	**%**	**C**	**f**	**%**	**R**	**f**	**%**	
**CGT**	47175,00	0,45	ACG	51383,00	0,49	GCA	182854,00	1,73	TGC	186039,00	1,76	IIa
**GTC**	111237,00	1,05	GAC	135566,00	1,28	CAG	267624,00	2,53	CTG	266075,00	2,52	
**GCT**	183030,00	1,73	AGC	195416,00	1,85	CGA	54009,00	0,51	TCG	49191,00	0,47	
**GCC**	188416,00	1,78	GGC	174127,00	1,65	CGG	79566,00	0,75	CCG	78894,00	0,75	IIb
**CCA**	223947,00	2,12	TGG	231889,00	2,20	GGT	120734,00	1,14	ACC	150940,00	1,43	
**CCT**	211508,00	2,00	AGG	199407,00	1,89	GGA	216001,00	2,05	TCC	174993,00	1,66	
**CAC**	159759,00	1,51	GTG	180385,00	1,71	GTG*			CAC*			IIc
**CGC**	68277,00	0,65	GCG	69559,00	0,66	GCG*			CGC*			
**CTC**	177422,00	1,68	GAG	212585,00	2,01	GAG*			CTC*			
**CCC**	183053,00	1,73	GGG	165281,00	1,57	GGG*			CCC*			

Quadruplet		f1	f2	fu
CGT	CGT(D)	0,45	1,73	2,18
	ACG(RC(D))	0,49	1,76	2,25
	GCA(C(D))	1,73	0,45	2,18
	TGC(R(D))	1,76	0,49	2,25
GTC	GTC(D)	1,05	2,53	3,59
	GAC(RC(D))	1,28	2,52	3,80
	CAG(C(D))	2,53	1,05	3,59
	CTG(R(D))	2,52	1,28	3,80
GCT	GCT(D)	1,73	0,51	2,24
	AGC(RC(D))	1,85	0,47	2,32
	CGA(C(D))	0,51	1,73	2,24
	TCG(R(D))	0,47	1,85	2,32
GCC	GCC(D)	1,78	0,75	2,54
	GGC(RC(D))	1,65	0,75	2,40
	CGG(C(D))	0,75	1,78	2,54
	CCG(R(D))	0,75	1,65	2,40
CCA	CCA(D)	2,12	1,14	3,26
	TGG(RC(D))	2,20	1,43	3,63
	GGT(C(D))	1,14	2,12	3,26
	ACC(R(D))	1,43	2,20	3,63
CCT	CCT(D)	2,00	2,05	4,05
	AGG(RC(D))	1,89	1,66	3,55
	GGA(C(D))	2,05	2,00	4,05
	TCC(R(D))	1,66	1,89	3,55
CAC	CAC(D)	0,76	0,85	1,61
	GTG(RC(D))	0,85	0,76	1,61
	GTG(C(D))	0,85	0,76	1,61
	CAC(R(D))	0,76	0,85	1,61
CGC	CGC(D)	0,32	0,33	0,65
	GCG(RC(D))	0,33	0,32	0,65
	GCG(C(D))	0,33	0,32	0,65
	CGC(R(D))	0,32	0,33	0,65
CTC	CTC(D)	0,84	1,01	1,85
	GAG(RC(D))	1,01	0,84	1,85
	GAG(C(D))	1,01	0,84	1,85
	CTC(R(D))	0,84	1,01	1,85
CCC	CCC(D)	0,87	0,78	1,65
	GGG(RC(D))	0,78	0,87	1,65
	GGG(C(D))	0,78	0,87	1,65
	CCC(R(D))	0,87	0,78	1,65

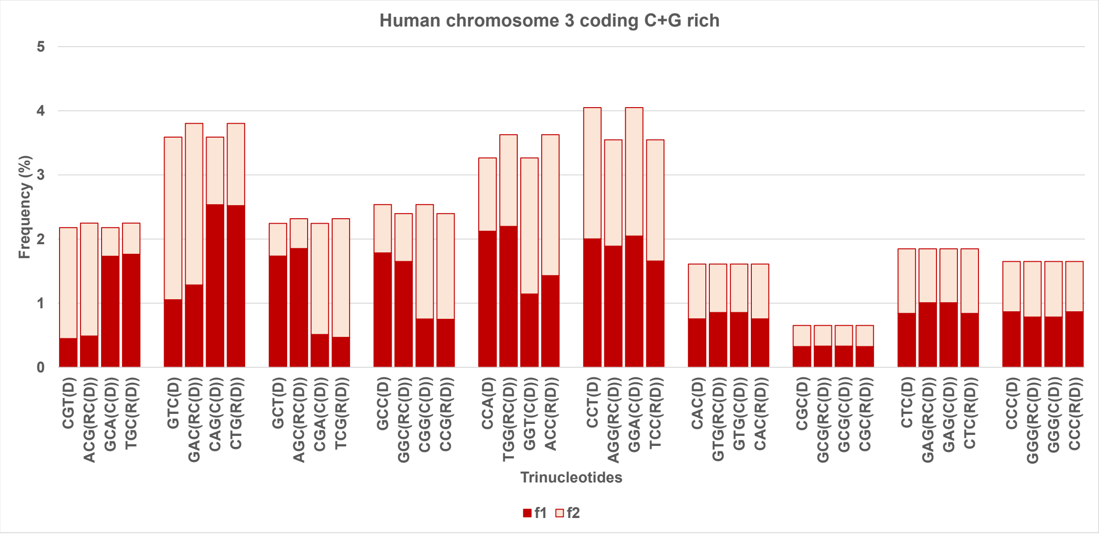

A-T rich												
**D**	**f**	**%**	**RC**	**f**	**%**	**C**	**f**	**%**	**R**	**f**	**%**	
**ATG**	3622387,00	1,91	CAT	3650677,00	1,92	TAC	2295105,00	1,21	GTA	2300660,00	1,21	Ia
**TGA**	3786364,00	2,00	TCA	3782217,00	1,99	ACT	3129985,00	1,65	AGT	3122541,00	1,65	
**TAG**	2596929,00	1,37	CTA	2581842,00	1,36	ATC	2600475,00	1,37	GAT	2612589,00	1,38	
**TAA**	4491471,00	2,37	TTA	4487185,00	2,36	ATT	5367888,00	2,83	AAT	5341300,00	2,81	Ib
**AAC**	2908054,00	1,53	GTT	2899239,00	1,53	TTG	3763645,00	1,98	CAA	3698267,00	1,95	
**AAG**	3885927,00	2,05	CTT	3908801,00	2,06	TTC	3925099,00	2,07	GAA	3945542,00	2,08	
**ACA**	3934996,00	2,07	TGT	3943338,00	2,08	TGT*			ACA*			Ic
**ATA**	4577129,00	2,41	TAT	4563311,00	2,40	TAT*			ATA*			
**AGA**	4262154,00	2,25	TCT	4257160,00	2,24	TCT*			AGA*			
**AAA**	7960585,00	4,20	TTT	7980275,00	4,21	TTT*			AAA*			

Quadruplet		f1	f2	fu
ATG	ATG(D)	1,91	1,21	3,12
	CAT(RC(D))	1,92	1,21	3,14
	TAC(C(D))	1,21	1,91	3,12
	GTA(R(D))	1,21	1,92	3,14
TGA	TGA(D)	2,00	1,65	3,64
	TCA(RC(D))	1,99	1,65	3,64
	ACT(C(D))	1,65	2,00	3,64
	AGT(R(D))	1,65	1,99	3,64
TAG	TAG(D)	1,37	1,37	2,74
	CTA(RC(D))	1,36	1,38	2,74
	ATC(C(D))	1,37	1,37	2,74
	GAT(R(D))	1,38	1,36	2,74
TAA	TAA(D)	2,37	2,83	5,20
	TTA(RC(D))	2,36	2,81	5,18
	ATT(C(D))	2,83	2,37	5,20
	AAT(R(D))	2,81	2,36	5,18
AAC	AAC(D)	1,53	1,98	3,52
	GTT(RC(D))	1,53	1,95	3,48
	TTG(C(D))	1,98	1,53	3,52
	CAA(R(D))	1,95	1,53	3,48
AAG	AAG(D)	2,05	2,07	4,12
	CTT(RC(D))	2,06	2,08	4,14
	TTC(C(D))	2,07	2,05	4,12
	GAA(R(D))	2,08	2,06	4,14
ACA	ACA(D)	1,04	1,04	2,08
	TGT(RC(D))	1,04	1,04	2,08
	TGT(C(D))	1,04	1,04	2,08
	ACA(R(D))	1,04	1,04	2,08
ATA	ATA(D)	1,21	1,20	2,41
	TAT(RC(D))	1,20	1,21	2,41
	TAT(C(D))	1,20	1,21	2,41
	ATA(R(D))	1,21	1,21	2,41
AGA	AGA(D)	1,12	1,12	2,24
	TCT(RC(D))	1,12	1,12	2,24
	TCT(C(D))	1,12	1,12	2,24
	AGA(R(D))	1,12	1,12	2,24
AAA	AAA(D)	2,10	2,10	4,20
	TTT(RC(D))	2,10	2,10	4,20
	TTT(C(D))	2,10	2,10	4,20
	AAA(R(D))	2,10	2,10	4,20

Composition of DNA sequence in assembled chromosome 4

Base	No.of bases	%
A	58561236	30,79%
C	36236976	19,05%
G	36331025	19,10%
T	58623430	30,82%
N	461888	0,24%
Ukupno:	190214555	100,00%

% of coding bases from complete assembled chromosome 1 (including N bases)

**3,82%**

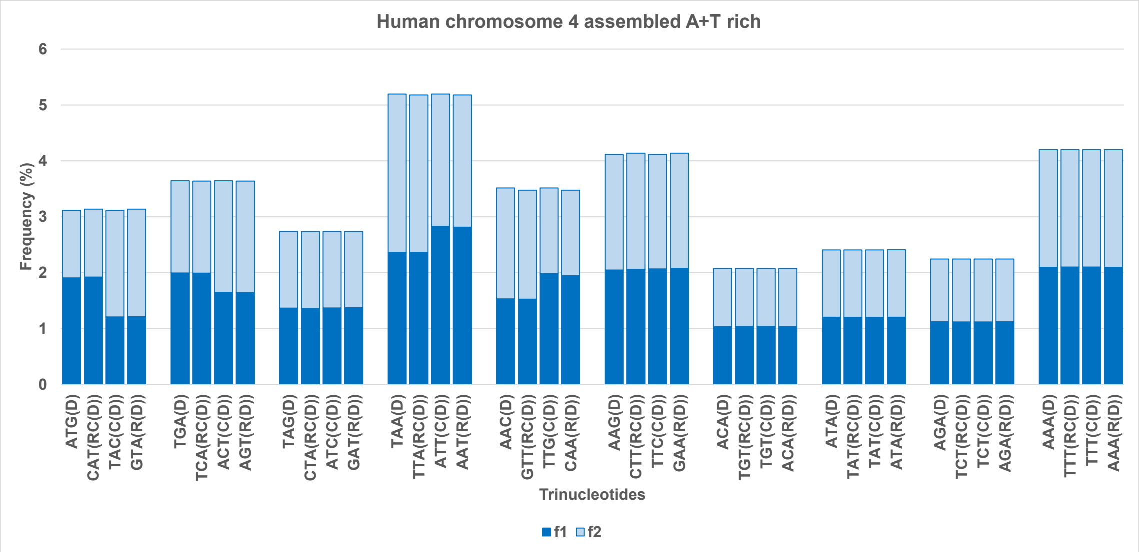

Human random chromomome 4

A-T rich

D	f	%	RC	f	%	C	f	%	R	f	%	
**ATG**	3793,00	1,91	CAT	3813,00	1,92	TAC	2369,00	1,19	GTA	2338,00	1,18	Ia
**TGA**	3981,00	2,00	TCA	3842,00	1,93	ACT	3249,00	1,63	AGT	3204,00	1,61	
**TAG**	2579,00	1,30	CTA	2701,00	1,36	ATC	2790,00	1,40	GAT	2764,00	1,39	
**TAA**	4475,00	2,25	TTA	4400,00	2,21	ATT	5347,00	2,69	AAT	5338,00	2,69	Ib
**AAC**	3104,00	1,56	GTT	2943,00	1,48	TTG	3903,00	1,96	CAA	3873,00	1,95	
**AAG**	3894,00	1,96	CTT	3975,00	2,00	TTC	3962,00	1,99	GAA	3988,00	2,01	
**ACA**	4207,00	2,12	TGT	4242,00	2,13	TGT*			ACA*			Ic
**ATA**	4640,00	2,33	TAT	4656,00	2,34	TAT*			ATA*			
**AGA**	4330,00	2,18	TCT	4443,00	2,24	TCT*			AGA*			
**AAA**	7873,00	3,96	TTT	7857,00	3,95	TTT*			AAA*			

Quadruplet		f1	f2	fu
ATG	ATG(D)	1,91	1,19	3,10
	CAT(RC(D))	1,92	1,18	3,09
	TAC(C(D))	1,19	1,91	3,10
	GTA(R(D))	1,18	1,92	3,09
TGA	TGA(D)	2,00	1,63	3,64
	TCA(RC(D))	1,93	1,61	3,54
	ACT(C(D))	1,63	2,00	3,64
	AGT(R(D))	1,61	1,93	3,54
TAG	TAG(D)	1,30	1,40	2,70
	CTA(RC(D))	1,36	1,39	2,75
	ATC(C(D))	1,40	1,30	2,70
	GAT(R(D))	1,39	1,36	2,75
TAA	TAA(D)	2,25	2,69	4,94
	TTA(RC(D))	2,21	2,69	4,90
	ATT(C(D))	2,69	2,25	4,94
	AAT(R(D))	2,69	2,21	4,90
AAC	AAC(D)	1,56	1,96	3,52
	GTT(RC(D))	1,48	1,95	3,43
	TTG(C(D))	1,96	1,56	3,52
	CAA(R(D))	1,95	1,48	3,43
AAG	AAG(D)	1,96	1,99	3,95
	CTT(RC(D))	2,00	2,01	4,01
	TTC(C(D))	1,99	1,96	3,95
	GAA(R(D))	2,01	2,00	4,01
ACA	ACA(D)	1,06	1,07	2,13
	TGT(RC(D))	1,07	1,06	2,13
	TGT(C(D))	1,07	1,06	2,13
	ACA(R(D))	1,06	1,07	2,13
ATA	ATA(D)	1,17	1,17	2,34
	TAT(RC(D))	1,17	1,17	2,34
	TAT(C(D))	1,17	1,17	2,34
	ATA(R(D))	1,17	1,17	2,33
AGA	AGA(D)	1,09	1,12	2,21
	TCT(RC(D))	1,12	1,09	2,21
	TCT(C(D))	1,12	1,09	2,21
	AGA(R(D))	1,09	1,12	2,21
AAA	AAA(D)	1,98	1,98	3,96
	TTT(RC(D))	1,98	1,98	3,96
	TTT(C(D))	1,98	1,98	3,96
	AAA(R(D))	1,98	1,98	3,96

Composition of DNA sequence in random chromosome 4

Base	No.of bases	%
A	60214	30,11%
C	39278	19,64%
G	38963	19,48%
T	60345	30,17%
N	1200	0,60%
Sum	200000	100,00%

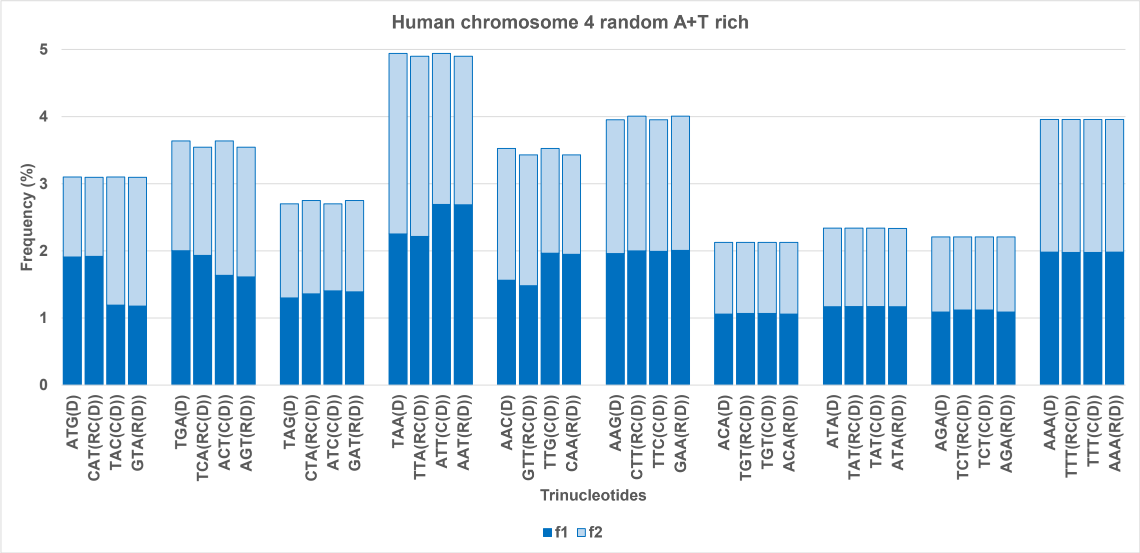

Human coding chromomome 4

A-T rich

D	f	%	RC	f	%	C	f	%	R	f	%	
**ATG**	142809,00	1,97	CAT	120013,00	1,65	TAC	77963,00	1,07	GTA	70772,00	0,97	Ia
**TGA**	150749,00	2,07	TCA	133774,00	1,84	ACT	110152,00	1,52	AGT	113921,00	1,57	
**TAG**	67038,00	0,92	CTA	75962,00	1,05	ATC	93862,00	1,29	GAT	108173,00	1,49	
**TAA**	113306,00	1,56	TTA	118902,00	1,64	ATT	149387,00	2,06	AAT	150543,00	2,07	Ib
**AAC**	109064,00	1,50	GTT	100570,00	1,38	TTG	133892,00	1,84	CAA	138365,00	1,90	
**AAG**	168656,00	2,32	CTT	132815,00	1,83	TTC	129978,00	1,79	GAA	176593,00	2,43	
**ACA**	139185,00	1,92	TGT	140418,00	1,93	TGT*			ACA*			Ic
**ATA**	108218,00	1,49	TAT	115547,00	1,59	TAT*			ATA*			
**AGA**	177550,00	2,44	TCT	131997,00	1,82	TCT*			AGA*			
**AAA**	241493,00	3,32	TTT	215358,00	2,96	TTT*			AAA*			

Quadruplet		f1	f2	fu
ATG	ATG(D)	1,97	1,07	3,04
	CAT(RC(D))	1,65	0,97	2,63
	TAC(C(D))	1,07	1,97	3,04
	GTA(R(D))	0,97	1,65	2,63
TGA	TGA(D)	2,07	1,52	3,59
	TCA(RC(D))	1,84	1,57	3,41
	ACT(C(D))	1,52	2,07	3,59
	AGT(R(D))	1,57	1,84	3,41
TAG	TAG(D)	0,92	1,29	2,21
	CTA(RC(D))	1,05	1,49	2,53
	ATC(C(D))	1,29	0,92	2,21
	GAT(R(D))	1,49	1,05	2,53
TAA	TAA(D)	1,56	2,06	3,61
	TTA(RC(D))	1,64	2,07	3,71
	ATT(C(D))	2,06	1,56	3,61
	AAT(R(D))	2,07	1,64	3,71
AAC	AAC(D)	1,50	1,84	3,34
	GTT(RC(D))	1,38	1,90	3,29
	TTG(C(D))	1,84	1,50	3,34
	CAA(R(D))	1,90	1,38	3,29
AAG	AAG(D)	2,32	1,79	4,11
	CTT(RC(D))	1,83	2,43	4,26
	TTC(C(D))	1,79	2,32	4,11
	GAA(R(D))	2,43	1,83	4,26
ACA	ACA(D)	0,96	0,97	1,92
	TGT(RC(D))	0,97	0,96	1,92
	TGT(C(D))	0,97	0,96	1,92
	ACA(R(D))	0,96	0,97	1,92
ATA	ATA(D)	0,74	0,80	1,54
	TAT(RC(D))	0,80	0,74	1,54
	TAT(C(D))	0,80	0,74	1,54
	ATA(R(D))	0,74	0,74	1,49
AGA	AGA(D)	1,22	0,91	2,13
	TCT(RC(D))	0,91	1,22	2,13
	TCT(C(D))	0,91	1,22	2,13
	AGA(R(D))	1,22	0,91	2,13
AAA	AAA(D)	1,66	1,48	3,14
	TTT(RC(D))	1,48	1,66	3,14
	TTT(C(D))	1,48	1,66	3,14
	AAA(R(D))	1,66	1,48	3,14

Composition of DNA sequence in coding chromosome 4

Base	No.of bases	%
A	2079088	28,61%
C	1582039	21,77%
G	1668662	22,96%
T	1937297	26,66%
N	0	0,00%
Sum	7267086	100,00%

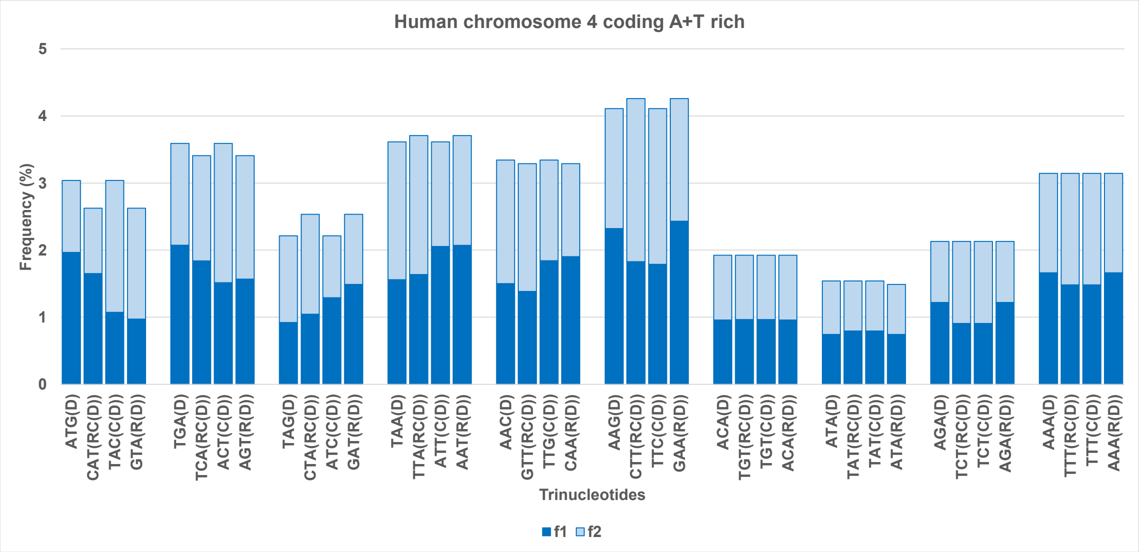

						C+G rich					
**D**	**f**	**%**	**RC**	**f**	**%**	**C**	**f**	**%**	**R**	**f**	**%**
**CGT**	418624,00	0,22	ACG	416904,00	0,22	GCA	2562248,00	1,35	TGC	2551852,00	1,34
**GTC**	1674456,00	0,88	GAC	1679588,00	0,89	CAG	3456386,00	1,82	CTG	3435697,00	1,81
**GCT**	2415291,00	1,27	AGC	2427361,00	1,28	CGA	351065,00	0,19	TCG	359140,00	0,19
**GCC**	1834224,00	0,97	GGC	1834398,00	0,97	CGG	395025,00	0,21	CCG	386822,00	0,20
**CCA**	3137057,00	1,65	TGG	3171161,00	1,67	GGT	2020839,00	1,06	ACC	2012050,00	1,06
**CCT**	2991565,00	1,58	AGG	2991490,00	1,58	GGA	2702438,00	1,42	TCC	2669170,00	1,41
**CAC**	2611187,00	1,38	GTG	2630987,00	1,39	GTG*			CAC*		
**CGC**	338715,00	0,18	GCG	340563,00	0,18	GCG*			CGC*		
**CTC**	2867660,00	1,51	GAG	2864303,00	1,51	GAG*			CTC*		
**CCC**	2007579,00	1,06	GGG	2013651,00	1,06	GGG*			CCC*		

Quadruplet		f1	f2	fu
CGT	CGT(D)	0,22	1,35	1,57
	ACG(RC(D))	0,22	1,34	1,56
	GCA(C(D))	1,35	0,22	1,57
	TGC(R(D))	1,34	0,22	1,56
GTC	GTC(D)	0,88	1,82	2,70
	GAC(RC(D))	0,89	1,81	2,70
	CAG(C(D))	1,82	0,88	2,70
	CTG(R(D))	1,81	0,89	2,70
GCT	GCT(D)	1,27	0,19	1,46
	AGC(RC(D))	1,28	0,19	1,47
	CGA(C(D))	0,19	1,27	1,46
	TCG(R(D))	0,19	1,28	1,47
GCC	GCC(D)	0,97	0,21	1,17
	GGC(RC(D))	0,97	0,20	1,17
	CGG(C(D))	0,21	0,97	1,17
	CCG(R(D))	0,20	0,97	1,17
CCA	CCA(D)	1,65	1,06	2,72
	TGG(RC(D))	1,67	1,06	2,73
	GGT(C(D))	1,06	1,65	2,72
	ACC(R(D))	1,06	1,67	2,73
CCT	CCT(D)	1,58	1,42	3,00
	AGG(RC(D))	1,58	1,41	2,98
	GGA(C(D))	1,42	1,58	3,00
	TCC(R(D))	1,41	1,58	2,98
CAC	CAC(D)	0,69	0,69	1,38
	GTG(RC(D))	0,69	0,69	1,38
	GTG(C(D))	0,69	0,69	1,38
	CAC(R(D))	0,69	0,69	1,38
CGC	CGC(D)	0,09	0,09	0,18
	GCG(RC(D))	0,09	0,09	0,18
	GCG(C(D))	0,09	0,09	0,18
	CGC(R(D))	0,09	0,09	0,18
CTC	CTC(D)	0,76	0,75	1,51
	GAG(RC(D))	0,75	0,76	1,51
	GAG(C(D))	0,75	0,76	1,51
	CTC(R(D))	0,76	0,75	1,51
CCC	CCC(D)	0,53	0,53	1,06
	GGG(RC(D))	0,53	0,53	1,06
	GGG(C(D))	0,53	0,53	1,06
	CCC(R(D))	0,53	0,53	1,06

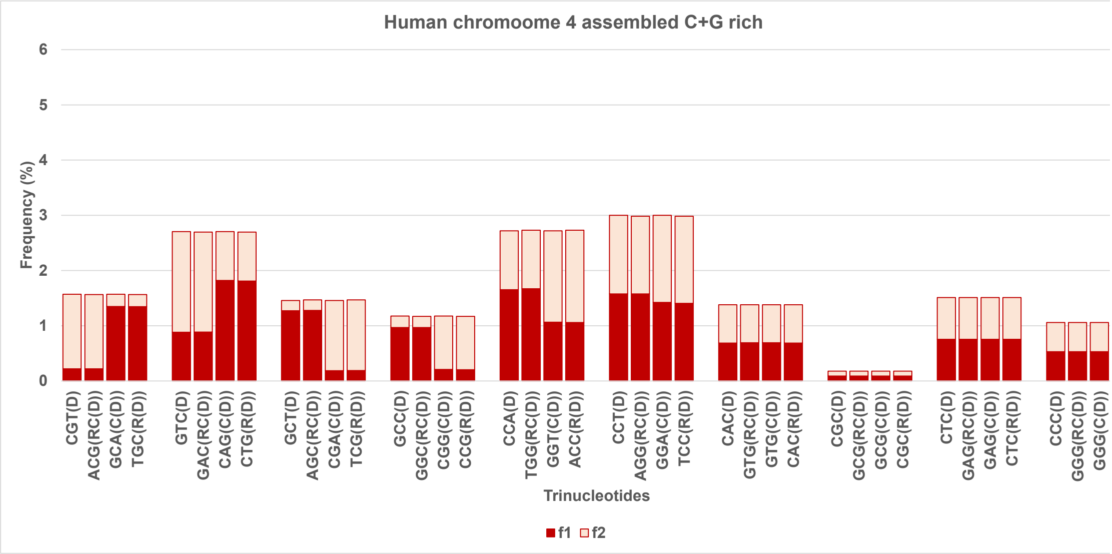

Human random chromomome 4

C+G rich

D	f	%	RC	f	%	C	f	%	R	f	%
**CGT**	502,00	0,25	ACG	493,00	0,25	GCA	2841,00	1,43	TGC	2808,00	1,41
**GTC**	1799,00	0,90	GAC	1839,00	0,93	CAG	3832,00	1,93	CTG	3810,00	1,92
**GCT**	2605,00	1,31	AGC	2675,00	1,35	CGA	381,00	0,19	TCG	427,00	0,21
**GCC**	2089,00	1,05	GGC	2023,00	1,02	CGG	479,00	0,24	CCG	472,00	0,24
**CCA**	3433,00	1,73	TGG	3448,00	1,73	GGT	2103,00	1,06	ACC	2168,00	1,09
**CCT**	3301,00	1,66	AGG	3106,00	1,56	GGA	2908,00	1,46	TCC	2951,00	1,48
**CAC**	2805,00	1,41	GTG	2972,00	1,50	GTG*			CAC*		
**CGC**	408,00	0,21	GCG	378,00	0,19	GCG*			CGC*		
**CTC**	3112,00	1,57	GAG	3010,00	1,51	GAG*			CTC*		
**CCC**	2375,00	1,19	GGG	2362,00	1,19	GGG*			CCC*		

Quadruplet		f1	f2	fu
CGT	CGT(D)	0,25	1,43	1,68
	ACG(RC(D))	0,25	1,41	1,66
	GCA(C(D))	1,43	0,25	1,68
	TGC(R(D))	1,41	0,25	1,66
GTC	GTC(D)	0,90	1,93	2,83
	GAC(RC(D))	0,93	1,92	2,84
	CAG(C(D))	1,93	0,90	2,83
	CTG(R(D))	1,92	0,93	2,84
GCT	GCT(D)	1,31	0,19	1,50
	AGC(RC(D))	1,35	0,21	1,56
	CGA(C(D))	0,19	1,31	1,50
	TCG(R(D))	0,21	1,35	1,56
GCC	GCC(D)	1,05	0,24	1,29
	GGC(RC(D))	1,02	0,24	1,26
	CGG(C(D))	0,24	1,05	1,29
	CCG(R(D))	0,24	1,02	1,26
CCA	CCA(D)	1,73	1,06	2,78
	TGG(RC(D))	1,73	1,09	2,83
	GGT(C(D))	1,06	1,73	2,78
	ACC(R(D))	1,09	1,73	2,83
CCT	CCT(D)	1,66	1,46	3,12
	AGG(RC(D))	1,56	1,48	3,05
	GGA(C(D))	1,46	1,66	3,12
	TCC(R(D))	1,48	1,56	3,05
CAC	CAC(D)	0,71	0,75	1,45
	GTG(RC(D))	0,75	0,71	1,45
	GTG(C(D))	0,75	0,71	1,45
	CAC(R(D))	0,71	0,75	1,45
CGC	CGC(D)	0,10	0,10	0,20
	GCG(RC(D))	0,10	0,10	0,20
	GCG(C(D))	0,10	0,10	0,20
	CGC(R(D))	0,10	0,10	0,20
CTC	CTC(D)	0,78	0,76	1,54
	GAG(RC(D))	0,76	0,78	1,54
	GAG(C(D))	0,76	0,78	1,54
	CTC(R(D))	0,78	0,76	1,54
CCC	CCC(D)	0,60	0,59	1,19
	GGG(RC(D))	0,59	0,60	1,19
	GGG(C(D))	0,59	0,60	1,19
	CCC(R(D))	0,60	0,59	1,19

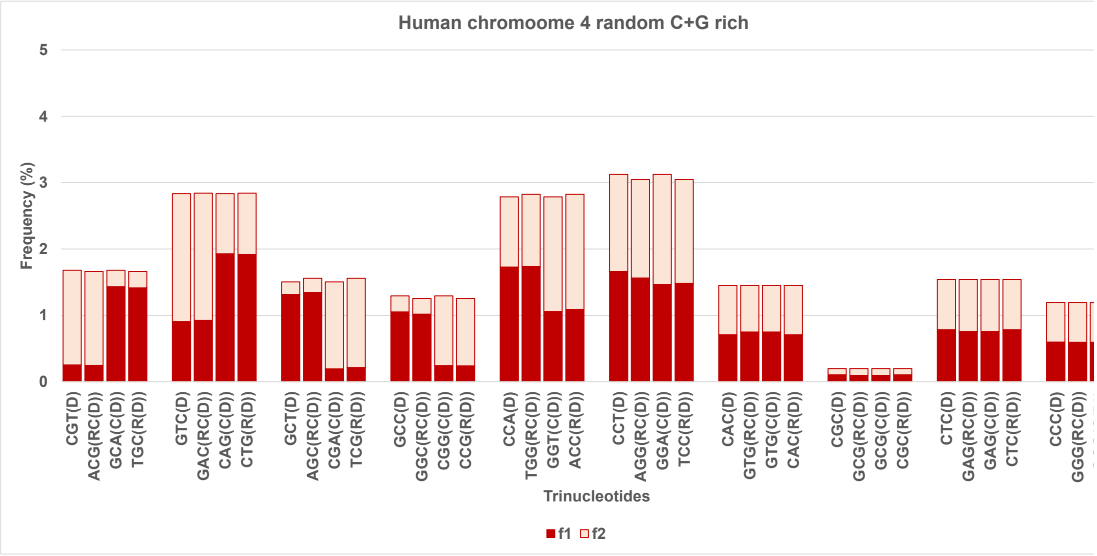

Human coding chromomome 4

					C+G rich						
**D**	**f**	**%**	**RC**	**f**	**%**	**C**	**f**	**%**	**R**	**f**	**%**
**CGT**	30714,00	0,42	ACG	33814,00	0,47	GCA	120245,00	1,65	TGC	120072,00	1,65
**GTC**	72374,00	1,00	GAC	88465,00	1,22	CAG	169190,00	2,33	CTG	161678,00	2,22
**GCT**	114176,00	1,57	AGC	122869,00	1,69	CGA	36280,00	0,50	TCG	31806,00	0,44
**GCC**	105937,00	1,46	GGC	99914,00	1,37	CGG	49545,00	0,68	CCG	48422,00	0,67
**CCA**	135811,00	1,87	TGG	146510,00	2,02	GGT	78033,00	1,07	ACC	93788,00	1,29
**CCT**	125479,00	1,73	AGG	123775,00	1,70	GGA	141883,00	1,95	TCC	109987,00	1,51
**CAC**	101447,00	1,40	GTG	119370,00	1,64	GTG*			CAC*		
**CGC**	43028,00	0,59	GCG	45525,00	0,63	GCG*			CGC*		
**CTC**	111349,00	1,53	GAG	133231,00	1,83	GAG*			CTC*		
**CCC**	101941,00	1,40	GGG	93401,00	1,29	GGG*			CCC*		

Quadruplet		f1	f2	fu
CGT	CGT(D)	0,42	1,65	2,08
	ACG(RC(D))	0,47	1,65	2,12
	GCA(C(D))	1,65	0,42	2,08
	TGC(R(D))	1,65	0,47	2,12
GTC	GTC(D)	1,00	2,33	3,32
	GAC(RC(D))	1,22	2,22	3,44
	CAG(C(D))	2,33	1,00	3,32
	CTG(R(D))	2,22	1,22	3,44
GCT	GCT(D)	1,57	0,50	2,07
	AGC(RC(D))	1,69	0,44	2,13
	CGA(C(D))	0,50	1,57	2,07
	TCG(R(D))	0,44	1,69	2,13
GCC	GCC(D)	1,46	0,68	2,14
	GGC(RC(D))	1,37	0,67	2,04
	CGG(C(D))	0,68	1,46	2,14
	CCG(R(D))	0,67	1,37	2,04
CCA	CCA(D)	1,87	1,07	2,94
	TGG(RC(D))	2,02	1,29	3,31
	GGT(C(D))	1,07	1,87	2,94
	ACC(R(D))	1,29	2,02	3,31
CCT	CCT(D)	1,73	1,95	3,68
	AGG(RC(D))	1,70	1,51	3,22
	GGA(C(D))	1,95	1,73	3,68
	TCC(R(D))	1,51	1,70	3,22
CAC	CAC(D)	0,70	0,82	1,52
	GTG(RC(D))	0,82	0,70	1,52
	GTG(C(D))	0,82	0,70	1,52
	CAC(R(D))	0,70	0,82	1,52
CGC	CGC(D)	0,30	0,31	0,61
	GCG(RC(D))	0,31	0,30	0,61
	GCG(C(D))	0,31	0,30	0,61
	CGC(R(D))	0,30	0,31	0,61
CTC	CTC(D)	0,77	0,92	1,68
	GAG(RC(D))	0,92	0,77	1,68
	GAG(C(D))	0,92	0,77	1,68
	CTC(R(D))	0,77	0,92	1,68
CCC	CCC(D)	0,70	0,64	1,34
	GGG(RC(D))	0,64	0,70	1,34
	GGG(C(D))	0,64	0,70	1,34
	CCC(R(D))	0,70	0,64	1,34

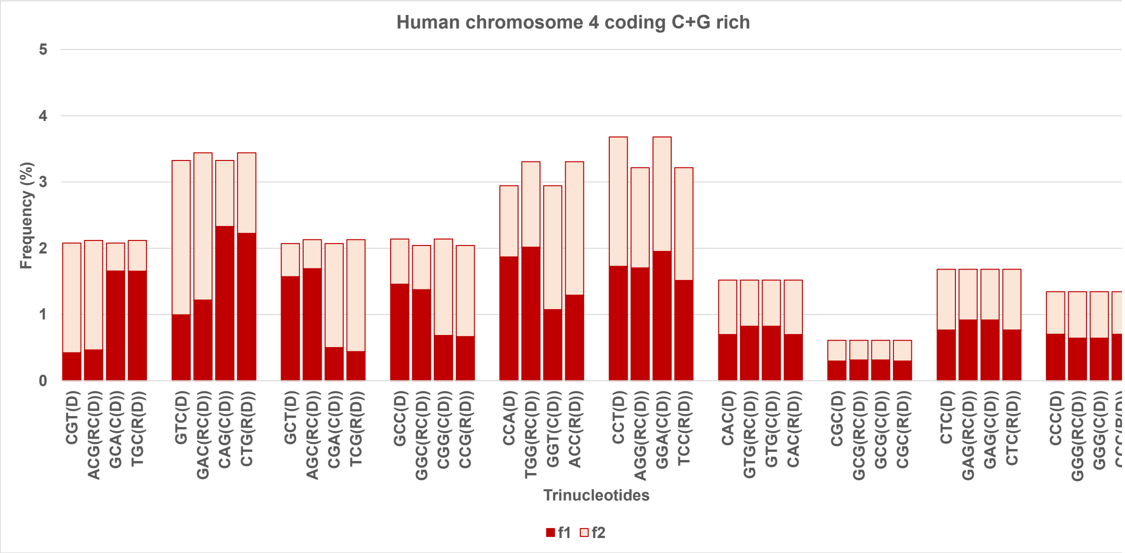

IIa

IIb

IIc

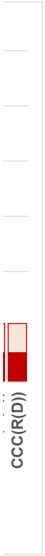

IIa

IIb

IIc

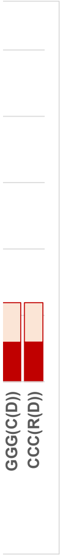

IIa

IIb

IIc

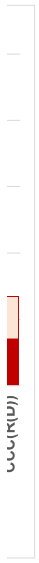

						A-T rich						
**D**	**f**	**%**	**RC**	**f**	**%**	**C**	**f**	**%**	**R**	**f**	**%**	
**ATG**	3392785,00	1,87	CAT	3397881,00	1,87	TAC	2098986,00	1,16	GTA	2132110,00	1,18	Ia
**TGA**	3589882,00	1,98	TCA	3617384,00	2,00	ACT	2976127,00	1,64	AGT	2972110,00	1,64	
**TAG**	2427849,00	1,34	CTA	2397143,00	1,32	ATC	2441384,00	1,35	GAT	2470856,00	1,36	
**TAA**	3987159,00	2,20	TTA	3983845,00	2,20	ATT	4803657,00	2,65	AAT	4769984,00	2,63	Ib
**AAC**	2748367,00	1,52	GTT	2755003,00	1,52	TTG	3575501,00	1,97	CAA	3489070,00	1,92	
**AAG**	3679243,00	2,03	CTT	3741544,00	2,06	TTC	3740856,00	2,06	GAA	3721367,00	2,05	
**ACA**	3712722,00	2,05	TGT	3772392,00	2,08	TGT*			ACA*			Ic
**ATA**	4005938,00	2,21	TAT	4005043,00	2,21	TAT*			ATA*			
**AGA**	4108677,00	2,27	TCT	4073023,00	2,25	TCT*			AGA*			
**AAA**	7241662,00	4,00	TTT	7339664,00	4,05	TTT*			AAA*			

Quadruplet		f1	f2	fu
	ATG(D)	1,87	1,16	3,03
ATG	CAT(RC(D))	1,87	1,18	3,05
	TAC(C(D))	1,16	1,87	3,03
	GTA(R(D))	1,18	1,87	3,05
	TGA(D)	1,98	1,64	3,62
TGA	TCA(RC(D))	2,00	1,64	3,64
	ACT(C(D))	1,64	1,98	3,62
	AGT(R(D))	1,64	2,00	3,64
	TAG(D)	1,34	1,35	2,69
TAG	CTA(RC(D))	1,32	1,36	2,69
	ATC(C(D))	1,35	1,34	2,69
	GAT(R(D))	1,36	1,32	2,69
	TAA(D)	2,20	2,65	4,85
TAA	TTA(RC(D))	2,20	2,63	4,83
	ATT(C(D))	2,65	2,20	4,85
	AAT(R(D))	2,63	2,20	4,83
	AAC(D)	1,52	1,97	3,49
AAC	GTT(RC(D))	1,52	1,92	3,44
	TTG(C(D))	1,97	1,52	3,49
	CAA(R(D))	1,92	1,52	3,44
	AAG(D)	2,03	2,06	4,09
AAG	CTT(RC(D))	2,06	2,05	4,12
	TTC(C(D))	2,06	2,03	4,09
	GAA(R(D))	2,05	2,06	4,12
	ACA(D)	1,02	1,04	2,06
ACA	TGT(RC(D))	1,04	1,02	2,06
	TGT(C(D))	1,04	1,02	2,06
	ACA(R(D))	1,02	1,04	2,06
	ATA(D)	1,10	1,10	2,21
ATA	TAT(RC(D))	1,10	1,10	2,21
	TAT(C(D))	1,10	1,10	2,21
	ATA(R(D))	1,10	1,10	2,21
	AGA(D)	1,13	1,12	2,26
AGA	TCT(RC(D))	1,12	1,13	2,26
	TCT(C(D))	1,12	1,13	2,26
	AGA(R(D))	1,13	1,12	2,26
	AAA(D)	2,00	2,02	4,02
AAA	TTT(RC(D))	2,02	2,00	4,02
	TTT(C(D))	2,02	2,00	4,02
	AAA(R(D))	2,00	2,02	4,02

Composition of DNA sequence in assembled chromosome 5

Base	No.of bases	%
A	54699094	30,13%
C	35731600	19,68%
G	35879674	19,76%
T	54955010	30,27%
N	272881	0,15%
Sum	181538259	100,00%

% of coding bases from complete assembled chromosome 1 (including N bases)

4,46%

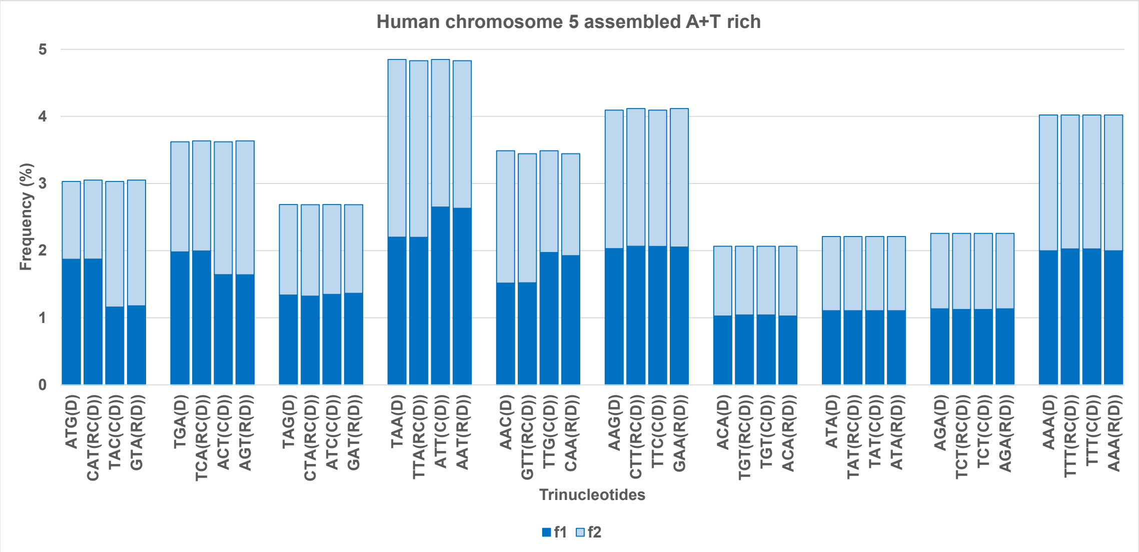

Human random chromomome 5

A-T rich

D	f	%	RC	f	%	C	f	%	R	f	%	
**ATG**	3708,00	1,86	CAT	3692,00	1,85	TAC	2302,00	1,15	GTA	2416,00	1,21	Ia
**TGA**	3932,00	1,97	TCA	4078,00	2,05	ACT	3356,00	1,68	AGT	3329,00	1,67	
**TAG**	2733,00	1,37	CTA	2598,00	1,30	ATC	2584,00	1,30	GAT	2743,00	1,38	
**TAA**	4358,00	2,19	TTA	4401,00	2,21	ATT	5430,00	2,72	AAT	5266,00	2,64	Ib
**AAC**	3008,00	1,51	GTT	3090,00	1,55	TTG	4020,00	2,02	CAA	3864,00	1,94	
**AAG**	4063,00	2,04	CTT	4064,00	2,04	TTC	4162,00	2,09	GAA	4115,00	2,06	
**ACA**	4034,00	2,02	TGT	4251,00	2,13	TGT*			ACA*			Ic
**ATA**	4455,00	2,23	TAT	4477,00	2,25	TAT*			ATA*			
**AGA**	4568,00	2,29	TCT	4455,00	2,23	TCT*			AGA*			
**AAA**	7822,00	3,92	TTT	8202,00	4,11	TTT*			AAA*			

Quadruplet		f1	f2	fu
ATG	ATG(D)	1,86	1,15	3,01
	CAT(RC(D))	1,85	1,21	3,06
	TAC(C(D))	1,15	1,86	3,01
	GTA(R(D))	1,21	1,85	3,06
TGA	TGA(D)	1,97	1,68	3,66
	TCA(RC(D))	2,05	1,67	3,71
	ACT(C(D))	1,68	1,97	3,66
	AGT(R(D))	1,67	2,05	3,71
TAG	TAG(D)	1,37	1,30	2,67
	CTA(RC(D))	1,30	1,38	2,68
	ATC(C(D))	1,30	1,37	2,67
	GAT(R(D))	1,38	1,30	2,68
TAA	TAA(D)	2,19	2,72	4,91
	TTA(RC(D))	2,21	2,64	4,85
	ATT(C(D))	2,72	2,19	4,91
	AAT(R(D))	2,64	2,21	4,85
AAC	AAC(D)	1,51	2,02	3,52
	GTT(RC(D))	1,55	1,94	3,49
	TTG(C(D))	2,02	1,51	3,52
	CAA(R(D))	1,94	1,55	3,49
AAG	AAG(D)	2,04	2,09	4,13
	CTT(RC(D))	2,04	2,06	4,10
	TTC(C(D))	2,09	2,04	4,13
	GAA(R(D))	2,06	2,04	4,10
ACA	ACA(D)	1,01	1,07	2,08
	TGT(RC(D))	1,07	1,01	2,08
	TGT(C(D))	1,07	1,01	2,08
	ACA(R(D))	1,01	1,07	2,08
ATA	ATA(D)	1,12	1,12	2,24
	TAT(RC(D))	1,12	1,12	2,24
	TAT(C(D))	1,12	1,12	2,24
	ATA(R(D))	1,12	1,12	2,23
AGA	AGA(D)	1,15	1,12	2,26
	TCT(RC(D))	1,12	1,15	2,26
	TCT(C(D))	1,12	1,15	2,26
	AGA(R(D))	1,15	1,12	2,26
AAA	AAA(D)	1,96	2,06	4,02
	TTT(RC(D))	2,06	1,96	4,02
	TTT(C(D))	2,06	1,96	4,02
	AAA(R(D))	1,96	2,06	4,02

Composition of DNA sequence in random chromosome 5

Base	No.of bases	%
A	60196	30,10%
C	38942	19,47%
G	39392	19,70%
T	60870	30,44%
N	600	0,30%
Sum	200000	100,00%

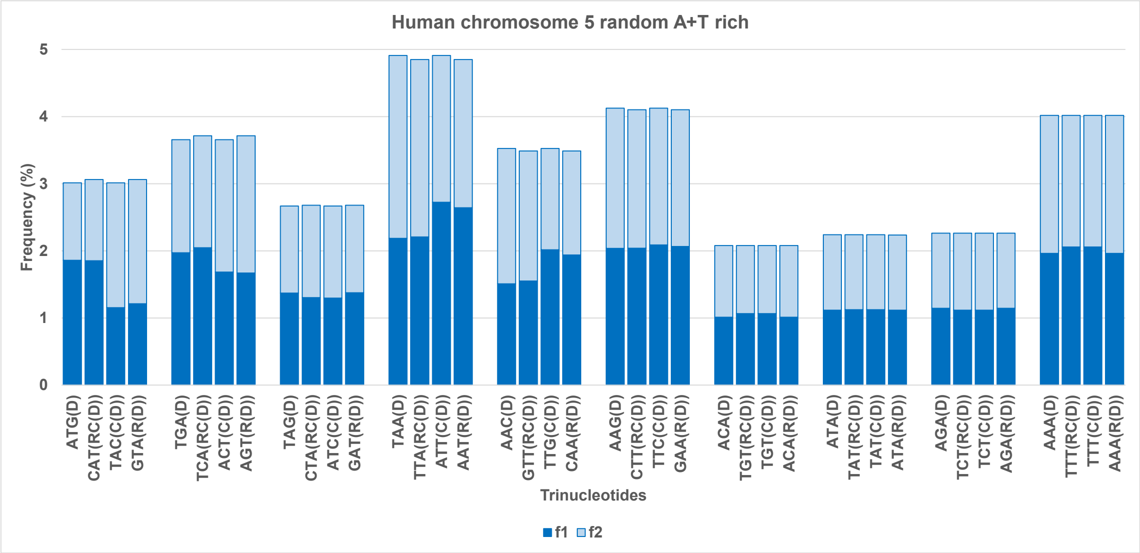

Human coding chromomome 5

A-T rich

D	f	%	RC	f	%	C	f	%	R	f	%	
**ATG**	151709,00	1,87	CAT	128582,00	1,59	TAC	84307,00	1,04	GTA	74915,00	0,93	Ia
**TGA**	163322,00	2,02	TCA	145087,00	1,79	ACT	119565,00	1,48	AGT	123009,00	1,52	
**TAG**	71857,00	0,89	CTA	84689,00	1,05	ATC	102197,00	1,26	GAT	117183,00	1,45	
**TAA**	116817,00	1,44	TTA	119980,00	1,48	ATT	150760,00	1,86	AAT	152298,00	1,88	Ib
**AAC**	116116,00	1,43	GTT	105662,00	1,31	TTG	139653,00	1,73	CAA	147224,00	1,82	
**AAG**	183297,00	2,26	CTT	144995,00	1,79	TTC	141783,00	1,75	GAA	187670,00	2,32	
**ACA**	147235,00	1,82	TGT	149861,00	1,85	TGT*			ACA*			Ic
**ATA**	109537,00	1,35	TAT	116140,00	1,43	TAT*			ATA*			
**AGA**	195475,00	2,41	TCT	147173,00	1,82	TCT*			AGA*			
**AAA**	244971,00	3,03	TTT	221047,00	2,73	TTT*			AAA*			

Quadruplet		f1	f2	fu
ATG	ATG(D)	1,87	1,04	2,92
	CAT(RC(D))	1,59	0,93	2,51
	TAC(C(D))	1,04	1,87	2,92
	GTA(R(D))	0,93	1,59	2,51
TGA	TGA(D)	2,02	1,48	3,49
	TCA(RC(D))	1,79	1,52	3,31
	ACT(C(D))	1,48	2,02	3,49
	AGT(R(D))	1,52	1,79	3,31
TAG	TAG(D)	0,89	1,26	2,15
	CTA(RC(D))	1,05	1,45	2,49
	ATC(C(D))	1,26	0,89	2,15
	GAT(R(D))	1,45	1,05	2,49
TAA	TAA(D)	1,44	1,86	3,31
	TTA(RC(D))	1,48	1,88	3,36
	ATT(C(D))	1,86	1,44	3,31
	AAT(R(D))	1,88	1,48	3,36
AAC	AAC(D)	1,43	1,73	3,16
	GTT(RC(D))	1,31	1,82	3,12
	TTG(C(D))	1,73	1,43	3,16
	CAA(R(D))	1,82	1,31	3,12
AAG	AAG(D)	2,26	1,75	4,02
	CTT(RC(D))	1,79	2,32	4,11
	TTC(C(D))	1,75	2,26	4,02
	GAA(R(D))	2,32	1,79	4,11
ACA	ACA(D)	0,91	0,93	1,83
	TGT(RC(D))	0,93	0,91	1,83
	TGT(C(D))	0,93	0,91	1,83
	ACA(R(D))	0,91	0,93	1,83
ATA	ATA(D)	0,68	0,72	1,39
	TAT(RC(D))	0,72	0,68	1,39
	TAT(C(D))	0,72	0,68	1,39
	ATA(R(D))	0,68	0,68	1,35
AGA	AGA(D)	1,21	0,91	2,12
	TCT(RC(D))	0,91	1,21	2,12
	TCT(C(D))	0,91	1,21	2,12
	AGA(R(D))	1,21	0,91	2,12
AAA	AAA(D)	1,51	1,37	2,88
	TTT(RC(D))	1,37	1,51	2,88
	TTT(C(D))	1,37	1,51	2,88
	AAA(R(D))	1,51	1,37	2,88

Composition of DNA sequence in coding chromosome 5

Base	No.of bases	%
A	2240505	27,68%
C	1841464	22,75%
G	1924304	23,77%
T	2089398	25,81%
N	0	0,00%
Sum	8095671	100,00%

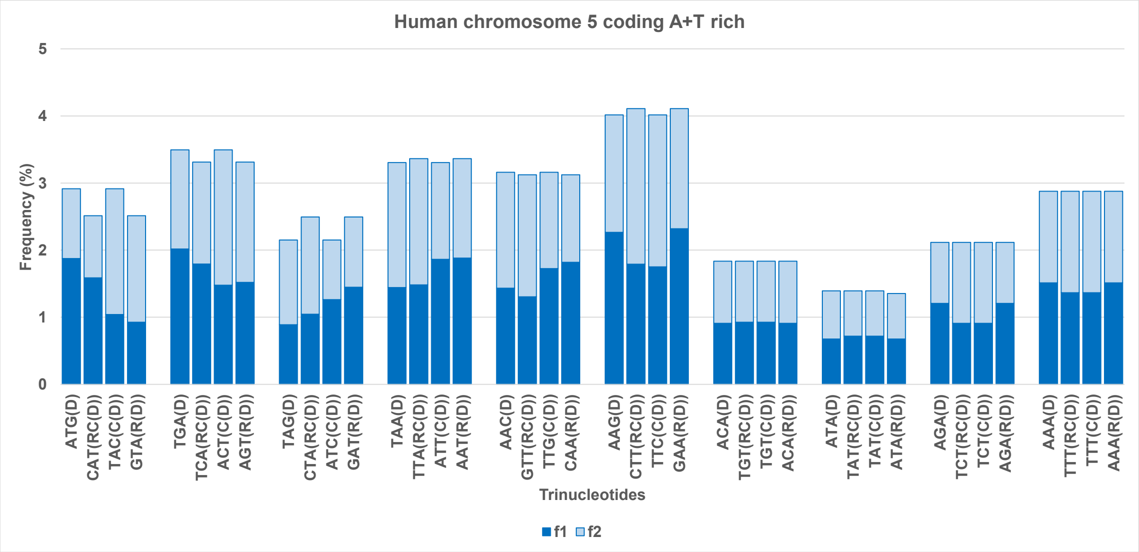

						C+G rich					
**D**	**f**	**%**	**RC**	**f**	**%**	**C**	**f**	**%**	**R**	**f**	**%**
**CGT**	435052,00	0,24	ACG	423811,00	0,23	GCA	2530970,00	1,40	TGC	2532272,00	1,40
**GTC**	1646549,00	0,91	GAC	1660542,00	0,92	CAG	3509347,00	1,94	CTG	3460922,00	1,91
**GCT**	2410352,00	1,33	AGC	2416044,00	1,33	CGA	356919,00	0,20	TCG	366150,00	0,20
**GCC**	1916726,00	1,06	GGC	1918695,00	1,06	CGG	415822,00	0,23	CCG	408867,00	0,23
**CCA**	3131649,00	1,73	TGG	3181262,00	1,76	GGT	2000708,00	1,10	ACC	1991663,00	1,10
**CCT**	3031597,00	1,67	AGG	3014895,00	1,66	GGA	2692577,00	1,49	TCC	2663724,00	1,47
**CAC**	2596429,00	1,43	GTG	2646601,00	1,46	GTG*			CAC*		
**CGC**	358742,00	0,20	GCG	367706,00	0,20	GCG*			CGC*		
**CTC**	2891490,00	1,60	GAG	2895278,00	1,60	GAG*			CTC*		
**CCC**	2109118,00	1,16	GGG	2113613,00	1,17	GGG*			CCC*		

Quadruplet		f1	f2	fu
	CGT(D)	0,24	1,40	1,64
CGT	ACG(RC(D))	0,23	1,40	1,63
	GCA(C(D))	1,40	0,24	1,64
	TGC(R(D))	1,40	0,23	1,63
	GTC(D)	0,91	1,94	2,84
GTC	GAC(RC(D))	0,92	1,91	2,83
	CAG(C(D))	1,94	0,91	2,84
	CTG(R(D))	1,91	0,92	2,83
	GCT(D)	1,33	0,20	1,53
GCT	AGC(RC(D))	1,33	0,20	1,53
	CGA(C(D))	0,20	1,33	1,53
	TCG(R(D))	0,20	1,33	1,53
	GCC(D)	1,06	0,23	1,29
GCC	GGC(RC(D))	1,06	0,23	1,28
	CGG(C(D))	0,23	1,06	1,29
	CCG(R(D))	0,23	1,06	1,28
	CCA(D)	1,73	1,10	2,83
CCA	TGG(RC(D))	1,76	1,10	2,85
	GGT(C(D))	1,10	1,73	2,83
	ACC(R(D))	1,10	1,76	2,85
	CCT(D)	1,67	1,49	3,16
CCT	AGG(RC(D))	1,66	1,47	3,13
	GGA(C(D))	1,49	1,67	3,16
	TCC(R(D))	1,47	1,66	3,13
	CAC(D)	0,72	0,73	1,45
CAC	GTG(RC(D))	0,73	0,72	1,45
	GTG(C(D))	0,73	0,72	1,45
	CAC(R(D))	0,72	0,73	1,45
	CGC(D)	0,10	0,10	0,20
CGC	GCG(RC(D))	0,10	0,10	0,20
	GCG(C(D))	0,10	0,10	0,20
	CGC(R(D))	0,10	0,10	0,20
	CTC(D)	0,80	0,80	1,60
CTC	GAG(RC(D))	0,80	0,80	1,60
	GAG(C(D))	0,80	0,80	1,60
	CTC(R(D))	0,80	0,80	1,60
	CCC(D)	0,58	0,58	1,16
CCC	GGG(RC(D))	0,58	0,58	1,16
	GGG(C(D))	0,58	0,58	1,16
	CCC(R(D))	0,58	0,58	1,16

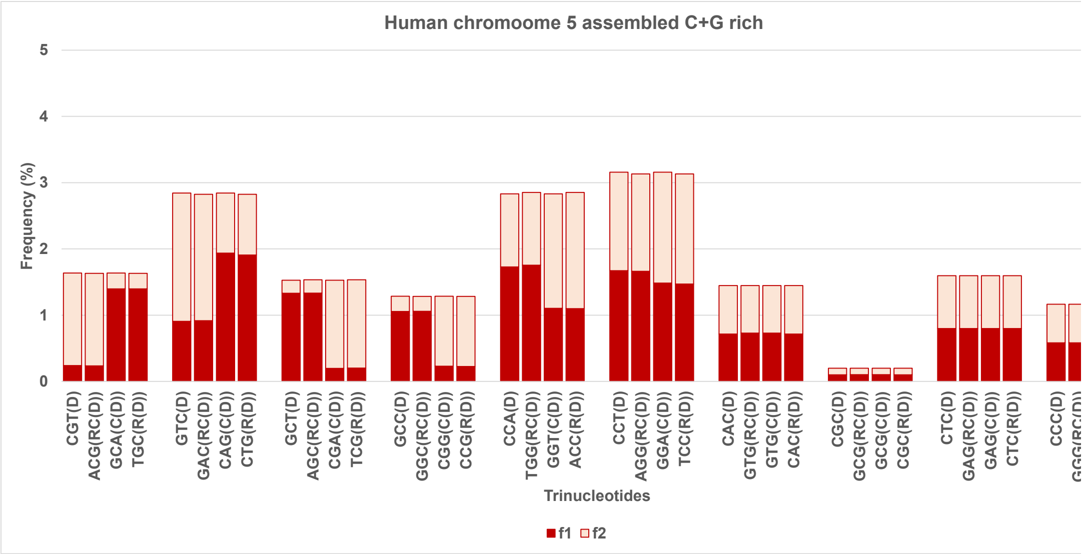

Human random chromomome 5

C+G rich											
**D**	**f**	**%**	**RC**	**f**	**%**	**C**	**f**	**%**	**R**	**f**	**%**
**CGT**	535,00	0,27	ACG	490,00	0,25	GCA	2818,00	1,41	TGC	2791,00	1,40
**GTC**	1787,00	0,90	GAC	1780,00	0,89	CAG	3796,00	1,90	CTG	3703,00	1,86
**GCT**	2645,00	1,33	AGC	2651,00	1,33	CGA	420,00	0,21	TCG	426,00	0,21
**GCC**	2028,00	1,02	GGC	2073,00	1,04	CGG	466,00	0,23	CCG	482,00	0,24
**CCA**	3364,00	1,69	TGG	3451,00	1,73	GGT	2171,00	1,09	ACC	2153,00	1,08
**CCT**	3162,00	1,59	AGG	3278,00	1,64	GGA	2951,00	1,48	TCC	2827,00	1,42
**CAC**	2943,00	1,48	GTG	2993,00	1,50	GTG*			CAC*		
**CGC**	434,00	0,22	GCG	457,00	0,23	GCG*			CGC*		
**CTC**	3254,00	1,63	GAG	3234,00	1,62	GAG*			CTC*		
**CCC**	2163,00	1,08	GGG	2090,00	1,05	GGG*			CCC*		

Quadruplet		f1	f2	fu
CGT	CGT(D)	0,27	1,41	1,68
	ACG(RC(D))	0,25	1,40	1,65
	GCA(C(D))	1,41	0,27	1,68
	TGC(R(D))	1,40	0,25	1,65
GTC	GTC(D)	0,90	1,90	2,80
	GAC(RC(D))	0,89	1,86	2,75
	CAG(C(D))	1,90	0,90	2,80
	CTG(R(D))	1,86	0,89	2,75
GCT	GCT(D)	1,33	0,21	1,54
	AGC(RC(D))	1,33	0,21	1,54
	CGA(C(D))	0,21	1,33	1,54
	TCG(R(D))	0,21	1,33	1,54
GCC	GCC(D)	1,02	0,23	1,25
	GGC(RC(D))	1,04	0,24	1,28
	CGG(C(D))	0,23	1,02	1,25
	CCG(R(D))	0,24	1,04	1,28
CCA	CCA(D)	1,69	1,09	2,78
	TGG(RC(D))	1,73	1,08	2,81
	GGT(C(D))	1,09	1,69	2,78
	ACC(R(D))	1,08	1,73	2,81
CCT	CCT(D)	1,59	1,48	3,07
	AGG(RC(D))	1,64	1,42	3,06
	GGA(C(D))	1,48	1,59	3,07
	TCC(R(D))	1,42	1,64	3,06
CAC	CAC(D)	0,74	0,75	1,49
	GTG(RC(D))	0,75	0,74	1,49
	GTG(C(D))	0,75	0,74	1,49
	CAC(R(D))	0,74	0,75	1,49
CGC	CGC(D)	0,11	0,11	0,22
	GCG(RC(D))	0,11	0,11	0,22
	GCG(C(D))	0,11	0,11	0,22
	CGC(R(D))	0,11	0,11	0,22
CTC	CTC(D)	0,82	0,81	1,63
	GAG(RC(D))	0,81	0,82	1,63
	GAG(C(D))	0,81	0,82	1,63
	CTC(R(D))	0,82	0,81	1,63
CCC	CCC(D)	0,54	0,52	1,07
	GGG(RC(D))	0,52	0,54	1,07
	GGG(C(D))	0,52	0,54	1,07
	CCC(R(D))	0,54	0,52	1,07

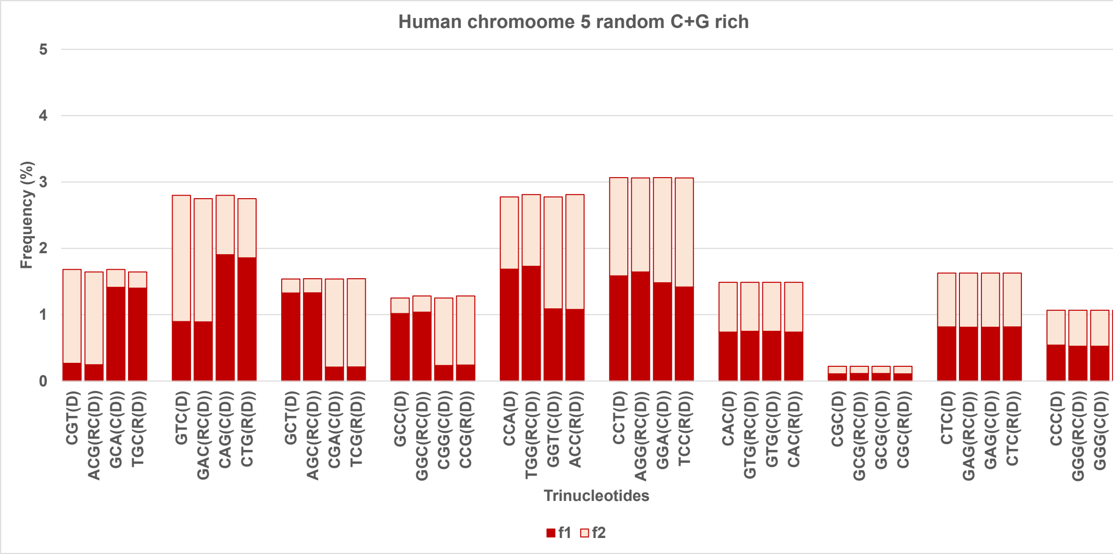

Human coding chromomome 5

C+G rich											
**D**	**f**	**%**	**RC**	**f**	**%**	**C**	**f**	**%**	**R**	**f**	**%**
**CGT**	35849,00	0,44	ACG	40402,00	0,50	GCA	133516,00	1,65	TGC	135072,00	1,67
**GTC**	83368,00	1,03	GAC	102778,00	1,27	CAG	198884,00	2,46	CTG	191852,00	2,37
**GCT**	135581,00	1,67	AGC	144768,00	1,79	CGA	42148,00	0,52	TCG	37680,00	0,47
**GCC**	134404,00	1,66	GGC	125289,00	1,55	CGG	61456,00	0,76	CCG	59742,00	0,74
**CCA**	163099,00	2,01	TGG	170500,00	2,11	GGT	90767,00	1,12	ACC	110246,00	1,36
**CCT**	150926,00	1,86	AGG	148920,00	1,84	GGA	164820,00	2,04	TCC	129117,00	1,59
**CAC**	114247,00	1,41	GTG	135541,00	1,67	GTG*			CAC*		
**CGC**	54658,00	0,68	GCG	56287,00	0,70	GCG*			CGC*		
**CTC**	131709,00	1,63	GAG	158134,00	1,95	GAG*			CTC*		
**CCC**	131404,00	1,62	GGG	118389,00	1,46	GGG*			CCC*		

Quadruplet		f1	f2	fu
CGT	CGT(D)	0,44	1,65	2,09
	ACG(RC(D))	0,50	1,67	2,17
	GCA(C(D))	1,65	0,44	2,09
	TGC(R(D))	1,67	0,50	2,17
GTC	GTC(D)	1,03	2,46	3,49
	GAC(RC(D))	1,27	2,37	3,64
	CAG(C(D))	2,46	1,03	3,49
	CTG(R(D))	2,37	1,27	3,64
GCT	GCT(D)	1,67	0,52	2,20
	AGC(RC(D))	1,79	0,47	2,25
	CGA(C(D))	0,52	1,67	2,20
	TCG(R(D))	0,47	1,79	2,25
GCC	GCC(D)	1,66	0,76	2,42
	GGC(RC(D))	1,55	0,74	2,29
	CGG(C(D))	0,76	1,66	2,42
	CCG(R(D))	0,74	1,55	2,29
CCA	CCA(D)	2,01	1,12	3,14
	TGG(RC(D))	2,11	1,36	3,47
	GGT(C(D))	1,12	2,01	3,14
	ACC(R(D))	1,36	2,11	3,47
CCT	CCT(D)	1,86	2,04	3,90
	AGG(RC(D))	1,84	1,59	3,43
	GGA(C(D))	2,04	1,86	3,90
	TCC(R(D))	1,59	1,84	3,43
CAC	CAC(D)	0,71	0,84	1,54
	GTG(RC(D))	0,84	0,71	1,54
	GTG(C(D))	0,84	0,71	1,54
	CAC(R(D))	0,71	0,84	1,54
CGC	CGC(D)	0,34	0,35	0,69
	GCG(RC(D))	0,35	0,34	0,69
	GCG(C(D))	0,35	0,34	0,69
	CGC(R(D))	0,34	0,35	0,69
CTC	CTC(D)	0,81	0,98	1,79
	GAG(RC(D))	0,98	0,81	1,79
	GAG(C(D))	0,98	0,81	1,79
	CTC(R(D))	0,81	0,98	1,79
CCC	CCC(D)	0,81	0,73	1,54
	GGG(RC(D))	0,73	0,81	1,54
	GGG(C(D))	0,73	0,81	1,54
	CCC(R(D))	0,81	0,73	1,54

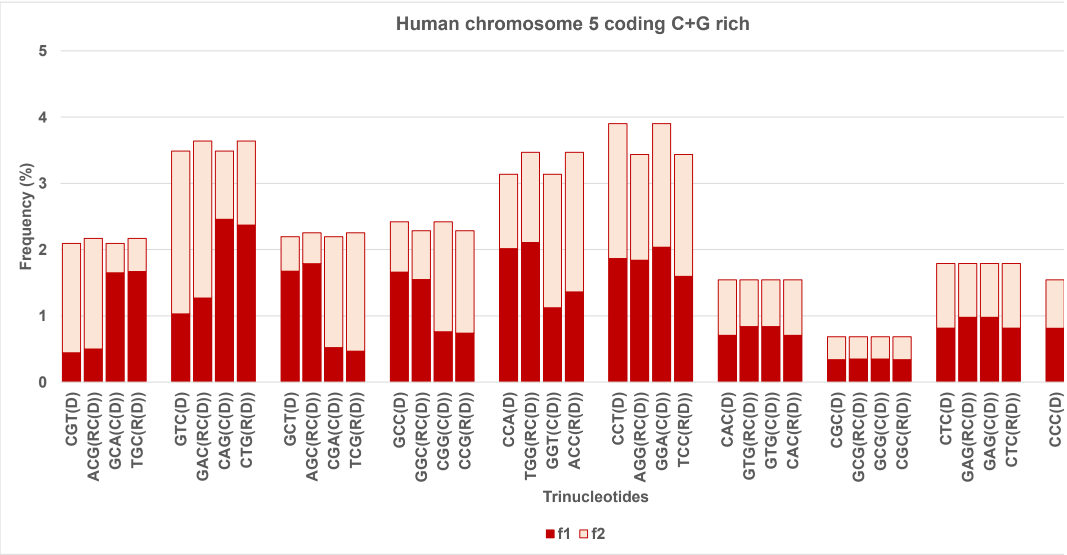

IIa

IIb

IIc

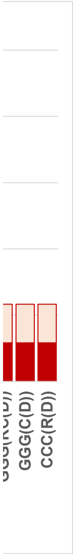

IIa

IIb

IIc

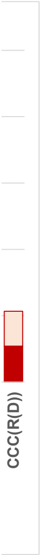

IIa

IIb

IIc

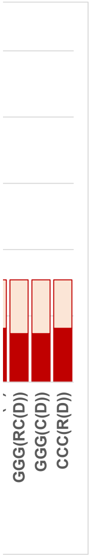
GGG(RC(D))
GGG(C(D))
CCC(R(D))

D	f	%	RC	f	%	C	f	%	R	f	%	
					A-T rich							
**ATG**	3171172,00	1,86	CAT	3175600,00	1,87	TAC	1986597,00	1,17	GTA	1998057,00	1,17	Ia
**TGA**	3364732,00	1,98	TCA	3357437,00	1,97	ACT	2781753,00	1,64	AGT	2781493,00	1,64	
**TAG**	2273553,00	1,34	CTA	2258498,00	1,33	ATC	2291476,00	1,35	GAT	2310697,00	1,36	
**TAA**	3759408,00	2,21	TTA	3755447,00	2,21	ATT	4490930,00	2,64	AAT	4474835,00	2,63	Ib
**AAC**	2566138,00	1,51	GTT	2550783,00	1,50	TTG	3304698,00	1,94	CAA	3271520,00	1,92	
**AAG**	3464669,00	2,04	CTT	3481001,00	2,05	TTC	3462570,00	2,04	GAA	3474715,00	2,04	
**ACA**	3475138,00	2,04	TGT	3483901,00	2,05	TGT*			ACA*			Ic
**ATA**	3740764,00	2,20	TAT	3733210,00	2,19	TAT*			ATA*			
**AGA**	3823792,00	2,25	TCT	3809186,00	2,24	TCT*			AGA*			
**AAA**	6839525,00	4,02	TTT	6869921,00	4,04	TTT*			AAA*			

Quadruplet		f1	f2	fu
ATG	ATG(D)	1,86	1,17	3,03
	CAT(RC(D))	1,87	1,17	3,04
	TAC(C(D))	1,17	1,86	3,03
	GTA(R(D))	1,17	1,87	3,04
TGA	TGA(D)	1,98	1,64	3,61
	TCA(RC(D))	1,97	1,64	3,61
	ACT(C(D))	1,64	1,98	3,61
	AGT(R(D))	1,64	1,97	3,61
TAG	TAG(D)	1,34	1,35	2,68
	CTA(RC(D))	1,33	1,36	2,69
	ATC(C(D))	1,35	1,34	2,68
	GAT(R(D))	1,36	1,33	2,69
TAA	TAA(D)	2,21	2,64	4,85
	TTA(RC(D))	2,21	2,63	4,84
	ATT(C(D))	2,64	2,21	4,85
	AAT(R(D))	2,63	2,21	4,84
AAC	AAC(D)	1,51	1,94	3,45
	GTT(RC(D))	1,50	1,92	3,42
	TTG(C(D))	1,94	1,51	3,45
	CAA(R(D))	1,92	1,50	3,42
AAG	AAG(D)	2,04	2,04	4,07
	CTT(RC(D))	2,05	2,04	4,09
	TTC(C(D))	2,04	2,04	4,07
	GAA(R(D))	2,04	2,05	4,09
ACA	ACA(D)	1,02	1,02	2,05
	TGT(RC(D))	1,02	1,02	2,05
	TGT(C(D))	1,02	1,02	2,05
	ACA(R(D))	1,02	1,02	2,05
ATA	ATA(D)	1,10	1,10	2,20
	TAT(RC(D))	1,10	1,10	2,20
	TAT(C(D))	1,10	1,10	2,20
	ATA(R(D))	1,10	1,10	2,20
AGA	AGA(D)	1,12	1,12	2,24
	TCT(RC(D))	1,12	1,12	2,24
	TCT(C(D))	1,12	1,12	2,24
	AGA(R(D))	1,12	1,12	2,24
AAA	AAA(D)	2,01	2,02	4,03
	TTT(RC(D))	2,02	2,01	4,03
	TTT(C(D))	2,02	2,01	4,03
	AAA(R(D))	2,01	2,02	4,03

Composition of DNA sequence in assembled chromosome 6

Base	No.of bases	%
A	51345477	30,06%
C	33646690	19,70%
G	33713330	19,74%
T	51373025	30,08%
N	727457	0,43%
Sum	170805979	100,00%

% of coding bases from complete assembled chromosome 1 (including N bases)

5,22%

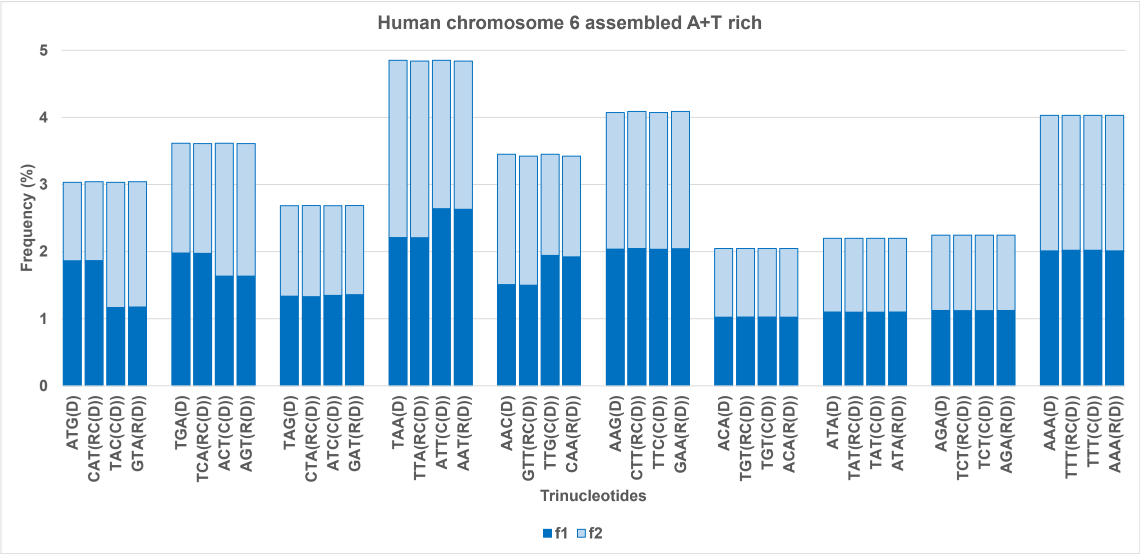

Human random chromomome 6

A-T rich												
**D**	**f**	**%**	**RC**	**f**	**%**	**C**	**f**	**%**	**R**	**f**	**%**	
**ATG**	3683,00	1,86	CAT	3603,00	1,82	TAC	2279,00	1,15	GTA	2321,00	1,17	Ia
**TGA**	4059,00	2,05	TCA	3798,00	1,91	ACT	3161,00	1,59	AGT	3264,00	1,65	
**TAG**	2579,00	1,30	CTA	2530,00	1,28	ATC	2689,00	1,36	GAT	2656,00	1,34	
**TAA**	4098,00	2,07	TTA	4118,00	2,08	ATT	4920,00	2,48	AAT	5022,00	2,53	Ib
**AAC**	2871,00	1,45	GTT	2909,00	1,47	TTG	3805,00	1,92	CAA	3763,00	1,90	
**AAG**	3897,00	1,96	CTT	4149,00	2,09	TTC	4055,00	2,04	GAA	3929,00	1,98	
**ACA**	3974,00	2,00	TGT	3958,00	2,00	TGT*			ACA*			Ic
**ATA**	4170,00	2,10	TAT	4181,00	2,11	TAT*			ATA*			
**AGA**	4271,00	2,15	TCT	4487,00	2,26	TCT*			AGA*			
**AAA**	7655,00	3,86	TTT	7570,00	3,82	TTT*			AAA*			

Quadruplet		f1	f2	fu
ATG	ATG(D)	1,86	1,15	3,01
	CAT(RC(D))	1,82	1,17	2,99
	TAC(C(D))	1,15	1,86	3,01
	GTA(R(D))	1,17	1,82	2,99
TGA	TGA(D)	2,05	1,59	3,64
	TCA(RC(D))	1,91	1,65	3,56
	ACT(C(D))	1,59	2,05	3,64
	AGT(R(D))	1,65	1,91	3,56
TAG	TAG(D)	1,30	1,36	2,66
	CTA(RC(D))	1,28	1,34	2,61
	ATC(C(D))	1,36	1,30	2,66
	GAT(R(D))	1,34	1,28	2,61
TAA	TAA(D)	2,07	2,48	4,55
	TTA(RC(D))	2,08	2,53	4,61
	ATT(C(D))	2,48	2,07	4,55
	AAT(R(D))	2,53	2,08	4,61
AAC	AAC(D)	1,45	1,92	3,37
	GTT(RC(D))	1,47	1,90	3,36
	TTG(C(D))	1,92	1,45	3,37
	CAA(R(D))	1,90	1,47	3,36
AAG	AAG(D)	1,96	2,04	4,01
	CTT(RC(D))	2,09	1,98	4,07
	TTC(C(D))	2,04	1,96	4,01
	GAA(R(D))	1,98	2,09	4,07
ACA	ACA(D)	1,00	1,00	2,00
	TGT(RC(D))	1,00	1,00	2,00
	TGT(C(D))	1,00	1,00	2,00
	ACA(R(D))	1,00	1,00	2,00
ATA	ATA(D)	1,05	1,05	2,10
	TAT(RC(D))	1,05	1,05	2,10
	TAT(C(D))	1,05	1,05	2,10
	ATA(R(D))	1,05	1,05	2,10
AGA	AGA(D)	1,08	1,13	2,21
	TCT(RC(D))	1,13	1,08	2,21
	TCT(C(D))	1,13	1,08	2,21
	AGA(R(D))	1,08	1,13	2,21
AAA	AAA(D)	1,93	1,91	3,84
	TTT(RC(D))	1,91	1,93	3,84
	TTT(C(D))	1,91	1,93	3,84
	AAA(R(D))	1,93	1,91	3,84

Composition of DNA sequence in random chromosome 6

Base	No.of bases	%
A	58593	29,30%
C	40391	20,20%
G	40397	20,20%
T	59019	29,51%
N	1600	0,80%
Sum	200000	100,00%

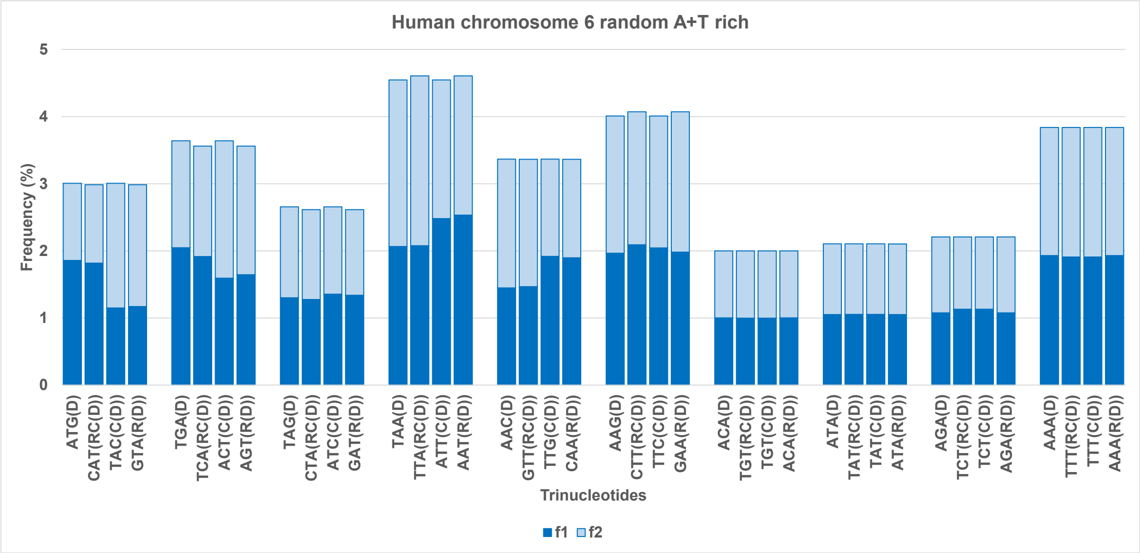

Human coding chromomome 6

A-T rich

D	f	%	RC	f	%	C	f	%	R	f	%	
**ATG**	163429,00	1,83	CAT	140475,00	1,58	TAC	90057,00	1,01	GTA	79607,00	0,89	Ia
**TGA**	177376,00	1,99	TCA	160182,00	1,80	ACT	131264,00	1,47	AGT	133833,00	1,50	
**TAG**	76127,00	0,85	CTA	90473,00	1,01	ATC	112615,00	1,26	GAT	125281,00	1,40	
**TAA**	119790,00	1,34	TTA	124682,00	1,40	ATT	157701,00	1,77	AAT	159201,00	1,78	Ib
**AAC**	125414,00	1,41	GTT	113535,00	1,27	TTG	151426,00	1,70	CAA	159251,00	1,79	
**AAG**	199858,00	2,24	CTT	159714,00	1,79	TTC	154842,00	1,74	GAA	205433,00	2,30	
**ACA**	159876,00	1,79	TGT	160192,00	1,80	TGT*			ACA*			Ic
**ATA**	111529,00	1,25	TAT	120317,00	1,35	TAT*			ATA*			
**AGA**	216498,00	2,43	TCT	164670,00	1,85	TCT*			AGA*			
**AAA**	256571,00	2,88	TTT	230393,00	2,58	TTT*			AAA*			

Quadruplet		f1	f2	fu
ATG	ATG(D)	1,83	1,01	2,84
	CAT(RC(D))	1,58	0,89	2,47
	TAC(C(D))	1,01	1,83	2,84
	GTA(R(D))	0,89	1,58	2,47
TGA	TGA(D)	1,99	1,47	3,46
	TCA(RC(D))	1,80	1,50	3,30
	ACT(C(D))	1,47	1,99	3,46
	AGT(R(D))	1,50	1,80	3,30
TAG	TAG(D)	0,85	1,26	2,12
	CTA(RC(D))	1,01	1,40	2,42
	ATC(C(D))	1,26	0,85	2,12
	GAT(R(D))	1,40	1,01	2,42
TAA	TAA(D)	1,34	1,77	3,11
	TTA(RC(D))	1,40	1,78	3,18
	ATT(C(D))	1,77	1,34	3,11
	AAT(R(D))	1,78	1,40	3,18
AAC	AAC(D)	1,41	1,70	3,10
	GTT(RC(D))	1,27	1,79	3,06
	TTG(C(D))	1,70	1,41	3,10
	CAA(R(D))	1,79	1,27	3,06
AAG	AAG(D)	2,24	1,74	3,98
	CTT(RC(D))	1,79	2,30	4,09
	TTC(C(D))	1,74	2,24	3,98
	GAA(R(D))	2,30	1,79	4,09
ACA	ACA(D)	0,90	0,90	1,79
	TGT(RC(D))	0,90	0,90	1,79
	TGT(C(D))	0,90	0,90	1,79
	ACA(R(D))	0,90	0,90	1,79
ATA	ATA(D)	0,63	0,67	1,30
	TAT(RC(D))	0,67	0,63	1,30
	TAT(C(D))	0,67	0,63	1,30
	ATA(R(D))	0,63	0,63	1,25
AGA	AGA(D)	1,21	0,92	2,14
	TCT(RC(D))	0,92	1,21	2,14
	TCT(C(D))	0,92	1,21	2,14
	AGA(R(D))	1,21	0,92	2,14
AAA	AAA(D)	1,44	1,29	2,73
	TTT(RC(D))	1,29	1,44	2,73
	TTT(C(D))	1,29	1,44	2,73
	AAA(R(D))	1,44	1,29	2,73

Composition of DNA sequence in coding chromosome 6

Base	No.of bases	%
A	2428889	27,23%
C	2078402	23,30%
G	2148159	24,09%
T	2263612	25,38%
N	0	0,00%
Sum	8919062	100,00%

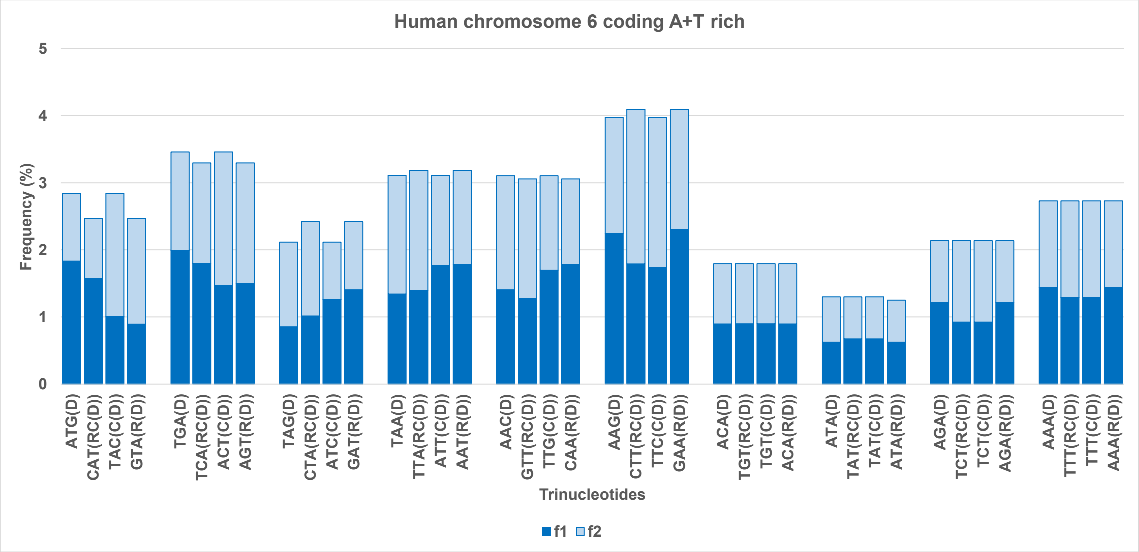

C+G rich											
**D**	**f**	**%**	**RC**	**f**	**%**	**C**	**f**	**%**	**R**	**f**	**%**
**CGT**	403119,00	0,24	ACG	402242,00	0,24	GCA	2371890,00	1,39	TGC	2361630,00	1,39
**GTC**	1550525,00	0,91	GAC	1558380,00	0,92	CAG	3272449,00	1,92	CTG	3256271,00	1,91
**GCT**	2264892,00	1,33	AGC	2279487,00	1,34	CGA	347647,00	0,20	TCG	348793,00	0,21
**GCC**	1816593,00	1,07	GGC	1817025,00	1,07	CGG	407720,00	0,24	CCG	402684,00	0,24
**CCA**	2964839,00	1,74	TGG	2983263,00	1,75	GGT	1892238,00	1,11	ACC	1901715,00	1,12
**CCT**	2869457,00	1,69	AGG	2860340,00	1,68	GGA	2542058,00	1,49	TCC	2518672,00	1,48
**CAC**	2449734,00	1,44	GTG	2461387,00	1,45	GTG*			CAC*		
**CGC**	352703,00	0,21	GCG	357470,00	0,21	GCG*			CGC*		
**CTC**	2729519,00	1,60	GAG	2734438,00	1,61	GAG*			CTC*		
**CCC**	2003923,00	1,18	GGG	2012173,00	1,18	GGG*			CCC*		

Quadruplet		f1	f2	fu
CGT	CGT(D)	0,24	1,39	1,63
	ACG(RC(D))	0,24	1,39	1,63
	GCA(C(D))	1,39	0,24	1,63
	TGC(R(D))	1,39	0,24	1,63
GTC	GTC(D)	0,91	1,92	2,84
	GAC(RC(D))	0,92	1,91	2,83
	CAG(C(D))	1,92	0,91	2,84
	CTG(R(D))	1,91	0,92	2,83
GCT	GCT(D)	1,33	0,20	1,54
	AGC(RC(D))	1,34	0,21	1,55
	CGA(C(D))	0,20	1,33	1,54
	TCG(R(D))	0,21	1,34	1,55
GCC	GCC(D)	1,07	0,24	1,31
	GGC(RC(D))	1,07	0,24	1,31
	CGG(C(D))	0,24	1,07	1,31
	CCG(R(D))	0,24	1,07	1,31
CCA	CCA(D)	1,74	1,11	2,86
	TGG(RC(D))	1,75	1,12	2,87
	GGT(C(D))	1,11	1,74	2,86
	ACC(R(D))	1,12	1,75	2,87
CCT	CCT(D)	1,69	1,49	3,18
	AGG(RC(D))	1,68	1,48	3,16
	GGA(C(D))	1,49	1,69	3,18
	TCC(R(D))	1,48	1,68	3,16
CAC	CAC(D)	0,72	0,72	1,44
	GTG(RC(D))	0,72	0,72	1,44
	GTG(C(D))	0,72	0,72	1,44
	CAC(R(D))	0,72	0,72	1,44
CGC	CGC(D)	0,10	0,11	0,21
	GCG(RC(D))	0,11	0,10	0,21
	GCG(C(D))	0,11	0,10	0,21
	CGC(R(D))	0,10	0,11	0,21
CTC	CTC(D)	0,80	0,80	1,61
	GAG(RC(D))	0,80	0,80	1,61
	GAG(C(D))	0,80	0,80	1,61
	CTC(R(D))	0,80	0,80	1,61
CCC	CCC(D)	0,59	0,59	1,18
	GGG(RC(D))	0,59	0,59	1,18
	GGG(C(D))	0,59	0,59	1,18
	CCC(R(D))	0,59	0,59	1,18

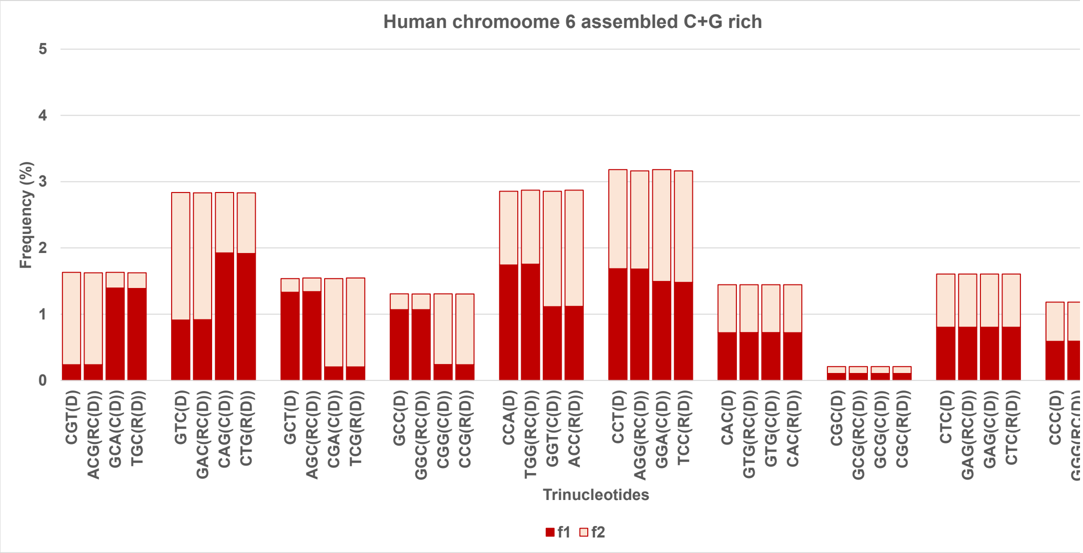

Human random chromomome 6

						C+G rich					
**D**	**f**	**%**	**RC**	**f**	**%**	**C**	**f**	**%**	**R**	**f**	**%**
**CGT**	528,00	0,27	ACG	520,00	0,26	GCA	2734,00	1,38	TGC	2818,00	1,42
**GTC**	1832,00	0,92	GAC	1914,00	0,96	CAG	3849,00	1,94	CTG	4060,00	2,05
**GCT**	2752,00	1,39	AGC	2751,00	1,39	CGA	450,00	0,23	TCG	450,00	0,23
**GCC**	2328,00	1,17	GGC	2258,00	1,14	CGG	534,00	0,27	CCG	529,00	0,27
**CCA**	3639,00	1,83	TGG	3667,00	1,85	GGT	2265,00	1,14	ACC	2337,00	1,18
**CCT**	3592,00	1,81	AGG	3404,00	1,72	GGA	3082,00	1,55	TCC	3095,00	1,56
**CAC**	2930,00	1,48	GTG	2953,00	1,49	GTG*			CAC*		
**CGC**	454,00	0,23	GCG	467,00	0,24	GCG*			CGC*		
**CTC**	3253,00	1,64	GAG	3364,00	1,70	GAG*			CTC*		
**CCC**	2526,00	1,27	GGG	2633,00	1,33	GGG*			CCC*		

Quadruplet		f1	f2	fu
CGT	CGT(D)	0,27	1,38	1,64
	ACG(RC(D))	0,26	1,42	1,68
	GCA(C(D))	1,38	0,27	1,64
	TGC(R(D))	1,42	0,26	1,68
GTC	GTC(D)	0,92	1,94	2,86
	GAC(RC(D))	0,96	2,05	3,01
	CAG(C(D))	1,94	0,92	2,86
	CTG(R(D))	2,05	0,96	3,01
GCT	GCT(D)	1,39	0,23	1,61
	AGC(RC(D))	1,39	0,23	1,61
	CGA(C(D))	0,23	1,39	1,61
	TCG(R(D))	0,23	1,39	1,61
GCC	GCC(D)	1,17	0,27	1,44
	GGC(RC(D))	1,14	0,27	1,40
	CGG(C(D))	0,27	1,17	1,44
	CCG(R(D))	0,27	1,14	1,40
CCA	CCA(D)	1,83	1,14	2,98
	TGG(RC(D))	1,85	1,18	3,03
	GGT(C(D))	1,14	1,83	2,98
	ACC(R(D))	1,18	1,85	3,03
CCT	CCT(D)	1,81	1,55	3,36
	AGG(RC(D))	1,72	1,56	3,28
	GGA(C(D))	1,55	1,81	3,36
	TCC(R(D))	1,56	1,72	3,28
CAC	CAC(D)	0,74	0,74	1,48
	GTG(RC(D))	0,74	0,74	1,48
	GTG(C(D))	0,74	0,74	1,48
	CAC(R(D))	0,74	0,74	1,48
CGC	CGC(D)	0,11	0,12	0,23
	GCG(RC(D))	0,12	0,11	0,23
	GCG(C(D))	0,12	0,11	0,23
	CGC(R(D))	0,11	0,12	0,23
CTC	CTC(D)	0,82	0,85	1,67
	GAG(RC(D))	0,85	0,82	1,67
	GAG(C(D))	0,85	0,82	1,67
	CTC(R(D))	0,82	0,85	1,67
CCC	CCC(D)	0,64	0,66	1,30
	GGG(RC(D))	0,66	0,64	1,30
	GGG(C(D))	0,66	0,64	1,30
	CCC(R(D))	0,64	0,66	1,30

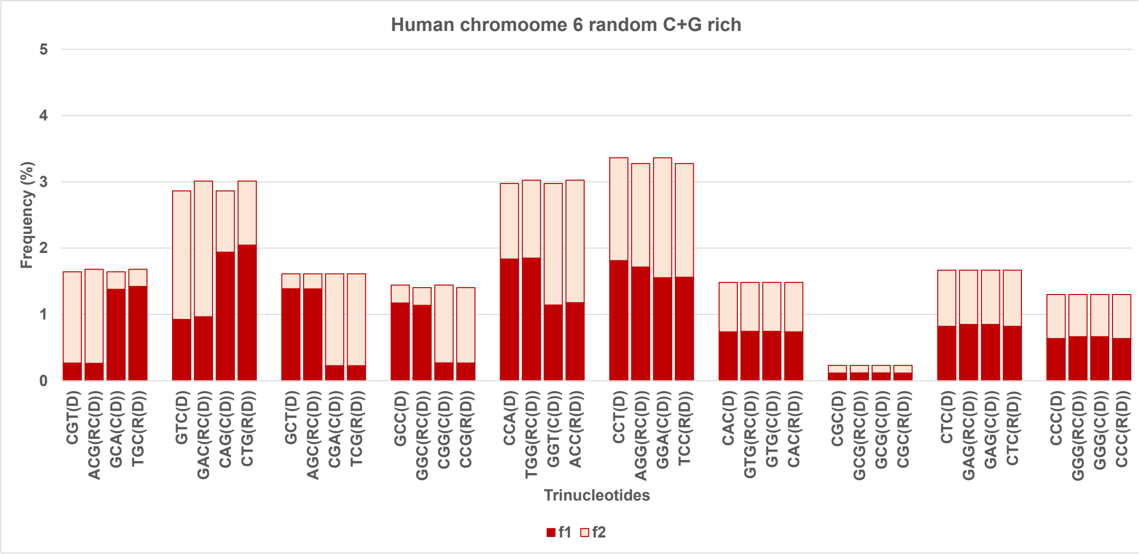

Human coding chromomome 6

C+G rich

D	f	%	RC	f	%	C	f	%	R	f	%
**CGT**	38061,00	0,43	ACG	42870,00	0,48	GCA	151033,00	1,69	TGC	152214,00	1,71
**GTC**	93181,00	1,04	GAC	113485,00	1,27	CAG	224932,00	2,52	CTG	219800,00	2,46
**GCT**	151059,00	1,69	AGC	162990,00	1,83	CGA	46017,00	0,52	TCG	39830,00	0,45
**GCC**	154064,00	1,73	GGC	142044,00	1,59	CGG	68120,00	0,76	CCG	68406,00	0,77
**CCA**	184173,00	2,06	TGG	192066,00	2,15	GGT	101430,00	1,14	ACC	125552,00	1,41
**CCT**	176486,00	1,98	AGG	169686,00	1,90	GGA	186398,00	2,09	TCC	149448,00	1,68
**CAC**	130606,00	1,46	GTG	147193,00	1,65	GTG*			CAC*		
**CGC**	57632,00	0,65	GCG	58724,00	0,66	GCG*			CGC*		
**CTC**	153492,00	1,72	GAG	182090,00	2,04	GAG*			CTC*		
**CCC**	160764,00	1,80	GGG	143602,00	1,61	GGG*			CCC*		

Quadruplet		f1	f2	fu
CGT	CGT(D)	0,43	1,69	2,12
	ACG(RC(D))	0,48	1,71	2,19
	GCA(C(D))	1,69	0,43	2,12
	TGC(R(D))	1,71	0,48	2,19
GTC	GTC(D)	1,04	2,52	3,57
	GAC(RC(D))	1,27	2,46	3,74
	CAG(C(D))	2,52	1,04	3,57
	CTG(R(D))	2,46	1,27	3,74
GCT	GCT(D)	1,69	0,52	2,21
	AGC(RC(D))	1,83	0,45	2,27
	CGA(C(D))	0,52	1,69	2,21
	TCG(R(D))	0,45	1,83	2,27
GCC	GCC(D)	1,73	0,76	2,49
	GGC(RC(D))	1,59	0,77	2,36
	CGG(C(D))	0,76	1,73	2,49
	CCG(R(D))	0,77	1,59	2,36
CCA	CCA(D)	2,06	1,14	3,20
	TGG(RC(D))	2,15	1,41	3,56
	GGT(C(D))	1,14	2,06	3,20
	ACC(R(D))	1,41	2,15	3,56
CCT	CCT(D)	1,98	2,09	4,07
	AGG(RC(D))	1,90	1,68	3,58
	GGA(C(D))	2,09	1,98	4,07
	TCC(R(D))	1,68	1,90	3,58
CAC	CAC(D)	0,73	0,83	1,56
	GTG(RC(D))	0,83	0,73	1,56
	GTG(C(D))	0,83	0,73	1,56
	CAC(R(D))	0,73	0,83	1,56
CGC	CGC(D)	0,32	0,33	0,65
	GCG(RC(D))	0,33	0,32	0,65
	GCG(C(D))	0,33	0,32	0,65
	CGC(R(D))	0,32	0,33	0,65
CTC	CTC(D)	0,86	1,02	1,88
	GAG(RC(D))	1,02	0,86	1,88
	GAG(C(D))	1,02	0,86	1,88
	CTC(R(D))	0,86	1,02	1,88
CCC	CCC(D)	0,90	0,81	1,71
	GGG(RC(D))	0,81	0,90	1,71
	GGG(C(D))	0,81	0,90	1,71
	CCC(R(D))	0,90	0,81	1,71

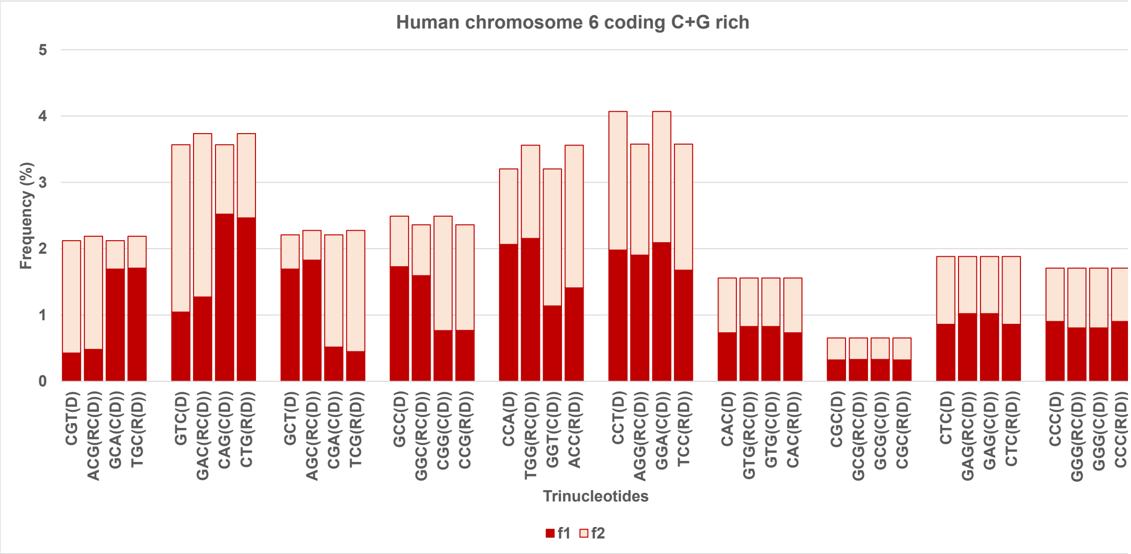

IIa

IIb

IIc

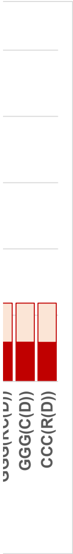

IIa

IIb

IIc

IIa

IIb

IIc

						A-T rich						
**D**	**f**	**%**	**RC**	**f**	**%**	**C**	**f**	**%**	**R**	**f**	**%**	
**ATG**	2888006,00	1,82	CAT	2905091,00	1,83	TAC	1775993,00	1,12	GTA	1798327,00	1,13	Ia
**TGA**	3103592,00	1,95	TCA	3154342,00	1,98	ACT	2561044,00	1,61	AGT	2573382,00	1,62	
**TAG**	2044274,00	1,29	CTA	2021161,00	1,27	ATC	2113619,00	1,33	GAT	2139629,00	1,35	
**TAA**	3305411,00	2,08	TTA	3301649,00	2,08	ATT	4018159,00	2,53	AAT	3979602,00	2,50	Ib
**AAC**	2357427,00	1,48	GTT	2336049,00	1,47	TTG	3059516,00	1,92	CAA	3004029,00	1,89	
**AAG**	3142493,00	1,98	CTT	3197957,00	2,01	TTC	3191000,00	2,01	GAA	3170083,00	1,99	
**ACA**	3214607,00	2,02	TGT	3234766,00	2,03	TGT*			ACA*			Ic
**ATA**	3277086,00	2,06	TAT	3272545,00	2,06	TAT*			ATA*			
**AGA**	3554554,00	2,24	TCT	3524383,00	2,22	TCT*			AGA*			
**AAA**	6174004,00	3,88	TTT	6278035,00	3,95	TTT*			AAA*			

Quadruplet		f1	f2	fu
ATG	ATG(D)	1,82	1,12	2,93
	CAT(RC(D))	1,83	1,13	2,96
	TAC(C(D))	1,12	1,82	2,93
	GTA(R(D))	1,13	1,83	2,96
TGA	TGA(D)	1,95	1,61	3,56
	TCA(RC(D))	1,98	1,62	3,60
	ACT(C(D))	1,61	1,95	3,56
	AGT(R(D))	1,62	1,98	3,60
TAG	TAG(D)	1,29	1,33	2,62
	CTA(RC(D))	1,27	1,35	2,62
	ATC(C(D))	1,33	1,29	2,62
	GAT(R(D))	1,35	1,27	2,62
TAA	TAA(D)	2,08	2,53	4,61
	TTA(RC(D))	2,08	2,50	4,58
	ATT(C(D))	2,53	2,08	4,61
	AAT(R(D))	2,50	2,08	4,58
AAC	AAC(D)	1,48	1,92	3,41
	GTT(RC(D))	1,47	1,89	3,36
	TTG(C(D))	1,92	1,48	3,41
	CAA(R(D))	1,89	1,47	3,36
AAG	AAG(D)	1,98	2,01	3,98
	CTT(RC(D))	2,01	1,99	4,01
	TTC(C(D))	2,01	1,98	3,98
	GAA(R(D))	1,99	2,01	4,01
ACA	ACA(D)	1,01	1,02	2,03
	TGT(RC(D))	1,02	1,01	2,03
	TGT(C(D))	1,02	1,01	2,03
	ACA(R(D))	1,01	1,02	2,03
ATA	ATA(D)	1,03	1,03	2,06
	TAT(RC(D))	1,03	1,03	2,06
	TAT(C(D))	1,03	1,03	2,06
	ATA(R(D))	1,03	1,03	2,06
AGA	AGA(D)	1,12	1,11	2,23
	TCT(RC(D))	1,11	1,12	2,23
	TCT(C(D))	1,11	1,12	2,23
	AGA(R(D))	1,12	1,11	2,23
AAA	AAA(D)	1,94	1,97	3,92
	TTT(RC(D))	1,97	1,94	3,92
	TTT(C(D))	1,97	1,94	3,92
	AAA(R(D))	1,94	1,97	3,92

Composition of DNA sequence in assembled chromosome 7

Base	No.of bases	%
A	47058248	29,53%
C	32317984	20,28%
G	32378859	20,32%
T	47215040	29,63%
N	375842	0,24%
Sum	159345973	100,00%

% of coding bases from complete assembled chromosome 1 (including N bases)

**5,07%**

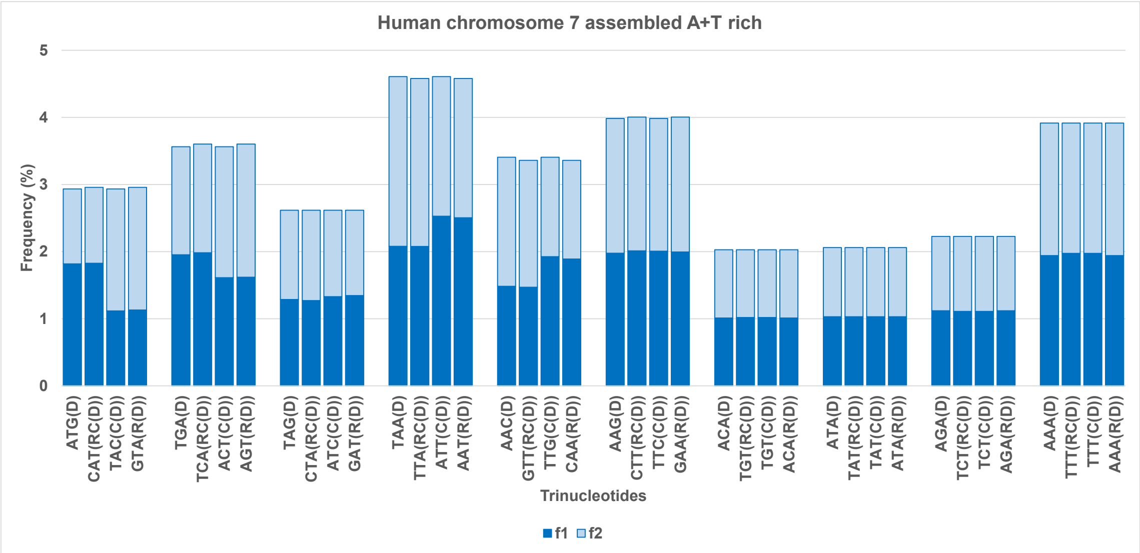

Human random chromomome 7

A-T rich

D	f	%	RC	f	%	C	f	%	R	f	%	
**ATG**	3578,00	1,79	CAT	3529,00	1,77	TAC	2097,00	1,05	GTA	2204,00	1,11	Ia
**TGA**	3903,00	1,96	TCA	4069,00	2,04	ACT	3236,00	1,62	AGT	3279,00	1,64	
**TAG**	2585,00	1,30	CTA	2514,00	1,26	ATC	2690,00	1,35	GAT	2672,00	1,34	
**TAA**	3956,00	1,98	TTA	3934,00	1,97	ATT	4852,00	2,43	AAT	4906,00	2,46	Ib
**AAC**	3010,00	1,51	GTT	2894,00	1,45	TTG	3938,00	1,98	CAA	3710,00	1,86	
**AAG**	3894,00	1,95	CTT	4083,00	2,05	TTC	3958,00	1,99	GAA	4143,00	2,08	
**ACA**	4018,00	2,02	TGT	4138,00	2,08	TGT*			ACA*			Ic
**ATA**	3973,00	1,99	TAT	3986,00	2,00	TAT*			ATA*			
**AGA**	4533,00	2,27	TCT	4337,00	2,18	TCT*			AGA*			
**AAA**	7799,00	3,91	TTT	7877,00	3,95	TTT*			AAA*			

Quadruplet		f1	f2	fu
ATG	ATG(D)	1,79	1,05	2,85
	CAT(RC(D))	1,77	1,11	2,88
	TAC(C(D))	1,05	1,79	2,85
	GTA(R(D))	1,11	1,77	2,88
TGA	TGA(D)	1,96	1,62	3,58
	TCA(RC(D))	2,04	1,64	3,69
	ACT(C(D))	1,62	1,96	3,58
	AGT(R(D))	1,64	2,04	3,69
TAG	TAG(D)	1,30	1,35	2,65
	CTA(RC(D))	1,26	1,34	2,60
	ATC(C(D))	1,35	1,30	2,65
	GAT(R(D))	1,34	1,26	2,60
TAA	TAA(D)	1,98	2,43	4,42
	TTA(RC(D))	1,97	2,46	4,43
	ATT(C(D))	2,43	1,98	4,42
	AAT(R(D))	2,46	1,97	4,43
AAC	AAC(D)	1,51	1,98	3,48
	GTT(RC(D))	1,45	1,86	3,31
	TTG(C(D))	1,98	1,51	3,48
	CAA(R(D))	1,86	1,45	3,31
AAG	AAG(D)	1,95	1,99	3,94
	CTT(RC(D))	2,05	2,08	4,13
	TTC(C(D))	1,99	1,95	3,94
	GAA(R(D))	2,08	2,05	4,13
ACA	ACA(D)	1,01	1,04	2,05
	TGT(RC(D))	1,04	1,01	2,05
	TGT(C(D))	1,04	1,01	2,05
	ACA(R(D))	1,01	1,04	2,05
ATA	ATA(D)	1,00	1,00	2,00
	TAT(RC(D))	1,00	1,00	2,00
	TAT(C(D))	1,00	1,00	2,00
	ATA(R(D))	1,00	1,00	1,99
AGA	AGA(D)	1,14	1,09	2,22
	TCT(RC(D))	1,09	1,14	2,22
	TCT(C(D))	1,09	1,14	2,22
	AGA(R(D))	1,14	1,09	2,22
AAA	AAA(D)	1,96	1,98	3,93
	TTT(RC(D))	1,98	1,96	3,93
	TTT(C(D))	1,98	1,96	3,93
	AAA(R(D))	1,96	1,98	3,93

Composition of DNA sequence in random chromosome 7

Base	No.of bases	%
A	58914	29,46%
C	40349	20,17%
G	41342	20,67%
T	58795	29,40%
N	600	0,30%
Sum	200000	100,00%

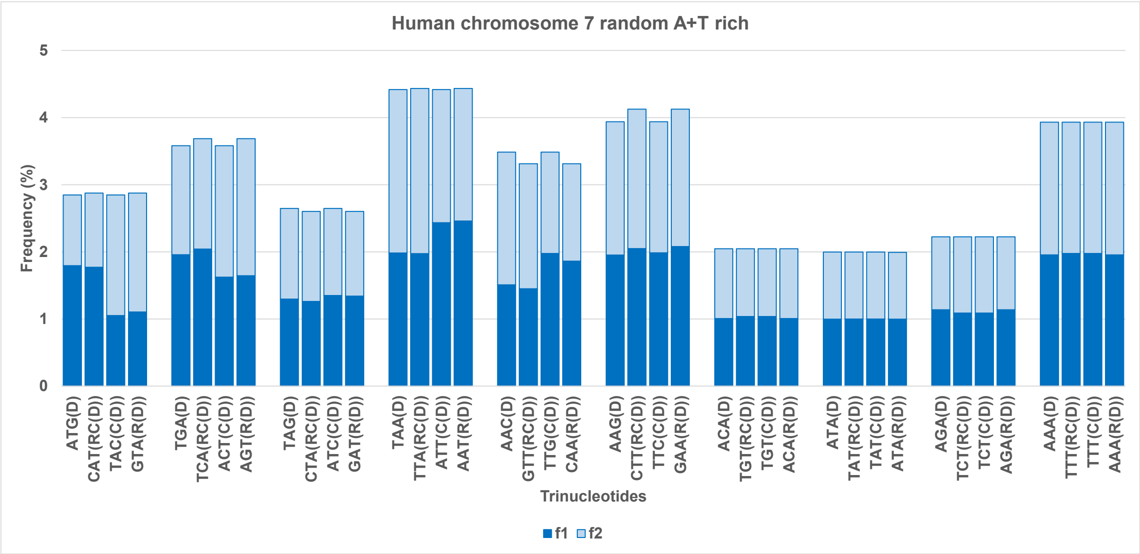

Human coding chromomome 7

A-T rich

D	f	%	RC	f	%	C	f	%	R	f	%	
**ATG**	139810,00	1,73	CAT	125878,00	1,56	TAC	78905,00	0,98	GTA	66271,00	0,82	Ia
**TGA**	153803,00	1,90	TCA	142182,00	1,76	ACT	114232,00	1,41	AGT	114702,00	1,42	
**TAG**	62604,00	0,77	CTA	77870,00	0,96	ATC	100239,00	1,24	GAT	106537,00	1,32	
**TAA**	98483,00	1,22	TTA	102263,00	1,26	ATT	132087,00	1,63	AAT	133309,00	1,65	Ib
**AAC**	109847,00	1,36	GTT	97148,00	1,20	TTG	128817,00	1,59	CAA	140055,00	1,73	
**AAG**	170809,00	2,11	CTT	140845,00	1,74	TTC	139000,00	1,72	GAA	175426,00	2,17	
**ACA**	142730,00	1,77	TGT	142947,00	1,77	TGT*			ACA*			Ic
**ATA**	94030,00	1,16	TAT	100442,00	1,24	TAT*			ATA*			
**AGA**	184333,00	2,28	TCT	142757,00	1,77	TCT*			AGA*			
**AAA**	214676,00	2,66	TTT	195505,00	2,42	TTT*			AAA*			

Quadruplet		f1	f2	fu
ATG	ATG(D)	1,73	0,98	2,71
	CAT(RC(D))	1,56	0,82	2,38
	TAC(C(D))	0,98	1,73	2,71
	GTA(R(D))	0,82	1,56	2,38
TGA	TGA(D)	1,90	1,41	3,32
	TCA(RC(D))	1,76	1,42	3,18
	ACT(C(D))	1,41	1,90	3,32
	AGT(R(D))	1,42	1,76	3,18
TAG	TAG(D)	0,77	1,24	2,01
	CTA(RC(D))	0,96	1,32	2,28
	ATC(C(D))	1,24	0,77	2,01
	GAT(R(D))	1,32	0,96	2,28
TAA	TAA(D)	1,22	1,63	2,85
	TTA(RC(D))	1,26	1,65	2,91
	ATT(C(D))	1,63	1,22	2,85
	AAT(R(D))	1,65	1,26	2,91
AAC	AAC(D)	1,36	1,59	2,95
	GTT(RC(D))	1,20	1,73	2,93
	TTG(C(D))	1,59	1,36	2,95
	CAA(R(D))	1,73	1,20	2,93
AAG	AAG(D)	2,11	1,72	3,83
	CTT(RC(D))	1,74	2,17	3,91
	TTC(C(D))	1,72	2,11	3,83
	GAA(R(D))	2,17	1,74	3,91
ACA	ACA(D)	0,88	0,88	1,77
	TGT(RC(D))	0,88	0,88	1,77
	TGT(C(D))	0,88	0,88	1,77
	ACA(R(D))	0,88	0,88	1,77
ATA	ATA(D)	0,58	0,62	1,20
	TAT(RC(D))	0,62	0,58	1,20
	TAT(C(D))	0,62	0,58	1,20
	ATA(R(D))	0,58	0,58	1,16
AGA	AGA(D)	1,14	0,88	2,02
	TCT(RC(D))	0,88	1,14	2,02
	TCT(C(D))	0,88	1,14	2,02
	AGA(R(D))	1,14	0,88	2,02
AAA	AAA(D)	1,33	1,21	2,54
	TTT(RC(D))	1,21	1,33	2,54
	TTT(C(D))	1,21	1,33	2,54
	AAA(R(D))	1,33	1,21	2,54

Composition of DNA sequence in coding chromosome 7

Base	No.of bases	%
A	2123073	26,26%
C	1974295	24,42%
G	1995862	24,68%
T	1992099	24,64%
N	0	0,00%
Sum	8085329	100,00%

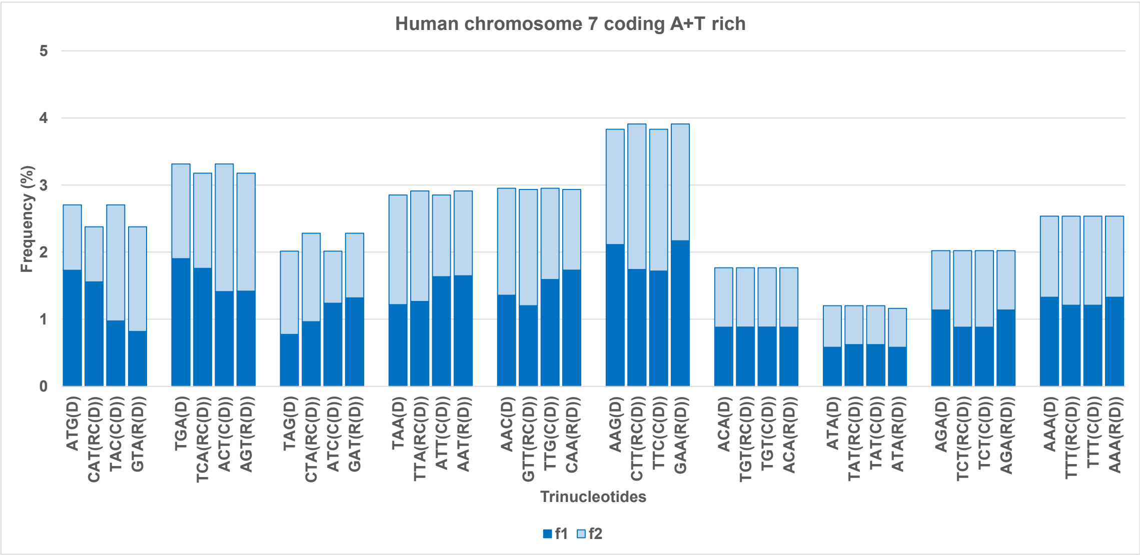

C+G rich											
**D**	**f**	**%**	**RC**	**f**	**%**	**C**	**f**	**%**	**R**	**f**	**%**
**CGT**	422629,00	0,27	ACG	418797,00	0,26	GCA	2286375,00	1,44	TGC	2302084,00	1,45
**GTC**	1474590,00	0,93	GAC	1492407,00	0,94	CAG	3222098,00	2,03	CTG	3165321,00	1,99
**GCT**	2187784,00	1,38	AGC	2193159,00	1,38	CGA	361735,00	0,23	TCG	367564,00	0,23
**GCC**	1864958,00	1,17	GGC	1852904,00	1,17	CGG	453163,00	0,29	CCG	442135,00	0,28
**CCA**	2873362,00	1,81	TGG	2897590,00	1,82	GGT	1803380,00	1,13	ACC	1828848,00	1,15
**CCT**	2780597,00	1,75	AGG	2763448,00	1,74	GGA	2457917,00	1,55	TCC	2402288,00	1,51
**CAC**	2397469,00	1,51	GTG	2425192,00	1,53	GTG*			CAC*		
**CGC**	385298,00	0,24	GCG	394329,00	0,25	GCG*			CGC*		
**CTC**	2669372,00	1,68	GAG	2675677,00	1,68	GAG*			CTC*		
**CCC**	2016562,00	1,27	GGG	2019246,00	1,27	GGG*			CCC*		

Quadruplet		f1	f2	fu
CGT	CGT(D)	0,27	1,44	1,70
	ACG(RC(D))	0,26	1,45	1,71
	GCA(C(D))	1,44	0,27	1,70
	TGC(R(D))	1,45	0,26	1,71
GTC	GTC(D)	0,93	2,03	2,95
	GAC(RC(D))	0,94	1,99	2,93
	CAG(C(D))	2,03	0,93	2,95
	CTG(R(D))	1,99	0,94	2,93
GCT	GCT(D)	1,38	0,23	1,60
	AGC(RC(D))	1,38	0,23	1,61
	CGA(C(D))	0,23	1,38	1,60
	TCG(R(D))	0,23	1,38	1,61
GCC	GCC(D)	1,17	0,29	1,46
	GGC(RC(D))	1,17	0,28	1,44
	CGG(C(D))	0,29	1,17	1,46
	CCG(R(D))	0,28	1,17	1,44
CCA	CCA(D)	1,81	1,13	2,94
	TGG(RC(D))	1,82	1,15	2,97
	GGT(C(D))	1,13	1,81	2,94
	ACC(R(D))	1,15	1,82	2,97
CCT	CCT(D)	1,75	1,55	3,30
	AGG(RC(D))	1,74	1,51	3,25
	GGA(C(D))	1,55	1,75	3,30
	TCC(R(D))	1,51	1,74	3,25
CAC	CAC(D)	0,75	0,76	1,52
	GTG(RC(D))	0,76	0,75	1,52
	GTG(C(D))	0,76	0,75	1,52
	CAC(R(D))	0,75	0,76	1,52
CGC	CGC(D)	0,12	0,12	0,25
	GCG(RC(D))	0,12	0,12	0,25
	GCG(C(D))	0,12	0,12	0,25
	CGC(R(D))	0,12	0,12	0,25
CTC	CTC(D)	0,84	0,84	1,68
	GAG(RC(D))	0,84	0,84	1,68
	GAG(C(D))	0,84	0,84	1,68
	CTC(R(D))	0,84	0,84	1,68
CCC	CCC(D)	0,63	0,64	1,27
	GGG(RC(D))	0,64	0,63	1,27
	GGG(C(D))	0,64	0,63	1,27
	CCC(R(D))	0,63	0,64	1,27

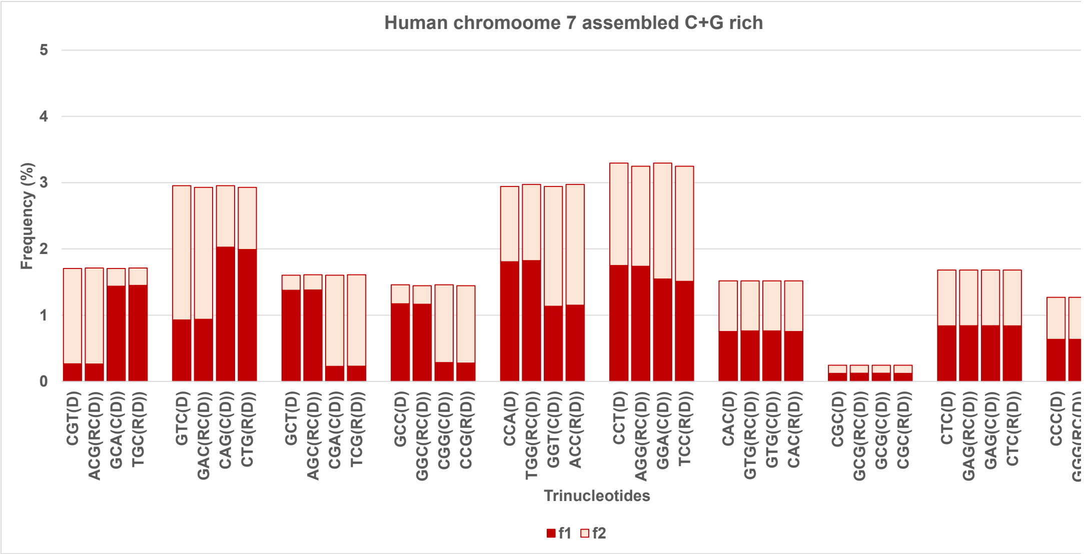

Human random chromomome 7

C+G rich											
**D**	f	%	RC	f	%	C	f	%	R	f	%
**CGT**	556,00	0,28	ACG	526,00	0,26	GCA	2902,00	1,46	TGC	2890,00	1,45
**GTC**	1901,00	0,95	GAC	1883,00	0,94	CAG	4202,00	2,11	CTG	3959,00	1,99
**GCT**	2777,00	1,39	AGC	2829,00	1,42	CGA	462,00	0,23	TCG	443,00	0,22
**GCC**	2307,00	1,16	GGC	2363,00	1,19	CGG	608,00	0,30	CCG	561,00	0,28
**CCA**	3533,00	1,77	TGG	3754,00	1,88	GGT	2238,00	1,12	ACC	2291,00	1,15
**CCT**	3431,00	1,72	AGG	3498,00	1,75	GGA	3259,00	1,63	TCC	2927,00	1,47
**CAC**	3081,00	1,55	GTG	3211,00	1,61	GTG*			CAC*		
**CGC**	437,00	0,22	GCG	533,00	0,27	GCG*			CGC*		
**CTC**	3227,00	1,62	GAG	3459,00	1,73	GAG*			CTC*		
**CCC**	2456,00	1,23	GGG	2593,00	1,30	GGG*			CCC*		

Quadruplet		f1	f2	fu
CGT	CGT(D)	0,28	1,46	1,73
	ACG(RC(D))	0,26	1,45	1,71
	GCA(C(D))	1,46	0,28	1,73
	TGC(R(D))	1,45	0,26	1,71
GTC	GTC(D)	0,95	2,11	3,06
	GAC(RC(D))	0,94	1,99	2,93
	CAG(C(D))	2,11	0,95	3,06
	CTG(R(D))	1,99	0,94	2,93
GCT	GCT(D)	1,39	0,23	1,62
	AGC(RC(D))	1,42	0,22	1,64
	CGA(C(D))	0,23	1,39	1,62
	TCG(R(D))	0,22	1,42	1,64
GCC	GCC(D)	1,16	0,30	1,46
	GGC(RC(D))	1,19	0,28	1,47
	CGG(C(D))	0,30	1,16	1,46
	CCG(R(D))	0,28	1,19	1,47
CCA	CCA(D)	1,77	1,12	2,89
	TGG(RC(D))	1,88	1,15	3,03
	GGT(C(D))	1,12	1,77	2,89
	ACC(R(D))	1,15	1,88	3,03
CCT	CCT(D)	1,72	1,63	3,36
	AGG(RC(D))	1,75	1,47	3,22
	GGA(C(D))	1,63	1,72	3,36
	TCC(R(D))	1,47	1,75	3,22
CAC	CAC(D)	0,77	0,81	1,58
	GTG(RC(D))	0,81	0,77	1,58
	GTG(C(D))	0,81	0,77	1,58
	CAC(R(D))	0,77	0,81	1,58
CGC	CGC(D)	0,11	0,13	0,24
	GCG(RC(D))	0,13	0,11	0,24
	GCG(C(D))	0,13	0,11	0,24
	CGC(R(D))	0,11	0,13	0,24
CTC	CTC(D)	0,81	0,87	1,68
	GAG(RC(D))	0,87	0,81	1,68
	GAG(C(D))	0,87	0,81	1,68
	CTC(R(D))	0,81	0,87	1,68
CCC	CCC(D)	0,62	0,65	1,27
	GGG(RC(D))	0,65	0,62	1,27
	GGG(C(D))	0,65	0,62	1,27
	CCC(R(D))	0,62	0,65	1,27

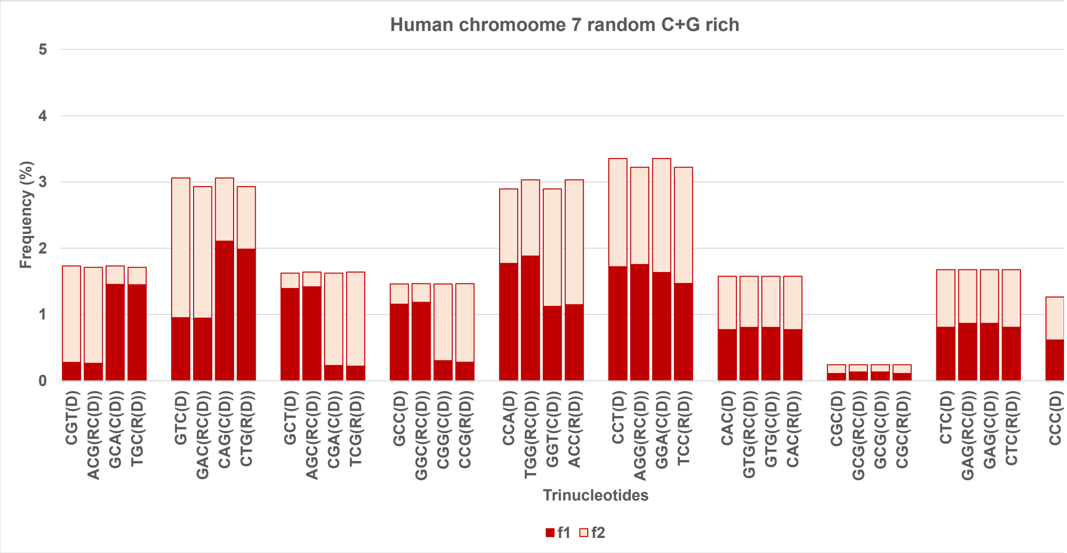

Human coding chromomome 7

C+G rich											
**D**	**f**	**%**	**RC**	**f**	**%**	**C**	**f**	**%**	**R**	**f**	**%**
**CGT**	40438,00	0,50	ACG	44732,00	0,55	GCA	141170,00	1,75	TGC	142494,00	1,76
**GTC**	86280,00	1,07	GAC	104525,00	1,29	CAG	207207,00	2,56	CTG	208589,00	2,58
**GCT**	144113,00	1,78	AGC	155188,00	1,92	CGA	46124,00	0,57	TCG	41232,00	0,51
**GCC**	156859,00	1,94	GGC	144595,00	1,79	CGG	74067,00	0,92	CCG	74530,00	0,92
**CCA**	173592,00	2,15	TGG	178675,00	2,21	GGT	92315,00	1,14	ACC	118117,00	1,46
**CCT**	168844,00	2,09	AGG	154231,00	1,91	GGA	170063,00	2,10	TCC	141990,00	1,76
**CAC**	126534,00	1,56	GTG	140703,00	1,74	GTG*			CAC*		
**CGC**	64766,00	0,80	GCG	64901,00	0,80	GCG*			CGC*		
**CTC**	142642,00	1,76	GAG	167834,00	2,08	GAG*			CTC*		
**CCC**	162314,00	2,01	GGG	137121,00	1,70	GGG*			CCC*		

Quadruplet		f1	f2	fu
CGT	CGT(D)	0,50	1,75	2,25
	ACG(RC(D))	0,55	1,76	2,32
	GCA(C(D))	1,75	0,50	2,25
	TGC(R(D))	1,76	0,55	2,32
GTC	GTC(D)	1,07	2,56	3,63
	GAC(RC(D))	1,29	2,58	3,87
	CAG(C(D))	2,56	1,07	3,63
	CTG(R(D))	2,58	1,29	3,87
GCT	GCT(D)	1,78	0,57	2,35
	AGC(RC(D))	1,92	0,51	2,43
	CGA(C(D))	0,57	1,78	2,35
	TCG(R(D))	0,51	1,92	2,43
GCC	GCC(D)	1,94	0,92	2,86
	GGC(RC(D))	1,79	0,92	2,71
	CGG(C(D))	0,92	1,94	2,86
	CCG(R(D))	0,92	1,79	2,71
CCA	CCA(D)	2,15	1,14	3,29
	TGG(RC(D))	2,21	1,46	3,67
	GGT(C(D))	1,14	2,15	3,29
	ACC(R(D))	1,46	2,21	3,67
CCT	CCT(D)	2,09	2,10	4,19
	AGG(RC(D))	1,91	1,76	3,66
	GGA(C(D))	2,10	2,09	4,19
	TCC(R(D))	1,76	1,91	3,66
CAC	CAC(D)	0,78	0,87	1,65
	GTG(RC(D))	0,87	0,78	1,65
	GTG(C(D))	0,87	0,78	1,65
	CAC(R(D))	0,78	0,87	1,65
CGC	CGC(D)	0,40	0,40	0,80
	GCG(RC(D))	0,40	0,40	0,80
	GCG(C(D))	0,40	0,40	0,80
	CGC(R(D))	0,40	0,40	0,80
CTC	CTC(D)	0,88	1,04	1,92
	GAG(RC(D))	1,04	0,88	1,92
	GAG(C(D))	1,04	0,88	1,92
	CTC(R(D))	0,88	1,04	1,92
CCC	CCC(D)	1,00	0,85	1,85
	GGG(RC(D))	0,85	1,00	1,85
	GGG(C(D))	0,85	1,00	1,85
	CCC(R(D))	1,00	0,85	1,85

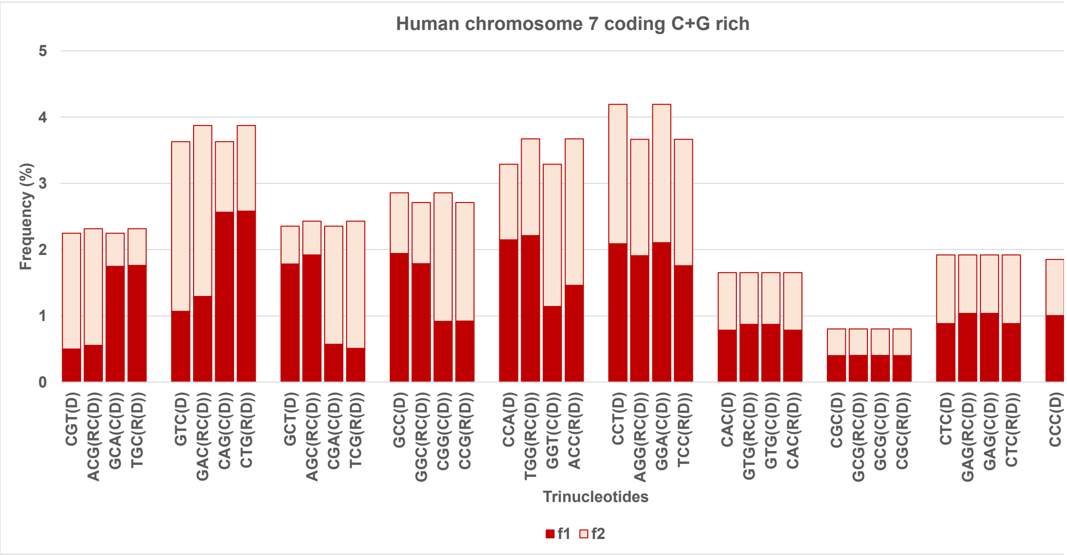

IIa

IIb

IIc

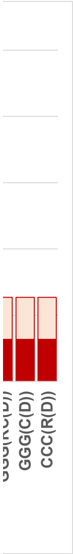

IIa

IIb

IIc

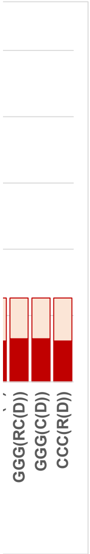

IIa

IIb

IIc

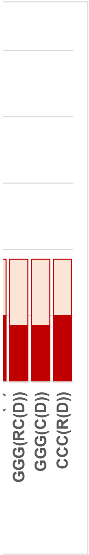
GGG(RC(D))
GGG(C(D))
CCC(R(D))

Human assembled chromomome 8

A-T rich												
**D**	**f**	**%**	**RC**	**f**	**%**	**C**	**f**	**%**	**R**	**f**	**%**	
**ATG**	2703850,00	1,87	CAT	2719664,00	1,88	TAC	1656965,00	1,14	GTA	1665280,00	1,15	Ia
**TGA**	2870965,00	1,98	TCA	2866345,00	1,98	ACT	2348614,00	1,62	AGT	2343218,00	1,62	
**TAG**	1896087,00	1,31	CTA	1891668,00	1,31	ATC	1962506,00	1,36	GAT	1964097,00	1,36	
**TAA**	3088882,00	2,13	TTA	3082124,00	2,13	ATT	3722924,00	2,57	AAT	3708381,00	2,56	Ib
**AAC**	2169193,00	1,50	GTT	2143097,00	1,48	TTG	2801796,00	1,94	CAA	2761609,00	1,91	
**AAG**	2930387,00	2,02	CTT	2940802,00	2,03	TTC	2922902,00	2,02	GAA	2957468,00	2,04	
**ACA**	2976704,00	2,06	TGT	2963394,00	2,05	TGT*			ACA*			Ic
**ATA**	3093321,00	2,14	TAT	3090459,00	2,13	TAT*			ATA*			
**AGA**	3246710,00	2,24	TCT	3240084,00	2,24	TCT*			AGA*			
**AAA**	5664156,00	3,91	TTT	5682429,00	3,93	TTT*			AAA*			

Quadruplet		f1	f2	fu
ATG	ATG(D)	1,87	1,14	3,01
	CAT(RC(D))	1,88	1,15	3,03
	TAC(C(D))	1,14	1,87	3,01
	GTA(R(D))	1,15	1,88	3,03
TGA	TGA(D)	1,98	1,62	3,61
	TCA(RC(D))	1,98	1,62	3,60
	ACT(C(D))	1,62	1,98	3,61
	AGT(R(D))	1,62	1,98	3,60
TAG	TAG(D)	1,31	1,36	2,67
	CTA(RC(D))	1,31	1,36	2,66
	ATC(C(D))	1,36	1,31	2,67
	GAT(R(D))	1,36	1,31	2,66
TAA	TAA(D)	2,13	2,57	4,71
	TTA(RC(D))	2,13	2,56	4,69
	ATT(C(D))	2,57	2,13	4,71
	AAT(R(D))	2,56	2,13	4,69
AAC	AAC(D)	1,50	1,94	3,43
	GTT(RC(D))	1,48	1,91	3,39
	TTG(C(D))	1,94	1,50	3,43
	CAA(R(D))	1,91	1,48	3,39
AAG	AAG(D)	2,02	2,02	4,04
	CTT(RC(D))	2,03	2,04	4,07
	TTC(C(D))	2,02	2,02	4,04
	GAA(R(D))	2,04	2,03	4,07
ACA	ACA(D)	1,03	1,02	2,05
	TGT(RC(D))	1,02	1,03	2,05
	TGT(C(D))	1,02	1,03	2,05
	ACA(R(D))	1,03	1,02	2,05
ATA	ATA(D)	1,07	1,07	2,14
	TAT(RC(D))	1,07	1,07	2,14
	TAT(C(D))	1,07	1,07	2,14
	ATA(R(D))	1,07	1,07	2,14
AGA	AGA(D)	1,12	1,12	2,24
	TCT(RC(D))	1,12	1,12	2,24
	TCT(C(D))	1,12	1,12	2,24
	AGA(R(D))	1,12	1,12	2,24
AAA	AAA(D)	1,96	1,96	3,92
	TTT(RC(D))	1,96	1,96	3,92
	TTT(C(D))	1,96	1,96	3,92
	AAA(R(D))	1,96	1,96	3,92

Composition of DNA sequence in assembled chromosome 8

Base	No.of bases	%
A	43333530	29,86%
C	29030173	20,00%
G	29103787	20,05%
T	43300646	29,83%
N	370500	0,26%
Sum	145138636	100,00%

% of coding bases from complete assembled chromosome 1 (including N bases)

**4,35%**

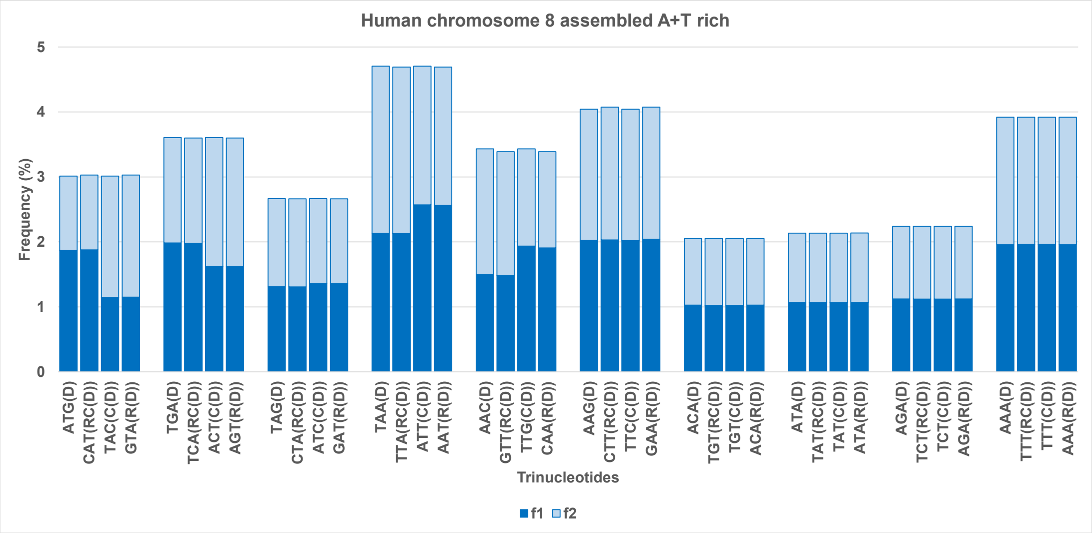

Human random chromomome 8

A-T rich

D	f	%	RC	f	%	C	f	%	R	f	%	
**ATG**	3658,00	1,83	CAT	3749,00	1,88	TAC	2241,00	1,12	GTA	2263,00	1,13	Ia
**TGA**	3946,00	1,98	TCA	3957,00	1,98	ACT	3155,00	1,58	AGT	3190,00	1,60	
**TAG**	2603,00	1,31	CTA	2556,00	1,28	ATC	2801,00	1,40	GAT	2804,00	1,41	
**TAA**	3973,00	1,99	TTA	4029,00	2,02	ATT	5022,00	2,52	AAT	4897,00	2,46	Ib
**AAC**	2934,00	1,47	GTT	2940,00	1,47	TTG	3901,00	1,96	CAA	3716,00	1,86	
**AAG**	3951,00	1,98	CTT	4012,00	2,01	TTC	4043,00	2,03	GAA	4094,00	2,05	
**ACA**	3987,00	2,00	TGT	4159,00	2,09	TGT*			ACA*			Ic
**ATA**	3965,00	1,99	TAT	3997,00	2,00	TAT*			ATA*			
**AGA**	4476,00	2,24	TCT	4412,00	2,21	TCT*			AGA*			
**AAA**	7671,00	3,85	TTT	7664,00	3,84	TTT*			AAA*			

Quadruplet		f1	f2	fu
	ATG(D)	1,83	1,12	2,96
ATG	CAT(RC(D))	1,88	1,13	3,02
	TAC(C(D))	1,12	1,83	2,96
	GTA(R(D))	1,13	1,88	3,02
	TGA(D)	1,98	1,58	3,56
TGA	TCA(RC(D))	1,98	1,60	3,58
	ACT(C(D))	1,58	1,98	3,56
	AGT(R(D))	1,60	1,98	3,58
	TAG(D)	1,31	1,40	2,71
TAG	CTA(RC(D))	1,28	1,41	2,69
	ATC(C(D))	1,40	1,31	2,71
	GAT(R(D))	1,41	1,28	2,69
	TAA(D)	1,99	2,52	4,51
TAA	TTA(RC(D))	2,02	2,46	4,48
	ATT(C(D))	2,52	1,99	4,51
	AAT(R(D))	2,46	2,02	4,48
	AAC(D)	1,47	1,96	3,43
AAC	GTT(RC(D))	1,47	1,86	3,34
	TTG(C(D))	1,96	1,47	3,43
	CAA(R(D))	1,86	1,47	3,34
	AAG(D)	1,98	2,03	4,01
AAG	CTT(RC(D))	2,01	2,05	4,07
	TTC(C(D))	2,03	1,98	4,01
	GAA(R(D))	2,05	2,01	4,07
	ACA(D)	1,00	1,04	2,04
ACA	TGT(RC(D))	1,04	1,00	2,04
	TGT(C(D))	1,04	1,00	2,04
	ACA(R(D))	1,00	1,04	2,04
	ATA(D)	0,99	1,00	2,00
ATA	TAT(RC(D))	1,00	0,99	2,00
	TAT(C(D))	1,00	0,99	2,00
	ATA(R(D))	0,99	0,99	1,99
	AGA(D)	1,12	1,11	2,23
AGA	TCT(RC(D))	1,11	1,12	2,23
	TCT(C(D))	1,11	1,12	2,23
	AGA(R(D))	1,12	1,11	2,23
	AAA(D)	1,92	1,92	3,85
AAA	TTT(RC(D))	1,92	1,92	3,85
	TTT(C(D))	1,92	1,92	3,85
	AAA(R(D))	1,92	1,92	3,85

Composition of DNA sequence in random chromosome 8

Base	No.of bases	%
A	58786	29,39%
C	40548	20,27%
G	40966	20,48%
T	59100	29,55%
N	600	0,30%
Sum	200000	100,00%

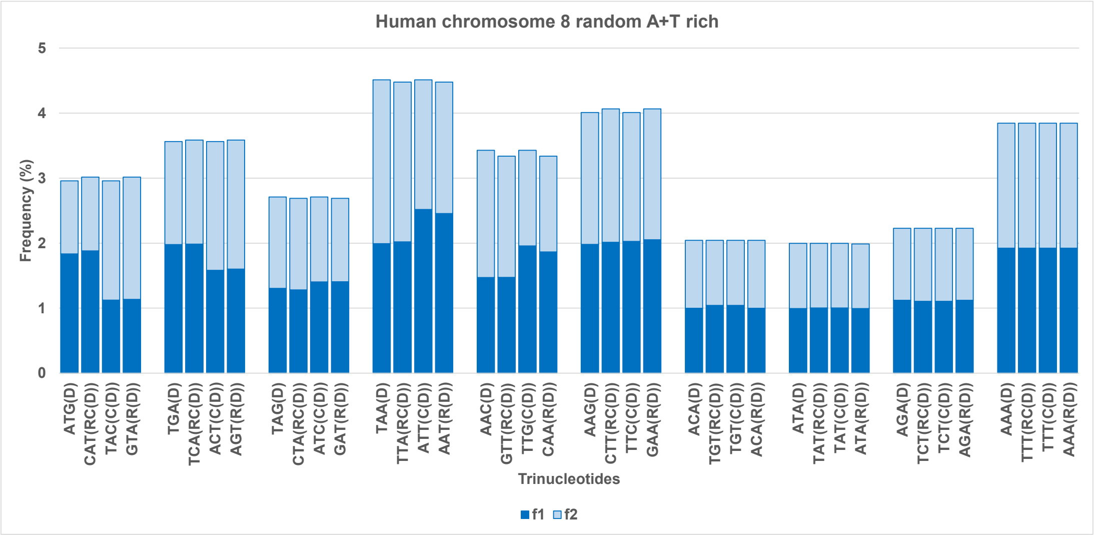

Human coding chromomome 8

A-T rich

D	f	%	RC	f	%	C	f	%	R	f	%	
**ATG**	113301,00	1,80	CAT	97428,00	1,54	TAC	62798,00	0,99	GTA	55200,00	0,87	Ia
**TGA**	121400,00	1,92	TCA	108116,00	1,71	ACT	89366,00	1,42	AGT	90598,00	1,44	
**TAG**	51827,00	0,82	CTA	61556,00	0,98	ATC	77667,00	1,23	GAT	85573,00	1,36	
**TAA**	82888,00	1,31	TTA	87656,00	1,39	ATT	109628,00	1,74	AAT	110696,00	1,75	Ib
**AAC**	85213,00	1,35	GTT	79263,00	1,26	TTG	105215,00	1,67	CAA	108376,00	1,72	
**AAG**	135852,00	2,15	CTT	111800,00	1,77	TTC	107820,00	1,71	GAA	140498,00	2,23	
**ACA**	109349,00	1,73	TGT	113312,00	1,80	TGT*			ACA*			Ic
**ATA**	78018,00	1,24	TAT	84917,00	1,35	TAT*			ATA*			
**AGA**	146133,00	2,32	TCT	111695,00	1,77	TCT*			AGA*			
**AAA**	175039,00	2,77	TTT	164102,00	2,60	TTT*			AAA*			

Quadruplet		f1	f2	fu
ATG	ATG(D)	1,80	0,99	2,79
	CAT(RC(D))	1,54	0,87	2,42
	TAC(C(D))	0,99	1,80	2,79
	GTA(R(D))	0,87	1,54	2,42
TGA	TGA(D)	1,92	1,42	3,34
	TCA(RC(D))	1,71	1,44	3,15
	ACT(C(D))	1,42	1,92	3,34
	AGT(R(D))	1,44	1,71	3,15
TAG	TAG(D)	0,82	1,23	2,05
	CTA(RC(D))	0,98	1,36	2,33
	ATC(C(D))	1,23	0,82	2,05
	GAT(R(D))	1,36	0,98	2,33
TAA	TAA(D)	1,31	1,74	3,05
	TTA(RC(D))	1,39	1,75	3,14
	ATT(C(D))	1,74	1,31	3,05
	AAT(R(D))	1,75	1,39	3,14
AAC	AAC(D)	1,35	1,67	3,02
	GTT(RC(D))	1,26	1,72	2,97
	TTG(C(D))	1,67	1,35	3,02
	CAA(R(D))	1,72	1,26	2,97
AAG	AAG(D)	2,15	1,71	3,86
	CTT(RC(D))	1,77	2,23	4,00
	TTC(C(D))	1,71	2,15	3,86
	GAA(R(D))	2,23	1,77	4,00
ACA	ACA(D)	0,87	0,90	1,76
	TGT(RC(D))	0,90	0,87	1,76
	TGT(C(D))	0,90	0,87	1,76
	ACA(R(D))	0,87	0,90	1,76
ATA	ATA(D)	0,62	0,67	1,29
	TAT(RC(D))	0,67	0,62	1,29
	TAT(C(D))	0,67	0,62	1,29
	ATA(R(D))	0,62	0,62	1,24
AGA	AGA(D)	1,16	0,88	2,04
	TCT(RC(D))	0,88	1,16	2,04
	TCT(C(D))	0,88	1,16	2,04
	AGA(R(D))	1,16	0,88	2,04
AAA	AAA(D)	1,39	1,30	2,69
	TTT(RC(D))	1,30	1,39	2,69
	TTT(C(D))	1,30	1,39	2,69
	AAA(R(D))	1,39	1,30	2,69

Composition of DNA sequence in coding chromosome 8

Base	No.of bases	%
A	1682135	26,65%
C	1491327	23,63%
G	1550467	24,56%
T	1588021	25,16%
N	0	0,00%
Sum	6311950	100,00%

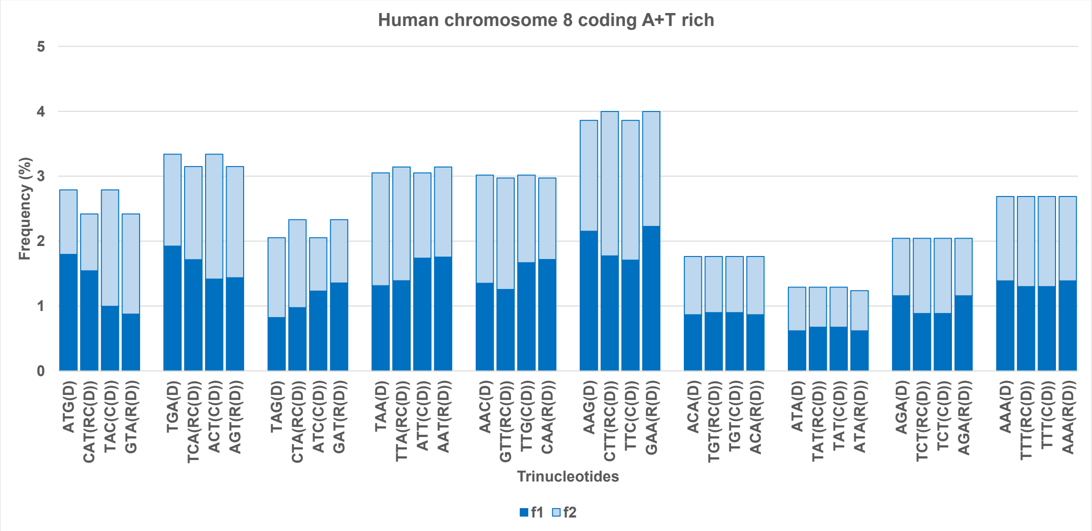

						C+G rich						
**D**	**f**	**%**	**RC**	**f**	**%**	**C**	**f**	**%**	**R**	**f**	**%**	
**CGT**	363522,00	0,25	ACG	361801,00	0,25	GCA	2079540,00	1,44	TGC	2057043,00	1,42	IIa
**GTC**	1345165,00	0,93	GAC	1354020,00	0,94	CAG	2871252,00	1,98	CTG	2839668,00	1,96	
**GCT**	1967618,00	1,36	AGC	1989968,00	1,37	CGA	301328,00	0,21	TCG	308696,00	0,21	
**GCC**	1592891,00	1,10	GGC	1595228,00	1,10	CGG	363475,00	0,25	CCG	355638,00	0,25	IIb
**CCA**	2567585,00	1,77	TGG	2597187,00	1,79	GGT	1626683,00	1,12	ACC	1630708,00	1,13	
**CCT**	2475658,00	1,71	AGG	2481083,00	1,71	GGA	2219834,00	1,53	TCC	2175282,00	1,50	
**CAC**	2137649,00	1,48	GTG	2143275,00	1,48	GTG*			CAC*			IIc
**CGC**	309875,00	0,21	GCG	312065,00	0,22	GCG*			CGC*			
**CTC**	2359835,00	1,63	GAG	2363251,00	1,63	GAG*			CTC*			
**CCC**	1770941,00	1,22	GGG	1774269,00	1,23	GGG*			CCC*			

Quadruplet		f1	f2	fu
CGT	CGT(D)	0,25	1,44	1,69
	ACG(RC(D))	0,25	1,42	1,67
	GCA(C(D))	1,44	0,25	1,69
	TGC(R(D))	1,42	0,25	1,67
GTC	GTC(D)	0,93	1,98	2,91
	GAC(RC(D))	0,94	1,96	2,90
	CAG(C(D))	1,98	0,93	2,91
	CTG(R(D))	1,96	0,94	2,90
GCT	GCT(D)	1,36	0,21	1,57
	AGC(RC(D))	1,37	0,21	1,59
	CGA(C(D))	0,21	1,36	1,57
	TCG(R(D))	0,21	1,37	1,59
GCC	GCC(D)	1,10	0,25	1,35
	GGC(RC(D))	1,10	0,25	1,35
	CGG(C(D))	0,25	1,10	1,35
	CCG(R(D))	0,25	1,10	1,35
CCA	CCA(D)	1,77	1,12	2,90
	TGG(RC(D))	1,79	1,13	2,92
	GGT(C(D))	1,12	1,77	2,90
	ACC(R(D))	1,13	1,79	2,92
CCT	CCT(D)	1,71	1,53	3,24
	AGG(RC(D))	1,71	1,50	3,22
	GGA(C(D))	1,53	1,71	3,24
	TCC(R(D))	1,50	1,71	3,22
CAC	CAC(D)	0,74	0,74	1,48
	GTG(RC(D))	0,74	0,74	1,48
	GTG(C(D))	0,74	0,74	1,48
	CAC(R(D))	0,74	0,74	1,48
CGC	CGC(D)	0,11	0,11	0,21
	GCG(RC(D))	0,11	0,11	0,21
	GCG(C(D))	0,11	0,11	0,21
	CGC(R(D))	0,11	0,11	0,21
CTC	CTC(D)	0,82	0,82	1,63
	GAG(RC(D))	0,82	0,82	1,63
	GAG(C(D))	0,82	0,82	1,63
	CTC(R(D))	0,82	0,82	1,63
CCC	CCC(D)	0,61	0,61	1,22
	GGG(RC(D))	0,61	0,61	1,22
	GGG(C(D))	0,61	0,61	1,22
	CCC(R(D))	0,61	0,61	1,22

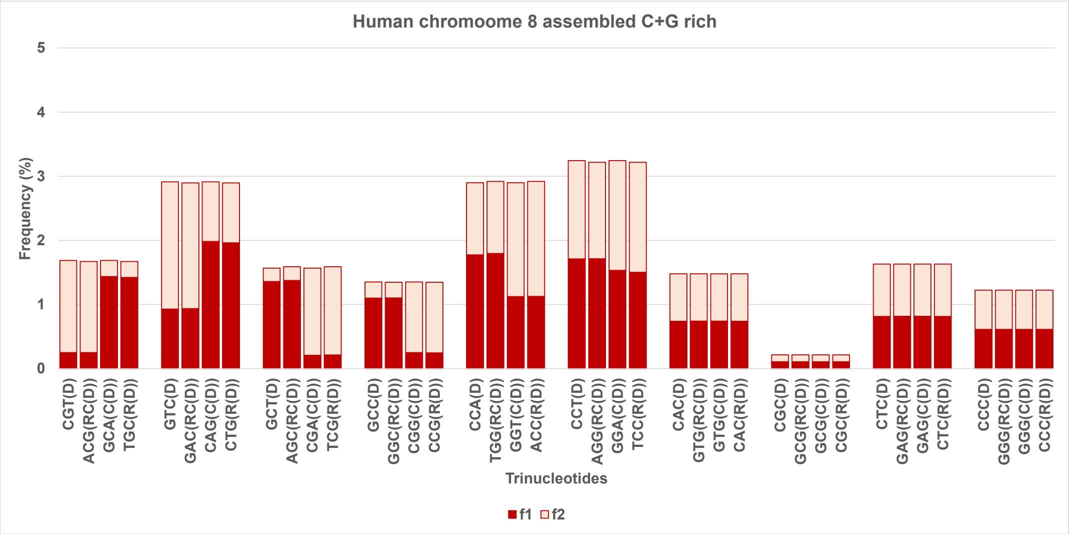

Human random chromomome 8

C+G rich												
**D**	**f**	**%**	**RC**	**f**	**%**	**C**	**f**	**%**	**R**	**f**	**%**	
**CGT**	593,00	0,30	ACG	545,00	0,27	GCA	3037,00	1,52	TGC	2856,00	1,43	IIa
**GTC**	1927,00	0,97	GAC	1833,00	0,92	CAG	4134,00	2,07	CTG	3977,00	1,99	
**GCT**	2755,00	1,38	AGC	2885,00	1,45	CGA	496,00	0,25	TCG	512,00	0,26	
**GCC**	2231,00	1,12	GGC	2277,00	1,14	CGG	565,00	0,28	CCG	602,00	0,30	IIb
**CCA**	3469,00	1,74	TGG	3645,00	1,83	GGT	2258,00	1,13	ACC	2173,00	1,09	
**CCT**	3491,00	1,75	AGG	3472,00	1,74	GGA	3147,00	1,58	TCC	3158,00	1,58	
**CAC**	2851,00	1,43	GTG	3070,00	1,54	GTG*			CAC*			IIc
**CGC**	539,00	0,27	GCG	534,00	0,27	GCG*			CGC*			
**CTC**	3268,00	1,64	GAG	3334,00	1,67	GAG*			CTC*			
**CCC**	2530,00	1,27	GGG	2462,00	1,23	GGG*			CCC*			

Quadruplet		f1	f2	fu
CGT	CGT(D)	0,30	1,52	1,82
	ACG(RC(D))	0,27	1,43	1,71
	GCA(C(D))	1,52	0,30	1,82
	TGC(R(D))	1,43	0,27	1,71
GTC	GTC(D)	0,97	2,07	3,04
	GAC(RC(D))	0,92	1,99	2,91
	CAG(C(D))	2,07	0,97	3,04
	CTG(R(D))	1,99	0,92	2,91
GCT	GCT(D)	1,38	0,25	1,63
	AGC(RC(D))	1,45	0,26	1,70
	CGA(C(D))	0,25	1,38	1,63
	TCG(R(D))	0,26	1,45	1,70
GCC	GCC(D)	1,12	0,28	1,40
	GGC(RC(D))	1,14	0,30	1,44
	CGG(C(D))	0,28	1,12	1,40
	CCG(R(D))	0,30	1,14	1,44
CCA	CCA(D)	1,74	1,13	2,87
	TGG(RC(D))	1,83	1,09	2,92
	GGT(C(D))	1,13	1,74	2,87
	ACC(R(D))	1,09	1,83	2,92
CCT	CCT(D)	1,75	1,58	3,33
	AGG(RC(D))	1,74	1,58	3,33
	GGA(C(D))	1,58	1,75	3,33
	TCC(R(D))	1,58	1,74	3,33
CAC	CAC(D)	0,71	0,77	1,48
	GTG(RC(D))	0,77	0,71	1,48
	GTG(C(D))	0,77	0,71	1,48
	CAC(R(D))	0,71	0,77	1,48
CGC	CGC(D)	0,14	0,13	0,27
	GCG(RC(D))	0,13	0,14	0,27
	GCG(C(D))	0,13	0,14	0,27
	CGC(R(D))	0,14	0,13	0,27
CTC	CTC(D)	0,82	0,84	1,66
	GAG(RC(D))	0,84	0,82	1,66
	GAG(C(D))	0,84	0,82	1,66
	CTC(R(D))	0,82	0,84	1,66
CCC	CCC(D)	0,63	0,62	1,25
	GGG(RC(D))	0,62	0,63	1,25
	GGG(C(D))	0,62	0,63	1,25
	CCC(R(D))	0,63	0,62	1,25

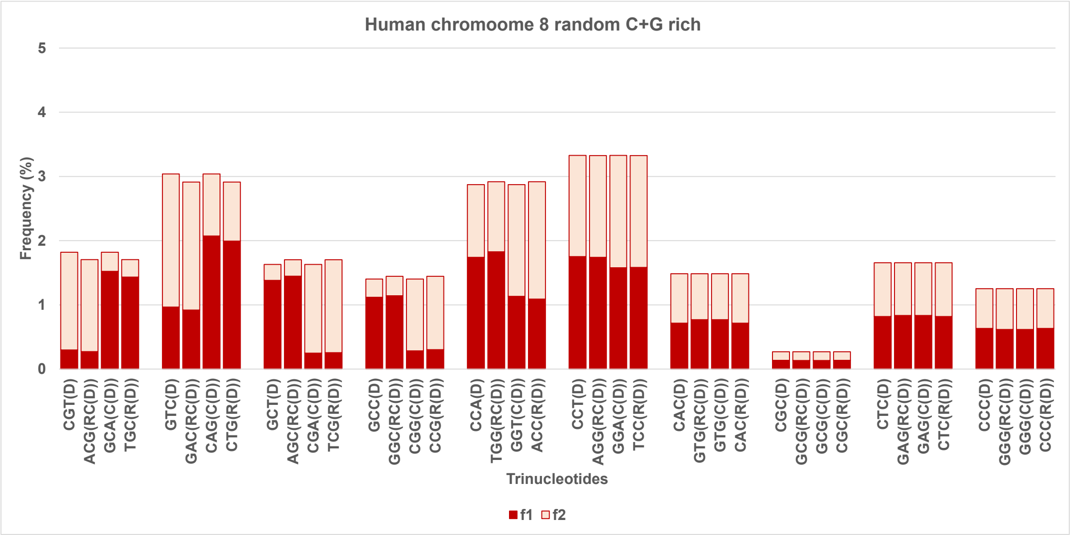

Human coding chromomome 8

C+G rich

D	f	%	RC	f	%	C	f	%	R	f	%	
**CGT**	31951,00	0,51	ACG	35006,00	0,55	GCA	111253,00	1,76	TGC	112788,00	1,79	IIa
**GTC**	65909,00	1,04	GAC	80683,00	1,28	CAG	158024,00	2,50	CTG	158489,00	2,51	
**GCT**	111514,00	1,77	AGC	117987,00	1,87	CGA	35988,00	0,57	TCG	31377,00	0,50	
**GCC**	115999,00	1,84	GGC	110767,00	1,75	CGG	58020,00	0,92	CCG	56800,00	0,90	IIb
**CCA**	127962,00	2,03	TGG	136826,00	2,17	GGT	71832,00	1,14	ACC	87825,00	1,39	
**CCT**	124346,00	1,97	AGG	120455,00	1,91	GGA	132703,00	2,10	TCC	105284,00	1,67	
**CAC**	92852,00	1,47	GTG	107321,00	1,70	GTG*			CAC*			IIc
**CGC**	49197,00	0,78	GCG	51973,00	0,82	GCG*			CGC*			
**CTC**	105076,00	1,66	GAG	129470,00	2,05	GAG*			CTC*			
**CCC**	113462,00	1,80	GGG	100509,00	1,59	GGG*			CCC*			

Quadruplet		f1	f2	fu
CGT	CGT(D)	0,51	1,76	2,27
	ACG(RC(D))	0,55	1,79	2,34
	GCA(C(D))	1,76	0,51	2,27
	TGC(R(D))	1,79	0,55	2,34
GTC	GTC(D)	1,04	2,50	3,55
	GAC(RC(D))	1,28	2,51	3,79
	CAG(C(D))	2,50	1,04	3,55
	CTG(R(D))	2,51	1,28	3,79
GCT	GCT(D)	1,77	0,57	2,34
	AGC(RC(D))	1,87	0,50	2,37
	CGA(C(D))	0,57	1,77	2,34
	TCG(R(D))	0,50	1,87	2,37
GCC	GCC(D)	1,84	0,92	2,76
	GGC(RC(D))	1,75	0,90	2,65
	CGG(C(D))	0,92	1,84	2,76
	CCG(R(D))	0,90	1,75	2,65
CCA	CCA(D)	2,03	1,14	3,17
	TGG(RC(D))	2,17	1,39	3,56
	GGT(C(D))	1,14	2,03	3,17
	ACC(R(D))	1,39	2,17	3,56
CCT	CCT(D)	1,97	2,10	4,07
	AGG(RC(D))	1,91	1,67	3,58
	GGA(C(D))	2,10	1,97	4,07
	TCC(R(D))	1,67	1,91	3,58
CAC	CAC(D)	0,74	0,85	1,59
	GTG(RC(D))	0,85	0,74	1,59
	GTG(C(D))	0,85	0,74	1,59
	CAC(R(D))	0,74	0,85	1,59
CGC	CGC(D)	0,39	0,41	0,80
	GCG(RC(D))	0,41	0,39	0,80
	GCG(C(D))	0,41	0,39	0,80
	CGC(R(D))	0,39	0,41	0,80
CTC	CTC(D)	0,83	1,03	1,86
	GAG(RC(D))	1,03	0,83	1,86
	GAG(C(D))	1,03	0,83	1,86
	CTC(R(D))	0,83	1,03	1,86
CCC	CCC(D)	0,90	0,80	1,69
	GGG(RC(D))	0,80	0,90	1,69
	GGG(C(D))	0,80	0,90	1,69
	CCC(R(D))	0,90	0,80	1,69

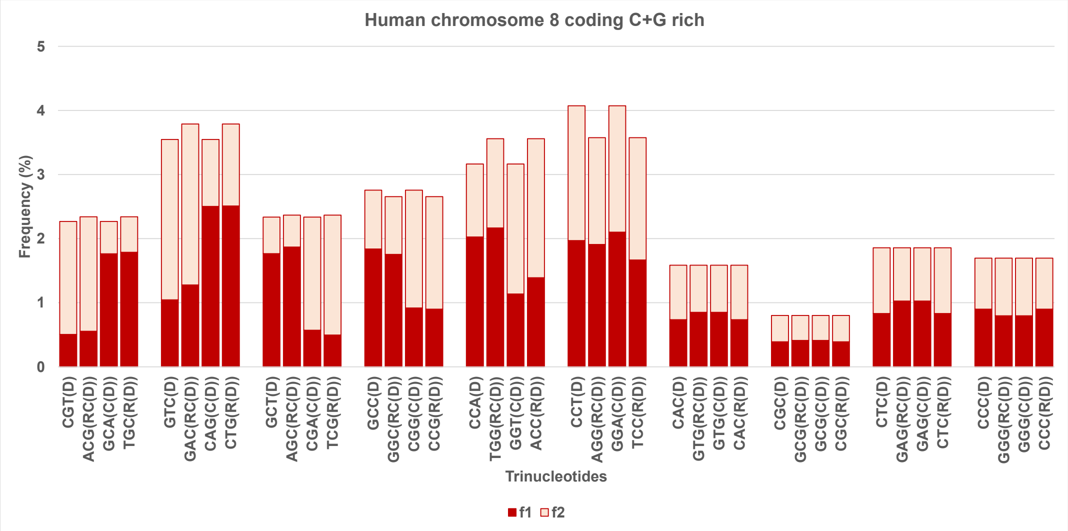

Human assembled chromomome 9

					A-T rich							
**D**	**f**	**%**	**RC**	**f**	**%**	**C**	**f**	**%**	**R**	**f**	**%**	
**ATG**	2210216,00	1,81	CAT	2229372,00	1,83	TAC	1355921,00	1,11	GTA	1360476,00	1,12	Ia
**TGA**	2393176,00	1,96	TCA	2385680,00	1,96	ACT	1960840,00	1,61	AGT	1945370,00	1,60	
**TAG**	1554667,00	1,28	CTA	1552658,00	1,27	ATC	1613301,00	1,32	GAT	1626204,00	1,34	
**TAA**	2449416,00	2,01	TTA	2441040,00	2,00	ATT	2967860,00	2,44	AAT	2941630,00	2,42	Ib
**AAC**	1800528,00	1,48	GTT	1773059,00	1,46	TTG	2326298,00	1,91	CAA	2276311,00	1,87	
**AAG**	2421335,00	1,99	CTT	2448643,00	2,01	TTC	2422227,00	1,99	GAA	2437767,00	2,00	
**ACA**	2460103,00	2,02	TGT	2463842,00	2,02	TGT*			ACA*			Ic
**ATA**	2446249,00	2,01	TAT	2440420,00	2,00	TAT*			ATA*			
**AGA**	2705781,00	2,22	TCT	2706023,00	2,22	TCT*			AGA*			
**AAA**	4616525,00	3,79	TTT	4649550,00	3,82	TTT*			AAA*			

Quadruplet		f1	f2	fu
ATG	ATG(D)	1,81	1,11	2,93
	CAT(RC(D))	1,83	1,12	2,95
	TAC(C(D))	1,11	1,81	2,93
	GTA(R(D))	1,12	1,83	2,95
TGA	TGA(D)	1,96	1,61	3,58
	TCA(RC(D))	1,96	1,60	3,56
	ACT(C(D))	1,61	1,96	3,58
	AGT(R(D))	1,60	1,96	3,56
TAG	TAG(D)	1,28	1,32	2,60
	CTA(RC(D))	1,27	1,34	2,61
	ATC(C(D))	1,32	1,28	2,60
	GAT(R(D))	1,34	1,27	2,61
TAA	TAA(D)	2,01	2,44	4,45
	TTA(RC(D))	2,00	2,42	4,42
	ATT(C(D))	2,44	2,01	4,45
	AAT(R(D))	2,42	2,00	4,42
AAC	AAC(D)	1,48	1,91	3,39
	GTT(RC(D))	1,46	1,87	3,32
	TTG(C(D))	1,91	1,48	3,39
	CAA(R(D))	1,87	1,46	3,32
AAG	AAG(D)	1,99	1,99	3,98
	CTT(RC(D))	2,01	2,00	4,01
	TTC(C(D))	1,99	1,99	3,98
	GAA(R(D))	2,00	2,01	4,01
ACA	ACA(D)	1,01	1,01	2,02
	TGT(RC(D))	1,01	1,01	2,02
	TGT(C(D))	1,01	1,01	2,02
	ACA(R(D))	1,01	1,01	2,02
ATA	ATA(D)	1,00	1,00	2,01
	TAT(RC(D))	1,00	1,00	2,01
	TAT(C(D))	1,00	1,00	2,01
	ATA(R(D))	1,00	1,00	2,01
AGA	AGA(D)	1,11	1,11	2,22
	TCT(RC(D))	1,11	1,11	2,22
	TCT(C(D))	1,11	1,11	2,22
	AGA(R(D))	1,11	1,11	2,22
AAA	AAA(D)	1,90	1,91	3,80
	TTT(RC(D))	1,91	1,90	3,80
	TTT(C(D))	1,91	1,90	3,80
	AAA(R(D))	1,90	1,91	3,80

Composition of DNA sequence in assembled chromosome 9

Base	No.of bases	%
A	35736329	25,82%
C	25099811	18,14%
G	25170662	18,19%
T	35783748	25,86%
N	16604167	12,00%
Sum	138394717	100,00%

% of coding bases from complete assembled chromosome 1 (including N bases)

**4,84%**

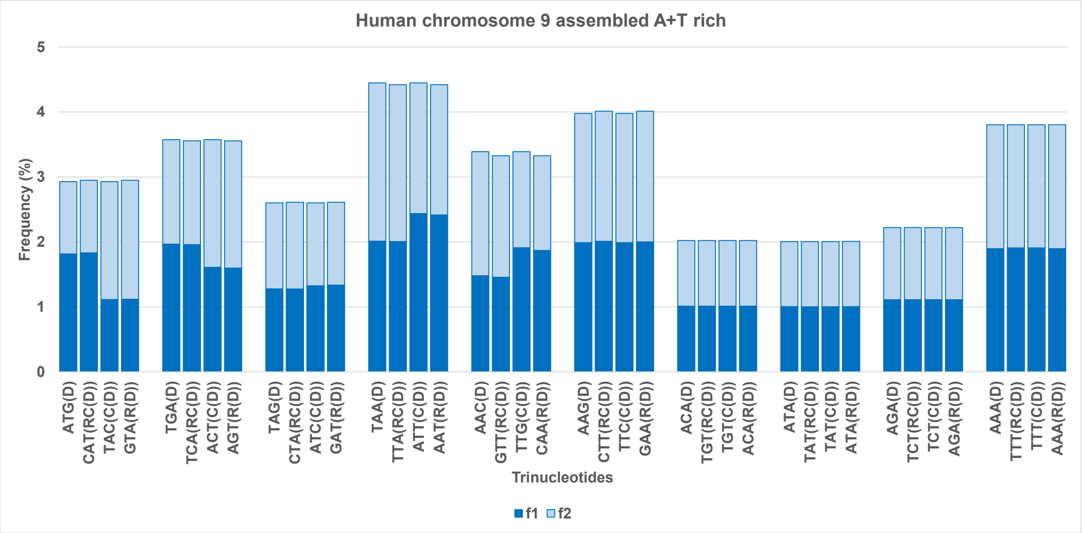

Human random chromomome 9

A-T rich

D	f	%	RC	f	%	C	f	%	R	f	%	
**ATG**	2870,00	1,83	CAT	2962,00	1,89	TAC	1856,00	1,19	GTA	1800,00	1,15	Ia
**TGA**	3090,00	1,97	TCA	3110,00	1,99	ACT	2577,00	1,65	AGT	2603,00	1,66	
**TAG**	2073,00	1,32	CTA	2144,00	1,37	ATC	2158,00	1,38	GAT	2173,00	1,39	
**TAA**	3362,00	2,15	TTA	3313,00	2,12	ATT	4110,00	2,62	AAT	4034,00	2,58	Ib
**AAC**	2390,00	1,53	GTT	2400,00	1,53	TTG	3179,00	2,03	CAA	2957,00	1,89	
**AAG**	3061,00	1,95	CTT	3311,00	2,11	TTC	3329,00	2,13	GAA	3166,00	2,02	
**ACA**	3143,00	2,01	TGT	3214,00	2,05	TGT*			ACA*			Ic
**ATA**	3496,00	2,23	TAT	3463,00	2,21	TAT*			ATA*			
**AGA**	3374,00	2,15	TCT	3674,00	2,35	TCT*			AGA*			
**AAA**	6317,00	4,03	TTT	6600,00	4,21	TTT*			AAA*			

Quadruplet		f1	f2	fu
ATG	ATG(D)	1,83	1,19	3,02
	CAT(RC(D))	1,89	1,15	3,04
	TAC(C(D))	1,19	1,83	3,02
	GTA(R(D))	1,15	1,89	3,04
TGA	TGA(D)	1,97	1,65	3,62
	TCA(RC(D))	1,99	1,66	3,65
	ACT(C(D))	1,65	1,97	3,62
	AGT(R(D))	1,66	1,99	3,65
TAG	TAG(D)	1,32	1,38	2,70
	CTA(RC(D))	1,37	1,39	2,76
	ATC(C(D))	1,38	1,32	2,70
	GAT(R(D))	1,39	1,37	2,76
TAA	TAA(D)	2,15	2,62	4,77
	TTA(RC(D))	2,12	2,58	4,69
	ATT(C(D))	2,62	2,15	4,77
	AAT(R(D))	2,58	2,12	4,69
AAC	AAC(D)	1,53	2,03	3,56
	GTT(RC(D))	1,53	1,89	3,42
	TTG(C(D))	2,03	1,53	3,56
	CAA(R(D))	1,89	1,53	3,42
AAG	AAG(D)	1,95	2,13	4,08
	CTT(RC(D))	2,11	2,02	4,14
	TTC(C(D))	2,13	1,95	4,08
	GAA(R(D))	2,02	2,11	4,14
ACA	ACA(D)	1,00	1,03	2,03
	TGT(RC(D))	1,03	1,00	2,03
	TGT(C(D))	1,03	1,00	2,03
	ACA(R(D))	1,00	1,03	2,03
ATA	ATA(D)	1,12	1,11	2,22
	TAT(RC(D))	1,11	1,12	2,22
	TAT(C(D))	1,11	1,12	2,22
	ATA(R(D))	1,12	1,12	2,23
AGA	AGA(D)	1,08	1,17	2,25
	TCT(RC(D))	1,17	1,08	2,25
	TCT(C(D))	1,17	1,08	2,25
	AGA(R(D))	1,08	1,17	2,25
AAA	AAA(D)	2,02	2,11	4,12
	TTT(RC(D))	2,11	2,02	4,12
	TTT(C(D))	2,11	2,02	4,12
	AAA(R(D))	2,02	2,11	4,12

Composition of DNA sequence in random chromosome 9

Base	No.of bases	%
A	46861	23,43%
C	31230	15,62%
G	30670	15,34%
T	47880	23,94%
N	43359	21,68%
Sum	200000	100,00%

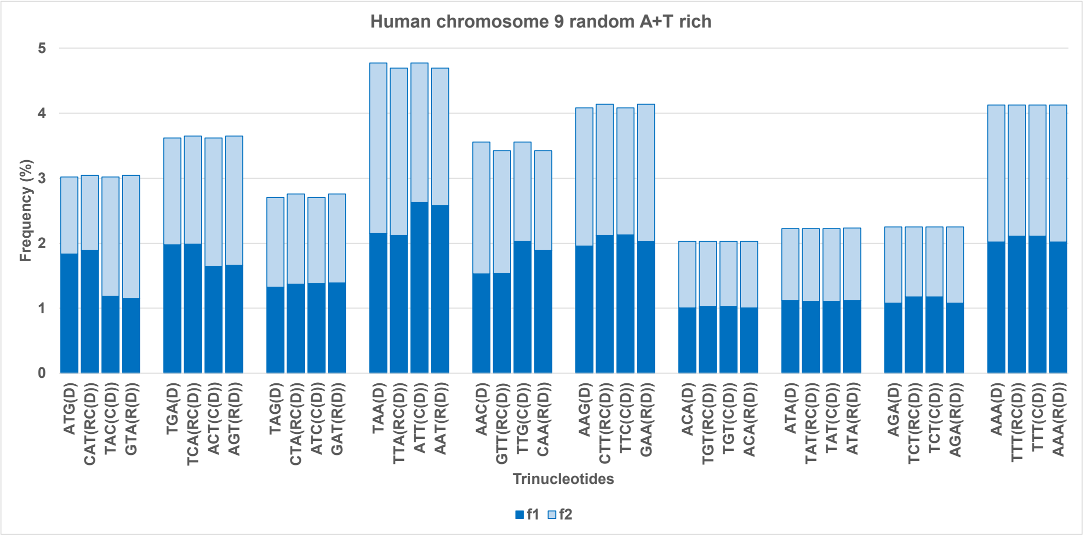

Human coding chromomome 9

A-T rich

D	f	%	RC	f	%	C	f	%	R	f	%	
**ATG**	114391,00	1,71	CAT	102460,00	1,53	TAC	62607,00	0,93	GTA	52663,00	0,79	Ia
**TGA**	126858,00	1,89	TCA	116438,00	1,74	ACT	92068,00	1,37	AGT	93011,00	1,39	
**TAG**	48996,00	0,73	CTA	61954,00	0,93	ATC	81914,00	1,22	GAT	86451,00	1,29	
**TAA**	72965,00	1,09	TTA	76112,00	1,14	ATT	98261,00	1,47	AAT	99494,00	1,49	Ib
**AAC**	87291,00	1,30	GTT	77161,00	1,15	TTG	102317,00	1,53	CAA	113975,00	1,70	
**AAG**	141236,00	2,11	CTT	112737,00	1,68	TTC	109730,00	1,64	GAA	141082,00	2,11	
**ACA**	116605,00	1,74	TGT	116649,00	1,74	TGT*			ACA*			Ic
**ATA**	68366,00	1,02	TAT	74527,00	1,11	TAT*			ATA*			
**AGA**	152563,00	2,28	TCT	116304,00	1,74	TCT*			AGA*			
**AAA**	162718,00	2,43	TTT	146357,00	2,19	TTT*			AAA*			

Quadruplet		f1	f2	fu
ATG	ATG(D)	1,71	0,93	2,64
	CAT(RC(D))	1,53	0,79	2,32
	TAC(C(D))	0,93	1,71	2,64
	GTA(R(D))	0,79	1,53	2,32
TGA	TGA(D)	1,89	1,37	3,27
	TCA(RC(D))	1,74	1,39	3,13
	ACT(C(D))	1,37	1,89	3,27
	AGT(R(D))	1,39	1,74	3,13
TAG	TAG(D)	0,73	1,22	1,95
	CTA(RC(D))	0,93	1,29	2,22
	ATC(C(D))	1,22	0,73	1,95
	GAT(R(D))	1,29	0,93	2,22
TAA	TAA(D)	1,09	1,47	2,56
	TTA(RC(D))	1,14	1,49	2,62
	ATT(C(D))	1,47	1,09	2,56
	AAT(R(D))	1,49	1,14	2,62
AAC	AAC(D)	1,30	1,53	2,83
	GTT(RC(D))	1,15	1,70	2,85
	TTG(C(D))	1,53	1,30	2,83
	CAA(R(D))	1,70	1,15	2,85
AAG	AAG(D)	2,11	1,64	3,75
	CTT(RC(D))	1,68	2,11	3,79
	TTC(C(D))	1,64	2,11	3,75
	GAA(R(D))	2,11	1,68	3,79
ACA	ACA(D)	0,87	0,87	1,74
	TGT(RC(D))	0,87	0,87	1,74
	TGT(C(D))	0,87	0,87	1,74
	ACA(R(D))	0,87	0,87	1,74
ATA	ATA(D)	0,51	0,56	1,07
	TAT(RC(D))	0,56	0,51	1,07
	TAT(C(D))	0,56	0,51	1,07
	ATA(R(D))	0,51	0,51	1,02
AGA	AGA(D)	1,14	0,87	2,01
	TCT(RC(D))	0,87	1,14	2,01
	TCT(C(D))	0,87	1,14	2,01
	AGA(R(D))	1,14	0,87	2,01
AAA	AAA(D)	1,21	1,09	2,31
	TTT(RC(D))	1,09	1,21	2,31
	TTT(C(D))	1,09	1,21	2,31
	AAA(R(D))	1,21	1,09	2,31

Composition of DNA sequence in coding chromosome 9

Base	No.of bases	%
A	1718948	25,67%
C	1678535	25,07%
G	1702340	25,42%
T	1596763	23,84%
N	0	0,00%
Sum	6696586	100,00%

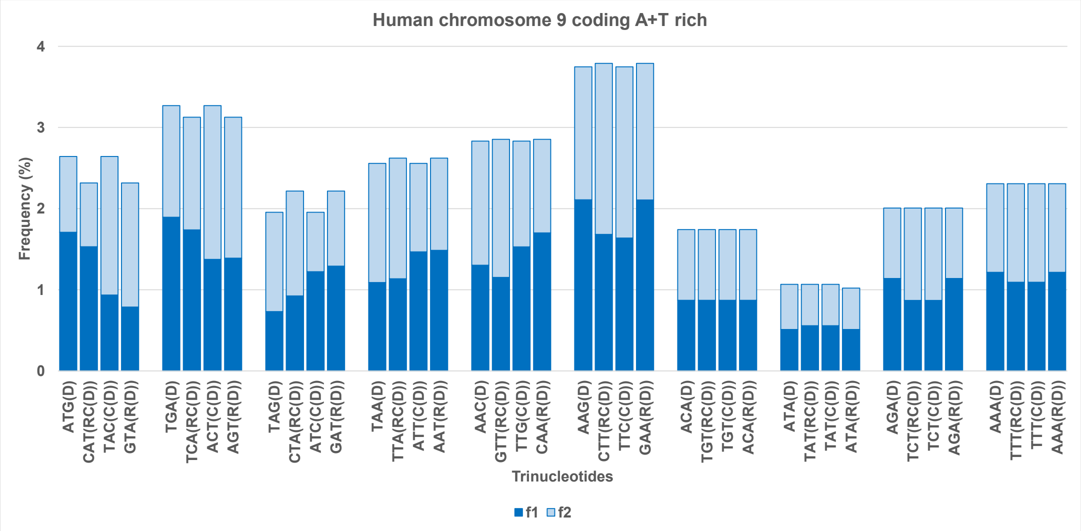

C+G rich												
**D**	**f**	**%**	**RC**	**f**	**%**	**C**	**f**	**%**	**R**	**f**	**%**	
**CGT**	316016,00	0,26	ACG	320456,00	0,26	GCA	1774231,00	1,46	TGC	1762138,00	1,45	IIa
**GTC**	1146453,00	0,94	GAC	1158353,00	0,95	CAG	2514211,00	2,06	CTG	2494727,00	2,05	
**GCT**	1711447,00	1,41	AGC	1726921,00	1,42	CGA	271168,00	0,22	TCG	276410,00	0,23	
**GCC**	1480928,00	1,22	GGC	1479344,00	1,21	CGG	362063,00	0,30	CCG	350587,00	0,29	IIb
**CCA**	2241717,00	1,84	TGG	2274367,00	1,87	GGT	1417043,00	1,16	ACC	1415239,00	1,16	
**CCT**	2186408,00	1,80	AGG	2183955,00	1,79	GGA	1924000,00	1,58	TCC	1882544,00	1,55	
**CAC**	1841838,00	1,51	GTG	1862280,00	1,53	GTG*			CAC*			IIc
**CGC**	306481,00	0,25	GCG	308275,00	0,25	GCG*			CGC*			
**CTC**	2068690,00	1,70	GAG	2071814,00	1,70	GAG*			CTC*			
**CCC**	1638893,00	1,35	GGG	1638975,00	1,35	GGG*			CCC*			

Quadruplet		f1	f2	fu
CGT	CGT(D)	0,26	1,46	1,72
	ACG(RC(D))	0,26	1,45	1,71
	GCA(C(D))	1,46	0,26	1,72
	TGC(R(D))	1,45	0,26	1,71
GTC	GTC(D)	0,94	2,06	3,01
	GAC(RC(D))	0,95	2,05	3,00
	CAG(C(D))	2,06	0,94	3,01
	CTG(R(D))	2,05	0,95	3,00
GCT	GCT(D)	1,41	0,22	1,63
	AGC(RC(D))	1,42	0,23	1,64
	CGA(C(D))	0,22	1,41	1,63
	TCG(R(D))	0,23	1,42	1,64
GCC	GCC(D)	1,22	0,30	1,51
	GGC(RC(D))	1,21	0,29	1,50
	CGG(C(D))	0,30	1,22	1,51
	CCG(R(D))	0,29	1,21	1,50
CCA	CCA(D)	1,84	1,16	3,00
	TGG(RC(D))	1,87	1,16	3,03
	GGT(C(D))	1,16	1,84	3,00
	ACC(R(D))	1,16	1,87	3,03
CCT	CCT(D)	1,80	1,58	3,37
	AGG(RC(D))	1,79	1,55	3,34
	GGA(C(D))	1,58	1,80	3,37
	TCC(R(D))	1,55	1,79	3,34
CAC	CAC(D)	0,76	0,76	1,52
	GTG(RC(D))	0,76	0,76	1,52
	GTG(C(D))	0,76	0,76	1,52
	CAC(R(D))	0,76	0,76	1,52
CGC	CGC(D)	0,13	0,13	0,25
	GCG(RC(D))	0,13	0,13	0,25
	GCG(C(D))	0,13	0,13	0,25
	CGC(R(D))	0,13	0,13	0,25
CTC	CTC(D)	0,85	0,85	1,70
	GAG(RC(D))	0,85	0,85	1,70
	GAG(C(D))	0,85	0,85	1,70
	CTC(R(D))	0,85	0,85	1,70
CCC	CCC(D)	0,67	0,67	1,35
	GGG(RC(D))	0,67	0,67	1,35
	GGG(C(D))	0,67	0,67	1,35
	CCC(R(D))	0,67	0,67	1,35

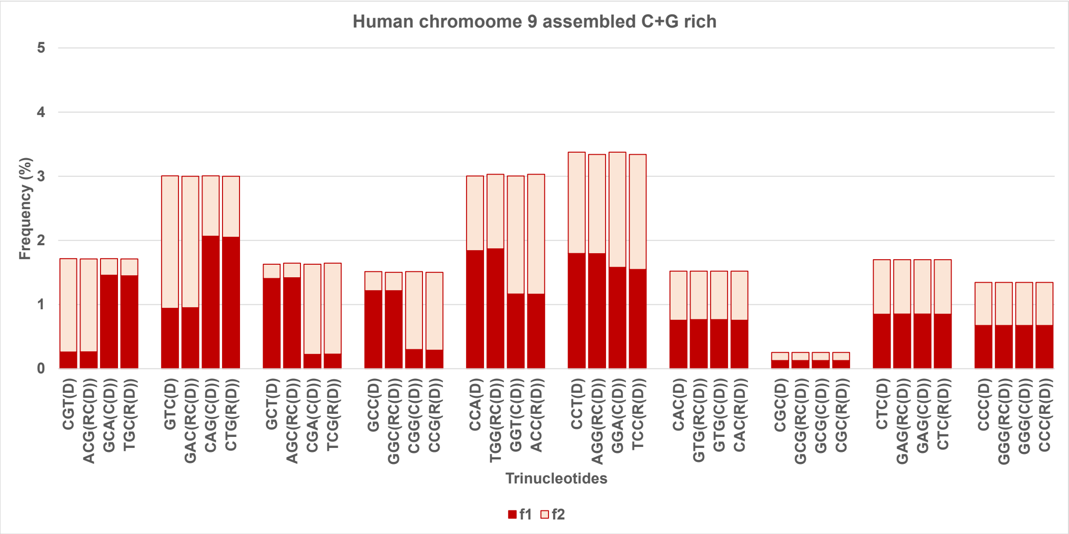

Human random chromomome 9

C+G rich

D	f	%	RC	f	%	C	f	%	R	f	%	
**CGT**	382,00	0,24	ACG	396,00	0,25	GCA	2169,00	1,38	TGC	2205,00	1,41	IIa
**GTC**	1401,00	0,89	GAC	1374,00	0,88	CAG	3017,00	1,93	CTG	2932,00	1,87	
**GCT**	2050,00	1,31	AGC	2087,00	1,33	CGA	325,00	0,21	TCG	316,00	0,20	
**GCC**	1711,00	1,09	GGC	1626,00	1,04	CGG	397,00	0,25	CCG	404,00	0,26	IIb
**CCA**	2729,00	1,74	TGG	2724,00	1,74	GGT	1655,00	1,06	ACC	1721,00	1,10	
**CCT**	2665,00	1,70	AGG	2518,00	1,61	GGA	2359,00	1,51	TCC	2365,00	1,51	
**CAC**	2217,00	1,42	GTG	2252,00	1,44	GTG*			CAC*			IIc
**CGC**	348,00	0,22	GCG	336,00	0,21	GCG*			CGC*			
**CTC**	2578,00	1,65	GAG	2435,00	1,55	GAG*			CTC*			
**CCC**	1857,00	1,19	GGG	1757,00	1,12	GGG*			CCC*			

Quadruplet		f1	f2	fu
	CGT(D)	0,24	1,38	1,63
CGT	ACG(RC(D))	0,25	1,41	1,66
	GCA(C(D))	1,38	0,24	1,63
	TGC(R(D))	1,41	0,25	1,66
	GTC(D)	0,89	1,93	2,82
GTC	GAC(RC(D))	0,88	1,87	2,75
	CAG(C(D))	1,93	0,89	2,82
	CTG(R(D))	1,87	0,88	2,75
	GCT(D)	1,31	0,21	1,52
GCT	AGC(RC(D))	1,33	0,20	1,53
	CGA(C(D))	0,21	1,31	1,52
	TCG(R(D))	0,20	1,33	1,53
	GCC(D)	1,09	0,25	1,35
GCC	GGC(RC(D))	1,04	0,26	1,30
	CGG(C(D))	0,25	1,09	1,35
	CCG(R(D))	0,26	1,04	1,30
	CCA(D)	1,74	1,06	2,80
CCA	TGG(RC(D))	1,74	1,10	2,84
	GGT(C(D))	1,06	1,74	2,80
	ACC(R(D))	1,10	1,74	2,84
	CCT(D)	1,70	1,51	3,21
CCT	AGG(RC(D))	1,61	1,51	3,12
	GGA(C(D))	1,51	1,70	3,21
	TCC(R(D))	1,51	1,61	3,12
	CAC(D)	0,71	0,72	1,43
CAC	GTG(RC(D))	0,72	0,71	1,43
	GTG(C(D))	0,72	0,71	1,43
	CAC(R(D))	0,71	0,72	1,43
	CGC(D)	0,11	0,11	0,22
CGC	GCG(RC(D))	0,11	0,11	0,22
	GCG(C(D))	0,11	0,11	0,22
	CGC(R(D))	0,11	0,11	0,22
	CTC(D)	0,82	0,78	1,60
CTC	GAG(RC(D))	0,78	0,82	1,60
	GAG(C(D))	0,78	0,82	1,60
	CTC(R(D))	0,82	0,78	1,60
	CCC(D)	0,59	0,56	1,15
CCC	GGG(RC(D))	0,56	0,59	1,15
	GGG(C(D))	0,56	0,59	1,15
	CCC(R(D))	0,59	0,56	1,15

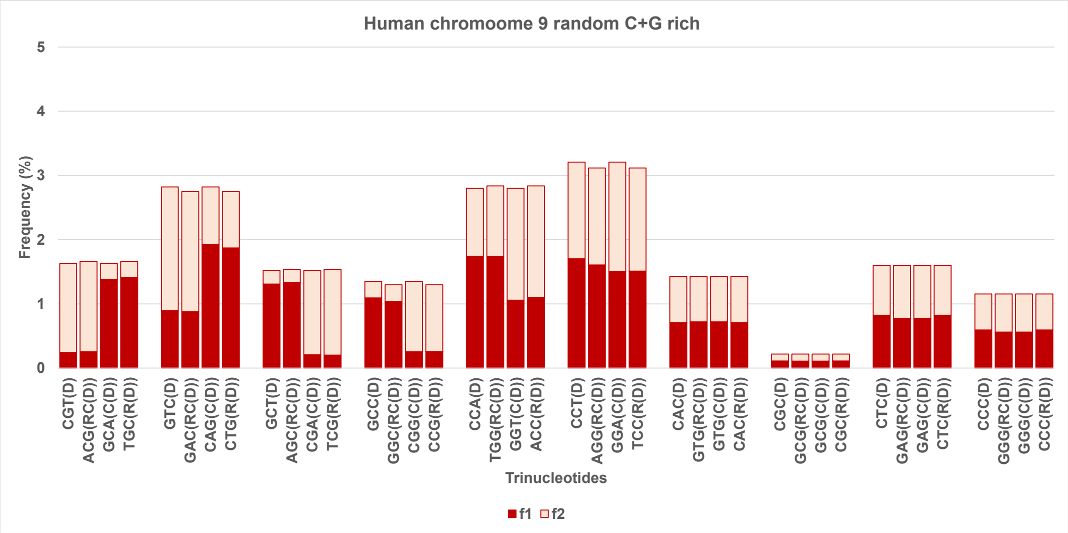

Human coding chromomome 9

C+G rich

D	f	%	RC	f	%	C	f	%	R	f	%	
**CGT**	34649,00	0,52	ACG	38152,00	0,57	GCA	120408,00	1,80	TGC	120848,00	1,80	IIa
**GTC**	74463,00	1,11	GAC	92041,00	1,37	CAG	181172,00	2,71	CTG	181407,00	2,71	
**GCT**	122236,00	1,83	AGC	134496,00	2,01	CGA	39542,00	0,59	TCG	34598,00	0,52	
**GCC**	141180,00	2,11	GGC	128716,00	1,92	CGG	64210,00	0,96	CCG	65415,00	0,98	IIb
**CCA**	150835,00	2,25	TGG	152579,00	2,28	GGT	78797,00	1,18	ACC	101793,00	1,52	
**CCT**	145601,00	2,17	AGG	136587,00	2,04	GGA	145864,00	2,18	TCC	118878,00	1,78	
**CAC**	106679,00	1,59	GTG	118819,00	1,77	GTG*			CAC*			IIc
**CGC**	54988,00	0,82	GCG	55224,00	0,82	GCG*			CGC*			
**CTC**	120111,00	1,79	GAG	145253,00	2,17	GAG*			CTC*			
**CCC**	142800,00	2,13	GGG	121982,00	1,82	GGG*			CCC*			

Quadruplet		f1	f2	fu
CGT	CGT(D)	0,52	1,80	2,32
	ACG(RC(D))	0,57	1,80	2,37
	GCA(C(D))	1,80	0,52	2,32
	TGC(R(D))	1,80	0,57	2,37
GTC	GTC(D)	1,11	2,71	3,82
	GAC(RC(D))	1,37	2,71	4,08
	CAG(C(D))	2,71	1,11	3,82
	CTG(R(D))	2,71	1,37	4,08
GCT	GCT(D)	1,83	0,59	2,42
	AGC(RC(D))	2,01	0,52	2,53
	CGA(C(D))	0,59	1,83	2,42
	TCG(R(D))	0,52	2,01	2,53
GCC	GCC(D)	2,11	0,96	3,07
	GGC(RC(D))	1,92	0,98	2,90
	CGG(C(D))	0,96	2,11	3,07
	CCG(R(D))	0,98	1,92	2,90
CCA	CCA(D)	2,25	1,18	3,43
	TGG(RC(D))	2,28	1,52	3,80
	GGT(C(D))	1,18	2,25	3,43
	ACC(R(D))	1,52	2,28	3,80
CCT	CCT(D)	2,17	2,18	4,35
	AGG(RC(D))	2,04	1,78	3,81
	GGA(C(D))	2,18	2,17	4,35
	TCC(R(D))	1,78	2,04	3,81
CAC	CAC(D)	0,80	0,89	1,68
	GTG(RC(D))	0,89	0,80	1,68
	GTG(C(D))	0,89	0,80	1,68
	CAC(R(D))	0,80	0,89	1,68
CGC	CGC(D)	0,41	0,41	0,82
	GCG(RC(D))	0,41	0,41	0,82
	GCG(C(D))	0,41	0,41	0,82
	CGC(R(D))	0,41	0,41	0,82
CTC	CTC(D)	0,90	1,08	1,98
	GAG(RC(D))	1,08	0,90	1,98
	GAG(C(D))	1,08	0,90	1,98
	CTC(R(D))	0,90	1,08	1,98
CCC	CCC(D)	1,07	0,91	1,98
	GGG(RC(D))	0,91	1,07	1,98
	GGG(C(D))	0,91	1,07	1,98
	CCC(R(D))	1,07	0,91	1,98

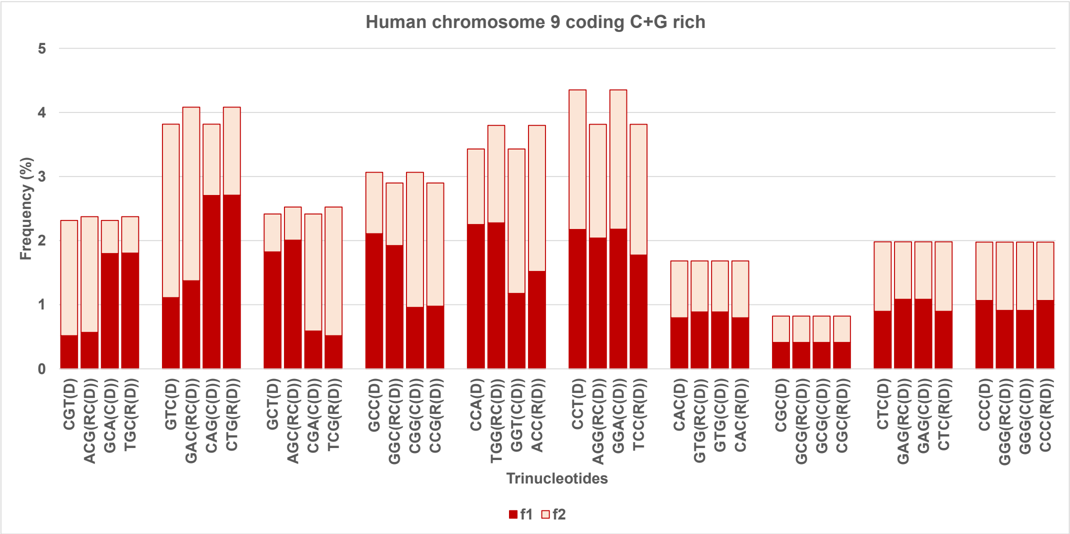

Human assembled chromomome 10

						A-T rich						
**D**	**f**	**%**	**RC**	**f**	**%**	**C**	**f**	**%**	**R**	**f**	**%**	
**ATG**	2433144,00	1,83	CAT	2423549,00	1,82	TAC	1455089,00	1,09	GTA	1469178,00	1,10	Ia
**TGA**	2592945,00	1,95	TCA	2627092,00	1,97	ACT	2129661,00	1,60	AGT	2138882,00	1,61	
**TAG**	1673486,00	1,26	CTA	1667004,00	1,25	ATC	1757192,00	1,32	GAT	1766417,00	1,33	
**TAA**	2644506,00	1,98	TTA	2642694,00	1,98	ATT	3218805,00	2,42	AAT	3213378,00	2,41	Ib
**AAC**	1938177,00	1,45	GTT	1939459,00	1,46	TTG	2535754,00	1,90	CAA	2490142,00	1,87	
**AAG**	2635729,00	1,98	CTT	2679487,00	2,01	TTC	2659306,00	2,00	GAA	2652633,00	1,99	
**ACA**	2678089,00	2,01	TGT	2711139,00	2,03	TGT*			ACA*			Ic
**ATA**	2577239,00	1,93	TAT	2583034,00	1,94	TAT*			ATA*			
**AGA**	2956834,00	2,22	TCT	2952781,00	2,22	TCT*			AGA*			
**AAA**	4971165,00	3,73	TTT	5038109,00	3,78	TTT*			AAA*			

Quadruplet		f1	f2	fu
ATG	ATG(D)	1,83	1,09	2,92
	CAT(RC(D))	1,82	1,10	2,92
	TAC(C(D))	1,09	1,83	2,92
	GTA(R(D))	1,10	1,82	2,92
TGA	TGA(D)	1,95	1,60	3,54
	TCA(RC(D))	1,97	1,61	3,58
	ACT(C(D))	1,60	1,95	3,54
	AGT(R(D))	1,61	1,97	3,58
TAG	TAG(D)	1,26	1,32	2,57
	CTA(RC(D))	1,25	1,33	2,58
	ATC(C(D))	1,32	1,26	2,57
	GAT(R(D))	1,33	1,25	2,58
TAA	TAA(D)	1,98	2,42	4,40
	TTA(RC(D))	1,98	2,41	4,39
	ATT(C(D))	2,42	1,98	4,40
	AAT(R(D))	2,41	1,98	4,39
AAC	AAC(D)	1,45	1,90	3,36
	GTT(RC(D))	1,46	1,87	3,32
	TTG(C(D))	1,90	1,45	3,36
	CAA(R(D))	1,87	1,46	3,32
AAG	AAG(D)	1,98	2,00	3,97
	CTT(RC(D))	2,01	1,99	4,00
	TTC(C(D))	2,00	1,98	3,97
	GAA(R(D))	1,99	2,01	4,00
ACA	ACA(D)	1,00	1,02	2,02
	TGT(RC(D))	1,02	1,00	2,02
	TGT(C(D))	1,02	1,00	2,02
	ACA(R(D))	1,00	1,02	2,02
ATA	ATA(D)	0,97	0,97	1,94
	TAT(RC(D))	0,97	0,97	1,94
	TAT(C(D))	0,97	0,97	1,94
	ATA(R(D))	0,97	0,97	1,93
AGA	AGA(D)	1,11	1,11	2,22
	TCT(RC(D))	1,11	1,11	2,22
	TCT(C(D))	1,11	1,11	2,22
	AGA(R(D))	1,11	1,11	2,22
AAA	AAA(D)	1,87	1,89	3,76
	TTT(RC(D))	1,89	1,87	3,76
	TTT(C(D))	1,89	1,87	3,76
	AAA(R(D))	1,87	1,89	3,76

Composition of DNA sequence in assembled chromosome 10

Base	No.of bases	%
A	38875926	29,06%
C	27639505	20,66%
G	27719976	20,72%
T	39027555	29,17%
N	534460	0,40%
Sum	133797422	100,00%

% of coding bases from complete assembled chromosome 1 (including N bases)

5,35%

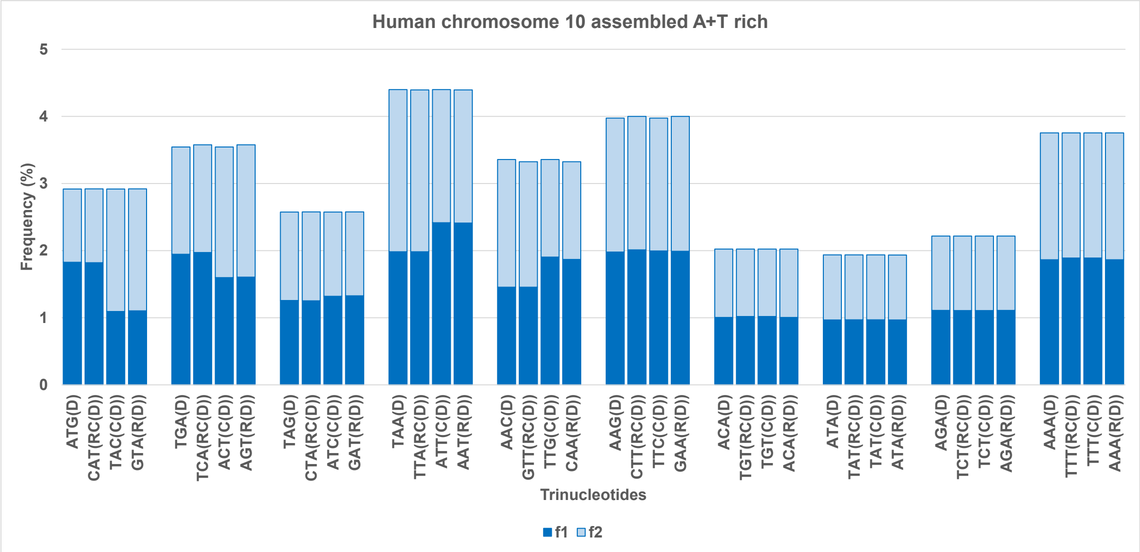

Human random chromomome 10

A-T rich

D	f	%	RC	f	%	C	f	%	R	f	%	
**ATG**	3728,00	1,87	CAT	3604,00	1,81	TAC	2219,00	1,12	GTA	2266,00	1,14	Ia
**TGA**	3824,00	1,92	TCA	3896,00	1,96	ACT	3253,00	1,63	AGT	3241,00	1,63	
**TAG**	2579,00	1,30	CTA	2580,00	1,30	ATC	2630,00	1,32	GAT	2616,00	1,31	
**TAA**	4125,00	2,07	TTA	4032,00	2,03	ATT	4924,00	2,47	AAT	5107,00	2,57	Ib
**AAC**	2958,00	1,49	GTT	2901,00	1,46	TTG	3833,00	1,93	CAA	3765,00	1,89	
**AAG**	3996,00	2,01	CTT	4031,00	2,03	TTC	3992,00	2,01	GAA	4170,00	2,10	
**ACA**	4047,00	2,03	TGT	4061,00	2,04	TGT*			ACA*			Ic
**ATA**	4092,00	2,06	TAT	4047,00	2,03	TAT*			ATA*			
**AGA**	4450,00	2,24	TCT	4366,00	2,19	TCT*			AGA*			
**AAA**	8003,00	4,02	TTT	7692,00	3,87	TTT*			AAA*			

Quadruplet		f1	f2	fu
ATG	ATG(D)	1,87	1,12	2,99
	CAT(RC(D))	1,81	1,14	2,95
	TAC(C(D))	1,12	1,87	2,99
	GTA(R(D))	1,14	1,81	2,95
TGA	TGA(D)	1,92	1,63	3,56
	TCA(RC(D))	1,96	1,63	3,59
	ACT(C(D))	1,63	1,92	3,56
	AGT(R(D))	1,63	1,96	3,59
TAG	TAG(D)	1,30	1,32	2,62
	CTA(RC(D))	1,30	1,31	2,61
	ATC(C(D))	1,32	1,30	2,62
	GAT(R(D))	1,31	1,30	2,61
TAA	TAA(D)	2,07	2,47	4,55
	TTA(RC(D))	2,03	2,57	4,59
	ATT(C(D))	2,47	2,07	4,55
	AAT(R(D))	2,57	2,03	4,59
AAC	AAC(D)	1,49	1,93	3,41
	GTT(RC(D))	1,46	1,89	3,35
	TTG(C(D))	1,93	1,49	3,41
	CAA(R(D))	1,89	1,46	3,35
AAG	AAG(D)	2,01	2,01	4,01
	CTT(RC(D))	2,03	2,10	4,12
	TTC(C(D))	2,01	2,01	4,01
	GAA(R(D))	2,10	2,03	4,12
ACA	ACA(D)	1,02	1,02	2,04
	TGT(RC(D))	1,02	1,02	2,04
	TGT(C(D))	1,02	1,02	2,04
	ACA(R(D))	1,02	1,02	2,04
ATA	ATA(D)	1,03	1,02	2,05
	TAT(RC(D))	1,02	1,03	2,05
	TAT(C(D))	1,02	1,03	2,05
	ATA(R(D))	1,03	1,03	2,06
AGA	AGA(D)	1,12	1,10	2,22
	TCT(RC(D))	1,10	1,12	2,22
	TCT(C(D))	1,10	1,12	2,22
	AGA(R(D))	1,12	1,10	2,22
AAA	AAA(D)	2,01	1,93	3,94
	TTT(RC(D))	1,93	2,01	3,94
	TTT(C(D))	1,93	2,01	3,94
	AAA(R(D))	2,01	1,93	3,94

Composition of DNA sequence in random chromosome 10

Base	No.of bases	%
A	59400	29,70%
C	40230	20,12%
G	40583	20,29%
T	58781	29,39%
N	1006	0,50%
Sum	200000	100,00%

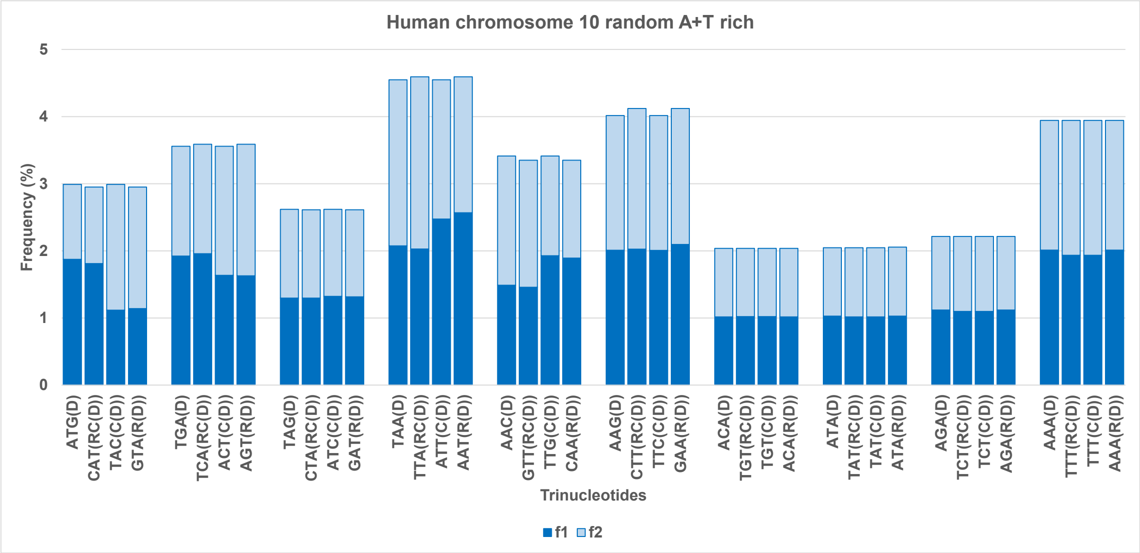

Human coding chromomome 10

A-T rich

D	f	%	RC	f	%	C	f	%	R	f	%	
**ATG**	132636,00	1,85	CAT	114580,00	1,60	TAC	71643,00	1,00	GTA	63042,00	0,88	Ia
**TGA**	142940,00	2,00	TCA	129895,00	1,81	ACT	103134,00	1,44	AGT	106686,00	1,49	
**TAG**	60189,00	0,84	CTA	70709,00	0,99	ATC	92206,00	1,29	GAT	101691,00	1,42	
**TAA**	95413,00	1,33	TTA	97948,00	1,37	ATT	124727,00	1,74	AAT	128845,00	1,80	Ib
**AAC**	101270,00	1,41	GTT	90970,00	1,27	TTG	120804,00	1,69	CAA	131902,00	1,84	
**AAG**	160330,00	2,24	CTT	126755,00	1,77	TTC	123700,00	1,73	GAA	163130,00	2,28	
**ACA**	131583,00	1,84	TGT	130472,00	1,82	TGT*			ACA*			Ic
**ATA**	89957,00	1,26	TAT	94411,00	1,32	TAT*			ATA*			
**AGA**	172412,00	2,41	TCT	128390,00	1,79	TCT*			AGA*			
**AAA**	208107,00	2,91	TTT	186554,00	2,60	TTT*			AAA*			

Quadruplet		f1	f2	fu
ATG	ATG(D)	1,85	1,00	2,85
	CAT(RC(D))	1,60	0,88	2,48
	TAC(C(D))	1,00	1,85	2,85
	GTA(R(D))	0,88	1,60	2,48
TGA	TGA(D)	2,00	1,44	3,44
	TCA(RC(D))	1,81	1,49	3,30
	ACT(C(D))	1,44	2,00	3,44
	AGT(R(D))	1,49	1,81	3,30
TAG	TAG(D)	0,84	1,29	2,13
	CTA(RC(D))	0,99	1,42	2,41
	ATC(C(D))	1,29	0,84	2,13
	GAT(R(D))	1,42	0,99	2,41
TAA	TAA(D)	1,33	1,74	3,07
	TTA(RC(D))	1,37	1,80	3,17
	ATT(C(D))	1,74	1,33	3,07
	AAT(R(D))	1,80	1,37	3,17
AAC	AAC(D)	1,41	1,69	3,10
	GTT(RC(D))	1,27	1,84	3,11
	TTG(C(D))	1,69	1,41	3,10
	CAA(R(D))	1,84	1,27	3,11
AAG	AAG(D)	2,24	1,73	3,97
	CTT(RC(D))	1,77	2,28	4,05
	TTC(C(D))	1,73	2,24	3,97
	GAA(R(D))	2,28	1,77	4,05
ACA	ACA(D)	0,92	0,91	1,83
	TGT(RC(D))	0,91	0,92	1,83
	TGT(C(D))	0,91	0,92	1,83
	ACA(R(D))	0,92	0,91	1,83
ATA	ATA(D)	0,63	0,66	1,29
	TAT(RC(D))	0,66	0,63	1,29
	TAT(C(D))	0,66	0,63	1,29
	ATA(R(D))	0,63	0,63	1,26
AGA	AGA(D)	1,20	0,90	2,10
	TCT(RC(D))	0,90	1,20	2,10
	TCT(C(D))	0,90	1,20	2,10
	AGA(R(D))	1,20	0,90	2,10
AAA	AAA(D)	1,45	1,30	2,76
	TTT(RC(D))	1,30	1,45	2,76
	TTT(C(D))	1,30	1,45	2,76
	AAA(R(D))	1,45	1,30	2,76

Composition of DNA sequence in coding chromosome 10

Base	No.of bases	%
A	1955837	27,31%
C	1678862	23,44%
G	1717729	23,98%
T	1810197	25,27%
N	0	0,00%
Sum	7162625	100,00%

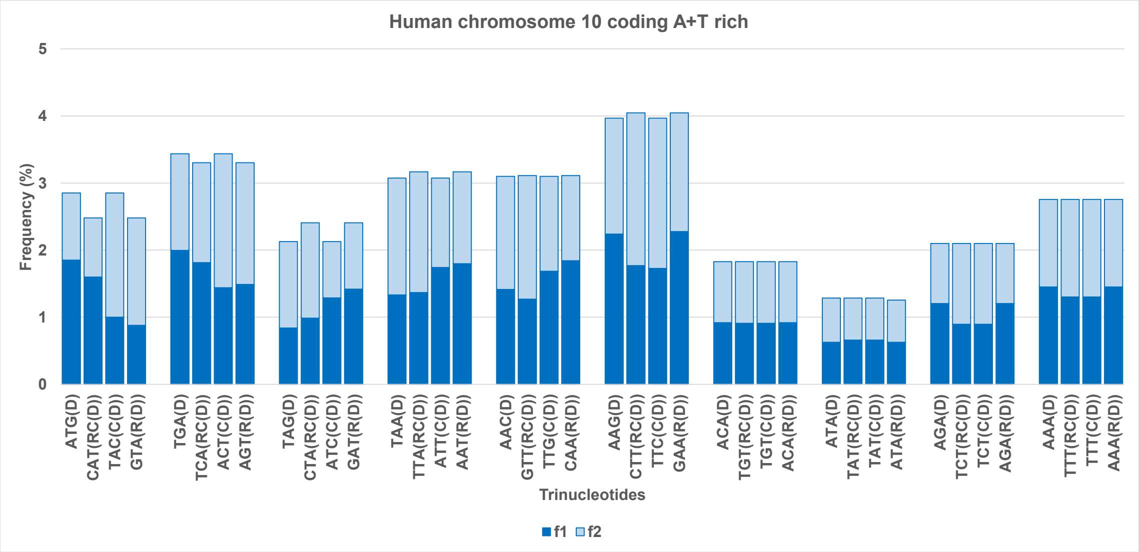

C+G rich											
**D**	**f**	**%**	**RC**	**f**	**%**	**C**	**f**	**%**	**R**	**f**	**%**
**CGT**	361899,00	0,27	ACG	356171,00	0,27	GCA	1970109,00	1,48	TGC	1969598,00	1,48
**GTC**	1274690,00	0,96	GAC	1279986,00	0,96	CAG	2793801,00	2,10	CTG	2761341,00	2,07
**GCT**	1900077,00	1,43	AGC	1905781,00	1,43	CGA	311224,00	0,23	TCG	320735,00	0,24
**GCC**	1636839,00	1,23	GGC	1633111,00	1,23	CGG	384477,00	0,29	CCG	379229,00	0,28
**CCA**	2486996,00	1,87	TGG	2536065,00	1,90	GGT	1550914,00	1,16	ACC	1564123,00	1,17
**CCT**	2419916,00	1,82	AGG	2401511,00	1,80	GGA	2138026,00	1,60	TCC	2085178,00	1,56
**CAC**	2054793,00	1,54	GTG	2079511,00	1,56	GTG*			CAC*		
**CGC**	331378,00	0,25	GCG	332842,00	0,25	GCG*			CGC*		
**CTC**	2294602,00	1,72	GAG	2299989,00	1,73	GAG*			CTC*		
**CCC**	1799633,00	1,35	GGG	1796161,00	1,35	GGG*			CCC*		

Quadruplet		f1	f2	fu
CGT	CGT(D)	0,27	1,48	1,75
	ACG(RC(D))	0,27	1,48	1,75
	GCA(C(D))	1,48	0,27	1,75
	TGC(R(D))	1,48	0,27	1,75
GTC	GTC(D)	0,96	2,10	3,05
	GAC(RC(D))	0,96	2,07	3,03
	CAG(C(D))	2,10	0,96	3,05
	CTG(R(D))	2,07	0,96	3,03
GCT	GCT(D)	1,43	0,23	1,66
	AGC(RC(D))	1,43	0,24	1,67
	CGA(C(D))	0,23	1,43	1,66
	TCG(R(D))	0,24	1,43	1,67
GCC	GCC(D)	1,23	0,29	1,52
	GGC(RC(D))	1,23	0,28	1,51
	CGG(C(D))	0,29	1,23	1,52
	CCG(R(D))	0,28	1,23	1,51
CCA	CCA(D)	1,87	1,16	3,03
	TGG(RC(D))	1,90	1,17	3,08
	GGT(C(D))	1,16	1,87	3,03
	ACC(R(D))	1,17	1,90	3,08
CCT	CCT(D)	1,82	1,60	3,42
	AGG(RC(D))	1,80	1,56	3,37
	GGA(C(D))	1,60	1,82	3,42
	TCC(R(D))	1,56	1,80	3,37
CAC	CAC(D)	0,77	0,78	1,55
	GTG(RC(D))	0,78	0,77	1,55
	GTG(C(D))	0,78	0,77	1,55
	CAC(R(D))	0,77	0,78	1,55
CGC	CGC(D)	0,12	0,12	0,25
	GCG(RC(D))	0,12	0,12	0,25
	GCG(C(D))	0,12	0,12	0,25
	CGC(R(D))	0,12	0,12	0,25
CTC	CTC(D)	0,86	0,86	1,72
	GAG(RC(D))	0,86	0,86	1,72
	GAG(C(D))	0,86	0,86	1,72
	CTC(R(D))	0,86	0,86	1,72
CCC	CCC(D)	0,68	0,67	1,35
	GGG(RC(D))	0,67	0,68	1,35
	GGG(C(D))	0,67	0,68	1,35
	CCC(R(D))	0,68	0,67	1,35

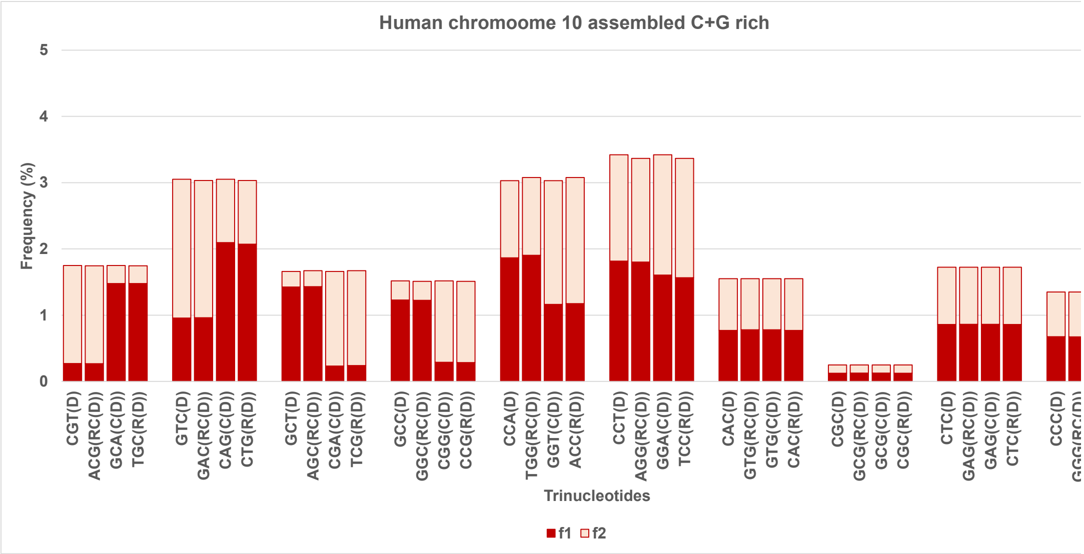

Human random chromomome 10

C+G rich

D	f	%	RC	f	%	C	f	%	R	f	%
**CGT**	544,00	0,27	ACG	511,00	0,26	GCA	2869,00	1,44	TGC	3000,00	1,51
**GTC**	1804,00	0,91	GAC	1866,00	0,94	CAG	3980,00	2,00	CTG	4015,00	2,02
**GCT**	2842,00	1,43	AGC	2746,00	1,38	CGA	458,00	0,23	TCG	489,00	0,25
**GCC**	2316,00	1,16	GGC	2284,00	1,15	CGG	522,00	0,26	CCG	521,00	0,26
**CCA**	3560,00	1,79	TGG	3679,00	1,85	GGT	2115,00	1,06	ACC	2254,00	1,13
**CCT**	3433,00	1,73	AGG	3456,00	1,74	GGA	3258,00	1,64	TCC	2945,00	1,48
**CAC**	3024,00	1,52	GTG	2990,00	1,50	GTG*			CAC*		
**CGC**	472,00	0,24	GCG	475,00	0,24	GCG*			CGC*		
**CTC**	3268,00	1,64	GAG	3338,00	1,68	GAG*			CTC*		
**CCC**	2449,00	1,23	GGG	2469,00	1,24	GGG*			CCC*		

Quadruplet		f1	f2	fu
CGT	CGT(D)	0,27	1,44	1,72
	ACG(RC(D))	0,26	1,51	1,76
	GCA(C(D))	1,44	0,27	1,72
	TGC(R(D))	1,51	0,26	1,76
GTC	GTC(D)	0,91	2,00	2,91
	GAC(RC(D))	0,94	2,02	2,96
	CAG(C(D))	2,00	0,91	2,91
	CTG(R(D))	2,02	0,94	2,96
GCT	GCT(D)	1,43	0,23	1,66
	AGC(RC(D))	1,38	0,25	1,63
	CGA(C(D))	0,23	1,43	1,66
	TCG(R(D))	0,25	1,38	1,63
GCC	GCC(D)	1,16	0,26	1,43
	GGC(RC(D))	1,15	0,26	1,41
	CGG(C(D))	0,26	1,16	1,43
	CCG(R(D))	0,26	1,15	1,41
CCA	CCA(D)	1,79	1,06	2,85
	TGG(RC(D))	1,85	1,13	2,98
	GGT(C(D))	1,06	1,79	2,85
	ACC(R(D))	1,13	1,85	2,98
CCT	CCT(D)	1,73	1,64	3,36
	AGG(RC(D))	1,74	1,48	3,22
	GGA(C(D))	1,64	1,73	3,36
	TCC(R(D))	1,48	1,74	3,22
CAC	CAC(D)	0,76	0,75	1,51
	GTG(RC(D))	0,75	0,76	1,51
	GTG(C(D))	0,75	0,76	1,51
	CAC(R(D))	0,76	0,75	1,51
CGC	CGC(D)	0,12	0,12	0,24
	GCG(RC(D))	0,12	0,12	0,24
	GCG(C(D))	0,12	0,12	0,24
	CGC(R(D))	0,12	0,12	0,24
CTC	CTC(D)	0,82	0,84	1,66
	GAG(RC(D))	0,84	0,82	1,66
	GAG(C(D))	0,84	0,82	1,66
	CTC(R(D))	0,82	0,84	1,66
CCC	CCC(D)	0,62	0,62	1,24
	GGG(RC(D))	0,62	0,62	1,24
	GGG(C(D))	0,62	0,62	1,24
	CCC(R(D))	0,62	0,62	1,24

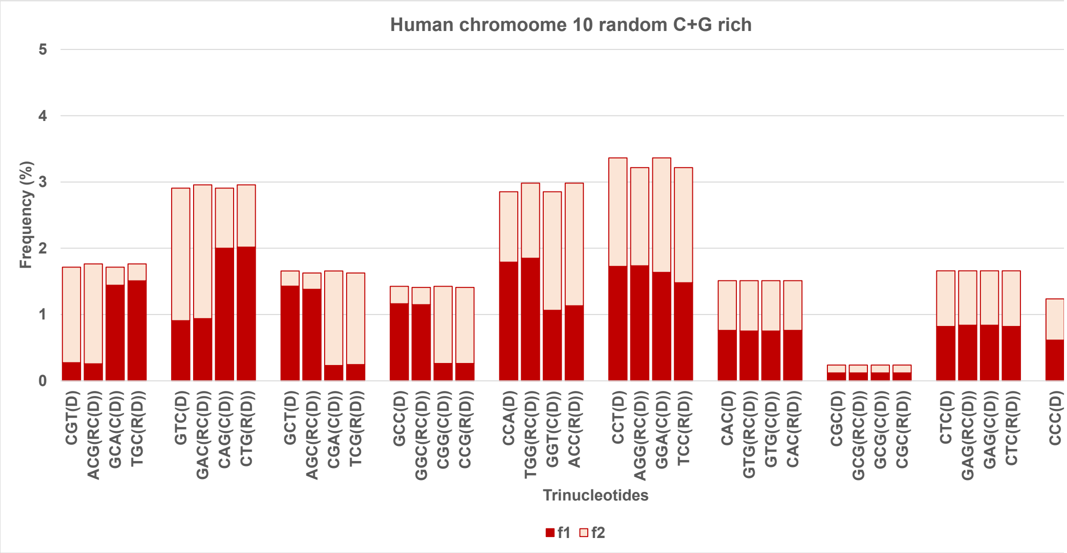

Human coding chromomome 10

C+G rich

D	f	%	RC	f	%	C	f	%	R	f	%
**CGT**	33489,00	0,47	ACG	37438,00	0,52	GCA	121147,00	1,69	TGC	122350,00	1,71
**GTC**	75249,00	1,05	GAC	92544,00	1,29	CAG	179393,00	2,50	CTG	173103,00	2,42
**GCT**	119937,00	1,67	AGC	130349,00	1,82	CGA	38423,00	0,54	TCG	33592,00	0,47
**GCC**	126015,00	1,76	GGC	115371,00	1,61	CGG	56329,00	0,79	CCG	56239,00	0,79
**CCA**	152300,00	2,13	TGG	151249,00	2,11	GGT	79082,00	1,10	ACC	102352,00	1,43
**CCT**	140474,00	1,96	AGG	133804,00	1,87	GGA	146929,00	2,05	TCC	120646,00	1,68
**CAC**	109050,00	1,52	GTG	120468,00	1,68	GTG*			CAC*		
**CGC**	46708,00	0,65	GCG	47679,00	0,67	GCG*			CGC*		
**CTC**	121368,00	1,69	GAG	143339,00	2,00	GAG*			CTC*		
**CCC**	128040,00	1,79	GGG	111136,00	1,55	GGG*			CCC*		

Quadruplet		f1	f2	fu
CGT	CGT(D)	0,47	1,69	2,16
	ACG(RC(D))	0,52	1,71	2,23
	GCA(C(D))	1,69	0,47	2,16
	TGC(R(D))	1,71	0,52	2,23
GTC	GTC(D)	1,05	2,50	3,56
	GAC(RC(D))	1,29	2,42	3,71
	CAG(C(D))	2,50	1,05	3,56
	CTG(R(D))	2,42	1,29	3,71
GCT	GCT(D)	1,67	0,54	2,21
	AGC(RC(D))	1,82	0,47	2,29
	CGA(C(D))	0,54	1,67	2,21
	TCG(R(D))	0,47	1,82	2,29
GCC	GCC(D)	1,76	0,79	2,55
	GGC(RC(D))	1,61	0,79	2,40
	CGG(C(D))	0,79	1,76	2,55
	CCG(R(D))	0,79	1,61	2,40
CCA	CCA(D)	2,13	1,10	3,23
	TGG(RC(D))	2,11	1,43	3,54
	GGT(C(D))	1,10	2,13	3,23
	ACC(R(D))	1,43	2,11	3,54
CCT	CCT(D)	1,96	2,05	4,01
	AGG(RC(D))	1,87	1,68	3,55
	GGA(C(D))	2,05	1,96	4,01
	TCC(R(D))	1,68	1,87	3,55
CAC	CAC(D)	0,76	0,84	1,60
	GTG(RC(D))	0,84	0,76	1,60
	GTG(C(D))	0,84	0,76	1,60
	CAC(R(D))	0,76	0,84	1,60
CGC	CGC(D)	0,33	0,33	0,66
	GCG(RC(D))	0,33	0,33	0,66
	GCG(C(D))	0,33	0,33	0,66
	CGC(R(D))	0,33	0,33	0,66
CTC	CTC(D)	0,85	1,00	1,85
	GAG(RC(D))	1,00	0,85	1,85
	GAG(C(D))	1,00	0,85	1,85
	CTC(R(D))	0,85	1,00	1,85
CCC	CCC(D)	0,89	0,78	1,67
	GGG(RC(D))	0,78	0,89	1,67
	GGG(C(D))	0,78	0,89	1,67
	CCC(R(D))	0,89	0,78	1,67

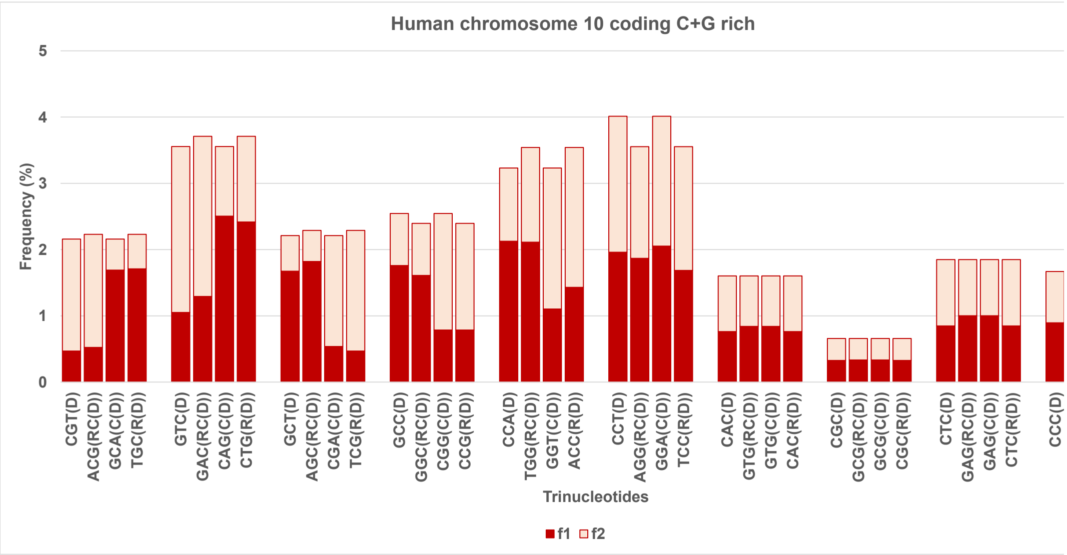

IIa

IIb

IIc

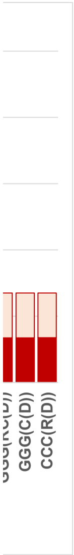

IIa

IIb

IIc

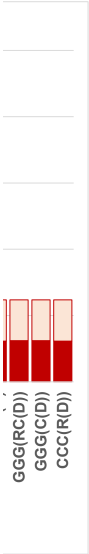

IIa

IIb

IIc

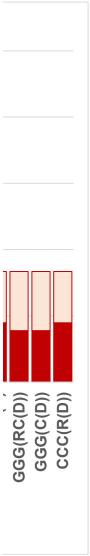
GGG(RC(D))
GGG(C(D))
CCC(R(D))

Human assembled chromomome 11

A-T rich												
**D**	**f**	**%**	**RC**	**f**	**%**	**C**	**f**	**%**	**R**	**f**	**%**	
**ATG**	2426068,00	1,80	CAT	2459057,00	1,83	TAC	1475168,00	1,10	GTA	1488038,00	1,11	Ia
**TGA**	2648218,00	1,97	TCA	2641927,00	1,96	ACT	2188830,00	1,63	AGT	2143856,00	1,59	
**TAG**	1736164,00	1,29	CTA	1730466,00	1,29	ATC	1794242,00	1,33	GAT	1824997,00	1,36	
**TAA**	2656864,00	1,97	TTA	2636499,00	1,96	ATT	3245135,00	2,41	AAT	3194583,00	2,37	Ib
**AAC**	1993601,00	1,48	GTT	1936677,00	1,44	TTG	2569084,00	1,91	CAA	2506256,00	1,86	
**AAG**	2675746,00	1,99	CTT	2739907,00	2,04	TTC	2716138,00	2,02	GAA	2700809,00	2,01	
**ACA**	2694701,00	2,00	TGT	2693643,00	2,00	TGT*			ACA*			Ic
**ATA**	2660181,00	1,98	TAT	2646989,00	1,97	TAT*			ATA*			
**AGA**	3022762,00	2,25	TCT	3037555,00	2,26	TCT*			AGA*			
**AAA**	4956058,00	3,68	TTT	4967748,00	3,69	TTT*			AAA*			

Quadruplet		f1	f2	fu
ATG	ATG(D)	1,80	1,10	2,90
	CAT(RC(D))	1,83	1,11	2,93
	TAC(C(D))	1,10	1,80	2,90
	GTA(R(D))	1,11	1,83	2,93
TGA	TGA(D)	1,97	1,63	3,60
	TCA(RC(D))	1,96	1,59	3,56
	ACT(C(D))	1,63	1,97	3,60
	AGT(R(D))	1,59	1,96	3,56
TAG	TAG(D)	1,29	1,33	2,62
	CTA(RC(D))	1,29	1,36	2,64
	ATC(C(D))	1,33	1,29	2,62
	GAT(R(D))	1,36	1,29	2,64
TAA	TAA(D)	1,97	2,41	4,39
	TTA(RC(D))	1,96	2,37	4,33
	ATT(C(D))	2,41	1,97	4,39
	AAT(R(D))	2,37	1,96	4,33
AAC	AAC(D)	1,48	1,91	3,39
	GTT(RC(D))	1,44	1,86	3,30
	TTG(C(D))	1,91	1,48	3,39
	CAA(R(D))	1,86	1,44	3,30
AAG	AAG(D)	1,99	2,02	4,01
	CTT(RC(D))	2,04	2,01	4,04
	TTC(C(D))	2,02	1,99	4,01
	GAA(R(D))	2,01	2,04	4,04
ACA	ACA(D)	1,00	1,00	2,00
	TGT(RC(D))	1,00	1,00	2,00
	TGT(C(D))	1,00	1,00	2,00
	ACA(R(D))	1,00	1,00	2,00
ATA	ATA(D)	0,99	0,98	1,97
	TAT(RC(D))	0,98	0,99	1,97
	TAT(C(D))	0,98	0,99	1,97
	ATA(R(D))	0,99	0,99	1,98
AGA	AGA(D)	1,12	1,13	2,25
	TCT(RC(D))	1,13	1,12	2,25
	TCT(C(D))	1,13	1,12	2,25
	AGA(R(D))	1,12	1,13	2,25
AAA	AAA(D)	1,84	1,85	3,69
	TTT(RC(D))	1,85	1,84	3,69
	TTT(C(D))	1,85	1,84	3,69
	AAA(R(D))	1,84	1,85	3,69

Composition of DNA sequence in assembled chromosome 11

Base	No.of bases	%
A	39286730	29,08%
C	27903257	20,66%
G	27981801	20,71%
T	39361954	29,14%
N	552880	0,41%
Sum	135086622	100,00%

% of coding bases from complete assembled chromosome 1 (including N bases)

7,54%

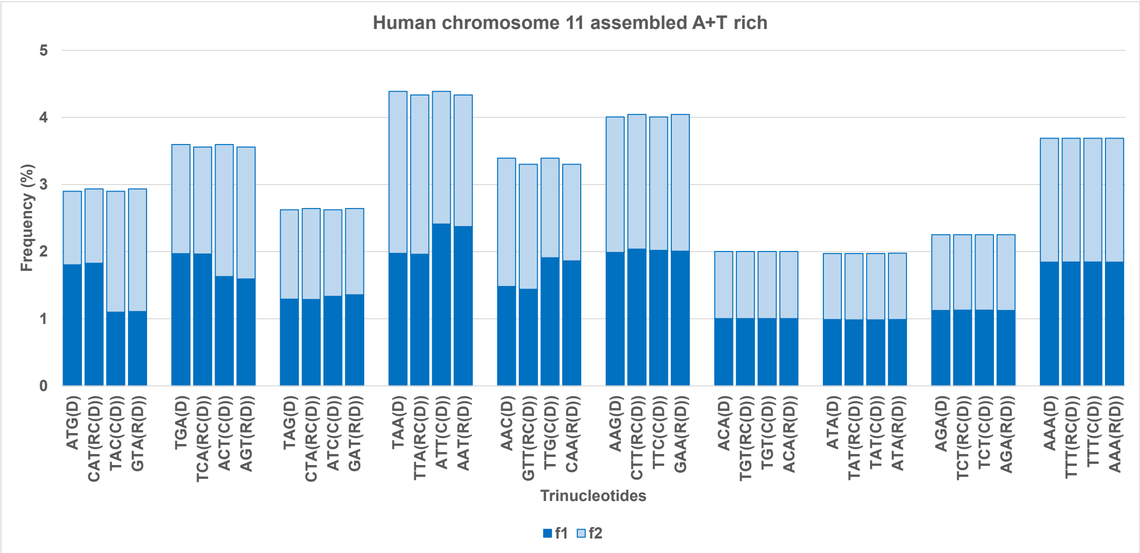

Human random chromomome 11

A-T rich

D	f	%	RC	f	%	C	f	%	R	f	%	
**ATG**	3425,00	1,72	CAT	3535,00	1,78	TAC	2077,00	1,05	GTA	2084,00	1,05	Ia
**TGA**	3893,00	1,96	TCA	3804,00	1,92	ACT	3253,00	1,64	AGT	3094,00	1,56	
**TAG**	2478,00	1,25	CTA	2549,00	1,28	ATC	2568,00	1,29	GAT	2637,00	1,33	
**TAA**	3676,00	1,85	TTA	3501,00	1,76	ATT	4570,00	2,30	AAT	4489,00	2,26	Ib
**AAC**	2981,00	1,50	GTT	2712,00	1,37	TTG	3742,00	1,88	CAA	3635,00	1,83	
**AAG**	3906,00	1,97	CTT	3997,00	2,01	TTC	4037,00	2,03	GAA	4066,00	2,05	
**ACA**	4010,00	2,02	TGT	3908,00	1,97	TGT*			ACA*			Ic
**ATA**	3691,00	1,86	TAT	3594,00	1,81	TAT*			ATA*			
**AGA**	4447,00	2,24	TCT	4490,00	2,26	TCT*			AGA*			
**AAA**	7247,00	3,65	TTT	7005,00	3,53	TTT*			AAA*			

Quadruplet		f1	f2	fu
ATG	ATG(D)	1,72	1,05	2,77
	CAT(RC(D))	1,78	1,05	2,83
	TAC(C(D))	1,05	1,72	2,77
	GTA(R(D))	1,05	1,78	2,83
TGA	TGA(D)	1,96	1,64	3,60
	TCA(RC(D))	1,92	1,56	3,47
	ACT(C(D))	1,64	1,96	3,60
	AGT(R(D))	1,56	1,92	3,47
TAG	TAG(D)	1,25	1,29	2,54
	CTA(RC(D))	1,28	1,33	2,61
	ATC(C(D))	1,29	1,25	2,54
	GAT(R(D))	1,33	1,28	2,61
TAA	TAA(D)	1,85	2,30	4,15
	TTA(RC(D))	1,76	2,26	4,02
	ATT(C(D))	2,30	1,85	4,15
	AAT(R(D))	2,26	1,76	4,02
AAC	AAC(D)	1,50	1,88	3,39
	GTT(RC(D))	1,37	1,83	3,20
	TTG(C(D))	1,88	1,50	3,39
	CAA(R(D))	1,83	1,37	3,20
AAG	AAG(D)	1,97	2,03	4,00
	CTT(RC(D))	2,01	2,05	4,06
	TTC(C(D))	2,03	1,97	4,00
	GAA(R(D))	2,05	2,01	4,06
ACA	ACA(D)	1,01	0,98	1,99
	TGT(RC(D))	0,98	1,01	1,99
	TGT(C(D))	0,98	1,01	1,99
	ACA(R(D))	1,01	0,98	1,99
ATA	ATA(D)	0,93	0,90	1,83
	TAT(RC(D))	0,90	0,93	1,83
	TAT(C(D))	0,90	0,93	1,83
	ATA(R(D))	0,93	0,93	1,86
AGA	AGA(D)	1,12	1,13	2,25
	TCT(RC(D))	1,13	1,12	2,25
	TCT(C(D))	1,13	1,12	2,25
	AGA(R(D))	1,12	1,13	2,25
AAA	AAA(D)	1,82	1,76	3,59
	TTT(RC(D))	1,76	1,82	3,59
	TTT(C(D))	1,76	1,82	3,59
	AAA(R(D))	1,82	1,76	3,59

Composition of DNA sequence in random chromosome 11

Base	No.of bases	%
A	57205	28,60%
C	42250	21,13%
G	42354	21,18%
T	56791	28,40%
N	1400	0,70%
Sum	200000	100,00%

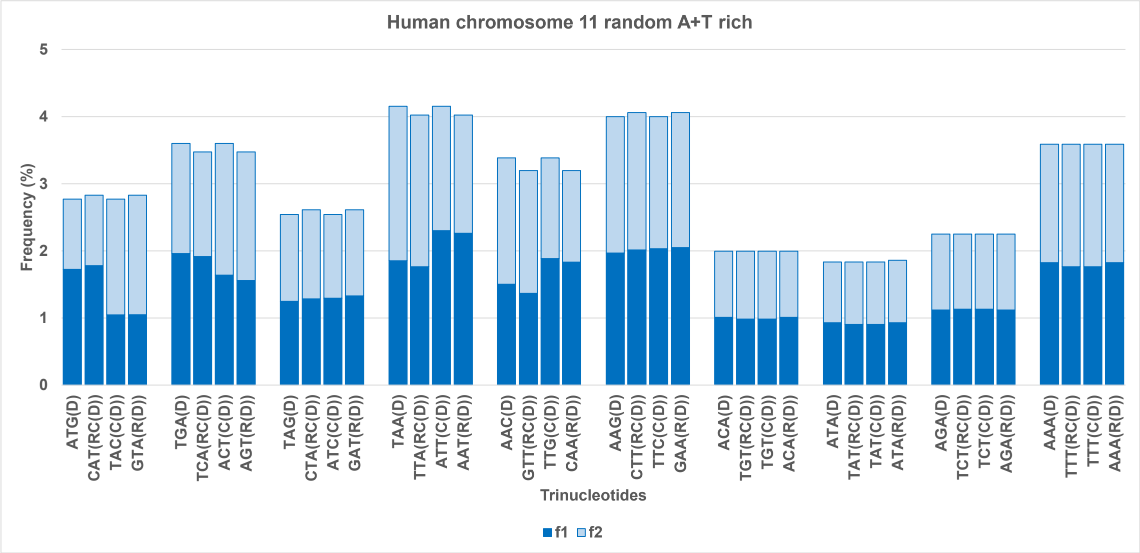

Human coding chromomome 11

A-T rich

D	f	%	RC	f	%	C	f	%	R	f	%	
**ATG**	2426068,00	1,80	CAT	2459057,00	1,83	TAC	1475168,00	1,10	GTA	1488038,00	1,11	Ia
**TGA**	2648218,00	1,97	TCA	2641927,00	1,96	ACT	2188830,00	1,63	AGT	2143856,00	1,59	
**TAG**	1736164,00	1,29	CTA	1730466,00	1,29	ATC	1794242,00	1,33	GAT	1824997,00	1,36	
**TAA**	2656864,00	1,97	TTA	2636499,00	1,96	ATT	3245135,00	2,41	AAT	3194583,00	2,37	Ib
**AAC**	1993601,00	1,48	GTT	1936677,00	1,44	TTG	2569084,00	1,91	CAA	2506256,00	1,86	
**AAG**	2675746,00	1,99	CTT	2739907,00	2,04	TTC	2716138,00	2,02	GAA	2700809,00	2,01	
**ACA**	2694701,00	2,00	TGT	2693643,00	2,00	TGT*			ACA*			Ic
**ATA**	2660181,00	1,98	TAT	2646989,00	1,97	TAT*			ATA*			
**AGA**	3022762,00	2,25	TCT	3037555,00	2,26	TCT*			AGA*			
**AAA**	4956058,00	3,68	TTT	4967748,00	3,69	TTT*			AAA*			

Quadruplet		f1	f2	fu
ATG	ATG(D)	1,80	1,10	2,90
	CAT(RC(D))	1,83	1,11	2,93
	TAC(C(D))	1,10	1,80	2,90
	GTA(R(D))	1,11	1,83	2,93
TGA	TGA(D)	1,97	1,63	3,60
	TCA(RC(D))	1,96	1,59	3,56
	ACT(C(D))	1,63	1,97	3,60
	AGT(R(D))	1,59	1,96	3,56
TAG	TAG(D)	1,29	1,33	2,62
	CTA(RC(D))	1,29	1,36	2,64
	ATC(C(D))	1,33	1,29	2,62
	GAT(R(D))	1,36	1,29	2,64
TAA	TAA(D)	1,97	2,41	4,39
	TTA(RC(D))	1,96	2,37	4,33
	ATT(C(D))	2,41	1,97	4,39
	AAT(R(D))	2,37	1,96	4,33
AAC	AAC(D)	1,48	1,91	3,39
	GTT(RC(D))	1,44	1,86	3,30
	TTG(C(D))	1,91	1,48	3,39
	CAA(R(D))	1,86	1,44	3,30
AAG	AAG(D)	1,99	2,02	4,01
	CTT(RC(D))	2,04	2,01	4,04
	TTC(C(D))	2,02	1,99	4,01
	GAA(R(D))	2,01	2,04	4,04
ACA	ACA(D)	1,00	1,00	2,00
	TGT(RC(D))	1,00	1,00	2,00
	TGT(C(D))	1,00	1,00	2,00
	ACA(R(D))	1,00	1,00	2,00
ATA	ATA(D)	0,99	0,98	1,97
	TAT(RC(D))	0,98	0,99	1,97
	TAT(C(D))	0,98	0,99	1,97
	ATA(R(D))	0,99	0,99	1,98
AGA	AGA(D)	1,12	1,13	2,25
	TCT(RC(D))	1,13	1,12	2,25
	TCT(C(D))	1,13	1,12	2,25
	AGA(R(D))	1,12	1,13	2,25
AAA	AAA(D)	1,84	1,85	3,69
	TTT(RC(D))	1,85	1,84	3,69
	TTT(C(D))	1,85	1,84	3,69
	AAA(R(D))	1,84	1,85	3,69

Composition of DNA sequence in coding chromosome 11

Base	No.of bases	%
A	2533443	24,87%
C	2627487	25,79%
G	2608384	25,60%
T	2418038	23,74%
N	0	0,00%
Sum	10187352	100,00%

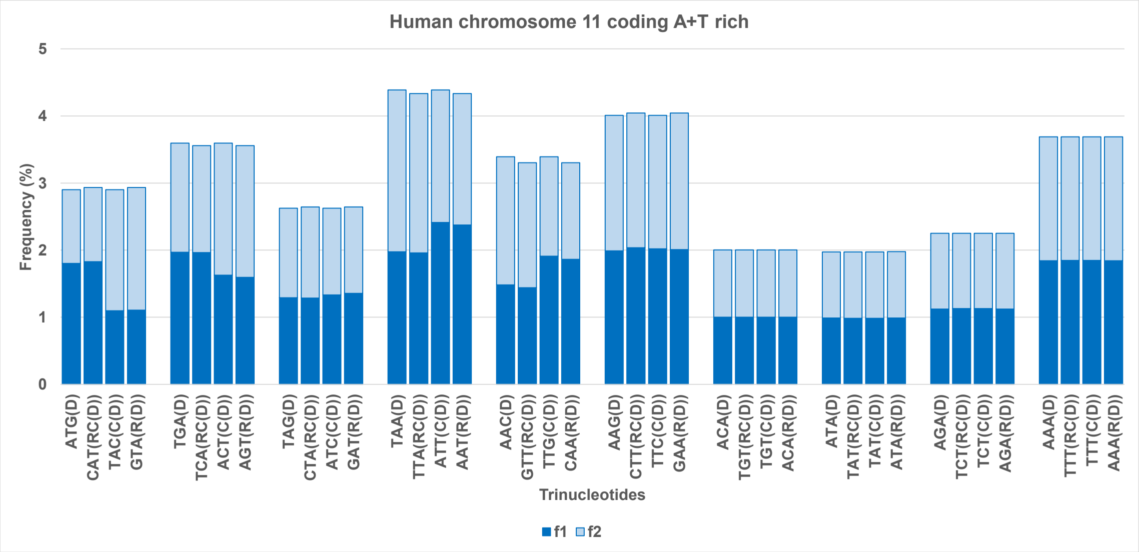

					C+G rich						
**D**	**f**	**%**	**RC**	**f**	**%**	**C**	**f**	**%**	**R**	**f**	**%**
**CGT**	332379,00	0,25	ACG	342671,00	0,25	GCA	1979499,00	1,47	TGC	1963363,00	1,46
**GTC**	1258765,00	0,94	GAC	1296325,00	0,96	CAG	2801616,00	2,08	CTG	2808061,00	2,09
**GCT**	1910926,00	1,42	AGC	1922977,00	1,43	CGA	296390,00	0,22	TCG	300174,00	0,22
**GCC**	1633366,00	1,21	GGC	1642746,00	1,22	CGG	389193,00	0,29	CCG	369821,00	0,27
**CCA**	2486995,00	1,85	TGG	2555184,00	1,90	GGT	1570798,00	1,17	ACC	1575085,00	1,17
**CCT**	2468866,00	1,84	AGG	2450222,00	1,82	GGA	2181054,00	1,62	TCC	2117231,00	1,57
**CAC**	2036193,00	1,51	GTG	2057195,00	1,53	GTG*			CAC*		
**CGC**	315152,00	0,23	GCG	320448,00	0,24	GCG*			CGC*		
**CTC**	2327743,00	1,73	GAG	2326289,00	1,73	GAG*			CTC*		
**CCC**	1835159,00	1,36	GGG	1853857,00	1,38	GGG*			CCC*		

Quadruplet		f1	f2	fu
CGT	CGT(D)	0,25	1,47	1,72
	ACG(RC(D))	0,25	1,46	1,71
	GCA(C(D))	1,47	0,25	1,72
	TGC(R(D))	1,46	0,25	1,71
GTC	GTC(D)	0,94	2,08	3,02
	GAC(RC(D))	0,96	2,09	3,05
	CAG(C(D))	2,08	0,94	3,02
	CTG(R(D))	2,09	0,96	3,05
GCT	GCT(D)	1,42	0,22	1,64
	AGC(RC(D))	1,43	0,22	1,65
	CGA(C(D))	0,22	1,42	1,64
	TCG(R(D))	0,22	1,43	1,65
GCC	GCC(D)	1,21	0,29	1,50
	GGC(RC(D))	1,22	0,27	1,50
	CGG(C(D))	0,29	1,21	1,50
	CCG(R(D))	0,27	1,22	1,50
CCA	CCA(D)	1,85	1,17	3,02
	TGG(RC(D))	1,90	1,17	3,07
	GGT(C(D))	1,17	1,85	3,02
	ACC(R(D))	1,17	1,90	3,07
CCT	CCT(D)	1,84	1,62	3,46
	AGG(RC(D))	1,82	1,57	3,40
	GGA(C(D))	1,62	1,84	3,46
	TCC(R(D))	1,57	1,82	3,40
CAC	CAC(D)	0,76	0,76	1,52
	GTG(RC(D))	0,76	0,76	1,52
	GTG(C(D))	0,76	0,76	1,52
	CAC(R(D))	0,76	0,76	1,52
CGC	CGC(D)	0,12	0,12	0,24
	GCG(RC(D))	0,12	0,12	0,24
	GCG(C(D))	0,12	0,12	0,24
	CGC(R(D))	0,12	0,12	0,24
CTC	CTC(D)	0,87	0,86	1,73
	GAG(RC(D))	0,86	0,87	1,73
	GAG(C(D))	0,86	0,87	1,73
	CTC(R(D))	0,87	0,86	1,73
CCC	CCC(D)	0,68	0,69	1,37
	GGG(RC(D))	0,69	0,68	1,37
	GGG(C(D))	0,69	0,68	1,37
	CCC(R(D))	0,68	0,69	1,37

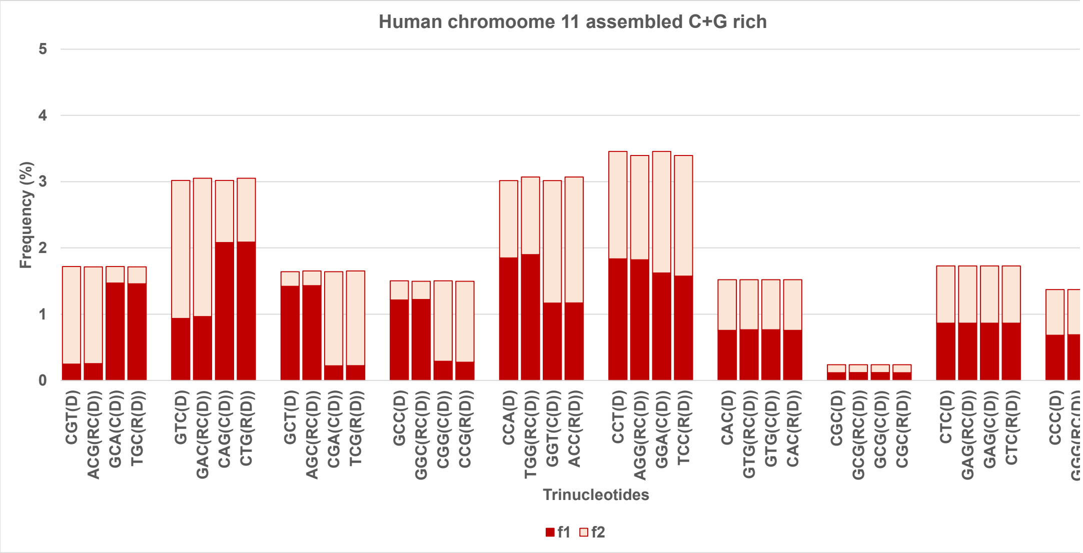

Human random chromomome 11

C+G rich											
D	f	%	RC	f	%	C	f	%	R	f	%
**CGT**	511,00	0,26	ACG	546,00	0,27	GCA	3028,00	1,52	TGC	2953,00	1,49
**GTC**	1845,00	0,93	GAC	2009,00	1,01	CAG	4233,00	2,13	CTG	4340,00	2,19
**GCT**	2861,00	1,44	AGC	2887,00	1,45	CGA	516,00	0,26	TCG	490,00	0,25
**GCC**	2581,00	1,30	GGC	2651,00	1,33	CGG	695,00	0,35	CCG	665,00	0,33
**CCA**	3674,00	1,85	TGG	3931,00	1,98	GGT	2307,00	1,16	ACC	2371,00	1,19
**CCT**	3824,00	1,93	AGG	3716,00	1,87	GGA	3384,00	1,70	TCC	3210,00	1,62
**CAC**	3113,00	1,57	GTG	3178,00	1,60	GTG*			CAC*		
**CGC**	551,00	0,28	GCG	572,00	0,29	GCG*			CGC*		
**CTC**	3542,00	1,78	GAG	3527,00	1,78	GAG*			CTC*		
**CCC**	2870,00	1,45	GGG	2910,00	1,47	GGG*			CCC*		

Quadruplet		f1	f2	fu
CGT	CGT(D)	0,26	1,52	1,78
	ACG(RC(D))	0,27	1,49	1,76
	GCA(C(D))	1,52	0,26	1,78
	TGC(R(D))	1,49	0,27	1,76
GTC	GTC(D)	0,93	2,13	3,06
	GAC(RC(D))	1,01	2,19	3,20
	CAG(C(D))	2,13	0,93	3,06
	CTG(R(D))	2,19	1,01	3,20
GCT	GCT(D)	1,44	0,26	1,70
	AGC(RC(D))	1,45	0,25	1,70
	CGA(C(D))	0,26	1,44	1,70
	TCG(R(D))	0,25	1,45	1,70
GCC	GCC(D)	1,30	0,35	1,65
	GGC(RC(D))	1,33	0,33	1,67
	CGG(C(D))	0,35	1,30	1,65
	CCG(R(D))	0,33	1,33	1,67
CCA	CCA(D)	1,85	1,16	3,01
	TGG(RC(D))	1,98	1,19	3,17
	GGT(C(D))	1,16	1,85	3,01
	ACC(R(D))	1,19	1,98	3,17
CCT	CCT(D)	1,93	1,70	3,63
	AGG(RC(D))	1,87	1,62	3,49
	GGA(C(D))	1,70	1,93	3,63
	TCC(R(D))	1,62	1,87	3,49
CAC	CAC(D)	0,78	0,80	1,58
	GTG(RC(D))	0,80	0,78	1,58
	GTG(C(D))	0,80	0,78	1,58
	CAC(R(D))	0,78	0,80	1,58
CGC	CGC(D)	0,14	0,14	0,28
	GCG(RC(D))	0,14	0,14	0,28
	GCG(C(D))	0,14	0,14	0,28
	CGC(R(D))	0,14	0,14	0,28
CTC	CTC(D)	0,89	0,89	1,78
	GAG(RC(D))	0,89	0,89	1,78
	GAG(C(D))	0,89	0,89	1,78
	CTC(R(D))	0,89	0,89	1,78
CCC	CCC(D)	0,72	0,73	1,46
	GGG(RC(D))	0,73	0,72	1,46
	GGG(C(D))	0,73	0,72	1,46
	CCC(R(D))	0,72	0,73	1,46

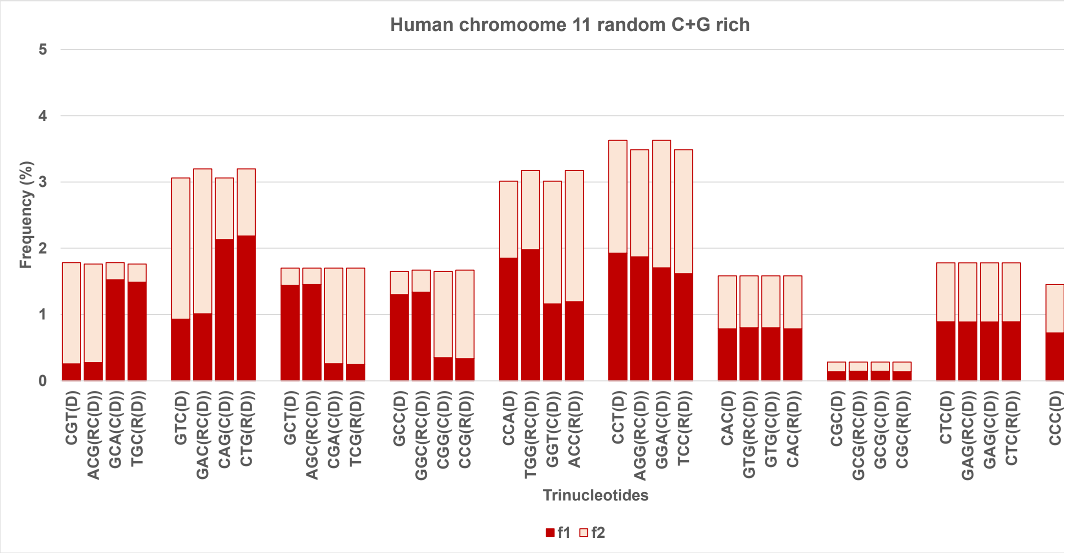

Human coding chromomome 11

C+G rich

D	f	%	RC	f	%	C	f	%	R	f	%
**CGT**	332379,00	0,25	ACG	342671,00	0,25	GCA	1979499,00	1,47	TGC	1963363,00	1,46
**GTC**	1258765,00	0,94	GAC	1296325,00	0,96	CAG	2801616,00	2,08	CTG	2808061,00	2,09
**GCT**	1910926,00	1,42	AGC	1922977,00	1,43	CGA	296390,00	0,22	TCG	300174,00	0,22
**GCC**	1633366,00	1,21	GGC	1642746,00	1,22	CGG	389193,00	0,29	CCG	369821,00	0,27
**CCA**	2486995,00	1,85	TGG	2555184,00	1,90	GGT	1570798,00	1,17	ACC	1575085,00	1,17
**CCT**	2468866,00	1,84	AGG	2450222,00	1,82	GGA	2181054,00	1,62	TCC	2117231,00	1,57
**CAC**	2036193,00	1,51	GTG	2057195,00	1,53	GTG*			CAC*		
**CGC**	315152,00	0,23	GCG	320448,00	0,24	GCG*			CGC*		
**CTC**	2327743,00	1,73	GAG	2326289,00	1,73	GAG*			CTC*		
**CCC**	1835159,00	1,36	GGG	1853857,00	1,38	GGG*			CCC*		

Quadruplet		f1	f2	fu
CGT	CGT(D)	0,25	1,47	1,72
	ACG(RC(D))	0,25	1,46	1,71
	GCA(C(D))	1,47	0,25	1,72
	TGC(R(D))	1,46	0,25	1,71
GTC	GTC(D)	0,94	2,08	3,02
	GAC(RC(D))	0,96	2,09	3,05
	CAG(C(D))	2,08	0,94	3,02
	CTG(R(D))	2,09	0,96	3,05
GCT	GCT(D)	1,42	0,22	1,64
	AGC(RC(D))	1,43	0,22	1,65
	CGA(C(D))	0,22	1,42	1,64
	TCG(R(D))	0,22	1,43	1,65
GCC	GCC(D)	1,21	0,29	1,50
	GGC(RC(D))	1,22	0,27	1,50
	CGG(C(D))	0,29	1,21	1,50
	CCG(R(D))	0,27	1,22	1,50
CCA	CCA(D)	1,85	1,17	3,02
	TGG(RC(D))	1,90	1,17	3,07
	GGT(C(D))	1,17	1,85	3,02
	ACC(R(D))	1,17	1,90	3,07
CCT	CCT(D)	1,84	1,62	3,46
	AGG(RC(D))	1,82	1,57	3,40
	GGA(C(D))	1,62	1,84	3,46
	TCC(R(D))	1,57	1,82	3,40
CAC	CAC(D)	0,76	0,76	1,52
	GTG(RC(D))	0,76	0,76	1,52
	GTG(C(D))	0,76	0,76	1,52
	CAC(R(D))	0,76	0,76	1,52
CGC	CGC(D)	0,12	0,12	0,24
	GCG(RC(D))	0,12	0,12	0,24
	GCG(C(D))	0,12	0,12	0,24
	CGC(R(D))	0,12	0,12	0,24
CTC	CTC(D)	0,87	0,86	1,73
	GAG(RC(D))	0,86	0,87	1,73
	GAG(C(D))	0,86	0,87	1,73
	CTC(R(D))	0,87	0,86	1,73
CCC	CCC(D)	0,68	0,69	1,37
	GGG(RC(D))	0,69	0,68	1,37
	GGG(C(D))	0,69	0,68	1,37
	CCC(R(D))	0,68	0,69	1,37

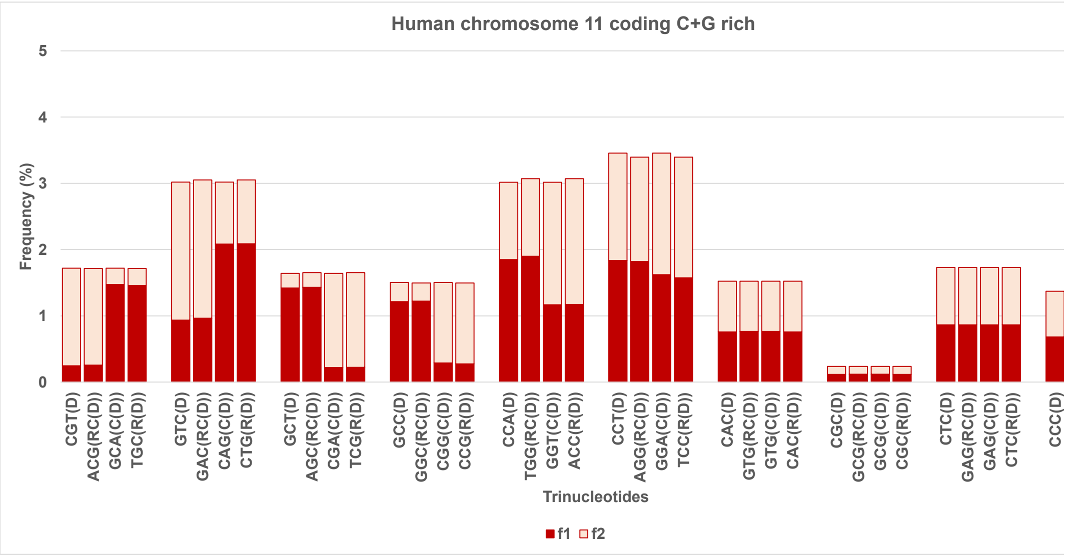

IIa

IIb

IIc

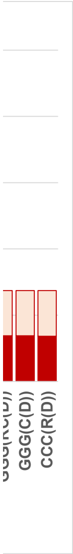

IIa

IIb

IIc

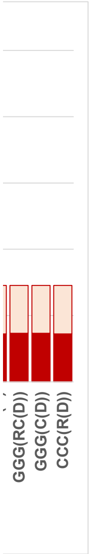

IIa

IIb

IIc

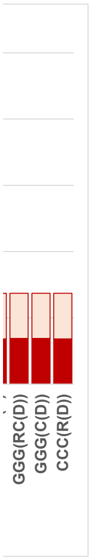
GGG(RC(D))
GGG(C(D))
CCC(R(D))

A-T rich												
D	f	%	RC	f	%	C	f	%	R	f	%	
**ATG**	2414883,00	1,81	CAT	2414851,00	1,81	TAC	1499801,00	1,13	GTA	1523302,00	1,14	Ia
**TGA**	2606665,00	1,96	TCA	2626438,00	1,97	ACT	2162899,00	1,62	AGT	2161398,00	1,62	
**TAG**	1732376,00	1,30	CTA	1720959,00	1,29	ATC	1776695,00	1,33	GAT	1800171,00	1,35	
**TAA**	2764331,00	2,08	TTA	2753677,00	2,07	ATT	3318063,00	2,49	AAT	3293184,00	2,47	Ib
**AAC**	1977691,00	1,49	GTT	1958131,00	1,47	TTG	2562685,00	1,92	CAA	2513595,00	1,89	
**AAG**	2661043,00	2,00	CTT	2706074,00	2,03	TTC	2665907,00	2,00	GAA	2653993,00	1,99	
**ACA**	2662190,00	2,00	TGT	2706674,00	2,03	TGT*			ACA*			Ic
**ATA**	2731126,00	2,05	TAT	2732557,00	2,05	TAT*			ATA*			
**AGA**	2995155,00	2,25	TCT	2971857,00	2,23	TCT*			AGA*			
**AAA**	5135764,00	3,86	TTT	5195879,00	3,90	TTT*			AAA*			

Quadruplet		f1	f2	fu
ATG	ATG(D)	1,81	1,13	2,94
	CAT(RC(D))	1,81	1,14	2,96
	TAC(C(D))	1,13	1,81	2,94
	GTA(R(D))	1,14	1,81	2,96
TGA	TGA(D)	1,96	1,62	3,58
	TCA(RC(D))	1,97	1,62	3,60
	ACT(C(D))	1,62	1,96	3,58
	AGT(R(D))	1,62	1,97	3,60
TAG	TAG(D)	1,30	1,33	2,64
	CTA(RC(D))	1,29	1,35	2,64
	ATC(C(D))	1,33	1,30	2,64
	GAT(R(D))	1,35	1,29	2,64
TAA	TAA(D)	2,08	2,49	4,57
	TTA(RC(D))	2,07	2,47	4,54
	ATT(C(D))	2,49	2,08	4,57
	AAT(R(D))	2,47	2,07	4,54
AAC	AAC(D)	1,49	1,92	3,41
	GTT(RC(D))	1,47	1,89	3,36
	TTG(C(D))	1,92	1,49	3,41
	CAA(R(D))	1,89	1,47	3,36
AAG	AAG(D)	2,00	2,00	4,00
	CTT(RC(D))	2,03	1,99	4,03
	TTC(C(D))	2,00	2,00	4,00
	GAA(R(D))	1,99	2,03	4,03
ACA	ACA(D)	1,00	1,02	2,02
	TGT(RC(D))	1,02	1,00	2,02
	TGT(C(D))	1,02	1,00	2,02
	ACA(R(D))	1,00	1,02	2,02
ATA	ATA(D)	1,03	1,03	2,05
	TAT(RC(D))	1,03	1,03	2,05
	TAT(C(D))	1,03	1,03	2,05
	ATA(R(D))	1,03	1,03	2,05
AGA	AGA(D)	1,12	1,12	2,24
	TCT(RC(D))	1,12	1,12	2,24
	TCT(C(D))	1,12	1,12	2,24
	AGA(R(D))	1,12	1,12	2,24
AAA	AAA(D)	1,93	1,95	3,88
	TTT(RC(D))	1,95	1,93	3,88
	TTT(C(D))	1,95	1,93	3,88
	AAA(R(D))	1,93	1,95	3,88

Composition of DNA sequence in assembled chromosome 12

Base	No.of bases	%
A	39370109	29,54%
C	27092804	20,33%
G	27182678	20,40%
T	39492225	29,63%
N	137493	0,10%
Sum	133275309	100,00%

% of coding bases from complete assembled chromosome 1 (including N bases)

**7,54%**

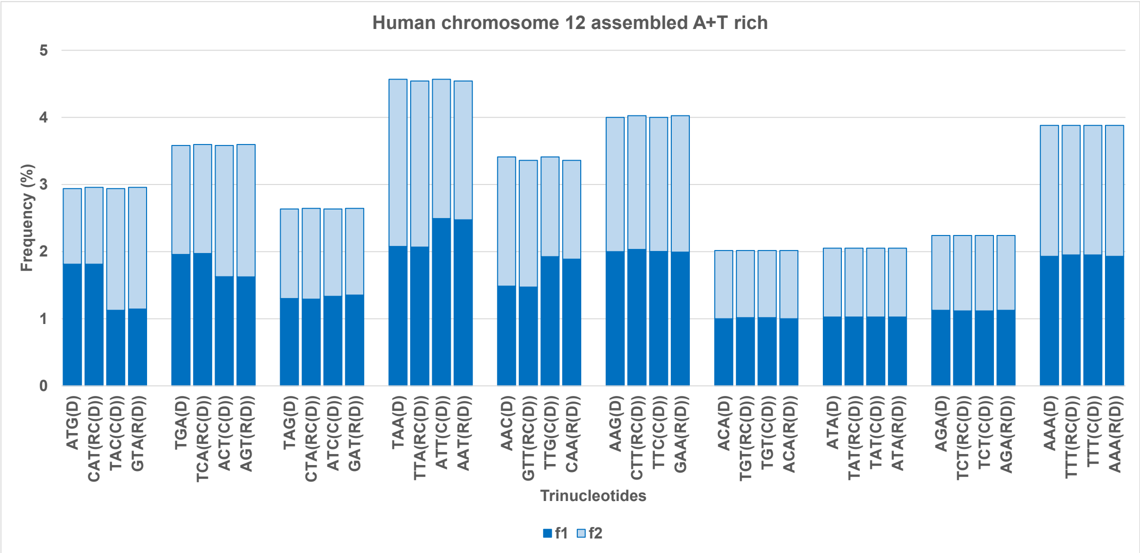

Human random chromomome 12

A-T rich

D	f	%	RC	f	%	C	f	%	R	f	%	
**ATG**	3621,00	1,81	CAT	3624,00	1,81	TAC	2284,00	1,14	GTA	2323,00	1,16	Ia
**TGA**	3918,00	1,96	TCA	4032,00	2,02	ACT	3299,00	1,65	AGT	3320,00	1,66	
**TAG**	2717,00	1,36	CTA	2609,00	1,31	ATC	2622,00	1,31	GAT	2703,00	1,35	
**TAA**	4300,00	2,15	TTA	4203,00	2,10	ATT	5132,00	2,57	AAT	5214,00	2,61	Ib
**AAC**	2990,00	1,50	GTT	3078,00	1,54	TTG	3965,00	1,98	CAA	3873,00	1,94	
**AAG**	4038,00	2,02	CTT	4153,00	2,08	TTC	4195,00	2,10	GAA	4069,00	2,04	
**ACA**	4052,00	2,03	TGT	4174,00	2,09	TGT*			ACA*			Ic
**ATA**	4469,00	2,24	TAT	4302,00	2,15	TAT*			ATA*			
**AGA**	4420,00	2,21	TCT	4495,00	2,25	TCT*			AGA*			
**AAA**	7721,00	3,86	TTT	8283,00	4,15	TTT*			AAA*			

Quadruplet		f1	f2	fu
ATG	ATG(D)	1,81	1,14	2,96
	CAT(RC(D))	1,81	1,16	2,98
	TAC(C(D))	1,14	1,81	2,96
	GTA(R(D))	1,16	1,81	2,98
TGA	TGA(D)	1,96	1,65	3,61
	TCA(RC(D))	2,02	1,66	3,68
	ACT(C(D))	1,65	1,96	3,61
	AGT(R(D))	1,66	2,02	3,68
TAG	TAG(D)	1,36	1,31	2,67
	CTA(RC(D))	1,31	1,35	2,66
	ATC(C(D))	1,31	1,36	2,67
	GAT(R(D))	1,35	1,31	2,66
TAA	TAA(D)	2,15	2,57	4,72
	TTA(RC(D))	2,10	2,61	4,71
	ATT(C(D))	2,57	2,15	4,72
	AAT(R(D))	2,61	2,10	4,71
AAC	AAC(D)	1,50	1,98	3,48
	GTT(RC(D))	1,54	1,94	3,48
	TTG(C(D))	1,98	1,50	3,48
	CAA(R(D))	1,94	1,54	3,48
AAG	AAG(D)	2,02	2,10	4,12
	CTT(RC(D))	2,08	2,04	4,12
	TTC(C(D))	2,10	2,02	4,12
	GAA(R(D))	2,04	2,08	4,12
ACA	ACA(D)	1,01	1,04	2,06
	TGT(RC(D))	1,04	1,01	2,06
	TGT(C(D))	1,04	1,01	2,06
	ACA(R(D))	1,01	1,04	2,06
ATA	ATA(D)	1,12	1,08	2,19
	TAT(RC(D))	1,08	1,12	2,19
	TAT(C(D))	1,08	1,12	2,19
	ATA(R(D))	1,12	1,12	2,24
AGA	AGA(D)	1,11	1,12	2,23
	TCT(RC(D))	1,12	1,11	2,23
	TCT(C(D))	1,12	1,11	2,23
	AGA(R(D))	1,11	1,12	2,23
AAA	AAA(D)	1,93	2,07	4,01
	TTT(RC(D))	2,07	1,93	4,01
	TTT(C(D))	2,07	1,93	4,01
	AAA(R(D))	1,93	2,07	4,01

Composition of DNA sequence in random chromosome 12

Base	No.of bases	%
A	58914	29,46%
C	40349	20,17%
G	41342	20,67%
T	58795	29,40%
N	600	0,30%
Sum	200000	100,00%

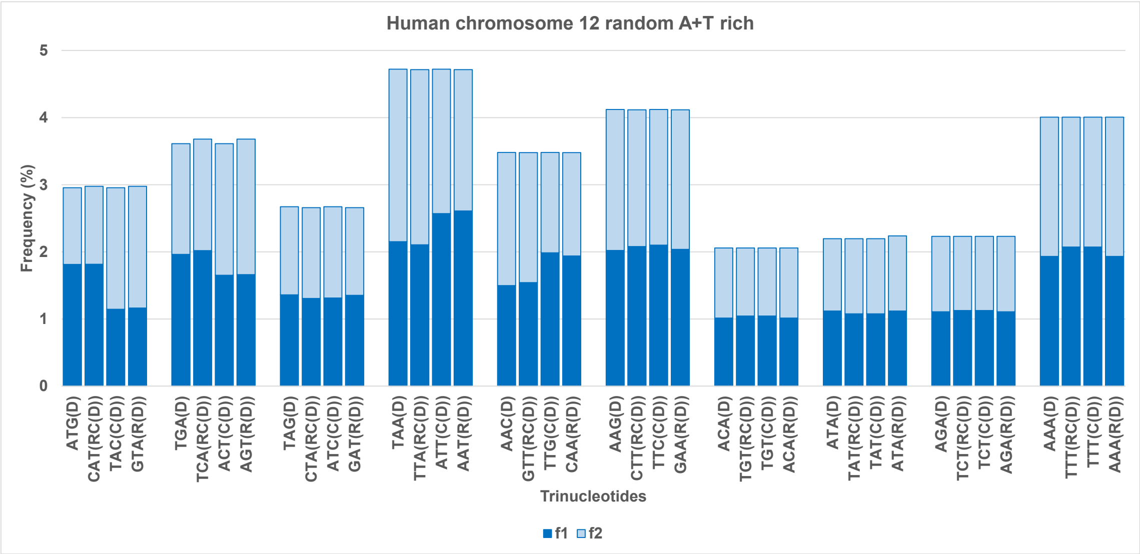

Human coding chromomome 12

A-T rich

D	f	%	RC	f	%	C	f	%	R	f	%	
**ATG**	180366,00	1,80	CAT	157142,00	1,56	TAC	98737,00	0,98	GTA	86805,00	0,86	Ia
**TGA**	198510,00	1,98	TCA	179600,00	1,79	ACT	145006,00	1,44	AGT	147242,00	1,47	
**TAG**	82971,00	0,83	CTA	101315,00	1,01	ATC	128721,00	1,28	GAT	138981,00	1,38	
**TAA**	123831,00	1,23	TTA	129087,00	1,29	ATT	167639,00	1,67	AAT	168935,00	1,68	Ib
**AAC**	138351,00	1,38	GTT	123330,00	1,23	TTG	168257,00	1,68	CAA	179635,00	1,79	
**AAG**	219739,00	2,19	CTT	180588,00	1,80	TTC	174212,00	1,73	GAA	223559,00	2,23	
**ACA**	174253,00	1,73	TGT	179451,00	1,79	TGT*			ACA*			Ic
**ATA**	116370,00	1,16	TAT	128038,00	1,27	TAT*			ATA*			
**AGA**	234863,00	2,34	TCT	187836,00	1,87	TCT*			AGA*			
**AAA**	268209,00	2,67	TTT	248907,00	2,48	TTT*			AAA*			

Quadruplet		f1	f2	fu
ATG	ATG(D)	1,80	0,98	2,78
	CAT(RC(D))	1,56	0,86	2,43
	TAC(C(D))	0,98	1,80	2,78
	GTA(R(D))	0,86	1,56	2,43
TGA	TGA(D)	1,98	1,44	3,42
	TCA(RC(D))	1,79	1,47	3,25
	ACT(C(D))	1,44	1,98	3,42
	AGT(R(D))	1,47	1,79	3,25
TAG	TAG(D)	0,83	1,28	2,11
	CTA(RC(D))	1,01	1,38	2,39
	ATC(C(D))	1,28	0,83	2,11
	GAT(R(D))	1,38	1,01	2,39
TAA	TAA(D)	1,23	1,67	2,90
	TTA(RC(D))	1,29	1,68	2,97
	ATT(C(D))	1,67	1,23	2,90
	AAT(R(D))	1,68	1,29	2,97
AAC	AAC(D)	1,38	1,68	3,05
	GTT(RC(D))	1,23	1,79	3,02
	TTG(C(D))	1,68	1,38	3,05
	CAA(R(D))	1,79	1,23	3,02
AAG	AAG(D)	2,19	1,73	3,92
	CTT(RC(D))	1,80	2,23	4,02
	TTC(C(D))	1,73	2,19	3,92
	GAA(R(D))	2,23	1,80	4,02
ACA	ACA(D)	0,87	0,89	1,76
	TGT(RC(D))	0,89	0,87	1,76
	TGT(C(D))	0,89	0,87	1,76
	ACA(R(D))	0,87	0,89	1,76
ATA	ATA(D)	0,58	0,64	1,22
	TAT(RC(D))	0,64	0,58	1,22
	TAT(C(D))	0,64	0,58	1,22
	ATA(R(D))	0,58	0,58	1,16
AGA	AGA(D)	1,17	0,94	2,10
	TCT(RC(D))	0,94	1,17	2,10
	TCT(C(D))	0,94	1,17	2,10
	AGA(R(D))	1,17	0,94	2,10
AAA	AAA(D)	1,34	1,24	2,57
	TTT(RC(D))	1,24	1,34	2,57
	TTT(C(D))	1,24	1,34	2,57
	AAA(R(D))	1,34	1,24	2,57

Composition of DNA sequence in coding chromosome 12

Base	No.of bases	%
A	2667221	26,56%
C	2399584	23,89%
G	2455517	24,45%
T	2521294	25,10%
N	0	0,00%
Sum	10043616	100,00%

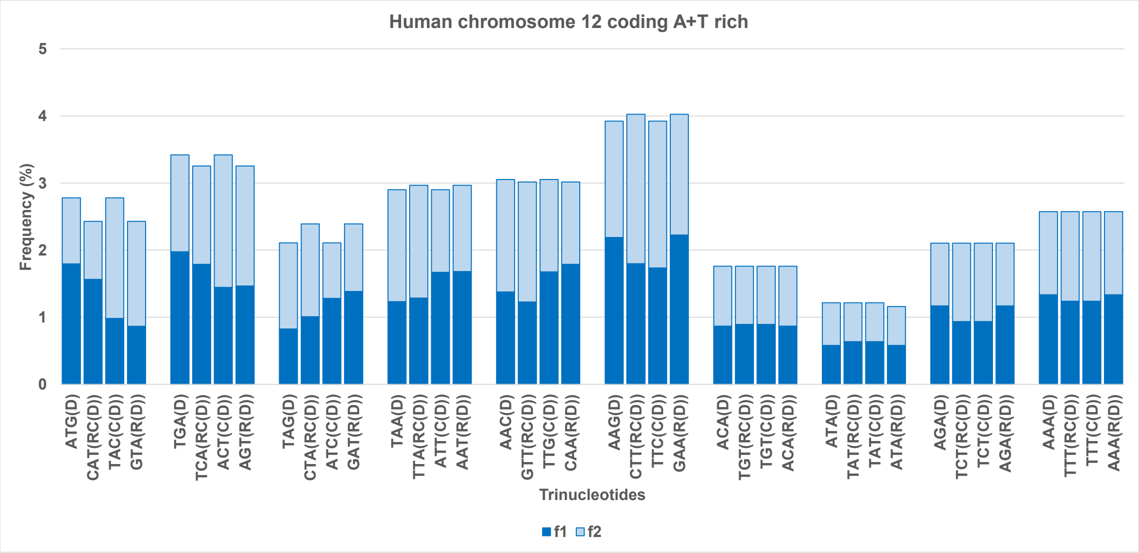

						C+G rich					
**D**	**f**	**%**	**RC**	**f**	**%**	**C**	**f**	**%**	**R**	**f**	**%**
**CGT**	341420,00	0,26	ACG	335869,00	0,25	GCA	1900855,00	1,43	TGC	1891233,00	1,42
**GTC**	1237591,00	0,93	GAC	1246525,00	0,94	CAG	2689903,00	2,02	CTG	2653980,00	1,99
**GCT**	1837967,00	1,38	AGC	1848830,00	1,39	CGA	292037,00	0,22	TCG	306200,00	0,23
**GCC**	1555580,00	1,17	GGC	1552973,00	1,17	CGG	364426,00	0,27	CCG	357182,00	0,27
**CCA**	2412782,00	1,81	TGG	2444380,00	1,84	GGT	1526938,00	1,15	ACC	1546974,00	1,16
**CCT**	2364148,00	1,78	AGG	2348322,00	1,76	GGA	2077218,00	1,56	TCC	2031557,00	1,53
**CAC**	1983914,00	1,49	GTG	2017407,00	1,52	GTG*			CAC*		
**CGC**	318085,00	0,24	GCG	316717,00	0,24	GCG*			CGC*		
**CTC**	2255859,00	1,69	GAG	2270380,00	1,71	GAG*			CTC*		
**CCC**	1703582,00	1,28	GGG	1706910,00	1,28	GGG*			CCC*		

Quadruplet		f1	f2	fu
CGT	CGT(D)	0,26	1,43	1,68
	ACG(RC(D))	0,25	1,42	1,67
	GCA(C(D))	1,43	0,26	1,68
	TGC(R(D))	1,42	0,25	1,67
GTC	GTC(D)	0,93	2,02	2,95
	GAC(RC(D))	0,94	1,99	2,93
	CAG(C(D))	2,02	0,93	2,95
	CTG(R(D))	1,99	0,94	2,93
GCT	GCT(D)	1,38	0,22	1,60
	AGC(RC(D))	1,39	0,23	1,62
	CGA(C(D))	0,22	1,38	1,60
	TCG(R(D))	0,23	1,39	1,62
GCC	GCC(D)	1,17	0,27	1,44
	GGC(RC(D))	1,17	0,27	1,43
	CGG(C(D))	0,27	1,17	1,44
	CCG(R(D))	0,27	1,17	1,43
CCA	CCA(D)	1,81	1,15	2,96
	TGG(RC(D))	1,84	1,16	3,00
	GGT(C(D))	1,15	1,81	2,96
	ACC(R(D))	1,16	1,84	3,00
CCT	CCT(D)	1,78	1,56	3,34
	AGG(RC(D))	1,76	1,53	3,29
	GGA(C(D))	1,56	1,78	3,34
	TCC(R(D))	1,53	1,76	3,29
CAC	CAC(D)	0,75	0,76	1,50
	GTG(RC(D))	0,76	0,75	1,50
	GTG(C(D))	0,76	0,75	1,50
	CAC(R(D))	0,75	0,76	1,50
CGC	CGC(D)	0,12	0,12	0,24
	GCG(RC(D))	0,12	0,12	0,24
	GCG(C(D))	0,12	0,12	0,24
	CGC(R(D))	0,12	0,12	0,24
CTC	CTC(D)	0,85	0,85	1,70
	GAG(RC(D))	0,85	0,85	1,70
	GAG(C(D))	0,85	0,85	1,70
	CTC(R(D))	0,85	0,85	1,70
CCC	CCC(D)	0,64	0,64	1,28
	GGG(RC(D))	0,64	0,64	1,28
	GGG(C(D))	0,64	0,64	1,28
	CCC(R(D))	0,64	0,64	1,28

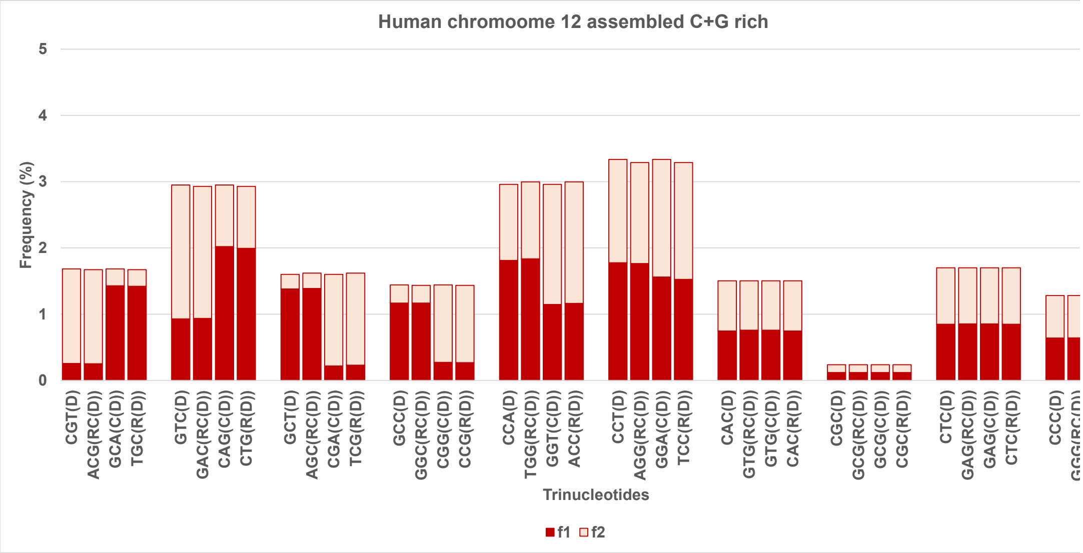

Human random chromomome 12

C+G rich											
**D**	**f**	**%**	**RC**	**f**	**%**	**C**	**f**	**%**	**R**	**f**	**%**
**CGT**	470,00	0,24	ACG	487,00	0,24	GCA	2751,00	1,38	TGC	2799,00	1,40
**GTC**	1836,00	0,92	GAC	1869,00	0,94	CAG	3877,00	1,94	CTG	3872,00	1,94
**GCT**	2754,00	1,38	AGC	2670,00	1,34	CGA	408,00	0,20	TCG	468,00	0,23
**GCC**	2120,00	1,06	GGC	2129,00	1,07	CGG	509,00	0,25	CCG	459,00	0,23
**CCA**	3345,00	1,67	TGG	3558,00	1,78	GGT	2265,00	1,13	ACC	2111,00	1,06
**CCT**	3385,00	1,69	AGG	3450,00	1,73	GGA	3123,00	1,56	TCC	2958,00	1,48
**CAC**	2806,00	1,40	GTG	2991,00	1,50	GTG*			CAC*		
**CGC**	466,00	0,23	GCG	439,00	0,22	GCG*			CGC*		
**CTC**	3300,00	1,65	GAG	3228,00	1,62	GAG*			CTC*		
**CCC**	2262,00	1,13	GGG	2433,00	1,22	GGG*			CCC*		

Quadruplet		f1	f2	fu
CGT	CGT(D)	0,24	1,38	1,61
	ACG(RC(D))	0,24	1,40	1,64
	GCA(C(D))	1,38	0,24	1,61
	TGC(R(D))	1,40	0,24	1,64
GTC	GTC(D)	0,92	1,94	2,86
	GAC(RC(D))	0,94	1,94	2,87
	CAG(C(D))	1,94	0,92	2,86
	CTG(R(D))	1,94	0,94	2,87
GCT	GCT(D)	1,38	0,20	1,58
	AGC(RC(D))	1,34	0,23	1,57
	CGA(C(D))	0,20	1,38	1,58
	TCG(R(D))	0,23	1,34	1,57
GCC	GCC(D)	1,06	0,25	1,32
	GGC(RC(D))	1,07	0,23	1,30
	CGG(C(D))	0,25	1,06	1,32
	CCG(R(D))	0,23	1,07	1,30
CCA	CCA(D)	1,67	1,13	2,81
	TGG(RC(D))	1,78	1,06	2,84
	GGT(C(D))	1,13	1,67	2,81
	ACC(R(D))	1,06	1,78	2,84
CCT	CCT(D)	1,69	1,56	3,26
	AGG(RC(D))	1,73	1,48	3,21
	GGA(C(D))	1,56	1,69	3,26
	TCC(R(D))	1,48	1,73	3,21
CAC	CAC(D)	0,70	0,75	1,45
	GTG(RC(D))	0,75	0,70	1,45
	GTG(C(D))	0,75	0,70	1,45
	CAC(R(D))	0,70	0,75	1,45
CGC	CGC(D)	0,12	0,11	0,23
	GCG(RC(D))	0,11	0,12	0,23
	GCG(C(D))	0,11	0,12	0,23
	CGC(R(D))	0,12	0,11	0,23
CTC	CTC(D)	0,83	0,81	1,63
	GAG(RC(D))	0,81	0,83	1,63
	GAG(C(D))	0,81	0,83	1,63
	CTC(R(D))	0,83	0,81	1,63
CCC	CCC(D)	0,57	0,61	1,17
	GGG(RC(D))	0,61	0,57	1,17
	GGG(C(D))	0,61	0,57	1,17
	CCC(R(D))	0,57	0,61	1,17

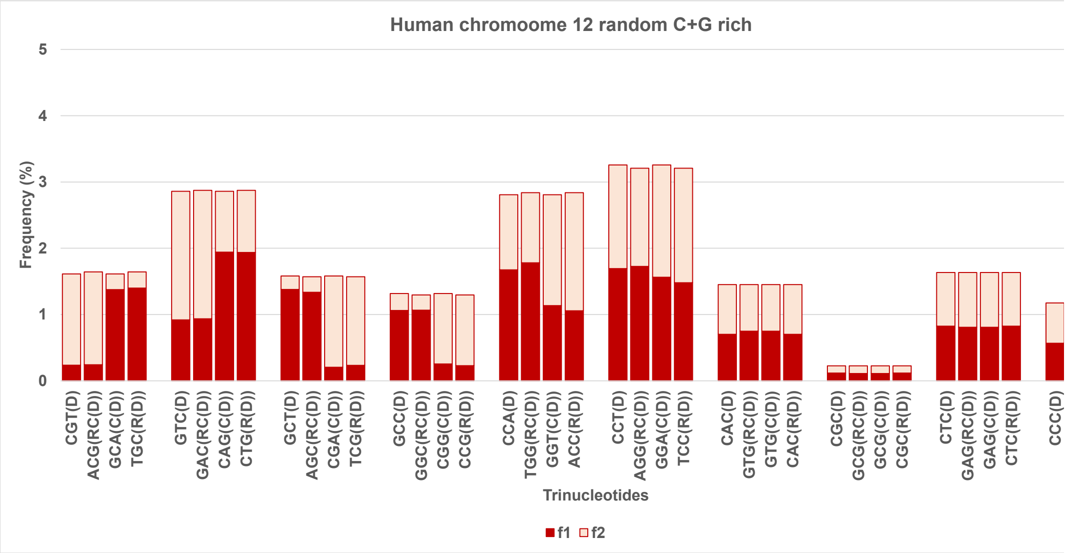

Human coding chromomome 12

C+G rich

D	f	%	RC	f	%	C	f	%	R	f	%
**CGT**	45371,00	0,45	ACG	49377,00	0,49	GCA	170670,00	1,70	TGC	173423,00	1,73
**GTC**	107613,00	1,07	GAC	128653,00	1,28	CAG	253501,00	2,52	CTG	257145,00	2,56
**GCT**	176995,00	1,76	AGC	187870,00	1,87	CGA	52599,00	0,52	TCG	47770,00	0,48
**GCC**	182189,00	1,81	GGC	169844,00	1,69	CGG	80129,00	0,80	CCG	79668,00	0,79
**CCA**	214391,00	2,13	TGG	225874,00	2,25	GGT	117174,00	1,17	ACC	145741,00	1,45
**CCT**	208659,00	2,08	AGG	194539,00	1,94	GGA	213524,00	2,13	TCC	174788,00	1,74
**CAC**	148636,00	1,48	GTG	171490,00	1,71	GTG*			CAC*		
**CGC**	65193,00	0,65	GCG	66477,00	0,66	GCG*			CGC*		
**CTC**	179448,00	1,79	GAG	208303,00	2,07	GAG*			CTC*		
**CCC**	196164,00	1,95	GGG	169910,00	1,69	GGG*			CCC*		

Quadruplet		f1	f2	fu
CGT	CGT(D)	0,45	1,70	2,15
	ACG(RC(D))	0,49	1,73	2,22
	GCA(C(D))	1,70	0,45	2,15
	TGC(R(D))	1,73	0,49	2,22
GTC	GTC(D)	1,07	2,52	3,60
	GAC(RC(D))	1,28	2,56	3,84
	CAG(C(D))	2,52	1,07	3,60
	CTG(R(D))	2,56	1,28	3,84
GCT	GCT(D)	1,76	0,52	2,29
	AGC(RC(D))	1,87	0,48	2,35
	CGA(C(D))	0,52	1,76	2,29
	TCG(R(D))	0,48	1,87	2,35
GCC	GCC(D)	1,81	0,80	2,61
	GGC(RC(D))	1,69	0,79	2,48
	CGG(C(D))	0,80	1,81	2,61
	CCG(R(D))	0,79	1,69	2,48
CCA	CCA(D)	2,13	1,17	3,30
	TGG(RC(D))	2,25	1,45	3,70
	GGT(C(D))	1,17	2,13	3,30
	ACC(R(D))	1,45	2,25	3,70
CCT	CCT(D)	2,08	2,13	4,20
	AGG(RC(D))	1,94	1,74	3,68
	GGA(C(D))	2,13	2,08	4,20
	TCC(R(D))	1,74	1,94	3,68
CAC	CAC(D)	0,74	0,85	1,59
	GTG(RC(D))	0,85	0,74	1,59
	GTG(C(D))	0,85	0,74	1,59
	CAC(R(D))	0,74	0,85	1,59
CGC	CGC(D)	0,32	0,33	0,66
	GCG(RC(D))	0,33	0,32	0,66
	GCG(C(D))	0,33	0,32	0,66
	CGC(R(D))	0,32	0,33	0,66
CTC	CTC(D)	0,89	1,04	1,93
	GAG(RC(D))	1,04	0,89	1,93
	GAG(C(D))	1,04	0,89	1,93
	CTC(R(D))	0,89	1,04	1,93
CCC	CCC(D)	0,98	0,85	1,82
	GGG(RC(D))	0,85	0,98	1,82
	GGG(C(D))	0,85	0,98	1,82
	CCC(R(D))	0,98	0,85	1,82

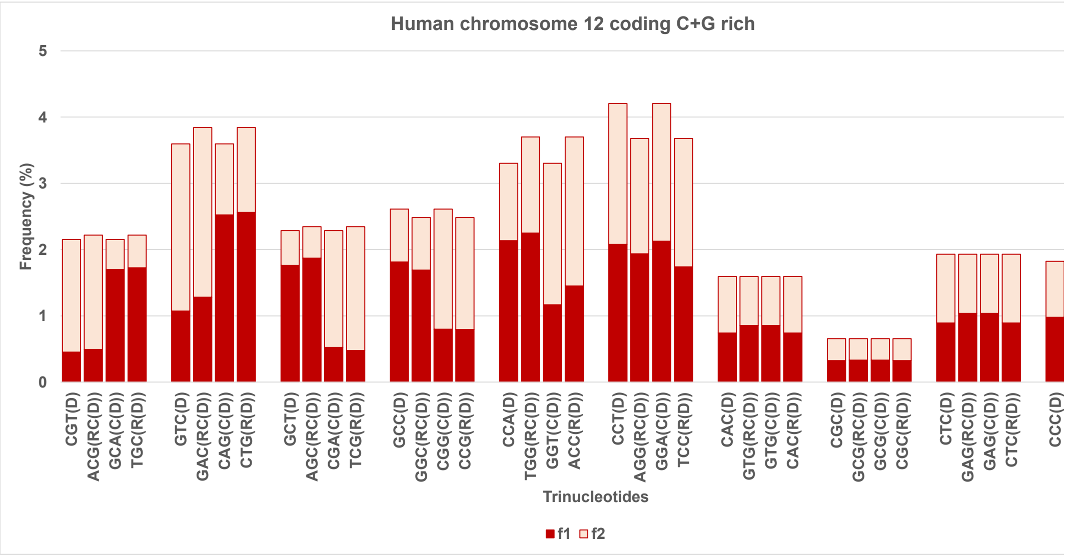

IIa

IIb

IIc

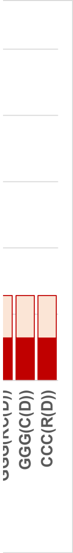

IIa

IIb

IIc

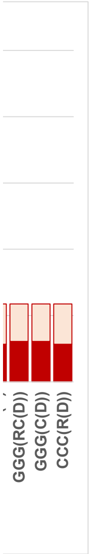

IIa

IIb

IIc

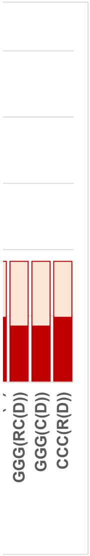
GGG(RC(D))
GGG(C(D))
CCC(R(D))

						A-T rich						
**D**	**f**	**%**	**RC**	**f**	**%**	**C**	**f**	**%**	**R**	**f**	**%**	
**ATG**	1860863,00	1,90	CAT	1877183,00	1,92	TAC	1171524,00	1,20	GTA	1181673,00	1,21	Ia
**TGA**	1961341,00	2,00	TCA	1945061,00	1,99	ACT	1604418,00	1,64	AGT	1610424,00	1,64	
**TAG**	1318399,00	1,35	CTA	1309197,00	1,34	ATC	1332697,00	1,36	GAT	1348878,00	1,38	
**TAA**	2281160,00	2,33	TTA	2275486,00	2,32	ATT	2736424,00	2,79	AAT	2708650,00	2,76	Ib
**AAC**	1497866,00	1,53	GTT	1492118,00	1,52	TTG	1944472,00	1,98	CAA	1877457,00	1,92	
**AAG**	1982506,00	2,02	CTT	2012785,00	2,05	TTC	2021368,00	2,06	GAA	2030404,00	2,07	
**ACA**	2043793,00	2,09	TGT	2055222,00	2,10	TGT*			ACA*			Ic
**ATA**	2323797,00	2,37	TAT	2319068,00	2,37	TAT*			ATA*			
**AGA**	2182819,00	2,23	TCT	2198494,00	2,24	TCT*			AGA*			
**AAA**	4063906,00	4,15	TTT	4116324,00	4,20	TTT*			AAA*			

Quadruplet		f1	f2	fu
ATG	ATG(D)	1,90	1,20	3,09
	CAT(RC(D))	1,92	1,21	3,12
	TAC(C(D))	1,20	1,90	3,09
	GTA(R(D))	1,21	1,92	3,12
TGA	TGA(D)	2,00	1,64	3,64
	TCA(RC(D))	1,99	1,64	3,63
	ACT(C(D))	1,64	2,00	3,64
	AGT(R(D))	1,64	1,99	3,63
TAG	TAG(D)	1,35	1,36	2,71
	CTA(RC(D))	1,34	1,38	2,71
	ATC(C(D))	1,36	1,35	2,71
	GAT(R(D))	1,38	1,34	2,71
TAA	TAA(D)	2,33	2,79	5,12
	TTA(RC(D))	2,32	2,76	5,09
	ATT(C(D))	2,79	2,33	5,12
	AAT(R(D))	2,76	2,32	5,09
AAC	AAC(D)	1,53	1,98	3,51
	GTT(RC(D))	1,52	1,92	3,44
	TTG(C(D))	1,98	1,53	3,51
	CAA(R(D))	1,92	1,52	3,44
AAG	AAG(D)	2,02	2,06	4,09
	CTT(RC(D))	2,05	2,07	4,13
	TTC(C(D))	2,06	2,02	4,09
	GAA(R(D))	2,07	2,05	4,13
ACA	ACA(D)	1,04	1,05	2,09
	TGT(RC(D))	1,05	1,04	2,09
	TGT(C(D))	1,05	1,04	2,09
	ACA(R(D))	1,04	1,05	2,09
ATA	ATA(D)	1,19	1,18	2,37
	TAT(RC(D))	1,18	1,19	2,37
	TAT(C(D))	1,18	1,19	2,37
	ATA(R(D))	1,19	1,19	2,37
AGA	AGA(D)	1,11	1,12	2,24
	TCT(RC(D))	1,12	1,11	2,24
	TCT(C(D))	1,12	1,11	2,24
	AGA(R(D))	1,11	1,12	2,24
AAA	AAA(D)	2,07	2,10	4,17
	TTT(RC(D))	2,10	2,07	4,17
	TTT(C(D))	2,10	2,07	4,17
	AAA(R(D))	2,07	2,10	4,17

Composition of DNA sequence in assembled chromosome 13

Base	No.of bases	%
A	30047611	26,27%
C	18839192	16,47%
G	18933605	16,56%
T	30162717	26,37%
N	16381203	14,32%
Sum	114364328	100,00%

% of coding bases from complete assembled chromosome 1 (including N bases)

2,65%

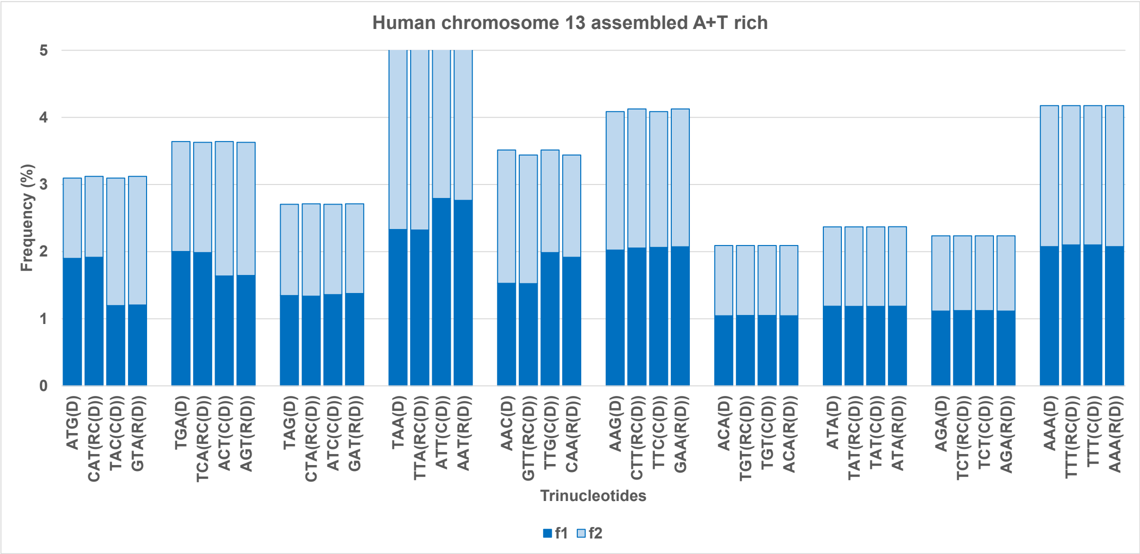

Human random chromomome 13

A-T rich

D	f	%	RC	f	%	C	f	%	R	f	%	
**ATG**	2923,00	1,86	CAT	2967,00	1,89	TAC	1867,00	1,19	GTA	1918,00	1,22	Ia
**TGA**	3164,00	2,02	TCA	3029,00	1,93	ACT	2512,00	1,60	AGT	2628,00	1,68	
**TAG**	2122,00	1,35	CTA	2124,00	1,35	ATC	2274,00	1,45	GAT	2213,00	1,41	
**TAA**	3680,00	2,35	TTA	3649,00	2,33	ATT	4368,00	2,79	AAT	4363,00	2,78	Ib
**AAC**	2447,00	1,56	GTT	2397,00	1,53	TTG	3149,00	2,01	CAA	2985,00	1,90	
**AAG**	3125,00	1,99	CTT	3125,00	1,99	TTC	3092,00	1,97	GAA	3268,00	2,08	
**ACA**	3263,00	2,08	TGT	3301,00	2,11	TGT*			ACA*			Ic
**ATA**	3704,00	2,36	TAT	3726,00	2,38	TAT*			ATA*			
**AGA**	3502,00	2,23	TCT	3528,00	2,25	TCT*			AGA*			
**AAA**	6536,00	4,17	TTT	6644,00	4,24	TTT*			AAA*			

Quadruplet		f1	f2	fu
ATG	ATG(D)	1,86	1,19	3,05
	CAT(RC(D))	1,89	1,22	3,12
	TAC(C(D))	1,19	1,86	3,05
	GTA(R(D))	1,22	1,89	3,12
TGA	TGA(D)	2,02	1,60	3,62
	TCA(RC(D))	1,93	1,68	3,61
	ACT(C(D))	1,60	2,02	3,62
	AGT(R(D))	1,68	1,93	3,61
TAG	TAG(D)	1,35	1,45	2,80
	CTA(RC(D))	1,35	1,41	2,77
	ATC(C(D))	1,45	1,35	2,80
	GAT(R(D))	1,41	1,35	2,77
TAA	TAA(D)	2,35	2,79	5,13
	TTA(RC(D))	2,33	2,78	5,11
	ATT(C(D))	2,79	2,35	5,13
	AAT(R(D))	2,78	2,33	5,11
AAC	AAC(D)	1,56	2,01	3,57
	GTT(RC(D))	1,53	1,90	3,43
	TTG(C(D))	2,01	1,56	3,57
	CAA(R(D))	1,90	1,53	3,43
AAG	AAG(D)	1,99	1,97	3,97
	CTT(RC(D))	1,99	2,08	4,08
	TTC(C(D))	1,97	1,99	3,97
	GAA(R(D))	2,08	1,99	4,08
ACA	ACA(D)	1,04	1,05	2,09
	TGT(RC(D))	1,05	1,04	2,09
	TGT(C(D))	1,05	1,04	2,09
	ACA(R(D))	1,04	1,05	2,09
ATA	ATA(D)	1,18	1,19	2,37
	TAT(RC(D))	1,19	1,18	2,37
	TAT(C(D))	1,19	1,18	2,37
	ATA(R(D))	1,18	1,18	2,36
AGA	AGA(D)	1,12	1,13	2,24
	TCT(RC(D))	1,13	1,12	2,24
	TCT(C(D))	1,13	1,12	2,24
	AGA(R(D))	1,12	1,13	2,24
AAA	AAA(D)	2,08	2,12	4,20
	TTT(RC(D))	2,12	2,08	4,20
	TTT(C(D))	2,12	2,08	4,20
	AAA(R(D))	2,08	2,12	4,20

Composition of DNA sequence in random chromosome 13

Base	No.of bases	%
A	48190	24,10%
C	30067	15,03%
G	30279	15,14%
T	48264	24,13%
N	43200	21,60%
Sum	200000	100,00%

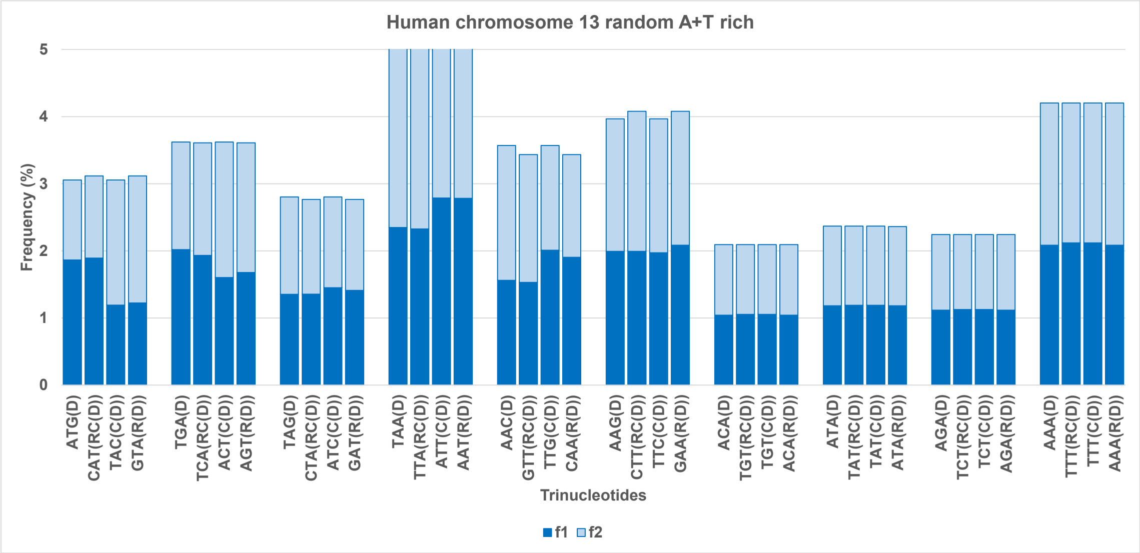

Human coding chromomome 13

A-T rich

D	f	%	RC	f	%	C	f	%	R	f	%	
**ATG**	58297,00	1,92	CAT	50504,00	1,67	TAC	32707,00	1,08	GTA	30746,00	1,01	Ia
**TGA**	62493,00	2,06	TCA	56658,00	1,87	ACT	46139,00	1,52	AGT	47513,00	1,57	
**TAG**	29758,00	0,98	CTA	31755,00	1,05	ATC	38983,00	1,29	GAT	45105,00	1,49	
**TAA**	50408,00	1,66	TTA	52499,00	1,73	ATT	65498,00	2,16	AAT	65043,00	2,15	Ib
**AAC**	45918,00	1,52	GTT	43256,00	1,43	TTG	56777,00	1,87	CAA	56733,00	1,87	
**AAG**	70650,00	2,33	CTT	56398,00	1,86	TTC	55876,00	1,84	GAA	74470,00	2,46	
**ACA**	57773,00	1,91	TGT	58811,00	1,94	TGT*			ACA*			Ic
**ATA**	47740,00	1,58	TAT	49866,00	1,65	TAT*			ATA*			
**AGA**	74335,00	2,45	TCT	55346,00	1,83	TCT*			AGA*			
**AAA**	104913,00	3,46	TTT	95257,00	3,14	TTT*			AAA*			

Quadruplet		f1	f2	fu
ATG	ATG(D)	1,92	1,08	3,00
	CAT(RC(D))	1,67	1,01	2,68
	TAC(C(D))	1,08	1,92	3,00
	GTA(R(D))	1,01	1,67	2,68
TGA	TGA(D)	2,06	1,52	3,59
	TCA(RC(D))	1,87	1,57	3,44
	ACT(C(D))	1,52	2,06	3,59
	AGT(R(D))	1,57	1,87	3,44
TAG	TAG(D)	0,98	1,29	2,27
	CTA(RC(D))	1,05	1,49	2,54
	ATC(C(D))	1,29	0,98	2,27
	GAT(R(D))	1,49	1,05	2,54
TAA	TAA(D)	1,66	2,16	3,83
	TTA(RC(D))	1,73	2,15	3,88
	ATT(C(D))	2,16	1,66	3,83
	AAT(R(D))	2,15	1,73	3,88
AAC	AAC(D)	1,52	1,87	3,39
	GTT(RC(D))	1,43	1,87	3,30
	TTG(C(D))	1,87	1,52	3,39
	CAA(R(D))	1,87	1,43	3,30
AAG	AAG(D)	2,33	1,84	4,18
	CTT(RC(D))	1,86	2,46	4,32
	TTC(C(D))	1,84	2,33	4,18
	GAA(R(D))	2,46	1,86	4,32
ACA	ACA(D)	0,95	0,97	1,92
	TGT(RC(D))	0,97	0,95	1,92
	TGT(C(D))	0,97	0,95	1,92
	ACA(R(D))	0,95	0,97	1,92
ATA	ATA(D)	0,79	0,82	1,61
	TAT(RC(D))	0,82	0,79	1,61
	TAT(C(D))	0,82	0,79	1,61
	ATA(R(D))	0,79	0,79	1,58
AGA	AGA(D)	1,23	0,91	2,14
	TCT(RC(D))	0,91	1,23	2,14
	TCT(C(D))	0,91	1,23	2,14
	AGA(R(D))	1,23	0,91	2,14
AAA	AAA(D)	1,73	1,57	3,30
	TTT(RC(D))	1,57	1,73	3,30
	TTT(C(D))	1,57	1,73	3,30
	AAA(R(D))	1,73	1,57	3,30

Composition of DNA sequence in coding chromosome 13

Base	No.of bases	%
A	876180	28,92%
C	646596	21,34%
G	685540	22,63%
T	821317	27,11%
N	0	0,00%
Sum	3029633	100,00%

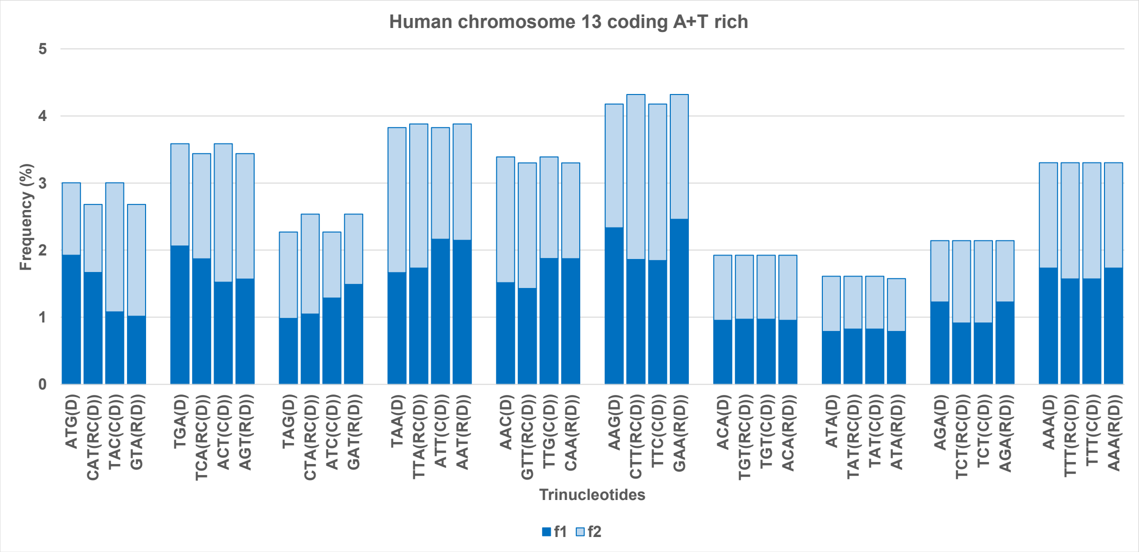

Human assembled chromomome 13

						C+G rich					
**D**	**f**	**%**	**RC**	**f**	**%**	**C**	**f**	**%**	**R**	**f**	**%**
**CGT**	237942,00	0,24	ACG	240344,00	0,25	GCA	1349307,00	1,38	TGC	1332314,00	1,36
**GTC**	871379,00	0,89	GAC	878797,00	0,90	CAG	1821032,00	1,86	CTG	1792629,00	1,83
**GCT**	1252657,00	1,28	AGC	1275626,00	1,30	CGA	191398,00	0,20	TCG	198482,00	0,20
**GCC**	967131,00	0,99	GGC	960069,00	0,98	CGG	222019,00	0,23	CCG	213618,00	0,22
**CCA**	1614253,00	1,65	TGG	1649757,00	1,68	GGT	1042254,00	1,06	ACC	1036375,00	1,06
**CCT**	1549870,00	1,58	AGG	1547087,00	1,58	GGA	1416543,00	1,45	TCC	1374236,00	1,40
**CAC**	1376743,00	1,41	GTG	1400671,00	1,43	GTG*			CAC*		
**CGC**	191110,00	0,20	GCG	190025,00	0,19	GCG*			CGC*		
**CTC**	1490829,00	1,52	GAG	1494013,00	1,52	GAG*			CTC*		
**CCC**	1061124,00	1,08	GGG	1057671,00	1,08	GGG*			CCC*		

Quadruplet		f1	f2	fu
CGT	CGT(D)	0,24	1,38	1,62
	ACG(RC(D))	0,25	1,36	1,61
	GCA(C(D))	1,38	0,24	1,62
	TGC(R(D))	1,36	0,25	1,61
GTC	GTC(D)	0,89	1,86	2,75
	GAC(RC(D))	0,90	1,83	2,73
	CAG(C(D))	1,86	0,89	2,75
	CTG(R(D))	1,83	0,90	2,73
GCT	GCT(D)	1,28	0,20	1,47
	AGC(RC(D))	1,30	0,20	1,50
	CGA(C(D))	0,20	1,28	1,47
	TCG(R(D))	0,20	1,30	1,50
GCC	GCC(D)	0,99	0,23	1,21
	GGC(RC(D))	0,98	0,22	1,20
	CGG(C(D))	0,23	0,99	1,21
	CCG(R(D))	0,22	0,98	1,20
CCA	CCA(D)	1,65	1,06	2,71
	TGG(RC(D))	1,68	1,06	2,74
	GGT(C(D))	1,06	1,65	2,71
	ACC(R(D))	1,06	1,68	2,74
CCT	CCT(D)	1,58	1,45	3,03
	AGG(RC(D))	1,58	1,40	2,98
	GGA(C(D))	1,45	1,58	3,03
	TCC(R(D))	1,40	1,58	2,98
CAC	CAC(D)	0,70	0,71	1,42
	GTG(RC(D))	0,71	0,70	1,42
	GTG(C(D))	0,71	0,70	1,42
	CAC(R(D))	0,70	0,71	1,42
CGC	CGC(D)	0,10	0,10	0,19
	GCG(RC(D))	0,10	0,10	0,19
	GCG(C(D))	0,10	0,10	0,19
	CGC(R(D))	0,10	0,10	0,19
CTC	CTC(D)	0,76	0,76	1,52
	GAG(RC(D))	0,76	0,76	1,52
	GAG(C(D))	0,76	0,76	1,52
	CTC(R(D))	0,76	0,76	1,52
CCC	CCC(D)	0,54	0,54	1,08
	GGG(RC(D))	0,54	0,54	1,08
	GGG(C(D))	0,54	0,54	1,08
	CCC(R(D))	0,54	0,54	1,08

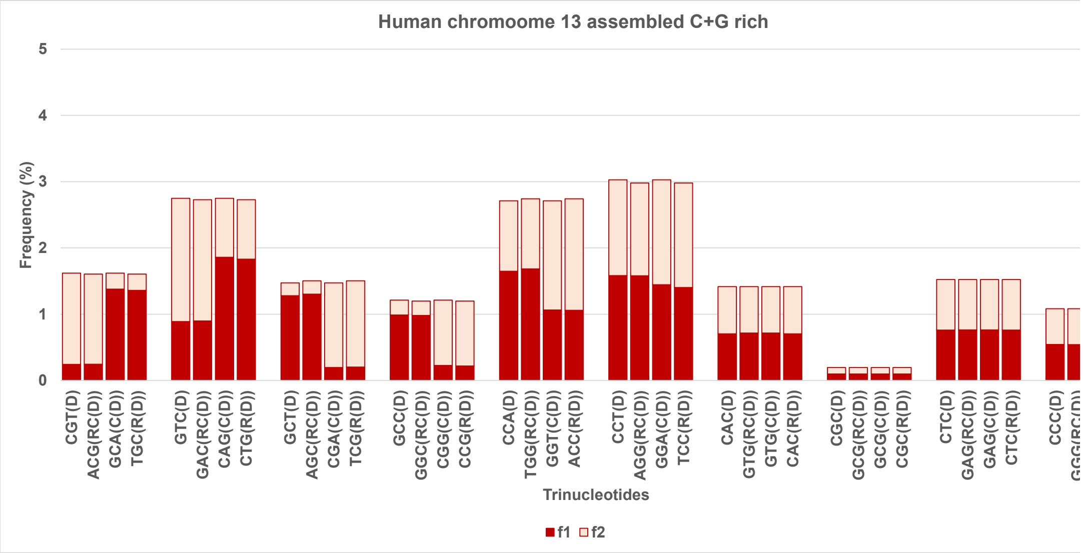

Human random chromomome 13

C+G rich											
**D**	**f**	**%**	**RC**	**f**	**%**	**C**	**f**	**%**	**R**	**f**	**%**
**CGT**	378,00	0,24	ACG	364,00	0,23	GCA	2088,00	1,33	TGC	2116,00	1,35
**GTC**	1345,00	0,86	GAC	1444,00	0,92	CAG	3005,00	1,92	CTG	2868,00	1,83
**GCT**	1949,00	1,24	AGC	2039,00	1,30	CGA	268,00	0,17	TCG	301,00	0,19
**GCC**	1574,00	1,00	GGC	1443,00	0,92	CGG	346,00	0,22	CCG	341,00	0,22
**CCA**	2660,00	1,70	TGG	2661,00	1,70	GGT	1656,00	1,06	ACC	1702,00	1,09
**CCT**	2509,00	1,60	AGG	2440,00	1,56	GGA	2348,00	1,50	TCC	2234,00	1,42
**CAC**	2083,00	1,33	GTG	2302,00	1,47	GTG*			CAC*		
**CGC**	305,00	0,19	GCG	291,00	0,19	GCG*			CGC*		
**CTC**	2381,00	1,52	GAG	2357,00	1,50	GAG*			CTC*		
**CCC**	1721,00	1,10	GGG	1684,00	1,07	GGG*			CCC*		

Quadruplet		f1	f2	fu
CGT	CGT(D)	0,24	1,33	1,57
	ACG(RC(D))	0,23	1,35	1,58
	GCA(C(D))	1,33	0,24	1,57
	TGC(R(D))	1,35	0,23	1,58
GTC	GTC(D)	0,86	1,92	2,77
	GAC(RC(D))	0,92	1,83	2,75
	CAG(C(D))	1,92	0,86	2,77
	CTG(R(D))	1,83	0,92	2,75
GCT	GCT(D)	1,24	0,17	1,41
	AGC(RC(D))	1,30	0,19	1,49
	CGA(C(D))	0,17	1,24	1,41
	TCG(R(D))	0,19	1,30	1,49
GCC	GCC(D)	1,00	0,22	1,22
	GGC(RC(D))	0,92	0,22	1,14
	CGG(C(D))	0,22	1,00	1,22
	CCG(R(D))	0,22	0,92	1,14
CCA	CCA(D)	1,70	1,06	2,75
	TGG(RC(D))	1,70	1,09	2,78
	GGT(C(D))	1,06	1,70	2,75
	ACC(R(D))	1,09	1,70	2,78
CCT	CCT(D)	1,60	1,50	3,10
	AGG(RC(D))	1,56	1,42	2,98
	GGA(C(D))	1,50	1,60	3,10
	TCC(R(D))	1,42	1,56	2,98
CAC	CAC(D)	0,66	0,73	1,40
	GTG(RC(D))	0,73	0,66	1,40
	GTG(C(D))	0,73	0,66	1,40
	CAC(R(D))	0,66	0,73	1,40
CGC	CGC(D)	0,10	0,09	0,19
	GCG(RC(D))	0,09	0,10	0,19
	GCG(C(D))	0,09	0,10	0,19
	CGC(R(D))	0,10	0,09	0,19
CTC	CTC(D)	0,76	0,75	1,51
	GAG(RC(D))	0,75	0,76	1,51
	GAG(C(D))	0,75	0,76	1,51
	CTC(R(D))	0,76	0,75	1,51
CCC	CCC(D)	0,55	0,54	1,09
	GGG(RC(D))	0,54	0,55	1,09
	GGG(C(D))	0,54	0,55	1,09
	CCC(R(D))	0,55	0,54	1,09

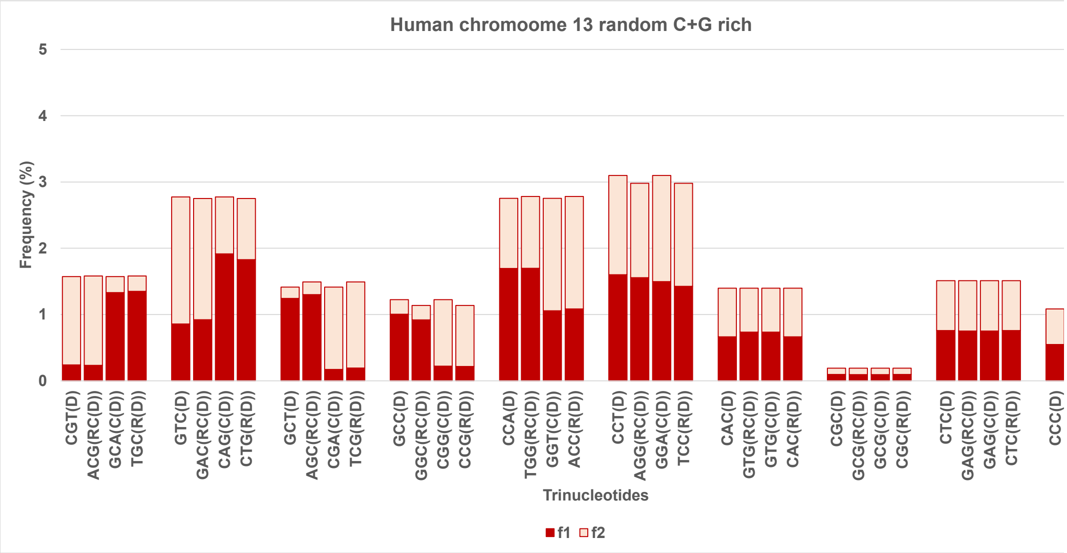

Human coding chromomome 13

C+G rich

D	f	%	RC	f	%	C	f	%	R	f	%
**CGT**	13512,00	0,45	ACG	14573,00	0,48	GCA	50030,00	1,65	TGC	48943,00	1,62
**GTC**	30287,00	1,00	GAC	35684,00	1,18	CAG	69033,00	2,28	CTG	65609,00	2,17
**GCT**	46540,00	1,54	AGC	50301,00	1,66	CGA	14458,00	0,48	TCG	13363,00	0,44
**GCC**	41863,00	1,38	GGC	40043,00	1,32	CGG	20740,00	0,68	CCG	19920,00	0,66
**CCA**	53490,00	1,77	TGG	57834,00	1,91	GGT	31851,00	1,05	ACC	37505,00	1,24
**CCT**	50678,00	1,67	AGG	50998,00	1,68	GGA	57678,00	1,90	TCC	44720,00	1,48
**CAC**	41681,00	1,38	GTG	47398,00	1,56	GTG*			CAC*		
**CGC**	17407,00	0,57	GCG	18261,00	0,60	GCG*			CGC*		
**CTC**	44941,00	1,48	GAG	53705,00	1,77	GAG*			CTC*		
**CCC**	39737,00	1,31	GGG	38623,00	1,27	GGG*			CCC*		

Quadruplet		f1	f2	fu
CGT	CGT(D)	0,45	1,65	2,10
	ACG(RC(D))	0,48	1,62	2,10
	GCA(C(D))	1,65	0,45	2,10
	TGC(R(D))	1,62	0,48	2,10
GTC	GTC(D)	1,00	2,28	3,28
	GAC(RC(D))	1,18	2,17	3,34
	CAG(C(D))	2,28	1,00	3,28
	CTG(R(D))	2,17	1,18	3,34
GCT	GCT(D)	1,54	0,48	2,01
	AGC(RC(D))	1,66	0,44	2,10
	CGA(C(D))	0,48	1,54	2,01
	TCG(R(D))	0,44	1,66	2,10
GCC	GCC(D)	1,38	0,68	2,07
	GGC(RC(D))	1,32	0,66	1,98
	CGG(C(D))	0,68	1,38	2,07
	CCG(R(D))	0,66	1,32	1,98
CCA	CCA(D)	1,77	1,05	2,82
	TGG(RC(D))	1,91	1,24	3,15
	GGT(C(D))	1,05	1,77	2,82
	ACC(R(D))	1,24	1,91	3,15
CCT	CCT(D)	1,67	1,90	3,58
	AGG(RC(D))	1,68	1,48	3,16
	GGA(C(D))	1,90	1,67	3,58
	TCC(R(D))	1,48	1,68	3,16
CAC	CAC(D)	0,69	0,78	1,47
	GTG(RC(D))	0,78	0,69	1,47
	GTG(C(D))	0,78	0,69	1,47
	CAC(R(D))	0,69	0,78	1,47
CGC	CGC(D)	0,29	0,30	0,59
	GCG(RC(D))	0,30	0,29	0,59
	GCG(C(D))	0,30	0,29	0,59
	CGC(R(D))	0,29	0,30	0,59
CTC	CTC(D)	0,74	0,89	1,63
	GAG(RC(D))	0,89	0,74	1,63
	GAG(C(D))	0,89	0,74	1,63
	CTC(R(D))	0,74	0,89	1,63
CCC	CCC(D)	0,66	0,64	1,29
	GGG(RC(D))	0,64	0,66	1,29
	GGG(C(D))	0,64	0,66	1,29
	CCC(R(D))	0,66	0,64	1,29

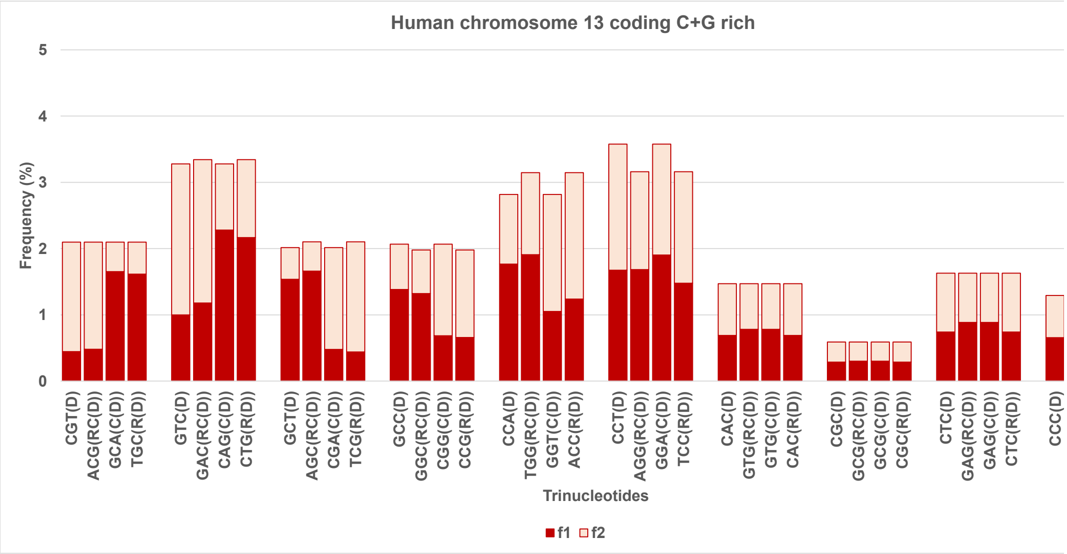

IIa

IIb

IIc

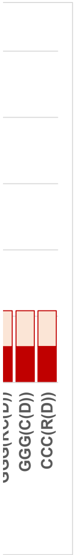

IIa

IIb

IIc

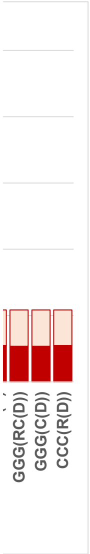

IIa

IIb

IIc

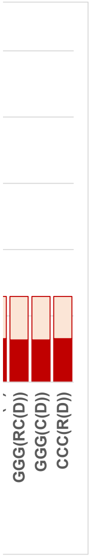
GGG(RC(D))
GGG(C(D))
CCC(R(D))

Human assembled chromomome 14

D	f	%	RC	f	%	C	f	%	R	f	%	
						A-T rich						
**ATG**	1637160,00	1,81	CAT	1651633,00	1,82	TAC	1017025,00	1,12	GTA	1036577,00	1,14	Ia
**TGA**	1785407,00	1,97	TCA	1763718,00	1,95	ACT	1459498,00	1,61	AGT	1475069,00	1,63	
**TAG**	1185405,00	1,31	CTA	1167720,00	1,29	ATC	1203608,00	1,33	GAT	1224092,00	1,35	
**TAA**	1864048,00	2,06	TTA	1868761,00	2,06	ATT	2264784,00	2,50	AAT	2223245,00	2,45	Ib
**AAC**	1331418,00	1,47	GTT	1339001,00	1,48	TTG	1766974,00	1,95	CAA	1682116,00	1,86	
**AAG**	1801098,00	1,99	CTT	1843497,00	2,04	TTC	1811545,00	2,00	GAA	1809598,00	2,00	
**ACA**	1811476,00	2,00	TGT	1839374,00	2,03	TGT*			ACA*			Ic
**ATA**	1860653,00	2,05	TAT	1867232,00	2,06	TAT*			ATA*			
**AGA**	2011925,00	2,22	TCT	2034999,00	2,25	TCT*			AGA*			
**AAA**	3453372,00	3,81	TTT	3548880,00	3,92	TTT*			AAA*			

Quadruplet		f1	f2	fu
ATG	ATG(D)	1,81	1,12	2,93
	CAT(RC(D))	1,82	1,14	2,97
	TAC(C(D))	1,12	1,81	2,93
	GTA(R(D))	1,14	1,82	2,97
TGA	TGA(D)	1,97	1,61	3,58
	TCA(RC(D))	1,95	1,63	3,58
	ACT(C(D))	1,61	1,97	3,58
	AGT(R(D))	1,63	1,95	3,58
TAG	TAG(D)	1,31	1,33	2,64
	CTA(RC(D))	1,29	1,35	2,64
	ATC(C(D))	1,33	1,31	2,64
	GAT(R(D))	1,35	1,29	2,64
TAA	TAA(D)	2,06	2,50	4,56
	TTA(RC(D))	2,06	2,45	4,52
	ATT(C(D))	2,50	2,06	4,56
	AAT(R(D))	2,45	2,06	4,52
AAC	AAC(D)	1,47	1,95	3,42
	GTT(RC(D))	1,48	1,86	3,34
	TTG(C(D))	1,95	1,47	3,42
	CAA(R(D))	1,86	1,48	3,34
AAG	AAG(D)	1,99	2,00	3,99
	CTT(RC(D))	2,04	2,00	4,03
	TTC(C(D))	2,00	1,99	3,99
	GAA(R(D))	2,00	2,04	4,03
ACA	ACA(D)	1,00	1,02	2,02
	TGT(RC(D))	1,02	1,00	2,02
	TGT(C(D))	1,02	1,00	2,02
	ACA(R(D))	1,00	1,02	2,02
ATA	ATA(D)	1,03	1,03	2,06
	TAT(RC(D))	1,03	1,03	2,06
	TAT(C(D))	1,03	1,03	2,06
	ATA(R(D))	1,03	1,03	2,05
AGA	AGA(D)	1,11	1,12	2,23
	TCT(RC(D))	1,12	1,11	2,23
	TCT(C(D))	1,12	1,11	2,23
	AGA(R(D))	1,11	1,12	2,23
AAA	AAA(D)	1,91	1,96	3,87
	TTT(RC(D))	1,96	1,91	3,87
	TTT(C(D))	1,96	1,91	3,87
	AAA(R(D))	1,91	1,96	3,87

Composition of DNA sequence in assembled chromosome 14

Base	No.of bases	%
A	26673415	24,92%
C	18423758	17,21%
G	18559033	17,34%
T	26911943	25,14%
N	16475569	15,39%
Sum	107043718	100,00%

% of coding bases from complete assembled chromosome 1 (including N bases)

**5,73%**

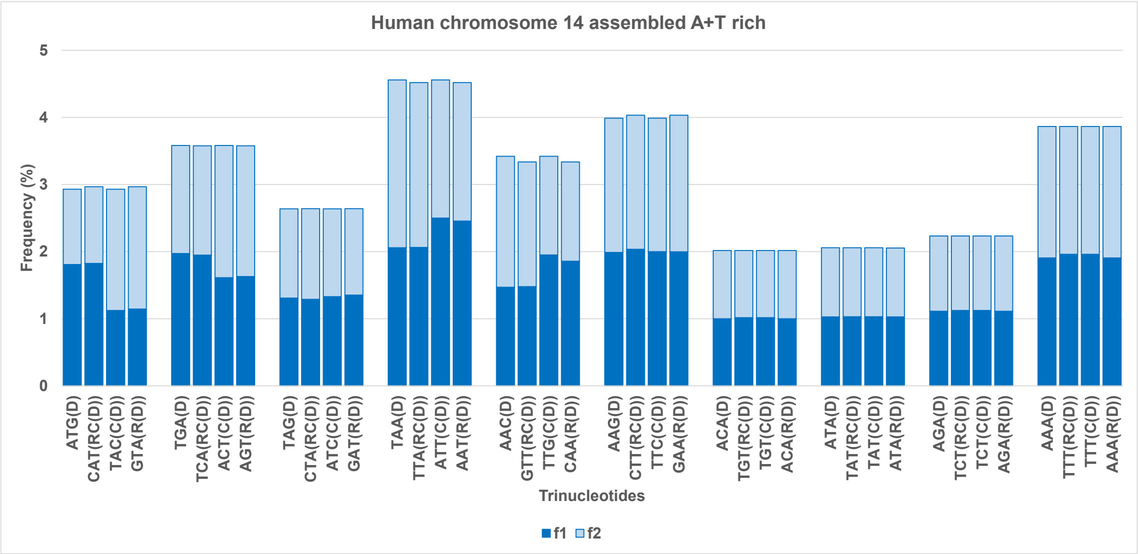

Human random chromomome 14

A-T rich

D	f	%	RC	f	%	C	f	%	R	f	%	
**ATG**	2873,00	1,83	CAT	3004,00	1,91	TAC	1876,00	1,20	GTA	1945,00	1,24	Ia
**TGA**	3094,00	1,97	TCA	3145,00	2,00	ACT	2530,00	1,61	AGT	2595,00	1,65	
**TAG**	2156,00	1,37	CTA	2122,00	1,35	ATC	2143,00	1,37	GAT	2227,00	1,42	
**TAA**	3401,00	2,17	TTA	3353,00	2,14	ATT	4222,00	2,69	AAT	4021,00	2,56	Ib
**AAC**	2417,00	1,54	GTT	2407,00	1,53	TTG	3289,00	2,10	CAA	2986,00	1,90	
**AAG**	3109,00	1,98	CTT	3214,00	2,05	TTC	3201,00	2,04	GAA	3160,00	2,01	
**ACA**	3300,00	2,10	TGT	3310,00	2,11	TGT*			ACA*			Ic
**ATA**	3489,00	2,22	TAT	3476,00	2,22	TAT*			ATA*			
**AGA**	3508,00	2,24	TCT	3576,00	2,28	TCT*			AGA*			
**AAA**	6095,00	3,88	TTT	6430,00	4,10	TTT*			AAA*			

Quadruplet		f1	f2	fu
ATG	ATG(D)	1,83	1,20	3,03
	CAT(RC(D))	1,91	1,24	3,15
	TAC(C(D))	1,20	1,83	3,03
	GTA(R(D))	1,24	1,91	3,15
TGA	TGA(D)	1,97	1,61	3,58
	TCA(RC(D))	2,00	1,65	3,66
	ACT(C(D))	1,61	1,97	3,58
	AGT(R(D))	1,65	2,00	3,66
TAG	TAG(D)	1,37	1,37	2,74
	CTA(RC(D))	1,35	1,42	2,77
	ATC(C(D))	1,37	1,37	2,74
	GAT(R(D))	1,42	1,35	2,77
TAA	TAA(D)	2,17	2,69	4,86
	TTA(RC(D))	2,14	2,56	4,70
	ATT(C(D))	2,69	2,17	4,86
	AAT(R(D))	2,56	2,14	4,70
AAC	AAC(D)	1,54	2,10	3,64
	GTT(RC(D))	1,53	1,90	3,44
	TTG(C(D))	2,10	1,54	3,64
	CAA(R(D))	1,90	1,53	3,44
AAG	AAG(D)	1,98	2,04	4,02
	CTT(RC(D))	2,05	2,01	4,06
	TTC(C(D))	2,04	1,98	4,02
	GAA(R(D))	2,01	2,05	4,06
ACA	ACA(D)	1,05	1,05	2,11
	TGT(RC(D))	1,05	1,05	2,11
	TGT(C(D))	1,05	1,05	2,11
	ACA(R(D))	1,05	1,05	2,11
ATA	ATA(D)	1,11	1,11	2,22
	TAT(RC(D))	1,11	1,11	2,22
	TAT(C(D))	1,11	1,11	2,22
	ATA(R(D))	1,11	1,11	2,22
AGA	AGA(D)	1,12	1,14	2,26
	TCT(RC(D))	1,14	1,12	2,26
	TCT(C(D))	1,14	1,12	2,26
	AGA(R(D))	1,12	1,14	2,26
AAA	AAA(D)	1,94	2,05	3,99
	TTT(RC(D))	2,05	1,94	3,99
	TTT(C(D))	2,05	1,94	3,99
	AAA(R(D))	1,94	2,05	3,99

Composition of DNA sequence in random chromosome 14

Base	No.of bases	%
A	47176	23,59%
C	30733	15,37%
G	31137	15,56%
T	47853	23,93%
N	43101	21,55%
Sum	200000	100,00%

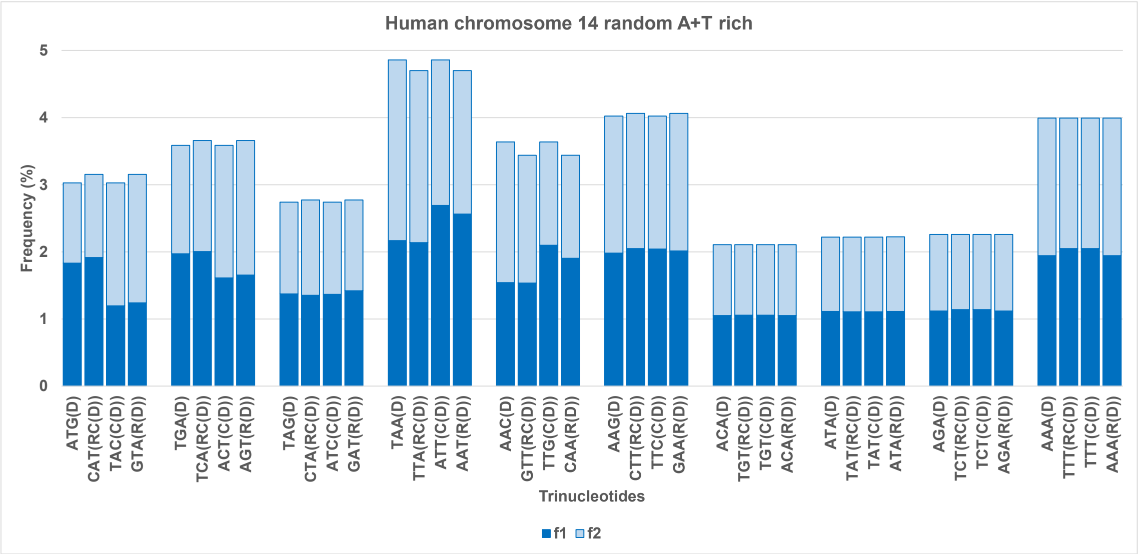

Human coding chromomome 14

A-T rich

D	f	%	RC	f	%	C	f	%	R	f	%	
**ATG**	109419,00	1,78	CAT	92799,00	1,51	TAC	60134,00	0,98	GTA	52565,00	0,86	Ia
**TGA**	121600,00	1,98	TCA	105551,00	1,72	ACT	88024,00	1,43	AGT	89411,00	1,46	
**TAG**	51032,00	0,83	CTA	61232,00	1,00	ATC	75515,00	1,23	GAT	85215,00	1,39	
**TAA**	75902,00	1,24	TTA	79407,00	1,29	ATT	99012,00	1,61	AAT	99797,00	1,63	Ib
**AAC**	83248,00	1,36	GTT	74798,00	1,22	TTG	100749,00	1,64	CAA	105709,00	1,72	
**AAG**	135488,00	2,21	CTT	109216,00	1,78	TTC	102870,00	1,68	GAA	136922,00	2,23	
**ACA**	105714,00	1,72	TGT	105953,00	1,73	TGT*			ACA*			Ic
**ATA**	69533,00	1,13	TAT	75669,00	1,23	TAT*			ATA*			
**AGA**	145956,00	2,38	TCT	110254,00	1,80	TCT*			AGA*			
**AAA**	163105,00	2,66	TTT	147861,00	2,41	TTT*			AAA*			

Quadruplet		f1	f2	fu
ATG	ATG(D)	1,78	0,98	2,76
	CAT(RC(D))	1,51	0,86	2,37
	TAC(C(D))	0,98	1,78	2,76
	GTA(R(D))	0,86	1,51	2,37
TGA	TGA(D)	1,98	1,43	3,42
	TCA(RC(D))	1,72	1,46	3,18
	ACT(C(D))	1,43	1,98	3,42
	AGT(R(D))	1,46	1,72	3,18
TAG	TAG(D)	0,83	1,23	2,06
	CTA(RC(D))	1,00	1,39	2,39
	ATC(C(D))	1,23	0,83	2,06
	GAT(R(D))	1,39	1,00	2,39
TAA	TAA(D)	1,24	1,61	2,85
	TTA(RC(D))	1,29	1,63	2,92
	ATT(C(D))	1,61	1,24	2,85
	AAT(R(D))	1,63	1,29	2,92
AAC	AAC(D)	1,36	1,64	3,00
	GTT(RC(D))	1,22	1,72	2,94
	TTG(C(D))	1,64	1,36	3,00
	CAA(R(D))	1,72	1,22	2,94
AAG	AAG(D)	2,21	1,68	3,88
	CTT(RC(D))	1,78	2,23	4,01
	TTC(C(D))	1,68	2,21	3,88
	GAA(R(D))	2,23	1,78	4,01
ACA	ACA(D)	0,86	0,86	1,72
	TGT(RC(D))	0,86	0,86	1,72
	TGT(C(D))	0,86	0,86	1,72
	ACA(R(D))	0,86	0,86	1,72
ATA	ATA(D)	0,57	0,62	1,18
	TAT(RC(D))	0,62	0,57	1,18
	TAT(C(D))	0,62	0,57	1,18
	ATA(R(D))	0,57	0,57	1,13
AGA	AGA(D)	1,19	0,90	2,09
	TCT(RC(D))	0,90	1,19	2,09
	TCT(C(D))	0,90	1,19	2,09
	AGA(R(D))	1,19	0,90	2,09
AAA	AAA(D)	1,33	1,20	2,53
	TTT(RC(D))	1,20	1,33	2,53
	TTT(C(D))	1,20	1,33	2,53
	AAA(R(D))	1,33	1,20	2,53

Composition of DNA sequence in coding chromosome 14

Base	No.of bases	%
A	1623913	26,46%
C	1470069	23,95%
G	1525882	24,86%
T	1517454	24,73%
N	0	0,00%
Sum	6137318	100,00%

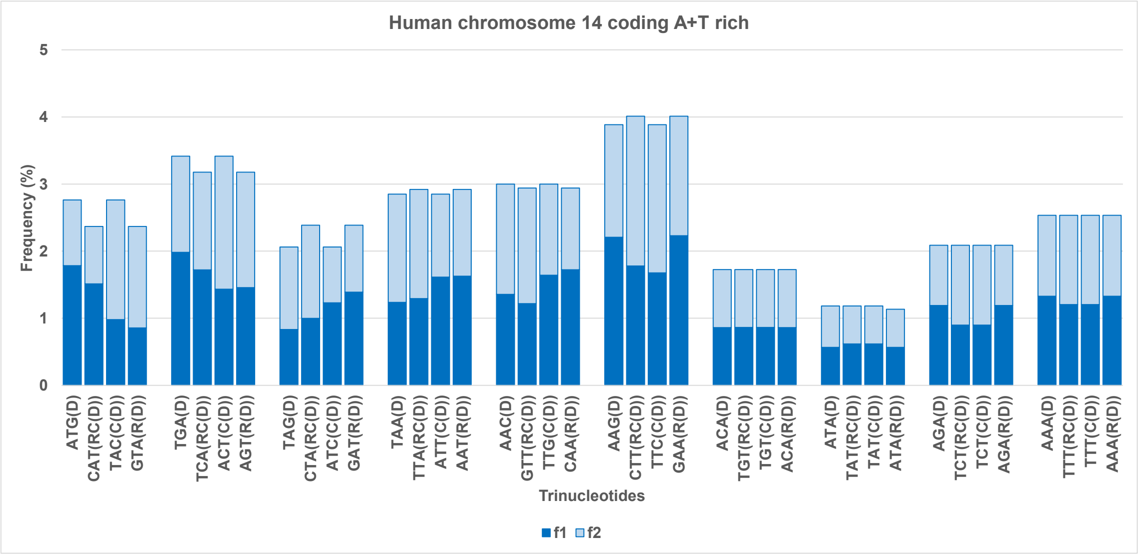

						C+G rich					
**D**	**f**	**%**	**RC**	**f**	**%**	**C**	**f**	**%**	**R**	**f**	**%**
**CGT**	233067,00	0,26	ACG	232791,00	0,26	GCA	1305089,00	1,44	TGC	1293784,00	1,43
**GTC**	847706,00	0,94	GAC	852322,00	0,94	CAG	1847242,00	2,04	CTG	1822558,00	2,01
**GCT**	1258944,00	1,39	AGC	1278944,00	1,41	CGA	196672,00	0,22	TCG	204771,00	0,23
**GCC**	1067733,00	1,18	GGC	1059454,00	1,17	CGG	251299,00	0,28	CCG	243060,00	0,27
**CCA**	1639052,00	1,81	TGG	1681561,00	1,86	GGT	1049206,00	1,16	ACC	1035345,00	1,14
**CCT**	1599414,00	1,77	AGG	1593009,00	1,76	GGA	1417211,00	1,56	TCC	1378447,00	1,52
**CAC**	1338345,00	1,48	GTG	1373432,00	1,52	GTG*			CAC*		
**CGC**	214843,00	0,24	GCG	215259,00	0,24	GCG*			CGC*		
**CTC**	1519078,00	1,68	GAG	1525195,00	1,68	GAG*			CTC*		
**CCC**	1174157,00	1,30	GGG	1178203,00	1,30	GGG*			CCC*		

Quadruplet		f1	f2	fu
CGT	CGT(D)	0,26	1,44	1,70
	ACG(RC(D))	0,26	1,43	1,69
	GCA(C(D))	1,44	0,26	1,70
	TGC(R(D))	1,43	0,26	1,69
GTC	GTC(D)	0,94	2,04	2,98
	GAC(RC(D))	0,94	2,01	2,95
	CAG(C(D))	2,04	0,94	2,98
	CTG(R(D))	2,01	0,94	2,95
GCT	GCT(D)	1,39	0,22	1,61
	AGC(RC(D))	1,41	0,23	1,64
	CGA(C(D))	0,22	1,39	1,61
	TCG(R(D))	0,23	1,41	1,64
GCC	GCC(D)	1,18	0,28	1,46
	GGC(RC(D))	1,17	0,27	1,44
	CGG(C(D))	0,28	1,18	1,46
	CCG(R(D))	0,27	1,17	1,44
CCA	CCA(D)	1,81	1,16	2,97
	TGG(RC(D))	1,86	1,14	3,00
	GGT(C(D))	1,16	1,81	2,97
	ACC(R(D))	1,14	1,86	3,00
CCT	CCT(D)	1,77	1,56	3,33
	AGG(RC(D))	1,76	1,52	3,28
	GGA(C(D))	1,56	1,77	3,33
	TCC(R(D))	1,52	1,76	3,28
CAC	CAC(D)	0,74	0,76	1,50
	GTG(RC(D))	0,76	0,74	1,50
	GTG(C(D))	0,76	0,74	1,50
	CAC(R(D))	0,74	0,76	1,50
CGC	CGC(D)	0,12	0,12	0,24
	GCG(RC(D))	0,12	0,12	0,24
	GCG(C(D))	0,12	0,12	0,24
	CGC(R(D))	0,12	0,12	0,24
CTC	CTC(D)	0,84	0,84	1,68
	GAG(RC(D))	0,84	0,84	1,68
	GAG(C(D))	0,84	0,84	1,68
	CTC(R(D))	0,84	0,84	1,68
CCC	CCC(D)	0,65	0,65	1,30
	GGG(RC(D))	0,65	0,65	1,30
	GGG(C(D))	0,65	0,65	1,30
	CCC(R(D))	0,65	0,65	1,30

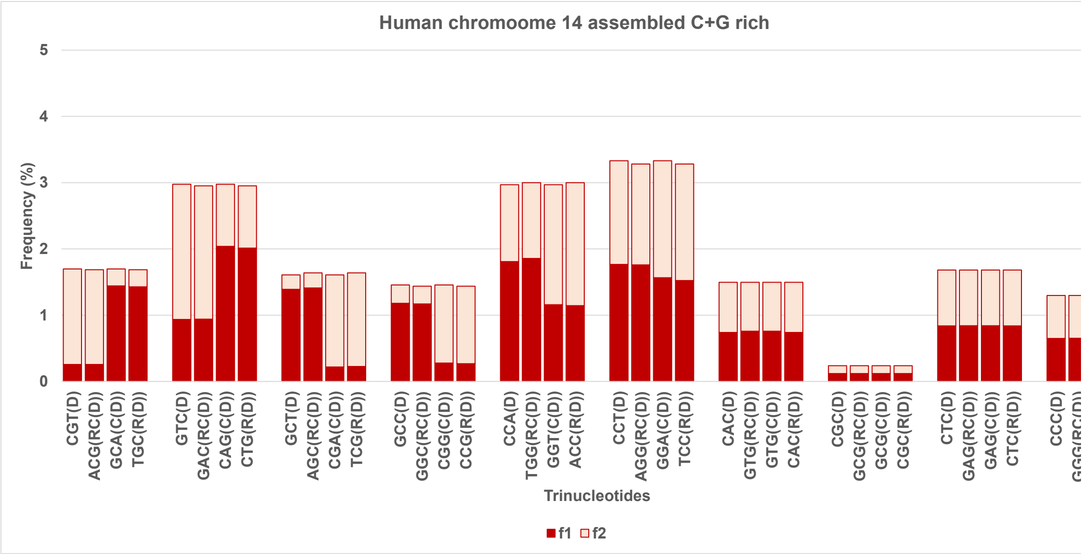

Human random chromomome 14

C+G rich

D	f	%	RC	f	%	C	f	%	R	f	%
**CGT**	383,00	0,24	ACG	389,00	0,25	GCA	2230,00	1,42	TGC	2228,00	1,42
**GTC**	1472,00	0,94	GAC	1419,00	0,90	CAG	3127,00	1,99	CTG	3002,00	1,91
**GCT**	2108,00	1,34	AGC	2158,00	1,38	CGA	328,00	0,21	TCG	325,00	0,21
**GCC**	1646,00	1,05	GGC	1589,00	1,01	CGG	338,00	0,22	CCG	344,00	0,22
**CCA**	2643,00	1,68	TGG	2765,00	1,76	GGT	1769,00	1,13	ACC	1693,00	1,08
**CCT**	2578,00	1,64	AGG	2632,00	1,68	GGA	2377,00	1,52	TCC	2225,00	1,42
**CAC**	2200,00	1,40	GTG	2233,00	1,42	GTG*			CAC*		
**CGC**	329,00	0,21	GCG	320,00	0,20	GCG*			CGC*		
**CTC**	2453,00	1,56	GAG	2501,00	1,59	GAG*			CTC*		
**CCC**	1679,00	1,07	GGG	1734,00	1,11	GGG*			CCC*		

Quadruplet		f1	f2	fu
CGT	CGT(D)	0,24	1,42	1,67
	ACG(RC(D))	0,25	1,42	1,67
	GCA(C(D))	1,42	0,24	1,67
	TGC(R(D))	1,42	0,25	1,67
GTC	GTC(D)	0,94	1,99	2,93
	GAC(RC(D))	0,90	1,91	2,82
	CAG(C(D))	1,99	0,94	2,93
	CTG(R(D))	1,91	0,90	2,82
GCT	GCT(D)	1,34	0,21	1,55
	AGC(RC(D))	1,38	0,21	1,58
	CGA(C(D))	0,21	1,34	1,55
	TCG(R(D))	0,21	1,38	1,58
GCC	GCC(D)	1,05	0,22	1,26
	GGC(RC(D))	1,01	0,22	1,23
	CGG(C(D))	0,22	1,05	1,26
	CCG(R(D))	0,22	1,01	1,23
CCA	CCA(D)	1,68	1,13	2,81
	TGG(RC(D))	1,76	1,08	2,84
	GGT(C(D))	1,13	1,68	2,81
	ACC(R(D))	1,08	1,76	2,84
CCT	CCT(D)	1,64	1,52	3,16
	AGG(RC(D))	1,68	1,42	3,10
	GGA(C(D))	1,52	1,64	3,16
	TCC(R(D))	1,42	1,68	3,10
CAC	CAC(D)	0,70	0,71	1,41
	GTG(RC(D))	0,71	0,70	1,41
	GTG(C(D))	0,71	0,70	1,41
	CAC(R(D))	0,70	0,71	1,41
CGC	CGC(D)	0,10	0,10	0,21
	GCG(RC(D))	0,10	0,10	0,21
	GCG(C(D))	0,10	0,10	0,21
	CGC(R(D))	0,10	0,10	0,21
CTC	CTC(D)	0,78	0,80	1,58
	GAG(RC(D))	0,80	0,78	1,58
	GAG(C(D))	0,80	0,78	1,58
	CTC(R(D))	0,78	0,80	1,58
CCC	CCC(D)	0,54	0,55	1,09
	GGG(RC(D))	0,55	0,54	1,09
	GGG(C(D))	0,55	0,54	1,09
	CCC(R(D))	0,54	0,55	1,09

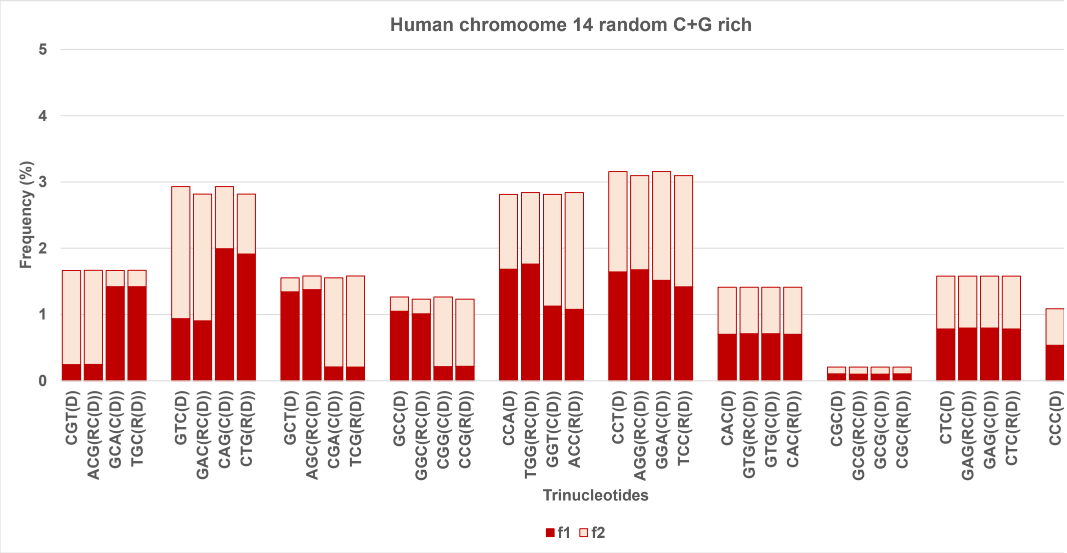

Human coding chromomome 14

C+G rich											
**D**	**f**	**%**	**RC**	**f**	**%**	**C**	**f**	**%**	**R**	**f**	**%**
**CGT**	28739,00	0,47	ACG	32350,00	0,53	GCA	106208,00	1,73	TGC	108990,00	1,78
**GTC**	64196,00	1,05	GAC	79083,00	1,29	CAG	157567,00	2,57	CTG	159587,00	2,60
**GCT**	111920,00	1,82	AGC	117914,00	1,92	CGA	34117,00	0,56	TCG	30098,00	0,49
**GCC**	116410,00	1,90	GGC	110238,00	1,80	CGG	53223,00	0,87	CCG	51027,00	0,83
**CCA**	129380,00	2,11	TGG	136397,00	2,22	GGT	70641,00	1,15	ACC	87156,00	1,42
**CCT**	128145,00	2,09	AGG	122270,00	1,99	GGA	131011,00	2,13	TCC	104986,00	1,71
**CAC**	90778,00	1,48	GTG	103185,00	1,68	GTG*			CAC*		
**CGC**	43978,00	0,72	GCG	46582,00	0,76	GCG*			CGC*		
**CTC**	108308,00	1,76	GAG	131464,00	2,14	GAG*			CTC*		
**CCC**	116264,00	1,89	GGG	105444,00	1,72	GGG*			CCC*		

Quadruplet		f1	f2	fu
CGT	CGT(D)	0,47	1,73	2,20
	ACG(RC(D))	0,53	1,78	2,30
	GCA(C(D))	1,73	0,47	2,20
	TGC(R(D))	1,78	0,53	2,30
GTC	GTC(D)	1,05	2,57	3,61
	GAC(RC(D))	1,29	2,60	3,89
	CAG(C(D))	2,57	1,05	3,61
	CTG(R(D))	2,60	1,29	3,89
GCT	GCT(D)	1,82	0,56	2,38
	AGC(RC(D))	1,92	0,49	2,41
	CGA(C(D))	0,56	1,82	2,38
	TCG(R(D))	0,49	1,92	2,41
GCC	GCC(D)	1,90	0,87	2,76
	GGC(RC(D))	1,80	0,83	2,63
	CGG(C(D))	0,87	1,90	2,76
	CCG(R(D))	0,83	1,80	2,63
CCA	CCA(D)	2,11	1,15	3,26
	TGG(RC(D))	2,22	1,42	3,64
	GGT(C(D))	1,15	2,11	3,26
	ACC(R(D))	1,42	2,22	3,64
CCT	CCT(D)	2,09	2,13	4,22
	AGG(RC(D))	1,99	1,71	3,70
	GGA(C(D))	2,13	2,09	4,22
	TCC(R(D))	1,71	1,99	3,70
CAC	CAC(D)	0,74	0,84	1,58
	GTG(RC(D))	0,84	0,74	1,58
	GTG(C(D))	0,84	0,74	1,58
	CAC(R(D))	0,74	0,84	1,58
CGC	CGC(D)	0,36	0,38	0,74
	GCG(RC(D))	0,38	0,36	0,74
	GCG(C(D))	0,38	0,36	0,74
	CGC(R(D))	0,36	0,38	0,74
CTC	CTC(D)	0,88	1,07	1,95
	GAG(RC(D))	1,07	0,88	1,95
	GAG(C(D))	1,07	0,88	1,95
	CTC(R(D))	0,88	1,07	1,95
CCC	CCC(D)	0,95	0,86	1,81
	GGG(RC(D))	0,86	0,95	1,81
	GGG(C(D))	0,86	0,95	1,81
	CCC(R(D))	0,95	0,86	1,81

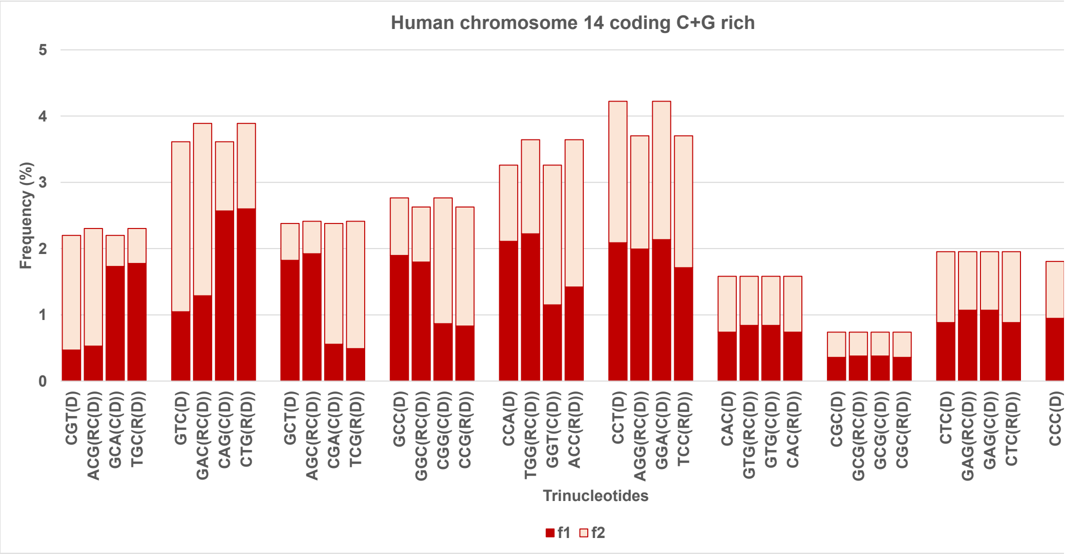

IIa

IIb

IIc

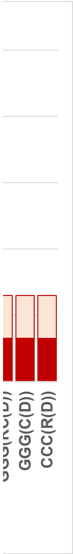

IIa

IIb

IIc

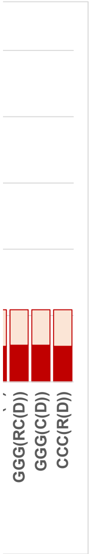

IIa

IIb

IIc

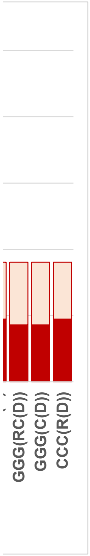
GGG(RC(D))
GGG(C(D))
CCC(R(D))

						A-T rich						
**D**	**f**	**%**	**RC**	**f**	**%**	**C**	**f**	**%**	**R**	**f**	**%**	
**ATG**	1485359,00	1,75	CAT	1519764,00	1,80	TAC	930745,00	1,10	GTA	926695,00	1,09	Ia
**TGA**	1658223,00	1,96	TCA	1645639,00	1,94	ACT	1362202,00	1,61	AGT	1354311,00	1,60	
**TAG**	1062302,00	1,26	CTA	1070275,00	1,26	ATC	1118621,00	1,32	GAT	1111794,00	1,31	
**TAA**	1611593,00	1,90	TTA	1601620,00	1,89	ATT	1969233,00	2,33	AAT	1947706,00	2,30	Ib
**AAC**	1241698,00	1,47	GTT	1217245,00	1,44	TTG	1629497,00	1,93	CAA	1561183,00	1,84	
**AAG**	1665281,00	1,97	CTT	1701471,00	2,01	TTC	1656833,00	1,96	GAA	1681907,00	1,99	
**ACA**	1695792,00	2,00	TGT	1691770,00	2,00	TGT*			ACA*			Ic
**ATA**	1593513,00	1,88	TAT	1587463,00	1,88	TAT*			ATA*			
**AGA**	1875341,00	2,22	TCT	1887849,00	2,23	TCT*			AGA*			
**AAA**	3140975,00	3,71	TTT	3171637,00	3,75	TTT*			AAA*			

Quadruplet		f1	f2	fu
ATG	ATG(D)	1,75	1,10	2,85
	CAT(RC(D))	1,80	1,09	2,89
	TAC(C(D))	1,10	1,75	2,85
	GTA(R(D))	1,09	1,80	2,89
TGA	TGA(D)	1,96	1,61	3,57
	TCA(RC(D))	1,94	1,60	3,54
	ACT(C(D))	1,61	1,96	3,57
	AGT(R(D))	1,60	1,94	3,54
TAG	TAG(D)	1,26	1,32	2,58
	CTA(RC(D))	1,26	1,31	2,58
	ATC(C(D))	1,32	1,26	2,58
	GAT(R(D))	1,31	1,26	2,58
TAA	TAA(D)	1,90	2,33	4,23
	TTA(RC(D))	1,89	2,30	4,19
	ATT(C(D))	2,33	1,90	4,23
	AAT(R(D))	2,30	1,89	4,19
AAC	AAC(D)	1,47	1,93	3,39
	GTT(RC(D))	1,44	1,84	3,28
	TTG(C(D))	1,93	1,47	3,39
	CAA(R(D))	1,84	1,44	3,28
AAG	AAG(D)	1,97	1,96	3,92
	CTT(RC(D))	2,01	1,99	4,00
	TTC(C(D))	1,96	1,97	3,92
	GAA(R(D))	1,99	2,01	4,00
ACA	ACA(D)	1,00	1,00	2,00
	TGT(RC(D))	1,00	1,00	2,00
	TGT(C(D))	1,00	1,00	2,00
	ACA(R(D))	1,00	1,00	2,00
ATA	ATA(D)	0,94	0,94	1,88
	TAT(RC(D))	0,94	0,94	1,88
	TAT(C(D))	0,94	0,94	1,88
	ATA(R(D))	0,94	0,94	1,88
AGA	AGA(D)	1,11	1,12	2,22
	TCT(RC(D))	1,12	1,11	2,22
	TCT(C(D))	1,12	1,11	2,22
	AGA(R(D))	1,11	1,12	2,22
AAA	AAA(D)	1,86	1,87	3,73
	TTT(RC(D))	1,87	1,86	3,73
	TTT(C(D))	1,87	1,86	3,73
	AAA(R(D))	1,86	1,87	3,73

Composition of DNA sequence in assembled chromosome 15

Base	No.of bases	%
A	24508669	24,03%
C	17752941	17,41%
G	17825903	17,48%
T	24553812	24,07%
N	17349864	17,01%
Sum	101991189	100,00%

% of coding bases from complete assembled chromosome 1 (including N bases)

6,21%

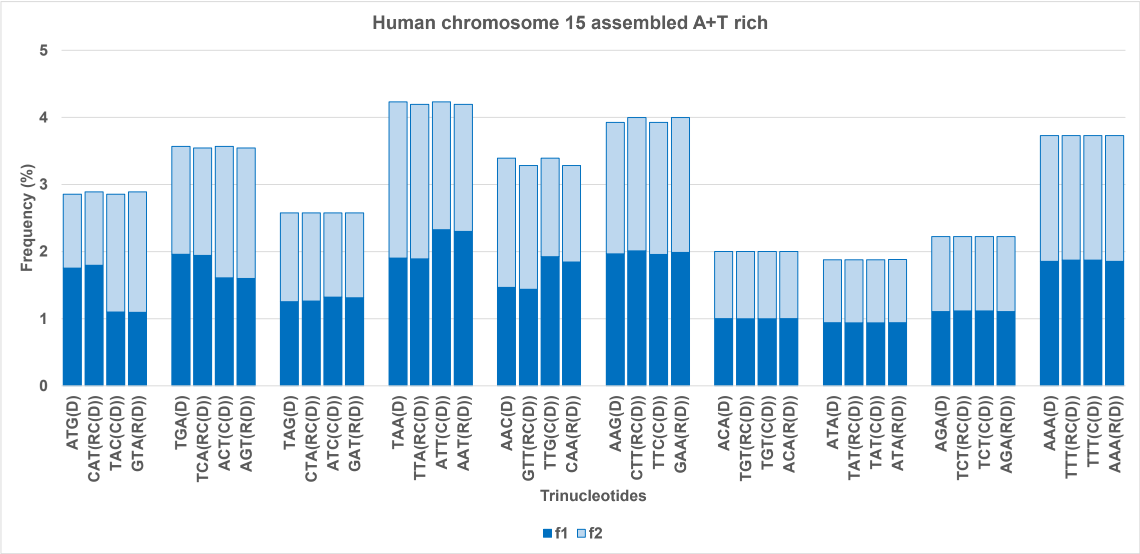

Human random chromomome 15

A-T rich

D	f	%	RC	f	%	C	f	%	R	f	%	
**ATG**	2950,00	1,53	CAT	3757,00	1,95	TAC	1972,00	1,02	GTA	1932,00	1,00	Ia
**TGA**	4382,00	2,28	TCA	3791,00	1,97	ACT	3117,00	1,62	AGT	3004,00	1,56	
**TAG**	2328,00	1,21	CTA	2443,00	1,27	ATC	2696,00	1,40	GAT	2733,00	1,42	
**TAA**	2996,00	1,56	TTA	2762,00	1,43	ATT	4978,00	2,58	AAT	4292,00	2,23	Ib
**AAC**	3534,00	1,84	GTT	3012,00	1,56	TTG	5254,00	2,73	CAA	3200,00	1,66	
**AAG**	3437,00	1,78	CTT	4421,00	2,30	TTC	4395,00	2,28	GAA	5065,00	2,63	
**ACA**	4237,00	2,20	TGT	4314,00	2,24	TGT*			ACA*			Ic
**ATA**	4003,00	2,08	TAT	3844,00	2,00	TAT*			ATA*			
**AGA**	4699,00	2,44	TCT	4788,00	2,49	TCT*			AGA*			
**AAA**	7376,00	3,83	TTT	8842,00	4,59	TTT*			AAA*			

Quadruplet		f1	f2	fu
ATG	ATG(D)	1,53	1,02	2,56
	CAT(RC(D))	1,95	1,00	2,95
	TAC(C(D))	1,02	1,53	2,56
	GTA(R(D))	1,00	1,95	2,95
TGA	TGA(D)	2,28	1,62	3,89
	TCA(RC(D))	1,97	1,56	3,53
	ACT(C(D))	1,62	2,28	3,89
	AGT(R(D))	1,56	1,97	3,53
TAG	TAG(D)	1,21	1,40	2,61
	CTA(RC(D))	1,27	1,42	2,69
	ATC(C(D))	1,40	1,21	2,61
	GAT(R(D))	1,42	1,27	2,69
TAA	TAA(D)	1,56	2,58	4,14
	TTA(RC(D))	1,43	2,23	3,66
	ATT(C(D))	2,58	1,56	4,14
	AAT(R(D))	2,23	1,43	3,66
AAC	AAC(D)	1,84	2,73	4,56
	GTT(RC(D))	1,56	1,66	3,23
	TTG(C(D))	2,73	1,84	4,56
	CAA(R(D))	1,66	1,56	3,23
AAG	AAG(D)	1,78	2,28	4,07
	CTT(RC(D))	2,30	2,63	4,93
	TTC(C(D))	2,28	1,78	4,07
	GAA(R(D))	2,63	2,30	4,93
ACA	ACA(D)	1,10	1,12	2,22
	TGT(RC(D))	1,12	1,10	2,22
	TGT(C(D))	1,12	1,10	2,22
	ACA(R(D))	1,10	1,12	2,22
ATA	ATA(D)	1,04	1,00	2,04
	TAT(RC(D))	1,00	1,04	2,04
	TAT(C(D))	1,00	1,04	2,04
	ATA(R(D))	1,04	1,04	2,08
AGA	AGA(D)	1,22	1,24	2,46
	TCT(RC(D))	1,24	1,22	2,46
	TCT(C(D))	1,24	1,22	2,46
	AGA(R(D))	1,22	1,24	2,46
AAA	AAA(D)	1,91	2,30	4,21
	TTT(RC(D))	2,30	1,91	4,21
	TTT(C(D))	2,30	1,91	4,21
	AAA(R(D))	1,91	2,30	4,21

Composition of DNA sequence in random chromosome 15

Base	No.of bases	%
A	56728	28,36%
C	37206	18,60%
G	39924	19,96%
T	58742	29,37%
N	7400	3,70%
Sum	200000	100,00%

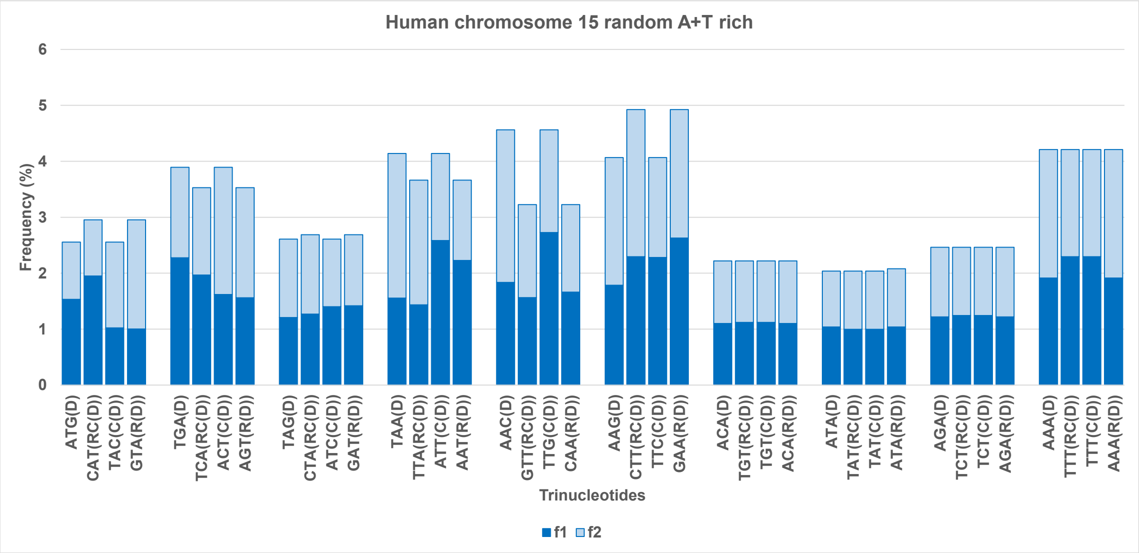

Human coding chromomome 15

A-T rich

D	f	%	RC	f	%	C	f	%	R	f	%	
**ATG**	113691,00	1,79	CAT	100653,00	1,59	TAC	62788,00	0,99	GTA	54836,00	0,87	Ia
**TGA**	125909,00	1,99	TCA	112441,00	1,77	ACT	91358,00	1,44	AGT	94902,00	1,50	
**TAG**	53167,00	0,84	CTA	63596,00	1,00	ATC	80349,00	1,27	GAT	88880,00	1,40	
**TAA**	77738,00	1,23	TTA	81519,00	1,29	ATT	104000,00	1,64	AAT	102249,00	1,61	Ib
**AAC**	87021,00	1,37	GTT	77392,00	1,22	TTG	104071,00	1,64	CAA	110995,00	1,75	
**AAG**	140055,00	2,21	CTT	112854,00	1,78	TTC	108656,00	1,71	GAA	140592,00	2,22	
**ACA**	112978,00	1,78	TGT	111875,00	1,77	TGT*			ACA*			Ic
**ATA**	73164,00	1,15	TAT	79422,00	1,25	TAT*			ATA*			
**AGA**	151378,00	2,39	TCT	116193,00	1,83	TCT*			AGA*			
**AAA**	168244,00	2,65	TTT	152310,00	2,40	TTT*			AAA*			

Quadruplet		f1	f2	fu
ATG	ATG(D)	1,79	0,99	2,78
	CAT(RC(D))	1,59	0,87	2,45
	TAC(C(D))	0,99	1,79	2,78
	GTA(R(D))	0,87	1,59	2,45
TGA	TGA(D)	1,99	1,44	3,43
	TCA(RC(D))	1,77	1,50	3,27
	ACT(C(D))	1,44	1,99	3,43
	AGT(R(D))	1,50	1,77	3,27
TAG	TAG(D)	0,84	1,27	2,11
	CTA(RC(D))	1,00	1,40	2,41
	ATC(C(D))	1,27	0,84	2,11
	GAT(R(D))	1,40	1,00	2,41
TAA	TAA(D)	1,23	1,64	2,87
	TTA(RC(D))	1,29	1,61	2,90
	ATT(C(D))	1,64	1,23	2,87
	AAT(R(D))	1,61	1,29	2,90
AAC	AAC(D)	1,37	1,64	3,02
	GTT(RC(D))	1,22	1,75	2,97
	TTG(C(D))	1,64	1,37	3,02
	CAA(R(D))	1,75	1,22	2,97
AAG	AAG(D)	2,21	1,71	3,92
	CTT(RC(D))	1,78	2,22	4,00
	TTC(C(D))	1,71	2,21	3,92
	GAA(R(D))	2,22	1,78	4,00
ACA	ACA(D)	0,89	0,88	1,77
	TGT(RC(D))	0,88	0,89	1,77
	TGT(C(D))	0,88	0,89	1,77
	ACA(R(D))	0,89	0,88	1,77
ATA	ATA(D)	0,58	0,63	1,20
	TAT(RC(D))	0,63	0,58	1,20
	TAT(C(D))	0,63	0,58	1,20
	ATA(R(D))	0,58	0,58	1,15
AGA	AGA(D)	1,19	0,92	2,11
	TCT(RC(D))	0,92	1,19	2,11
	TCT(C(D))	0,92	1,19	2,11
	AGA(R(D))	1,19	0,92	2,11
AAA	AAA(D)	1,33	1,20	2,53
	TTT(RC(D))	1,20	1,33	2,53
	TTT(C(D))	1,20	1,33	2,53
	AAA(R(D))	1,33	1,20	2,53

Composition of DNA sequence in coding chromosome 15

Base	No.of bases	%
A	1689759	26,66%
C	1509839	23,83%
G	1561131	24,63%
T	1576390	24,88%
N	0	0,00%
Sum	6337119	100,00%

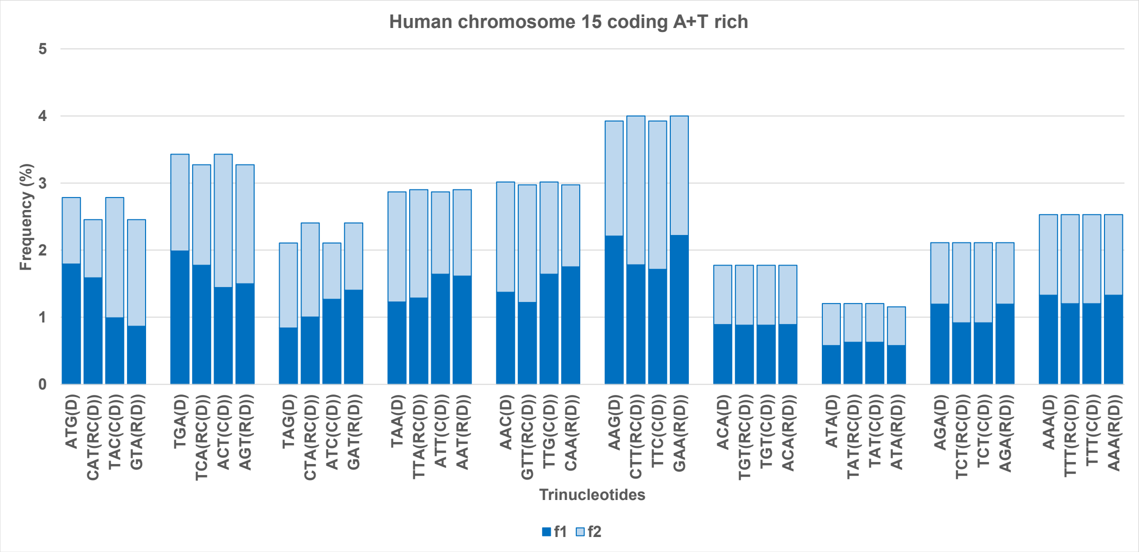

C+G rich											
**D**	**f**	**%**	**RC**	**f**	**%**	**C**	**f**	**%**	**R**	**f**	**%**
**CGT**	228040,00	0,27	ACG	227739,00	0,27	GCA	1273120,00	1,50	TGC	1243328,00	1,47
**GTC**	803878,00	0,95	GAC	813685,00	0,96	CAG	1816093,00	2,15	CTG	1785619,00	2,11
**GCT**	1221921,00	1,44	AGC	1252907,00	1,48	CGA	200191,00	0,24	TCG	205655,00	0,24
**GCC**	1077732,00	1,27	GGC	1077210,00	1,27	CGG	256262,00	0,30	CCG	250428,00	0,30
**CCA**	1592700,00	1,88	TGG	1639594,00	1,94	GGT	1006137,00	1,19	ACC	1010607,00	1,19
**CCT**	1575265,00	1,86	AGG	1567374,00	1,85	GGA	1379883,00	1,63	TCC	1330054,00	1,57
**CAC**	1310212,00	1,55	GTG	1332440,00	1,57	GTG*			CAC*		
**CGC**	221532,00	0,26	GCG	222203,00	0,26	GCG*			CGC*		
**CTC**	1489873,00	1,76	GAG	1506254,00	1,78	GAG*			CTC*		
**CCC**	1174023,00	1,39	GGG	1173793,00	1,39	GGG*			CCC*		

Quadruplet		f1	f2	fu
CGT	CGT(D)	0,27	1,50	1,77
	ACG(RC(D))	0,27	1,47	1,74
	GCA(C(D))	1,50	0,27	1,77
	TGC(R(D))	1,47	0,27	1,74
GTC	GTC(D)	0,95	2,15	3,10
	GAC(RC(D))	0,96	2,11	3,07
	CAG(C(D))	2,15	0,95	3,10
	CTG(R(D))	2,11	0,96	3,07
GCT	GCT(D)	1,44	0,24	1,68
	AGC(RC(D))	1,48	0,24	1,72
	CGA(C(D))	0,24	1,44	1,68
	TCG(R(D))	0,24	1,48	1,72
GCC	GCC(D)	1,27	0,30	1,58
	GGC(RC(D))	1,27	0,30	1,57
	CGG(C(D))	0,30	1,27	1,58
	CCG(R(D))	0,30	1,27	1,57
CCA	CCA(D)	1,88	1,19	3,07
	TGG(RC(D))	1,94	1,19	3,13
	GGT(C(D))	1,19	1,88	3,07
	ACC(R(D))	1,19	1,94	3,13
CCT	CCT(D)	1,86	1,63	3,49
	AGG(RC(D))	1,85	1,57	3,42
	GGA(C(D))	1,63	1,86	3,49
	TCC(R(D))	1,57	1,85	3,42
CAC	CAC(D)	0,77	0,79	1,56
	GTG(RC(D))	0,79	0,77	1,56
	GTG(C(D))	0,79	0,77	1,56
	CAC(R(D))	0,77	0,79	1,56
CGC	CGC(D)	0,13	0,13	0,26
	GCG(RC(D))	0,13	0,13	0,26
	GCG(C(D))	0,13	0,13	0,26
	CGC(R(D))	0,13	0,13	0,26
CTC	CTC(D)	0,88	0,89	1,77
	GAG(RC(D))	0,89	0,88	1,77
	GAG(C(D))	0,89	0,88	1,77
	CTC(R(D))	0,88	0,89	1,77
CCC	CCC(D)	0,69	0,69	1,39
	GGG(RC(D))	0,69	0,69	1,39
	GGG(C(D))	0,69	0,69	1,39
	CCC(R(D))	0,69	0,69	1,39

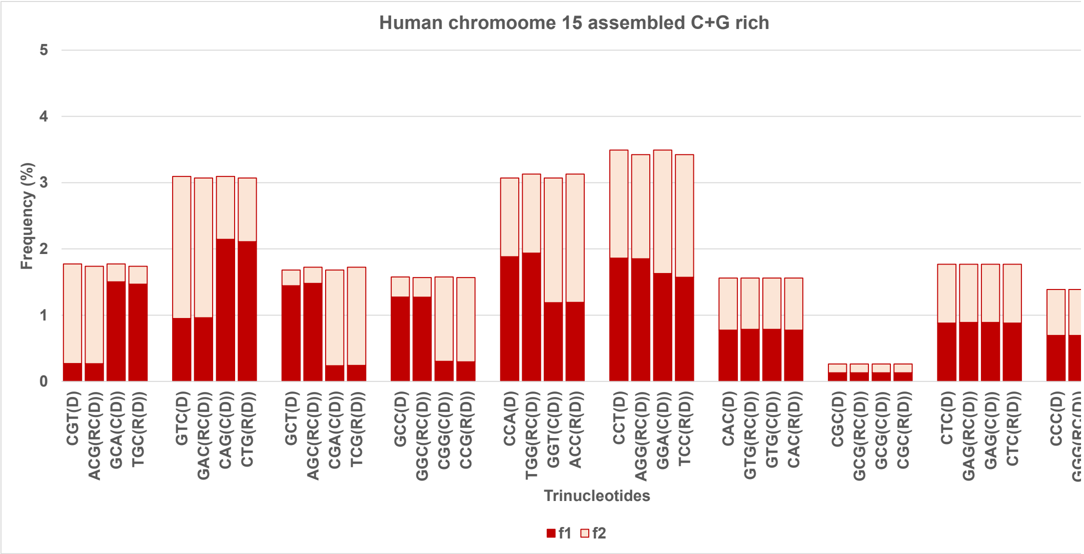

Human random chromomome 15

C+G rich											
**D**	**f**	**%**	**RC**	**f**	**%**	**C**	**f**	**%**	**R**	**f**	**%**
**CGT**	625,00	0,32	ACG	641,00	0,33	GCA	3325,00	1,73	TGC	2485,00	1,29
**GTC**	1322,00	0,69	GAC	1693,00	0,88	CAG	4135,00	2,15	CTG	3290,00	1,71
**GCT**	2186,00	1,14	AGC	2985,00	1,55	CGA	503,00	0,26	TCG	643,00	0,33
**GCC**	1861,00	0,97	GGC	1934,00	1,00	CGG	638,00	0,33	CCG	450,00	0,23
**CCA**	2486,00	1,29	TGG	3770,00	1,96	GGT	1778,00	0,92	ACC	1949,00	1,01
**CCT**	3045,00	1,58	AGG	2827,00	1,47	GGA	3522,00	1,83	TCC	2171,00	1,13
**CAC**	2746,00	1,43	GTG	3456,00	1,79	GTG*			CAC*		
**CGC**	541,00	0,28	GCG	573,00	0,30	GCG*			CGC*		
**CTC**	2981,00	1,55	GAG	3615,00	1,88	GAG*			CTC*		
**CCC**	1941,00	1,01	GGG	1915,00	0,99	GGG*			CCC*		

Quadruplet		f1	f2	fu
CGT	CGT(D)	0,32	1,73	2,05
	ACG(RC(D))	0,33	1,29	1,62
	GCA(C(D))	1,73	0,32	2,05
	TGC(R(D))	1,29	0,33	1,62
GTC	GTC(D)	0,69	2,15	2,83
	GAC(RC(D))	0,88	1,71	2,59
	CAG(C(D))	2,15	0,69	2,83
	CTG(R(D))	1,71	0,88	2,59
GCT	GCT(D)	1,14	0,26	1,40
	AGC(RC(D))	1,55	0,33	1,88
	CGA(C(D))	0,26	1,14	1,40
	TCG(R(D))	0,33	1,55	1,88
GCC	GCC(D)	0,97	0,33	1,30
	GGC(RC(D))	1,00	0,23	1,24
	CGG(C(D))	0,33	0,97	1,30
	CCG(R(D))	0,23	1,00	1,24
CCA	CCA(D)	1,29	0,92	2,21
	TGG(RC(D))	1,96	1,01	2,97
	GGT(C(D))	0,92	1,29	2,21
	ACC(R(D))	1,01	1,96	2,97
CCT	CCT(D)	1,58	1,83	3,41
	AGG(RC(D))	1,47	1,13	2,60
	GGA(C(D))	1,83	1,58	3,41
	TCC(R(D))	1,13	1,47	2,60
CAC	CAC(D)	0,71	0,90	1,61
	GTG(RC(D))	0,90	0,71	1,61
	GTG(C(D))	0,90	0,71	1,61
	CAC(R(D))	0,71	0,90	1,61
CGC	CGC(D)	0,14	0,15	0,29
	GCG(RC(D))	0,15	0,14	0,29
	GCG(C(D))	0,15	0,14	0,29
	CGC(R(D))	0,14	0,15	0,29
CTC	CTC(D)	0,77	0,94	1,71
	GAG(RC(D))	0,94	0,77	1,71
	GAG(C(D))	0,94	0,77	1,71
	CTC(R(D))	0,77	0,94	1,71
CCC	CCC(D)	0,50	0,50	1,00
	GGG(RC(D))	0,50	0,50	1,00
	GGG(C(D))	0,50	0,50	1,00
	CCC(R(D))	0,50	0,50	1,00

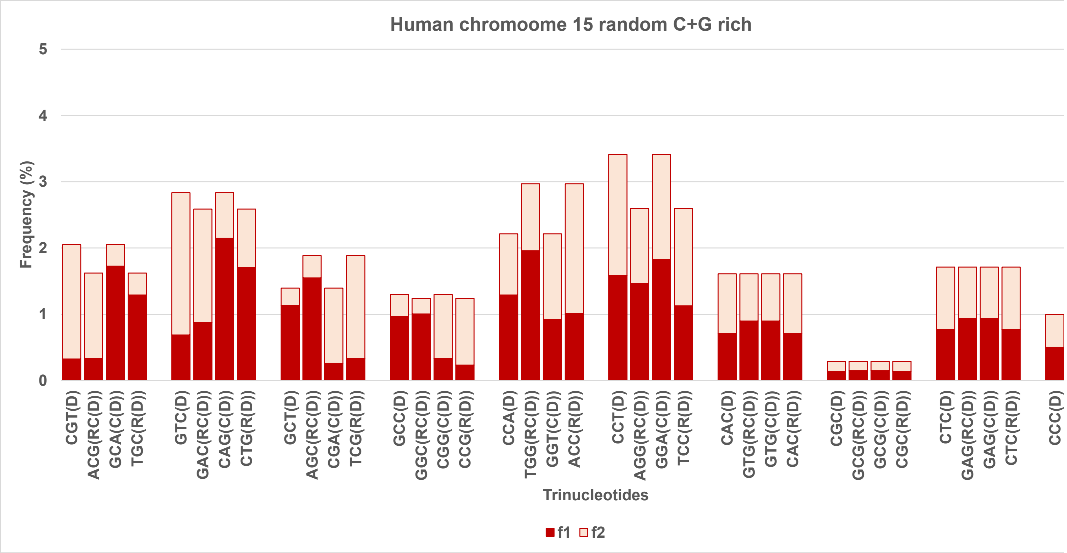

Human coding chromomome 15

C+G rich

D	f	%	RC	f	%	C	f	%	R	f	%
**CGT**	27992,00	0,44	ACG	31481,00	0,50	GCA	110971,00	1,75	TGC	111727,00	1,76
**GTC**	66258,00	1,05	GAC	81274,00	1,28	CAG	165364,00	2,61	CTG	164687,00	2,60
**GCT**	113719,00	1,79	AGC	122181,00	1,93	CGA	33812,00	0,53	TCG	29631,00	0,47
**GCC**	117577,00	1,86	GGC	109568,00	1,73	CGG	49869,00	0,79	CCG	49352,00	0,78
**CCA**	136089,00	2,15	TGG	142035,00	2,24	GGT	72813,00	1,15	ACC	90733,00	1,43
**CCT**	129777,00	2,05	AGG	125975,00	1,99	GGA	135497,00	2,14	TCC	106908,00	1,69
**CAC**	95467,00	1,51	GTG	109097,00	1,72	GTG*			CAC*		
**CGC**	43122,00	0,68	GCG	44331,00	0,70	GCG*			CGC*		
**CTC**	109910,00	1,73	GAG	135850,00	2,14	GAG*			CTC*		
**CCC**	116300,00	1,84	GGG	102474,00	1,62	GGG*			CCC*		

Quadruplet		f1	f2	fu
CGT	CGT(D)	0,44	1,75	2,19
	ACG(RC(D))	0,50	1,76	2,26
	GCA(C(D))	1,75	0,44	2,19
	TGC(R(D))	1,76	0,50	2,26
GTC	GTC(D)	1,05	2,61	3,66
	GAC(RC(D))	1,28	2,60	3,88
	CAG(C(D))	2,61	1,05	3,66
	CTG(R(D))	2,60	1,28	3,88
GCT	GCT(D)	1,79	0,53	2,33
	AGC(RC(D))	1,93	0,47	2,40
	CGA(C(D))	0,53	1,79	2,33
	TCG(R(D))	0,47	1,93	2,40
GCC	GCC(D)	1,86	0,79	2,64
	GGC(RC(D))	1,73	0,78	2,51
	CGG(C(D))	0,79	1,86	2,64
	CCG(R(D))	0,78	1,73	2,51
CCA	CCA(D)	2,15	1,15	3,30
	TGG(RC(D))	2,24	1,43	3,67
	GGT(C(D))	1,15	2,15	3,30
	ACC(R(D))	1,43	2,24	3,67
CCT	CCT(D)	2,05	2,14	4,19
	AGG(RC(D))	1,99	1,69	3,67
	GGA(C(D))	2,14	2,05	4,19
	TCC(R(D))	1,69	1,99	3,67
CAC	CAC(D)	0,75	0,86	1,61
	GTG(RC(D))	0,86	0,75	1,61
	GTG(C(D))	0,86	0,75	1,61
	CAC(R(D))	0,75	0,86	1,61
CGC	CGC(D)	0,34	0,35	0,69
	GCG(RC(D))	0,35	0,34	0,69
	GCG(C(D))	0,35	0,34	0,69
	CGC(R(D))	0,34	0,35	0,69
CTC	CTC(D)	0,87	1,07	1,94
	GAG(RC(D))	1,07	0,87	1,94
	GAG(C(D))	1,07	0,87	1,94
	CTC(R(D))	0,87	1,07	1,94
CCC	CCC(D)	0,92	0,81	1,73
	GGG(RC(D))	0,81	0,92	1,73
	GGG(C(D))	0,81	0,92	1,73
	CCC(R(D))	0,92	0,81	1,73

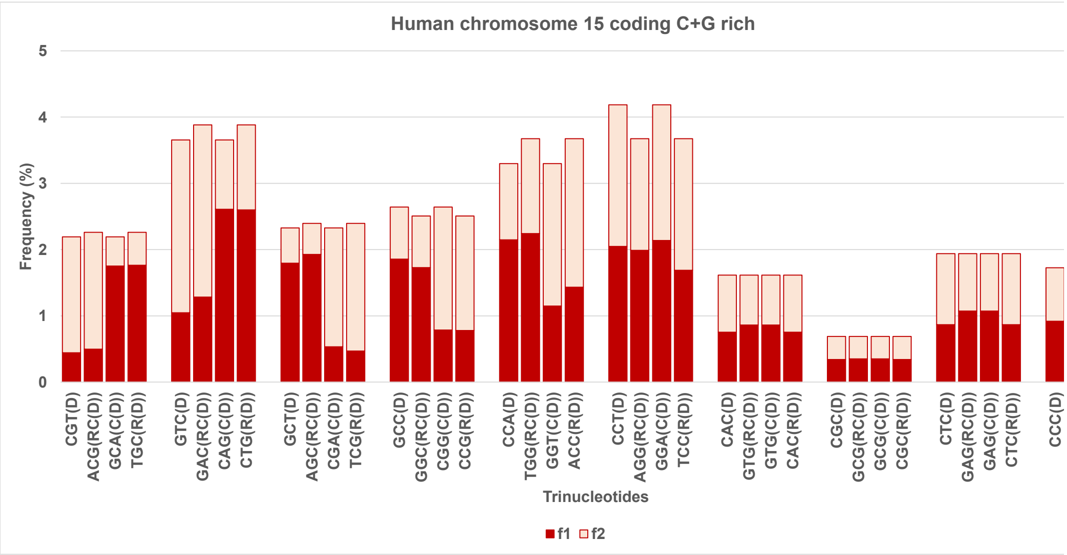

IIa

IIb

IIc

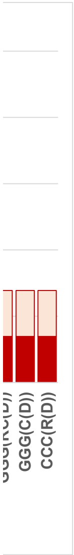

IIa

IIb

IIc

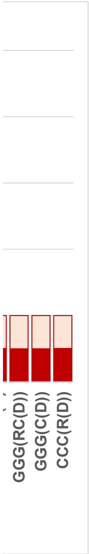

IIa

IIb

IIc

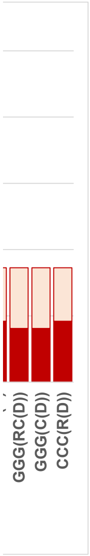
GGG(RC(D))
GGG(C(D))
CCC(R(D))

					A-T rich							
**D**	**f**	**%**	**RC**	**f**	**%**	**C**	**f**	**%**	**R**	**f**	**%**	
**ATG**	1410219,00	1,72	CAT	1403706,00	1,72	TAC	804133,00	0,98	GTA	829458,00	1,01	Ia
**TGA**	1578794,00	1,93	TCA	1587257,00	1,94	ACT	1256001,00	1,54	AGT	1265436,00	1,55	
**TAG**	941383,00	1,15	CTA	920926,00	1,13	ATC	1038380,00	1,27	GAT	1082224,00	1,32	
**TAA**	1359357,00	1,66	TTA	1360955,00	1,66	ATT	1713709,00	2,09	AAT	1669912,00	2,04	Ib
**AAC**	1123413,00	1,37	GTT	1128673,00	1,38	TTG	1493613,00	1,83	CAA	1446512,00	1,77	
**AAG**	1518942,00	1,86	CTT	1561613,00	1,91	TTC	1549427,00	1,89	GAA	1506402,00	1,84	
**ACA**	1569856,00	1,92	TGT	1611704,00	1,97	TGT*			ACA*			Ic
**ATA**	1295139,00	1,58	TAT	1301605,00	1,59	TAT*			ATA*			
**AGA**	1765152,00	2,16	TCT	1759333,00	2,15	TCT*			AGA*			
**AAA**	2721109,00	3,33	TTT	2787175,00	3,41	TTT*			AAA*			

Quadruplet		f1	f2	fu
ATG	ATG(D)	1,72	0,98	2,71
	CAT(RC(D))	1,72	1,01	2,73
	TAC(C(D))	0,98	1,72	2,71
	GTA(R(D))	1,01	1,72	2,73
TGA	TGA(D)	1,93	1,54	3,47
	TCA(RC(D))	1,94	1,55	3,49
	ACT(C(D))	1,54	1,93	3,47
	AGT(R(D))	1,55	1,94	3,49
TAG	TAG(D)	1,15	1,27	2,42
	CTA(RC(D))	1,13	1,32	2,45
	ATC(C(D))	1,27	1,15	2,42
	GAT(R(D))	1,32	1,13	2,45
TAA	TAA(D)	1,66	2,09	3,76
	TTA(RC(D))	1,66	2,04	3,70
	ATT(C(D))	2,09	1,66	3,76
	AAT(R(D))	2,04	1,66	3,70
AAC	AAC(D)	1,37	1,83	3,20
	GTT(RC(D))	1,38	1,77	3,15
	TTG(C(D))	1,83	1,37	3,20
	CAA(R(D))	1,77	1,38	3,15
AAG	AAG(D)	1,86	1,89	3,75
	CTT(RC(D))	1,91	1,84	3,75
	TTC(C(D))	1,89	1,86	3,75
	GAA(R(D))	1,84	1,91	3,75
ACA	ACA(D)	0,96	0,99	1,94
	TGT(RC(D))	0,99	0,96	1,94
	TGT(C(D))	0,99	0,96	1,94
	ACA(R(D))	0,96	0,99	1,94
ATA	ATA(D)	0,79	0,80	1,59
	TAT(RC(D))	0,80	0,79	1,59
	TAT(C(D))	0,80	0,79	1,59
	ATA(R(D))	0,79	0,79	1,58
AGA	AGA(D)	1,08	1,08	2,15
	TCT(RC(D))	1,08	1,08	2,15
	TCT(C(D))	1,08	1,08	2,15
	AGA(R(D))	1,08	1,08	2,15
AAA	AAA(D)	1,66	1,70	3,37
	TTT(RC(D))	1,70	1,66	3,37
	TTT(C(D))	1,70	1,66	3,37
	AAA(R(D))	1,66	1,70	3,37

Composition of DNA sequence in assembled chromosome 16

Base	No.of bases	%
A	22558319	24,97%
C	18172742	20,12%
G	18299976	20,26%
T	22774906	25,21%
N	8532402	9,44%
Sum	90338345	100,00%

% of coding bases from complete assembled chromosome 1 (including N bases)

8,23%

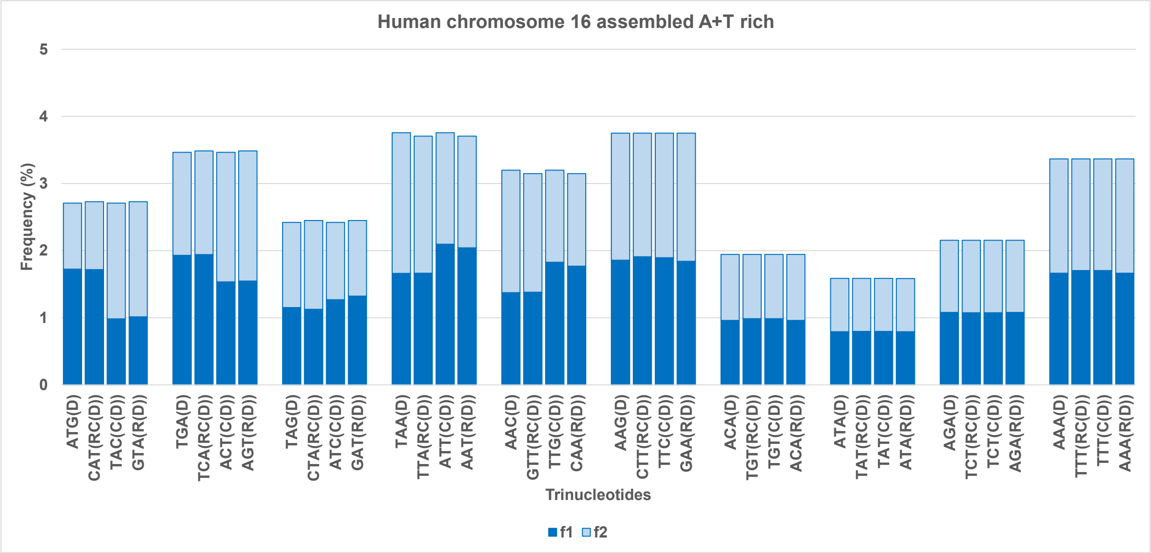

Human random chromomome 16

A-T rich

D	f	%	RC	f	%	C	f	%	R	f	%	
**ATG**	3389,00	1,70	CAT	3394,00	1,71	TAC	2060,00	1,04	GTA	2126,00	1,07	Ia
**TGA**	3883,00	1,95	TCA	3920,00	1,97	ACT	3188,00	1,60	AGT	3209,00	1,61	
**TAG**	2519,00	1,27	CTA	2401,00	1,21	ATC	2641,00	1,33	GAT	2746,00	1,38	
**TAA**	3456,00	1,74	TTA	3400,00	1,71	ATT	4100,00	2,06	AAT	4098,00	2,06	Ib
**AAC**	2666,00	1,34	GTT	2768,00	1,39	TTG	3628,00	1,82	CAA	3648,00	1,84	
**AAG**	3892,00	1,96	CTT	3732,00	1,88	TTC	3573,00	1,80	GAA	3552,00	1,79	
**ACA**	3677,00	1,85	TGT	3719,00	1,87	TGT*			ACA*			Ic
**ATA**	3209,00	1,61	TAT	3101,00	1,56	TAT*			ATA*			
**AGA**	4468,00	2,25	TCT	4221,00	2,12	TCT*			AGA*			
**AAA**	6981,00	3,51	TTT	6692,00	3,37	TTT*			AAA*			

Quadruplet		f1	f2	fu
	ATG(D)	1,70	1,04	2,74
ATG	CAT(RC(D))	1,71	1,07	2,78
	TAC(C(D))	1,04	1,70	2,74
	GTA(R(D))	1,07	1,71	2,78
	TGA(D)	1,95	1,60	3,56
TGA	TCA(RC(D))	1,97	1,61	3,59
	ACT(C(D))	1,60	1,95	3,56
	AGT(R(D))	1,61	1,97	3,59
	TAG(D)	1,27	1,33	2,60
TAG	CTA(RC(D))	1,21	1,38	2,59
	ATC(C(D))	1,33	1,27	2,60
	GAT(R(D))	1,38	1,21	2,59
	TAA(D)	1,74	2,06	3,80
TAA	TTA(RC(D))	1,71	2,06	3,77
	ATT(C(D))	2,06	1,74	3,80
	AAT(R(D))	2,06	1,71	3,77
	AAC(D)	1,34	1,82	3,17
AAC	GTT(RC(D))	1,39	1,84	3,23
	TTG(C(D))	1,82	1,34	3,17
	CAA(R(D))	1,84	1,39	3,23
	AAG(D)	1,96	1,80	3,76
AAG	CTT(RC(D))	1,88	1,79	3,66
	TTC(C(D))	1,80	1,96	3,76
	GAA(R(D))	1,79	1,88	3,66
	ACA(D)	0,92	0,94	1,86
ACA	TGT(RC(D))	0,94	0,92	1,86
	TGT(C(D))	0,94	0,92	1,86
	ACA(R(D))	0,92	0,94	1,86
	ATA(D)	0,81	0,78	1,59
ATA	TAT(RC(D))	0,78	0,81	1,59
	TAT(C(D))	0,78	0,81	1,59
	ATA(R(D))	0,81	0,81	1,61
	AGA(D)	1,12	1,06	2,19
AGA	TCT(RC(D))	1,06	1,12	2,19
	TCT(C(D))	1,06	1,12	2,19
	AGA(R(D))	1,12	1,06	2,19
	AAA(D)	1,76	1,68	3,44
AAA	TTT(RC(D))	1,68	1,76	3,44
	TTT(C(D))	1,68	1,76	3,44
	AAA(R(D))	1,76	1,68	3,44

Composition of DNA sequence in random chromosome 16

Base	No.of bases	%
A	55976	27,99%
C	43451	21,73%
G	44222	22,11%
T	55151	27,58%
N	1200	0,60%
Sum	200000	100,00%

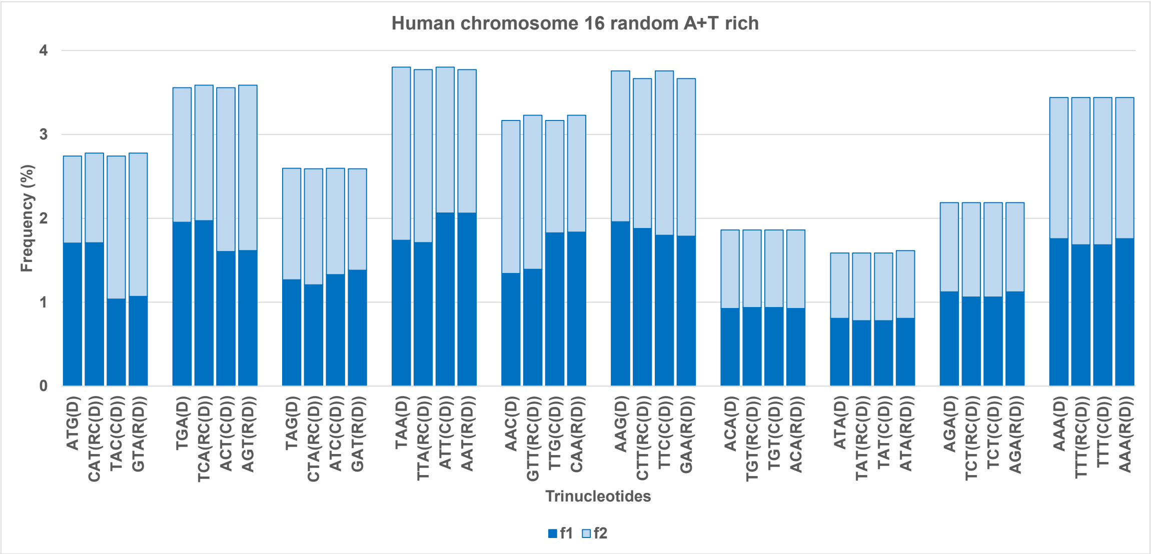

Human coding chromomome 16

A-T rich

D	f	%	RC	f	%	C	f	%	R	f	%	
**ATG**	111343,00	1,50	CAT	105969,00	1,43	TAC	60186,00	0,81	GTA	47197,00	0,63	Ia
**TGA**	129152,00	1,74	TCA	119739,00	1,61	ACT	93097,00	1,25	AGT	93697,00	1,26	
**TAG**	44438,00	0,60	CTA	60500,00	0,81	ATC	85810,00	1,15	GAT	85551,00	1,15	
**TAA**	58679,00	0,79	TTA	61021,00	0,82	ATT	80733,00	1,09	AAT	80952,00	1,09	Ib
**AAC**	85553,00	1,15	GTT	75945,00	1,02	TTG	99859,00	1,34	CAA	112596,00	1,51	
**AAG**	139474,00	1,88	CTT	118893,00	1,60	TTC	114691,00	1,54	GAA	134704,00	1,81	
**ACA**	118337,00	1,59	TGT	117510,00	1,58	TGT*			ACA*			Ic
**ATA**	52708,00	0,71	TAT	58122,00	0,78	TAT*			ATA*			
**AGA**	153415,00	2,06	TCT	125893,00	1,69	TCT*			AGA*			
**AAA**	135530,00	1,82	TTT	130606,00	1,76	TTT*			AAA*			

Quadruplet		f1	f2	fu
ATG	ATG(D)	1,50	0,81	2,31
	CAT(RC(D))	1,43	0,63	2,06
	TAC(C(D))	0,81	1,50	2,31
	GTA(R(D))	0,63	1,43	2,06
TGA	TGA(D)	1,74	1,25	2,99
	TCA(RC(D))	1,61	1,26	2,87
	ACT(C(D))	1,25	1,74	2,99
	AGT(R(D))	1,26	1,61	2,87
TAG	TAG(D)	0,60	1,15	1,75
	CTA(RC(D))	0,81	1,15	1,96
	ATC(C(D))	1,15	0,60	1,75
	GAT(R(D))	1,15	0,81	1,96
TAA	TAA(D)	0,79	1,09	1,88
	TTA(RC(D))	0,82	1,09	1,91
	ATT(C(D))	1,09	0,79	1,88
	AAT(R(D))	1,09	0,82	1,91
AAC	AAC(D)	1,15	1,34	2,49
	GTT(RC(D))	1,02	1,51	2,54
	TTG(C(D))	1,34	1,15	2,49
	CAA(R(D))	1,51	1,02	2,54
AAG	AAG(D)	1,88	1,54	3,42
	CTT(RC(D))	1,60	1,81	3,41
	TTC(C(D))	1,54	1,88	3,42
	GAA(R(D))	1,81	1,60	3,41
ACA	ACA(D)	0,80	0,79	1,59
	TGT(RC(D))	0,79	0,80	1,59
	TGT(C(D))	0,79	0,80	1,59
	ACA(R(D))	0,80	0,79	1,59
ATA	ATA(D)	0,35	0,39	0,75
	TAT(RC(D))	0,39	0,35	0,75
	TAT(C(D))	0,39	0,35	0,75
	ATA(R(D))	0,35	0,35	0,71
AGA	AGA(D)	1,03	0,85	1,88
	TCT(RC(D))	0,85	1,03	1,88
	TCT(C(D))	0,85	1,03	1,88
	AGA(R(D))	1,03	0,85	1,88
AAA	AAA(D)	0,91	0,88	1,79
	TTT(RC(D))	0,88	0,91	1,79
	TTT(C(D))	0,88	0,91	1,79
	AAA(R(D))	0,91	0,88	1,79

Composition of DNA sequence in coding chromosome 16

Base	No.of bases	%
A	1726109	23,22%
C	2044072	27,49%
G	2018378	27,15%
T	1646460	22,14%
N	0	0,00%
Sum	7435019	100,00%

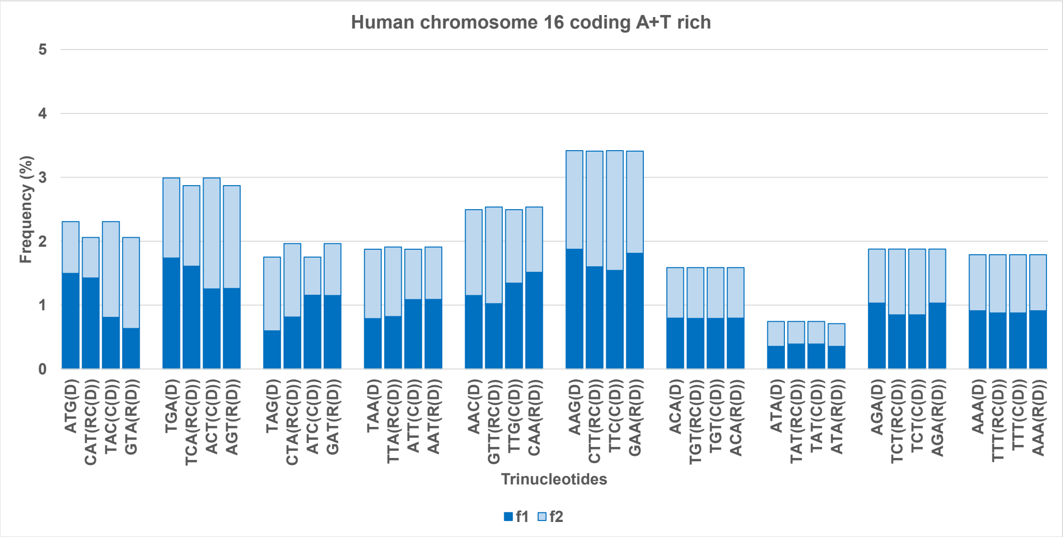

C+G rich											
**D**	**f**	**%**	**RC**	**f**	**%**	**C**	**f**	**%**	**R**	**f**	**%**
**CGT**	279951,00	0,34	ACG	267851,00	0,33	GCA	1286630,00	1,57	TGC	1290928,00	1,58
**GTC**	828820,00	1,01	GAC	836441,00	1,02	CAG	1899034,00	2,32	CTG	1872105,00	2,29
**GCT**	1266418,00	1,55	AGC	1269181,00	1,55	CGA	245231,00	0,30	TCG	256199,00	0,31
**GCC**	1229601,00	1,50	GGC	1226726,00	1,50	CGG	338369,00	0,41	CCG	335315,00	0,41
**CCA**	1675792,00	2,05	TGG	1710296,00	2,09	GGT	1045642,00	1,28	ACC	1040561,00	1,27
**CCT**	1641796,00	2,01	AGG	1633438,00	2,00	GGA	1409734,00	1,72	TCC	1382741,00	1,69
**CAC**	1370283,00	1,68	GTG	1415782,00	1,73	GTG*			CAC*		
**CGC**	287340,00	0,35	GCG	291526,00	0,36	GCG*			CGC*		
**CTC**	1568906,00	1,92	GAG	1573847,00	1,92	GAG*			CTC*		
**CCC**	1325853,00	1,62	GGG	1342046,00	1,64	GGG*			CCC*		

Quadruplet		f1	f2	fu
CGT	CGT(D)	0,34	1,57	1,91
	ACG(RC(D))	0,33	1,58	1,91
	GCA(C(D))	1,57	0,34	1,91
	TGC(R(D))	1,58	0,33	1,91
GTC	GTC(D)	1,01	2,32	3,33
	GAC(RC(D))	1,02	2,29	3,31
	CAG(C(D))	2,32	1,01	3,33
	CTG(R(D))	2,29	1,02	3,31
GCT	GCT(D)	1,55	0,30	1,85
	AGC(RC(D))	1,55	0,31	1,86
	CGA(C(D))	0,30	1,55	1,85
	TCG(R(D))	0,31	1,55	1,86
GCC	GCC(D)	1,50	0,41	1,92
	GGC(RC(D))	1,50	0,41	1,91
	CGG(C(D))	0,41	1,50	1,92
	CCG(R(D))	0,41	1,50	1,91
CCA	CCA(D)	2,05	1,28	3,33
	TGG(RC(D))	2,09	1,27	3,36
	GGT(C(D))	1,28	2,05	3,33
	ACC(R(D))	1,27	2,09	3,36
CCT	CCT(D)	2,01	1,72	3,73
	AGG(RC(D))	2,00	1,69	3,69
	GGA(C(D))	1,72	2,01	3,73
	TCC(R(D))	1,69	2,00	3,69
CAC	CAC(D)	0,84	0,87	1,70
	GTG(RC(D))	0,87	0,84	1,70
	GTG(C(D))	0,87	0,84	1,70
	CAC(R(D))	0,84	0,87	1,70
CGC	CGC(D)	0,18	0,18	0,35
	GCG(RC(D))	0,18	0,18	0,35
	GCG(C(D))	0,18	0,18	0,35
	CGC(R(D))	0,18	0,18	0,35
CTC	CTC(D)	0,96	0,96	1,92
	GAG(RC(D))	0,96	0,96	1,92
	GAG(C(D))	0,96	0,96	1,92
	CTC(R(D))	0,96	0,96	1,92
CCC	CCC(D)	0,81	0,82	1,63
	GGG(RC(D))	0,82	0,81	1,63
	GGG(C(D))	0,82	0,81	1,63
	CCC(R(D))	0,81	0,82	1,63

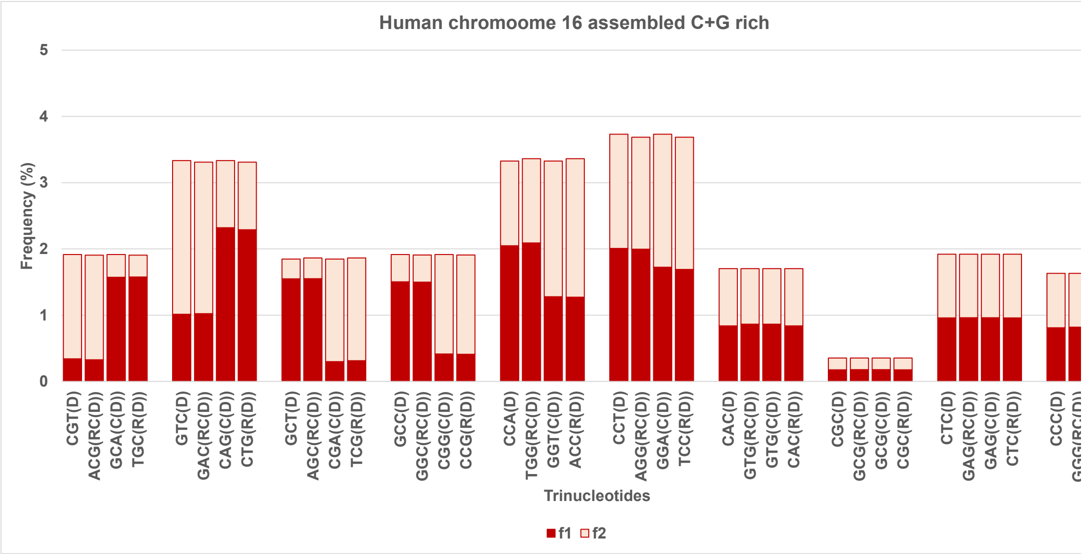

Human random chromomome 16

C+G rich											
**D**	**f**	**%**	**RC**	**f**	**%**	**C**	**f**	**%**	**R**	**f**	**%**
**CGT**	577,00	0,29	ACG	598,00	0,30	GCA	3173,00	1,60	TGC	3069,00	1,54
**GTC**	1988,00	1,00	GAC	2055,00	1,03	CAG	4630,00	2,33	CTG	4470,00	2,25
**GCT**	3121,00	1,57	AGC	3170,00	1,59	CGA	562,00	0,28	TCG	565,00	0,28
**GCC**	2872,00	1,44	GGC	2880,00	1,45	CGG	688,00	0,35	CCG	711,00	0,36
**CCA**	4136,00	2,08	TGG	4108,00	2,07	GGT	2669,00	1,34	ACC	2553,00	1,28
**CCT**	3814,00	1,92	AGG	4136,00	2,08	GGA	3383,00	1,70	TCC	3236,00	1,63
**CAC**	3235,00	1,63	GTG	3292,00	1,66	GTG*			CAC*		
**CGC**	658,00	0,33	GCG	611,00	0,31	GCG*			CGC*		
**CTC**	3741,00	1,88	GAG	3943,00	1,98	GAG*			CTC*		
**CCC**	3053,00	1,54	GGG	3042,00	1,53	GGG*			CCC*		

Quadruplet		f1	f2	fu
CGT	CGT(D)	0,29	1,60	1,89
	ACG(RC(D))	0,30	1,54	1,84
	GCA(C(D))	1,60	0,29	1,89
	TGC(R(D))	1,54	0,30	1,84
GTC	GTC(D)	1,00	2,33	3,33
	GAC(RC(D))	1,03	2,25	3,28
	CAG(C(D))	2,33	1,00	3,33
	CTG(R(D))	2,25	1,03	3,28
GCT	GCT(D)	1,57	0,28	1,85
	AGC(RC(D))	1,59	0,28	1,88
	CGA(C(D))	0,28	1,57	1,85
	TCG(R(D))	0,28	1,59	1,88
GCC	GCC(D)	1,44	0,35	1,79
	GGC(RC(D))	1,45	0,36	1,81
	CGG(C(D))	0,35	1,44	1,79
	CCG(R(D))	0,36	1,45	1,81
CCA	CCA(D)	2,08	1,34	3,42
	TGG(RC(D))	2,07	1,28	3,35
	GGT(C(D))	1,34	2,08	3,42
	ACC(R(D))	1,28	2,07	3,35
CCT	CCT(D)	1,92	1,70	3,62
	AGG(RC(D))	2,08	1,63	3,71
	GGA(C(D))	1,70	1,92	3,62
	TCC(R(D))	1,63	2,08	3,71
CAC	CAC(D)	0,81	0,83	1,64
	GTG(RC(D))	0,83	0,81	1,64
	GTG(C(D))	0,83	0,81	1,64
	CAC(R(D))	0,81	0,83	1,64
CGC	CGC(D)	0,17	0,15	0,32
	GCG(RC(D))	0,15	0,17	0,32
	GCG(C(D))	0,15	0,17	0,32
	CGC(R(D))	0,17	0,15	0,32
CTC	CTC(D)	0,94	0,99	1,93
	GAG(RC(D))	0,99	0,94	1,93
	GAG(C(D))	0,99	0,94	1,93
	CTC(R(D))	0,94	0,99	1,93
CCC	CCC(D)	0,77	0,77	1,53
	GGG(RC(D))	0,77	0,77	1,53
	GGG(C(D))	0,77	0,77	1,53
	CCC(R(D))	0,77	0,77	1,53

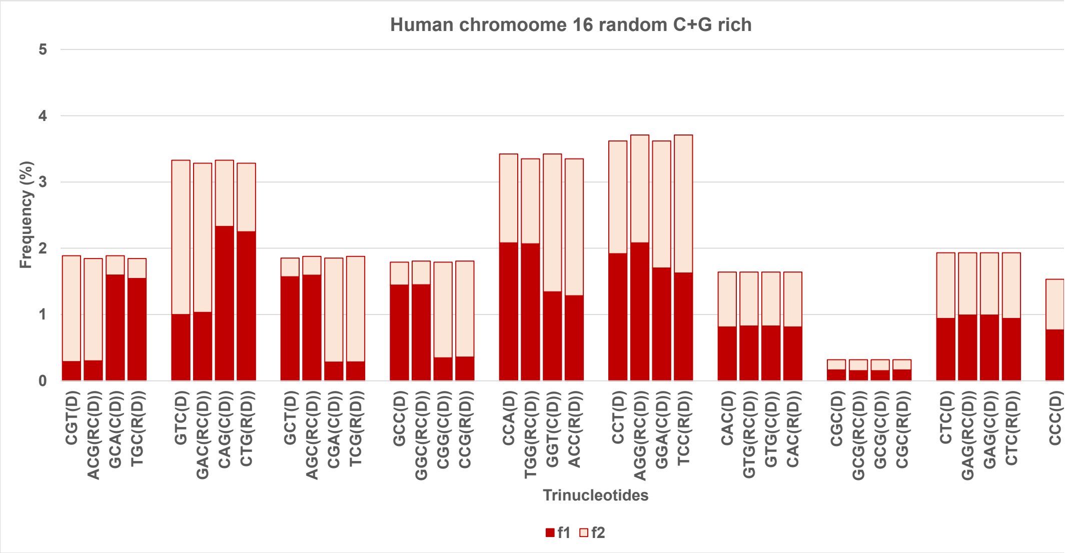

Human coding chromomome 16

C+G rich

D	f	%	RC	f	%	C	f	%	R	f	%
**CGT**	47265,00	0,64	ACG	51665,00	0,69	GCA	139694,00	1,88	TGC	150800,00	2,03
**GTC**	87117,00	1,17	GAC	110086,00	1,48	CAG	209622,00	2,82	CTG	228211,00	3,07
**GCT**	153252,00	2,06	AGC	158810,00	2,14	CGA	51713,00	0,70	TCG	45736,00	0,62
**GCC**	189780,00	2,55	GGC	173951,00	2,34	CGG	92235,00	1,24	CCG	92977,00	1,25
**CCA**	181472,00	2,44	TGG	183481,00	2,47	GGT	93316,00	1,26	ACC	123781,00	1,66
**CCT**	185658,00	2,50	AGG	161203,00	2,17	GGA	169652,00	2,28	TCC	146546,00	1,97
**CAC**	131055,00	1,76	GTG	141530,00	1,90	GTG*			CAC*		
**CGC**	78640,00	1,06	GCG	79475,00	1,07	GCG*			CGC*		
**CTC**	150296,00	2,02	GAG	173591,00	2,33	GAG*			CTC*		
**CCC**	196970,00	2,65	GGG	163537,00	2,20	GGG*			CCC*		

Quadruplet		f1	f2	fu
CGT	CGT(D)	0,64	1,88	2,51
	ACG(RC(D))	0,69	2,03	2,72
	GCA(C(D))	1,88	0,64	2,51
	TGC(R(D))	2,03	0,69	2,72
GTC	GTC(D)	1,17	2,82	3,99
	GAC(RC(D))	1,48	3,07	4,55
	CAG(C(D))	2,82	1,17	3,99
	CTG(R(D))	3,07	1,48	4,55
GCT	GCT(D)	2,06	0,70	2,76
	AGC(RC(D))	2,14	0,62	2,75
	CGA(C(D))	0,70	2,06	2,76
	TCG(R(D))	0,62	2,14	2,75
GCC	GCC(D)	2,55	1,24	3,79
	GGC(RC(D))	2,34	1,25	3,59
	CGG(C(D))	1,24	2,55	3,79
	CCG(R(D))	1,25	2,34	3,59
CCA	CCA(D)	2,44	1,26	3,70
	TGG(RC(D))	2,47	1,66	4,13
	GGT(C(D))	1,26	2,44	3,70
	ACC(R(D))	1,66	2,47	4,13
CCT	CCT(D)	2,50	2,28	4,78
	AGG(RC(D))	2,17	1,97	4,14
	GGA(C(D))	2,28	2,50	4,78
	TCC(R(D))	1,97	2,17	4,14
CAC	CAC(D)	0,88	0,95	1,83
	GTG(RC(D))	0,95	0,88	1,83
	GTG(C(D))	0,95	0,88	1,83
	CAC(R(D))	0,88	0,95	1,83
CGC	CGC(D)	0,53	0,53	1,06
	GCG(RC(D))	0,53	0,53	1,06
	GCG(C(D))	0,53	0,53	1,06
	CGC(R(D))	0,53	0,53	1,06
CTC	CTC(D)	1,01	1,17	2,18
	GAG(RC(D))	1,17	1,01	2,18
	GAG(C(D))	1,17	1,01	2,18
	CTC(R(D))	1,01	1,17	2,18
CCC	CCC(D)	1,32	1,10	2,42
	GGG(RC(D))	1,10	1,32	2,42
	GGG(C(D))	1,10	1,32	2,42
	CCC(R(D))	1,32	1,10	2,42

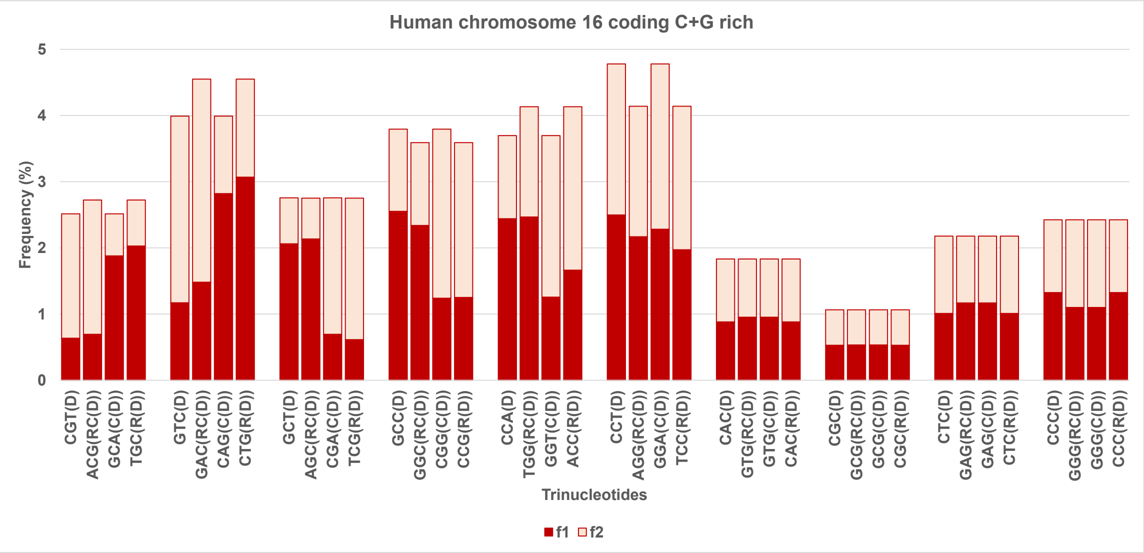

IIa

IIb

IIc

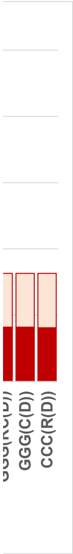

IIa

IIb

IIc

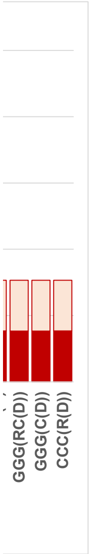

IIa

IIb

IIc

						A-T rich						
**D**	**f**	**%**	**RC**	**f**	**%**	**C**	**f**	**%**	**R**	**f**	**%**	
**ATG**	1347510,00	1,63	CAT	1347013,00	1,62	TAC	799228,00	0,96	GTA	820014,00	0,99	Ia
**TGA**	1575489,00	1,90	TCA	1561503,00	1,88	ACT	1298559,00	1,57	AGT	1280008,00	1,54	
**TAG**	956458,00	1,15	CTA	944546,00	1,14	ATC	1027035,00	1,24	GAT	1068293,00	1,29	
**TAA**	1321456,00	1,59	TTA	1288517,00	1,55	ATT	1635579,00	1,97	AAT	1618882,00	1,95	Ib
**AAC**	1164510,00	1,40	GTT	1118941,00	1,35	TTG	1515960,00	1,83	CAA	1419870,00	1,71	
**AAG**	1526559,00	1,84	CTT	1583094,00	1,91	TTC	1533137,00	1,85	GAA	1568625,00	1,89	
**ACA**	1553740,00	1,87	TGT	1544148,00	1,86	TGT*			ACA*			Ic
**ATA**	1259913,00	1,52	TAT	1235847,00	1,49	TAT*			ATA*			
**AGA**	1785926,00	2,15	TCT	1806326,00	2,18	TCT*			AGA*			
**AAA**	2751246,00	3,32	TTT	2781703,00	3,35	TTT*			AAA*			

Quadruplet		f1	f2	fu
ATG	ATG(D)	1,63	0,96	2,59
	CAT(RC(D))	1,62	0,99	2,61
	TAC(C(D))	0,96	1,63	2,59
	GTA(R(D))	0,99	1,62	2,61
TGA	TGA(D)	1,90	1,57	3,47
	TCA(RC(D))	1,88	1,54	3,43
	ACT(C(D))	1,57	1,90	3,47
	AGT(R(D))	1,54	1,88	3,43
TAG	TAG(D)	1,15	1,24	2,39
	CTA(RC(D))	1,14	1,29	2,43
	ATC(C(D))	1,24	1,15	2,39
	GAT(R(D))	1,29	1,14	2,43
TAA	TAA(D)	1,59	1,97	3,57
	TTA(RC(D))	1,55	1,95	3,51
	ATT(C(D))	1,97	1,59	3,57
	AAT(R(D))	1,95	1,55	3,51
AAC	AAC(D)	1,40	1,83	3,23
	GTT(RC(D))	1,35	1,71	3,06
	TTG(C(D))	1,83	1,40	3,23
	CAA(R(D))	1,71	1,35	3,06
AAG	AAG(D)	1,84	1,85	3,69
	CTT(RC(D))	1,91	1,89	3,80
	TTC(C(D))	1,85	1,84	3,69
	GAA(R(D))	1,89	1,91	3,80
ACA	ACA(D)	0,94	0,93	1,87
	TGT(RC(D))	0,93	0,94	1,87
	TGT(C(D))	0,93	0,94	1,87
	ACA(R(D))	0,94	0,93	1,87
ATA	ATA(D)	0,76	0,75	1,50
	TAT(RC(D))	0,75	0,76	1,50
	TAT(C(D))	0,75	0,76	1,50
	ATA(R(D))	0,76	0,76	1,52
AGA	AGA(D)	1,08	1,09	2,17
	TCT(RC(D))	1,09	1,08	2,17
	TCT(C(D))	1,09	1,08	2,17
	AGA(R(D))	1,08	1,09	2,17
AAA	AAA(D)	1,66	1,68	3,34
	TTT(RC(D))	1,68	1,66	3,34
	TTT(C(D))	1,68	1,66	3,34
	AAA(R(D))	1,66	1,68	3,34

Composition of DNA sequence in assembled chromosome 17

Base	No.of bases	%
A	22639499	27,19%
C	18723944	22,49%
G	18851500	22,64%
T	22705261	27,27%
N	337237	0,41%
Sum	83257441	100,00%

% of coding bases from complete assembled chromosome 1 (including N bases)

12,15%

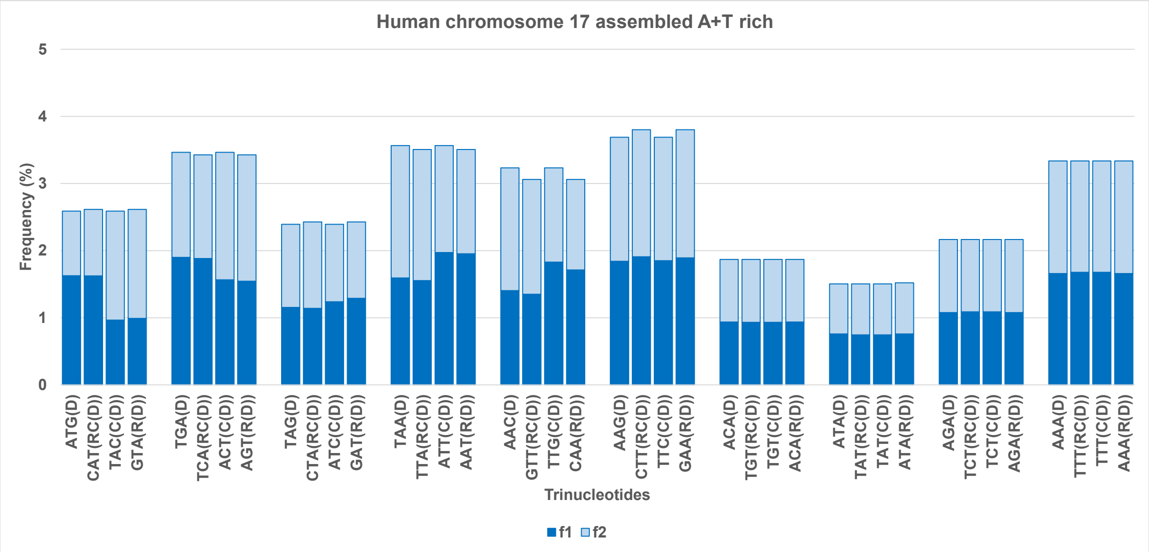

Human random chromomome 17

A-T rich												
**D**	**f**	**%**	**RC**	**f**	**%**	**C**	**f**	**%**	**R**	**f**	**%**	
**ATG**	3225,00	1,62	CAT	3250,00	1,63	TAC	1961,00	0,98	GTA	1991,00	1,00	Ia
**TGA**	3911,00	1,96	TCA	3794,00	1,90	ACT	3212,00	1,61	AGT	3167,00	1,59	
**TAG**	2364,00	1,18	CTA	2352,00	1,18	ATC	2477,00	1,24	GAT	2608,00	1,31	
**TAA**	3351,00	1,68	TTA	3190,00	1,60	ATT	4076,00	2,04	AAT	4064,00	2,04	Ib
**AAC**	2889,00	1,45	GTT	2708,00	1,36	TTG	3744,00	1,88	CAA	3489,00	1,75	
**AAG**	3741,00	1,87	CTT	3963,00	1,99	TTC	3813,00	1,91	GAA	3854,00	1,93	
**ACA**	3634,00	1,82	TGT	3675,00	1,84	TGT*			ACA*			Ic
**ATA**	3131,00	1,57	TAT	2987,00	1,50	TAT*			ATA*			
**AGA**	4342,00	2,18	TCT	4467,00	2,24	TCT*			AGA*			
**AAA**	6666,00	3,34	TTT	7062,00	3,54	TTT*			AAA*			

Quadruplet		f1	f2	fu
	ATG(D)	1,62	0,98	2,60
ATG	CAT(RC(D))	1,63	1,00	2,63
	TAC(C(D))	0,98	1,62	2,60
	GTA(R(D))	1,00	1,63	2,63
	TGA(D)	1,96	1,61	3,57
TGA	TCA(RC(D))	1,90	1,59	3,49
	ACT(C(D))	1,61	1,96	3,57
	AGT(R(D))	1,59	1,90	3,49
	TAG(D)	1,18	1,24	2,43
TAG	CTA(RC(D))	1,18	1,31	2,49
	ATC(C(D))	1,24	1,18	2,43
	GAT(R(D))	1,31	1,18	2,49
	TAA(D)	1,68	2,04	3,72
TAA	TTA(RC(D))	1,60	2,04	3,63
	ATT(C(D))	2,04	1,68	3,72
	AAT(R(D))	2,04	1,60	3,63
	AAC(D)	1,45	1,88	3,32
AAC	GTT(RC(D))	1,36	1,75	3,10
	TTG(C(D))	1,88	1,45	3,32
	CAA(R(D))	1,75	1,36	3,10
	AAG(D)	1,87	1,91	3,78
AAG	CTT(RC(D))	1,99	1,93	3,92
	TTC(C(D))	1,91	1,87	3,78
	GAA(R(D))	1,93	1,99	3,92
	ACA(D)	0,91	0,92	1,83
ACA	TGT(RC(D))	0,92	0,91	1,83
	TGT(C(D))	0,92	0,91	1,83
	ACA(R(D))	0,91	0,92	1,83
	ATA(D)	0,78	0,75	1,53
ATA	TAT(RC(D))	0,75	0,78	1,53
	TAT(C(D))	0,75	0,78	1,53
	ATA(R(D))	0,78	0,78	1,57
	AGA(D)	1,09	1,12	2,21
AGA	TCT(RC(D))	1,12	1,09	2,21
	TCT(C(D))	1,12	1,09	2,21
	AGA(R(D))	1,09	1,12	2,21
	AAA(D)	1,67	1,77	3,44
AAA	TTT(RC(D))	1,77	1,67	3,44
	TTT(C(D))	1,77	1,67	3,44
	AAA(R(D))	1,67	1,77	3,44

Composition of DNA sequence in random chromosome 17

Base	No.of bases	%
A	55084	27,54%
C	44389	22,19%
G	44587	22,29%
T	55540	27,77%
N	400	0,20%
Sum	200000	100,00%

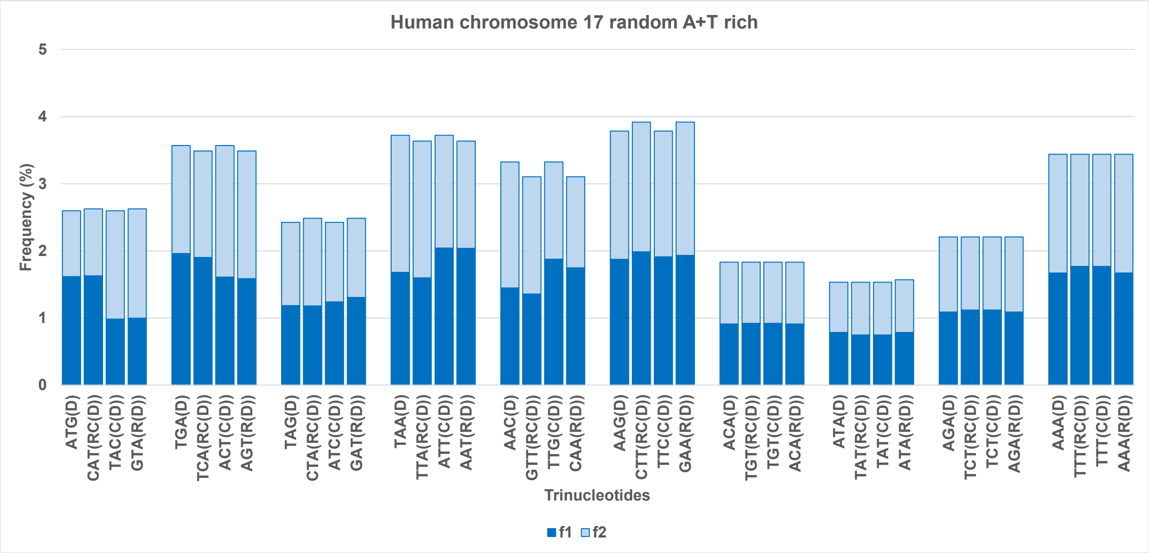

Human coding chromomome 17

A-T rich

D	f	%	RC	f	%	C	f	%	R	f	%	
**ATG**	156503,00	1,55	CAT	145862,00	1,44	TAC	85386,00	0,84	GTA	67364,00	0,67	Ia
**TGA**	181979,00	1,80	TCA	165689,00	1,64	ACT	130596,00	1,29	AGT	129127,00	1,28	
**TAG**	62952,00	0,62	CTA	86622,00	0,86	ATC	121338,00	1,20	GAT	122330,00	1,21	
**TAA**	80514,00	0,80	TTA	83693,00	0,83	ATT	112645,00	1,11	AAT	113467,00	1,12	Ib
**AAC**	120823,00	1,19	GTT	103287,00	1,02	TTG	140244,00	1,39	CAA	160506,00	1,59	
**AAG**	199767,00	1,97	CTT	166002,00	1,64	TTC	157997,00	1,56	GAA	193037,00	1,91	
**ACA**	161428,00	1,60	TGT	160950,00	1,59	TGT*			ACA*			Ic
**ATA**	73530,00	0,73	TAT	82357,00	0,81	TAT*			ATA*			
**AGA**	219013,00	2,16	TCT	175052,00	1,73	TCT*			AGA*			
**AAA**	187954,00	1,86	TTT	175877,00	1,74	TTT*			AAA*			

Quadruplet		f1	f2	fu
ATG	ATG(D)	1,55	0,84	2,39
	CAT(RC(D))	1,44	0,67	2,11
	TAC(C(D))	0,84	1,55	2,39
	GTA(R(D))	0,67	1,44	2,11
TGA	TGA(D)	1,80	1,29	3,09
	TCA(RC(D))	1,64	1,28	2,91
	ACT(C(D))	1,29	1,80	3,09
	AGT(R(D))	1,28	1,64	2,91
TAG	TAG(D)	0,62	1,20	1,82
	CTA(RC(D))	0,86	1,21	2,07
	ATC(C(D))	1,20	0,62	1,82
	GAT(R(D))	1,21	0,86	2,07
TAA	TAA(D)	0,80	1,11	1,91
	TTA(RC(D))	0,83	1,12	1,95
	ATT(C(D))	1,11	0,80	1,91
	AAT(R(D))	1,12	0,83	1,95
AAC	AAC(D)	1,19	1,39	2,58
	GTT(RC(D))	1,02	1,59	2,61
	TTG(C(D))	1,39	1,19	2,58
	CAA(R(D))	1,59	1,02	2,61
AAG	AAG(D)	1,97	1,56	3,54
	CTT(RC(D))	1,64	1,91	3,55
	TTC(C(D))	1,56	1,97	3,54
	GAA(R(D))	1,91	1,64	3,55
ACA	ACA(D)	0,80	0,80	1,59
	TGT(RC(D))	0,80	0,80	1,59
	TGT(C(D))	0,80	0,80	1,59
	ACA(R(D))	0,80	0,80	1,59
ATA	ATA(D)	0,36	0,41	0,77
	TAT(RC(D))	0,41	0,36	0,77
	TAT(C(D))	0,41	0,36	0,77
	ATA(R(D))	0,36	0,36	0,73
AGA	AGA(D)	1,08	0,87	1,95
	TCT(RC(D))	0,87	1,08	1,95
	TCT(C(D))	0,87	1,08	1,95
	AGA(R(D))	1,08	0,87	1,95
AAA	AAA(D)	0,93	0,87	1,80
	TTT(RC(D))	0,87	0,93	1,80
	TTT(C(D))	0,87	0,93	1,80
	AAA(R(D))	0,93	0,87	1,80

Composition of DNA sequence in coding chromosome 17

Base	No.of bases	%
A	2401611	23,738%
C	2732997	27,014%
G	2722841	26,913%
T	2259639	22,335%
N	0	0,00%
Sum	10117088	100,00%

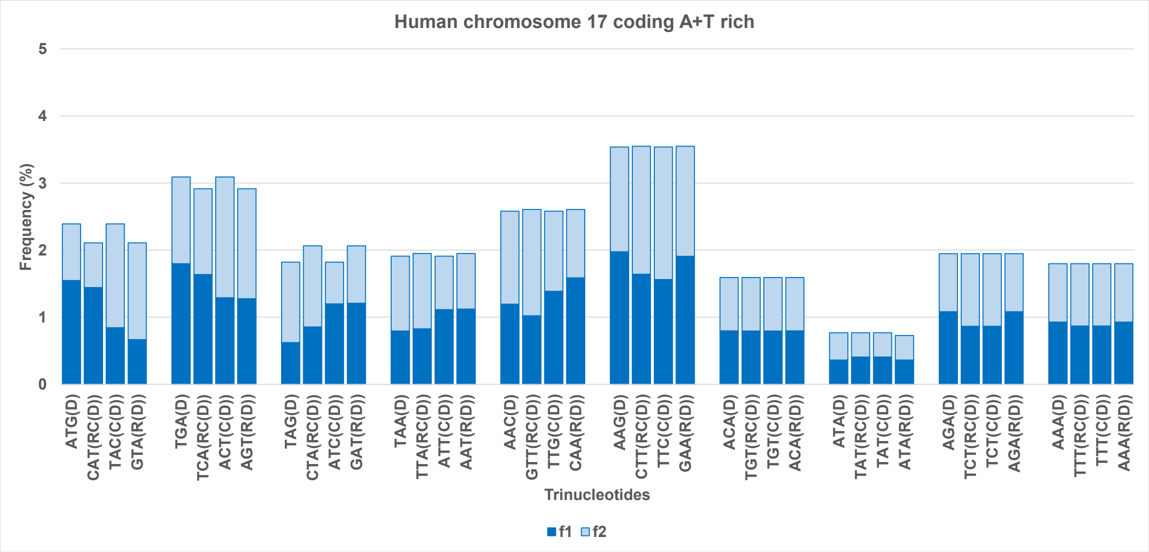

C+G rich											
**D**	**f**	**%**	**RC**	**f**	**%**	**C**	**f**	**%**	**R**	**f**	**%**
**CGT**	285606,00	0,34	ACG	286180,00	0,35	GCA	1328180,00	1,60	TGC	1301019,00	1,57
**GTC**	825753,00	1,00	GAC	847141,00	1,02	CAG	1967932,00	2,37	CTG	1935828,00	2,33
**GCT**	1306367,00	1,58	AGC	1328163,00	1,60	CGA	257406,00	0,31	TCG	272305,00	0,33
**GCC**	1296568,00	1,56	GGC	1296097,00	1,56	CGG	380784,00	0,46	CCG	371149,00	0,45
**CCA**	1694991,00	2,04	TGG	1794269,00	2,16	GGT	1070574,00	1,29	ACC	1076002,00	1,30
**CCT**	1724306,00	2,08	AGG	1699665,00	2,05	GGA	1508047,00	1,82	TCC	1417872,00	1,71
**CAC**	1403599,00	1,69	GTG	1415628,00	1,71	GTG*			CAC*		
**CGC**	324530,00	0,39	GCG	318693,00	0,38	GCG*			CGC*		
**CTC**	1672089,00	2,02	GAG	1642808,00	1,98	GAG*			CTC*		
**CCC**	1411178,00	1,70	GGG	1419746,00	1,71	GGG*			CCC*		

Quadruplet		f1	f2	fu
CGT	CGT(D)	0,34	1,60	1,95
	ACG(RC(D))	0,35	1,57	1,91
	GCA(C(D))	1,60	0,34	1,95
	TGC(R(D))	1,57	0,35	1,91
GTC	GTC(D)	1,00	2,37	3,37
	GAC(RC(D))	1,02	2,33	3,36
	CAG(C(D))	2,37	1,00	3,37
	CTG(R(D))	2,33	1,02	3,36
GCT	GCT(D)	1,58	0,31	1,89
	AGC(RC(D))	1,60	0,33	1,93
	CGA(C(D))	0,31	1,58	1,89
	TCG(R(D))	0,33	1,60	1,93
GCC	GCC(D)	1,56	0,46	2,02
	GGC(RC(D))	1,56	0,45	2,01
	CGG(C(D))	0,46	1,56	2,02
	CCG(R(D))	0,45	1,56	2,01
CCA	CCA(D)	2,04	1,29	3,34
	TGG(RC(D))	2,16	1,30	3,46
	GGT(C(D))	1,29	2,04	3,34
	ACC(R(D))	1,30	2,16	3,46
CCT	CCT(D)	2,08	1,82	3,90
	AGG(RC(D))	2,05	1,71	3,76
	GGA(C(D))	1,82	2,08	3,90
	TCC(R(D))	1,71	2,05	3,76
CAC	CAC(D)	0,85	0,85	1,70
	GTG(RC(D))	0,85	0,85	1,70
	GTG(C(D))	0,85	0,85	1,70
	CAC(R(D))	0,85	0,85	1,70
CGC	CGC(D)	0,20	0,19	0,39
	GCG(RC(D))	0,19	0,20	0,39
	GCG(C(D))	0,19	0,20	0,39
	CGC(R(D))	0,20	0,19	0,39
CTC	CTC(D)	1,01	0,99	2,00
	GAG(RC(D))	0,99	1,01	2,00
	GAG(C(D))	0,99	1,01	2,00
	CTC(R(D))	1,01	0,99	2,00
CCC	CCC(D)	0,85	0,86	1,71
	GGG(RC(D))	0,86	0,85	1,71
	GGG(C(D))	0,86	0,85	1,71
	CCC(R(D))	0,85	0,86	1,71

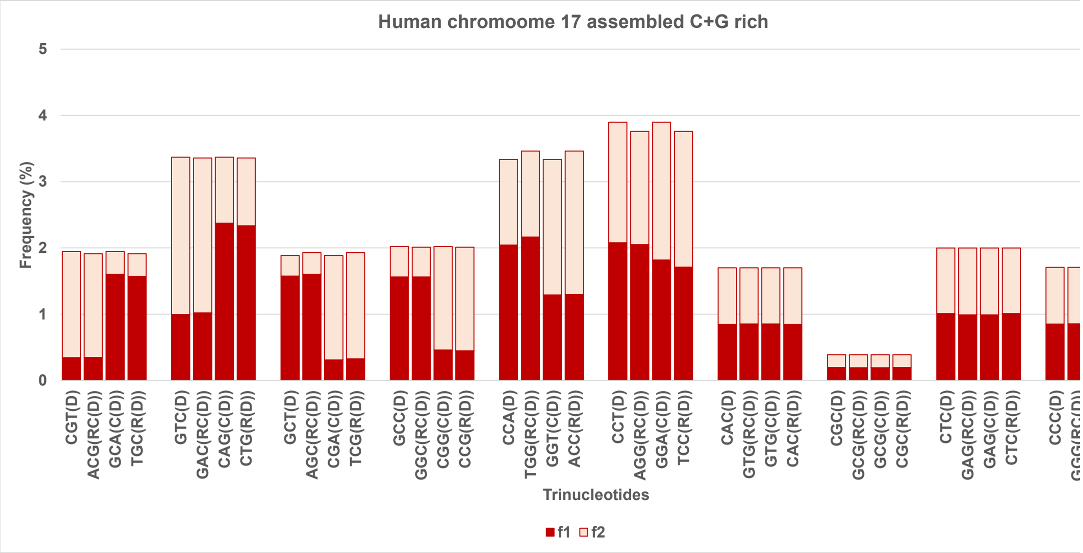

Human random chromomome 17

C+G rich											
D	f	%	RC	f	%	C	f	%	R	f	%
**CGT**	650,00	0,33	ACG	633,00	0,32	GCA	3141,00	1,57	TGC	3107,00	1,56
**GTC**	1945,00	0,97	GAC	2004,00	1,00	CAG	4684,00	2,35	CTG	4509,00	2,26
**GCT**	3093,00	1,55	AGC	3227,00	1,62	CGA	634,00	0,32	TCG	626,00	0,31
**GCC**	2999,00	1,50	GGC	2910,00	1,46	CGG	802,00	0,40	CCG	816,00	0,41
**CCA**	4036,00	2,02	TGG	4137,00	2,07	GGT	2504,00	1,25	ACC	2559,00	1,28
**CCT**	4054,00	2,03	AGG	4040,00	2,02	GGA	3565,00	1,79	TCC	3348,00	1,68
**CAC**	3183,00	1,59	GTG	3352,00	1,68	GTG*			CAC*		
**CGC**	675,00	0,34	GCG	686,00	0,34	GCG*			CGC*		
**CTC**	4001,00	2,00	GAG	3986,00	2,00	GAG*			CTC*		
**CCC**	3289,00	1,65	GGG	3241,00	1,62	GGG*			CCC*		

Quadruplet		f1	f2	fu
CGT	CGT(D)	0,33	1,57	1,90
	ACG(RC(D))	0,32	1,56	1,87
	GCA(C(D))	1,57	0,33	1,90
	TGC(R(D))	1,56	0,32	1,87
GTC	GTC(D)	0,97	2,35	3,32
	GAC(RC(D))	1,00	2,26	3,26
	CAG(C(D))	2,35	0,97	3,32
	CTG(R(D))	2,26	1,00	3,26
GCT	GCT(D)	1,55	0,32	1,87
	AGC(RC(D))	1,62	0,31	1,93
	CGA(C(D))	0,32	1,55	1,87
	TCG(R(D))	0,31	1,62	1,93
GCC	GCC(D)	1,50	0,40	1,90
	GGC(RC(D))	1,46	0,41	1,87
	CGG(C(D))	0,40	1,50	1,90
	CCG(R(D))	0,41	1,46	1,87
CCA	CCA(D)	2,02	1,25	3,28
	TGG(RC(D))	2,07	1,28	3,35
	GGT(C(D))	1,25	2,02	3,28
	ACC(R(D))	1,28	2,07	3,35
CCT	CCT(D)	2,03	1,79	3,82
	AGG(RC(D))	2,02	1,68	3,70
	GGA(C(D))	1,79	2,03	3,82
	TCC(R(D))	1,68	2,02	3,70
CAC	CAC(D)	0,80	0,84	1,64
	GTG(RC(D))	0,84	0,80	1,64
	GTG(C(D))	0,84	0,80	1,64
	CAC(R(D))	0,80	0,84	1,64
CGC	CGC(D)	0,17	0,17	0,34
	GCG(RC(D))	0,17	0,17	0,34
	GCG(C(D))	0,17	0,17	0,34
	CGC(R(D))	0,17	0,17	0,34
CTC	CTC(D)	1,00	1,00	2,00
	GAG(RC(D))	1,00	1,00	2,00
	GAG(C(D))	1,00	1,00	2,00
	CTC(R(D))	1,00	1,00	2,00
CCC	CCC(D)	0,82	0,81	1,64
	GGG(RC(D))	0,81	0,82	1,64
	GGG(C(D))	0,81	0,82	1,64
	CCC(R(D))	0,82	0,81	1,64

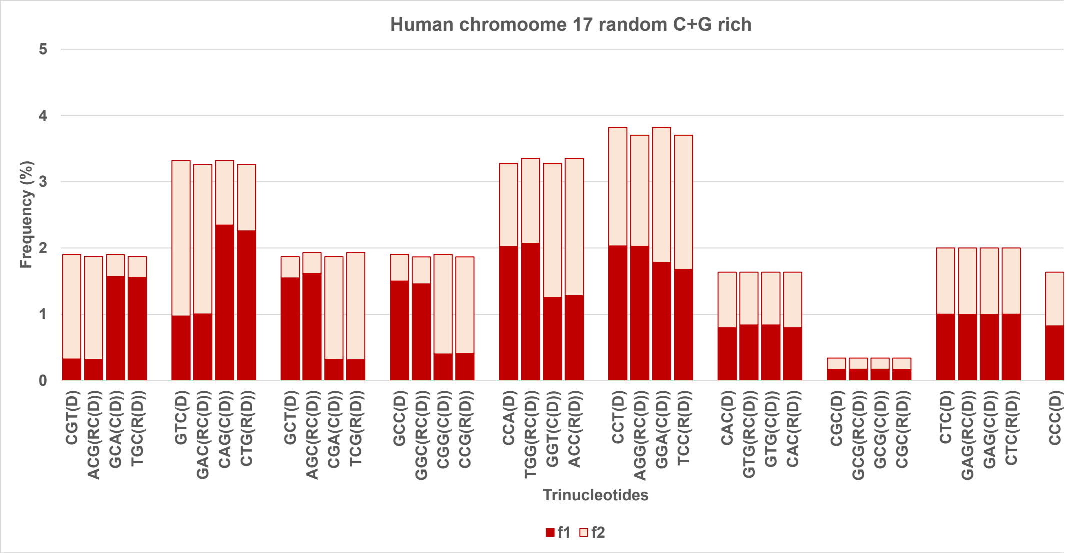

Human coding chromomome 17

C+G rich

D	f	%	RC	f	%	C	f	%	R	f	%
**CGT**	58258,00	0,58	ACG	63351,00	0,63	GCA	188612,00	1,86	TGC	197832,00	1,96
**GTC**	116907,00	1,16	GAC	146614,00	1,45	CAG	288452,00	2,85	CTG	306237,00	3,03
**GCT**	207582,00	2,05	AGC	220062,00	2,18	CGA	65632,00	0,65	TCG	59030,00	0,58
**GCC**	244654,00	2,42	GGC	223945,00	2,21	CGG	113333,00	1,12	CCG	113851,00	1,13
**CCA**	249655,00	2,47	TGG	250871,00	2,48	GGT	127872,00	1,26	ACC	168012,00	1,66
**CCT**	248375,00	2,46	AGG	223995,00	2,21	GGA	236382,00	2,34	TCC	199215,00	1,97
**CAC**	170564,00	1,69	GTG	188649,00	1,86	GTG*			CAC*		
**CGC**	94344,00	0,93	GCG	95335,00	0,94	GCG*			CGC*		
**CTC**	202744,00	2,00	GAG	241026,00	2,38	GAG*			CTC*		
**CCC**	262560,00	2,60	GGG	219244,00	2,17	GGG*			CCC*		

Quadruplet		f1	f2	fu
CGT	CGT(D)	0,58	1,86	2,44
	ACG(RC(D))	0,63	1,96	2,58
	GCA(C(D))	1,86	0,58	2,44
	TGC(R(D))	1,96	0,63	2,58
GTC	GTC(D)	1,16	2,85	4,01
	GAC(RC(D))	1,45	3,03	4,48
	CAG(C(D))	2,85	1,16	4,01
	CTG(R(D))	3,03	1,45	4,48
GCT	GCT(D)	2,05	0,65	2,70
	AGC(RC(D))	2,18	0,58	2,76
	CGA(C(D))	0,65	2,05	2,70
	TCG(R(D))	0,58	2,18	2,76
GCC	GCC(D)	2,42	1,12	3,54
	GGC(RC(D))	2,21	1,13	3,34
	CGG(C(D))	1,12	2,42	3,54
	CCG(R(D))	1,13	2,21	3,34
CCA	CCA(D)	2,47	1,26	3,73
	TGG(RC(D))	2,48	1,66	4,14
	GGT(C(D))	1,26	2,47	3,73
	ACC(R(D))	1,66	2,48	4,14
CCT	CCT(D)	2,46	2,34	4,79
	AGG(RC(D))	2,21	1,97	4,18
	GGA(C(D))	2,34	2,46	4,79
	TCC(R(D))	1,97	2,21	4,18
CAC	CAC(D)	0,84	0,93	1,78
	GTG(RC(D))	0,93	0,84	1,78
	GTG(C(D))	0,93	0,84	1,78
	CAC(R(D))	0,84	0,93	1,78
CGC	CGC(D)	0,47	0,47	0,94
	GCG(RC(D))	0,47	0,47	0,94
	GCG(C(D))	0,47	0,47	0,94
	CGC(R(D))	0,47	0,47	0,94
CTC	CTC(D)	1,00	1,19	2,19
	GAG(RC(D))	1,19	1,00	2,19
	GAG(C(D))	1,19	1,00	2,19
	CTC(R(D))	1,00	1,19	2,19
CCC	CCC(D)	1,30	1,08	2,38
	GGG(RC(D))	1,08	1,30	2,38
	GGG(C(D))	1,08	1,30	2,38
	CCC(R(D))	1,30	1,08	2,38

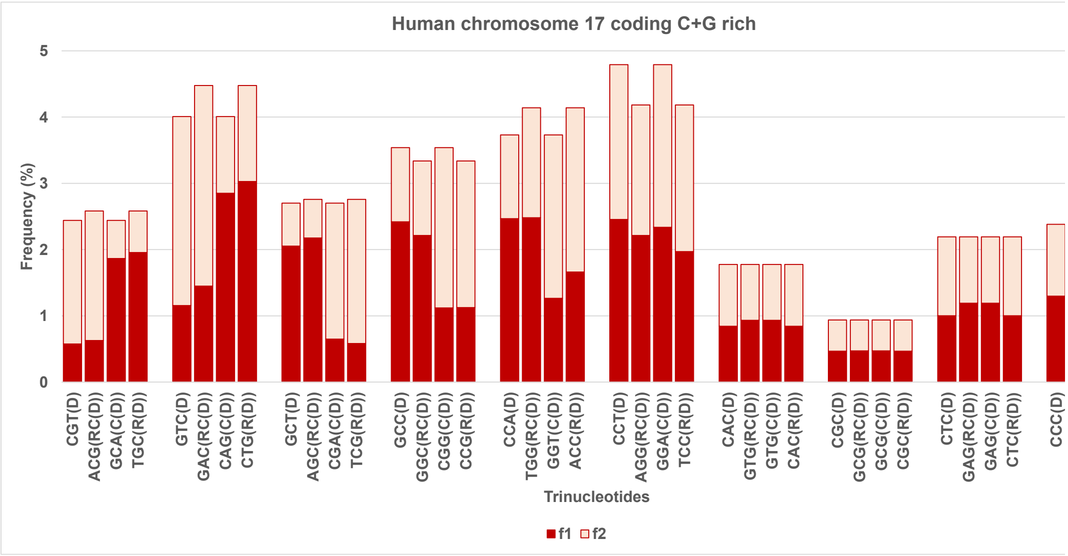

IIa

IIb

IIc

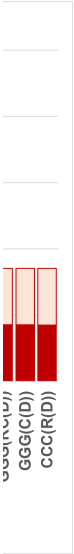

IIa

IIb

IIc

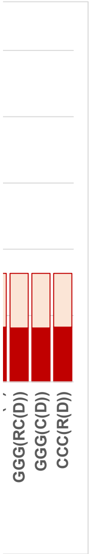

IIa

IIb

IIc

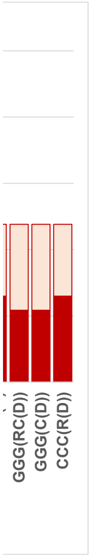
GGG(RC(D))
GGG(C(D))
CCC(R(D))

Human assembled chromomome 18

						A-T rich						
**D**	**f**	**%**	**RC**	**f**	**%**	**C**	**f**	**%**	**R**	**f**	**%**	
**ATG**	1457945,00	1,82	CAT	1519379,00	1,90	TAC	896228,00	1,12	GTA	931880,00	1,16	Ia
**TGA**	1598290,00	2,00	TCA	1589895,00	1,99	ACT	1313826,00	1,64	AGT	1316477,00	1,64	
**TAG**	1063514,00	1,33	CTA	1048580,00	1,31	ATC	1074345,00	1,34	GAT	1090522,00	1,36	
**TAA**	1694373,00	2,12	TTA	1668022,00	2,08	ATT	2103711,00	2,63	AAT	2031727,00	2,54	Ib
**AAC**	1254402,00	1,57	GTT	1220202,00	1,52	TTG	1643656,00	2,05	CAA	1493970,00	1,87	
**AAG**	1627964,00	2,03	CTT	1659497,00	2,07	TTC	1671728,00	2,09	GAA	1725747,00	2,15	
**ACA**	1680341,00	2,10	TGT	1664521,00	2,08	TGT*			ACA*			Ic
**ATA**	1722339,00	2,15	TAT	1716708,00	2,14	TAT*			ATA*			
**AGA**	1823057,00	2,28	TCT	1814990,00	2,27	TCT*			AGA*			
**AAA**	3146593,00	3,93	TTT	3268887,00	4,08	TTT*			AAA*			

Quadruplet		f1	f2	fu
ATG	ATG(D)	1,82	1,12	2,94
	CAT(RC(D))	1,90	1,16	3,06
	TAC(C(D))	1,12	1,82	2,94
	GTA(R(D))	1,16	1,90	3,06
TGA	TGA(D)	2,00	1,64	3,64
	TCA(RC(D))	1,99	1,64	3,63
	ACT(C(D))	1,64	2,00	3,64
	AGT(R(D))	1,64	1,99	3,63
TAG	TAG(D)	1,33	1,34	2,67
	CTA(RC(D))	1,31	1,36	2,67
	ATC(C(D))	1,34	1,33	2,67
	GAT(R(D))	1,36	1,31	2,67
TAA	TAA(D)	2,12	2,63	4,74
	TTA(RC(D))	2,08	2,54	4,62
	ATT(C(D))	2,63	2,12	4,74
	AAT(R(D))	2,54	2,08	4,62
AAC	AAC(D)	1,57	2,05	3,62
	GTT(RC(D))	1,52	1,87	3,39
	TTG(C(D))	2,05	1,57	3,62
	CAA(R(D))	1,87	1,52	3,39
AAG	AAG(D)	2,03	2,09	4,12
	CTT(RC(D))	2,07	2,15	4,23
	TTC(C(D))	2,09	2,03	4,12
	GAA(R(D))	2,15	2,07	4,23
ACA	ACA(D)	1,05	1,04	2,09
	TGT(RC(D))	1,04	1,05	2,09
	TGT(C(D))	1,04	1,05	2,09
	ACA(R(D))	1,05	1,04	2,09
ATA	ATA(D)	1,08	1,07	2,15
	TAT(RC(D))	1,07	1,08	2,15
	TAT(C(D))	1,07	1,08	2,15
	ATA(R(D))	1,08	1,08	2,15
AGA	AGA(D)	1,14	1,13	2,27
	TCT(RC(D))	1,13	1,14	2,27
	TCT(C(D))	1,13	1,14	2,27
	AGA(R(D))	1,14	1,13	2,27
AAA	AAA(D)	1,96	2,04	4,01
	TTT(RC(D))	2,04	1,96	4,01
	TTT(C(D))	2,04	1,96	4,01
	AAA(R(D))	1,96	2,04	4,01

Composition of DNA sequence in assembled chromosome 18

Base	No.of bases	%
A	24050680	29,92%
C	15794455	19,65%
G	16061651	19,98%
T	24182819	30,09%
N	283680	0,35%
Sum	80373285	100,00%

% of coding bases from complete assembled chromosome 1 (including N bases)

**4,15%**

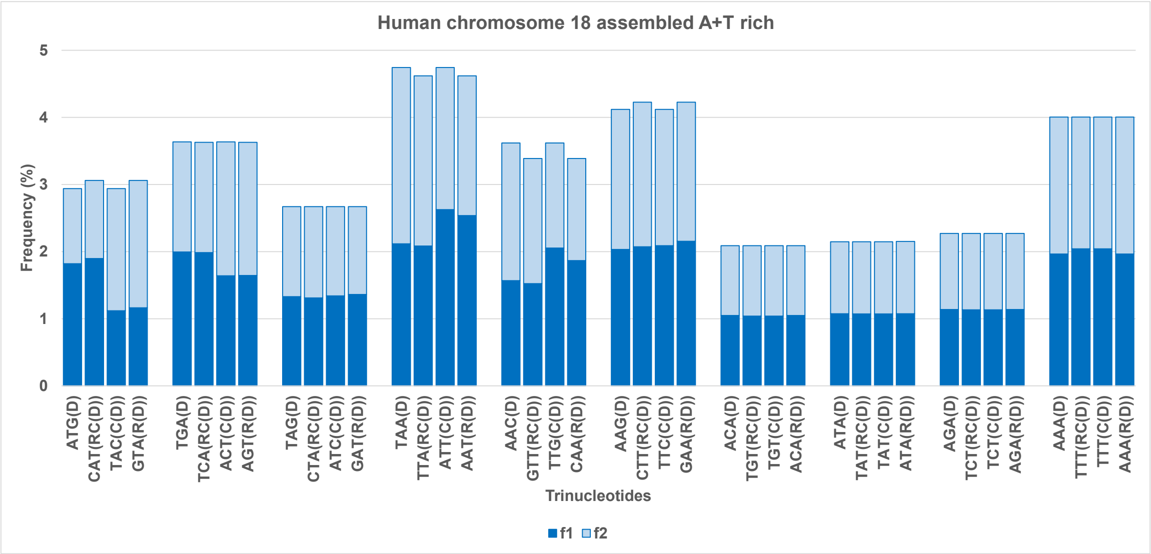

Human random chromomome 18

A-T rich

D	f	%	RC	f	%	C	f	%	R	f	%	
**ATG**	3623,00	1,82	CAT	3780,00	1,90	TAC	2364,00	1,19	GTA	2416,00	1,21	Ia
**TGA**	3966,00	1,99	TCA	3861,00	1,94	ACT	3354,00	1,68	AGT	3249,00	1,63	
**TAG**	2727,00	1,37	CTA	2664,00	1,34	ATC	2615,00	1,31	GAT	2697,00	1,35	
**TAA**	4262,00	2,14	TTA	4137,00	2,07	ATT	5308,00	2,66	AAT	5205,00	2,61	Ib
**AAC**	3089,00	1,55	GTT	3037,00	1,52	TTG	4159,00	2,09	CAA	3714,00	1,86	
**AAG**	4125,00	2,07	CTT	4172,00	2,09	TTC	4220,00	2,12	GAA	4445,00	2,23	
**ACA**	4217,00	2,11	TGT	4191,00	2,10	TGT*			ACA*			Ic
**ATA**	4511,00	2,26	TAT	4375,00	2,19	TAT*			ATA*			
**AGA**	4579,00	2,30	TCT	4566,00	2,29	TCT*			AGA*			
**AAA**	7818,00	3,92	TTT	8230,00	4,13	TTT*			AAA*			

Quadruplet		f1	f2	fu
ATG	ATG(D)	1,82	1,19	3,00
	CAT(RC(D))	1,90	1,21	3,11
	TAC(C(D))	1,19	1,82	3,00
	GTA(R(D))	1,21	1,90	3,11
TGA	TGA(D)	1,99	1,68	3,67
	TCA(RC(D))	1,94	1,63	3,57
	ACT(C(D))	1,68	1,99	3,67
	AGT(R(D))	1,63	1,94	3,57
TAG	TAG(D)	1,37	1,31	2,68
	CTA(RC(D))	1,34	1,35	2,69
	ATC(C(D))	1,31	1,37	2,68
	GAT(R(D))	1,35	1,34	2,69
TAA	TAA(D)	2,14	2,66	4,80
	TTA(RC(D))	2,07	2,61	4,69
	ATT(C(D))	2,66	2,14	4,80
	AAT(R(D))	2,61	2,07	4,69
AAC	AAC(D)	1,55	2,09	3,64
	GTT(RC(D))	1,52	1,86	3,39
	TTG(C(D))	2,09	1,55	3,64
	CAA(R(D))	1,86	1,52	3,39
AAG	AAG(D)	2,07	2,12	4,19
	CTT(RC(D))	2,09	2,23	4,32
	TTC(C(D))	2,12	2,07	4,19
	GAA(R(D))	2,23	2,09	4,32
ACA	ACA(D)	1,06	1,05	2,11
	TGT(RC(D))	1,05	1,06	2,11
	TGT(C(D))	1,05	1,06	2,11
	ACA(R(D))	1,06	1,05	2,11
ATA	ATA(D)	1,13	1,10	2,23
	TAT(RC(D))	1,10	1,13	2,23
	TAT(C(D))	1,10	1,13	2,23
	ATA(R(D))	1,13	1,13	2,26
AGA	AGA(D)	1,15	1,14	2,29
	TCT(RC(D))	1,14	1,15	2,29
	TCT(C(D))	1,14	1,15	2,29
	AGA(R(D))	1,15	1,14	2,29
AAA	AAA(D)	1,96	2,06	4,02
	TTT(RC(D))	2,06	1,96	4,02
	TTT(C(D))	2,06	1,96	4,02
	AAA(R(D))	1,96	2,06	4,02

Composition of DNA sequence in random chromosome 18

Base	No.of bases	%
A	60356	30,18%
C	38898	19,45%
G	39551	19,78%
T	60595	30,30%
N	600	0,30%
Sum	200000	100,00%

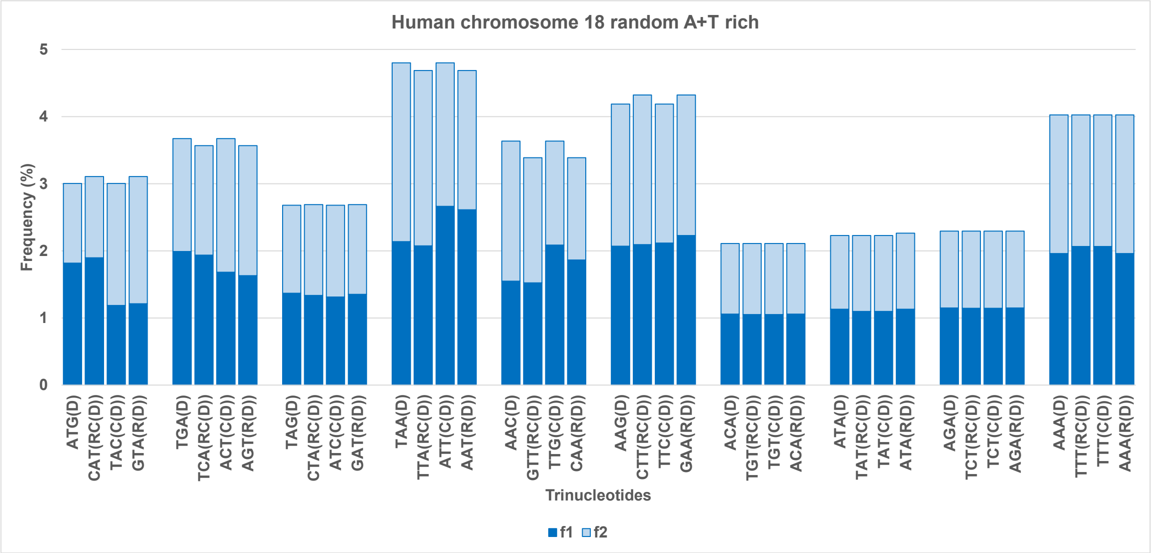

Human coding chromomome 18

A-T rich

D	f	%	RC	f	%	C	f	%	R	f	%	
**ATG**	63793,00	1,91	CAT	55251,00	1,66	TAC	33908,00	1,02	GTA	31116,00	0,93	Ia
**TGA**	66924,00	2,01	TCA	61785,00	1,85	ACT	49106,00	1,47	AGT	51549,00	1,55	
**TAG**	29935,00	0,90	CTA	33471,00	1,00	ATC	43399,00	1,30	GAT	47413,00	1,42	
**TAA**	48513,00	1,46	TTA	51605,00	1,55	ATT	63723,00	1,91	AAT	64415,00	1,93	Ib
**AAC**	48796,00	1,46	GTT	45855,00	1,38	TTG	58740,00	1,76	CAA	61102,00	1,83	
**AAG**	74253,00	2,23	CTT	61078,00	1,83	TTC	60311,00	1,81	GAA	77849,00	2,33	
**ACA**	62556,00	1,88	TGT	62168,00	1,86	TGT*			ACA*			Ic
**ATA**	45632,00	1,37	TAT	49468,00	1,48	TAT*			ATA*			
**AGA**	80681,00	2,42	TCT	61258,00	1,84	TCT*			AGA*			
**AAA**	105416,00	3,16	TTT	96719,00	2,90	TTT*			AAA*			

Quadruplet		f1	f2	fu
ATG	ATG(D)	1,91	1,02	2,93
	CAT(RC(D))	1,66	0,93	2,59
	TAC(C(D))	1,02	1,91	2,93
	GTA(R(D))	0,93	1,66	2,59
TGA	TGA(D)	2,01	1,47	3,48
	TCA(RC(D))	1,85	1,55	3,40
	ACT(C(D))	1,47	2,01	3,48
	AGT(R(D))	1,55	1,85	3,40
TAG	TAG(D)	0,90	1,30	2,20
	CTA(RC(D))	1,00	1,42	2,43
	ATC(C(D))	1,30	0,90	2,20
	GAT(R(D))	1,42	1,00	2,43
TAA	TAA(D)	1,46	1,91	3,37
	TTA(RC(D))	1,55	1,93	3,48
	ATT(C(D))	1,91	1,46	3,37
	AAT(R(D))	1,93	1,55	3,48
AAC	AAC(D)	1,46	1,76	3,23
	GTT(RC(D))	1,38	1,83	3,21
	TTG(C(D))	1,76	1,46	3,23
	CAA(R(D))	1,83	1,38	3,21
AAG	AAG(D)	2,23	1,81	4,04
	CTT(RC(D))	1,83	2,33	4,17
	TTC(C(D))	1,81	2,23	4,04
	GAA(R(D))	2,33	1,83	4,17
ACA	ACA(D)	0,94	0,93	1,87
	TGT(RC(D))	0,93	0,94	1,87
	TGT(C(D))	0,93	0,94	1,87
	ACA(R(D))	0,94	0,93	1,87
ATA	ATA(D)	0,68	0,74	1,43
	TAT(RC(D))	0,74	0,68	1,43
	TAT(C(D))	0,74	0,68	1,43
	ATA(R(D))	0,68	0,68	1,37
AGA	AGA(D)	1,21	0,92	2,13
	TCT(RC(D))	0,92	1,21	2,13
	TCT(C(D))	0,92	1,21	2,13
	AGA(R(D))	1,21	0,92	2,13
AAA	AAA(D)	1,58	1,45	3,03
	TTT(RC(D))	1,45	1,58	3,03
	TTT(C(D))	1,45	1,58	3,03
	AAA(R(D))	1,58	1,45	3,03

Composition of DNA sequence in coding chromosome 18

Base	No.of bases	%
A	930813	27,92%
C	752959	22,58%
G	777327	23,31%
T	873072	26,19%
N	0	0,00%
Sum	3334171	100,00%

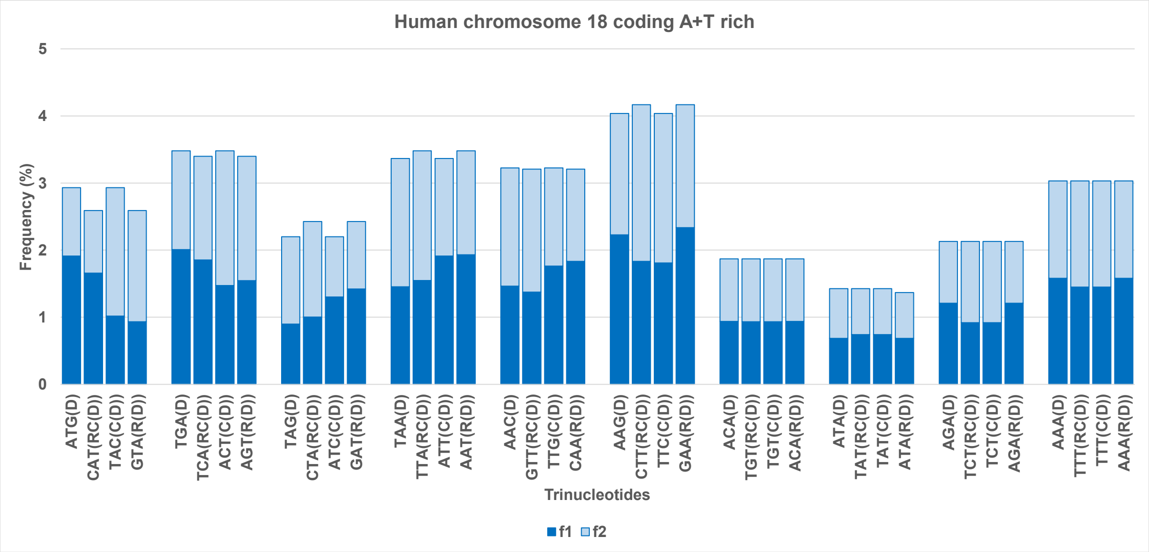

					C+G rich						
D	f	%	RC	f	%	C	f	%	R	f	%
**CGT**	221660,00	0,28	ACG	201055,00	0,25	GCA	1162736,00	1,45	TGC	1112082,00	1,39
**GTC**	722559,00	0,90	GAC	743809,00	0,93	CAG	1593812,00	1,99	CTG	1519584,00	1,90
**GCT**	1054781,00	1,32	AGC	1103886,00	1,38	CGA	158241,00	0,20	TCG	177655,00	0,22
**GCC**	825714,00	1,03	GGC	836750,00	1,04	CGG	201058,00	0,25	CCG	192770,00	0,24
**CCA**	1328725,00	1,66	TGG	1439494,00	1,80	GGT	865188,00	1,08	ACC	853753,00	1,07
**CCT**	1320719,00	1,65	AGG	1339232,00	1,67	GGA	1277846,00	1,60	TCC	1162744,00	1,45
**CAC**	1154534,00	1,44	GTG	1193202,00	1,49	GTG*			CAC*		
**CGC**	175055,00	0,22	GCG	184533,00	0,23	GCG*			CGC*		
**CTC**	1276654,00	1,59	GAG	1297351,00	1,62	GAG*			CTC*		
**CCC**	930193,00	1,16	GGG	928798,00	1,16	GGG*			CCC*		

Quadruplet		f1	f2	fu
CGT	CGT(D)	0,28	1,45	1,73
	ACG(RC(D))	0,25	1,39	1,64
	GCA(C(D))	1,45	0,28	1,73
	TGC(R(D))	1,39	0,25	1,64
GTC	GTC(D)	0,90	1,99	2,89
	GAC(RC(D))	0,93	1,90	2,83
	CAG(C(D))	1,99	0,90	2,89
	CTG(R(D))	1,90	0,93	2,83
GCT	GCT(D)	1,32	0,20	1,51
	AGC(RC(D))	1,38	0,22	1,60
	CGA(C(D))	0,20	1,32	1,51
	TCG(R(D))	0,22	1,38	1,60
GCC	GCC(D)	1,03	0,25	1,28
	GGC(RC(D))	1,04	0,24	1,29
	CGG(C(D))	0,25	1,03	1,28
	CCG(R(D))	0,24	1,04	1,29
CCA	CCA(D)	1,66	1,08	2,74
	TGG(RC(D))	1,80	1,07	2,86
	GGT(C(D))	1,08	1,66	2,74
	ACC(R(D))	1,07	1,80	2,86
CCT	CCT(D)	1,65	1,60	3,24
	AGG(RC(D))	1,67	1,45	3,12
	GGA(C(D))	1,60	1,65	3,24
	TCC(R(D))	1,45	1,67	3,12
CAC	CAC(D)	0,72	0,74	1,47
	GTG(RC(D))	0,74	0,72	1,47
	GTG(C(D))	0,74	0,72	1,47
	CAC(R(D))	0,72	0,74	1,47
CGC	CGC(D)	0,11	0,12	0,22
	GCG(RC(D))	0,12	0,11	0,22
	GCG(C(D))	0,12	0,11	0,22
	CGC(R(D))	0,11	0,12	0,22
CTC	CTC(D)	0,80	0,81	1,61
	GAG(RC(D))	0,81	0,80	1,61
	GAG(C(D))	0,81	0,80	1,61
	CTC(R(D))	0,80	0,81	1,61
CCC	CCC(D)	0,58	0,58	1,16
	GGG(RC(D))	0,58	0,58	1,16
	GGG(C(D))	0,58	0,58	1,16
	CCC(R(D))	0,58	0,58	1,16

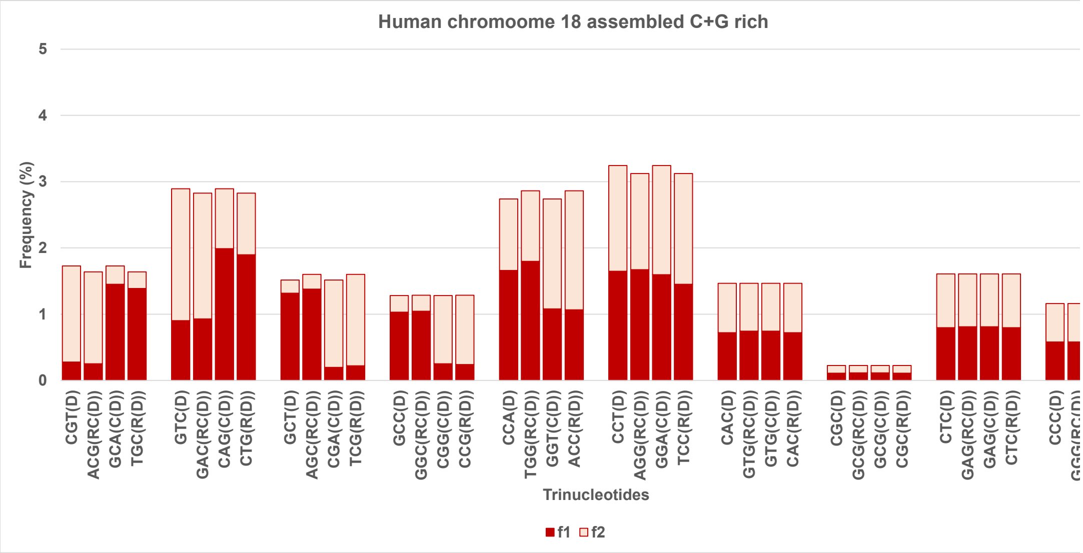

Human random chromomome 18

C+G rich											
**D**	**f**	**%**	**RC**	**f**	**%**	**C**	**f**	**%**	**R**	**f**	**%**
**CGT**	540,00	0,27	ACG	488,00	0,24	GCA	2808,00	1,41	TGC	2684,00	1,35
**GTC**	1804,00	0,90	GAC	1903,00	0,95	CAG	3929,00	1,97	CTG	3748,00	1,88
**GCT**	2542,00	1,27	AGC	2737,00	1,37	CGA	401,00	0,20	TCG	437,00	0,22
**GCC**	2008,00	1,01	GGC	1974,00	0,99	CGG	445,00	0,22	CCG	424,00	0,21
**CCA**	3324,00	1,67	TGG	3524,00	1,77	GGT	2112,00	1,06	ACC	2085,00	1,05
**CCT**	3235,00	1,62	AGG	3348,00	1,68	GGA	3231,00	1,62	TCC	2890,00	1,45
**CAC**	2788,00	1,40	GTG	2835,00	1,42	GTG*			CAC*		
**CGC**	385,00	0,19	GCG	422,00	0,21	GCG*			CGC*		
**CTC**	3114,00	1,56	GAG	3132,00	1,57	GAG*			CTC*		
**CCC**	2235,00	1,12	GGG	2184,00	1,10	GGG*			CCC*		

Quadruplet		f1	f2	fu
CGT	CGT(D)	0,27	1,41	1,68
	ACG(RC(D))	0,24	1,35	1,59
	GCA(C(D))	1,41	0,27	1,68
	TGC(R(D))	1,35	0,24	1,59
GTC	GTC(D)	0,90	1,97	2,88
	GAC(RC(D))	0,95	1,88	2,83
	CAG(C(D))	1,97	0,90	2,88
	CTG(R(D))	1,88	0,95	2,83
GCT	GCT(D)	1,27	0,20	1,48
	AGC(RC(D))	1,37	0,22	1,59
	CGA(C(D))	0,20	1,27	1,48
	TCG(R(D))	0,22	1,37	1,59
GCC	GCC(D)	1,01	0,22	1,23
	GGC(RC(D))	0,99	0,21	1,20
	CGG(C(D))	0,22	1,01	1,23
	CCG(R(D))	0,21	0,99	1,20
CCA	CCA(D)	1,67	1,06	2,73
	TGG(RC(D))	1,77	1,05	2,81
	GGT(C(D))	1,06	1,67	2,73
	ACC(R(D))	1,05	1,77	2,81
CCT	CCT(D)	1,62	1,62	3,24
	AGG(RC(D))	1,68	1,45	3,13
	GGA(C(D))	1,62	1,62	3,24
	TCC(R(D))	1,45	1,68	3,13
CAC	CAC(D)	0,70	0,71	1,41
	GTG(RC(D))	0,71	0,70	1,41
	GTG(C(D))	0,71	0,70	1,41
	CAC(R(D))	0,70	0,71	1,41
CGC	CGC(D)	0,10	0,11	0,20
	GCG(RC(D))	0,11	0,10	0,20
	GCG(C(D))	0,11	0,10	0,20
	CGC(R(D))	0,10	0,11	0,20
CTC	CTC(D)	0,78	0,79	1,57
	GAG(RC(D))	0,79	0,78	1,57
	GAG(C(D))	0,79	0,78	1,57
	CTC(R(D))	0,78	0,79	1,57
CCC	CCC(D)	0,56	0,55	1,11
	GGG(RC(D))	0,55	0,56	1,11
	GGG(C(D))	0,55	0,56	1,11
	CCC(R(D))	0,56	0,55	1,11

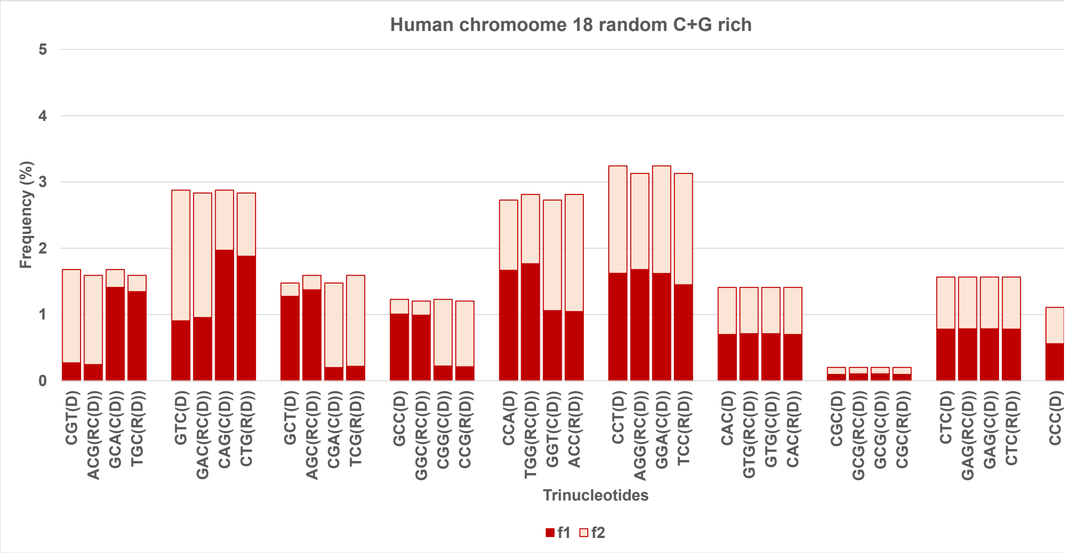

Human coding chromomome 18

C+G rich											
**D**	**f**	**%**	**RC**	**f**	**%**	**C**	**f**	**%**	**R**	**f**	**%**
**CGT**	15302,00	0,46	ACG	17109,00	0,51	GCA	55555,00	1,67	TGC	55018,00	1,65
**GTC**	34393,00	1,03	GAC	41607,00	1,25	CAG	80290,00	2,41	CTG	75193,00	2,26
**GCT**	53282,00	1,60	AGC	58260,00	1,75	CGA	17546,00	0,53	TCG	15668,00	0,47
**GCC**	53261,00	1,60	GGC	48890,00	1,47	CGG	25348,00	0,76	CCG	25350,00	0,76
**CCA**	65675,00	1,97	TGG	67348,00	2,02	GGT	36077,00	1,08	ACC	44468,00	1,33
**CCT**	60408,00	1,81	AGG	57657,00	1,73	GGA	65386,00	1,96	TCC	53704,00	1,61
**CAC**	48928,00	1,47	GTG	53732,00	1,61	GTG*			CAC*		
**CGC**	22393,00	0,67	GCG	22462,00	0,67	GCG*			CGC*		
**CTC**	54312,00	1,63	GAG	63668,00	1,91	GAG*			CTC*		
**CCC**	51311,00	1,54	GGG	46780,00	1,40	GGG*			CCC*		

Quadruplet		f1	f2	fu
CGT	CGT(D)	0,46	1,67	2,13
	ACG(RC(D))	0,51	1,65	2,16
	GCA(C(D))	1,67	0,46	2,13
	TGC(R(D))	1,65	0,51	2,16
GTC	GTC(D)	1,03	2,41	3,44
	GAC(RC(D))	1,25	2,26	3,50
	CAG(C(D))	2,41	1,03	3,44
	CTG(R(D))	2,26	1,25	3,50
GCT	GCT(D)	1,60	0,53	2,12
	AGC(RC(D))	1,75	0,47	2,22
	CGA(C(D))	0,53	1,60	2,12
	TCG(R(D))	0,47	1,75	2,22
GCC	GCC(D)	1,60	0,76	2,36
	GGC(RC(D))	1,47	0,76	2,23
	CGG(C(D))	0,76	1,60	2,36
	CCG(R(D))	0,76	1,47	2,23
CCA	CCA(D)	1,97	1,08	3,05
	TGG(RC(D))	2,02	1,33	3,35
	GGT(C(D))	1,08	1,97	3,05
	ACC(R(D))	1,33	2,02	3,35
CCT	CCT(D)	1,81	1,96	3,77
	AGG(RC(D))	1,73	1,61	3,34
	GGA(C(D))	1,96	1,81	3,77
	TCC(R(D))	1,61	1,73	3,34
CAC	CAC(D)	0,73	0,81	1,54
	GTG(RC(D))	0,81	0,73	1,54
	GTG(C(D))	0,81	0,73	1,54
	CAC(R(D))	0,73	0,81	1,54
CGC	CGC(D)	0,34	0,34	0,67
	GCG(RC(D))	0,34	0,34	0,67
	GCG(C(D))	0,34	0,34	0,67
	CGC(R(D))	0,34	0,34	0,67
CTC	CTC(D)	0,81	0,95	1,77
	GAG(RC(D))	0,95	0,81	1,77
	GAG(C(D))	0,95	0,81	1,77
	CTC(R(D))	0,81	0,95	1,77
CCC	CCC(D)	0,77	0,70	1,47
	GGG(RC(D))	0,70	0,77	1,47
	GGG(C(D))	0,70	0,77	1,47
	CCC(R(D))	0,77	0,70	1,47

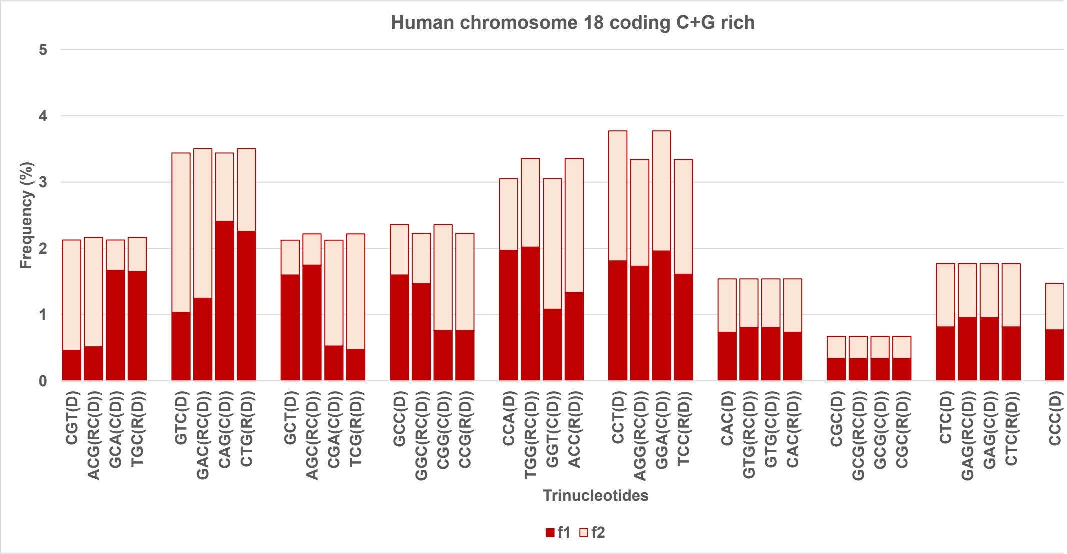

IIa

IIb

IIc

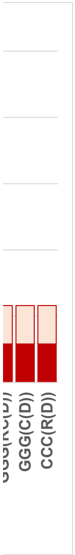

IIa

IIb

IIc

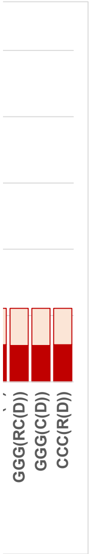

IIa

IIb

IIc

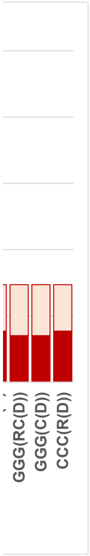
GGG(RC(D))
GGG(C(D))
CCC(R(D))

						A-T rich						
**D**	**f**	**%**	**RC**	**f**	**%**	**C**	**f**	**%**	**R**	**f**	**%**	
**ATG**	871447,00	1,49	CAT	880588,00	1,51	TAC	509753,00	0,87	GTA	535125,00	0,92	Ia
**TGA**	1069161,00	1,83	TCA	1102909,00	1,89	ACT	874193,00	1,50	AGT	862595,00	1,48	
**TAG**	612364,00	1,05	CTA	588732,00	1,01	ATC	697737,00	1,19	GAT	722824,00	1,24	
**TAA**	770229,00	1,32	TTA	764397,00	1,31	ATT	1006979,00	1,72	AAT	976842,00	1,67	Ib
**AAC**	758082,00	1,30	GTT	752132,00	1,29	TTG	1013421,00	1,73	CAA	955858,00	1,64	
**AAG**	977323,00	1,67	CTT	1019485,00	1,74	TTC	1000778,00	1,71	GAA	986161,00	1,69	
**ACA**	1070889,00	1,83	TGT	1107265,00	1,89	TGT*			ACA*			Ic
**ATA**	736375,00	1,26	TAT	732283,00	1,25	TAT*			ATA*			
**AGA**	1252047,00	2,14	TCT	1215733,00	2,08	TCT*			AGA*			
**AAA**	1767107,00	3,02	TTT	1833038,00	3,14	TTT*			AAA*			

Quadruplet		f1	f2	fu
ATG	ATG(D)	1,49	0,87	2,36
	CAT(RC(D))	1,51	0,92	2,42
	TAC(C(D))	0,87	1,49	2,36
	GTA(R(D))	0,92	1,51	2,42
TGA	TGA(D)	1,83	1,50	3,33
	TCA(RC(D))	1,89	1,48	3,36
	ACT(C(D))	1,50	1,83	3,33
	AGT(R(D))	1,48	1,89	3,36
TAG	TAG(D)	1,05	1,19	2,24
	CTA(RC(D))	1,01	1,24	2,24
	ATC(C(D))	1,19	1,05	2,24
	GAT(R(D))	1,24	1,01	2,24
TAA	TAA(D)	1,32	1,72	3,04
	TTA(RC(D))	1,31	1,67	2,98
	ATT(C(D))	1,72	1,32	3,04
	AAT(R(D))	1,67	1,31	2,98
AAC	AAC(D)	1,30	1,73	3,03
	GTT(RC(D))	1,29	1,64	2,92
	TTG(C(D))	1,73	1,30	3,03
	CAA(R(D))	1,64	1,29	2,92
AAG	AAG(D)	1,67	1,71	3,38
	CTT(RC(D))	1,74	1,69	3,43
	TTC(C(D))	1,71	1,67	3,38
	GAA(R(D))	1,69	1,74	3,43
ACA	ACA(D)	0,92	0,95	1,86
	TGT(RC(D))	0,95	0,92	1,86
	TGT(C(D))	0,95	0,92	1,86
	ACA(R(D))	0,92	0,95	1,86
ATA	ATA(D)	0,63	0,63	1,26
	TAT(RC(D))	0,63	0,63	1,26
	TAT(C(D))	0,63	0,63	1,26
	ATA(R(D))	0,63	0,63	1,26
AGA	AGA(D)	1,07	1,04	2,11
	TCT(RC(D))	1,04	1,07	2,11
	TCT(C(D))	1,04	1,07	2,11
	AGA(R(D))	1,07	1,04	2,11
AAA	AAA(D)	1,51	1,57	3,08
	TTT(RC(D))	1,57	1,51	3,08
	TTT(C(D))	1,57	1,51	3,08
	AAA(R(D))	1,51	1,57	3,08

Composition of DNA sequence in assembled chromosome 19

Base	No.of bases	%
A	15142293	25,83%
C	13954580	23,81%
G	14061132	23,99%
T	15282753	26,07%
N	176858	0,30%
Sum	58617616	100,00%

% of coding bases from complete assembled chromosome 1 (including N bases)

**18,04%**

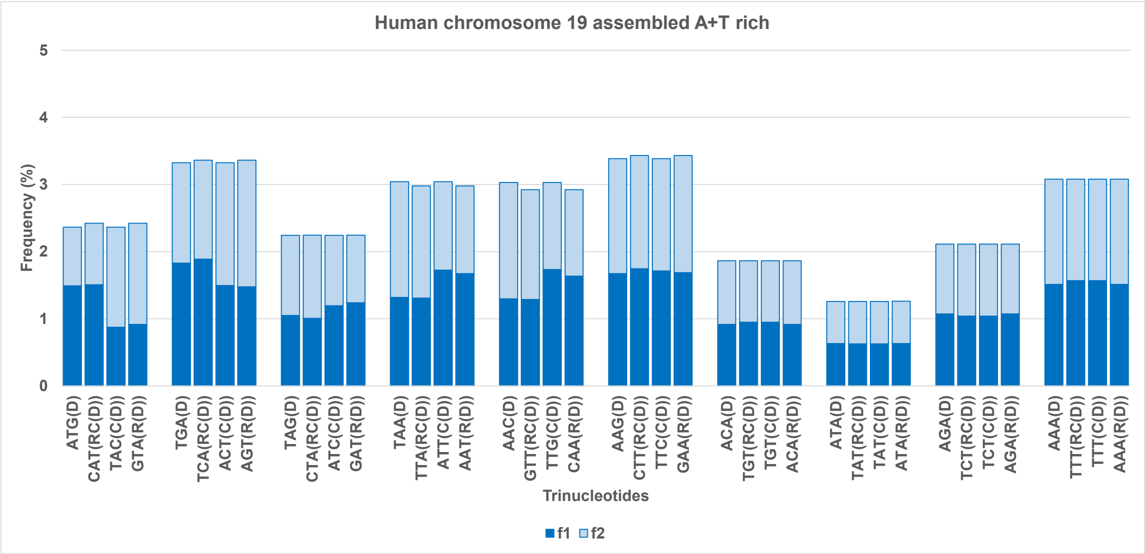

Human random chromomome 19

					A-T rich							
**D**	**f**	**%**	**RC**	**f**	**%**	**C**	**f**	**%**	**R**	**f**	**%**	
**ATG**	2760,00	1,46	CAT	2438,00	1,29	TAC	1428,00	0,75	GTA	1371,00	0,72	Ia
**TGA**	3165,00	1,67	TCA	3143,00	1,66	ACT	2413,00	1,27	AGT	2273,00	1,20	
**TAG**	1475,00	0,78	CTA	1565,00	0,83	ATC	1974,00	1,04	GAT	2272,00	1,20	
**TAA**	2184,00	1,15	TTA	1881,00	0,99	ATT	2358,00	1,24	AAT	2652,00	1,40	Ib
**AAC**	2231,00	1,18	GTT	1862,00	0,98	TTG	2337,00	1,23	CAA	2833,00	1,50	
**AAG**	3008,00	1,59	CTT	2524,00	1,33	TTC	2526,00	1,33	GAA	2874,00	1,52	
**ACA**	3461,00	1,83	TGT	2957,00	1,56	TGT*			ACA*			Ic
**ATA**	2061,00	1,09	TAT	1791,00	0,95	TAT*			ATA*			
**AGA**	3741,00	1,97	TCT	3212,00	1,70	TCT*			AGA*			
**AAA**	5032,00	2,66	TTT	3829,00	2,02	TTT*			AAA*			

Quadruplet		f1	f2	fu
ATG	ATG(D)	1,46	0,75	2,21
	CAT(RC(D))	1,29	0,72	2,01
	TAC(C(D))	0,75	1,46	2,21
	GTA(R(D))	0,72	1,29	2,01
TGA	TGA(D)	1,67	1,27	2,94
	TCA(RC(D))	1,66	1,20	2,86
	ACT(C(D))	1,27	1,67	2,94
	AGT(R(D))	1,20	1,66	2,86
TAG	TAG(D)	0,78	1,04	1,82
	CTA(RC(D))	0,83	1,20	2,03
	ATC(C(D))	1,04	0,78	1,82
	GAT(R(D))	1,20	0,83	2,03
TAA	TAA(D)	1,15	1,24	2,40
	TTA(RC(D))	0,99	1,40	2,39
	ATT(C(D))	1,24	1,15	2,40
	AAT(R(D))	1,40	0,99	2,39
AAC	AAC(D)	1,18	1,23	2,41
	GTT(RC(D))	0,98	1,50	2,48
	TTG(C(D))	1,23	1,18	2,41
	CAA(R(D))	1,50	0,98	2,48
AAG	AAG(D)	1,59	1,33	2,92
	CTT(RC(D))	1,33	1,52	2,85
	TTC(C(D))	1,33	1,59	2,92
	GAA(R(D))	1,52	1,33	2,85
ACA	ACA(D)	0,91	0,78	1,69
	TGT(RC(D))	0,78	0,91	1,69
	TGT(C(D))	0,78	0,91	1,69
	ACA(R(D))	0,91	0,78	1,69
ATA	ATA(D)	0,54	0,47	1,02
	TAT(RC(D))	0,47	0,54	1,02
	TAT(C(D))	0,47	0,54	1,02
	ATA(R(D))	0,54	0,54	1,09
AGA	AGA(D)	0,99	0,85	1,83
	TCT(RC(D))	0,85	0,99	1,83
	TCT(C(D))	0,85	0,99	1,83
	AGA(R(D))	0,99	0,85	1,83
AAA	AAA(D)	1,33	1,01	2,34
	TTT(RC(D))	1,01	1,33	2,34
	TTT(C(D))	1,01	1,33	2,34
	AAA(R(D))	1,33	1,01	2,34

Composition of DNA sequence in random chromosome 19

Base	No.of bases	%
A	46192	23,10%
C	49545	24,77%
G	51414	25,71%
T	42330	21,17%
N	10519	5,26%
Sum	200000	100,00%

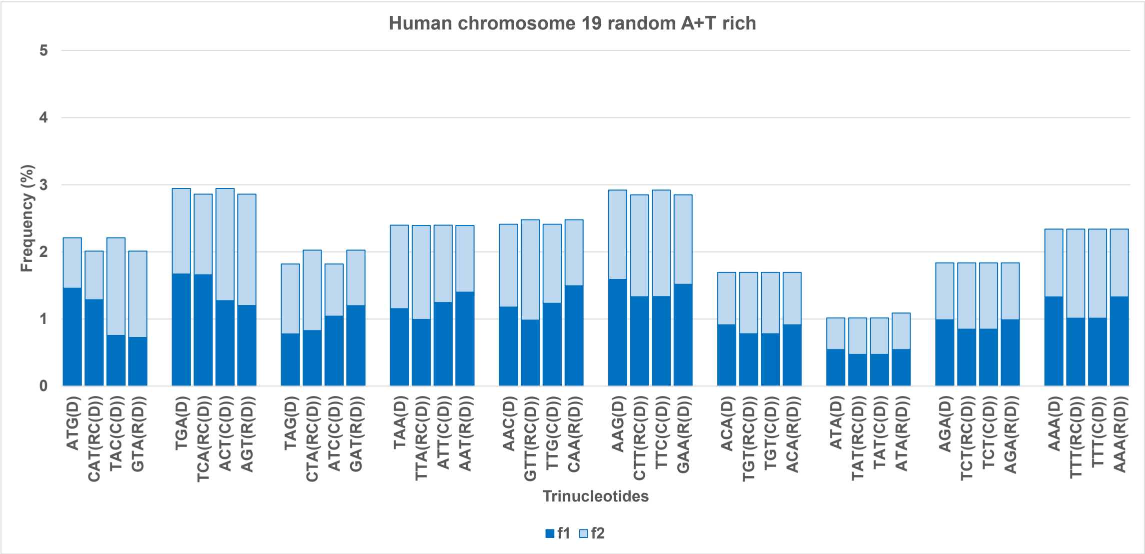

Human coding chromomome 19

A-T rich

D	f	%	RC	f	%	C	f	%	R	f	%	
**ATG**	160061,00	1,51	CAT	152583,00	1,44	TAC	90354,00	0,85	GTA	65926,00	0,62	Ia
**TGA**	173087,00	1,64	TCA	179749,00	1,70	ACT	129173,00	1,22	AGT	125506,00	1,19	
**TAG**	55436,00	0,52	CTA	82728,00	0,78	ATC	120714,00	1,14	GAT	110653,00	1,05	
**TAA**	75356,00	0,71	TTA	76022,00	0,72	ATT	100929,00	0,95	AAT	114938,00	1,09	Ib
**AAC**	123407,00	1,17	GTT	91198,00	0,86	TTG	117348,00	1,11	CAA	160486,00	1,52	
**AAG**	191627,00	1,81	CTT	160198,00	1,52	TTC	158955,00	1,50	GAA	194130,00	1,84	
**ACA**	168812,00	1,60	TGT	163926,00	1,55	TGT*			ACA*			Ic
**ATA**	74049,00	0,70	TAT	77579,00	0,73	TAT*			ATA*			
**AGA**	222516,00	2,10	TCT	162859,00	1,54	TCT*			AGA*			
**AAA**	182108,00	1,72	TTT	155409,00	1,47	TTT*			AAA*			

Quadruplet		f1	f2	fu
ATG	ATG(D)	1,51	0,85	2,37
	CAT(RC(D))	1,44	0,62	2,07
	TAC(C(D))	0,85	1,51	2,37
	GTA(R(D))	0,62	1,44	2,07
TGA	TGA(D)	1,64	1,22	2,86
	TCA(RC(D))	1,70	1,19	2,89
	ACT(C(D))	1,22	1,64	2,86
	AGT(R(D))	1,19	1,70	2,89
TAG	TAG(D)	0,52	1,14	1,67
	CTA(RC(D))	0,78	1,05	1,83
	ATC(C(D))	1,14	0,52	1,67
	GAT(R(D))	1,05	0,78	1,83
TAA	TAA(D)	0,71	0,95	1,67
	TTA(RC(D))	0,72	1,09	1,81
	ATT(C(D))	0,95	0,71	1,67
	AAT(R(D))	1,09	0,72	1,81
AAC	AAC(D)	1,17	1,11	2,28
	GTT(RC(D))	0,86	1,52	2,38
	TTG(C(D))	1,11	1,17	2,28
	CAA(R(D))	1,52	0,86	2,38
AAG	AAG(D)	1,81	1,50	3,32
	CTT(RC(D))	1,52	1,84	3,35
	TTC(C(D))	1,50	1,81	3,32
	GAA(R(D))	1,84	1,52	3,35
ACA	ACA(D)	0,80	0,78	1,57
	TGT(RC(D))	0,78	0,80	1,57
	TGT(C(D))	0,78	0,80	1,57
	ACA(R(D))	0,80	0,78	1,57
ATA	ATA(D)	0,35	0,37	0,72
	TAT(RC(D))	0,37	0,35	0,72
	TAT(C(D))	0,37	0,35	0,72
	ATA(R(D))	0,35	0,35	0,70
AGA	AGA(D)	1,05	0,77	1,82
	TCT(RC(D))	0,77	1,05	1,82
	TCT(C(D))	0,77	1,05	1,82
	AGA(R(D))	1,05	0,77	1,82
AAA	AAA(D)	0,86	0,73	1,60
	TTT(RC(D))	0,73	0,86	1,60
	TTT(C(D))	0,73	0,86	1,60
	AAA(R(D))	0,86	0,73	1,60

Composition of DNA sequence in coding chromosome 19

Base	No.of bases	%
A	2438858	23,07%
C	2984779	28,23%
G	2925446	27,67%
T	2224211	21,04%
N	0	0,00%
Sum	10573294	100,00%

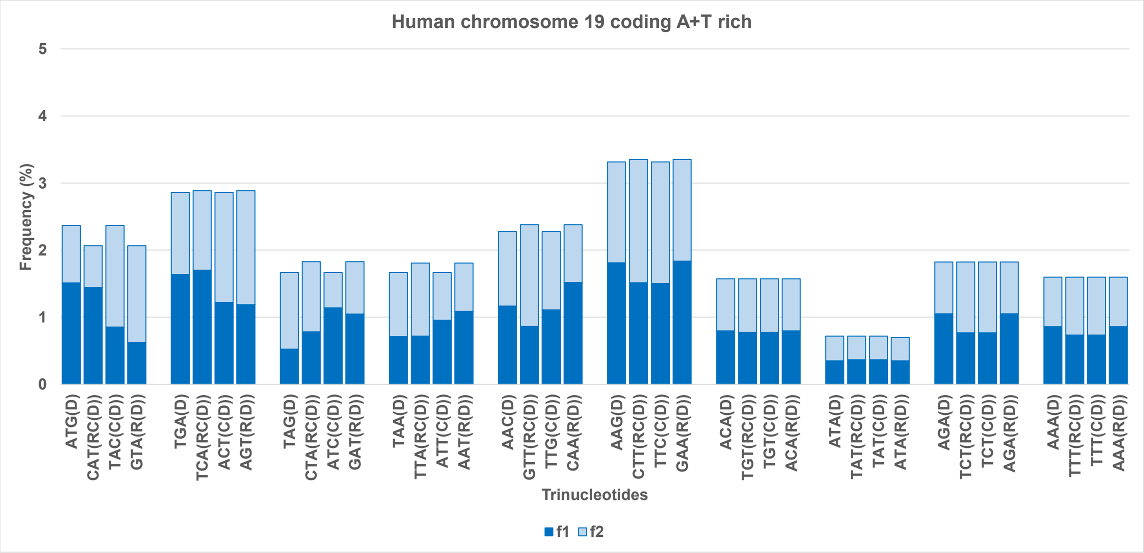

						C+G rich					
**D**	**f**	**%**	**RC**	**f**	**%**	**C**	**f**	**%**	**R**	**f**	**%**
**CGT**	239774,00	0,41	ACG	228681,00	0,39	GCA	943580,00	1,61	TGC	940378,00	1,61
**GTC**	628811,00	1,08	GAC	642214,00	1,10	CAG	1497674,00	2,56	CTG	1453736,00	2,49
**GCT**	945867,00	1,62	AGC	953998,00	1,63	CGA	213160,00	0,36	TCG	223534,00	0,38
**GCC**	1064499,00	1,82	GGC	1065305,00	1,82	CGG	348048,00	0,60	CCG	343032,00	0,59
**CCA**	1292344,00	2,21	TGG	1327545,00	2,27	GGT	812178,00	1,39	ACC	811888,00	1,39
**CCT**	1300972,00	2,23	AGG	1296104,00	2,22	GGA	1094214,00	1,87	TCC	1059961,00	1,81
**CAC**	1075602,00	1,84	GTG	1105745,00	1,89	GTG*			CAC*		
**CGC**	304638,00	0,52	GCG	310373,00	0,53	GCG*			CGC*		
**CTC**	1274813,00	2,18	GAG	1277382,00	2,19	GAG*			CTC*		
**CCC**	1166122,00	2,00	GGG	1174718,00	2,01	GGG*			CCC*		

Quadruplet		f1	f2	fu
CGT	CGT(D)	0,41	1,61	2,02
	ACG(RC(D))	0,39	1,61	2,00
	GCA(C(D))	1,61	0,41	2,02
	TGC(R(D))	1,61	0,39	2,00
GTC	GTC(D)	1,08	2,56	3,64
	GAC(RC(D))	1,10	2,49	3,59
	CAG(C(D))	2,56	1,08	3,64
	CTG(R(D))	2,49	1,10	3,59
GCT	GCT(D)	1,62	0,36	1,98
	AGC(RC(D))	1,63	0,38	2,01
	CGA(C(D))	0,36	1,62	1,98
	TCG(R(D))	0,38	1,63	2,01
GCC	GCC(D)	1,82	0,60	2,42
	GGC(RC(D))	1,82	0,59	2,41
	CGG(C(D))	0,60	1,82	2,42
	CCG(R(D))	0,59	1,82	2,41
CCA	CCA(D)	2,21	1,39	3,60
	TGG(RC(D))	2,27	1,39	3,66
	GGT(C(D))	1,39	2,21	3,60
	ACC(R(D))	1,39	2,27	3,66
CCT	CCT(D)	2,23	1,87	4,10
	AGG(RC(D))	2,22	1,81	4,03
	GGA(C(D))	1,87	2,23	4,10
	TCC(R(D))	1,81	2,22	4,03
CAC	CAC(D)	0,92	0,95	1,87
	GTG(RC(D))	0,95	0,92	1,87
	GTG(C(D))	0,95	0,92	1,87
	CAC(R(D))	0,92	0,95	1,87
CGC	CGC(D)	0,26	0,27	0,53
	GCG(RC(D))	0,27	0,26	0,53
	GCG(C(D))	0,27	0,26	0,53
	CGC(R(D))	0,26	0,27	0,53
CTC	CTC(D)	1,09	1,09	2,18
	GAG(RC(D))	1,09	1,09	2,18
	GAG(C(D))	1,09	1,09	2,18
	CTC(R(D))	1,09	1,09	2,18
CCC	CCC(D)	1,00	1,01	2,00
	GGG(RC(D))	1,01	1,00	2,00
	GGG(C(D))	1,01	1,00	2,00
	CCC(R(D))	1,00	1,01	2,00

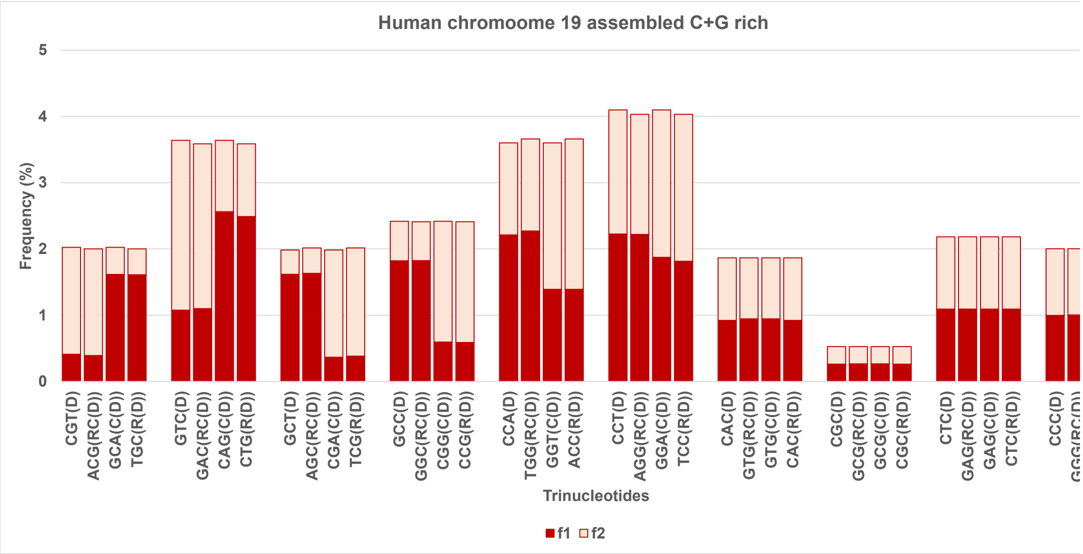

Human random chromomome 19

					C+G rich						
**D**	**f**	**%**	**RC**	**f**	**%**	**C**	**f**	**%**	**R**	**f**	**%**
**CGT**	1114,00	0,59	ACG	1195,00	0,63	GCA	3245,00	1,71	TGC	2956,00	1,56
**GTC**	2262,00	1,19	GAC	2396,00	1,26	CAG	5112,00	2,70	CTG	5058,00	2,67
**GCT**	3230,00	1,70	AGC	3282,00	1,73	CGA	966,00	0,51	TCG	917,00	0,48
**GCC**	4370,00	2,31	GGC	4612,00	2,43	CGG	2057,00	1,09	CCG	2020,00	1,07
**CCA**	4473,00	2,36	TGG	4844,00	2,56	GGT	2919,00	1,54	ACC	2925,00	1,54
**CCT**	4486,00	2,37	AGG	4826,00	2,55	GGA	4196,00	2,21	TCC	3684,00	1,94
**CAC**	3939,00	2,08	GTG	3767,00	1,99	GTG*			CAC*		
**CGC**	1623,00	0,86	GCG	1628,00	0,86	GCG*			CGC*		
**CTC**	4194,00	2,21	GAG	4527,00	2,39	GAG*			CTC*		
**CCC**	5143,00	2,71	GGG	5882,00	3,10	GGG*			CCC*		

Quadruplet		f1	f2	fu
CGT	CGT(D)	0,59	1,71	2,30
	ACG(RC(D))	0,63	1,56	2,19
	GCA(C(D))	1,71	0,59	2,30
	TGC(R(D))	1,56	0,63	2,19
GTC	GTC(D)	1,19	2,70	3,89
	GAC(RC(D))	1,26	2,67	3,93
	CAG(C(D))	2,70	1,19	3,89
	CTG(R(D))	2,67	1,26	3,93
GCT	GCT(D)	1,70	0,51	2,21
	AGC(RC(D))	1,73	0,48	2,22
	CGA(C(D))	0,51	1,70	2,21
	TCG(R(D))	0,48	1,73	2,22
GCC	GCC(D)	2,31	1,09	3,39
	GGC(RC(D))	2,43	1,07	3,50
	CGG(C(D))	1,09	2,31	3,39
	CCG(R(D))	1,07	2,43	3,50
CCA	CCA(D)	2,36	1,54	3,90
	TGG(RC(D))	2,56	1,54	4,10
	GGT(C(D))	1,54	2,36	3,90
	ACC(R(D))	1,54	2,56	4,10
CCT	CCT(D)	2,37	2,21	4,58
	AGG(RC(D))	2,55	1,94	4,49
	GGA(C(D))	2,21	2,37	4,58
	TCC(R(D))	1,94	2,55	4,49
CAC	CAC(D)	1,04	0,99	2,03
	GTG(RC(D))	0,99	1,04	2,03
	GTG(C(D))	0,99	1,04	2,03
	CAC(R(D))	1,04	0,99	2,03
CGC	CGC(D)	0,43	0,43	0,86
	GCG(RC(D))	0,43	0,43	0,86
	GCG(C(D))	0,43	0,43	0,86
	CGC(R(D))	0,43	0,43	0,86
CTC	CTC(D)	1,11	1,19	2,30
	GAG(RC(D))	1,19	1,11	2,30
	GAG(C(D))	1,19	1,11	2,30
	CTC(R(D))	1,11	1,19	2,30
CCC	CCC(D)	1,36	1,55	2,91
	GGG(RC(D))	1,55	1,36	2,91
	GGG(C(D))	1,55	1,36	2,91
	CCC(R(D))	1,36	1,55	2,91

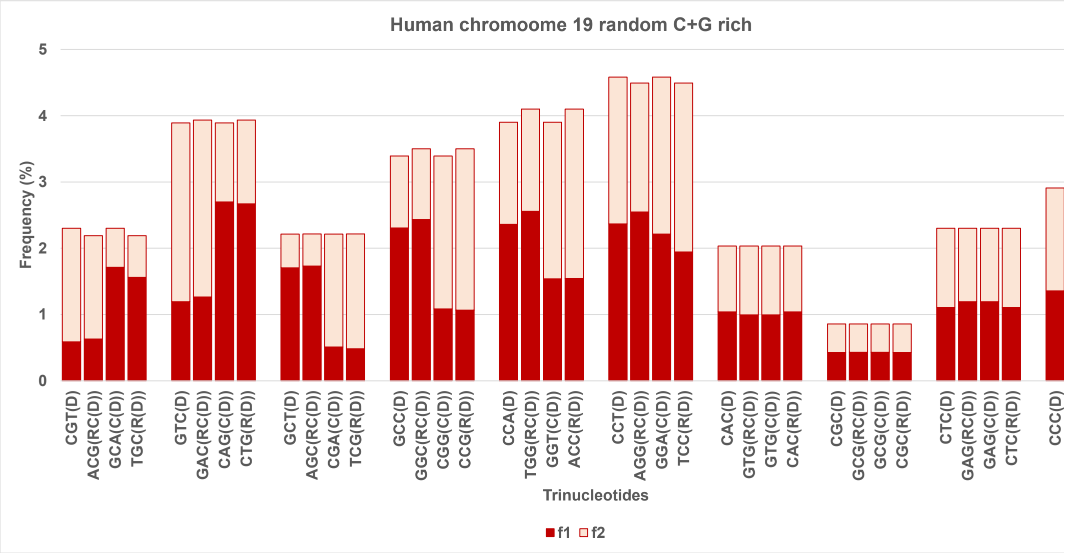

Human coding chromomome 19

C+G rich

D	f	%	RC	f	%	C	f	%	R	f	%
**CGT**	69664,00	0,66	ACG	80510,00	0,76	GCA	188737,00	1,79	TGC	195737,00	1,85
**GTC**	124683,00	1,18	GAC	163300,00	1,54	CAG	290147,00	2,74	CTG	312882,00	2,96
**GCT**	204813,00	1,94	AGC	219426,00	2,08	CGA	81125,00	0,77	TCG	69084,00	0,65
**GCC**	282791,00	2,67	GGC	259540,00	2,45	CGG	146303,00	1,38	CCG	149136,00	1,41
**CCA**	258482,00	2,44	TGG	266460,00	2,52	GGT	131630,00	1,24	ACC	191130,00	1,81
**CCT**	273153,00	2,58	AGG	233951,00	2,21	GGA	255545,00	2,42	TCC	206850,00	1,96
**CAC**	192564,00	1,82	GTG	208919,00	1,98	GTG*			CAC*		
**CGC**	129933,00	1,23	GCG	128295,00	1,21	GCG*			CGC*		
**CTC**	214190,00	2,03	GAG	264189,00	2,50	GAG*			CTC*		
**CCC**	311205,00	2,94	GGG	251096,00	2,37	GGG*			CCC*		

Quadruplet		f1	f2	fu
CGT	CGT(D)	0,66	1,79	2,44
	ACG(RC(D))	0,76	1,85	2,61
	GCA(C(D))	1,79	0,66	2,44
	TGC(R(D))	1,85	0,76	2,61
GTC	GTC(D)	1,18	2,74	3,92
	GAC(RC(D))	1,54	2,96	4,50
	CAG(C(D))	2,74	1,18	3,92
	CTG(R(D))	2,96	1,54	4,50
GCT	GCT(D)	1,94	0,77	2,70
	AGC(RC(D))	2,08	0,65	2,73
	CGA(C(D))	0,77	1,94	2,70
	TCG(R(D))	0,65	2,08	2,73
GCC	GCC(D)	2,67	1,38	4,06
	GGC(RC(D))	2,45	1,41	3,87
	CGG(C(D))	1,38	2,67	4,06
	CCG(R(D))	1,41	2,45	3,87
CCA	CCA(D)	2,44	1,24	3,69
	TGG(RC(D))	2,52	1,81	4,33
	GGT(C(D))	1,24	2,44	3,69
	ACC(R(D))	1,81	2,52	4,33
CCT	CCT(D)	2,58	2,42	5,00
	AGG(RC(D))	2,21	1,96	4,17
	GGA(C(D))	2,42	2,58	5,00
	TCC(R(D))	1,96	2,21	4,17
CAC	CAC(D)	0,91	0,99	1,90
	GTG(RC(D))	0,99	0,91	1,90
	GTG(C(D))	0,99	0,91	1,90
	CAC(R(D))	0,91	0,99	1,90
CGC	CGC(D)	0,61	0,61	1,22
	GCG(RC(D))	0,61	0,61	1,22
	GCG(C(D))	0,61	0,61	1,22
	CGC(R(D))	0,61	0,61	1,22
CTC	CTC(D)	1,01	1,25	2,26
	GAG(RC(D))	1,25	1,01	2,26
	GAG(C(D))	1,25	1,01	2,26
	CTC(R(D))	1,01	1,25	2,26
CCC	CCC(D)	1,47	1,19	2,66
	GGG(RC(D))	1,19	1,47	2,66
	GGG(C(D))	1,19	1,47	2,66
	CCC(R(D))	1,47	1,19	2,66

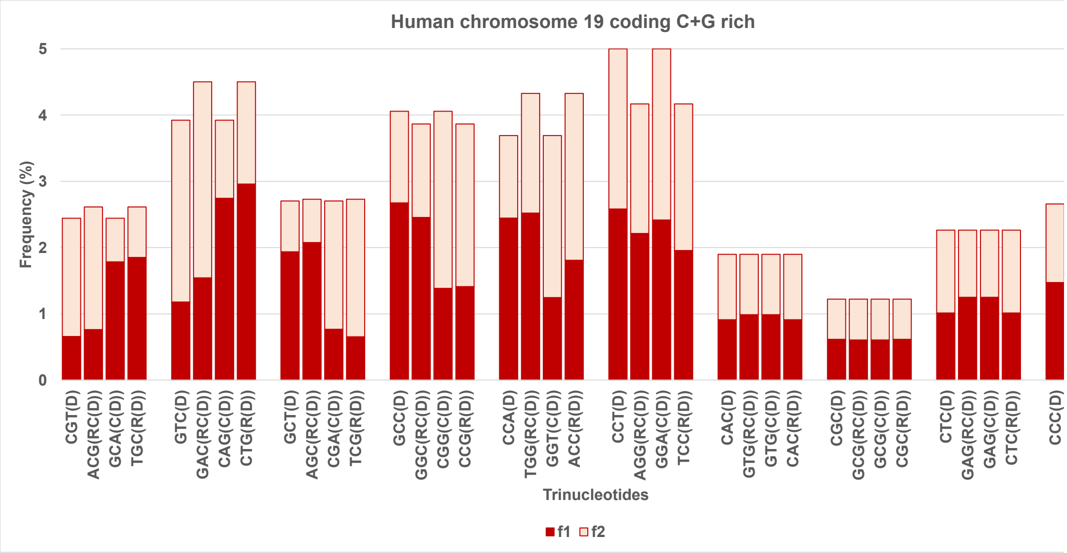

IIa

IIb

IIc

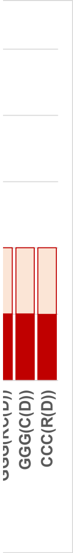

IIa

IIb

IIc

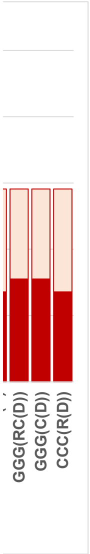

IIa

IIb

IIc

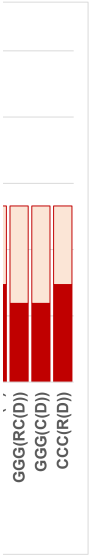
GGG(RC(D))
GGG(C(D))
CCC(R(D))

						A-T rich						
**D**	**f**	**%**	**RC**	**f**	**%**	**C**	**f**	**%**	**R**	**f**	**%**	
**ATG**	1127469,00	1,76	CAT	1131512,00	1,77	TAC	634098,00	0,99	GTA	648503,00	1,01	Ia
**TGA**	1256597,00	1,97	TCA	1235564,00	1,93	ACT	1001475,00	1,57	AGT	996767,00	1,56	
**TAG**	758242,00	1,19	CTA	746641,00	1,17	ATC	815200,00	1,27	GAT	840613,00	1,31	
**TAA**	1075720,00	1,68	TTA	1074557,00	1,68	ATT	1366919,00	2,14	AAT	1335821,00	2,09	Ib
**AAC**	908702,00	1,42	GTT	902012,00	1,41	TTG	1232393,00	1,93	CAA	1131552,00	1,77	
**AAG**	1224711,00	1,92	CTT	1273244,00	1,99	TTC	1235220,00	1,93	GAA	1261962,00	1,97	
**ACA**	1251030,00	1,96	TGT	1282865,00	2,01	TGT*			ACA*			Ic
**ATA**	1058650,00	1,66	TAT	1060293,00	1,66	TAT*			ATA*			
**AGA**	1403118,00	2,19	TCT	1418033,00	2,22	TCT*			AGA*			
**AAA**	2155995,00	3,37	TTT	2253241,00	3,52	TTT*			AAA*			

Quadruplet		f1	f2	fu
ATG	ATG(D)	1,76	0,99	2,75
	CAT(RC(D))	1,77	1,01	2,78
	TAC(C(D))	0,99	1,76	2,75
	GTA(R(D))	1,01	1,77	2,78
TGA	TGA(D)	1,97	1,57	3,53
	TCA(RC(D))	1,93	1,56	3,49
	ACT(C(D))	1,57	1,97	3,53
	AGT(R(D))	1,56	1,93	3,49
TAG	TAG(D)	1,19	1,27	2,46
	CTA(RC(D))	1,17	1,31	2,48
	ATC(C(D))	1,27	1,19	2,46
	GAT(R(D))	1,31	1,17	2,48
TAA	TAA(D)	1,68	2,14	3,82
	TTA(RC(D))	1,68	2,09	3,77
	ATT(C(D))	2,14	1,68	3,82
	AAT(R(D))	2,09	1,68	3,77
AAC	AAC(D)	1,42	1,93	3,35
	GTT(RC(D))	1,41	1,77	3,18
	TTG(C(D))	1,93	1,42	3,35
	CAA(R(D))	1,77	1,41	3,18
AAG	AAG(D)	1,92	1,93	3,85
	CTT(RC(D))	1,99	1,97	3,96
	TTC(C(D))	1,93	1,92	3,85
	GAA(R(D))	1,97	1,99	3,96
ACA	ACA(D)	0,98	1,00	1,98
	TGT(RC(D))	1,00	0,98	1,98
	TGT(C(D))	1,00	0,98	1,98
	ACA(R(D))	0,98	1,00	1,98
ATA	ATA(D)	0,83	0,83	1,66
	TAT(RC(D))	0,83	0,83	1,66
	TAT(C(D))	0,83	0,83	1,66
	ATA(R(D))	0,83	0,83	1,66
AGA	AGA(D)	1,10	1,11	2,21
	TCT(RC(D))	1,11	1,10	2,21
	TCT(C(D))	1,11	1,10	2,21
	AGA(R(D))	1,10	1,11	2,21
AAA	AAA(D)	1,69	1,76	3,45
	TTT(RC(D))	1,76	1,69	3,45
	TTT(C(D))	1,76	1,69	3,45
	AAA(R(D))	1,69	1,76	3,45

Composition of DNA sequence in assembled chromosome 20

Base	No.of bases	%
A	17867246	27,73%
C	13916133	21,59%
G	14094472	21,87%
T	18066406	28,03%
N	499910	0,78%
Sum	64444167	100,00%

% of coding bases from complete assembled chromosome 1 (including N bases)

**6,64%**

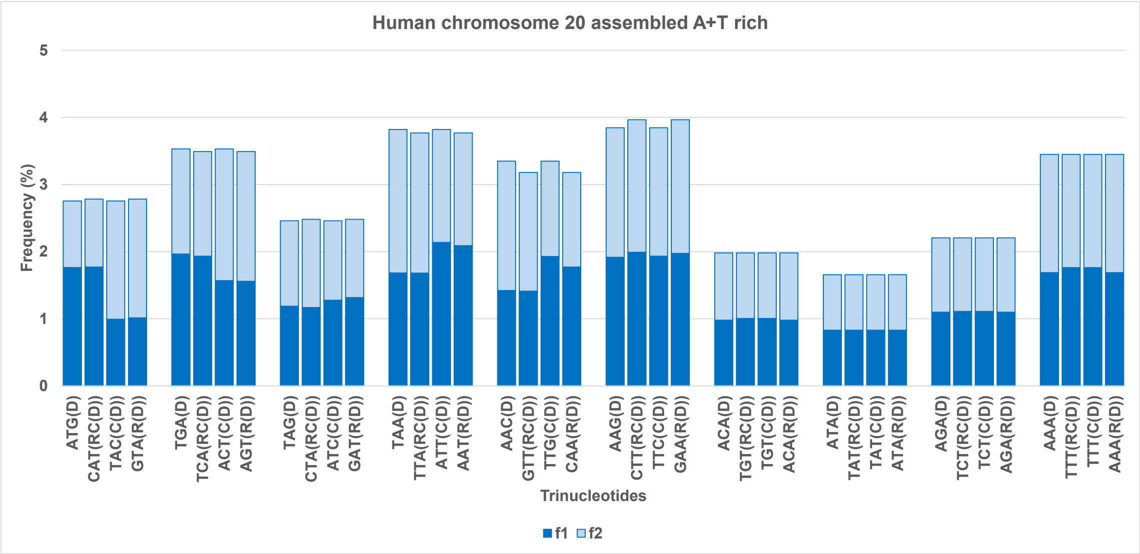

Human random chromomome 20

A-T rich

D	f	%	RC	f	%	C	f	%	R	f	%	
**ATG**	3169,00	1,68	CAT	3365,00	1,78	TAC	2084,00	1,10	GTA	1844,00	0,98	Ia
**TGA**	3425,00	1,81	TCA	3618,00	1,91	ACT	2998,00	1,59	AGT	2855,00	1,51	
**TAG**	2122,00	1,12	CTA	2306,00	1,22	ATC	2474,00	1,31	GAT	2272,00	1,20	
**TAA**	3103,00	1,64	TTA	2949,00	1,56	ATT	3680,00	1,95	AAT	3896,00	2,06	Ib
**AAC**	2657,00	1,41	GTT	2323,00	1,23	TTG	3119,00	1,65	CAA	3584,00	1,90	
**AAG**	3596,00	1,90	CTT	3486,00	1,84	TTC	3421,00	1,81	GAA	3462,00	1,83	
**ACA**	3808,00	2,01	TGT	3374,00	1,79	TGT*			ACA*			Ic
**ATA**	3081,00	1,63	TAT	2871,00	1,52	TAT*			ATA*			
**AGA**	4061,00	2,15	TCT	4010,00	2,12	TCT*			AGA*			
**AAA**	6602,00	3,49	TTT	5774,00	3,06	TTT*			AAA*			

Quadruplet		f1	f2	fu
ATG	ATG(D)	1,68	1,10	2,78
	CAT(RC(D))	1,78	0,98	2,76
	TAC(C(D))	1,10	1,68	2,78
	GTA(R(D))	0,98	1,78	2,76
TGA	TGA(D)	1,81	1,59	3,40
	TCA(RC(D))	1,91	1,51	3,42
	ACT(C(D))	1,59	1,81	3,40
	AGT(R(D))	1,51	1,91	3,42
TAG	TAG(D)	1,12	1,31	2,43
	CTA(RC(D))	1,22	1,20	2,42
	ATC(C(D))	1,31	1,12	2,43
	GAT(R(D))	1,20	1,22	2,42
TAA	TAA(D)	1,64	1,95	3,59
	TTA(RC(D))	1,56	2,06	3,62
	ATT(C(D))	1,95	1,64	3,59
	AAT(R(D))	2,06	1,56	3,62
AAC	AAC(D)	1,41	1,65	3,06
	GTT(RC(D))	1,23	1,90	3,13
	TTG(C(D))	1,65	1,41	3,06
	CAA(R(D))	1,90	1,23	3,13
AAG	AAG(D)	1,90	1,81	3,71
	CTT(RC(D))	1,84	1,83	3,68
	TTC(C(D))	1,81	1,90	3,71
	GAA(R(D))	1,83	1,84	3,68
ACA	ACA(D)	1,01	0,89	1,90
	TGT(RC(D))	0,89	1,01	1,90
	TGT(C(D))	0,89	1,01	1,90
	ACA(R(D))	1,01	0,89	1,90
ATA	ATA(D)	0,82	0,76	1,57
	TAT(RC(D))	0,76	0,82	1,57
	TAT(C(D))	0,76	0,82	1,57
	ATA(R(D))	0,82	0,82	1,63
AGA	AGA(D)	1,07	1,06	2,14
	TCT(RC(D))	1,06	1,07	2,14
	TCT(C(D))	1,06	1,07	2,14
	AGA(R(D))	1,07	1,06	2,14
AAA	AAA(D)	1,75	1,53	3,27
	TTT(RC(D))	1,53	1,75	3,27
	TTT(C(D))	1,53	1,75	3,27
	AAA(R(D))	1,75	1,53	3,27

Composition of DNA sequence in random chromosome 20

Base	No.of bases	%
A	52865	26,43%
C	43622	21,81%
G	41801	20,90%
T	50712	25,36%
N	11000	5,50%
Sum	200000	100,00%

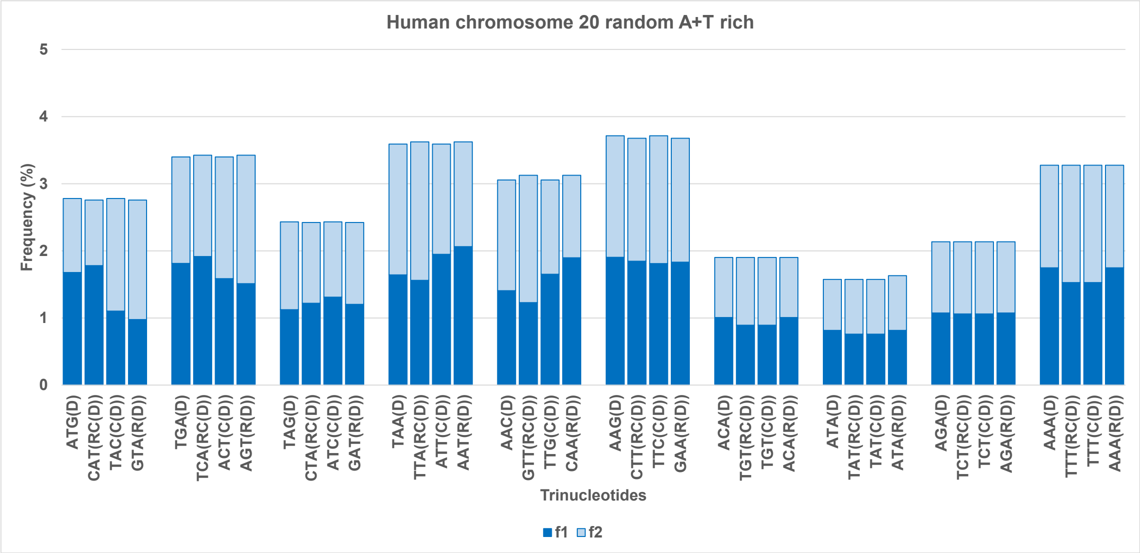

Human coding chromomome 20

A-T rich

D	f	%	RC	f	%	C	f	%	R	f	%	
**ATG**	68714,00	1,61	CAT	63695,00	1,49	TAC	36440,00	0,85	GTA	31108,00	0,73	Ia
**TGA**	78158,00	1,83	TCA	71199,00	1,66	ACT	56146,00	1,31	AGT	57610,00	1,35	
**TAG**	29325,00	0,69	CTA	36563,00	0,85	ATC	50468,00	1,18	GAT	51901,00	1,21	
**TAA**	40280,00	0,94	TTA	41042,00	0,96	ATT	54051,00	1,26	AAT	54969,00	1,28	Ib
**AAC**	53221,00	1,24	GTT	46854,00	1,09	TTG	63537,00	1,48	CAA	69995,00	1,64	
**AAG**	87163,00	2,04	CTT	71313,00	1,67	TTC	67639,00	1,58	GAA	85078,00	1,99	
**ACA**	71032,00	1,66	TGT	73029,00	1,71	TGT*			ACA*			Ic
**ATA**	36072,00	0,84	TAT	38740,00	0,90	TAT*			ATA*			
**AGA**	93260,00	2,18	TCT	73326,00	1,71	TCT*			AGA*			
**AAA**	93225,00	2,18	TTT	86391,00	2,02	TTT*			AAA*			

Quadruplet		f1	f2	fu
ATG	ATG(D)	1,61	0,85	2,46
	CAT(RC(D))	1,49	0,73	2,21
	TAC(C(D))	0,85	1,61	2,46
	GTA(R(D))	0,73	1,49	2,21
TGA	TGA(D)	1,83	1,31	3,14
	TCA(RC(D))	1,66	1,35	3,01
	ACT(C(D))	1,31	1,83	3,14
	AGT(R(D))	1,35	1,66	3,01
TAG	TAG(D)	0,69	1,18	1,86
	CTA(RC(D))	0,85	1,21	2,07
	ATC(C(D))	1,18	0,69	1,86
	GAT(R(D))	1,21	0,85	2,07
TAA	TAA(D)	0,94	1,26	2,20
	TTA(RC(D))	0,96	1,28	2,24
	ATT(C(D))	1,26	0,94	2,20
	AAT(R(D))	1,28	0,96	2,24
AAC	AAC(D)	1,24	1,48	2,73
	GTT(RC(D))	1,09	1,64	2,73
	TTG(C(D))	1,48	1,24	2,73
	CAA(R(D))	1,64	1,09	2,73
AAG	AAG(D)	2,04	1,58	3,62
	CTT(RC(D))	1,67	1,99	3,65
	TTC(C(D))	1,58	2,04	3,62
	GAA(R(D))	1,99	1,67	3,65
ACA	ACA(D)	0,83	0,85	1,68
	TGT(RC(D))	0,85	0,83	1,68
	TGT(C(D))	0,85	0,83	1,68
	ACA(R(D))	0,83	0,85	1,68
ATA	ATA(D)	0,42	0,45	0,87
	TAT(RC(D))	0,45	0,42	0,87
	TAT(C(D))	0,45	0,42	0,87
	ATA(R(D))	0,42	0,42	0,84
AGA	AGA(D)	1,09	0,86	1,95
	TCT(RC(D))	0,86	1,09	1,95
	TCT(C(D))	0,86	1,09	1,95
	AGA(R(D))	1,09	0,86	1,95
AAA	AAA(D)	1,09	1,01	2,10
	TTT(RC(D))	1,01	1,09	2,10
	TTT(C(D))	1,01	1,09	2,10
	AAA(R(D))	1,09	1,01	2,10

Composition of DNA sequence in coding chromosome 20

Base	No.of bases	%
A	1051660	24,57%
C	1122721	26,23%
G	1121997	26,21%
T	984541	23,00%
N	0	0,00%
Sum	4280919	100,00%

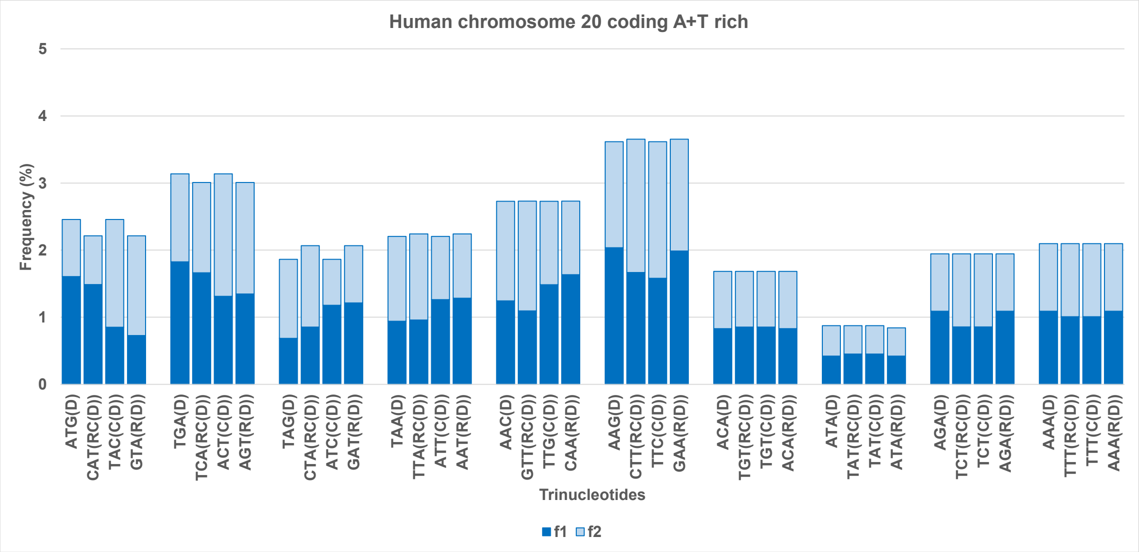

						C+G rich					
**D**	**f**	**%**	**RC**	**f**	**%**	**C**	**f**	**%**	**R**	**f**	**%**
**CGT**	193560,00	0,30	ACG	191564,00	0,30	GCA	1013704,00	1,59	TGC	989652,00	1,55
**GTC**	641998,00	1,00	GAC	643004,00	1,01	CAG	1464650,00	2,29	CTG	1422618,00	2,22
**GCT**	969200,00	1,52	AGC	993109,00	1,55	CGA	161149,00	0,25	TCG	170430,00	0,27
**GCC**	889717,00	1,39	GGC	891747,00	1,39	CGG	227793,00	0,36	CCG	218627,00	0,34
**CCA**	1266533,00	1,98	TGG	1326590,00	2,07	GGT	792537,00	1,24	ACC	780852,00	1,22
**CCT**	1248272,00	1,95	AGG	1255815,00	1,96	GGA	1125912,00	1,76	TCC	1062864,00	1,66
**CAC**	1039118,00	1,63	GTG	1073219,00	1,68	GTG*			CAC*		
**CGC**	190974,00	0,30	GCG	192856,00	0,30	GCG*			CGC*		
**CTC**	1194479,00	1,87	GAG	1201186,00	1,88	GAG*			CTC*		
**CCC**	1005374,00	1,57	GGG	1006257,00	1,57	GGG*			CCC*		

Quadruplet		f1	f2	fu
CGT	CGT(D)	0,30	1,59	1,89
	ACG(RC(D))	0,30	1,55	1,85
	GCA(C(D))	1,59	0,30	1,89
	TGC(R(D))	1,55	0,30	1,85
GTC	GTC(D)	1,00	2,29	3,29
	GAC(RC(D))	1,01	2,22	3,23
	CAG(C(D))	2,29	1,00	3,29
	CTG(R(D))	2,22	1,01	3,23
GCT	GCT(D)	1,52	0,25	1,77
	AGC(RC(D))	1,55	0,27	1,82
	CGA(C(D))	0,25	1,52	1,77
	TCG(R(D))	0,27	1,55	1,82
GCC	GCC(D)	1,39	0,36	1,75
	GGC(RC(D))	1,39	0,34	1,74
	CGG(C(D))	0,36	1,39	1,75
	CCG(R(D))	0,34	1,39	1,74
CCA	CCA(D)	1,98	1,24	3,22
	TGG(RC(D))	2,07	1,22	3,30
	GGT(C(D))	1,24	1,98	3,22
	ACC(R(D))	1,22	2,07	3,30
CCT	CCT(D)	1,95	1,76	3,71
	AGG(RC(D))	1,96	1,66	3,63
	GGA(C(D))	1,76	1,95	3,71
	TCC(R(D))	1,66	1,96	3,63
CAC	CAC(D)	0,81	0,84	1,65
	GTG(RC(D))	0,84	0,81	1,65
	GTG(C(D))	0,84	0,81	1,65
	CAC(R(D))	0,81	0,84	1,65
CGC	CGC(D)	0,15	0,15	0,30
	GCG(RC(D))	0,15	0,15	0,30
	GCG(C(D))	0,15	0,15	0,30
	CGC(R(D))	0,15	0,15	0,30
CTC	CTC(D)	0,93	0,94	1,87
	GAG(RC(D))	0,94	0,93	1,87
	GAG(C(D))	0,94	0,93	1,87
	CTC(R(D))	0,93	0,94	1,87
CCC	CCC(D)	0,79	0,79	1,57
	GGG(RC(D))	0,79	0,79	1,57
	GGG(C(D))	0,79	0,79	1,57
	CCC(R(D))	0,79	0,79	1,57

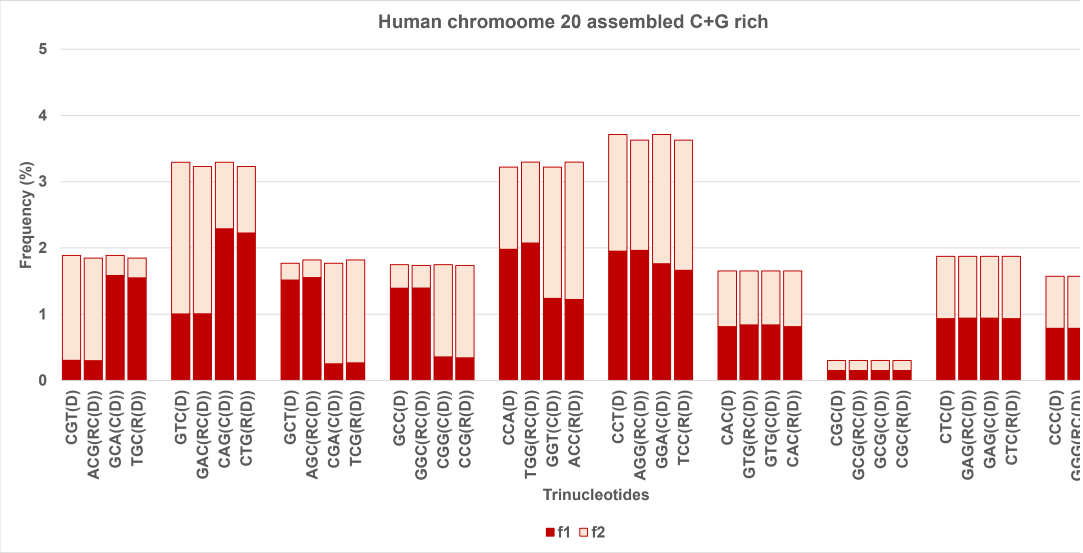

Human random chromomome 20

C+G rich

D	f	%	RC	f	%	C	f	%	R	f	%
**CGT**	562,00	0,30	ACG	623,00	0,33	GCA	2951,00	1,56	TGC	2953,00	1,56
**GTC**	1917,00	1,01	GAC	1912,00	1,01	CAG	4336,00	2,29	CTG	4247,00	2,25
**GCT**	2842,00	1,50	AGC	2971,00	1,57	CGA	552,00	0,29	TCG	527,00	0,28
**GCC**	3038,00	1,61	GGC	2870,00	1,52	CGG	821,00	0,43	CCG	822,00	0,43
**CCA**	4230,00	2,24	TGG	3850,00	2,04	GGT	2360,00	1,25	ACC	2546,00	1,35
**CCT**	4043,00	2,14	AGG	3847,00	2,04	GGA	3288,00	1,74	TCC	3512,00	1,86
**CAC**	3322,00	1,76	GTG	3067,00	1,62	GTG*			CAC*		
**CGC**	801,00	0,42	GCG	764,00	0,40	GCG*			CGC*		
**CTC**	3854,00	2,04	GAG	3680,00	1,95	GAG*			CTC*		
**CCC**	3288,00	1,74	GGG	3211,00	1,70	GGG*			CCC*		

Quadruplet		f1	f2	fu
CGT	CGT(D)	0,30	1,56	1,86
	ACG(RC(D))	0,33	1,56	1,89
	GCA(C(D))	1,56	0,30	1,86
	TGC(R(D))	1,56	0,33	1,89
GTC	GTC(D)	1,01	2,29	3,31
	GAC(RC(D))	1,01	2,25	3,26
	CAG(C(D))	2,29	1,01	3,31
	CTG(R(D))	2,25	1,01	3,26
GCT	GCT(D)	1,50	0,29	1,80
	AGC(RC(D))	1,57	0,28	1,85
	CGA(C(D))	0,29	1,50	1,80
	TCG(R(D))	0,28	1,57	1,85
GCC	GCC(D)	1,61	0,43	2,04
	GGC(RC(D))	1,52	0,43	1,95
	CGG(C(D))	0,43	1,61	2,04
	CCG(R(D))	0,43	1,52	1,95
CCA	CCA(D)	2,24	1,25	3,49
	TGG(RC(D))	2,04	1,35	3,38
	GGT(C(D))	1,25	2,24	3,49
	ACC(R(D))	1,35	2,04	3,38
CCT	CCT(D)	2,14	1,74	3,88
	AGG(RC(D))	2,04	1,86	3,89
	GGA(C(D))	1,74	2,14	3,88
	TCC(R(D))	1,86	2,04	3,89
CAC	CAC(D)	0,88	0,81	1,69
	GTG(RC(D))	0,81	0,88	1,69
	GTG(C(D))	0,81	0,88	1,69
	CAC(R(D))	0,88	0,81	1,69
CGC	CGC(D)	0,21	0,20	0,41
	GCG(RC(D))	0,20	0,21	0,41
	GCG(C(D))	0,20	0,21	0,41
	CGC(R(D))	0,21	0,20	0,41
CTC	CTC(D)	1,02	0,97	1,99
	GAG(RC(D))	0,97	1,02	1,99
	GAG(C(D))	0,97	1,02	1,99
	CTC(R(D))	1,02	0,97	1,99
CCC	CCC(D)	0,87	0,85	1,72
	GGG(RC(D))	0,85	0,87	1,72
	GGG(C(D))	0,85	0,87	1,72
	CCC(R(D))	0,87	0,85	1,72

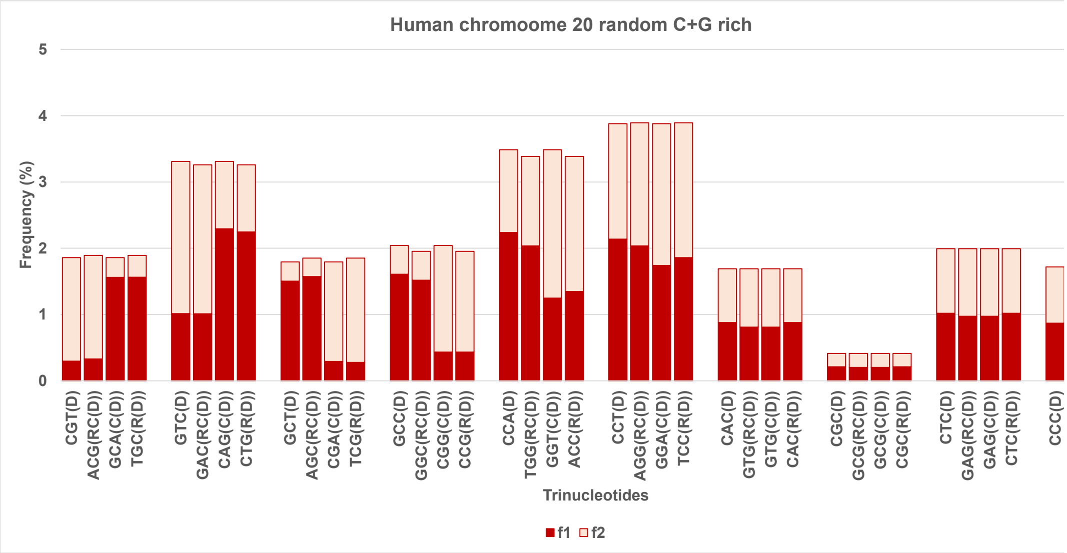

Human coding chromomome 20

C+G rich

D	f	%	RC	f	%	C	f	%	R	f	%
**CGT**	23292,00	0,54	ACG	26547,00	0,62	GCA	79164,00	1,85	TGC	81338,00	1,90
**GTC**	48777,00	1,14	GAC	60075,00	1,40	CAG	119070,00	2,78	CTG	120768,00	2,82
**GCT**	82577,00	1,93	AGC	89548,00	2,09	CGA	27043,00	0,63	TCG	23829,00	0,56
**GCC**	99465,00	2,32	GGC	89942,00	2,10	CGG	45709,00	1,07	CCG	46046,00	1,08
**CCA**	103309,00	2,41	TGG	99248,00	2,32	GGT	51562,00	1,20	ACC	67955,00	1,59
**CCT**	99084,00	2,31	AGG	91679,00	2,14	GGA	95132,00	2,22	TCC	81020,00	1,89
**CAC**	71944,00	1,68	GTG	78754,00	1,84	GTG*			CAC*		
**CGC**	39840,00	0,93	GCG	39462,00	0,92	GCG*			CGC*		
**CTC**	82490,00	1,93	GAG	96539,00	2,26	GAG*			CTC*		
**CCC**	102558,00	2,40	GGG	85607,00	2,00	GGG*			CCC*		

Quadruplet		f1	f2	fu
CGT	CGT(D)	0,54	1,85	2,39
	ACG(RC(D))	0,62	1,90	2,52
	GCA(C(D))	1,85	0,54	2,39
	TGC(R(D))	1,90	0,62	2,52
GTC	GTC(D)	1,14	2,78	3,92
	GAC(RC(D))	1,40	2,82	4,22
	CAG(C(D))	2,78	1,14	3,92
	CTG(R(D))	2,82	1,40	4,22
GCT	GCT(D)	1,93	0,63	2,56
	AGC(RC(D))	2,09	0,56	2,65
	CGA(C(D))	0,63	1,93	2,56
	TCG(R(D))	0,56	2,09	2,65
GCC	GCC(D)	2,32	1,07	3,39
	GGC(RC(D))	2,10	1,08	3,18
	CGG(C(D))	1,07	2,32	3,39
	CCG(R(D))	1,08	2,10	3,18
CCA	CCA(D)	2,41	1,20	3,62
	TGG(RC(D))	2,32	1,59	3,91
	GGT(C(D))	1,20	2,41	3,62
	ACC(R(D))	1,59	2,32	3,91
CCT	CCT(D)	2,31	2,22	4,54
	AGG(RC(D))	2,14	1,89	4,03
	GGA(C(D))	2,22	2,31	4,54
	TCC(R(D))	1,89	2,14	4,03
CAC	CAC(D)	0,84	0,92	1,76
	GTG(RC(D))	0,92	0,84	1,76
	GTG(C(D))	0,92	0,84	1,76
	CAC(R(D))	0,84	0,92	1,76
CGC	CGC(D)	0,47	0,46	0,93
	GCG(RC(D))	0,46	0,47	0,93
	GCG(C(D))	0,46	0,47	0,93
	CGC(R(D))	0,47	0,46	0,93
CTC	CTC(D)	0,96	1,13	2,09
	GAG(RC(D))	1,13	0,96	2,09
	GAG(C(D))	1,13	0,96	2,09
	CTC(R(D))	0,96	1,13	2,09
CCC	CCC(D)	1,20	1,00	2,20
	GGG(RC(D))	1,00	1,20	2,20
	GGG(C(D))	1,00	1,20	2,20
	CCC(R(D))	1,20	1,00	2,20

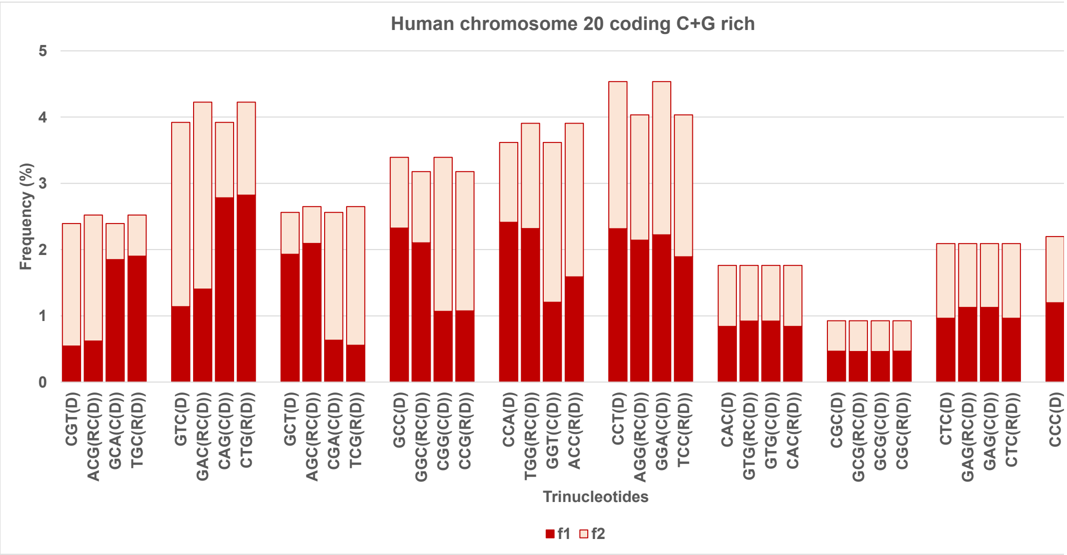

IIa

IIb

IIc

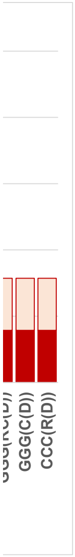

IIa

IIb

IIc

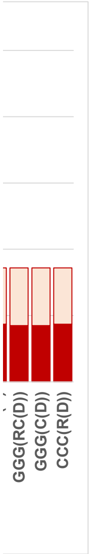

IIa

IIb

IIc

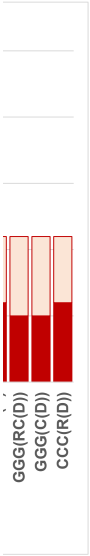
GGG(RC(D))
GGG(C(D))
CCC(R(D))

A-T rich												
**D**	**f**	**%**	**RC**	**f**	**%**	**C**	**f**	**%**	**R**	**f**	**%**	
**ATG**	719565,00	1,79	CAT	749235,00	1,87	TAC	433556,00	1,08	GTA	440653,00	1,10	Ia
**TGA**	795176,00	1,98	TCA	782568,00	1,95	ACT	633553,00	1,58	AGT	638600,00	1,59	
**TAG**	500305,00	1,25	CTA	494063,00	1,23	ATC	530068,00	1,32	GAT	541663,00	1,35	
**TAA**	808551,00	2,02	TTA	795935,00	1,99	ATT	1010116,00	2,52	AAT	980611,00	2,45	Ib
**AAC**	605695,00	1,51	GTT	586607,00	1,46	TTG	783263,00	1,95	CAA	737155,00	1,84	
**AAG**	787766,00	1,97	CTT	806378,00	2,01	TTC	823902,00	2,06	GAA	828366,00	2,07	
**ACA**	832184,00	2,08	TGT	825116,00	2,06	TGT*			ACA*			Ic
**ATA**	832560,00	2,08	TAT	820798,00	2,05	TAT*			ATA*			
**AGA**	895605,00	2,23	TCT	902456,00	2,25	TCT*			AGA*			
**AAA**	1541041,00	3,84	TTT	1565770,00	3,91	TTT*			AAA*			

Quadruplet		f1	f2	fu
ATG	ATG(D)	1,79	1,08	2,88
	CAT(RC(D))	1,87	1,10	2,97
	TAC(C(D))	1,08	1,79	2,88
	GTA(R(D))	1,10	1,87	2,97
TGA	TGA(D)	1,98	1,58	3,56
	TCA(RC(D))	1,95	1,59	3,55
	ACT(C(D))	1,58	1,98	3,56
	AGT(R(D))	1,59	1,95	3,55
TAG	TAG(D)	1,25	1,32	2,57
	CTA(RC(D))	1,23	1,35	2,58
	ATC(C(D))	1,32	1,25	2,57
	GAT(R(D))	1,35	1,23	2,58
TAA	TAA(D)	2,02	2,52	4,54
	TTA(RC(D))	1,99	2,45	4,43
	ATT(C(D))	2,52	2,02	4,54
	AAT(R(D))	2,45	1,99	4,43
AAC	AAC(D)	1,51	1,95	3,46
	GTT(RC(D))	1,46	1,84	3,30
	TTG(C(D))	1,95	1,51	3,46
	CAA(R(D))	1,84	1,46	3,30
AAG	AAG(D)	1,97	2,06	4,02
	CTT(RC(D))	2,01	2,07	4,08
	TTC(C(D))	2,06	1,97	4,02
	GAA(R(D))	2,07	2,01	4,08
ACA	ACA(D)	1,04	1,03	2,07
	TGT(RC(D))	1,03	1,04	2,07
	TGT(C(D))	1,03	1,04	2,07
	ACA(R(D))	1,04	1,03	2,07
ATA	ATA(D)	1,04	1,02	2,06
	TAT(RC(D))	1,02	1,04	2,06
	TAT(C(D))	1,02	1,04	2,06
	ATA(R(D))	1,04	1,04	2,08
AGA	AGA(D)	1,12	1,13	2,24
	TCT(RC(D))	1,13	1,12	2,24
	TCT(C(D))	1,13	1,12	2,24
	AGA(R(D))	1,12	1,13	2,24
AAA	AAA(D)	1,92	1,95	3,87
	TTT(RC(D))	1,95	1,92	3,87
	TTT(C(D))	1,95	1,92	3,87
	AAA(R(D))	1,92	1,95	3,87

Composition of DNA sequence in assembled chromosome 21

Base	No.of bases	%
A	11820664	25,31%
C	8185244	17,52%
G	8226381	17,61%
T	11856330	25,38%
N	6621364	14,18%
Sum	46709983	100,00%

% of coding bases from complete assembled chromosome 1 (including N bases)

**4,15%**

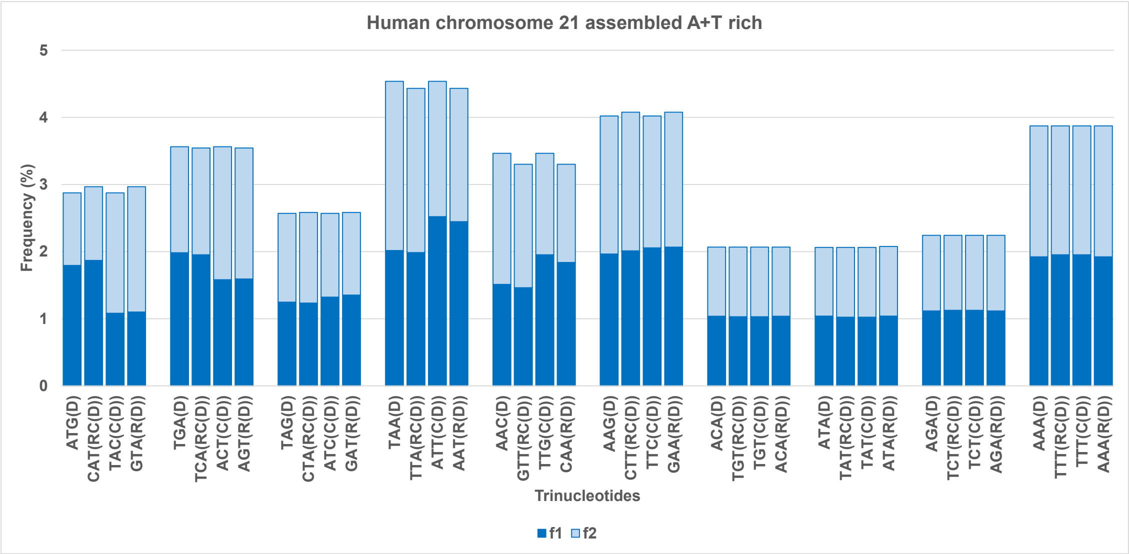

Human random chromomome 21

A-T rich												
**D**	**f**	**%**	**RC**	**f**	**%**	**C**	**f**	**%**	**R**	**f**	**%**	
**ATG**	3207,00	1,66	CAT	3844,00	1,99	TAC	2045,00	1,06	GTA	2184,00	1,13	Ia
**TGA**	4188,00	2,17	TCA	3799,00	1,97	ACT	3203,00	1,66	AGT	3399,00	1,76	
**TAG**	2664,00	1,38	CTA	2655,00	1,37	ATC	2772,00	1,44	GAT	2970,00	1,54	
**TAA**	3825,00	1,98	TTA	3527,00	1,83	ATT	5402,00	2,80	AAT	4736,00	2,45	Ib
**AAC**	3555,00	1,84	GTT	2997,00	1,55	TTG	4717,00	2,44	CAA	3463,00	1,79	
**AAG**	3987,00	2,06	CTT	4501,00	2,33	TTC	4657,00	2,41	GAA	4990,00	2,58	
**ACA**	4334,00	2,24	TGT	3966,00	2,05	TGT*			ACA*			Ic
**ATA**	4444,00	2,30	TAT	4276,00	2,21	TAT*			ATA*			
**AGA**	4784,00	2,48	TCT	4968,00	2,57	TCT*			AGA*			
**AAA**	8230,00	4,26	TTT	8816,00	4,57	TTT*			AAA*			

Quadruplet		f1	f2	fu
ATG	ATG(D)	1,66	1,06	2,72
	CAT(RC(D))	1,99	1,13	3,12
	TAC(C(D))	1,06	1,66	2,72
	GTA(R(D))	1,13	1,99	3,12
TGA	TGA(D)	2,17	1,66	3,83
	TCA(RC(D))	1,97	1,76	3,73
	ACT(C(D))	1,66	2,17	3,83
	AGT(R(D))	1,76	1,97	3,73
TAG	TAG(D)	1,38	1,44	2,82
	CTA(RC(D))	1,37	1,54	2,91
	ATC(C(D))	1,44	1,38	2,82
	GAT(R(D))	1,54	1,37	2,91
TAA	TAA(D)	1,98	2,80	4,78
	TTA(RC(D))	1,83	2,45	4,28
	ATT(C(D))	2,80	1,98	4,78
	AAT(R(D))	2,45	1,83	4,28
AAC	AAC(D)	1,84	2,44	4,28
	GTT(RC(D))	1,55	1,79	3,35
	TTG(C(D))	2,44	1,84	4,28
	CAA(R(D))	1,79	1,55	3,35
AAG	AAG(D)	2,06	2,41	4,48
	CTT(RC(D))	2,33	2,58	4,91
	TTC(C(D))	2,41	2,06	4,48
	GAA(R(D))	2,58	2,33	4,91
ACA	ACA(D)	1,12	1,03	2,15
	TGT(RC(D))	1,03	1,12	2,15
	TGT(C(D))	1,03	1,12	2,15
	ACA(R(D))	1,12	1,03	2,15
ATA	ATA(D)	1,15	1,11	2,26
	TAT(RC(D))	1,11	1,15	2,26
	TAT(C(D))	1,11	1,15	2,26
	ATA(R(D))	1,15	1,15	2,30
AGA	AGA(D)	1,24	1,29	2,53
	TCT(RC(D))	1,29	1,24	2,53
	TCT(C(D))	1,29	1,24	2,53
	AGA(R(D))	1,24	1,29	2,53
AAA	AAA(D)	2,13	2,28	4,41
	TTT(RC(D))	2,28	2,13	4,41
	TTT(C(D))	2,28	2,13	4,41
	AAA(R(D))	2,13	2,28	4,41

Composition of DNA sequence in random chromosome 21

Base	No.of bases	%
A	59716	29,86%
C	35795	17,90%
G	37401	18,70%
T	60206	30,10%
N	6882	3,44%
Sum	200000	100,00%

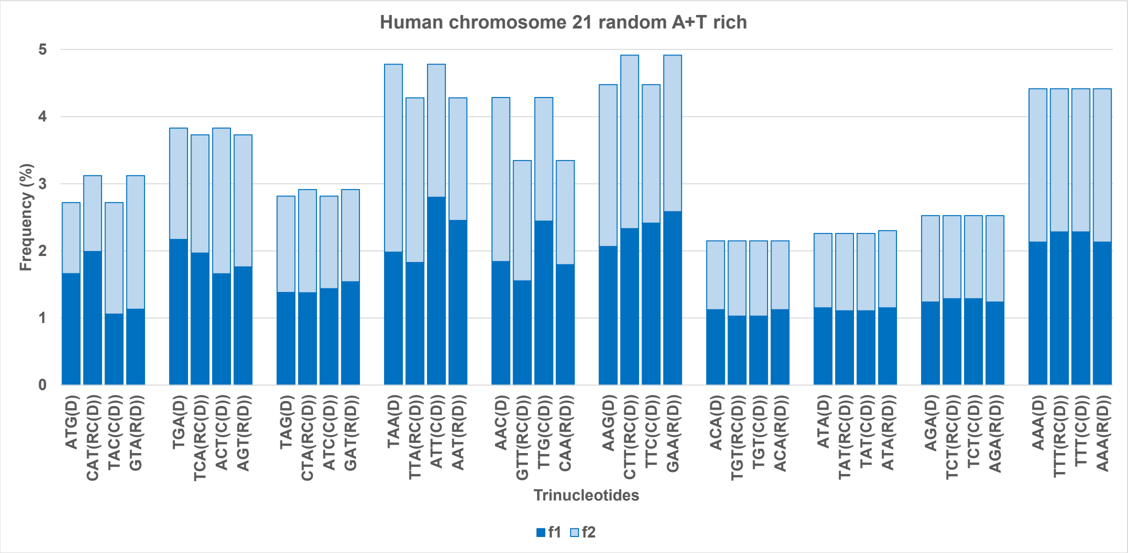

Human coding chromomome 21

A-T rich

D	f	%	RC	f	%	C	f	%	R	f	%	
**ATG**	32594,00	1,68	CAT	29614,00	1,53	TAC	17820,00	0,92	GTA	15651,00	0,81	Ia
**TGA**	37162,00	1,92	TCA	33417,00	1,72	ACT	26155,00	1,35	AGT	27655,00	1,43	
**TAG**	14861,00	0,77	CTA	17294,00	0,89	ATC	23694,00	1,22	GAT	25414,00	1,31	
**TAA**	22823,00	1,18	TTA	24131,00	1,25	ATT	30498,00	1,57	AAT	30186,00	1,56	Ib
**AAC**	25687,00	1,33	GTT	24561,00	1,27	TTG	30798,00	1,59	CAA	32070,00	1,66	
**AAG**	41241,00	2,13	CTT	32318,00	1,67	TTC	32448,00	1,67	GAA	42221,00	2,18	
**ACA**	33193,00	1,71	TGT	35391,00	1,83	TGT*			ACA*			Ic
**ATA**	20929,00	1,08	TAT	22501,00	1,16	TAT*			ATA*			
**AGA**	44424,00	2,29	TCT	34135,00	1,76	TCT*			AGA*			
**AAA**	50279,00	2,60	TTT	46635,00	2,41	TTT*			AAA*			

Quadruplet		f1	f2	fu
ATG	ATG(D)	1,68	0,92	2,60
	CAT(RC(D))	1,53	0,81	2,34
	TAC(C(D))	0,92	1,68	2,60
	GTA(R(D))	0,81	1,53	2,34
TGA	TGA(D)	1,92	1,35	3,27
	TCA(RC(D))	1,72	1,43	3,15
	ACT(C(D))	1,35	1,92	3,27
	AGT(R(D))	1,43	1,72	3,15
TAG	TAG(D)	0,77	1,22	1,99
	CTA(RC(D))	0,89	1,31	2,20
	ATC(C(D))	1,22	0,77	1,99
	GAT(R(D))	1,31	0,89	2,20
TAA	TAA(D)	1,18	1,57	2,75
	TTA(RC(D))	1,25	1,56	2,80
	ATT(C(D))	1,57	1,18	2,75
	AAT(R(D))	1,56	1,25	2,80
AAC	AAC(D)	1,33	1,59	2,92
	GTT(RC(D))	1,27	1,66	2,92
	TTG(C(D))	1,59	1,33	2,92
	CAA(R(D))	1,66	1,27	2,92
AAG	AAG(D)	2,13	1,67	3,80
	CTT(RC(D))	1,67	2,18	3,85
	TTC(C(D))	1,67	2,13	3,80
	GAA(R(D))	2,18	1,67	3,85
ACA	ACA(D)	0,86	0,91	1,77
	TGT(RC(D))	0,91	0,86	1,77
	TGT(C(D))	0,91	0,86	1,77
	ACA(R(D))	0,86	0,91	1,77
ATA	ATA(D)	0,54	0,58	1,12
	TAT(RC(D))	0,58	0,54	1,12
	TAT(C(D))	0,58	0,54	1,12
	ATA(R(D))	0,54	0,54	1,08
AGA	AGA(D)	1,15	0,88	2,03
	TCT(RC(D))	0,88	1,15	2,03
	TCT(C(D))	0,88	1,15	2,03
	AGA(R(D))	1,15	0,88	2,03
AAA	AAA(D)	1,30	1,20	2,50
	TTT(RC(D))	1,20	1,30	2,50
	TTT(C(D))	1,20	1,30	2,50
	AAA(R(D))	1,30	1,20	2,50

Composition of DNA sequence in coding chromosome 21

Base	No.of bases	%
A	501988	25,91%
C	473137	24,42%
G	487525	25,16%
T	474818	24,51%
N	0	0,00%
Sum	1937468	100,00%

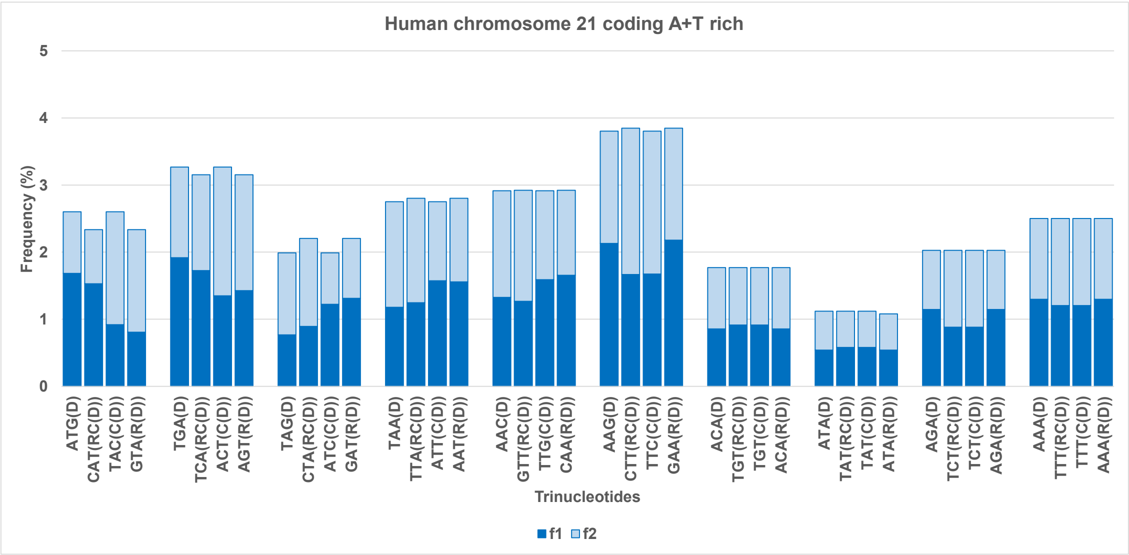

Human assembled chromomome 21

						C+G rich						
**D**	**f**	**%**	**RC**	**f**	**%**	**C**	**f**	**%**	**R**	**f**	**%**	
**CGT**	124172,00	0,31	ACG	125079,00	0,31	GCA	591034,00	1,47	TGC	573105,00	1,43	IIa
**GTC**	377390,00	0,94	GAC	383329,00	0,96	CAG	819512,00	2,04	CTG	791025,00	1,97	
**GCT**	538367,00	1,34	AGC	563171,00	1,40	CGA	95946,00	0,24	TCG	103237,00	0,26	
**GCC**	466603,00	1,16	GGC	458964,00	1,14	CGG	131673,00	0,33	CCG	124241,00	0,31	IIb
**CCA**	715972,00	1,79	TGG	733206,00	1,83	GGT	449511,00	1,12	ACC	447623,00	1,12	
**CCT**	683372,00	1,70	AGG	677401,00	1,69	GGA	633807,00	1,58	TCC	609358,00	1,52	
**CAC**	615856,00	1,54	GTG	632749,00	1,58	GTG*			CAC*			IIc
**CGC**	110506,00	0,28	GCG	109742,00	0,27	GCG*			CGC*			
**CTC**	666283,00	1,66	GAG	667188,00	1,66	GAG*			CTC*			
**CCC**	519822,00	1,30	GGG	520393,00	1,30	GGG*			CCC*			

Quadruplet		f1	f2	fu
	CGT(D)	0,31	1,47	1,78
CGT	ACG(RC(D))	0,31	1,43	1,74
	GCA(C(D))	1,47	0,31	1,78
	TGC(R(D))	1,43	0,31	1,74
	GTC(D)	0,94	2,04	2,99
GTC	GAC(RC(D))	0,96	1,97	2,93
	CAG(C(D))	2,04	0,94	2,99
	CTG(R(D))	1,97	0,96	2,93
	GCT(D)	1,34	0,24	1,58
GCT	AGC(RC(D))	1,40	0,26	1,66
	CGA(C(D))	0,24	1,34	1,58
	TCG(R(D))	0,26	1,40	1,66
	GCC(D)	1,16	0,33	1,49
GCC	GGC(RC(D))	1,14	0,31	1,45
	CGG(C(D))	0,33	1,16	1,49
	CCG(R(D))	0,31	1,14	1,45
	CCA(D)	1,79	1,12	2,91
CCA	TGG(RC(D))	1,83	1,12	2,95
	GGT(C(D))	1,12	1,79	2,91
	ACC(R(D))	1,12	1,83	2,95
	CCT(D)	1,70	1,58	3,29
CCT	AGG(RC(D))	1,69	1,52	3,21
	GGA(C(D))	1,58	1,70	3,29
	TCC(R(D))	1,52	1,69	3,21
	CAC(D)	0,77	0,79	1,56
CAC	GTG(RC(D))	0,79	0,77	1,56
	GTG(C(D))	0,79	0,77	1,56
	CAC(R(D))	0,77	0,79	1,56
	CGC(D)	0,14	0,14	0,27
CGC	GCG(RC(D))	0,14	0,14	0,27
	GCG(C(D))	0,14	0,14	0,27
	CGC(R(D))	0,14	0,14	0,27
	CTC(D)	0,83	0,83	1,66
CTC	GAG(RC(D))	0,83	0,83	1,66
	GAG(C(D))	0,83	0,83	1,66
	CTC(R(D))	0,83	0,83	1,66
	CCC(D)	0,65	0,65	1,30
CCC	GGG(RC(D))	0,65	0,65	1,30
	GGG(C(D))	0,65	0,65	1,30
	CCC(R(D))	0,65	0,65	1,30

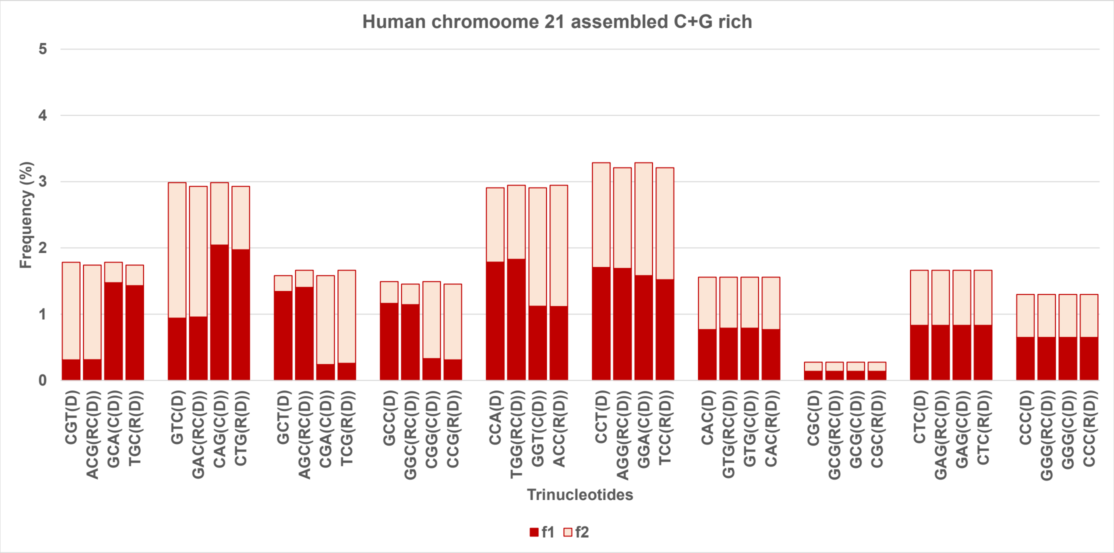

Human random chromomome 21

C+G rich

D	f	%	RC	f	%	C	f	%	R	f	%	
**CGT**	638,00	0,33	ACG	712,00	0,37	GCA	2895,00	1,50	TGC	2444,00	1,27	IIa
**GTC**	1475,00	0,76	GAC	1756,00	0,91	CAG	3826,00	1,98	CTG	2949,00	1,53	
**GCT**	2118,00	1,10	AGC	2755,00	1,43	CGA	403,00	0,21	TCG	558,00	0,29	
**GCC**	1554,00	0,80	GGC	1326,00	0,69	CGG	505,00	0,26	CCG	319,00	0,17	IIb
**CCA**	2606,00	1,35	TGG	3290,00	1,70	GGT	1667,00	0,86	ACC	1609,00	0,83	
**CCT**	2701,00	1,40	AGG	2583,00	1,34	GGA	3385,00	1,75	TCC	2463,00	1,28	
**CAC**	2502,00	1,30	GTG	3015,00	1,56	GTG*			CAC*			IIc
**CGC**	443,00	0,23	GCG	401,00	0,21	GCG*			CGC*			
**CTC**	2885,00	1,49	GAG	3044,00	1,58	GAG*			CTC*			
**CCC**	1553,00	0,80	GGG	1621,00	0,84	GGG*			CCC*			

Quadruplet		f1	f2	fu
CGT	CGT(D)	0,33	1,50	1,83
	ACG(RC(D))	0,37	1,27	1,63
	GCA(C(D))	1,50	0,33	1,83
	TGC(R(D))	1,27	0,37	1,63
GTC	GTC(D)	0,76	1,98	2,75
	GAC(RC(D))	0,91	1,53	2,44
	CAG(C(D))	1,98	0,76	2,75
	CTG(R(D))	1,53	0,91	2,44
GCT	GCT(D)	1,10	0,21	1,31
	AGC(RC(D))	1,43	0,29	1,72
	CGA(C(D))	0,21	1,10	1,31
	TCG(R(D))	0,29	1,43	1,72
GCC	GCC(D)	0,80	0,26	1,07
	GGC(RC(D))	0,69	0,17	0,85
	CGG(C(D))	0,26	0,80	1,07
	CCG(R(D))	0,17	0,69	0,85
CCA	CCA(D)	1,35	0,86	2,21
	TGG(RC(D))	1,70	0,83	2,54
	GGT(C(D))	0,86	1,35	2,21
	ACC(R(D))	0,83	1,70	2,54
CCT	CCT(D)	1,40	1,75	3,15
	AGG(RC(D))	1,34	1,28	2,61
	GGA(C(D))	1,75	1,40	3,15
	TCC(R(D))	1,28	1,34	2,61
CAC	CAC(D)	0,65	0,78	1,43
	GTG(RC(D))	0,78	0,65	1,43
	GTG(C(D))	0,78	0,65	1,43
	CAC(R(D))	0,65	0,78	1,43
CGC	CGC(D)	0,11	0,10	0,22
	GCG(RC(D))	0,10	0,11	0,22
	GCG(C(D))	0,10	0,11	0,22
	CGC(R(D))	0,11	0,10	0,22
CTC	CTC(D)	0,75	0,79	1,54
	GAG(RC(D))	0,79	0,75	1,54
	GAG(C(D))	0,79	0,75	1,54
	CTC(R(D))	0,75	0,79	1,54
CCC	CCC(D)	0,40	0,42	0,82
	GGG(RC(D))	0,42	0,40	0,82
	GGG(C(D))	0,42	0,40	0,82
	CCC(R(D))	0,40	0,42	0,82
		0,00	0,00	

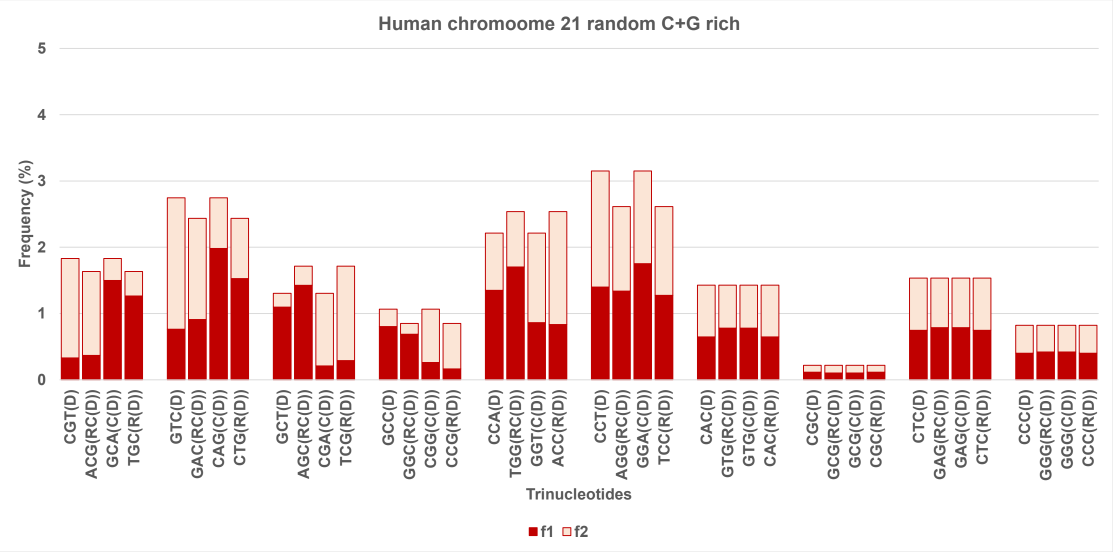

Human coding chromomome 21

D	f	%	RC	f	%	C	f	%	R	f	%	
						C+G rich						
**CGT**	12081,00	0,62	ACG	12798,00	0,66	GCA	34501,00	1,78	TGC	35981,00	1,86	IIa
**GTC**	22019,00	1,14	GAC	26175,00	1,35	CAG	49920,00	2,58	CTG	51570,00	2,66	
**GCT**	35078,00	1,81	AGC	36857,00	1,90	CGA	11701,00	0,60	TCG	10618,00	0,55	
**GCC**	37647,00	1,94	GGC	34608,00	1,79	CGG	18720,00	0,97	CCG	18866,00	0,97	IIb
**CCA**	41265,00	2,13	TGG	42404,00	2,19	GGT	23080,00	1,19	ACC	28308,00	1,46	
**CCT**	39516,00	2,04	AGG	37490,00	1,94	GGA	40927,00	2,11	TCC	33693,00	1,74	
**CAC**	30772,00	1,59	GTG	35976,00	1,86	GTG*			CAC*			IIc
**CGC**	16140,00	0,83	GCG	16360,00	0,84	GCG*			CGC*			
**CTC**	33702,00	1,74	GAG	40404,00	2,09	GAG*			CTC*			
**CCC**	37586,00	1,94	GGG	32903,00	1,70	GGG*			CCC*			

Quadruplet		f1	f2	fu
CGT	CGT(D)	0,62	1,78	2,40
	ACG(RC(D))	0,66	1,86	2,52
	GCA(C(D))	1,78	0,62	2,40
	TGC(R(D))	1,86	0,66	2,52
GTC	GTC(D)	1,14	2,58	3,71
	GAC(RC(D))	1,35	2,66	4,01
	CAG(C(D))	2,58	1,14	3,71
	CTG(R(D))	2,66	1,35	4,01
GCT	GCT(D)	1,81	0,60	2,41
	AGC(RC(D))	1,90	0,55	2,45
	CGA(C(D))	0,60	1,81	2,41
	TCG(R(D))	0,55	1,90	2,45
GCC	GCC(D)	1,94	0,97	2,91
	GGC(RC(D))	1,79	0,97	2,76
	CGG(C(D))	0,97	1,94	2,91
	CCG(R(D))	0,97	1,79	2,76
CCA	CCA(D)	2,13	1,19	3,32
	TGG(RC(D))	2,19	1,46	3,65
	GGT(C(D))	1,19	2,13	3,32
	ACC(R(D))	1,46	2,19	3,65
CCT	CCT(D)	2,04	2,11	4,15
	AGG(RC(D))	1,94	1,74	3,67
	GGA(C(D))	2,11	2,04	4,15
	TCC(R(D))	1,74	1,94	3,67
CAC	CAC(D)	0,79	0,93	1,72
	GTG(RC(D))	0,93	0,79	1,72
	GTG(C(D))	0,93	0,79	1,72
	CAC(R(D))	0,79	0,93	1,72
CGC	CGC(D)	0,42	0,42	0,84
	GCG(RC(D))	0,42	0,42	0,84
	GCG(C(D))	0,42	0,42	0,84
	CGC(R(D))	0,42	0,42	0,84
CTC	CTC(D)	0,87	1,04	1,91
	GAG(RC(D))	1,04	0,87	1,91
	GAG(C(D))	1,04	0,87	1,91
	CTC(R(D))	0,87	1,04	1,91
CCC	CCC(D)	0,97	0,85	1,82
	GGG(RC(D))	0,85	0,97	1,82
	GGG(C(D))	0,85	0,97	1,82
	CCC(R(D))	0,97	0,85	1,82

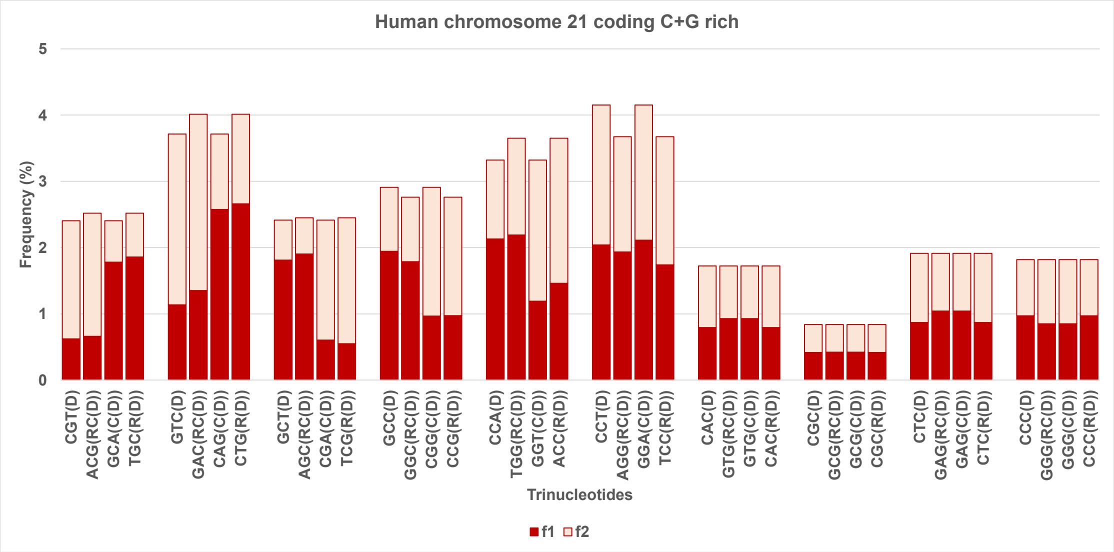

Human assembled chromomome 22

A-T rich												
**D**	**f**	**%**	**RC**	**f**	**%**	**C**	**f**	**%**	**R**	**f**	**%**	
**ATG**	624364,00	1,59	CAT	640853,00	1,64	TAC	352771,00	0,90	GTA	362692,00	0,93	Ia
**TGA**	747362,00	1,91	TCA	730966,00	1,87	ACT	582671,00	1,49	AGT	588367,00	1,50	
**TAG**	423754,00	1,08	CTA	416954,00	1,06	ATC	481698,00	1,23	GAT	497207,00	1,27	
**TAA**	544868,00	1,39	TTA	529392,00	1,35	ATT	702555,00	1,79	AAT	682911,00	1,74	Ib
**AAC**	521254,00	1,33	GTT	500676,00	1,28	TTG	689132,00	1,76	CAA	645056,00	1,65	
**AAG**	701245,00	1,79	CTT	713297,00	1,82	TTC	698001,00	1,78	GAA	715485,00	1,83	
**ACA**	749787,00	1,91	TGT	742273,00	1,90	TGT*			ACA*			Ic
**ATA**	541987,00	1,38	TAT	529630,00	1,35	TAT*			ATA*			
**AGA**	835960,00	2,13	TCT	834278,00	2,13	TCT*			AGA*			
**AAA**	1188130,00	3,03	TTT	1199966,00	3,06	TTT*			AAA*			

Quadruplet		f1	f2	fu
ATG	ATG(D)	1,59	0,90	2,50
	CAT(RC(D))	1,64	0,93	2,56
	TAC(C(D))	0,90	1,59	2,50
	GTA(R(D))	0,93	1,64	2,56
TGA	TGA(D)	1,91	1,49	3,40
	TCA(RC(D))	1,87	1,50	3,37
	ACT(C(D))	1,49	1,91	3,40
	AGT(R(D))	1,50	1,87	3,37
TAG	TAG(D)	1,08	1,23	2,31
	CTA(RC(D))	1,06	1,27	2,33
	ATC(C(D))	1,23	1,08	2,31
	GAT(R(D))	1,27	1,06	2,33
TAA	TAA(D)	1,39	1,79	3,19
	TTA(RC(D))	1,35	1,74	3,10
	ATT(C(D))	1,79	1,39	3,19
	AAT(R(D))	1,74	1,35	3,10
AAC	AAC(D)	1,33	1,76	3,09
	GTT(RC(D))	1,28	1,65	2,93
	TTG(C(D))	1,76	1,33	3,09
	CAA(R(D))	1,65	1,28	2,93
AAG	AAG(D)	1,79	1,78	3,57
	CTT(RC(D))	1,82	1,83	3,65
	TTC(C(D))	1,78	1,79	3,57
	GAA(R(D))	1,83	1,82	3,65
ACA	ACA(D)	0,96	0,95	1,91
	TGT(RC(D))	0,95	0,96	1,91
	TGT(C(D))	0,95	0,96	1,91
	ACA(R(D))	0,96	0,95	1,91
ATA	ATA(D)	0,69	0,68	1,37
	TAT(RC(D))	0,68	0,69	1,37
	TAT(C(D))	0,68	0,69	1,37
	ATA(R(D))	0,69	0,69	1,38
AGA	AGA(D)	1,07	1,07	2,13
	TCT(RC(D))	1,07	1,07	2,13
	TCT(C(D))	1,07	1,07	2,13
	AGA(R(D))	1,07	1,07	2,13
AAA	AAA(D)	1,52	1,53	3,05
	TTT(RC(D))	1,53	1,52	3,05
	TTT(C(D))	1,53	1,52	3,05
	AAA(R(D))	1,52	1,53	3,05

Composition of DNA sequence in assembled chromosome 22

Base	No.of bases	%
A	10382214	20,43%
C	9160652	18,03%
G	9246186	18,19%
T	10370725	20,41%
N	11658691	22,94%
Sum	50818468	100,00%

% of coding bases from complete assembled chromosome 1 (including N bases)

7,21%

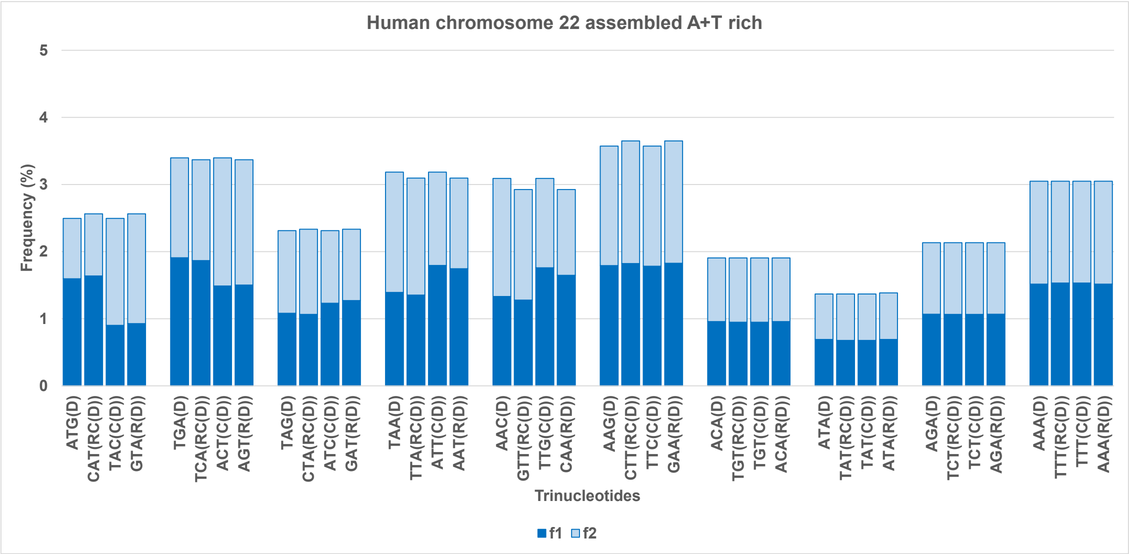

Human random chromomome 22

						A-T rich						
**D**	**f**	**%**	**RC**	**f**	**%**	**C**	**f**	**%**	**R**	**f**	**%**	
**ATG**	2670,00	1,67	CAT	3053,00	1,91	TAC	1432,00	0,89	GTA	1729,00	1,08	Ia
**TGA**	3723,00	2,32	TCA	3209,00	2,00	ACT	2570,00	1,60	AGT	2694,00	1,68	
**TAG**	2103,00	1,31	CTA	1907,00	1,19	ATC	2250,00	1,40	GAT	2744,00	1,71	
**TAA**	2453,00	1,53	TTA	2050,00	1,28	ATT	3993,00	2,49	AAT	3425,00	2,14	Ib
**AAC**	3035,00	1,89	GTT	2427,00	1,51	TTG	3893,00	2,43	CAA	2800,00	1,75	
**AAG**	3165,00	1,98	CTT	3465,00	2,16	TTC	3944,00	2,46	GAA	4372,00	2,73	
**ACA**	3576,00	2,23	TGT	3369,00	2,10	TGT*			ACA*			Ic
**ATA**	3293,00	2,06	TAT	2989,00	1,87	TAT*			ATA*			
**AGA**	4171,00	2,60	TCT	4068,00	2,54	TCT*			AGA*			
**AAA**	6320,00	3,94	TTT	6688,00	4,17	TTT*			AAA*			

Quadruplet		f1	f2	fu
ATG	ATG(D)	1,67	0,89	2,56
	CAT(RC(D))	1,91	1,08	2,98
	TAC(C(D))	0,89	1,67	2,56
	GTA(R(D))	1,08	1,91	2,98
TGA	TGA(D)	2,32	1,60	3,93
	TCA(RC(D))	2,00	1,68	3,68
	ACT(C(D))	1,60	2,32	3,93
	AGT(R(D))	1,68	2,00	3,68
TAG	TAG(D)	1,31	1,40	2,72
	CTA(RC(D))	1,19	1,71	2,90
	ATC(C(D))	1,40	1,31	2,72
	GAT(R(D))	1,71	1,19	2,90
TAA	TAA(D)	1,53	2,49	4,02
	TTA(RC(D))	1,28	2,14	3,42
	ATT(C(D))	2,49	1,53	4,02
	AAT(R(D))	2,14	1,28	3,42
AAC	AAC(D)	1,89	2,43	4,32
	GTT(RC(D))	1,51	1,75	3,26
	TTG(C(D))	2,43	1,89	4,32
	CAA(R(D))	1,75	1,51	3,26
AAG	AAG(D)	1,98	2,46	4,44
	CTT(RC(D))	2,16	2,73	4,89
	TTC(C(D))	2,46	1,98	4,44
	GAA(R(D))	2,73	2,16	4,89
ACA	ACA(D)	1,12	1,05	2,17
	TGT(RC(D))	1,05	1,12	2,17
	TGT(C(D))	1,05	1,12	2,17
	ACA(R(D))	1,12	1,05	2,17
ATA	ATA(D)	1,03	0,93	1,96
	TAT(RC(D))	0,93	1,03	1,96
	TAT(C(D))	0,93	1,03	1,96
	ATA(R(D))	1,03	1,03	2,06
AGA	AGA(D)	1,30	1,27	2,57
	TCT(RC(D))	1,27	1,30	2,57
	TCT(C(D))	1,27	1,30	2,57
	AGA(R(D))	1,30	1,27	2,57
AAA	AAA(D)	1,97	2,09	4,06
	TTT(RC(D))	2,09	1,97	4,06
	TTT(C(D))	2,09	1,97	4,06
	AAA(R(D))	1,97	2,09	4,06

Composition of DNA sequence in random chromosome 22

Base	No.of bases	%
A	48046	24,02%
C	31125	15,56%
G	33318	16,66%
T	47793	23,90%
N	39718	19,86%
Sum	200000	100,00%

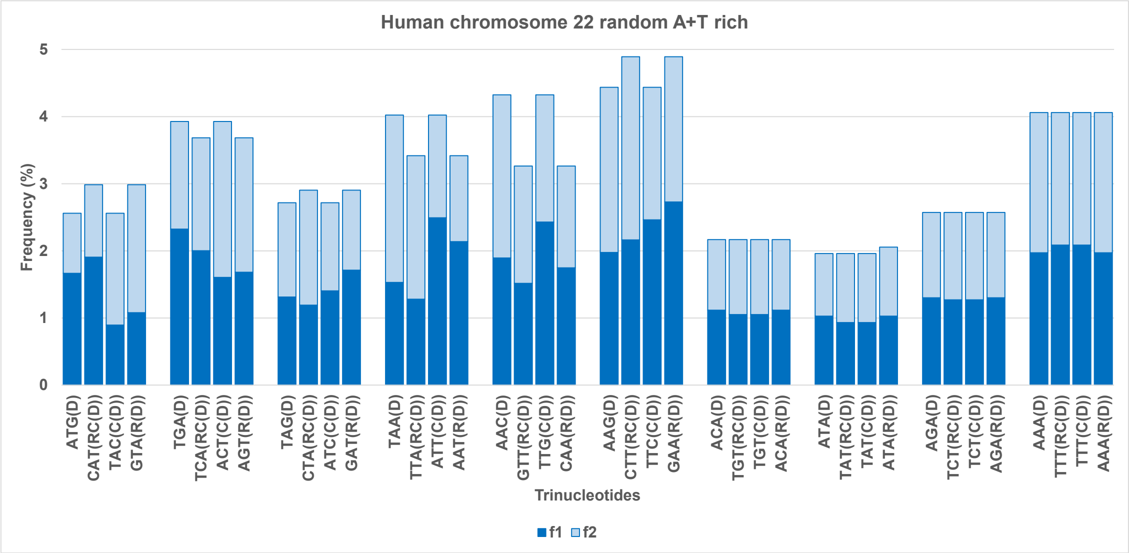

Human coding chromomome 22

A-T rich

D	f	%	RC	f	%	C	f	%	R	f	%	
**ATG**	54552,00	1,49	CAT	51258,00	1,40	TAC	28663,00	0,78	GTA	21825,00	0,60	Ia
**TGA**	63124,00	1,72	TCA	56386,00	1,54	ACT	44860,00	1,22	AGT	45224,00	1,23	
**TAG**	20756,00	0,57	CTA	29393,00	0,80	ATC	40264,00	1,10	GAT	41033,00	1,12	
**TAA**	23968,00	0,65	TTA	25061,00	0,68	ATT	33805,00	0,92	AAT	33438,00	0,91	Ib
**AAC**	40006,00	1,09	GTT	34625,00	0,94	TTG	46136,00	1,26	CAA	54368,00	1,48	
**AAG**	68177,00	1,86	CTT	55940,00	1,53	TTC	53173,00	1,45	GAA	63285,00	1,73	
**ACA**	56912,00	1,55	TGT	56470,00	1,54	TGT*			ACA*			Ic
**ATA**	21682,00	0,59	TAT	24574,00	0,67	TAT*			ATA*			
**AGA**	74079,00	2,02	TCT	59627,00	1,63	TCT*			AGA*			
**AAA**	56264,00	1,54	TTT	57267,00	1,56	TTT*			AAA*			

Quadruplet		f1	f2	fu
ATG	ATG(D)	1,49	0,78	2,27
	CAT(RC(D))	1,40	0,60	1,99
	TAC(C(D))	0,78	1,49	2,27
	GTA(R(D))	0,60	1,40	1,99
TGA	TGA(D)	1,72	1,22	2,95
	TCA(RC(D))	1,54	1,23	2,77
	ACT(C(D))	1,22	1,72	2,95
	AGT(R(D))	1,23	1,54	2,77
TAG	TAG(D)	0,57	1,10	1,67
	CTA(RC(D))	0,80	1,12	1,92
	ATC(C(D))	1,10	0,57	1,67
	GAT(R(D))	1,12	0,80	1,92
TAA	TAA(D)	0,65	0,92	1,58
	TTA(RC(D))	0,68	0,91	1,60
	ATT(C(D))	0,92	0,65	1,58
	AAT(R(D))	0,91	0,68	1,60
AAC	AAC(D)	1,09	1,26	2,35
	GTT(RC(D))	0,94	1,48	2,43
	TTG(C(D))	1,26	1,09	2,35
	CAA(R(D))	1,48	0,94	2,43
AAG	AAG(D)	1,86	1,45	3,31
	CTT(RC(D))	1,53	1,73	3,25
	TTC(C(D))	1,45	1,86	3,31
	GAA(R(D))	1,73	1,53	3,25
ACA	ACA(D)	0,78	0,77	1,55
	TGT(RC(D))	0,77	0,78	1,55
	TGT(C(D))	0,77	0,78	1,55
	ACA(R(D))	0,78	0,77	1,55
ATA	ATA(D)	0,30	0,34	0,63
	TAT(RC(D))	0,34	0,30	0,63
	TAT(C(D))	0,34	0,30	0,63
	ATA(R(D))	0,30	0,30	0,59
AGA	AGA(D)	1,01	0,81	1,82
	TCT(RC(D))	0,81	1,01	1,82
	TCT(C(D))	0,81	1,01	1,82
	AGA(R(D))	1,01	0,81	1,82
AAA	AAA(D)	0,77	0,78	1,55
	TTT(RC(D))	0,78	0,77	1,55
	TTT(C(D))	0,78	0,77	1,55
	AAA(R(D))	0,77	0,78	1,55

Composition of DNA sequence in coding chromosome 22

Base	No.of bases	%
A	827260	22,57%
C	1029316	28,09%
G	1027485	28,04%
T	780732	21,30%
N	0	0,00%
Sum	3664793	100,00%

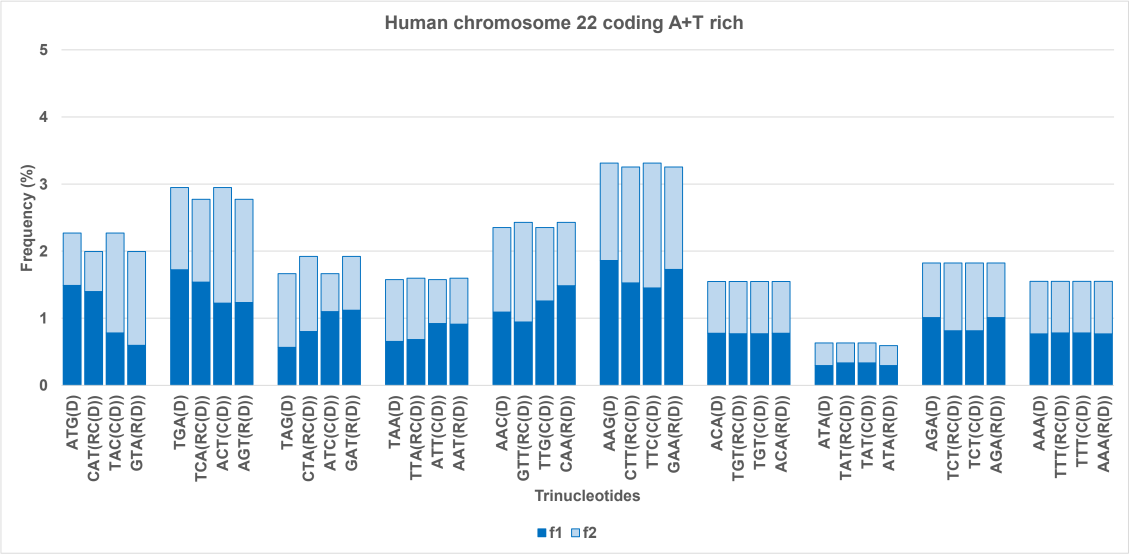

						C+G rich						
**D**	**f**	**%**	**RC**	**f**	**%**	**C**	**f**	**%**	**R**	**f**	**%**	
**CGT**	145546,00	0,37	ACG	148623,00	0,38	GCA	660789,00	1,69	TGC	640053,00	1,63	IIa
**GTC**	412954,00	1,05	GAC	423396,00	1,08	CAG	1003603,00	2,56	CTG	971470,00	2,48	
**GCT**	639215,00	1,63	AGC	665282,00	1,70	CGA	125868,00	0,32	TCG	132013,00	0,34	
**GCC**	670890,00	1,71	GGC	664788,00	1,70	CGG	197532,00	0,50	CCG	189083,00	0,48	IIb
**CCA**	851511,00	2,17	TGG	878748,00	2,24	GGT	523603,00	1,34	ACC	519884,00	1,33	
**CCT**	847664,00	2,16	AGG	847474,00	2,16	GGA	735366,00	1,88	TCC	697484,00	1,78	
**CAC**	703544,00	1,80	GTG	723469,00	1,85	GTG*			CAC*			IIc
**CGC**	165698,00	0,42	GCG	164927,00	0,42	GCG*			CGC*			
**CTC**	802104,00	2,05	GAG	808474,00	2,06	GAG*			CTC*			
**CCC**	740845,00	1,89	GGG	742239,00	1,90	GGG*			CCC*			

Quadruplet		f1	f2	fu
CGT	CGT(D)	0,37	1,69	2,06
	ACG(RC(D))	0,38	1,63	2,01
	GCA(C(D))	1,69	0,37	2,06
	TGC(R(D))	1,63	0,38	2,01
GTC	GTC(D)	1,05	2,56	3,62
	GAC(RC(D))	1,08	2,48	3,56
	CAG(C(D))	2,56	1,05	3,62
	CTG(R(D))	2,48	1,08	3,56
GCT	GCT(D)	1,63	0,32	1,95
	AGC(RC(D))	1,70	0,34	2,04
	CGA(C(D))	0,32	1,63	1,95
	TCG(R(D))	0,34	1,70	2,04
GCC	GCC(D)	1,71	0,50	2,22
	GGC(RC(D))	1,70	0,48	2,18
	CGG(C(D))	0,50	1,71	2,22
	CCG(R(D))	0,48	1,70	2,18
CCA	CCA(D)	2,17	1,34	3,51
	TGG(RC(D))	2,24	1,33	3,57
	GGT(C(D))	1,34	2,17	3,51
	ACC(R(D))	1,33	2,24	3,57
CCT	CCT(D)	2,16	1,88	4,04
	AGG(RC(D))	2,16	1,78	3,95
	GGA(C(D))	1,88	2,16	4,04
	TCC(R(D))	1,78	2,16	3,95
CAC	CAC(D)	0,90	0,92	1,82
	GTG(RC(D))	0,92	0,90	1,82
	GTG(C(D))	0,92	0,90	1,82
	CAC(R(D))	0,90	0,92	1,82
CGC	CGC(D)	0,21	0,21	0,42
	GCG(RC(D))	0,21	0,21	0,42
	GCG(C(D))	0,21	0,21	0,42
	CGC(R(D))	0,21	0,21	0,42
CTC	CTC(D)	1,02	1,03	2,06
	GAG(RC(D))	1,03	1,02	2,06
	GAG(C(D))	1,03	1,02	2,06
	CTC(R(D))	1,02	1,03	2,06
CCC	CCC(D)	0,95	0,95	1,89
	GGG(RC(D))	0,95	0,95	1,89
	GGG(C(D))	0,95	0,95	1,89
	CCC(R(D))	0,95	0,95	1,89

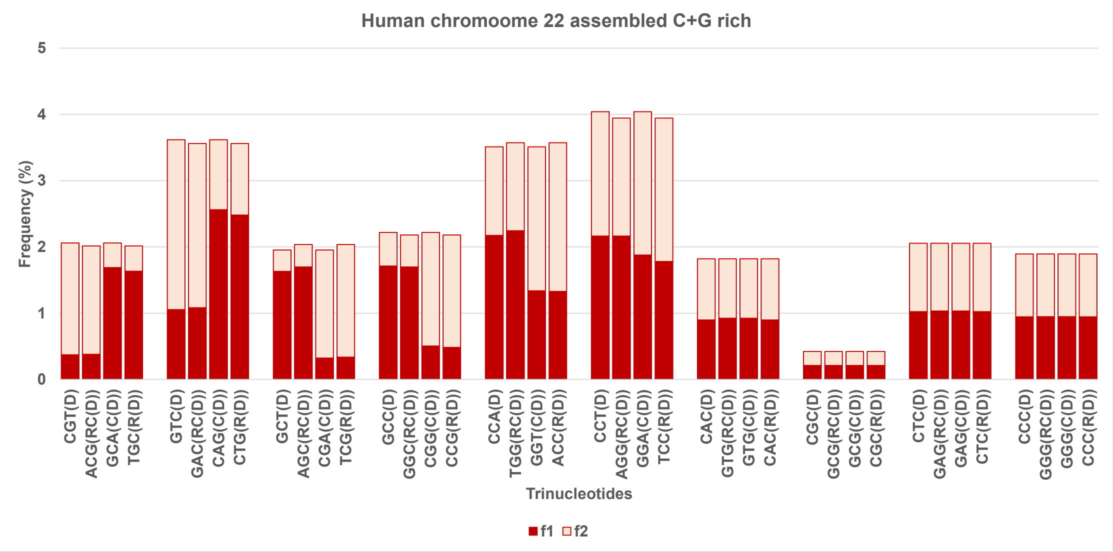

Human random chromomome 22

C+G rich												
**D**	**f**	**%**	**RC**	**f**	**%**	**C**	**f**	**%**	**R**	**f**	**%**	
**CGT**	599,00	0,37	ACG	698,00	0,44	GCA	2581,00	1,61	TGC	2061,00	1,29	IIa
**GTC**	1241,00	0,77	GAC	1532,00	0,96	CAG	3533,00	2,20	CTG	2843,00	1,77	
**GCT**	1787,00	1,12	AGC	2480,00	1,55	CGA	485,00	0,30	TCG	627,00	0,39	
**GCC**	1445,00	0,90	GGC	1288,00	0,80	CGG	578,00	0,36	CCG	319,00	0,20	IIb
**CCA**	2274,00	1,42	TGG	3013,00	1,88	GGT	1489,00	0,93	ACC	1410,00	0,88	
**CCT**	2419,00	1,51	AGG	2281,00	1,42	GGA	3094,00	1,93	TCC	2158,00	1,35	
**CAC**	2255,00	1,41	GTG	2758,00	1,72	GTG*			CAC*			IIc
**CGC**	423,00	0,26	GCG	441,00	0,28	GCG*			CGC*			
**CTC**	2629,00	1,64	GAG	2824,00	1,76	GAG*			CTC*			
**CCC**	1535,00	0,96	GGG	1560,00	0,97	GGG*			CCC*			

Quadruplet		f1	f2	fu
CGT	CGT(D)	0,37	1,61	1,98
	ACG(RC(D))	0,44	1,29	1,72
	GCA(C(D))	1,61	0,37	1,98
	TGC(R(D))	1,29	0,44	1,72
GTC	GTC(D)	0,77	2,20	2,98
	GAC(RC(D))	0,96	1,77	2,73
	CAG(C(D))	2,20	0,77	2,98
	CTG(R(D))	1,77	0,96	2,73
GCT	GCT(D)	1,12	0,30	1,42
	AGC(RC(D))	1,55	0,39	1,94
	CGA(C(D))	0,30	1,12	1,42
	TCG(R(D))	0,39	1,55	1,94
GCC	GCC(D)	0,90	0,36	1,26
	GGC(RC(D))	0,80	0,20	1,00
	CGG(C(D))	0,36	0,90	1,26
	CCG(R(D))	0,20	0,80	1,00
CCA	CCA(D)	1,42	0,93	2,35
	TGG(RC(D))	1,88	0,88	2,76
	GGT(C(D))	0,93	1,42	2,35
	ACC(R(D))	0,88	1,88	2,76
CCT	CCT(D)	1,51	1,93	3,44
	AGG(RC(D))	1,42	1,35	2,77
	GGA(C(D))	1,93	1,51	3,44
	TCC(R(D))	1,35	1,42	2,77
CAC	CAC(D)	0,70	0,86	1,56
	GTG(RC(D))	0,86	0,70	1,56
	GTG(C(D))	0,86	0,70	1,56
	CAC(R(D))	0,70	0,86	1,56
CGC	CGC(D)	0,13	0,14	0,27
	GCG(RC(D))	0,14	0,13	0,27
	GCG(C(D))	0,14	0,13	0,27
	CGC(R(D))	0,13	0,14	0,27
CTC	CTC(D)	0,82	0,88	1,70
	GAG(RC(D))	0,88	0,82	1,70
	GAG(C(D))	0,88	0,82	1,70
	CTC(R(D))	0,82	0,88	1,70
CCC	CCC(D)	0,48	0,49	0,97
	GGG(RC(D)	0,49	0,48	0,97
	GGG(C(D))	0,49	0,48	0,97
	CCC(R(D))	0,48	0,49	0,97

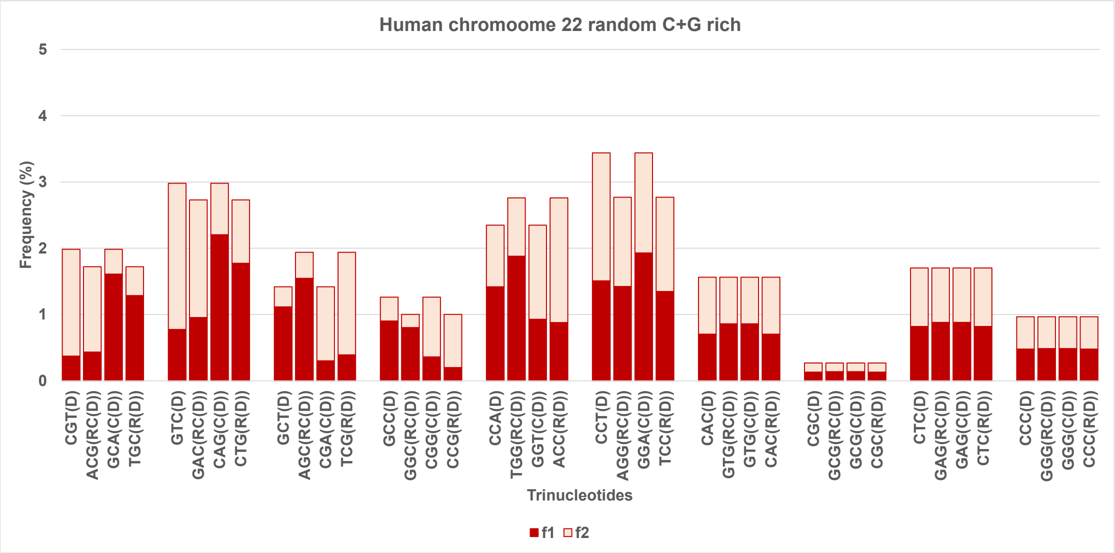

Human coding chromomome 22

C+G rich

D	f	%	RC	f	%	C	f	%	R	f	%	
**CGT**	22707,00	0,62	ACG	26013,00	0,71	GCA	73460,00	2,00	TGC	75763,00	2,07	IIa
**GTC**	42480,00	1,16	GAC	56129,00	1,53	CAG	109310,00	2,98	CTG	116459,00	3,18	
**GCT**	78534,00	2,14	AGC	83768,00	2,29	CGA	26537,00	0,72	TCG	22257,00	0,61	
**GCC**	99381,00	2,71	GGC	91386,00	2,49	CGG	45660,00	1,25	CCG	47092,00	1,28	IIb
**CCA**	93976,00	2,56	TGG	95220,00	2,60	GGT	47959,00	1,31	ACC	62811,00	1,71	
**CCT**	93411,00	2,55	AGG	85404,00	2,33	GGA	86939,00	2,37	TCC	72287,00	1,97	
**CAC**	65798,00	1,80	GTG	73430,00	2,00	GTG*			CAC*			IIc
**CGC**	40952,00	1,12	GCG	40494,00	1,10	GCG*			CGC*			
**CTC**	74640,00	2,04	GAG	90232,00	2,46	GAG*			CTC*			
**CCC**	101815,00	2,78	GGG	86292,00	2,35	GGG*			CCC*			

Quadruplet		f1	f2	fu
CGT	CGT(D)	0,62	2,00	2,62
	ACG(RC(D))	0,71	2,07	2,78
	GCA(C(D))	2,00	0,62	2,62
	TGC(R(D))	2,07	0,71	2,78
GTC	GTC(D)	1,16	2,98	4,14
	GAC(RC(D))	1,53	3,18	4,71
	CAG(C(D))	2,98	1,16	4,14
	CTG(R(D))	3,18	1,53	4,71
GCT	GCT(D)	2,14	0,72	2,87
	AGC(RC(D))	2,29	0,61	2,89
	CGA(C(D))	0,72	2,14	2,87
	TCG(R(D))	0,61	2,29	2,89
GCC	GCC(D)	2,71	1,25	3,96
	GGC(RC(D))	2,49	1,28	3,78
	CGG(C(D))	1,25	2,71	3,96
	CCG(R(D))	1,28	2,49	3,78
CCA	CCA(D)	2,56	1,31	3,87
	TGG(RC(D))	2,60	1,71	4,31
	GGT(C(D))	1,31	2,56	3,87
	ACC(R(D))	1,71	2,60	4,31
CCT	CCT(D)	2,55	2,37	4,92
	AGG(RC(D))	2,33	1,97	4,30
	GGA(C(D))	2,37	2,55	4,92
	TCC(R(D))	1,97	2,33	4,30
CAC	CAC(D)	0,90	1,00	1,90
	GTG(RC(D))	1,00	0,90	1,90
	GTG(C(D))	1,00	0,90	1,90
	CAC(R(D))	0,90	1,00	1,90
CGC	CGC(D)	0,56	0,55	1,11
	GCG(RC(D))	0,55	0,56	1,11
	GCG(C(D))	0,55	0,56	1,11
	CGC(R(D))	0,56	0,55	1,11
CTC	CTC(D)	1,02	1,23	2,25
	GAG(RC(D))	1,23	1,02	2,25
	GAG(C(D))	1,23	1,02	2,25
	CTC(R(D))	1,02	1,23	2,25
CCC	CCC(D)	1,39	1,18	2,57
	GGG(RC(D)	1,18	1,39	2,57
	GGG(C(D))	1,18	1,39	2,57
	CCC(R(D))	1,39	1,18	2,57

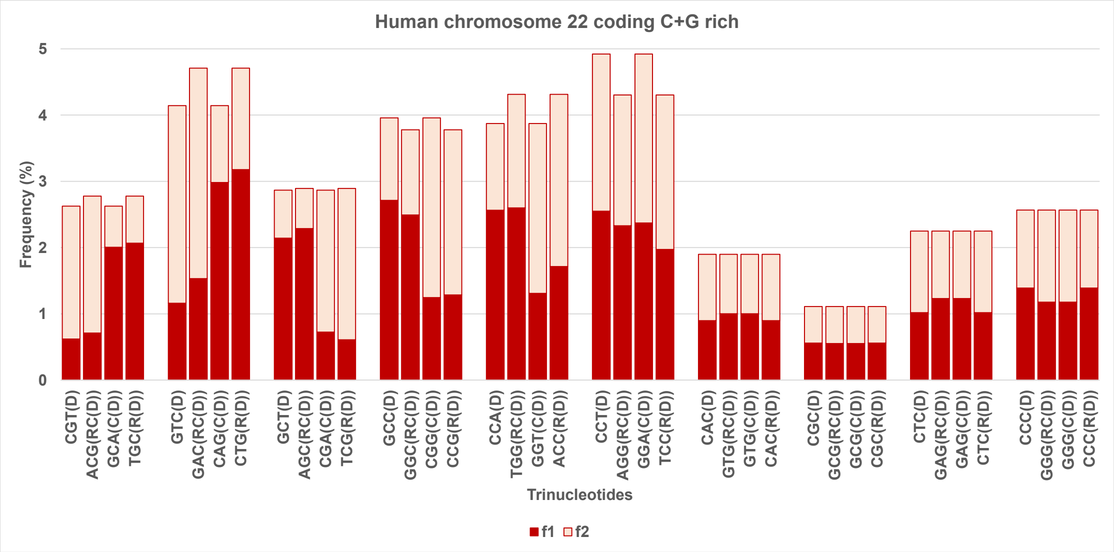

Human assembled chromomome X

A-T rich												
**D**	**f**	**%**	**RC**	**f**	**%**	**C**	**f**	**%**	**R**	**f**	**%**	
**ATG**	2950712,00	1,91	CAT	2960335,00	1,91	TAC	1811693,00	1,17	GTA	1840326,00	1,19	Ia
**TGA**	3099865,00	2,00	TCA	3070480,00	1,98	ACT	2527616,00	1,63	AGT	2513605,00	1,62	
**TAG**	2086790,00	1,35	CTA	2072867,00	1,34	ATC	2129987,00	1,38	GAT	2171879,00	1,40	
**TAA**	3270805,00	2,11	TTA	3255256,00	2,10	ATT	4066554,00	2,63	AAT	4015878,00	2,59	Ib
**AAC**	2396220,00	1,55	GTT	2369800,00	1,53	TTG	3161533,00	2,04	CAA	3065430,00	1,98	
**AAG**	3128712,00	2,02	CTT	3179043,00	2,05	TTC	3198607,00	2,07	GAA	3204572,00	2,07	
**ACA**	3201493,00	2,07	TGT	3208953,00	2,07	TGT*			ACA*			Ic
**ATA**	3482068,00	2,25	TAT	3481227,00	2,25	TAT*			ATA*			
**AGA**	3487257,00	2,25	TCT	3493023,00	2,26	TCT*			AGA*			
**AAA**	6138782,00	3,96	TTT	6245590,00	4,03	TTT*			AAA*			

Quadruplet		f1	f2	fu
ATG	ATG(D)	1,91	1,17	3,07
	CAT(RC(D))	1,91	1,19	3,10
	TAC(C(D))	1,17	1,91	3,07
	GTA(R(D))	1,19	1,91	3,10
TGA	TGA(D)	2,00	1,63	3,63
	TCA(RC(D))	1,98	1,62	3,61
	ACT(C(D))	1,63	2,00	3,63
	AGT(R(D))	1,62	1,98	3,61
TAG	TAG(D)	1,35	1,38	2,72
	CTA(RC(D))	1,34	1,40	2,74
	ATC(C(D))	1,38	1,35	2,72
	GAT(R(D))	1,40	1,34	2,74
TAA	TAA(D)	2,11	2,63	4,74
	TTA(RC(D))	2,10	2,59	4,69
	ATT(C(D))	2,63	2,11	4,74
	AAT(R(D))	2,59	2,10	4,69
AAC	AAC(D)	1,55	2,04	3,59
	GTT(RC(D))	1,53	1,98	3,51
	TTG(C(D))	2,04	1,55	3,59
	CAA(R(D))	1,98	1,53	3,51
AAG	AAG(D)	2,02	2,07	4,08
	CTT(RC(D))	2,05	2,07	4,12
	TTC(C(D))	2,07	2,02	4,08
	GAA(R(D))	2,07	2,05	4,12
ACA	ACA(D)	1,03	1,04	2,07
	TGT(RC(D))	1,04	1,03	2,07
	TGT(C(D))	1,04	1,03	2,07
	ACA(R(D))	1,03	1,04	2,07
ATA	ATA(D)	1,12	1,12	2,25
	TAT(RC(D))	1,12	1,12	2,25
	TAT(C(D))	1,12	1,12	2,25
	ATA(R(D))	1,12	1,12	2,25
AGA	AGA(D)	1,13	1,13	2,25
	TCT(RC(D))	1,13	1,13	2,25
	TCT(C(D))	1,13	1,13	2,25
	AGA(R(D))	1,13	1,13	2,25
AAA	AAA(D)	1,98	2,02	4,00
	TTT(RC(D))	2,02	1,98	4,00
	TTT(C(D))	2,02	1,98	4,00
	AAA(R(D))	1,98	2,02	4,00

Composition of DNA sequence in assembled chromosome X

Base	No.of bases	%
A	46754807	29,96%
C	30523780	19,56%
G	30697741	19,67%
T	46916701	30,07%
N	1147866	0,74%
Sum	156040895	100,00%

% of coding bases from complete assembled chromosome 1 (including N bases)

4,27%

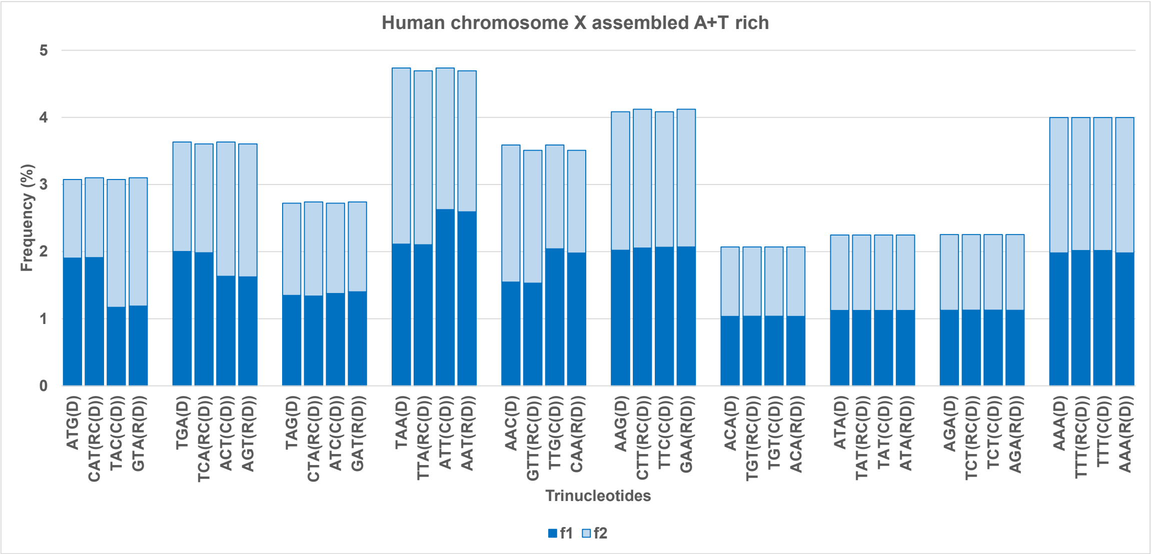

Human random chromomome X

A-T rich

D	f	%	RC	f	%	C	f	%	R	f	%	
**ATG**	3692,00	1,86	CAT	3683,00	1,86	TAC	2220,00	1,12	GTA	2274,00	1,15	Ia
**TGA**	4002,00	2,02	TCA	3867,00	1,95	ACT	3232,00	1,63	AGT	3129,00	1,58	
**TAG**	2647,00	1,34	CTA	2501,00	1,26	ATC	2601,00	1,31	GAT	2832,00	1,43	
**TAA**	3974,00	2,01	TTA	3961,00	2,00	ATT	5005,00	2,53	AAT	4888,00	2,47	Ib
**AAC**	3225,00	1,63	GTT	3024,00	1,53	TTG	3986,00	2,01	CAA	3855,00	1,95	
**AAG**	3888,00	1,96	CTT	4138,00	2,09	TTC	4220,00	2,13	GAA	4173,00	2,11	
**ACA**	4033,00	2,03	TGT	4184,00	2,11	TGT*			ACA*			Ic
**ATA**	4154,00	2,10	TAT	4049,00	2,04	TAT*			ATA*			
**AGA**	4490,00	2,27	TCT	4646,00	2,34	TCT*			AGA*			
**AAA**	7579,00	3,82	TTT	7871,00	3,97	TTT*			AAA*			

Quadruplet		f1	f2	fu
ATG	ATG(D)	1,86	1,12	2,98
	CAT(RC(D))	1,86	1,15	3,01
	TAC(C(D))	1,12	1,86	2,98
	GTA(R(D))	1,15	1,86	3,01
TGA	TGA(D)	2,02	1,63	3,65
	TCA(RC(D))	1,95	1,58	3,53
	ACT(C(D))	1,63	2,02	3,65
	AGT(R(D))	1,58	1,95	3,53
TAG	TAG(D)	1,34	1,31	2,65
	CTA(RC(D))	1,26	1,43	2,69
	ATC(C(D))	1,31	1,34	2,65
	GAT(R(D))	1,43	1,26	2,69
TAA	TAA(D)	2,01	2,53	4,53
	TTA(RC(D))	2,00	2,47	4,47
	ATT(C(D))	2,53	2,01	4,53
	AAT(R(D))	2,47	2,00	4,47
AAC	AAC(D)	1,63	2,01	3,64
	GTT(RC(D))	1,53	1,95	3,47
	TTG(C(D))	2,01	1,63	3,64
	CAA(R(D))	1,95	1,53	3,47
AAG	AAG(D)	1,96	2,13	4,09
	CTT(RC(D))	2,09	2,11	4,19
	TTC(C(D))	2,13	1,96	4,09
	GAA(R(D))	2,11	2,09	4,19
ACA	ACA(D)	1,02	1,06	2,07
	TGT(RC(D))	1,06	1,02	2,07
	TGT(C(D))	1,06	1,02	2,07
	ACA(R(D))	1,02	1,06	2,07
ATA	ATA(D)	1,05	1,02	2,07
	TAT(RC(D))	1,02	1,05	2,07
	TAT(C(D))	1,02	1,05	2,07
	ATA(R(D))	1,05	1,05	2,10
AGA	AGA(D)	1,13	1,17	2,30
	TCT(RC(D))	1,17	1,13	2,30
	TCT(C(D))	1,17	1,13	2,30
	AGA(R(D))	1,13	1,17	2,30
AAA	AAA(D)	1,91	1,99	3,90
	TTT(RC(D))	1,99	1,91	3,90
	TTT(C(D))	1,99	1,91	3,90
	AAA(R(D))	1,91	1,99	3,90

Composition of DNA sequence in random chromosome X

Base	No.of bases	%
A	58791	29,40%
C	39891	19,95%
G	40062	20,03%
T	59456	29,73%
N	1800	0,90%
Sum	200000	100,00%

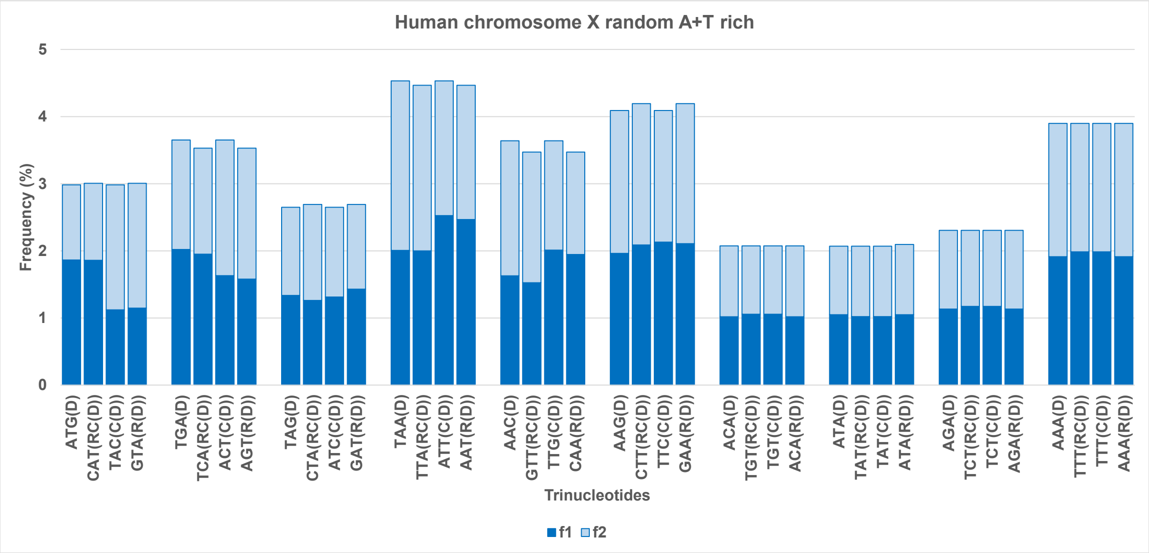

Human coding chromomome X

A-T rich

D	f	%	RC	f	%	C	f	%	R	f	%	
**ATG**	122899,00	1,85	CAT	105793,00	1,59	TAC	67229,00	1,01	GTA	59925,00	0,90	Ia
**TGA**	134894,00	2,03	TCA	118102,00	1,77	ACT	96631,00	1,45	AGT	100442,00	1,51	
**TAG**	57080,00	0,86	CTA	69443,00	1,04	ATC	84442,00	1,27	GAT	94435,00	1,42	
**TAA**	84415,00	1,27	TTA	86714,00	1,30	ATT	113502,00	1,70	AAT	113257,00	1,70	Ib
**AAC**	91386,00	1,37	GTT	85191,00	1,28	TTG	115207,00	1,73	CAA	122122,00	1,83	
**AAG**	149892,00	2,25	CTT	119840,00	1,80	TTC	116612,00	1,75	GAA	147999,00	2,22	
**ACA**	117232,00	1,76	TGT	121256,00	1,82	TGT*			ACA*			Ic
**ATA**	80764,00	1,21	TAT	88122,00	1,32	TAT*			ATA*			
**AGA**	157325,00	2,36	TCT	122034,00	1,83	TCT*			AGA*			
**AAA**	179523,00	2,70	TTT	167221,00	2,51	TTT*			AAA*			

Quadruplet		f1	f2	fu
ATG	ATG(D)	1,85	1,01	2,85
	CAT(RC(D))	1,59	0,90	2,49
	TAC(C(D))	1,01	1,85	2,85
	GTA(R(D))	0,90	1,59	2,49
TGA	TGA(D)	2,03	1,45	3,48
	TCA(RC(D))	1,77	1,51	3,28
	ACT(C(D))	1,45	2,03	3,48
	AGT(R(D))	1,51	1,77	3,28
TAG	TAG(D)	0,86	1,27	2,12
	CTA(RC(D))	1,04	1,42	2,46
	ATC(C(D))	1,27	0,86	2,12
	GAT(R(D))	1,42	1,04	2,46
TAA	TAA(D)	1,27	1,70	2,97
	TTA(RC(D))	1,30	1,70	3,00
	ATT(C(D))	1,70	1,27	2,97
	AAT(R(D))	1,70	1,30	3,00
AAC	AAC(D)	1,37	1,73	3,10
	GTT(RC(D))	1,28	1,83	3,11
	TTG(C(D))	1,73	1,37	3,10
	CAA(R(D))	1,83	1,28	3,11
AAG	AAG(D)	2,25	1,75	4,00
	CTT(RC(D))	1,80	2,22	4,02
	TTC(C(D))	1,75	2,25	4,00
	GAA(R(D))	2,22	1,80	4,02
ACA	ACA(D)	0,88	0,91	1,79
	TGT(RC(D))	0,91	0,88	1,79
	TGT(C(D))	0,91	0,88	1,79
	ACA(R(D))	0,88	0,91	1,79
ATA	ATA(D)	0,61	0,66	1,27
	TAT(RC(D))	0,66	0,61	1,27
	TAT(C(D))	0,66	0,61	1,27
	ATA(R(D))	0,61	0,61	1,21
AGA	AGA(D)	1,18	0,92	2,10
	TCT(RC(D))	0,92	1,18	2,10
	TCT(C(D))	0,92	1,18	2,10
	AGA(R(D))	1,18	0,92	2,10
AAA	AAA(D)	1,35	1,26	2,60
	TTT(RC(D))	1,26	1,35	2,60
	TTT(C(D))	1,26	1,35	2,60
	AAA(R(D))	1,35	1,26	2,60

Composition of DNA sequence in coding chromosome X

Base	No.of bases	%
A	1789548	26,87%
C	1570526	23,58%
G	1616627	24,27%
T	1683702	25,28%
N	0	0,00%
Sum	6660403	100,00%

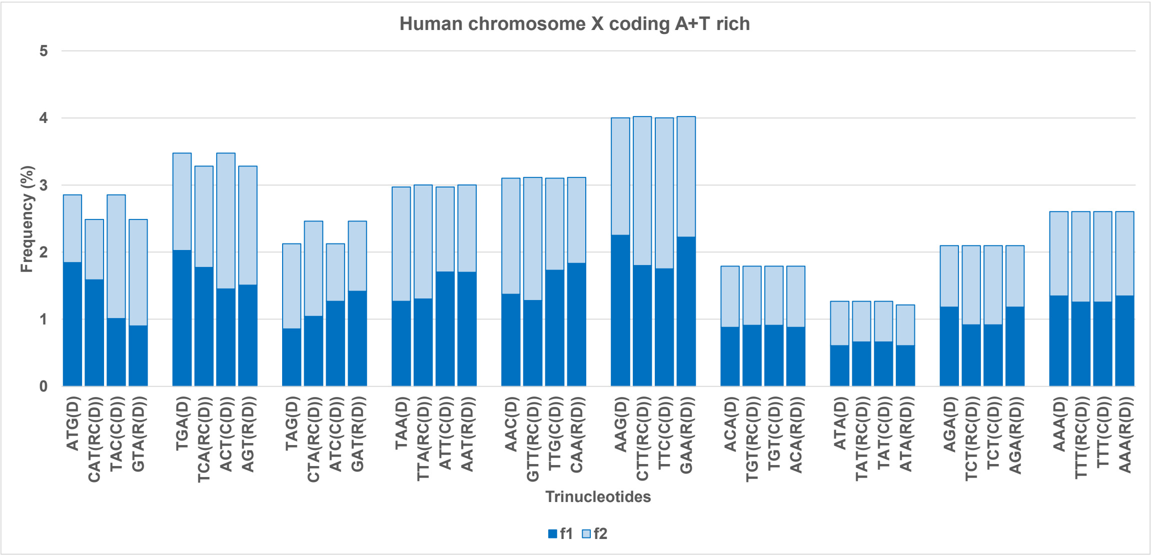

						C+G rich					
**D**	**f**	**%**	**RC**	**f**	**%**	**C**	**f**	**%**	**R**	**f**	**%**
**CGT**	358461,00	0,23	ACG	373447,00	0,24	GCA	2175151,00	1,40	TGC	2143154,00	1,38
**GTC**	1390431,00	0,90	GAC	1419285,00	0,92	CAG	2915208,00	1,88	CTG	2905793,00	1,88
**GCT**	2008064,00	1,30	AGC	2034322,00	1,31	CGA	318613,00	0,21	TCG	325067,00	0,21
**GCC**	1601755,00	1,03	GGC	1605076,00	1,04	CGG	353019,00	0,23	CCG	333997,00	0,22
**CCA**	2718716,00	1,76	TGG	2790749,00	1,80	GGT	1744219,00	1,13	ACC	1749503,00	1,13
**CCT**	2572440,00	1,66	AGG	2558632,00	1,65	GGA	2353103,00	1,52	TCC	2273893,00	1,47
**CAC**	2224865,00	1,44	GTG	2224678,00	1,44	GTG*			CAC*		
**CGC**	292616,00	0,19	GCG	290198,00	0,19	GCG*			CGC*		
**CTC**	2443443,00	1,58	GAG	2463098,00	1,59	GAG*			CTC*		
**CCC**	1808917,00	1,17	GGG	1836092,00	1,19	GGG*			CCC*		

Quadruplet		f1	f2	fu
CGT	CGT(D)	0,23	1,40	1,64
	ACG(RC(D))	0,24	1,38	1,62
	GCA(C(D))	1,40	0,23	1,64
	TGC(R(D))	1,38	0,24	1,62
GTC	GTC(D)	0,90	1,88	2,78
	GAC(RC(D))	0,92	1,88	2,79
	CAG(C(D))	1,88	0,90	2,78
	CTG(R(D))	1,88	0,92	2,79
GCT	GCT(D)	1,30	0,21	1,50
	AGC(RC(D))	1,31	0,21	1,52
	CGA(C(D))	0,21	1,30	1,50
	TCG(R(D))	0,21	1,31	1,52
GCC	GCC(D)	1,03	0,23	1,26
	GGC(RC(D))	1,04	0,22	1,25
	CGG(C(D))	0,23	1,03	1,26
	CCG(R(D))	0,22	1,04	1,25
CCA	CCA(D)	1,76	1,13	2,88
	TGG(RC(D))	1,80	1,13	2,93
	GGT(C(D))	1,13	1,76	2,88
	ACC(R(D))	1,13	1,80	2,93
CCT	CCT(D)	1,66	1,52	3,18
	AGG(RC(D))	1,65	1,47	3,12
	GGA(C(D))	1,52	1,66	3,18
	TCC(R(D))	1,47	1,65	3,12
CAC	CAC(D)	0,72	0,72	1,44
	GTG(RC(D))	0,72	0,72	1,44
	GTG(C(D))	0,72	0,72	1,44
	CAC(R(D))	0,72	0,72	1,44
CGC	CGC(D)	0,09	0,09	0,19
	GCG(RC(D))	0,09	0,09	0,19
	GCG(C(D))	0,09	0,09	0,19
	CGC(R(D))	0,09	0,09	0,19
CTC	CTC(D)	0,79	0,80	1,58
	GAG(RC(D))	0,80	0,79	1,58
	GAG(C(D))	0,80	0,79	1,58
	CTC(R(D))	0,79	0,80	1,58
CCC	CCC(D)	0,58	0,59	1,18
	GGG(RC(D))	0,59	0,58	1,18
	GGG(C(D))	0,59	0,58	1,18
	CCC(R(D))	0,58	0,59	1,18

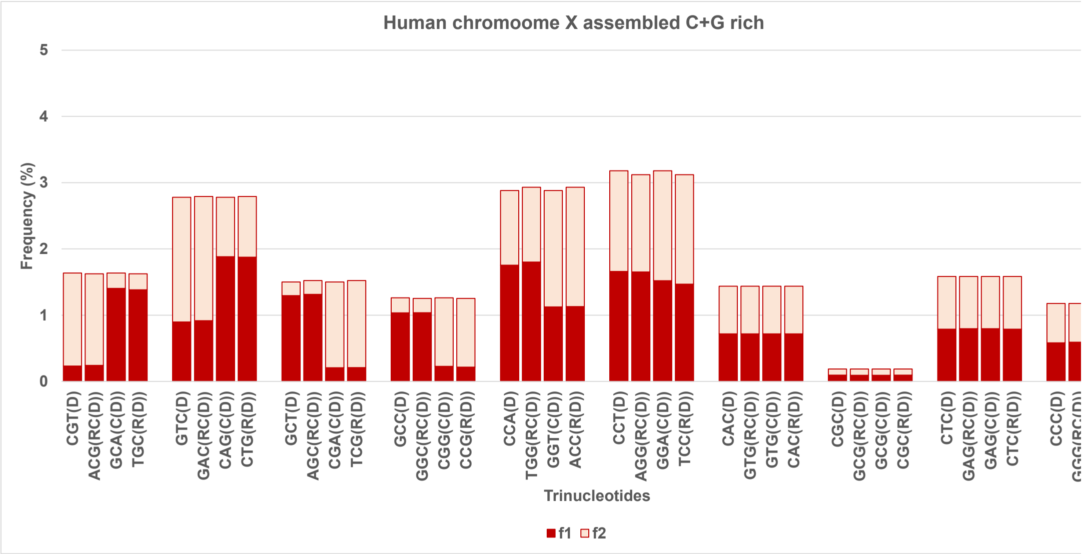

Human random chromomome X

C+G rich											
**D**	**f**	**%**	**RC**	**f**	**%**	**C**	**f**	**%**	**R**	**f**	**%**
**CGT**	548,00	0,28	ACG	603,00	0,30	GCA	2895,00	1,46	TGC	2766,00	1,40
**GTC**	1811,00	0,91	GAC	1863,00	0,94	CAG	3865,00	1,95	CTG	3933,00	1,98
**GCT**	2570,00	1,30	AGC	2665,00	1,34	CGA	463,00	0,23	TCG	458,00	0,23
**GCC**	2073,00	1,05	GGC	2116,00	1,07	CGG	500,00	0,25	CCG	442,00	0,22
**CCA**	3436,00	1,73	TGG	3636,00	1,83	GGT	2222,00	1,12	ACC	2267,00	1,14
**CCT**	3429,00	1,73	AGG	3336,00	1,68	GGA	3133,00	1,58	TCC	2967,00	1,50
**CAC**	2828,00	1,43	GTG	2974,00	1,50	GTG*			CAC*		
**CGC**	407,00	0,21	GCG	416,00	0,21	GCG*			CGC*		
**CTC**	3305,00	1,67	GAG	3219,00	1,62	GAG*			CTC*		
**CCC**	2555,00	1,29	GGG	2462,00	1,24	GGG*			CCC*		

Quadruplet		f1	f2	fu
CGT	CGT(D)	0,28	1,46	1,74
	ACG(RC(D))	0,30	1,40	1,70
	GCA(C(D))	1,46	0,28	1,74
	TGC(R(D))	1,40	0,30	1,70
GTC	GTC(D)	0,91	1,95	2,86
	GAC(RC(D))	0,94	1,98	2,92
	CAG(C(D))	1,95	0,91	2,86
	CTG(R(D))	1,98	0,94	2,92
GCT	GCT(D)	1,30	0,23	1,53
	AGC(RC(D))	1,34	0,23	1,58
	CGA(C(D))	0,23	1,30	1,53
	TCG(R(D))	0,23	1,34	1,58
GCC	GCC(D)	1,05	0,25	1,30
	GGC(RC(D))	1,07	0,22	1,29
	CGG(C(D))	0,25	1,05	1,30
	CCG(R(D))	0,22	1,07	1,29
CCA	CCA(D)	1,73	1,12	2,85
	TGG(RC(D))	1,83	1,14	2,98
	GGT(C(D))	1,12	1,73	2,85
	ACC(R(D))	1,14	1,83	2,98
CCT	CCT(D)	1,73	1,58	3,31
	AGG(RC(D))	1,68	1,50	3,18
	GGA(C(D))	1,58	1,73	3,31
	TCC(R(D))	1,50	1,68	3,18
CAC	CAC(D)	0,71	0,75	1,46
	GTG(RC(D))	0,75	0,71	1,46
	GTG(C(D))	0,75	0,71	1,46
	CAC(R(D))	0,71	0,75	1,46
CGC	CGC(D)	0,10	0,10	0,21
	GCG(RC(D))	0,10	0,10	0,21
	GCG(C(D))	0,10	0,10	0,21
	CGC(R(D))	0,10	0,10	0,21
CTC	CTC(D)	0,83	0,81	1,65
	GAG(RC(D))	0,81	0,83	1,65
	GAG(C(D))	0,81	0,83	1,65
	CTC(R(D))	0,83	0,81	1,65
CCC	CCC(D)	0,64	0,62	1,27
	GGG(RC(D))	0,62	0,64	1,27
	GGG(C(D))	0,62	0,64	1,27
	CCC(R(D))	0,64	0,62	1,27

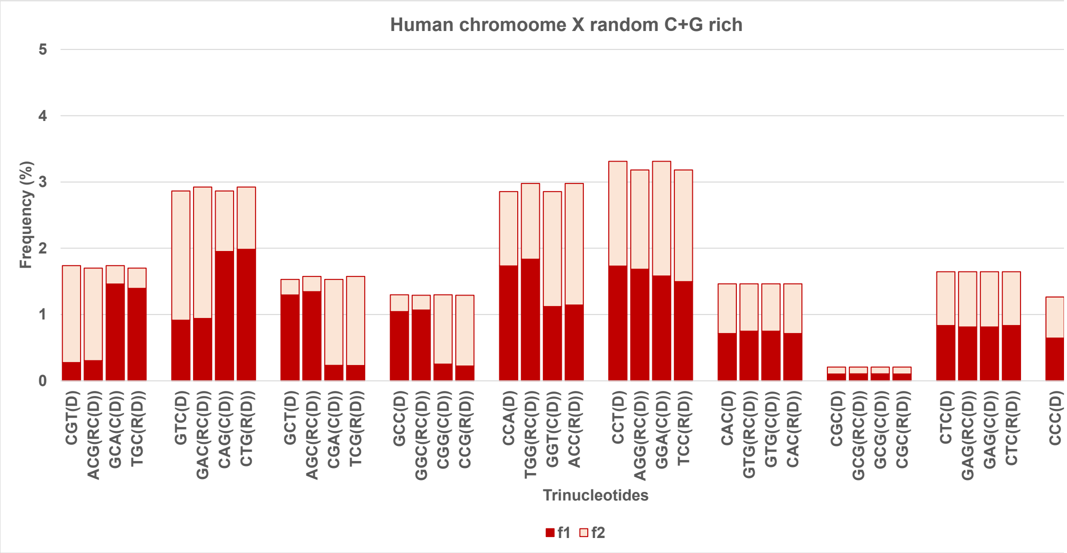

Human coding chromomome X

C+G rich

D	f	%	RC	f	%	C	f	%	R	f	%
**CGT**	30459,00	0,46	ACG	31677,00	0,48	GCA	114471,00	1,72	TGC	114927,00	1,73
**GTC**	70759,00	1,06	GAC	82932,00	1,25	CAG	165856,00	2,49	CTG	164857,00	2,48
**GCT**	113505,00	1,70	AGC	123143,00	1,85	CGA	35889,00	0,54	TCG	31139,00	0,47
**GCC**	120774,00	1,81	GGC	111050,00	1,67	CGG	48222,00	0,72	CCG	51384,00	0,77
**CCA**	143811,00	2,16	TGG	144645,00	2,17	GGT	76477,00	1,15	ACC	95853,00	1,44
**CCT**	135537,00	2,03	AGG	131578,00	1,98	GGA	136918,00	2,06	TCC	114105,00	1,71
**CAC**	99845,00	1,50	GTG	112759,00	1,69	GTG*			CAC*		
**CGC**	42399,00	0,64	GCG	42769,00	0,64	GCG*			CGC*		
**CTC**	113567,00	1,71	GAG	139660,00	2,10	GAG*			CTC*		
**CCC**	121502,00	1,82	GGG	107003,00	1,61	GGG*			CCC*		

Quadruplet		f1	f2	fu
CGT	CGT(D)	0,46	1,72	2,18
	ACG(RC(D))	0,48	1,73	2,20
	GCA(C(D))	1,72	0,46	2,18
	TGC(R(D))	1,73	0,48	2,20
GTC	GTC(D)	1,06	2,49	3,55
	GAC(RC(D))	1,25	2,48	3,72
	CAG(C(D))	2,49	1,06	3,55
	CTG(R(D))	2,48	1,25	3,72
GCT	GCT(D)	1,70	0,54	2,24
	AGC(RC(D))	1,85	0,47	2,32
	CGA(C(D))	0,54	1,70	2,24
	TCG(R(D))	0,47	1,85	2,32
GCC	GCC(D)	1,81	0,72	2,54
	GGC(RC(D))	1,67	0,77	2,44
	CGG(C(D))	0,72	1,81	2,54
	CCG(R(D))	0,77	1,67	2,44
CCA	CCA(D)	2,16	1,15	3,31
	TGG(RC(D))	2,17	1,44	3,61
	GGT(C(D))	1,15	2,16	3,31
	ACC(R(D))	1,44	2,17	3,61
CCT	CCT(D)	2,03	2,06	4,09
	AGG(RC(D))	1,98	1,71	3,69
	GGA(C(D))	2,06	2,03	4,09
	TCC(R(D))	1,71	1,98	3,69
CAC	CAC(D)	0,75	0,85	1,60
	GTG(RC(D))	0,85	0,75	1,60
	GTG(C(D))	0,85	0,75	1,60
	CAC(R(D))	0,75	0,85	1,60
CGC	CGC(D)	0,32	0,32	0,64
	GCG(RC(D))	0,32	0,32	0,64
	GCG(C(D))	0,32	0,32	0,64
	CGC(R(D))	0,32	0,32	0,64
CTC	CTC(D)	0,85	1,05	1,90
	GAG(RC(D))	1,05	0,85	1,90
	GAG(C(D))	1,05	0,85	1,90
	CTC(R(D))	0,85	1,05	1,90
CCC	CCC(D)	0,91	0,80	1,72
	GGG(RC(D))	0,80	0,91	1,72
	GGG(C(D))	0,80	0,91	1,72
	CCC(R(D))	0,91	0,80	1,72

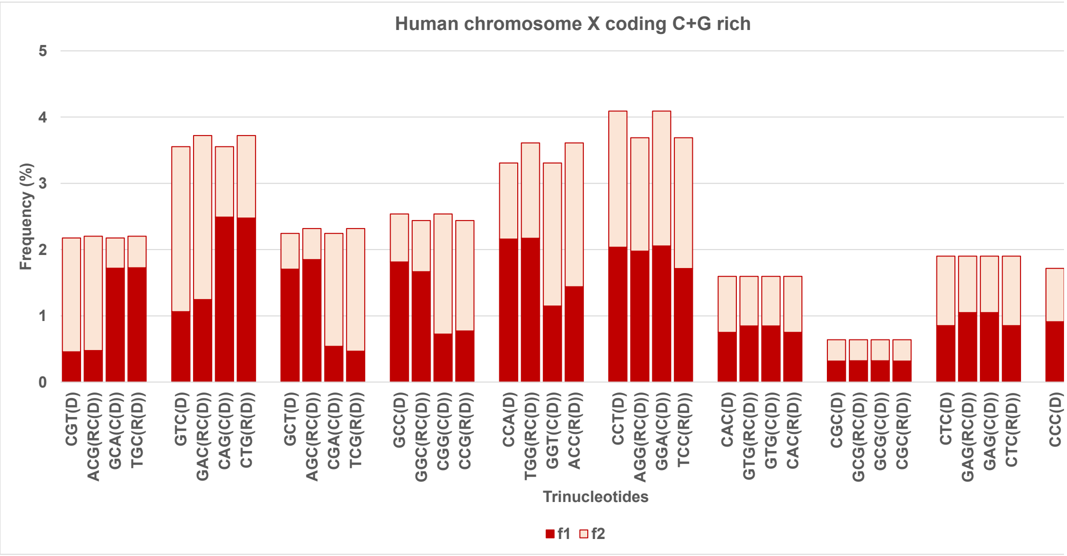

IIa

IIb

IIc

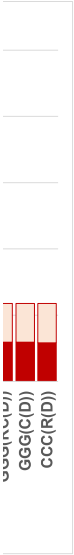

IIa

IIb

IIc

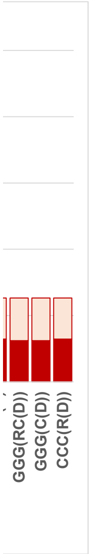

IIa

IIb

IIc

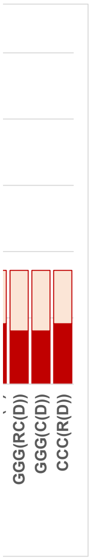
GGG(RC(D))
GGG(C(D))
CCC(R(D))

Human assembled chromomome Y

						A-T rich						
**D**	**f**	**%**	**RC**	**f**	**%**	**C**	**f**	**%**	**R**	**f**	**%**	
**ATG**	529913,00	2,01	CAT	519239,00	1,97	TAC	302379,00	1,14	GTA	303227,00	1,15	Ia
**TGA**	523758,00	1,98	TCA	522232,00	1,98	ACT	433252,00	1,64	AGT	423303,00	1,60	
**TAG**	339093,00	1,28	CTA	335512,00	1,27	ATC	360819,00	1,37	GAT	361377,00	1,37	
**TAA**	532846,00	2,02	TTA	539534,00	2,04	ATT	685338,00	2,59	AAT	691503,00	2,62	Ib
**AAC**	387745,00	1,47	GTT	400423,00	1,52	TTG	520123,00	1,97	CAA	505602,00	1,91	
**AAG**	514482,00	1,95	CTT	528668,00	2,00	TTC	554773,00	2,10	GAA	555283,00	2,10	
**ACA**	557097,00	2,11	TGT	571309,00	2,16	TGT*			ACA*			Ic
**ATA**	565978,00	2,14	TAT	569929,00	2,16	TAT*			ATA*			
**AGA**	588911,00	2,23	TCT	595131,00	2,25	TCT*			AGA*			
**AAA**	1007799,00	3,82	TTT	1029163,00	3,90	TTT*			AAA*			

Quadruplet		f1	f2	fu
ATG	ATG(D)	2,01	1,14	3,15
	CAT(RC(D))	1,97	1,15	3,11
	TAC(C(D))	1,14	2,01	3,15
	GTA(R(D))	1,15	1,97	3,11
TGA	TGA(D)	1,98	1,64	3,62
	TCA(RC(D))	1,98	1,60	3,58
	ACT(C(D))	1,64	1,98	3,62
	AGT(R(D))	1,60	1,98	3,58
TAG	TAG(D)	1,28	1,37	2,65
	CTA(RC(D))	1,27	1,37	2,64
	ATC(C(D))	1,37	1,28	2,65
	GAT(R(D))	1,37	1,27	2,64
TAA	TAA(D)	2,02	2,59	4,61
	TTA(RC(D))	2,04	2,62	4,66
	ATT(C(D))	2,59	2,02	4,61
	AAT(R(D))	2,62	2,04	4,66
AAC	AAC(D)	1,47	1,97	3,44
	GTT(RC(D))	1,52	1,91	3,43
	TTG(C(D))	1,97	1,47	3,44
	CAA(R(D))	1,91	1,52	3,43
AAG	AAG(D)	1,95	2,10	4,05
	CTT(RC(D))	2,00	2,10	4,10
	TTC(C(D))	2,10	1,95	4,05
	GAA(R(D))	2,10	2,00	4,10
ACA	ACA(D)	1,05	1,08	2,14
	TGT(RC(D))	1,08	1,05	2,14
	TGT(C(D))	1,08	1,05	2,14
	ACA(R(D))	1,05	1,08	2,14
ATA	ATA(D)	1,07	1,08	2,15
	TAT(RC(D))	1,08	1,07	2,15
	TAT(C(D))	1,08	1,07	2,15
	ATA(R(D))	1,07	1,07	2,14
AGA	AGA(D)	1,11	1,13	2,24
	TCT(RC(D))	1,13	1,11	2,24
	TCT(C(D))	1,13	1,11	2,24
	AGA(R(D))	1,11	1,13	2,24
AAA	AAA(D)	1,91	1,95	3,86
	TTT(RC(D))	1,95	1,91	3,86
	TTT(C(D))	1,95	1,91	3,86
	AAA(R(D))	1,91	1,95	3,86

Composition of DNA sequence in assembled chromosome Y

Base	No.of bases	%
A	7886192	13,78%
C	5285789	9,24%
G	5286894	9,24%
T	7956168	13,90%
N	30812372	53,84%
Sum	57227415	100,00%

% of coding bases from complete assembled chromosome 1 (including N bases)

**0,61%**

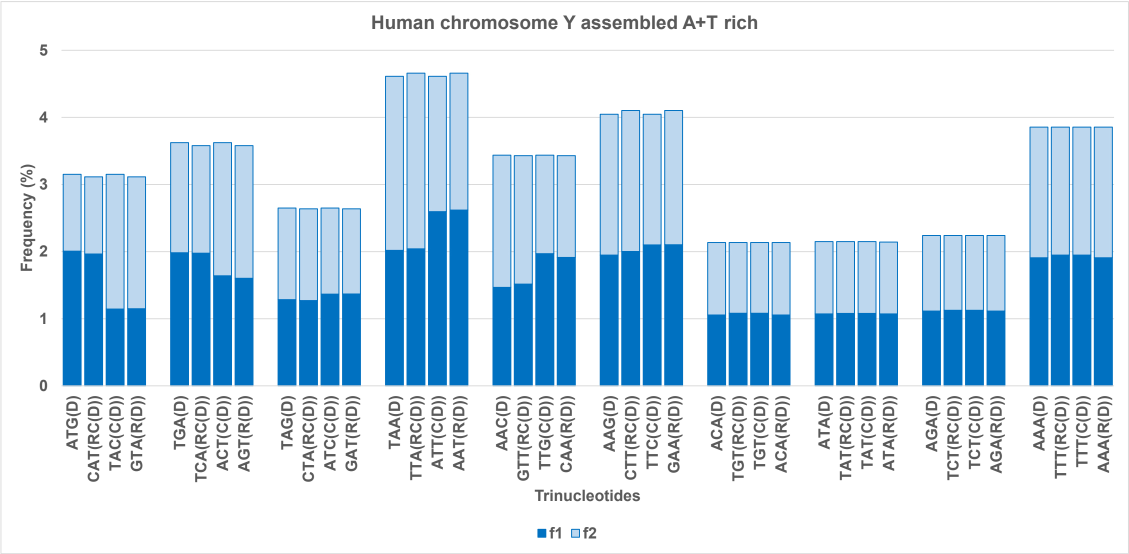

Human random chromomome Y

A-T rich

D	f	%	RC	f	%	C	f	%	R	f	%	
**ATG**	2383,00	1,46	CAT	2365,00	1,45	TAC	1171,00	0,72	GTA	1292,00	0,79	Ia
**TGA**	2620,00	1,61	TCA	2672,00	1,64	ACT	1931,00	1,19	AGT	2130,00	1,31	
**TAG**	1844,00	1,13	CTA	1811,00	1,11	ATC	1964,00	1,21	GAT	1973,00	1,21	
**TAA**	1826,00	1,12	TTA	1804,00	1,11	ATT	2406,00	1,48	AAT	2350,00	1,44	Ib
**AAC**	1647,00	1,01	GTT	1724,00	1,06	TTG	2310,00	1,42	CAA	2322,00	1,43	
**AAG**	2248,00	1,38	CTT	2487,00	1,53	TTC	2503,00	1,54	GAA	2097,00	1,29	
**ACA**	2984,00	1,83	TGT	3014,00	1,85	TGT*			ACA*			Ic
**ATA**	2305,00	1,42	TAT	2370,00	1,46	TAT*			ATA*			
**AGA**	4021,00	2,47	TCT	4422,00	2,72	TCT*			AGA*			
**AAA**	3579,00	2,20	TTT	4540,00	2,79	TTT*			AAA*			

Quadruplet		f1	f2	fu
ATG	ATG(D)	1,46	0,72	2,18
	CAT(RC(D))	1,45	0,79	2,25
	TAC(C(D))	0,72	1,46	2,18
	GTA(R(D))	0,79	1,45	2,25
TGA	TGA(D)	1,61	1,19	2,79
	TCA(RC(D))	1,64	1,31	2,95
	ACT(C(D))	1,19	1,61	2,79
	AGT(R(D))	1,31	1,64	2,95
TAG	TAG(D)	1,13	1,21	2,34
	CTA(RC(D))	1,11	1,21	2,32
	ATC(C(D))	1,21	1,13	2,34
	GAT(R(D))	1,21	1,11	2,32
TAA	TAA(D)	1,12	1,48	2,60
	TTA(RC(D))	1,11	1,44	2,55
	ATT(C(D))	1,48	1,12	2,60
	AAT(R(D))	1,44	1,11	2,55
AAC	AAC(D)	1,01	1,42	2,43
	GTT(RC(D))	1,06	1,43	2,48
	TTG(C(D))	1,42	1,01	2,43
	CAA(R(D))	1,43	1,06	2,48
AAG	AAG(D)	1,38	1,54	2,92
	CTT(RC(D))	1,53	1,29	2,81
	TTC(C(D))	1,54	1,38	2,92
	GAA(R(D))	1,29	1,53	2,81
ACA	ACA(D)	0,92	0,93	1,84
	TGT(RC(D))	0,93	0,92	1,84
	TGT(C(D))	0,93	0,92	1,84
	ACA(R(D))	0,92	0,93	1,84
ATA	ATA(D)	0,71	0,73	1,44
	TAT(RC(D))	0,73	0,71	1,44
	TAT(C(D))	0,73	0,71	1,44
	ATA(R(D))	0,71	0,71	1,42
AGA	AGA(D)	1,23	1,36	2,59
	TCT(RC(D))	1,36	1,23	2,59
	TCT(C(D))	1,36	1,23	2,59
	AGA(R(D))	1,23	1,36	2,59
AAA	AAA(D)	1,10	1,39	2,49
	TTT(RC(D))	1,39	1,10	2,49
	TTT(C(D))	1,39	1,10	2,49
	AAA(R(D))	1,10	1,39	2,49

Composition of DNA sequence in random chromosome Y

Base	No.of bases	%
A	39524	19,76%
C	41217	20,61%
G	40658	20,33%
T	41459	20,73%
N	37142	18,57%
Sum	200000	100,00%

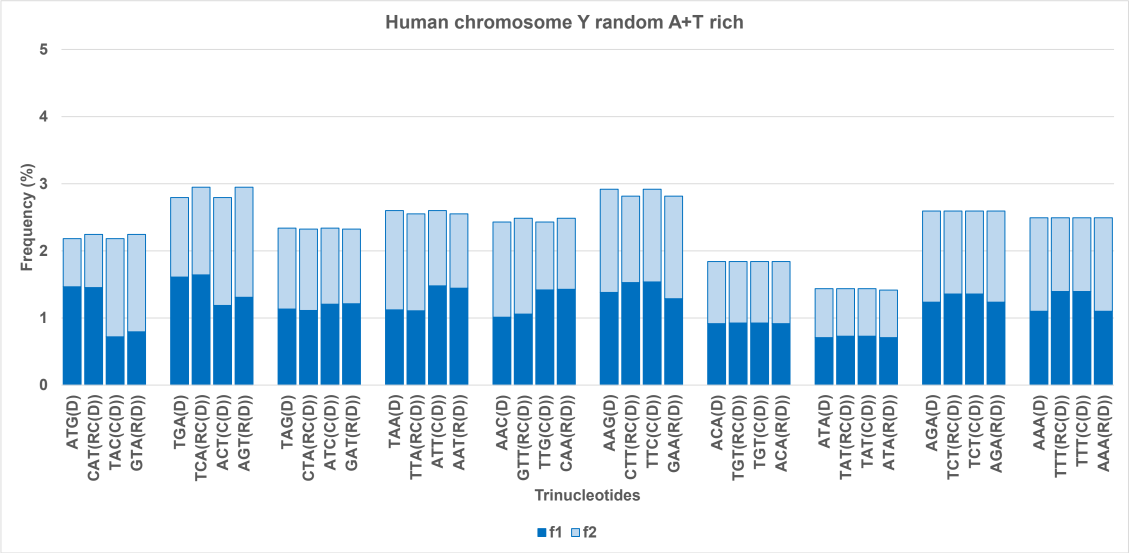

Human coding chromomome Y

A-T rich

D	f	%	RC	f	%	C	f	%	R	f	%	
**ATG**	7009,00	2,00	CAT	6034,00	1,72	TAC	4191,00	1,20	GTA	3888,00	1,11	Ia
**TGA**	7064,00	2,02	TCA	7353,00	2,10	ACT	5414,00	1,55	AGT	6117,00	1,75	
**TAG**	3497,00	1,00	CTA	4037,00	1,15	ATC	5344,00	1,53	GAT	5772,00	1,65	
**TAA**	5823,00	1,66	TTA	6323,00	1,80	ATT	7937,00	2,27	AAT	7747,00	2,21	Ib
**AAC**	5022,00	1,43	GTT	5615,00	1,60	TTG	6590,00	1,88	CAA	6868,00	1,96	
**AAG**	7986,00	2,28	CTT	6243,00	1,78	TTC	6882,00	1,96	GAA	8063,00	2,30	
**ACA**	6846,00	1,95	TGT	6973,00	1,99	TGT*			ACA*			Ic
**ATA**	6107,00	1,74	TAT	6844,00	1,95	TAT*			ATA*			
**AGA**	8201,00	2,34	TCT	5902,00	1,68	TCT*			AGA*			
**AAA**	11343,00	3,24	TTT	10241,00	2,92	TTT*			AAA*			

Quadruplet		f1	f2	fu
ATG	ATG(D)	2,00	1,20	3,20
	CAT(RC(D))	1,72	1,11	2,83
	TAC(C(D))	1,20	2,00	3,20
	GTA(R(D))	1,11	1,72	2,83
TGA	TGA(D)	2,02	1,55	3,56
	TCA(RC(D))	2,10	1,75	3,84
	ACT(C(D))	1,55	2,02	3,56
	AGT(R(D))	1,75	2,10	3,84
TAG	TAG(D)	1,00	1,53	2,52
	CTA(RC(D))	1,15	1,65	2,80
	ATC(C(D))	1,53	1,00	2,52
	GAT(R(D))	1,65	1,15	2,80
TAA	TAA(D)	1,66	2,27	3,93
	TTA(RC(D))	1,80	2,21	4,02
	ATT(C(D))	2,27	1,66	3,93
	AAT(R(D))	2,21	1,80	4,02
AAC	AAC(D)	1,43	1,88	3,31
	GTT(RC(D))	1,60	1,96	3,56
	TTG(C(D))	1,88	1,43	3,31
	CAA(R(D))	1,96	1,60	3,56
AAG	AAG(D)	2,28	1,96	4,24
	CTT(RC(D))	1,78	2,30	4,08
	TTC(C(D))	1,96	2,28	4,24
	GAA(R(D))	2,30	1,78	4,08
ACA	ACA(D)	0,98	0,99	1,97
	TGT(RC(D))	0,99	0,98	1,97
	TGT(C(D))	0,99	0,98	1,97
	ACA(R(D))	0,98	0,99	1,97
ATA	ATA(D)	0,87	0,98	1,85
	TAT(RC(D))	0,98	0,87	1,85
	TAT(C(D))	0,98	0,87	1,85
	ATA(R(D))	0,87	0,87	1,74
AGA	AGA(D)	1,17	0,84	2,01
	TCT(RC(D))	0,84	1,17	2,01
	TCT(C(D))	0,84	1,17	2,01
	AGA(R(D))	1,17	0,84	2,01
AAA	AAA(D)	1,62	1,46	3,08
	TTT(RC(D))	1,46	1,62	3,08
	TTT(C(D))	1,46	1,62	3,08
	AAA(R(D))	1,62	1,46	3,08

Composition of DNA sequence in coding chromosome Y

Base	No.of bases	%
A	102355	29,21%
C	73998	21,12%
G	76294	21,77%
T	97759	27,90%
N	0	0,00%
Sum	350406	100,00%

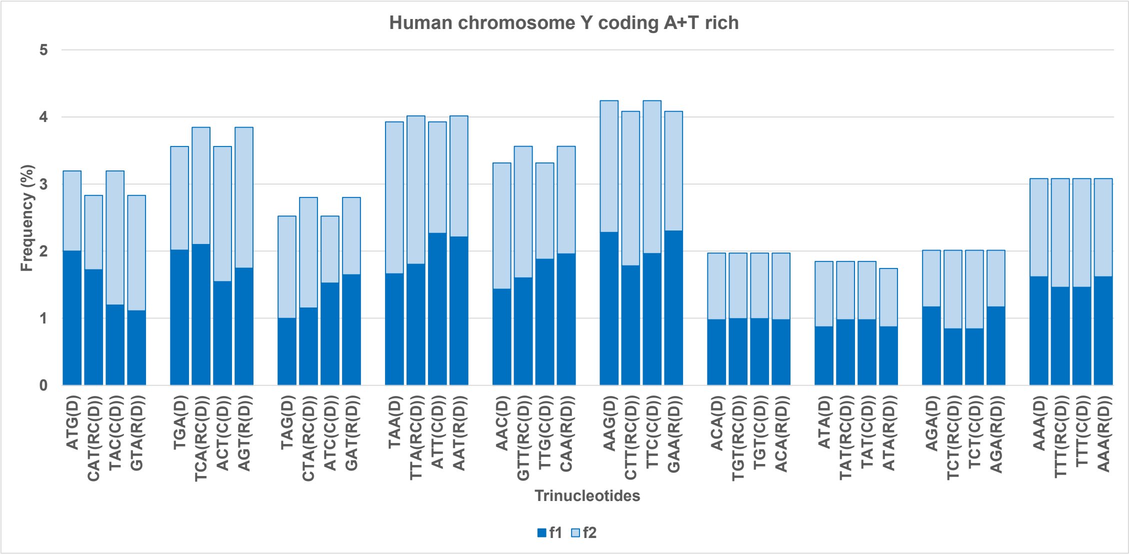

						C+G rich						
**D**	**f**	**%**	**RC**	**f**	**%**	**C**	**f**	**%**	**R**	**f**	**%**	
**CGT**	61725,00	0,23	ACG	62856,00	0,24	GCA	377684,00	1,43	TGC	376870,00	1,43	IIa
**GTC**	252778,00	0,96	GAC	252795,00	0,96	CAG	516259,00	1,95	CTG	513588,00	1,94	
**GCT**	337086,00	1,28	AGC	336779,00	1,27	CGA	55956,00	0,21	TCG	56192,00	0,21	
**GCC**	271332,00	1,03	GGC	272549,00	1,03	CGG	60934,00	0,23	CCG	59471,00	0,23	IIb
**CCA**	493846,00	1,87	TGG	496881,00	1,88	GGT	305284,00	1,16	ACC	299469,00	1,13	
**CCT**	443403,00	1,68	AGG	440914,00	1,67	GGA	420893,00	1,59	TCC	425916,00	1,61	
**CAC**	409752,00	1,55	GTG	405192,00	1,53	GTG*			CAC*			IIc
**CGC**	47297,00	0,18	GCG	47393,00	0,18	GCG*			CGC*			
**CTC**	431108,00	1,63	GAG	420069,00	1,59	GAG*			CTC*			
**CCC**	303399,00	1,15	GGG	303512,00	1,15	GGG*			CCC*			

Quadruplet		f1	f2	fu
CGT	CGT(D)	0,23	1,43	1,66
	ACG(RC(D))	0,24	1,43	1,66
	GCA(C(D))	1,43	0,23	1,66
	TGC(R(D))	1,43	0,24	1,66
GTC	GTC(D)	0,96	1,95	2,91
	GAC(RC(D))	0,96	1,94	2,90
	CAG(C(D))	1,95	0,96	2,91
	CTG(R(D))	1,94	0,96	2,90
GCT	GCT(D)	1,28	0,21	1,49
	AGC(RC(D))	1,27	0,21	1,49
	CGA(C(D))	0,21	1,28	1,49
	TCG(R(D))	0,21	1,27	1,49
GCC	GCC(D)	1,03	0,23	1,26
	GGC(RC(D))	1,03	0,23	1,26
	CGG(C(D))	0,23	1,03	1,26
	CCG(R(D))	0,23	1,03	1,26
CCA	CCA(D)	1,87	1,16	3,03
	TGG(RC(D))	1,88	1,13	3,01
	GGT(C(D))	1,16	1,87	3,03
	ACC(R(D))	1,13	1,88	3,01
CCT	CCT(D)	1,68	1,59	3,27
	AGG(RC(D))	1,67	1,61	3,28
	GGA(C(D))	1,59	1,68	3,27
	TCC(R(D))	1,61	1,67	3,28
CAC	CAC(D)	0,78	0,77	1,54
	GTG(RC(D))	0,77	0,78	1,54
	GTG(C(D))	0,77	0,78	1,54
	CAC(R(D))	0,78	0,77	1,54
CGC	CGC(D)	0,09	0,09	0,18
	GCG(RC(D))	0,09	0,09	0,18
	GCG(C(D))	0,09	0,09	0,18
	CGC(R(D))	0,09	0,09	0,18
CTC	CTC(D)	0,82	0,80	1,61
	GAG(RC(D))	0,80	0,82	1,61
	GAG(C(D))	0,80	0,82	1,61
	CTC(R(D))	0,82	0,80	1,61
CCC	CCC(D)	0,57	0,57	1,15
	GGG(RC(D))	0,57	0,57	1,15
	GGG(C(D))	0,57	0,57	1,15
	CCC(R(D))	0,57	0,57	1,15

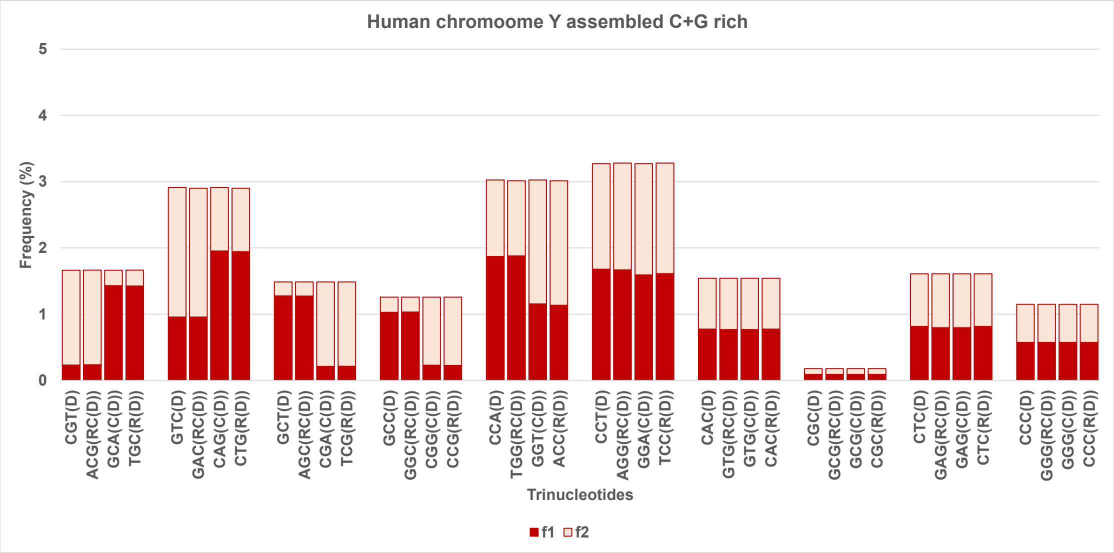

Human random chromomome Y

C+G rich												
**D**	**f**	**%**	**RC**	**f**	**%**	**C**	**f**	**%**	**R**	**f**	**%**	
**CGT**	1054,00	0,65	ACG	1089,00	0,67	GCA	2914,00	1,79	TGC	2986,00	1,83	IIa
**GTC**	2294,00	1,41	GAC	2518,00	1,55	CAG	3945,00	2,42	CTG	4036,00	2,48	
**GCT**	2376,00	1,46	AGC	2261,00	1,39	CGA	764,00	0,47	TCG	754,00	0,46	
**GCC**	2907,00	1,79	GGC	2891,00	1,78	CGG	1452,00	0,89	CCG	1486,00	0,91	IIb
**CCA**	3234,00	1,99	TGG	3318,00	2,04	GGT	2320,00	1,42	ACC	2505,00	1,54	
**CCT**	3995,00	2,45	AGG	3720,00	2,28	GGA	3278,00	2,01	TCC	3302,00	2,03	
**CAC**	3172,00	1,95	GTG	3208,00	1,97	GTG*			CAC*			IIc
**CGC**	1117,00	0,69	GCG	1058,00	0,65	GCG*			CGC*			
**CTC**	4390,00	2,70	GAG	4095,00	2,51	GAG*			CTC*			
**CCC**	3585,00	2,20	GGG	3711,00	2,28	GGG*			CCC*			

Quadruplet		f1	f2	fu
CGT	CGT(D)	0,65	1,79	2,44
	ACG(RC(D))	0,67	1,83	2,50
	GCA(C(D))	1,79	0,65	2,44
	TGC(R(D))	1,83	0,67	2,50
GTC	GTC(D)	1,41	2,42	3,83
	GAC(RC(D))	1,55	2,48	4,02
	CAG(C(D))	2,42	1,41	3,83
	CTG(R(D))	2,48	1,55	4,02
GCT	GCT(D)	1,46	0,47	1,93
	AGC(RC(D))	1,39	0,46	1,85
	CGA(C(D))	0,47	1,46	1,93
	TCG(R(D))	0,46	1,39	1,85
GCC	GCC(D)	1,79	0,89	2,68
	GGC(RC(D))	1,78	0,91	2,69
	CGG(C(D))	0,89	1,79	2,68
	CCG(R(D))	0,91	1,78	2,69
CCA	CCA(D)	1,99	1,42	3,41
	TGG(RC(D))	2,04	1,54	3,58
	GGT(C(D))	1,42	1,99	3,41
	ACC(R(D))	1,54	2,04	3,58
CCT	CCT(D)	2,45	2,01	4,47
	AGG(RC(D))	2,28	2,03	4,31
	GGA(C(D))	2,01	2,45	4,47
	TCC(R(D))	2,03	2,28	4,31
CAC	CAC(D)	0,97	0,98	1,96
	GTG(RC(D))	0,98	0,97	1,96
	GTG(C(D))	0,98	0,97	1,96
	CAC(R(D))	0,97	0,98	1,96
CGC	CGC(D)	0,34	0,32	0,67
	GCG(RC(D))	0,32	0,34	0,67
	GCG(C(D))	0,32	0,34	0,67
	CGC(R(D))	0,34	0,32	0,67
CTC	CTC(D)	1,35	1,26	2,61
	GAG(RC(D))	1,26	1,35	2,61
	GAG(C(D))	1,26	1,35	2,61
	CTC(R(D))	1,35	1,26	2,61
CCC	CCC(D)	1,10	1,14	2,24
	GGG(RC(D))	1,14	1,10	2,24
	GGG(C(D))	1,14	1,10	2,24
	CCC(R(D))	1,10	1,14	2,24

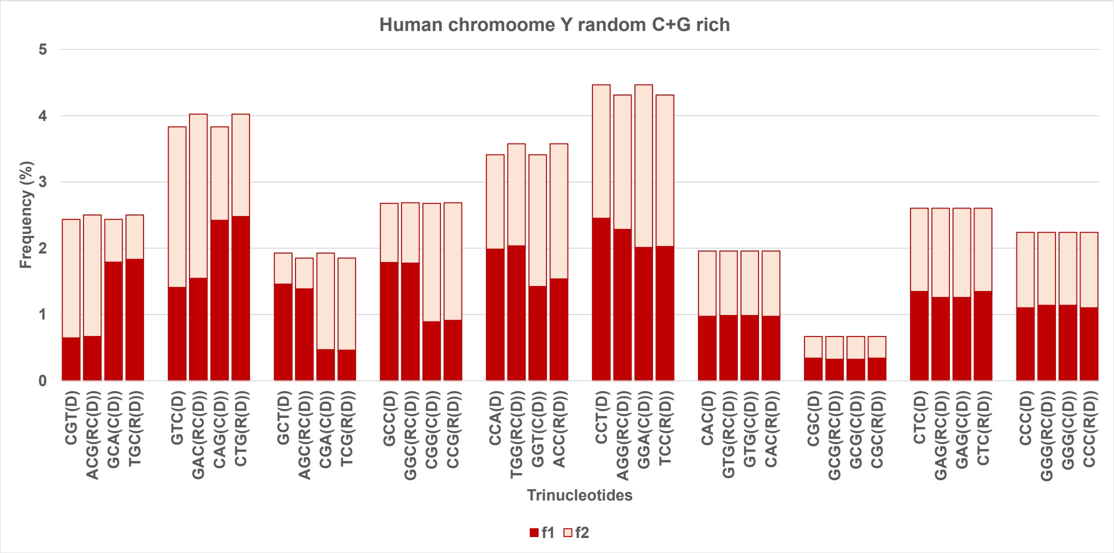

Human coding chromomome Y

C+G rich												
**D**	**f**	**%**	**RC**	**f**	**%**	**C**	**f**	**%**	**R**	**f**	**%**	
**CGT**	1505,00	0,43	ACG	1394,00	0,40	GCA	5712,00	1,63	TGC	5853,00	1,67	IIa
**GTC**	3674,00	1,05	GAC	3657,00	1,04	CAG	8688,00	2,48	CTG	8018,00	2,29	
**GCT**	5379,00	1,54	AGC	5464,00	1,56	CGA	1554,00	0,44	TCG	1711,00	0,49	
**GCC**	4612,00	1,32	GGC	4394,00	1,25	CGG	1534,00	0,44	CCG	1480,00	0,42	IIb
**CCA**	7108,00	2,03	TGG	7114,00	2,03	GGT	3969,00	1,13	ACC	4645,00	1,33	
**CCT**	6067,00	1,73	AGG	5779,00	1,65	GGA	6063,00	1,73	TCC	5398,00	1,54	
**CAC**	5429,00	1,55	GTG	5387,00	1,54	GTG*			CAC*			IIc
**CGC**	1499,00	0,43	GCG	1507,00	0,43	GCG*			CGC*			
**CTC**	4464,00	1,27	GAG	5390,00	1,54	GAG*			CTC*			
**CCC**	3470,00	0,99	GGG	3210,00	0,92	GGG*			CCC*			

Quadruplet		f1	f2	fu
CGT	CGT(D)	0,43	1,63	2,06
	ACG(RC(D))	0,40	1,67	2,07
	GCA(C(D))	1,63	0,43	2,06
	TGC(R(D))	1,67	0,40	2,07
GTC	GTC(D)	1,05	2,48	3,53
	GAC(RC(D))	1,04	2,29	3,33
	CAG(C(D))	2,48	1,05	3,53
	CTG(R(D))	2,29	1,04	3,33
GCT	GCT(D)	1,54	0,44	1,98
	AGC(RC(D))	1,56	0,49	2,05
	CGA(C(D))	0,44	1,54	1,98
	TCG(R(D))	0,49	1,56	2,05
GCC	GCC(D)	1,32	0,44	1,75
	GGC(RC(D))	1,25	0,42	1,68
	CGG(C(D))	0,44	1,32	1,75
	CCG(R(D))	0,42	1,25	1,68
CCA	CCA(D)	2,03	1,13	3,16
	TGG(RC(D))	2,03	1,33	3,36
	GGT(C(D))	1,13	2,03	3,16
	ACC(R(D))	1,33	2,03	3,36
CCT	CCT(D)	1,73	1,73	3,46
	AGG(RC(D))	1,65	1,54	3,19
	GGA(C(D))	1,73	1,73	3,46
	TCC(R(D))	1,54	1,65	3,19
CAC	CAC(D)	0,77	0,77	1,54
	GTG(RC(D))	0,77	0,77	1,54
	GTG(C(D))	0,77	0,77	1,54
	CAC(R(D))	0,77	0,77	1,54
CGC	CGC(D)	0,21	0,22	0,43
	GCG(RC(D))	0,22	0,21	0,43
	GCG(C(D))	0,22	0,21	0,43
	CGC(R(D))	0,21	0,22	0,43
CTC	CTC(D)	0,64	0,77	1,41
	GAG(RC(D))	0,77	0,64	1,41
	GAG(C(D))	0,77	0,64	1,41
	CTC(R(D))	0,64	0,77	1,41
CCC	CCC(D)	0,50	0,46	0,95
	GGG(RC(D)	0,46	0,50	0,95
	GGG(C(D))	0,46	0,50	0,95
	CCC(R(D))	0,50	0,46	0,95

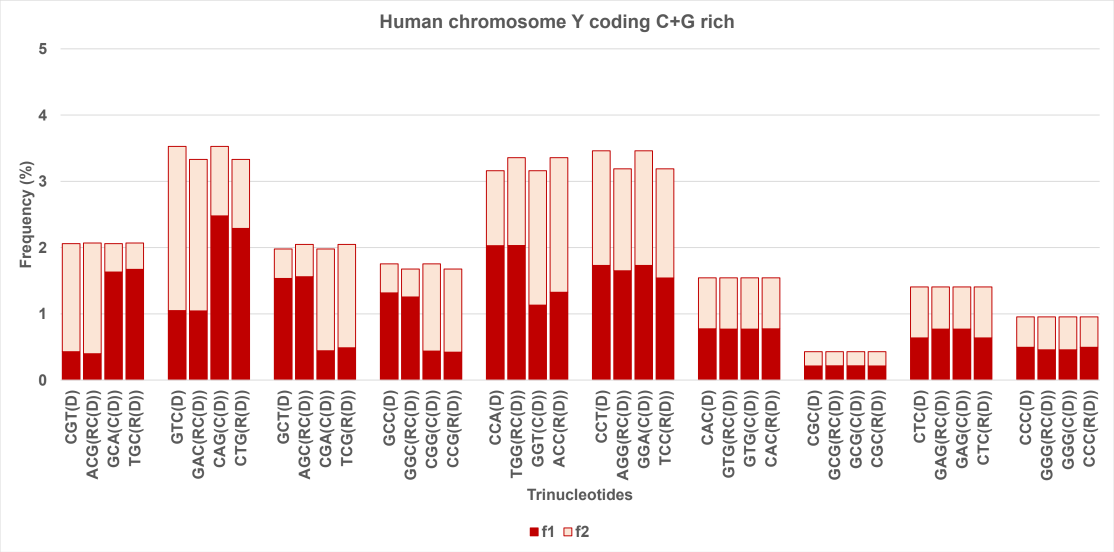

Supplement: Supplementary file 1 [file genes-13-01929-s001.zip › Supplemetary Table S9.pdf]
